# Prognostic and Predictive Models for Left- and Right- Colorectal Cancer Patients: A Bioinformatics Analysis Based on Ferroptosis-Related Genes

**DOI:** 10.3389/fonc.2022.833834

**Published:** 2022-02-21

**Authors:** Yingying Chen, Hua Li

**Affiliations:** Department of Gastrointestinal Oncology (Ward I), The First Affiliated Hospital of Jinzhou Medical University, Jinzhou, China

**Keywords:** The Cancer Genome Atlas (TCGA), colorectal cancer (CRC), left- and right-sided, prognosis, ferroptosis-related genes (FRGs)

## Abstract

**Background:**

Left- and right-sided colorectal cancer (LCRC, RCRC) are significantly different in epidemiology and clinical manifestations and have altered outcomes. However, as a hot tumor prognostic marker, the role of ferroptosis-related genes (FRGs) in LCRC and RCRC is unknown.

**Methods:**

From The Cancer Genome Atlas (TCGA) database, we downloaded the expression profiles of CRC patients. A “DESeq2” package was performed to compare the differentially expressed genes (DEGs) of LCRC and RCRC. FRGs were identified using the FerrDb. The prognostic value of differentially expressed FRG (DE-FRG) in left- and right-CRC was assessed separately by Cox regression analysis. Subsequently, functional enrichment analysis, ESTIMATE, and single sample Gene Set Enrichment Analysis (ssGSEA) were performed based on LCRC and RCRC samples to reveal the potential function of FRGs-related risk signatures. The differential expression of FRGs in tumor tissues and adjacent normal tissues were verified by Western blot. The differential expression and prognosis in LCC and RCC were verified by immunohistochemistry.

**Results:**

Based on the identified 14 DE-FRGs, the LCRC prognostic model consisted of *NOS2* and *IFNG*; *NOS2* and *ALOXE* established the prognostic signature that could distinguish RCRC outcomes. In the functional analysis, the DEGs (high risk vs. low risk) of the LCRC and RCRC were significantly enriched in the immune- and lipid-related terms and pathways. ESTIMATE and ssGSEA suggested that these FRGs-related risk signatures were affiliated with the infiltration of immune cell subtypes. Western blotting results showed that NOS2 and ALOXE3 were significantly highly expressed in cancer, and the difference was statistically significant (P < 0.05). Immunohistochemical results showed that ALOXE3 was highly expressed in RCC, and those with high expression had a worse prognosis, while NOS2 gene had an effect on the prognosis of both LCC and RCC.

**Conclusion:**

This study constructed a potential prognostic model of LCRC and RCRC, respectively. We also identified the crucial pathways that contribute to elucidating the pathogenesis of CRC.

## 1 Introduction

Colorectal cancer (CRC) is the third most common cancer worldwide and also a fatal disease. Although the mortality rate of CRC has been declining since 1990, it still remains at approximately 1.7-1.9% (1). According to the origin of the lesion, the disease can be divided into right-sided colorectal cancer (RCRC) and left-sided colorectal cancer (LCRC) (2). RCRC derives from the midgut including the cecum, ascending colon, and transverse colon. In contrast, LCRC derives from the hindgut mainly composed of splenic flexure, rectum, descending colon and sigmoid colon (3). In recent years, the difference between LCRC and RCRC has attracted increasing attention.

Studies have shown evident difference between LCRC and RCRC in terms of epidemiology, pathology, clinical manifestations, survival rates and gene mutations. In the 1990s, published research showed that the 5-year overall survival (OS) of LCRC and RCRC were different, namely 56.3% and 59.7% (4). In 2000, the rates increased to 67% and 71%, respectively (*p* < 0.01) (5). This change may be attributed to the development of adjuvant and palliative chemotherapy in the treatment of CRC. A previous study reported that the disease-free survival rate for LCRC and RCRC after radical surgery is similar (6), and the survival benefit from adjuvant chemotherapy is affected by the stage and tumor location. For stage II CRC, adjuvant chemotherapy cannot improve the OS for either LCRC or RCRC but for stage III CRC, it can reduce the risk of death for LCRC and RCRC by 36% and 39%, respectively (7). After palliative chemotherapy, the survival time of metastatic LCRC is longer than that of RCRC. There are more adverse prognostic factors for RCRC, including poor differentiation, late stage, and aggressive histological types, which lead to poor treatment outcomes in patients with RCRC (8–10). But so far, the specific mechanism of the huge difference between LCRC and RCRC is still unclear.

Ferroptosis is an iron-dependent form of nonapoptotic cell death, which is driven by excessive accumulation of lipid peroxides (11). In recent years, iron-induced cell death has become a promising treatment that can trigger cancer cell death, especially for patients with malignant tumors that are resistant to traditional therapies (12, 13). At present, there is no report clearly pointing out that FRGs have a prognostic role in LCRC and RCRC.

The Cancer Genome Atlas (TCGA) is a public funded project that aims to provide public available datasets (14). In this study, we obtained the transcriptome and corresponding clinical information of CRC samples from TCGA database. Through univariate and multivariate Cox regression analyses, gene signatures with strong prognostic efficacy were constructed for LCRC and RCRC respectively. At the same time, we explored the correlation between risk score and the clinical characteristics of LCRC and RCRC. Unfortunately, there seems no close correlation between the two. Additionally, we analyzed the relationship between FRG-related risk signatures and immune cell infiltration in LCRC and RCRC by ssGSEA and ESTIMATE analyses. Moreover, the differentially expressed genes (DEGs) in LCRC and RCRC were screened for the first time based on the high- and low-risk groups. Following Kyoto encyclopedia of genes and genomes (KEGG) and gene ontology (GO) enrichment analyses, the DEGs between the high- and low-risk groups were found to be involved in the peroxisome proliferator-activated receptor (PPAR) signaling pathway. This study aims to elucidate the effect of FRGs on the prognosis of LCRC and RCRC, and provide novel prognostic markers NOS2, IFNG and ALOXE3.

## 2 Materials and Methods

### 2.1 Data Sources

Clinical information, mutation profiles, and mRNA expression data for CRC patients were available for download from TCGA (https://portal.gdc.cancer.gov/). Among them, there were 497 samples of transcriptome data, 41 normal samples, and 456 tumor samples. According to the ‘site of resection or biopsy’ information, we classified patients from the hind intestine (including splenic flexure, rectum, descending colon, and sigmoid colon) as LCRC patients (n = 228), while the lesions of the patient including the cecum, ascending colon, and transverse colon were identified as the RCRC (n = 198).

FRGs were obtained using FerrDb (http://www.zhounan.org/ferrdb/) to identify prognostically relevant FRGs in CRC.

### 2.2 DEGs Analyses

In our study, the ‘edgeR’ package was employed to analyze tissue samples from TCGA-CRC dataset, including LCRC vs. RCRC, the high-risk group in LCRC vs. low-risk group in LCRC, and high-risk group in RCRC vs. low-risk group in RCRC (14). Genes fulfilling *P* < 0.05 and |log_2_ fold change (FC)| > 0.5 were deemed to be DEGs (LCRC vs. RCRC) **(** **Supplementary Table 1** **)**. The overlapping genes between DEGs (LCRC vs. RCRC) and the above FRGs were then identified as DE-FRG. While the choice criterion for the DEGs (high-risk group in LCRC/RCRC vs. low-risk group in LCRC/RCRC) contained the *P* < 0.05 and |log_2_FC| > 1 **(** **Supplementary Tables 2**, **3** **)**.

### 2.3 Structuring and Validating Risk Scoring System

Here, to ensure the availability of CRC samples, 9 patients with lack of survival data and a survival time of 0 were excluded from the LCRC (n=228), and a total of 219 LCRC patients were used in the follow-up analysis; for right-sided CRC patients, 12 of 198 samples with missing survival information and a survival time of 0 were excluded, and the remaining 186 were included in the follow-up analysis. Simultaneously, using the ‘set.seed’ R package, LCRC patients were randomly divided into a training cohort (n = 153) and a testing cohort (n = 66) based on a 7:3 ratio; similarly, the RCRC samples were randomly divided into a training cohort of 130 and a validation cohort of 56 samples as described above.

To construct the risk signature based on DE-FRGs, univariate Cox regression analysis was used to evaluate the association between individual DE-FRGs and patients’ OS in the LCRC and RCRC training cohorts. Then, those DE-FRGs that were significant (*P* < 0.2) were combined in a stepwise multivariate Cox regression analysis to determine the best variables for constructing the risk signature. Risk scores (15)were defined as follows:


$$risk\;score=\frac{{\text{e}}^{\text{sum}(\text{each gene's expression levels}\times \text{corresponding coefficient})}}{{\text{e}}^{\text{sum}(\text{each gene's mean expression levels}\times \text{corresponding coefficient})}}$$

Risk scores were calculated for each sample in the training, testing, and overall cohorts, and the median risk score in the respective cohort was used as a cut-off value to classify the samples into high- and low-risk groups. The Kaplan-Meier curves performed by the ‘survival’ R package were used to compare the OS of patients in the high- and low-risk groups. Besides, ROC curve analysis was performed with the ‘timeROC’ R package to enable the assessment of the prognostic efficacy of two DE-FRG-based risk signatures.

### 2.4 Construction of the Nomogram

We integrated risk scores and clinical indicators into univariate and multivariate Cox regression analyses to identify independent prognostic factors for CRC. Nomograms were then plotted to construct nomogram models to assess their predictive power for the prognosis of CRC patients. By drawing a calibration chart and the clinical decision curve analysis (DCA) to evaluate the predictive effect of the nomogram. Also, correlations between patient age, gender, ajcc pathologic t, ajcc pathologic n, ajcc pathologic m, ajcc pathologic stage, and DE-FRGs-related risk score were assessed in the overall LCRC and RCRC patient cohorts.

### 2.5 Functional Enrichment Analysis

We conducted the Gene Ontology (GO) and the Kyoto Encyclopedia of Genes and Genomes (KEGG) analyses for DEGs (high-risk group in LCRC/RCRC vs. low-risk group in LCRC/RCRC) with ‘clusterProfiler’, ‘ggplot2’, and ‘enrichplot’ packages. *P* < 0.05 was set as the cut-off criterion for the significant enrichment.

### 2.6 Immune Landscape Analysis Based on Prognostic Signatures

The ESTIMATE algorithm estimates the proportion of immune cells and stromal cells in the TME of each sample in the form of three scores (ImmuneScore, StromalScore and ESTIMATEScore). The higher the score, the greater the proportion of the corresponding component in the TME of that sample. Besides, ssGSEA was utilized to characterize the extent of infiltration of 28 immune cell subtypes between high- and low-risk groups. The *P* < 0.05 indicated statistical significance.

### 2.7 Main Reagents

NOS2 antibody (Biogot, WB dilution 1:500, IHC-P dilution 1:100), ALOXE3 antibody (Biogot, WB dilution 1:1000), ALOXE3 (Bioss, IHC-P dilution 1:200), β-actin antibody (Biogot, dilution 1:5000), tissue protein extraction kit (Bestbio), animal tissue RNA stable preservation solution (Beyotime), BCA protein concentration assay kit (Beyotime), high-sensitivity ECL chemiluminescence kit, universal SP kit (ZSGB-BIO), DAB chromogenic kit (ZSGB-BIO).

### 2.8 Western Blotting and Immunohistochemistry

#### 2.8.1 Western Blotting

The required tissue homogenate was extracted by tissue protein extraction kit, lysed on ice throughout, centrifuged at 12 000 r/min for 10 min at 4°C, and the supernatant was collected, that is, the required total protein. After protein quantification by BCA, it was mixed with 5 × protein buffer, boiled in a bath for 5 min, and dispensed and stored in a − 20°C refrigerator. Proteins were separated by SDS-PAGE gel electrophoresis, then transferred to PVDF membranes for 2 h, blocked with 5% skim milk at room temperature for 1 h, and incubated with primary antibodies at 4° C overnight. Every other day the membranes were washed with TBST three times and incubated with secondary antibodies for 1h, then washed three times again and detected by autoradiography.

#### 2.8.2 Expression Quantity Detected by Immunohistochemistry

Pathological sections were baked at 60°C for 2 h. They were successively deparaffinized in xylene, gradient alcohol, double-distilled water, PBS solution, and repaired at high temperature in a microwave oven. Then the sections were bathed in blocking agent of the universal SP kit, and incubated at 4°C overnight. Subsequently, washed with PBS three times and the secondary antibody was applied for 15 min. Then, sections were developed with horseradish peroxidase (HRP) and diaminobenzidine (DAB), counterstained with hematoxylin, differentiated with hydrochloric acid ethanol, rinsed with running water, and then hydrated with gradient alcohol and xylene, and mounted with neutral balsam. After that, a microscopic examination was performed.

### 2.9 Statistical Analysis

Statistical significance for variables between two groups or more than two groups was estimated by t-tests, Wilcoxon tests, or Kruskal-Wallis respectively. All statistical analyses were performed with R software. R is a language and environment for statistical computing and graphics. It is a GNU project which is similar to the S language and environment which was developed at Bell Laboratories (formerly AT&T, now Lucent Technologies) by John Chambers and colleagues (https://www.r-project.org/about.html). Statistical significance was set at probability values of *P* < 0.05. Statistical analysis was performed with the statistical software package SPSS 25.0, Graphpad Prism 9.0 and imageJ. Kaplan-Meier survival analysis and the Log rank test were used to plot the survival curve and compare the survival time, test level α = 0.05. The comparison between the means of two independent samples was performed using the t-test; ANOVA was performed to compare the differences in means between multiple groups, and P < 0.05 was considered statistically significant.

## 3 Results

### 3.1 Identification of DE-FRGs Between LCRC and RCRC

**Figure 1:**
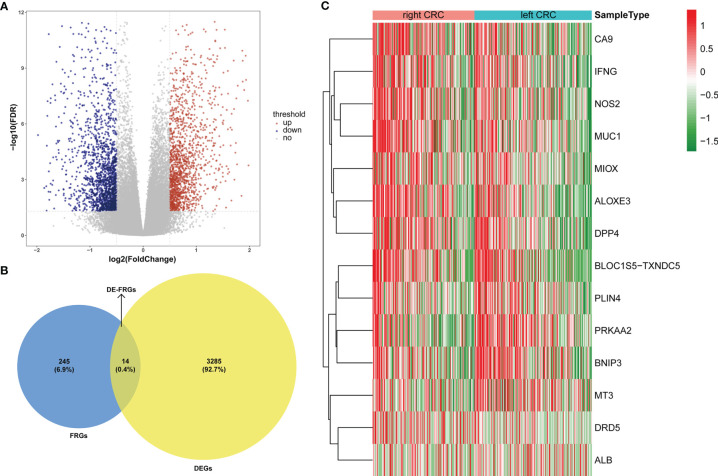
Expression pattern of FRGs between LCRC and RCRC patients. **(A)** The volcano plots of 3299 DEGs between LCRC and RCRC samples in TCGA database, with the cut-off criteria of |log2 FC| > 0.5 and P < 0.05. Red plots: upregulation; blue plots: downregulation; gray plots: normally expressed genes. **(B)** The Venn diagram shows the intersecting genes from DEGs and FRGs. Blue area: FRGs; yellow area: DEGs; cross area: DE-FRGs. **(C)** The heatmap of 14 DE-FRGs between LCRC and RCRC groups. Genes with higher expression were shown in red, while lower expressions were shown in green.

For the TCGA data, gene expression in LCRC was compared with that in RCRC. A total of 3299 DEGs were identified (|log_2_FC| > 0.5, *P*  <  0.05), which included 1533 upregulated and 1766 downregulated genes **(** **Figure 1A** **)**. 14 DE-FRGs overlapped across the TCGA gene expression series and FRGs set **(** **Figure 1B** **)**, including 9 genes (containing *CA9*, *IFNG*, *NOS2*, *MUC1*, *MIOX*, *ALOXE3*, *DPP4*, *BLOC1S5-TXNDC5*, and *DRD5*) highly expressed in RCRC and 5 genes (containing *PLIN4*, *PRKAA2*, *BNIP3*, *MT3*, and *ALB*) highly expressed in LCRC **(** **Table 1** **)**. Subsequently, the expression values of DE-FRGs were hierarchically clustered, and the result was presented in the form of a heatmap **(** **Figure 1C** **)**.

**Table 1:** 14 DE-FRGs expression results in left- and right- colorectal cancer.

	Base mean	log2FoldC hange	lfcSE	stat	pvalue	padj	Threshold (right vs. left)
CA9	1883.0967	0.87991	0.197256	4.46076	8.17E-06	0.000211	up
IFNG	12.450558	0.784518	0.176403	4.447315	8.70E-06	0.000222	up
NOS2	2272.772	0.928535	0.167954	5.528518	3.23E-08	2.20E-06	up
MUC1	6059.4166	0.660524	0.13606	4.854639	1.21E-06	4.34E-05	up
MIOX	14.783479	1.346227	0.193265	6.965715	3.27E-12	8.67E-10	up
ALOXE3	11.431155	0.898554	0.178943	5.021467	5.13E-07	2.17E-05	up
DPP4	1430.9574	0.657883	0.137316	4.791011	1.66E-06	5.62E-05	up
OC1S5-TXND	3.106773	0.530071	0.162195	3.268106	0.001083	0.009689	up
DRD5	10.093523	1.900199	0.335701	5.660392	1.51E-08	1.20E-06	up
ALB	19.744024	-1.22273	0.33147	-3.6888	0.000225	0.002889	down
PLIN4	169.04116	-0.56343	0.205693	-2.73915	0.00616	0.035176	down
PRKAA2	69.944021	-1.11148	0.208614	-5.32794	9.93E-08	5.55E-06	down
BNIP3	520.14065	-0.52306	0.135256	-3.86719	0.00011	0.00165	down
MT3	53.456813	-1.27938	0.213792	-5.98422	2.17E-09	2.30E-07	down

### 3.2 Identification of Prognosis Signatures for LCRC and RCRC

We randomly divided 219 LCRC samples and the corresponding clinical data into a training set (n = 153) and a test set (n = 66) according to the ratio of 7:3. Similarly, 186 RCRC samples were also randomly divided into a training set (n = 130) and a test set (n = 56).

**Figure 2:**
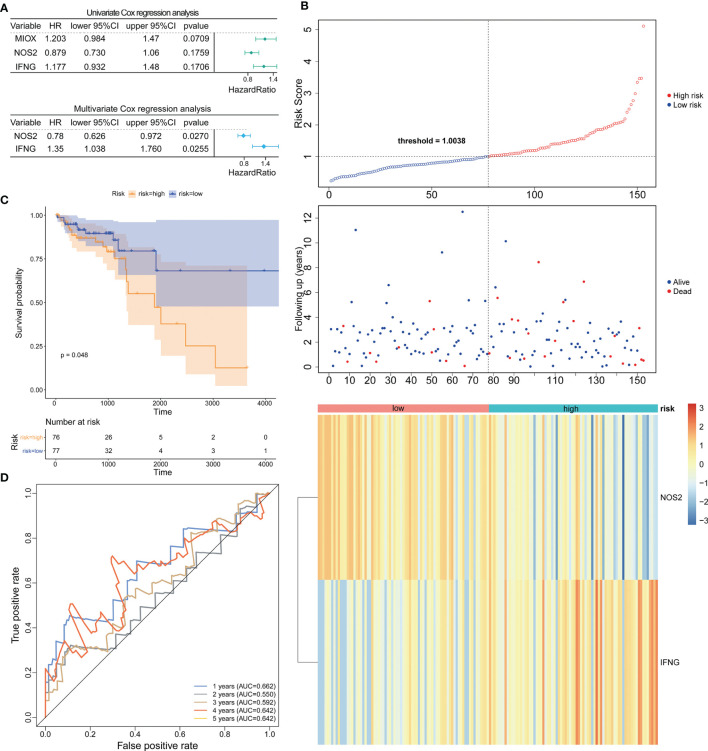
Construction and evaluation of NOS2 and IFNG-based prognostic signature in the LCRC-training set. **(A)** Results of the univariate (top) and multivariate (bottom) Cox regression analysis regarding OS in the LCRC-training cohort. **(B)** The risk curve based on the risk score of each sample (top). The scatterplot based on the survival status of each sample (middle). The green and red dots represent survival and death, respectively. The heatmap displayed the expression levels of DE-FRGs in the high‐risk and low‐risk groups (bottom). **(C)** Kaplan‐Meier survival analysis of the high‐risk and low‐risk groups based on the risk model and median risk score. **(D)** AUC of time-dependent ROC curves verified the prognostic performance of the risk score in the LCRC-training cohort.

**Figure 3:**
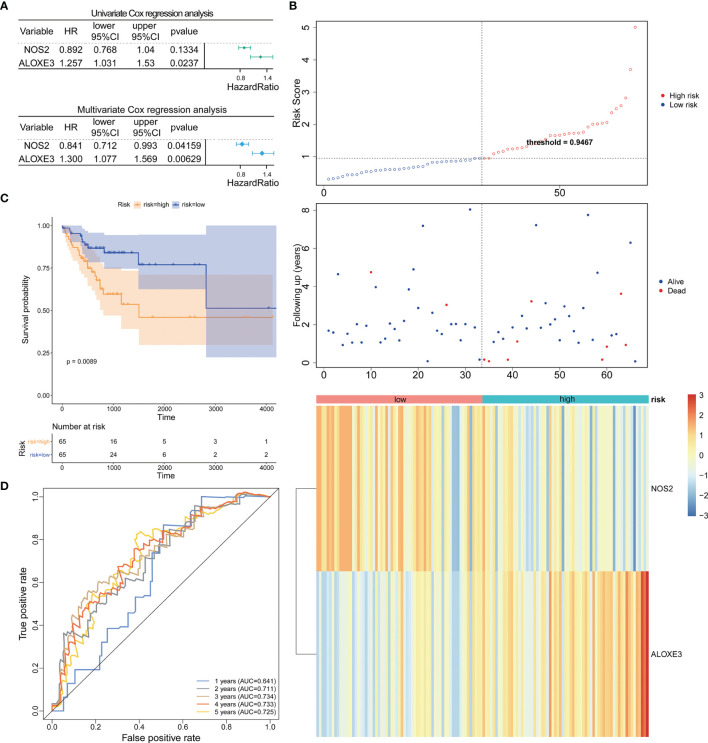
Prognostic analysis of the 2-gene signature model in the RCRC-training cohort. **(A)** Forest plot of the relationship between DE-FRGs and OS of patients with RCRC by univariate (top) and multivariate (bottom) Cox regression analysis. **(B)** The Distributions of risk scores (top), survival statuses of patients in low‐risk and high‐risk groups (middle), and two‐gene expression profiles of each patient (bottom). **(C)**. Kaplan-Meier curves for the OS of patients in the high-risk group and low-risk group in the RCRC-training cohort. **(D)** The ROC analysis of RCRC-training cohort for survival prediction by DE-FRGs-based signature.

Based on the TCGA-CRC database, we performed univariate Cox regression analysis in the LCRC-training set and RCRC-training set for the 14 DE-FRGs mentioned above to investigate whether these genes were associated with OS in LCRC/RCRC patients (*P* < 0.2). The results showed that *MIOX*, *NOS2*, and *IFNG* among the 14 DE-FRGs were associated with OS in LCRC patients; for RCRC patients, *NOS2* and *ALOXE3* were the two DE-FRGs associated with their OS. Subsequently, we implemented a stepwise regression multivariate Cox analysis in the LCRC- and RCRC-training sets based on the identified DE-FRGs associated with OS in LCRC/RCRC patients to screen the best DE-FRGs for constructing prognostic models for LCRC and RCRC. The results showed that the best DE-FRGs for predicting OS in LCRC patients were *NOS2* and *IFNG* (*P* < 0.05); the optimal prognostic genes for RCRC were *NOS2* and *ALOXE3* (*P* < 0.05). The results of univariate and multivariate Cox regression analyses in the LCRC-training set and RCRC-training set were presented as forest plots and could be reviewed in **Figures 2A**, **3A**.

Expression data of these genes were introduced into the risk score equation mentioned in the methods. According to the median value of the risk score, the LCRC and RCRC cases of the corresponding training set were assigned into a low-risk group and a high-risk group. The risk curves suggest that an increase in patient (LCRC and RCRC) risk scores contributed to the clustering of deaths **(** **Figures 2B**, **3B** **)**. K-M survival curves indicated that patients with LCRC (n = 77) and RCRC (n = 65) in the low-risk group had significantly better OS than patients of LCRC (n = 76; *P* = 0.048; **Figure 2C**) and RCRC (n = 65; *P* = 0.0089; **Figure 3C**) who had a high-risk score. Next, we observed the sensitivity and specificity of the risk scoring system for predicting the OS of LCRC and RCRC patients from 1 to 5 years by ROC curves. In the LCRC-training set, the AUCs of the risk scoring system consisting of *NOS2* and *IFNG* in predicting the OS of LCRC patients at 1 to 5 years were 0.662, 0.550, 0.592, 0.642, and 0.642, respectively **(** **Figure 2D** **)**. In the RCRC-training set, the risk score based on *NOS2* and *ALOXE3* had an AUC of 0.641 for 1-year OS, 0.711 for 2-year OS, 0.734 for 3-year OS, 0.733 for 4-year OS, and 0.725 for 5-year OS in RCRC patients **(** **Figure 3D** **)**. *NOS2* is expressed higher in the low-risk group, while *ALOXE3* expressed higher in the high-risk group. Consistently, in both the LCRC- and RCRC-test sets, a higher risk score (n _LCRC high-risk group_ = 33; n _RCRC high-risk group_ = 28) implied more deaths and a poorer clinical outcome (poorer OS) compared with low-risk LCRC (n = 33) and RCRC (n = 28) patients. The prognostic signature of LCRC based on 2 DE-FRGs (*NOS2* and *IFNG*) had AUCs of 0.662, 0.550, 0.592, 0.642, and 0.642 in the LCRC-test set for predicting patients’ OS at 1, 2, 3, 4, and 5 years, respectively. In the RCRC-test set, the efficiency of *NOS2* and *ALOXE3*-based prognostic features of RCRC in predicting patients’ OS from 1 to 5 years was 0.641, 0.711, 0.734, 0.733, and 0.725, respectively. *NOS2* is expressed higher in the left- and right-colorectal cancer low-risk group. On the contrary, IFNG is higher expressed in the left-colorectal cancer high-risk group, while Aloxe3 is more highly expressed in the right-colorectal cancer high-risk group.

### 3.3 Validation of Prognosis Signatures in the Entire TCGA-LCRC/RCRC Datasets

**Figure 4:**
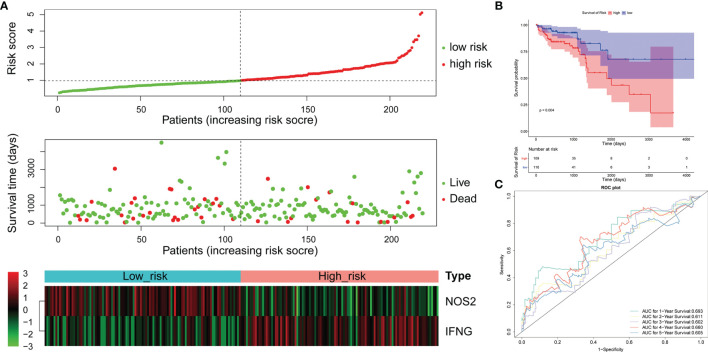
Validation of the 2-gene signature in the whole TCGA-LCRC cohort. **(A)** Distribution of the risk score, the associated survival data, and the mRNA expression heat map in the whole TCGA-LCRC cohort. **(B)** Kaplan-Meier plot for OS based on risk score of the two gene-based signature of patients with LCRC in the whole TCGA-LCRC cohort. **(C)** AUC of time-dependent ROC curves in the whole TCGA-LCRC cohort.

**Figure 5:**
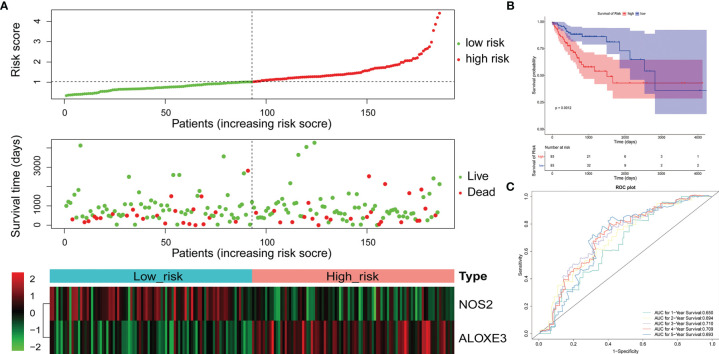
Validation of the 2-gene signature in the whole TCGA-RCRC cohort. **(A)** Distribution of the risk score, the associated survival data, and the mRNA expression heat map in the whole TCGA-RCRC cohort. **(B)** Kaplan-Meier plot for OS based on risk score of the two gene-based signature of patients with RCRC in the whole TCGA-RCRC cohort. **(C)** AUC of time-dependent ROC curves in the whole TCGA-RCRC cohort.

To confirm our findings, we validated our two signatures in the entire LCRC and RCRC cohorts. By using the same risk formula, we classified LCRC patients into high-risk (n = 109) or low-risk group (n = 110) using the median score of the entire LCRC cohort as the cutoff point. While there were 93 cases in both the high- and low-risk groups of RCRC patients. The risk curves were plotted separately for the entire LCRC and RCRC groups to illustrate the risk scores and survival status of each subject, meanwhile, the expression patterns of prognostic genes in the whole LCRC group and the whole RCRC group were demonstrated in the heatmaps **(** **Figures 4A**, **5A** **)**. The classification of the entire LCRC and RCRC cohorts based on risk scores by Kaplan-Meier analysis yielded similar results of poor prognosis for LCRC and RCRC patients in the high-risk group compared with the low-risk group (log-rank test *P* < 0.05; **Figures 4B**, **5B**). The AUCs of the ROC curves for the 2-gene signature based on *NOS2* and *IFNG* were 0.693 (1-year OS), 0.611 (2-year OS), 0.602 (3-year OS), 0.660 (4-year OS), and 0.605 (5-year OS), respectively, in the entire LCRC dataset **(** **Figure 4C** **)**. In the entire RCRC dataset, the prognostic signature consisting of *NOS2* and *ALOXE3* had AUCs of 0.650, 0.694, 0.710, 0.709, and 0.693 in predicting 1 to 5-year OS in RCRC patients, respectively **(** **Figure 5C** **)**. The above evidence demonstrated that the LCRC prognostic signature consisting of *NOS2* and *IFNG* and the 2-gene signature of RCRC based on *NOS2* and *ALOXE3* were able to predict clinical outcomes in CRC patients with tolerable confidence.

### 3.4 The Gene Signatures Were an Independent Predictor of the LCRC and RCRC Prognosis

**Figure 6:**
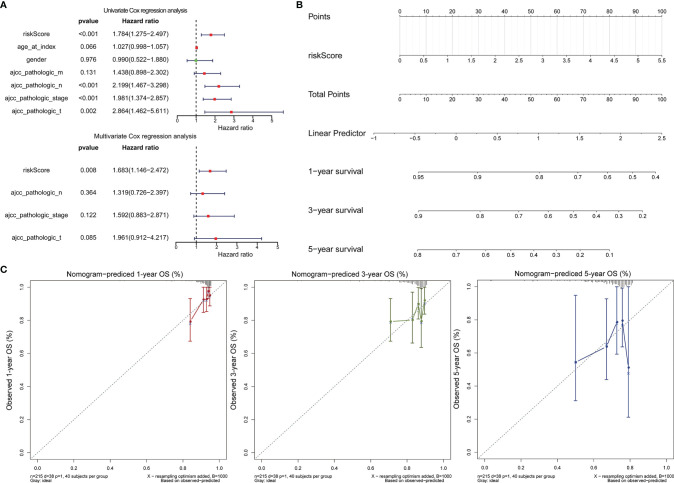
Assessment of the prognostic risk model of the 2 DE-FRGs in LCRC. **(A)** The univariate (top) and multivariate (bottom) Cox regression analysis of risk score and clinical features regarding prognostic value. Clinical features: age, gender, ajcc pathologic t (tumor size), ajcc pathologic n (lymph node metastasis), ajcc pathologic m (distant metastasis), and ajcc pathologic stage. **(B)** Nomogram predicting OS for LCRC and RCRC patients. For each patient, one line is drawn upward to determine the points received from the three predictors in the nomogram. The sum of the point is located on the ‘Total Points’ axis. Then a line is drawn downward to determine the possibility of 1-, 3-, and 5-year overall survival of LCRC. **(C)** The calibration plot for internal validation of the nomogram. The Y-axis represents actual survival, and the X-axis represents nomogram-predicted survival.

**Figure 7:**
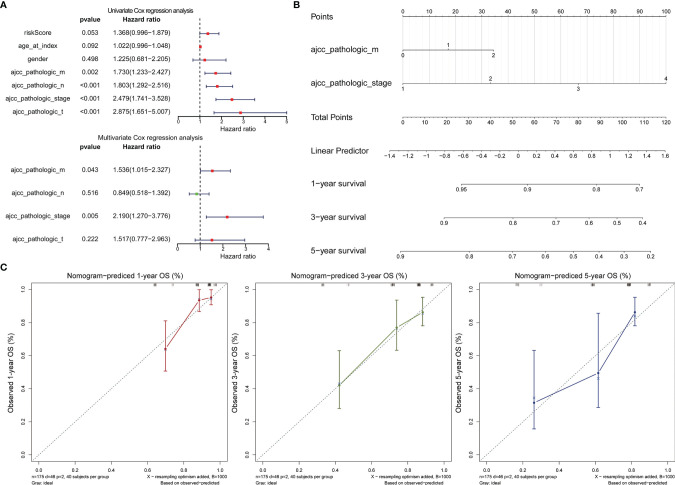
Assessment of the prognostic risk model of the 2 DE-FRGs in RCRC. **(A)** The univariate (top) and multivariate (bottom) Cox regression analysis of risk score and clinical features regarding prognostic value. Clinical features: age, gender, ajcc pathologic t (tumor size), ajcc pathologic n (lymph node metastasis), ajcc pathologic m (distant metastasis), and ajcc pathologic stage. **(B)** Nomogram predicting OS for RCRC patients. For each patient, two lines are drawn upward to determine the points received from the predictor in the nomogram. The sum of these points is located on the ‘Total Points’ axis. Then a line is drawn downward to determine the possibility of 1-, 3-, and 5-year OS of RCRC. **(C)** The calibration plot for internal validation of the Nomogram. The Y-axis represents actual survival, and the X-axis represents nomogram-predicted survival.

Cox regression analysis was performed to observe whether the prognostic models of LCRC and RCRC could influence the OS of patients in the presence of multiple clinicopathological characteristic factors (including age, gender, ajcc pathologic t, ajcc pathologic n, ajcc pathologic m, and ajcc pathologic stage). Univariate Cox regression analysis showed that for LCRC patients, risk scores, ajcc pathologic n, ajcc pathologic t, and ajcc pathologic stage were significantly associated with patient OS (*P* < 0.05; **Figure 6A**); besides, ajcc pathologic t, ajcc pathologic n, ajcc pathologic m, and ajcc pathologic stage were identified as factors associated with OS in patients with RCRC (*P* < 0.05; **Figure 7A**). Multivariate Cox analysis pointed out that risk score (*P* = 0.008) was an independent prognostic factor for LCRC patients **(** **Figure 6A** **)**, whereas independent prognostic factors for RCRC patients were ajcc pathologic m (*P* = 0.043) and ajcc pathologic stage (*P* = 0.005) **(** **Figure 7A** **)**. Subsequently, we constructed a Nomogram predicting 1, 3, and 5-year OS in LCRC patients based on the risk scores of LCRC patients **(** **Figure 6B** **)**. The higher the total points in the Nomogram, the worse the prognosis of the patient. The calibration curve assessed the predictive validity of the nomogram model for 1, 3, and 5-year OS in LCRC patients. The results suggested that we may have overestimated the ability of the risk score-based Nomogram to assess the prognosis of LCRC patients **(** **Figure 6C** **)**. However, the Nomogram **(** **Figure 7B** **)** model constructed based on independent prognostic factors for RCRC (ajcc pathologic m and ajcc pathologic stage) had the similar performance to the ideal model (slope = 1) **(** **Figure 7C** **)**. The DCA suggested that the combined model would exhibit the best net benefit in predicting patients’ 5-year OS **(** **Supplementary Figure 2** **)**. Collectively, these results suggested that the combined model was probably the best nomogram for predicting long-term survival (5 years), which might assist in the clinical management of CRC. However, we also revealed that the level of risk score was not correlated with all of the above clinicopathological characteristics in the entire LCRC either RCRC set **(** **Supplementary Figure 3** **)**, implying that the impact of our prognostic signatures on patient OS may not be confounded by patient pathologic features.

### 3.5 DEGs’ GO Analysis and Pathway Enrichment Analysis

**Figure 8:**
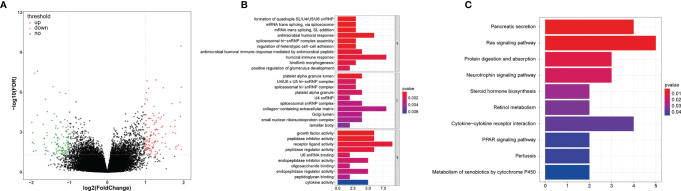
The GO and KEGG function enrichment analyses of the DEGs between high- and low-risk groups in LCRC patients. **(A)** A volcano plot of DEGs in the high- and low-risk groups. Red indicates up-regulated genes, green indicates down-regulated genes (high-risk group versus low-risk group), and black indicates no significant difference. The Top 10 biological processes, cellular components, molecular functions **(B)** and KEGG pathways **(C)** were illustrated. The color of the bar demonstrates P-value. Therefore, blue bars have a more significant P-value than red ones.

**Figure 9:**
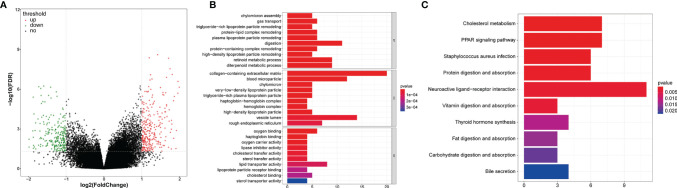
GO and KEGG enrichment analyses of the DEGs between high- and low-risk groups in RCRC patients. **(A)** A volcano plot of DEGs in the high- and low-risk groups. Red indicates up-regulated genes, green indicates down-regulated genes (high-risk group versus low-risk group), and black indicates no significant difference. The Top 10 biological processes, cellular components, molecular functions **(B)** and KEGG pathways **(C)** were illustrated. The color of the bar demonstrates P-value. Therefore, blue bars have a more significant P-value than red ones.

We first screened the DEGs (high-risk vs. low-risk) in each data series of the LCRC and RCRC independently, which identified 263 and 560 DEGs, respectively **(** **Figures 8A**, **9A** **)**.

In order to investigate the biological functions of the DEGs, the present study performed a functional pathway enrichment analysis of the DEGs through GO analysis and KEGG pathway enrichment in cluster Profiler. A *P* < 0.05 was considered to indicate a statistically significant difference. (**Figures 8B**, **9B**)presented the top 10 enriched terms of the three categories of GO analysis. We found that the DEGs associated with the high-risk group of LCRC were primarily involved in ‘antimicrobial humoral response’ (BP, *P* = 8.05E-05), ‘antimicrobial humoral immune response mediated by antimicrobial peptide (BP, *P* = 0.0009), ‘humoral immune response’ (BP, *P* = 0.001), ‘innate immune response-activating signal transduction’ (BP, *P* = 0.008), ‘activation of innate immune response’ (BP, *P* = 0.011), ‘chemokine activity’ (MF, *P* = 0.028), ‘chemokine receptor binding’ (MF, *P* = 0.048), and other GO processes related to immunity **(** **Supplementary Table 4** **)**. Interestingly, we found that DEGs associated with the RCRC high-risk group were mainly involved in lipid-related biological processes, such as ‘triglyceride-rich lipoprotein particle remodeling’ (*P* = 3.90E-07), ‘plasma lipoprotein particle remodeling’ (*P* = 7.39E-07), ‘phospholipid efflux’ (*P* = 8.47E-06), and ‘antibiotic catabolic process’ (*P* = 0.023). Consistently, DEGs related to the RCRC high-risk group were also significantly enriched in immune-related processes such as ‘humoral immune response’ (BP, *P* = 8.42E-05), ‘antimicrobial humoral response’ (BP, *P* = 0.0006), ‘regulation of cytokine secretion involved in immune response’ (BP, *P* = 0.015), ‘positive regulation of toll-like receptor signaling pathway’ (BP, *P* = 0.030), and ‘positive regulation of neutrophil chemotaxis’ (BP, *P* = 0.032) **(** **Supplementary Table 5** **)**.

The significantly enriched KEGG pathways of DEGs associated with the high-risk group of LCRC were the ‘Pancreatic secretion’, ‘Ras signaling pathway’, ‘Protein digestion and absorption’, ‘Neurotrophin signaling pathway’ and ‘Steroid hormone biosynthesis’ **(** **Figure 8C** **)**. The high-risk group of DEGs in the RCRC had primarily enriched in lipid-related terms (‘Cholesterol metabolism’, ‘Fat digestion and absorption’, ‘Steroid hormone biosynthesis’, and ‘Adipocytokine signaling pathway’) **(** **Figure 9C** **)**. However, the DEGs between the high- and low-risk groups in the left- or right-CRC dataset were all involved in the ‘PPAR signaling pathway’, which was related to tumor occurrence and development (16, 17) **(** **Supplementary Tables 6**, **7** **)**.

### 3.6 Analysis of the Immune Landscape Between the High- and Low-Risk Groups of CRC Patients on the Left- and Right-Sided

**Figure 10:**
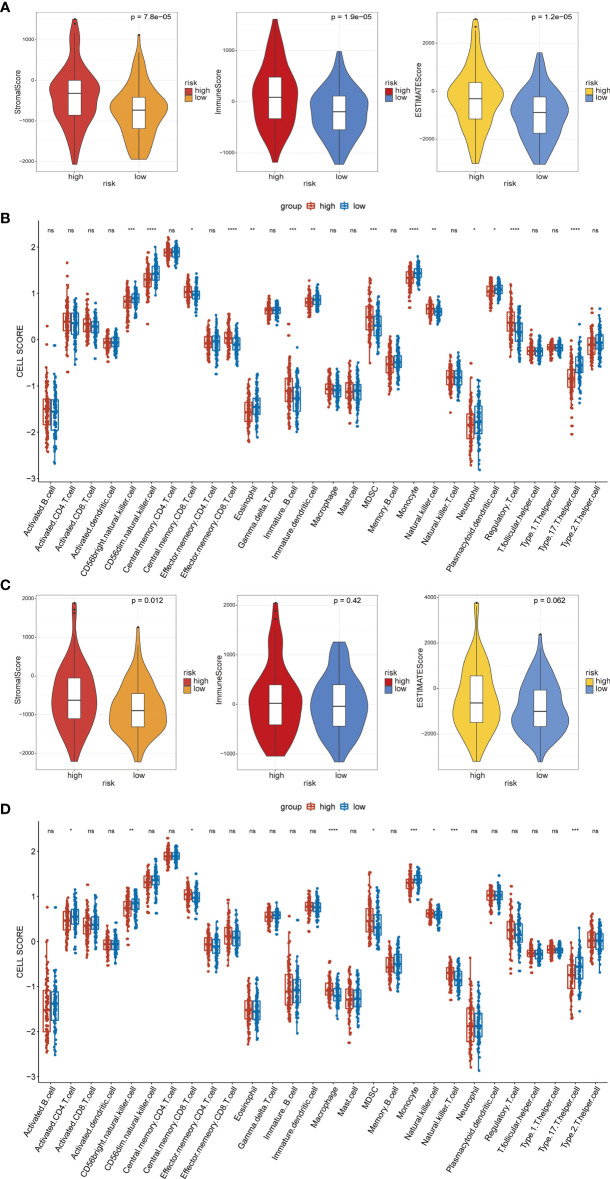
Immune analysis of the high- and low-risk groups. **(A)** The distribution of stromal scores, immune scores, and ESTIMATE scores in high- and low-risk groups of LCRC, all the P < 0.05. **(B)** Boxplot showing the differential abundance of 28 infiltrative immune cells calculated by ssGSEA between high-and low-risk groups in the LCRC. **(C)** The violin plot shows a correlation between the high-/low-risk group of RCRC and the stromal/immune/ESTIMATE scores level. **(D)** Boxplot of the abundance of immune cells between risk groups in TCGA-RCRC samples. *p < 0.05, **p < 0.01, ***p < 0.001, ****p < 0.0001. ns, not statistically significant.

The stromal and immune scores were consistently distributed between the high- and low-risk groups in the LCRC and RCRC **(** **Figures 10A, C** **)**. In both the LCRC and RCRC datasets, patients in the low-risk group had significantly lower stromal scores than the high-risk group (both *P* < 0.05). Besides, LCRC’s high-risk group yielded higher immune and ESTIMATE scores than those low-risk cases (all *P* < 0.0001). No significant differences between high- and low-risk RCRC were found for the immune and ESTIMATE scores (*P* = 0.42, 0.062, respectively).

Next, in LCRC and RCRC patients, ssGSEA revealed the association between FRG-based risk signatures and immune infiltrating cells. Overall, half of the 28 immune cells were significantly different between high- and low-risk groups in the LCRC patient cohort **(** **Figure 10B** **)**. Notably, the expression of central memory CD8 T cell, effector memory CD8 T cell, immature B cell, Myeloid-derived suppressor cell (MDSC), natural killer (NK) cell, and regulatory T cell were significantly increased in high-risk LCRC patients relative to low-risk LCRC patients. In the RCRC patient cohort, additionally, FRGs-related risk scores were significantly positively correlated with many immune cell types, including central memory CD8 T cell, macrophage, MDSC, NK cell, and NK T cell **(** **Figure 10D** **)**.

### 3.7 The Landscape of FRGs’ Mutation Profiles in CRC

**Figure 11:**
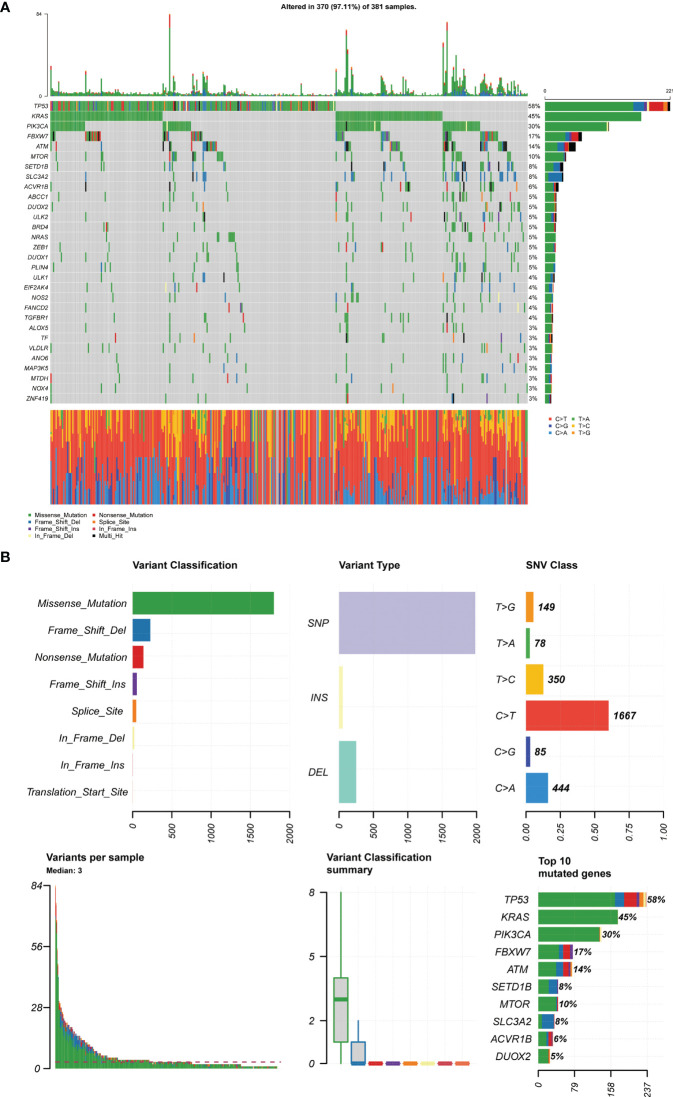
TCGA-CRC mutation cohort. **(A)** The waterfall map depicts the frequently mutated FRGs (top 30) in CRC. The right panel shows mutation frequency, and genes are ordered by their mutation frequencies. The bottom panel presents different mutation types. **(B)** Overview of TGCA-CRC cohort FRGs mutations. Classification and frequency of mutation types (top left). Frequency of variant types (top middle). Frequency of SNV classes (top right). Tumor mutation burden in specific samples (bottom left and middle). The top 10 mutated genes in CRC (bottom right).

We downloaded somatic mutation profiles of 381 CRC patients from TCGA. Mutations and mutation frequencies in the top 30 FRGs were exhibited in a waterfall plot, where various colors with annotations at the bottom represented the different mutation types **(** **Figure 11A** **)**. In summary, these mutations were further classified according to different classified categories, in which missense mutation accounts for the most fraction, single nucleotide polymorphism (SNP) occurred more frequently than deletion (DEL) or insertion (INS), and C > T was the most common of single nucleotide variants (SNV) in CRC. Besides, we counted the number of altered bases in each sample and showed the mutation type with different colors in the box plot for CRC. Last, we exhibited the top 10 mutated FRGs in CRC with ranked percentages, including *TP53* (58%), *KRAS* (45%), *PIK3CA* (30%), *FBXW7* (17%), *ATM* (14%), *MYOR* (10%), *SETD1B* (8%), *SKC3A2* (8%), *ACVR1B* (6%), and *DUOX2* (5%) **(** **Figure 11B** **)**. By comparison, the mutation frequency of NOS2 among the prognostic genes was the highest at 4%, but in fact, this was still relatively conservative. These data thus suggested that FRGs-related risk signatures were unrelated to TMB in CRC patients.

#### 3.7.1 Differential Expression of Target Genes in Tumor Tissues and Normal Tissues

**Figure 12:**
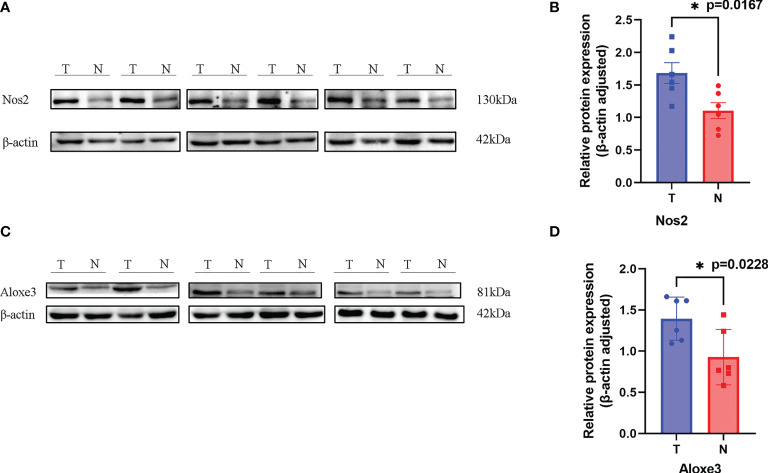
Results of Western blotting. **(A, C)**. NOS2 and ALOXE3 were significantly more highly expressed in tumor tissues than in normal tissues. (T, Tumor; N, Normal) **(B)**. P = 0.0167 < 0.05, the difference was statistically significant. **(D)**. P = 0.0228, the difference was statistically significant. *p < 0.05.

**Figure 13:**
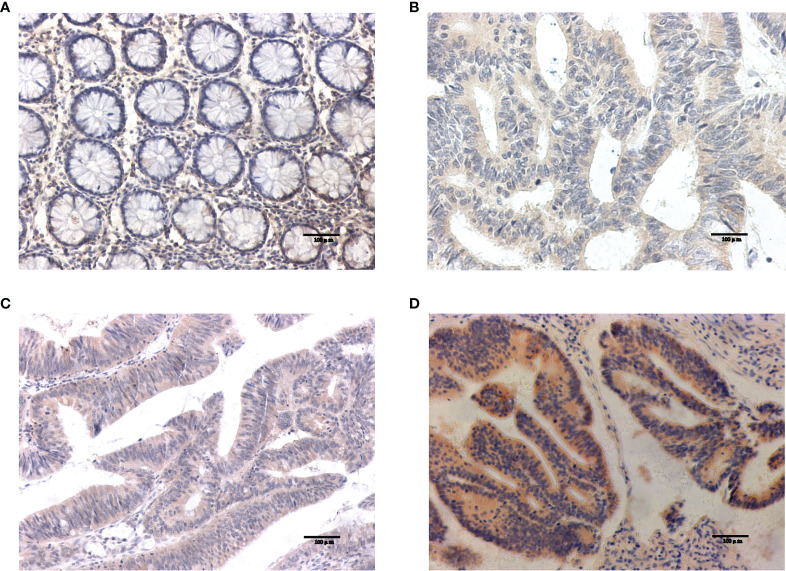
Results of NOS2 immunohistochemistry. **(A)** Negative expression of NOS2 in normal tissues. **(B–D)** The weak positive, moderate strong positive and strong positive expression of NOS2 in tumor tissues, in turn.

**Figure 14:**
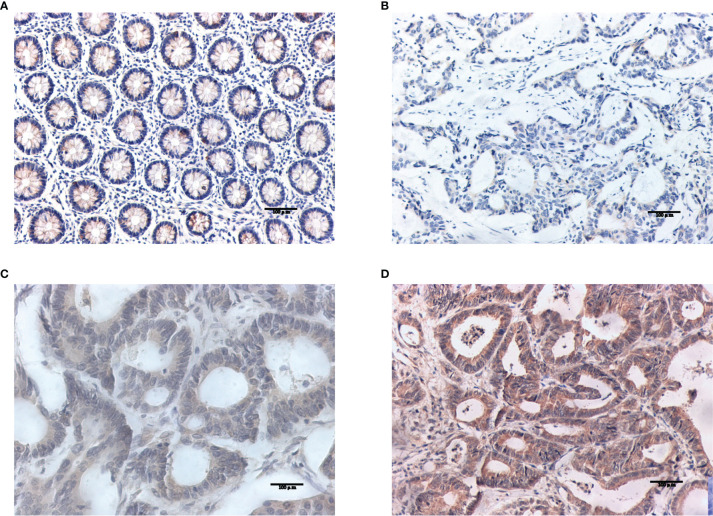
Results of ALOXE3 immunohistochemistry. **(A)** Negative expression of ALOXE3 in normal tissues. **(B–D)** The weak positive, moderate strong positive and strong positive expression of ALOXE3 in tumor tissues, in turn.

Western blotting results showed that NOS2 and ALOXE3 expression was significantly increased in tumor tissues compared with that in normal tissues **(** **Figure 12**, P < 0.01). The positive staining of NOS2 and ALOXE3 in cells was mainly localized in the cell membrane and cytoplasm, and NOS2 was positively expressed in 90 cases, with a positive expression rate of 63.4% (90/142), mainly showing moderate and strong positive expression. The difference of NOS2 positive expression rate between tumor tissues and normal tissues was statistically significant(χ²=17.261, P=0.000).ALOXE3 was positively expressed in 103 cases, with a positive expression rate of 72.5% (103/142), mainly showing moderate and strong positive expression. The difference of ALOXE3 positive expression rate between tumor tissues and normal tissues was statistically significant(χ²=21.044, P=0.000) (**Figures 13**, **14**).

#### 3.7.2 Correlation of NOS2 and ALOXE3 Expression With Clinicopathological Characteristics of Patients in LCC and RCC

The differential expression of NOS2 and ALOXE3 in tumor tissues and normal tissues suggests that NOS2 and ALOXE3 may be involved in the regulation of tumorigenesis and progression. Therefore, we further analyzed the correlation between their expression in LCC and RCC tumor tissues and clinicopathological characteristics of tumors. Immunohistochemical results showed that the positive rate of ALOXE3 in RCC tumor tissues (78.1%) was higher than that in LCC tumor tissues (53.6%) (P < 0.05). The positive rate of ALOXE3 expression in tumor samples with positive lymph node metastasis (78.3%) was significantly higher than that in those without lymph node metastasis (60.3%) (P < 0.05). However, statistical analysis did not reveal that the expression of NOS2 was correlated with gender, age, tumor location, tumor differentiation, or TNM stage (P > 0.05, **Table 2** **)**. The above results indicate that ALOXE3 is closely related to the invasion and metastasis of RCC while NOS2 may affect both LCC and RCC.

**Table 2:** Correlation between NOS2, ALOXE3 expression levels and clinicopathological characteristics of patients with colon cancer.

Item		Aloxe3				Nos2			
	n	High expression(%)	Low expression(%)	χ²	P	High expression(%)	Low expression(%)	χ²	P
**Age (years)**				0.067	0.796			1.151	0.283
≥60	97	71 (73.2)	26 (26.8)			63 (64.9)	34 (35.1)		
<60	45	32 (71.1)	13 (28.9)			25 (55.6)	20 (44.4)		
**Gender**				0.082	0.775			0.054	0.816
Male	81	58 (71.6)	23 (28.4)			52 (64.2)	29 (35.8)		
Female	61	45 (73.8)	16 (26.2)			38 (62.3)	23 (37.7)		
**Tumor location**				9.484	0.002			0.365	0.546
Right	73	57 (78.1)	16 (21.9)			48 (65.8)	25 (34.2)		
Left	69	37 (53.6)	32 (46.4)			42 (60.9)	27 (39.1)		
**Differentiation status**				0.332	0.564			0.067	0.795
Low differentiation	49	20 (80.0)	12 (24.5)			30 (61.2)	19 (38.8)		
Moderate and high differentiation	93	83 (71.0)	27 (29.0)			59 (63.4)	34 (36.6)		
**Depth of invasion**				0.849	0.357			0.063	0.802
T1/T2	25	20 (80.0)	5 (20.0)			10 (40.0)	15 (60.0)		
T3/T4	117	83 (71.0)	34 (29.0)			50 (42.7)	67 (57.3)		
**Lymph node metastasis**				5.367	0.021			0.002	0.966
N0	73	44 (60.3)	29 (39.7)			32 (43.8)	41 (56.2)		
N+	69	54 (78.3)	15 (21.7)			30 (43.5)	39 (56.5)		
**Distant metastasis**				0.425	0.514			3.611	0.057
M0	117	87 (74.4)	30 (25.6)			70 (59.8)	47 (40.2)		
M1	25	17 (68.0)	8 (32.0)			20 (80.0)	5 (20.0)		

### 3.8 Correlation of NOS2 and ALOXE3 Expression With Clinical Prognosis of Patients With Colon Cancer

**Figure 15:**
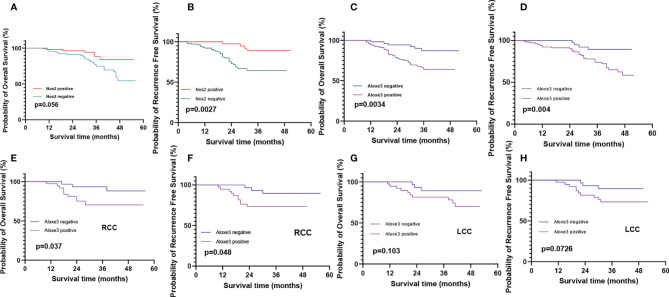
Recurrence-free survival and overall survival curves of patients with positive and negative expression of NOS2 and ALOXE3 in colon cancer. **(A, C)** Overall survival curves of NOS2, ALOXE3 for all patients. **(B, D)** Recurrence-free survival curves of NOS2, ALOXE3 for all patients. **(E, G)** Overall survival curves of RCC and LCC. **(F, H)** Recurrence-free survival curves of RCC and LCC.

Invasion and metastasis is an important risk factor in patients with colon cancer death. Since the expression of ALOXE3 in colon cancer is closely related to tumor invasion and metastasis, we further analyzed the correlation between NOS2, ALOXE3 and clinical prognosis of patients. Kaplan-Meier survival analysis showed that the overall survival of colon cancer patients with low ALOXE3 expression was significantly higher than that of those with high ALOXE3 expression, (P < 0.05), meanwhile the 5-year recurrence-free survival of colon cancer patients with high NOS2 expression was significantly higher than that of those with low NOS2 expression, and the difference was significant (P < 0.05). Further stratified analysis according to tumor location showed that ALOXE3 was significantly correlated with 5-year recurrence-free survival and overall survival in RCC, which was significantly lower in positive patients than in negative patients (P < 0.05), while in LCC, there was no significant difference. **(** **Figure 15** **).**

## 4 Discussion

Programmed cell death is a hot topic in biological research and medicine. Targeting the cell death process is a common method for cancer therapy. As a novel programmed cell death process, ferroptosis, characterized by iron-dependent lipid peroxidation (IDLPO) accumulation, shows great potential in cancer therapy. However, until now, little is known about the roles and mechanisms of ferroptosis-related genes in left-sided and right-sided colon cancers. Therefore, it is necessary to identify the key ferroptosis-related genes that are differentially expressed in left-sided and right-sided colorectal cancer (CRC) and explore their impact on patient prognosis. In this study, for the first time, we constructed robust polygenic prognostic models for left-sided and right-sided CRC, respectively.

Liu et al. established a prognostic model consisting of 10 FRGs in CRC and confirmed the predictive value of Overall Survival (OS) risk score in CRC patients using log-rank test and Kaplan-Meier analysis (18), but they did not distinguish the expression difference of FRGs in left-sided and right-sided colon cancers. In this study, we identified 14 ferroptosis-related genes that were differentially expressed in left-sided and right-sided colon cancers, and constructed robust prognostic models for left-sided and right-sided CRC by univariate and multivariate COX analysis, respectively. We found that NOS2 could simultaneously affect the prognosis of patients with left-sided and right-sided colon cancer. IFNG is highly expressed in left-sided colon cancer, while ALOXE3 is highly expressed in right-sided colon cancer. The relationship between the expression of NOS2 and ALOXE3 and the PFS and OS of patients was further verified by experiments.

NOS2 is an inducible nitric oxide synthase, and as a pro-inflammatory mediator, NOS2 may promote cancer initiation and progression (19, 20). It was initially shown to be a major player in the antitumor component of the immune response. However, recent data suggest that high expression of NOS2 in cancer cells often predicts poor outcomes, such as high expression in breast cancer (21), lung cancer (22),gliomas (23) and colon cancer (24).Shao et al. (25)also found that NOS2 was significantly up-regulated in colon cancer in their study on ferroptosis-related genes predicting the prognosis of colon cancer patients. Similarly, in this experiment, we also found that NOS2 was significantly overexpressed in colon cancer, but there was no significant difference in expression between left-sided and right-sided colon cancers. Studies in the early 2000s showed that NOS2 is present in 50%-60% of colon cancer patients. High expression of NOS2 is associated with decreased long-term survival and increased incidence of lymph node metastasis and lymphatic invasion (26). However, studies have shown that NOS2 is significantly downregulated in individuals with advanced CRC (27). NOS2 expression is associated with colon cancer progression, but its role in tumor development is not clear. We found that elevated levels of NOS2 expression, early in colon cancer progression, can significantly affect the 5-year recurrence-free productivity of patients, but there was no significant effect on patient OS. In the previous analysis, we found that NOS2 was significantly down-regulated in the high-risk group, and in the left-sided and right-sided colon cancer, patients with T3-4 and stage III-IV had relatively high risk score levels. In conclusion, NOS2 may have multiple roles in the induction and early progression of colon cancer as well as in the late stage.

Interferon gamma (IFNG) is a pro-inflammatory cytokine that regulates many immune-related genes. It has been found that in some cases, IFNG obviously plays a role in inducing tumor progression, and its induced PD-L1 expression could serve as a novel mechanism by which it impairs tumor immunity. Therefore, tumor cells acquire the ability to attack against immune cells to induce immune escape phenomenon (28). Genetic variation of IFNG leads to an increased risk of colon and rectal cancer and affects its diagnosis and survival (29). Similarly, this experiment found that IFNG was significantly overexpressed in left-sided colon cancer through bioinformatics analysis. Unfortunately, IFNG is an interferon, we need to examine the content in the patients’ blood to evaluate the relationship between its expression and prognosis, but we cannot collect relevant samples, so we have not verified its effect on the prognosis of patients through experiments.

ALOXE3 is an encoding arachidonic acid, whose metabolism plays an important role in tumor progression and metastasis (30, 31). ALOXE3, also known as ARCI3, E-LOX, elox3, and Elox-3, is a member of the lipoxygenase family. and mutations in ALOXE3 have been reported to be associated with the development of autosomal recessive congenital ichthyosis (ARCI) (32).But little is known about the function of ALOXE3 in cancer and its mechanism of action. Xia et al. (33)have found that talaroconvolutinA (TalaA), a novel ferroptosis inducer, increases lipid peroxidation by increasing ALOXE3 expression, which in turn enhances ferroptosis. This study suggests that it is of great significance to develop new anticancer drugs through ferroptosis induction. Ruan et al. (34) investigated the potential relationship of perlipoxygenase (LOX) family genes in the diagnostic and prognostic value of colon cancer. They used multivariate survival analysis and comprehensive prognosis to show that ALOXE3 and ALOX12 were associated with colon cancer OS, the low expression of both is better for the prognosis of COAD, and ALOXE3 combined with ALOX12 may serve as a potential prognostic biomarker for COAD. However, its differential expression in left-sided and right-sided colon cancer and its effect on prognosis were not distinguished. In this study, we first used bioinformatics analysis to find that ALOXE3 is a ferroptosis-related gene and is differentially expressed in left-sided and right-sided colon cancer. Western blotting confirmed that ALOXE3 was significantly highly expressed in right-sided colon cancer, and patients with its high expression in right-sided colon cancer had a worse prognosis, indicating that its high expression increased the risk of death.

Functional enrichment analysis showed that DEGs associated with the prognostic model of FRGs were significantly enriched in terms and pathways related to lipids, while FRGs were also associated with lipids. Interestingly, CRC was also associated with lipids. Steroids are essential components of membrane lipids and can act as signaling molecules. Very low-density lipoprotein (VLDL) was positively associated with the frequency of colonic adenomas. Importantly, triglycerides (TG) and LDL were associated with CRC prognosis, as the levels of TG and LDL were significantly elevated in patients with distant metastases. In addition, cholesterol in a high-fat diet, which is strongly associated with the development of colorectal tumors (35). In this study, through the enrichment analysis of DEGs, we found that in LCRC and RCRC, DEGs were involved in the “PPAR signaling pathway” in both high and low risk groups. The expression is significantly reduced in cancers such as gastric cancer (36), cervical cancer (37), and esophageal cancer (38).Numerous studies have shown that PPARγ has antitumor effects on lung cancer, breast cancer, prostate cancer and colon cancer (39), which undoubtedly provides a new direction for the treatment of colon cancer.

In this study, we revealed changes in the immune microenvironment using ssGSEA and ESTIMATE analysis. RCRC has been found to have a higher degree of immune infiltration than LCRC, and right-sided tumors have a unique immunophenotype characterized by more immune infiltration and higher levels of immune activation compared with left-sided tumors (40).We performed differential analysis using 28 immune-related gene sets, of which half of the genes were significantly different in high and low risk groups. We found that in RCRC, risk scores associated with FRGs were significantly positively associated with many immune cell types, including Central memory CD8 T cell, macrophages, MDSC, NK cell and NK T cell. In CRC, patients with mild and moderate NK cell infiltration have been reported to have significantly lower 5-year survival rates than patients with extensive NK cell infiltration (41).Higher NKT cell infiltration is an independent prognostic factor for good prognosis in patients with colon cancer (42).

In this study, we found that there were significant differences in NK cell in both left-sided and right-sided colon cancer at high and low risk, while NKT cell was only significantly different in right-sided colon cancer at high and low risk, and there was no significant difference in left-sided colon cancer. Macrophage infiltration is often a poor prognostic factor in different types of cancer, but the increased degree of macrophage infiltration in CRC is associated with good prognosis (43). We found that the degree of immune infiltration of macrophages was significantly different in the high and low risk groups of right-sided colon cancer, but not in the high and low risk groups of left-sided colon cancer. MDSC is an immunosuppressive cell, as a regulatory T cell, it promotes immune tolerance by inhibiting the function of CD8+ T cells. The prognostic value of MDSC is not known. However, it has been shown in related experiments that elimination of MDSC enhances the anti-tumor response in mouse tumor models (44). In this study, the risk of MDSC in left-sided and right-sided colon cancer was significantly different, and the role and prognosis of immune cell infiltration in CRC remains to be further explored.

Prognostic FRGs in LCRC and RCRC were not significantly associated with TMB. In our study, although mutations in prognostic genes were conserved, we found that TP53 was the most frequently mutated among all FRGs in CRC. CRC development is a multifactorial, multistage process involving the activation of oncogenes and the inactivation of tumor suppressor genes. Numerous studies have confirmed that p53 is a key tumor suppressor gene and is one of the most important elements of human anti-cancer defense (45). It is well known that CRC progression is accompanied by mutations in APC, K-Ras and p53 genes (46). Unfortunately, we did not find a direct association between prognostic gene and mutation or P53 in functional enrichment analysis. However, NOS2 expression has been reported to correlate with p53 status in both LCRC and RCRC prognostic models. Studies have shown that p53 and vascular endothelial growth factor can regulate the expression of NOS2 to promote tumor growth (47). These evidences suggest that there may be a mechanism of action to be explored between prognostic gene and p53 mutation.

## 5 Conclusions

In this study, firstly, we used bioinformatics methods to explore the differentially expressed ferroptosis-related genes and potential prognostic value in left-sided and right-sided colon cancer, but there are still some shortcomings. On the one hand, the relevant data comes from public websites, and the clinical parameters are not perfect. On the other hand, we have not verified the cell experiments and mouse tumorigenesis experiments, and have not further explored the mechanism of NOS2, IFNG and ALOXE3 in the occurrence and development of colon cancer. This study systematically evaluated the differential expression of the screened ferroptosis-related genes in left-sided and right-sided colon cancer and the potential prognostic value in colon cancer.

## Data Availability Statement

The authors acknowledge that the data presented in this study must be deposited and made publicly available in an acceptable repository, prior to publication.

## Ethics Statement

The studies involving human participants were reviewed and approved by the Ethics Committee of the First Affiliated Hospital of Jinzhou Medical University. The patients/participants provided their written informed consent to participate in this study. Written informed consent was obtained from the individual(s) for the publication of any potentially identifiable images or data included in this article.

## Author Contributions

YC and HL conceived and designed the Study. YC collected data, conduct experiment, analyzed statistical. YC was a major contributor in writing the manuscript. HL collected the funds. All authors read and approved the final manuscript.

## Funding

The research was funded by Science and Technology Project of Jinzhou Science and Technology Bureau(19B1D34).

## Conflict of Interest

The authors declare that the research was conducted in the absence of any commercial or financial relationships that could be construed as a potential conflict of interest.

## Publisher’s Note

All claims expressed in this article are solely those of the authors and do not necessarily represent those of their affiliated organizations, or those of the publisher, the editors and the reviewers. Any product that may be evaluated in this article, or claim that may be made by its manufacturer, is not guaranteed or endorsed by the publisher.

## Supplementary Material

Supplementary Figure 1 Prognostic analysis of DE-FRGs signatures in LCRC- and RCRC-test sets. Risk score analysis (the dotted line represented the median risk score and divided the patients into low- and high-risk groups), heatmap of the expression profiles of the two prognostic genes in low- and high-risk group, Kaplan–Meier curve survival analysis, and time–ROC curve analysis of the DE-FRGs signatures in LCRC **(A)**- and RCRC **(B)**-test sets.

Supplementary Figure 2 The DCA curves of the nomogram. **(A, B)** The DCA curves of the nomograms compared for 3- and 5-year OS in LCRC and RCRC, respectively. The none plot represented the assumption that no patients have 3- or 5-year survival; while all plot represented the assumption that all patients have 3- or 5-year survival at a specific threshold probability. The x-axis represented the threshold probabilities, and the y-axis measured the net benefit.

Supplementary Figure 3 The risk score of LCRC and RCRC is not associated with the clinicopathological characteristics. **(A, C)** A heat map of the correlation between clinical characteristics of CRC. The redder color reflects higher expression and the bluer color reflects lower expression. **(B)** The distribution of risk score among different clinical characteristics of LCRC. **(D)** The distribution of risk score among different clinical characteristics of RCRC. Clinical characteristics: age, gender, ajcc pathologic t (tumor size), ajcc pathologic n (lymph node metastasis), ajcc pathologic m (distant metastasis), and ajcc pathologic stage.

Supplementary Table 1 DEGs expression results in left- and right- colorectal cancer. P < 0.05 and |log2 fold change (FC)| > 0.5.

Supplementary Table 2 The choice criterion for the DEGs (high-risk group in LCRC/RCRC vs. low-risk group in LCRC/RCRC). P < 0.05 and |log2 fold change (FC)| > 1.

Supplementary Table 3 The choice criterion for the DEGs (high-risk group in LCRC/RCRC vs. low-risk group in LCRC/RCRC). P < 0.05 and |log2 fold change (FC)| > 1.

Supplementary Table 4 Results of GO enrichment analysis related to immunity. GO – Gene Ontology. *P*-value <0.05.

Supplementary Table 5 Results of GO enrichment analysis related to lipid-related biological processes. *P*-value <0.05.

Supplementary Table 6 KEGG pathway analysis in left- and right- colorectal cancer. KEGG – Kyoto Encyclopedia of Genes and Genomes. *P*-value <0.05.

Supplementary Table 7 KEGG pathway analysis in left- and right- colorectal cancer. KEGG – Kyoto Encyclopedia of Genes and Genomes. *P*-value <0.05.

## Abbreviations

LCRC, Left-sided colorectal cancer; RCRC, Right-sided colorectal cancer; FRGs, Ferroptosis-related genes; CRC, Colorectal cancer; TCGA, The Cancer Genome Atlas; DEGs, Differentially expressed genes; DE-FRGs, Differentially expressed ferroptosis-related genes; GO, Gene ontology; KEGG, Kyoto encyclopedia of genes and genomes; ssGSEA, Single sample Gene Set Enrichment Analysis; TME, Tumor microenvironment; OS, Overall survival; ccRCRC, Clear cell renal cell carcinoma; BLCA, Bladder urothelial carcinoma; CHOL, Cholangiocarcinoma; SKCM, Skin cutaneous melanoma; PPAR, Peroxisome proliferator-activated receptor; AUC, Area under the curve; NK, Natural killer; PD-L1, Programmed death ligand 1; VLDL, Very low-density lipoprotein; TG, Triglycerides; MDSC, Myeloid-derived suppressor cell; NKT, Human invariant natural killer T.
