# Supplementary figures and images for: Prognostic and Predictive Models for Left- and Right- Colorectal Cancer Patients: A Bioinformatics Analysis Based on Ferroptosis-Related Genes
Source: Front Oncol. 2022 Feb 21;12:833834. doi: 10.3389/fonc.2022.833834 (PMC8899601; doi:10.3389/fonc.2022.833834)

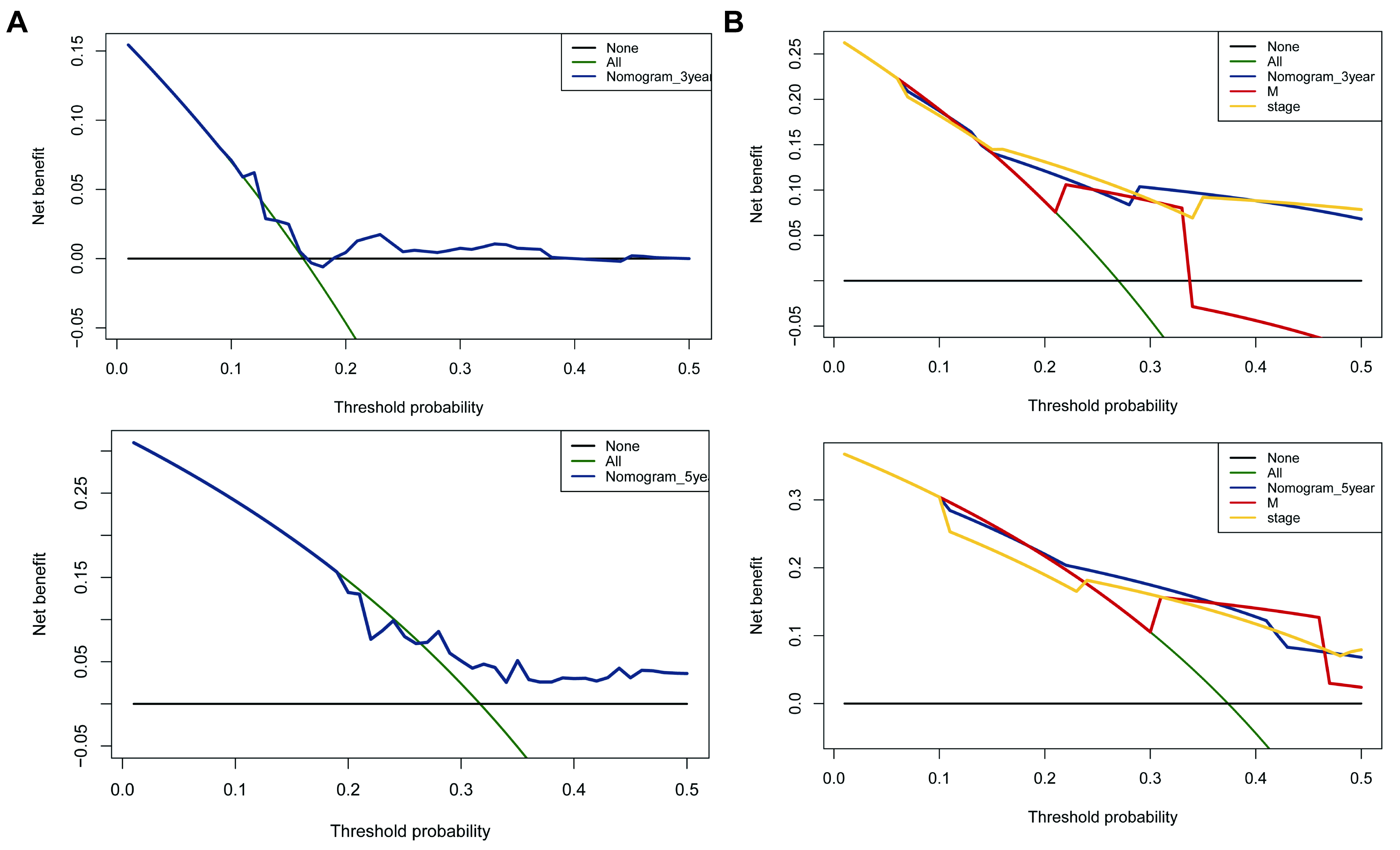

Supplement: Supplementary Figure 2 — The DCA curves of the nomogram. (A, B) The DCA curves of the nomograms compared for 3- and 5-year OS in LCRC and RCRC, respectively. The none plot represented the assumption that no patients have 3- or 5-year survival; while all plot represented the assumption that all patients have 3- or 5-year survival at a specific threshold probability. The x-axis represented the threshold probabilities, and the y-axis measured the net benefit. [file Image_2.tif]
