# Supplementary material for: Prognostic and Predictive Models for Left- and Right- Colorectal Cancer Patients: A Bioinformatics Analysis Based on Ferroptosis-Related Genes
Source: Front Oncol. 2022 Feb 21;12:833834. doi: 10.3389/fonc.2022.833834 (PMC8899601; doi:10.3389/fonc.2022.833834)
Supplement: Supplementary Table 1 — DEGs expression results in left- and right- colorectal cancer. P < 0.05 and |log2 fold change (FC)| > 0.5. [file Table_1.docx]

|  | baseMean | log2FoldChange | lfcSE | stat | pvalue | padj | threshold |
| --- | --- | --- | --- | --- | --- | --- | --- |
| MTND1P23 | 177.1672817 | 2.876213101 | 0.232292881 | 12.38183919 | 3.28E-35 | 1.10E-30 | up |
| PTF1A | 8.961744991 | -4.341602696 | 0.358568616 | -12.1081503 | 9.56E-34 | 1.60E-29 | down |
| MUC6 | 327.0069119 | 2.911012742 | 0.262749737 | 11.07903199 | 1.59E-28 | 1.77E-24 | up |
| PRAC1 | 489.0287089 | -3.652929906 | 0.341145325 | -10.70784103 | 9.35E-27 | 6.66E-23 | down |
| GDF10 | 36.13648249 | -2.68120329 | 0.250533148 | -10.70199017 | 9.96E-27 | 6.66E-23 | down |
| M1AP | 12.92199586 | 1.913554325 | 0.179504313 | 10.66021362 | 1.56E-26 | 8.71E-23 | up |
| INSL5 | 13.73785346 | -3.439570743 | 0.349031784 | -9.854606074 | 6.55E-23 | 3.13E-19 | down |
| CLDN10 | 23.44275069 | -2.768658429 | 0.282623257 | -9.796286627 | 1.17E-22 | 4.88E-19 | down |
| ZNF880 | 116.7098051 | -1.192089042 | 0.124271492 | -9.592618719 | 8.59E-22 | 3.04E-18 | down |
| NXF3 | 102.9149724 | 2.463551977 | 0.256979121 | 9.586584166 | 9.10E-22 | 3.04E-18 | up |
| WNT7B | 64.66174137 | 1.825584069 | 0.192822395 | 9.467697302 | 2.86E-21 | 8.70E-18 | up |
| WIF1 | 161.0873392 | -3.030924756 | 0.320958732 | -9.44334723 | 3.61E-21 | 1.01E-17 | down |
| FITM2 | 1265.673118 | -0.740003068 | 0.07970594 | -9.284164624 | 1.63E-20 | 4.19E-17 | down |
| VNN1 | 423.4735382 | 1.725726847 | 0.188080844 | 9.175452503 | 4.50E-20 | 1.07E-16 | up |
| CRYBA2 | 17.91547476 | 1.837827929 | 0.203712293 | 9.021683978 | 1.85E-19 | 4.13E-16 | up |
| TFAP2A | 250.6969206 | 1.382503716 | 0.154948268 | 8.922356722 | 4.56E-19 | 9.54E-16 | up |
| HOXB13 | 744.1184138 | -1.204199068 | 0.135207822 | -8.90628255 | 5.28E-19 | 1.04E-15 | down |
| MYBPHL | 17.14231783 | -2.331789992 | 0.265195171 | -8.792731724 | 1.46E-18 | 2.58E-15 | down |
| ELAVL2 | 33.88661832 | -1.834363073 | 0.208634101 | -8.792249494 | 1.47E-18 | 2.58E-15 | down |
| FABP3 | 293.5502021 | -1.420427691 | 0.163618319 | -8.681348747 | 3.91E-18 | 6.54E-15 | down |
| ANKRD45 | 9.806950831 | 1.662251803 | 0.191764151 | 8.668209333 | 4.39E-18 | 6.99E-15 | up |
| SNORA71D | 3.282401406 | -2.385651099 | 0.276589 | -8.625256621 | 6.39E-18 | 9.72E-15 | down |
| TP53RK | 1355.909254 | -0.589516401 | 0.068459394 | -8.611183454 | 7.23E-18 | 1.05E-14 | down |
| HOXC6 | 72.16493118 | 1.845382773 | 0.21635249 | 8.529519462 | 1.47E-17 | 2.05E-14 | up |
| HOXC10 | 16.19494598 | 2.188492878 | 0.258222793 | 8.475211847 | 2.35E-17 | 3.09E-14 | up |
| ANPEP | 4786.638864 | 1.654731185 | 0.195700792 | 8.455413828 | 2.78E-17 | 3.32E-14 | up |
| AL355075.4 | 3.825964265 | -3.010895887 | 0.356952531 | -8.435003619 | 3.31E-17 | 3.69E-14 | down |
| NXPH4 | 301.5402864 | 1.87695047 | 0.223693683 | 8.390717364 | 4.83E-17 | 5.21E-14 | up |
| POFUT1 | 5857.702916 | -0.628071434 | 0.075964499 | -8.267959911 | 1.36E-16 | 1.42E-13 | down |
| ARL11 | 165.9508949 | -1.120584497 | 0.135891634 | -8.246162499 | 1.64E-16 | 1.66E-13 | down |
| VSTM2L | 136.5464603 | -1.577032244 | 0.191669957 | -8.227853082 | 1.91E-16 | 1.87E-13 | down |
| TRPV6 | 54.65803128 | 2.119571081 | 0.259186898 | 8.177770938 | 2.89E-16 | 2.76E-13 | up |
| TMEM252 | 87.19662617 | -1.798284607 | 0.220674484 | -8.149037319 | 3.67E-16 | 3.32E-13 | down |
| MTCO1P12 | 3792.70574 | 1.363035071 | 0.167915923 | 8.117366389 | 4.76E-16 | 4.09E-13 | up |
| OSER1 | 1994.607239 | -0.565985063 | 0.070029969 | -8.08204081 | 6.37E-16 | 5.32E-13 | down |
| H3F3AP6 | 16.35225945 | 1.257370138 | 0.155881096 | 8.066213078 | 7.25E-16 | 5.91E-13 | up |
| SLC26A9 | 31.31931163 | 1.997152108 | 0.248135446 | 8.048636899 | 8.37E-16 | 6.36E-13 | up |
| SNORA71A | 15.8678447 | -1.255837749 | 0.156565664 | -8.02115683 | 1.05E-15 | 7.78E-13 | down |
| KCTD1 | 174.2999573 | 0.823908476 | 0.102759415 | 8.017839301 | 1.08E-15 | 7.82E-13 | up |
| RBP3 | 6.22788462 | 2.183073117 | 0.272640589 | 8.007146416 | 1.17E-15 | 8.35E-13 | up |
| NEUROG3 | 30.56749893 | 2.11856763 | 0.26616777 | 7.959519765 | 1.73E-15 | 1.20E-12 | up |
| ONECUT3 | 142.8958064 | 2.527439362 | 0.317773075 | 7.953598211 | 1.81E-15 | 1.24E-12 | up |
| PDRG1 | 1293.310091 | -0.523474053 | 0.065912063 | -7.942006837 | 1.99E-15 | 1.33E-12 | down |
| PKLR | 110.6616623 | -1.718272721 | 0.216553075 | -7.934649365 | 2.11E-15 | 1.38E-12 | down |
| ZSWIM3 | 315.776428 | -0.609132478 | 0.076800504 | -7.931360478 | 2.17E-15 | 1.39E-12 | down |
| SNHG11 | 463.5351006 | -0.541751269 | 0.068721563 | -7.883279233 | 3.19E-15 | 1.97E-12 | down |
| SCT | 24.63368235 | -1.375520613 | 0.174655176 | -7.875636126 | 3.39E-15 | 2.06E-12 | down |
| LPO | 29.36018323 | -1.610690479 | 0.205600424 | -7.834081508 | 4.72E-15 | 2.82E-12 | down |
| MOCS3 | 772.9430899 | -0.523137584 | 0.06692117 | -7.817221112 | 5.40E-15 | 3.17E-12 | down |
| AC007996.1 | 64.56505172 | 0.787181665 | 0.100923743 | 7.799766866 | 6.20E-15 | 3.58E-12 | up |
| ANK1 | 62.71517309 | 0.971171765 | 0.125209053 | 7.756402145 | 8.74E-15 | 4.95E-12 | up |
| AMT | 489.3469394 | -0.906896601 | 0.117170475 | -7.739975428 | 9.94E-15 | 5.54E-12 | down |
| FGD1 | 404.4873537 | -0.670894002 | 0.086861623 | -7.723710193 | 1.13E-14 | 6.19E-12 | down |
| SNPH | 183.4551153 | 0.997456248 | 0.129386032 | 7.709149392 | 1.27E-14 | 6.81E-12 | up |
| DNTTIP1 | 2608.271731 | -0.562945432 | 0.073233697 | -7.6869727 | 1.51E-14 | 7.75E-12 | down |
| AC067904.2 | 2.065676588 | 1.777342558 | 0.232302637 | 7.650978836 | 1.99E-14 | 1.01E-11 | up |
| ZNF492 | 5.882281881 | -1.853260564 | 0.242972259 | -7.627457446 | 2.39E-14 | 1.18E-11 | down |
| SUSD3 | 255.7349202 | -0.964510794 | 0.126456804 | -7.627195731 | 2.40E-14 | 1.18E-11 | down |
| ACOT8 | 1194.427843 | -0.53102672 | 0.069734151 | -7.615016629 | 2.64E-14 | 1.28E-11 | down |
| KRT7 | 333.1817025 | 1.16722056 | 0.153334261 | 7.612261959 | 2.69E-14 | 1.29E-11 | up |
| ZNF853 | 168.5001503 | -0.927912724 | 0.122085013 | -7.600545724 | 2.95E-14 | 1.39E-11 | down |
| ERGIC3 | 14154.90606 | -0.525665658 | 0.069325946 | -7.582524118 | 3.39E-14 | 1.57E-11 | down |
| KRT5 | 67.82828525 | 1.856352816 | 0.245044051 | 7.575588165 | 3.58E-14 | 1.64E-11 | up |
| ABCA4 | 16.65961468 | 1.333160901 | 0.176090225 | 7.570896688 | 3.71E-14 | 1.68E-11 | up |
| PLAGL2 | 3289.783874 | -0.643582514 | 0.085118964 | -7.56097683 | 4.00E-14 | 1.78E-11 | down |
| TFAP2A-AS1 | 31.01663486 | 1.240316657 | 0.164318214 | 7.548260351 | 4.41E-14 | 1.92E-11 | up |
| AC012531.1 | 4.042665366 | 2.148117481 | 0.285425001 | 7.52603126 | 5.23E-14 | 2.24E-11 | up |
| PCSK1N | 169.6229597 | -1.806320168 | 0.240341691 | -7.51563392 | 5.66E-14 | 2.40E-11 | down |
| ZNF610 | 37.58871511 | -0.991430004 | 0.132152868 | -7.502145188 | 6.28E-14 | 2.62E-11 | down |
| GP2 | 88.74820682 | 2.162546477 | 0.289008057 | 7.482651171 | 7.28E-14 | 3.01E-11 | up |
| ST6GAL2 | 129.589609 | -1.44205388 | 0.1927849 | -7.480118404 | 7.43E-14 | 3.03E-11 | down |
| CAMK2N2 | 18.287897 | 1.259501563 | 0.168928964 | 7.455805881 | 8.93E-14 | 3.56E-11 | up |
| AQP5 | 185.5638352 | 2.101856596 | 0.283986599 | 7.401252759 | 1.35E-13 | 5.25E-11 | up |
| SCARNA6 | 5.042085554 | -1.5515737 | 0.210231344 | -7.380315766 | 1.58E-13 | 6.06E-11 | down |
| C8orf33 | 3612.811787 | -0.563276901 | 0.076336021 | -7.378913547 | 1.60E-13 | 6.06E-11 | down |
| VSTM2B | 5.149469731 | -2.948677617 | 0.400504011 | -7.362417193 | 1.81E-13 | 6.79E-11 | down |
| TRIM7 | 283.0339212 | 1.320432771 | 0.179788127 | 7.344382481 | 2.07E-13 | 7.60E-11 | up |
| AC108134.3 | 95.54203917 | -1.168673113 | 0.159560715 | -7.32431608 | 2.40E-13 | 8.63E-11 | down |
| CEACAM20 | 3.160933845 | 1.781275676 | 0.243290293 | 7.321606029 | 2.45E-13 | 8.64E-11 | up |
| APOL2 | 1306.025682 | 0.607210736 | 0.08293696 | 7.321352744 | 2.45E-13 | 8.64E-11 | up |
| GGT7 | 947.5765896 | -0.790052154 | 0.108086584 | -7.309437733 | 2.68E-13 | 9.34E-11 | down |
| PYY | 52.33949088 | -1.828859263 | 0.251207624 | -7.280269726 | 3.33E-13 | 1.15E-10 | down |
| SNORA23 | 4.553498408 | -1.77803913 | 0.244344682 | -7.276766232 | 3.42E-13 | 1.17E-10 | down |
| MTCO1P40 | 106.3131672 | 1.381652925 | 0.18996298 | 7.273274634 | 3.51E-13 | 1.18E-10 | up |
| PANCR | 3.200910337 | 1.784858484 | 0.245427612 | 7.27244368 | 3.53E-13 | 1.18E-10 | up |
| AC046168.2 | 3.746389155 | 1.664493471 | 0.229768958 | 7.244205153 | 4.35E-13 | 1.44E-10 | up |
| CTXND1 | 15.41105817 | -1.551460376 | 0.215648187 | -7.194404913 | 6.27E-13 | 2.04E-10 | down |
| ZNF813 | 148.3921267 | -1.096557453 | 0.152663363 | -7.182846187 | 6.83E-13 | 2.20E-10 | down |
| FCGRT | 8081.600311 | -0.636630622 | 0.088660368 | -7.180554668 | 6.94E-13 | 2.21E-10 | down |
| LHFPL4 | 16.44673865 | -2.025425774 | 0.283065143 | -7.155334468 | 8.35E-13 | 2.62E-10 | down |
| HOXC4 | 33.80130337 | 1.277267982 | 0.178514988 | 7.154962158 | 8.37E-13 | 2.62E-10 | up |
| SEMG1 | 53.87137614 | 2.039794285 | 0.285579969 | 7.142637816 | 9.16E-13 | 2.83E-10 | up |
| HAGLR | 357.2890102 | 0.972239552 | 0.136146655 | 7.141119624 | 9.26E-13 | 2.84E-10 | up |
| AOAH | 879.347956 | -0.798509704 | 0.111957037 | -7.132286866 | 9.87E-13 | 3.00E-10 | down |
| AC126564.1 | 4.033470379 | 1.786679843 | 0.251114088 | 7.115012383 | 1.12E-12 | 3.37E-10 | up |
| TLE6 | 50.17839774 | 0.847415776 | 0.119164774 | 7.111294261 | 1.15E-12 | 3.43E-10 | up |
| HOXB2 | 187.6517434 | 0.838736995 | 0.11812156 | 7.100625787 | 1.24E-12 | 3.68E-10 | up |
| HSPB3 | 15.48191789 | -2.025458399 | 0.285391327 | -7.097126685 | 1.27E-12 | 3.74E-10 | down |
| ERFE | 95.38736239 | 1.062197716 | 0.150236558 | 7.070168073 | 1.55E-12 | 4.50E-10 | up |
| LGI4 | 135.5727864 | -0.921361554 | 0.130417529 | -7.064706406 | 1.61E-12 | 4.61E-10 | down |
| AC009318.4 | 5.089009809 | 1.19054289 | 0.168528182 | 7.064354921 | 1.61E-12 | 4.61E-10 | up |
| FBXO2 | 295.9105567 | -1.208523998 | 0.171809303 | -7.034101044 | 2.01E-12 | 5.68E-10 | down |
| TCEA2 | 253.5458848 | -0.751314325 | 0.107299079 | -7.00205751 | 2.52E-12 | 6.99E-10 | down |
| MAGEE1 | 49.17427788 | -0.948537302 | 0.135473705 | -7.001634018 | 2.53E-12 | 6.99E-10 | down |
| PIPOX | 235.7622746 | -1.26364507 | 0.180879847 | -6.986102048 | 2.83E-12 | 7.68E-10 | down |
| SPACA3 | 34.00549786 | -1.476704015 | 0.211625037 | -6.977926774 | 3.00E-12 | 8.01E-10 | down |
| MIOX | 14.78347873 | 1.346226613 | 0.193264681 | 6.965714631 | 3.27E-12 | 8.67E-10 | up |
| SCN5A | 30.02737985 | -1.151403183 | 0.165696151 | -6.94888309 | 3.68E-12 | 9.54E-10 | down |
| CFAP74 | 17.40323218 | -1.028691367 | 0.148354736 | -6.933997507 | 4.09E-12 | 1.05E-09 | down |
| HOXB6 | 1391.965823 | 0.819477291 | 0.118542696 | 6.912929447 | 4.75E-12 | 1.20E-09 | up |
| ROS1 | 18.33576814 | -1.526511022 | 0.221551834 | -6.890085228 | 5.58E-12 | 1.39E-09 | down |
| TRIB2 | 834.482188 | 0.742253232 | 0.107755684 | 6.888297698 | 5.65E-12 | 1.39E-09 | up |
| TSPY26P | 151.1472702 | -0.726388508 | 0.105643833 | -6.875825007 | 6.16E-12 | 1.49E-09 | down |
| AIRE | 11.29696656 | -1.363420384 | 0.198587363 | -6.865594874 | 6.62E-12 | 1.59E-09 | down |
| OIT3 | 55.70994281 | -1.068575269 | 0.155810888 | -6.858155296 | 6.98E-12 | 1.67E-09 | down |
| GAD1 | 129.4847341 | 1.141536052 | 0.167038503 | 6.833969598 | 8.26E-12 | 1.95E-09 | up |
| OSER1-AS1 | 303.3757491 | -0.688555715 | 0.100780721 | -6.83221662 | 8.36E-12 | 1.96E-09 | down |
| HOXC8 | 12.08596708 | 1.536724463 | 0.22506044 | 6.828052346 | 8.61E-12 | 1.99E-09 | up |
| COL2A1 | 30.64014431 | 1.381605541 | 0.202436389 | 6.824887303 | 8.80E-12 | 2.02E-09 | up |
| SRPX2 | 1104.49351 | -0.884753111 | 0.129692405 | -6.821934633 | 8.98E-12 | 2.04E-09 | down |
| APOL1 | 4225.699261 | 0.785925088 | 0.11522428 | 6.820828812 | 9.05E-12 | 2.05E-09 | up |
| TNFRSF14-AS1 | 406.5266361 | 0.619117797 | 0.090828666 | 6.816326011 | 9.34E-12 | 2.10E-09 | up |
| CPNE1 | 7946.215353 | -0.612531288 | 0.089936301 | -6.810723585 | 9.71E-12 | 2.16E-09 | down |
| ABAT | 962.3665156 | -0.786299026 | 0.115657974 | -6.798485229 | 1.06E-11 | 2.32E-09 | down |
| DUSP15 | 104.1612341 | -1.030397704 | 0.151581996 | -6.797625917 | 1.06E-11 | 2.32E-09 | down |
| ST18 | 25.60463345 | 1.176334006 | 0.173188708 | 6.792209619 | 1.10E-11 | 2.40E-09 | up |
| BST2 | 2986.741483 | 0.927056341 | 0.136760949 | 6.77866268 | 1.21E-11 | 2.60E-09 | up |
| AHCY | 17698.61914 | -0.513583346 | 0.075890837 | -6.767396016 | 1.31E-11 | 2.78E-09 | down |
| CIITA | 765.7660685 | 0.893326008 | 0.132227602 | 6.755972258 | 1.42E-11 | 2.98E-09 | up |
| KCNG1 | 69.5670952 | -1.209470032 | 0.179138422 | -6.751594769 | 1.46E-11 | 3.05E-09 | down |
| GLDC | 74.48155408 | -1.312768043 | 0.194453234 | -6.751073328 | 1.47E-11 | 3.05E-09 | down |
| RSPO4 | 14.56391178 | -1.775600664 | 0.263142014 | -6.747689728 | 1.50E-11 | 3.10E-09 | down |
| S100A14 | 6200.94697 | 0.662495258 | 0.098221403 | 6.744917474 | 1.53E-11 | 3.14E-09 | up |
| ZNF793-AS1 | 14.34239642 | -1.156631123 | 0.171547723 | -6.742328617 | 1.56E-11 | 3.18E-09 | down |
| LAG3 | 148.7971898 | 0.852098194 | 0.126552671 | 6.733150592 | 1.66E-11 | 3.34E-09 | up |
| CLDN8 | 33.29229272 | -2.383976633 | 0.354693786 | -6.721224689 | 1.80E-11 | 3.61E-09 | down |
| AL121832.1 | 41.23506758 | -1.086756733 | 0.162421603 | -6.69096175 | 2.22E-11 | 4.41E-09 | down |
| SEMG2 | 3.04451394 | 2.58175444 | 0.386637347 | 6.677457466 | 2.43E-11 | 4.81E-09 | up |
| CDIP1 | 464.4633463 | -0.724130634 | 0.108459501 | -6.676507133 | 2.45E-11 | 4.81E-09 | down |
| SNORA5A | 9.263810308 | -0.912161124 | 0.136705693 | -6.672444325 | 2.52E-11 | 4.92E-09 | down |
| CCK | 11.05181512 | 2.141546466 | 0.32114869 | 6.668395465 | 2.59E-11 | 5.00E-09 | up |
| HOXC9 | 15.37691445 | 1.35615416 | 0.204494974 | 6.631723676 | 3.32E-11 | 6.25E-09 | up |
| GABRP | 238.1002396 | 1.359358156 | 0.205385151 | 6.618580515 | 3.63E-11 | 6.74E-09 | up |
| ZFP28 | 85.0792356 | -0.895692834 | 0.135361264 | -6.617054303 | 3.66E-11 | 6.77E-09 | down |
| ZNF570 | 106.6561293 | -0.756862233 | 0.114440186 | -6.613605386 | 3.75E-11 | 6.89E-09 | down |
| TMPRSS6 | 39.17384041 | -0.888664216 | 0.134856531 | -6.589700988 | 4.41E-11 | 8.05E-09 | down |
| ROMO1 | 3506.586865 | -0.674257886 | 0.102426267 | -6.5828611 | 4.61E-11 | 8.39E-09 | down |
| TTC9B | 13.9614257 | 0.843209403 | 0.129236347 | 6.524553059 | 6.82E-11 | 1.21E-08 | up |
| RN7SKP9 | 1.431371735 | -2.463279626 | 0.377748808 | -6.520946121 | 6.99E-11 | 1.24E-08 | down |
| ADGRG6 | 888.1594688 | 0.809029146 | 0.124160743 | 6.515981839 | 7.22E-11 | 1.27E-08 | up |
| PCP4 | 116.7658829 | -1.568534805 | 0.240725521 | -6.515864213 | 7.23E-11 | 1.27E-08 | down |
| GNG4 | 1455.310982 | -1.240013454 | 0.190467697 | -6.510360926 | 7.50E-11 | 1.31E-08 | down |
| AC063926.3 | 2.770561287 | 1.540207214 | 0.237046567 | 6.497487954 | 8.17E-11 | 1.42E-08 | up |
| CHN2 | 942.3315742 | -0.688925807 | 0.106059086 | -6.495679269 | 8.27E-11 | 1.42E-08 | down |
| NR1H4 | 41.42572476 | 1.44945806 | 0.223330014 | 6.490207165 | 8.57E-11 | 1.47E-08 | up |
| NEURL2 | 33.33689949 | -0.59541379 | 0.091795801 | -6.486285665 | 8.80E-11 | 1.50E-08 | down |
| AL117382.1 | 293.0492682 | -0.855491381 | 0.131934174 | -6.484228854 | 8.92E-11 | 1.51E-08 | down |
| MEP1B | 33.30625378 | 1.24831552 | 0.192715556 | 6.477502612 | 9.33E-11 | 1.57E-08 | up |
| F10 | 230.6986731 | -1.028679779 | 0.159065851 | -6.467005792 | 1.00E-10 | 1.67E-08 | down |
| CLSTN2 | 133.4417015 | -1.147322968 | 0.177414914 | -6.466891324 | 1.00E-10 | 1.67E-08 | down |
| MAMLD1 | 101.8585166 | 0.805427036 | 0.124596661 | 6.464274656 | 1.02E-10 | 1.69E-08 | up |
| DLGAP1-AS5 | 26.61614178 | 2.513419571 | 0.388818457 | 6.464249633 | 1.02E-10 | 1.69E-08 | up |
| SNORA54 | 2.181288355 | -2.052663594 | 0.317622651 | -6.462585684 | 1.03E-10 | 1.69E-08 | down |
| DBH-AS1 | 22.38126046 | 0.898737142 | 0.139069204 | 6.462517346 | 1.03E-10 | 1.69E-08 | up |
| ATP9A | 7382.830276 | -0.534939908 | 0.082873891 | -6.454866539 | 1.08E-10 | 1.77E-08 | down |
| AC136475.5 | 10.27033404 | -0.969241713 | 0.150280003 | -6.449572066 | 1.12E-10 | 1.82E-08 | down |
| SLC9C2 | 1.057745166 | 1.86503493 | 0.289374476 | 6.445056779 | 1.16E-10 | 1.87E-08 | up |
| CKM | 12.70616514 | -1.000157228 | 0.155728855 | -6.422427168 | 1.34E-10 | 2.16E-08 | down |
| REC8 | 258.3441436 | 0.617456385 | 0.096353586 | 6.408234644 | 1.47E-10 | 2.36E-08 | up |
| AL133520.1 | 33.76347652 | -0.556430542 | 0.086862464 | -6.405880277 | 1.50E-10 | 2.38E-08 | down |
| YAE1D1 | 800.2424382 | -0.506002642 | 0.079041096 | -6.401766542 | 1.54E-10 | 2.43E-08 | down |
| ZNF528 | 114.4895843 | -0.803735517 | 0.125646851 | -6.396782009 | 1.59E-10 | 2.50E-08 | down |
| AC023043.4 | 13.90945015 | 0.554053412 | 0.086714495 | 6.389397886 | 1.67E-10 | 2.61E-08 | up |
| FOXD4L1 | 3.18359586 | 0.961759246 | 0.150951383 | 6.371317871 | 1.87E-10 | 2.93E-08 | up |
| RBP2 | 189.3892655 | -1.321233774 | 0.207530107 | -6.36646795 | 1.93E-10 | 3.01E-08 | down |
| RTBDN | 8.609029679 | 1.318726404 | 0.207276866 | 6.36214949 | 1.99E-10 | 3.08E-08 | up |
| TP53AIP1 | 5.766891124 | 1.09186066 | 0.17195514 | 6.349683165 | 2.16E-10 | 3.31E-08 | up |
| FABP7 | 3.810071095 | 1.927350341 | 0.303561444 | 6.349127595 | 2.17E-10 | 3.31E-08 | up |
| TFF2 | 216.7588772 | 1.397580127 | 0.220321354 | 6.343371174 | 2.25E-10 | 3.42E-08 | up |
| SLC13A3 | 314.1609976 | -1.298550074 | 0.204781058 | -6.341163033 | 2.28E-10 | 3.45E-08 | down |
| TEKT5 | 15.06283611 | -1.083180557 | 0.171382821 | -6.320239994 | 2.61E-10 | 3.92E-08 | down |
| ABCC8 | 11.12497521 | 1.081196996 | 0.171103993 | 6.318946596 | 2.63E-10 | 3.93E-08 | up |
| CDH26 | 23.55391253 | -0.966906073 | 0.153080833 | -6.316310505 | 2.68E-10 | 3.98E-08 | down |
| AL117335.1 | 10.64550092 | -1.158983094 | 0.183608365 | -6.312256479 | 2.75E-10 | 4.07E-08 | down |
| PRDM8 | 124.1861651 | 0.911720609 | 0.144532761 | 6.308055032 | 2.83E-10 | 4.16E-08 | up |
| SP140 | 91.2361001 | 0.792380173 | 0.125702851 | 6.303597448 | 2.91E-10 | 4.27E-08 | up |
| F5 | 306.4002596 | 1.21439199 | 0.192750086 | 6.300344751 | 2.97E-10 | 4.34E-08 | up |
| MAP3K6 | 784.3444816 | 0.519725292 | 0.082603788 | 6.291785207 | 3.14E-10 | 4.56E-08 | up |
| ABCA17P | 22.09074593 | 1.277014399 | 0.203232751 | 6.283506914 | 3.31E-10 | 4.78E-08 | up |
| GALNT9 | 43.39652037 | 1.38821996 | 0.220937626 | 6.283311645 | 3.31E-10 | 4.78E-08 | up |
| HLA-DMA | 1773.82643 | 0.708275798 | 0.112821614 | 6.277837864 | 3.43E-10 | 4.91E-08 | up |
| ZNF415 | 73.53807168 | -0.857404602 | 0.136592412 | -6.277102737 | 3.45E-10 | 4.91E-08 | down |
| CAPN14 | 18.36296408 | 1.245857817 | 0.198531935 | 6.275352215 | 3.49E-10 | 4.94E-08 | up |
| CARM1P1 | 0.974992239 | -2.529484782 | 0.403252119 | -6.272712937 | 3.55E-10 | 5.01E-08 | down |
| XKR7 | 7.363468719 | 1.702844013 | 0.272192457 | 6.256029409 | 3.95E-10 | 5.50E-08 | up |
| DRGX | 20.62075569 | 1.872239337 | 0.299384074 | 6.253637046 | 4.01E-10 | 5.56E-08 | up |
| SLC2A5 | 200.753335 | 0.766611875 | 0.122668845 | 6.249442349 | 4.12E-10 | 5.69E-08 | up |
| AC136475.7 | 5.974922779 | -0.988921398 | 0.158286928 | -6.247650472 | 4.17E-10 | 5.73E-08 | down |
| GZMA | 209.2938134 | 0.835337907 | 0.133733173 | 6.24630288 | 4.20E-10 | 5.76E-08 | up |
| KRBOX1 | 4.875963028 | -1.303208905 | 0.20873638 | -6.243324268 | 4.28E-10 | 5.82E-08 | down |
| FOXD4 | 11.96626078 | 0.824665349 | 0.132100158 | 6.242727938 | 4.30E-10 | 5.82E-08 | up |
| DAPK1 | 728.9834055 | 0.798650152 | 0.128026257 | 6.238174677 | 4.43E-10 | 5.97E-08 | up |
| FAM83C | 11.17935129 | -1.631024197 | 0.261683285 | -6.232817659 | 4.58E-10 | 6.15E-08 | down |
| CFAP46 | 15.49347444 | 1.327857977 | 0.213094862 | 6.231299822 | 4.63E-10 | 6.17E-08 | up |
| TSPAN6 | 5216.958846 | -0.524630642 | 0.084330793 | -6.221104119 | 4.94E-10 | 6.47E-08 | down |
| FOXD1 | 91.56589889 | 1.349973607 | 0.217056813 | 6.219448222 | 4.99E-10 | 6.52E-08 | up |
| AL138789.1 | 6.706987297 | 1.088698692 | 0.175144455 | 6.216004354 | 5.10E-10 | 6.61E-08 | up |
| AC005696.4 | 24.8999181 | 0.940056214 | 0.151814704 | 6.192128884 | 5.94E-10 | 7.66E-08 | up |
| ZNF347 | 148.3569874 | -0.685377403 | 0.110734234 | -6.189390358 | 6.04E-10 | 7.77E-08 | down |
| SYT12 | 35.2892388 | 0.975248684 | 0.15774573 | 6.182409411 | 6.31E-10 | 8.07E-08 | up |
| PDCD1 | 66.20533401 | 0.767920702 | 0.124214719 | 6.182203768 | 6.32E-10 | 8.07E-08 | up |
| SMC1B | 11.34526707 | 0.993043372 | 0.160695953 | 6.179641447 | 6.42E-10 | 8.17E-08 | up |
| AC107302.1 | 2.090309615 | 3.569268125 | 0.577776477 | 6.177593349 | 6.51E-10 | 8.24E-08 | up |
| IL34 | 156.1399301 | -0.678227095 | 0.109885873 | -6.172104565 | 6.74E-10 | 8.50E-08 | down |
| AC098934.2 | 22.39650698 | -0.6118168 | 0.099144451 | -6.170963643 | 6.79E-10 | 8.53E-08 | down |
| DNAH2 | 204.0701449 | 0.810597708 | 0.131459762 | 6.166127904 | 7.00E-10 | 8.74E-08 | up |
| MPP1 | 1087.365697 | -0.656868904 | 0.106529978 | -6.166047483 | 7.00E-10 | 8.74E-08 | down |
| UNC5A | 34.1399463 | 1.155999928 | 0.187506323 | 6.165125032 | 7.04E-10 | 8.76E-08 | up |
| COLEC11 | 31.63985679 | -0.763480167 | 0.123999184 | -6.157138626 | 7.41E-10 | 9.17E-08 | down |
| AC008514.1 | 4.383575646 | 1.316847778 | 0.214152533 | 6.149111375 | 7.79E-10 | 9.61E-08 | up |
| PLTP | 3314.62393 | -0.680447049 | 0.110793893 | -6.141557358 | 8.17E-10 | 9.97E-08 | down |
| CX3CL1 | 941.092092 | 0.695326785 | 0.113744215 | 6.113073842 | 9.77E-10 | 1.17E-07 | up |
| MT1X | 612.0164502 | 0.598860029 | 0.098003884 | 6.110574444 | 9.93E-10 | 1.18E-07 | up |
| GBGT1 | 180.0398527 | -0.609338836 | 0.09972395 | -6.110255705 | 9.95E-10 | 1.18E-07 | down |
| RPL39P40 | 15.48160414 | -0.939422676 | 0.153796382 | -6.108223507 | 1.01E-09 | 1.19E-07 | down |
| TBX10 | 117.5480774 | -1.086940037 | 0.178001642 | -6.106348374 | 1.02E-09 | 1.20E-07 | down |
| AC022613.1 | 63.82907529 | -1.122975297 | 0.18432699 | -6.092299874 | 1.11E-09 | 1.31E-07 | down |
| STK4-AS1 | 10.09472024 | -0.678082736 | 0.111358562 | -6.089183652 | 1.13E-09 | 1.33E-07 | down |
| KLRG2 | 36.86234864 | -1.515633644 | 0.249229879 | -6.081267817 | 1.19E-09 | 1.39E-07 | down |
| SNORA73B | 48.77227555 | -0.950098039 | 0.156565122 | -6.068388851 | 1.29E-09 | 1.49E-07 | down |
| MSH4 | 11.96372402 | 1.176992453 | 0.193978793 | 6.067634692 | 1.30E-09 | 1.49E-07 | up |
| CCDC68 | 399.1176029 | 0.617383332 | 0.101767051 | 6.066632849 | 1.31E-09 | 1.50E-07 | up |
| TMEM92 | 456.4355164 | 0.632515757 | 0.104325656 | 6.062897451 | 1.34E-09 | 1.53E-07 | up |
| LYG1 | 27.48976071 | 0.699255189 | 0.11543847 | 6.057384428 | 1.38E-09 | 1.57E-07 | up |
| RPE65 | 4.772605324 | 1.591919992 | 0.263040095 | 6.052005078 | 1.43E-09 | 1.62E-07 | up |
| DRD2 | 151.9959166 | -1.360452189 | 0.224851515 | -6.050447071 | 1.44E-09 | 1.63E-07 | down |
| ULBP2 | 76.66210351 | 0.9134797 | 0.151426805 | 6.032483477 | 1.61E-09 | 1.81E-07 | up |
| ZNF461 | 90.83759676 | -0.598786774 | 0.099357471 | -6.026590335 | 1.67E-09 | 1.87E-07 | down |
| LINC00342 | 246.8923183 | 0.60606161 | 0.100588563 | 6.02515425 | 1.69E-09 | 1.88E-07 | up |
| MUC16 | 46.70441294 | 1.510853795 | 0.251035763 | 6.018480292 | 1.76E-09 | 1.95E-07 | up |
| LINC00658 | 4.475460538 | -1.471887103 | 0.244717429 | -6.014639445 | 1.80E-09 | 1.98E-07 | down |
| CARD17 | 3.762039062 | 1.197580537 | 0.199169963 | 6.012857159 | 1.82E-09 | 2.00E-07 | up |
| LMO4 | 1178.278968 | 0.51484182 | 0.08570861 | 6.006885637 | 1.89E-09 | 2.06E-07 | up |
| HSD17B2 | 559.6633858 | 0.936193984 | 0.155892067 | 6.005398493 | 1.91E-09 | 2.07E-07 | up |
| AGAP2 | 198.27061 | 0.757275725 | 0.126130794 | 6.003892476 | 1.93E-09 | 2.08E-07 | up |
| AP006284.1 | 62.14988043 | 0.765492637 | 0.127503304 | 6.003708261 | 1.93E-09 | 2.08E-07 | up |
| TBX3 | 1313.252225 | 0.63421329 | 0.105656627 | 6.002588833 | 1.94E-09 | 2.09E-07 | up |
| STS | 735.906104 | 0.55036539 | 0.09172809 | 5.999965647 | 1.97E-09 | 2.11E-07 | up |
| GNLY | 253.7476115 | 0.91483349 | 0.152675899 | 5.99199674 | 2.07E-09 | 2.21E-07 | up |
| CCNB3 | 13.84338328 | 0.763482592 | 0.127436335 | 5.991090327 | 2.08E-09 | 2.21E-07 | up |
| MT3 | 53.4568135 | -1.279380371 | 0.213792464 | -5.984216404 | 2.17E-09 | 2.30E-07 | down |
| R3HDML | 59.9008466 | -0.918601399 | 0.153712354 | -5.976106513 | 2.29E-09 | 2.40E-07 | down |
| SCAND1 | 2727.186929 | -0.524720467 | 0.087951407 | -5.966026975 | 2.43E-09 | 2.52E-07 | down |
| KLRK1 | 8.285271497 | 0.83154737 | 0.140015081 | 5.938984334 | 2.87E-09 | 2.96E-07 | up |
| VWA5B2 | 34.39984739 | 1.045138626 | 0.176010068 | 5.937947961 | 2.89E-09 | 2.96E-07 | up |
| GCNT3 | 3615.562677 | 0.885829804 | 0.149185254 | 5.937783928 | 2.89E-09 | 2.96E-07 | up |
| PLA2G12B | 153.710286 | -1.404354701 | 0.236680588 | -5.933544069 | 2.96E-09 | 3.02E-07 | down |
| SLC1A7 | 253.9857144 | -1.110136506 | 0.187229388 | -5.929285545 | 3.04E-09 | 3.09E-07 | down |
| SLC7A4 | 173.3677139 | -0.90478323 | 0.15262614 | -5.928101373 | 3.06E-09 | 3.11E-07 | down |
| ADGRG2 | 34.51613491 | -0.953299442 | 0.161188029 | -5.914207444 | 3.33E-09 | 3.37E-07 | down |
| ABCC6P2 | 23.63932126 | -0.737579984 | 0.124821322 | -5.909086443 | 3.44E-09 | 3.47E-07 | down |
| SNORD17 | 37.87011404 | -0.884258504 | 0.149680578 | -5.907636897 | 3.47E-09 | 3.49E-07 | down |
| LINC00896 | 36.94797038 | 0.763965992 | 0.129373541 | 5.905117746 | 3.52E-09 | 3.53E-07 | up |
| AC022784.1 | 11.20736305 | 1.177236452 | 0.199504478 | 5.900802154 | 3.62E-09 | 3.60E-07 | up |
| ADGRF1 | 99.28196406 | 1.100455998 | 0.186520578 | 5.899917378 | 3.64E-09 | 3.61E-07 | up |
| ZNF471 | 43.52586703 | -0.802993555 | 0.136169073 | -5.897033272 | 3.70E-09 | 3.66E-07 | down |
| AC093908.1 | 36.57826541 | -0.760713922 | 0.129079117 | -5.893392658 | 3.78E-09 | 3.73E-07 | down |
| WNT10B | 24.23550365 | 0.778691815 | 0.13225505 | 5.887804023 | 3.91E-09 | 3.84E-07 | up |
| ATP8A1 | 1021.05767 | 0.55923648 | 0.095001015 | 5.886636884 | 3.94E-09 | 3.85E-07 | up |
| GBP4 | 1465.705308 | 0.840179887 | 0.142739758 | 5.886095762 | 3.95E-09 | 3.86E-07 | up |
| AC008443.6 | 3.292579923 | 0.922310279 | 0.156765983 | 5.883357219 | 4.02E-09 | 3.91E-07 | up |
| CCL5 | 655.6541665 | 0.737024042 | 0.125468249 | 5.874187656 | 4.25E-09 | 4.10E-07 | up |
| MYRF | 1563.309776 | 0.756831179 | 0.128848023 | 5.873828439 | 4.26E-09 | 4.10E-07 | up |
| SPRR1B | 30.70027941 | 1.935113226 | 0.329609092 | 5.870933999 | 4.33E-09 | 4.14E-07 | up |
| SKOR1 | 18.02908079 | 0.623264418 | 0.106167418 | 5.870580934 | 4.34E-09 | 4.14E-07 | up |
| NKG7 | 188.2812799 | 0.766300258 | 0.130641247 | 5.865683885 | 4.47E-09 | 4.24E-07 | up |
| CA14 | 19.74222112 | 0.577819907 | 0.098519279 | 5.865043993 | 4.49E-09 | 4.24E-07 | up |
| GRAMD1B | 219.9669121 | 0.802132835 | 0.136908587 | 5.858893509 | 4.66E-09 | 4.36E-07 | up |
| C19orf38 | 48.45008279 | 0.707661455 | 0.121038811 | 5.84656648 | 5.02E-09 | 4.66E-07 | up |
| AC011298.1 | 44.47641822 | -0.953705659 | 0.163413658 | -5.836144124 | 5.34E-09 | 4.95E-07 | down |
| PAX4 | 9.97458441 | 1.872236171 | 0.320822775 | 5.835733358 | 5.36E-09 | 4.95E-07 | up |
| KRT7-AS | 7.099389095 | 0.871205436 | 0.149307293 | 5.834982462 | 5.38E-09 | 4.96E-07 | up |
| ALKAL1 | 63.71297011 | -0.875270829 | 0.150077908 | -5.832109753 | 5.47E-09 | 5.03E-07 | down |
| LINC01260 | 12.49750791 | 1.068715287 | 0.183392227 | 5.827484102 | 5.63E-09 | 5.16E-07 | up |
| AC009318.1 | 19.68697325 | 0.757230285 | 0.129989425 | 5.825322233 | 5.70E-09 | 5.21E-07 | up |
| PNMA2 | 168.2595974 | 0.921702372 | 0.158594327 | 5.811698229 | 6.18E-09 | 5.62E-07 | up |
| LINC01315 | 166.6914882 | -0.577555941 | 0.099415789 | -5.809499154 | 6.27E-09 | 5.68E-07 | down |
| SLC17A9 | 1856.641585 | -0.663744473 | 0.114350281 | -5.804484848 | 6.46E-09 | 5.83E-07 | down |
| SLC38A3 | 42.0093669 | -1.319702142 | 0.227372089 | -5.804151906 | 6.47E-09 | 5.83E-07 | down |
| RNU4-2 | 36.49879308 | -1.547967257 | 0.267212192 | -5.793026312 | 6.91E-09 | 6.18E-07 | down |
| AC092198.1 | 7.077069644 | 1.107236937 | 0.191278017 | 5.788626172 | 7.10E-09 | 6.33E-07 | up |
| ZNF549 | 86.85532643 | -0.74000096 | 0.128008865 | -5.780857123 | 7.43E-09 | 6.59E-07 | down |
| BHLHA9 | 5.052745693 | -1.706114736 | 0.295244654 | -5.778647344 | 7.53E-09 | 6.64E-07 | down |
| ZNF829 | 27.2945852 | -0.712420132 | 0.123319226 | -5.777040258 | 7.60E-09 | 6.69E-07 | down |
| PIWIL2 | 197.9710091 | -0.682771613 | 0.118320194 | -5.7705417 | 7.90E-09 | 6.90E-07 | down |
| VSTM5 | 82.87469679 | 0.833687241 | 0.144580013 | 5.766268963 | 8.10E-09 | 7.04E-07 | up |
| CA8 | 363.466368 | 1.082962801 | 0.187821743 | 5.765907535 | 8.12E-09 | 7.04E-07 | up |
| TLE2 | 1855.946012 | -0.702871906 | 0.121994908 | -5.761485621 | 8.34E-09 | 7.20E-07 | down |
| ASPHD2 | 319.4235642 | 0.574260307 | 0.099712296 | 5.75917244 | 8.45E-09 | 7.26E-07 | up |
| BTNL10 | 6.328285955 | 0.750722426 | 0.130358262 | 5.758917099 | 8.47E-09 | 7.26E-07 | up |
| TNNC2 | 523.037119 | -1.005841578 | 0.174829672 | -5.753265834 | 8.75E-09 | 7.49E-07 | down |
| RHPN1-AS1 | 49.78860384 | -0.617877785 | 0.107418788 | -5.752045764 | 8.82E-09 | 7.52E-07 | down |
| ESPN | 1139.635575 | -0.675268669 | 0.117432224 | -5.750284279 | 8.91E-09 | 7.58E-07 | down |
| AC004009.2 | 2.167676313 | 1.778533739 | 0.309329898 | 5.749634136 | 8.94E-09 | 7.59E-07 | up |
| HMGN2P6 | 2.667900783 | 0.960381086 | 0.167112895 | 5.746899939 | 9.09E-09 | 7.68E-07 | up |
| UGT1A1 | 16.10359892 | 1.317492622 | 0.22953998 | 5.739708717 | 9.48E-09 | 7.99E-07 | up |
| EXOC3L4 | 199.7140168 | 0.58206077 | 0.10143726 | 5.738135792 | 9.57E-09 | 8.04E-07 | up |
| SOAT2 | 7.449220728 | -1.480427484 | 0.258119074 | -5.735443969 | 9.73E-09 | 8.15E-07 | down |
| AC139749.1 | 54.15387144 | 0.831811082 | 0.145225378 | 5.727725381 | 1.02E-08 | 8.51E-07 | up |
| CYP3A4 | 21.23153171 | 0.99840543 | 0.174426526 | 5.723931175 | 1.04E-08 | 8.67E-07 | up |
| LOXL1-AS1 | 91.88260035 | 0.688128944 | 0.120385004 | 5.716068608 | 1.09E-08 | 9.04E-07 | up |
| PAX9 | 85.8621726 | 0.854955187 | 0.149579636 | 5.715719118 | 1.09E-08 | 9.04E-07 | up |
| TCAF2 | 71.32645183 | 0.569275106 | 0.099623282 | 5.714277757 | 1.10E-08 | 9.10E-07 | up |
| ACTBL2 | 2.860498997 | 1.433287184 | 0.250908671 | 5.712386026 | 1.11E-08 | 9.18E-07 | up |
| FAM72C | 7.51609116 | 0.862086968 | 0.150930681 | 5.711807321 | 1.12E-08 | 9.18E-07 | up |
| CLCA2 | 15.50285317 | 1.364848582 | 0.239061575 | 5.70919262 | 1.14E-08 | 9.30E-07 | up |
| PNPLA3 | 50.88348949 | 1.04650099 | 0.183427137 | 5.705268078 | 1.16E-08 | 9.50E-07 | up |
| AMH | 151.4709223 | 1.097764039 | 0.192426853 | 5.704838071 | 1.16E-08 | 9.50E-07 | up |
| RHOV | 257.7741874 | 0.719368452 | 0.126148614 | 5.702547423 | 1.18E-08 | 9.60E-07 | up |
| HOXB-AS1 | 16.38575544 | 0.593522968 | 0.10425267 | 5.693120095 | 1.25E-08 | 1.01E-06 | up |
| AGT | 962.9504175 | -0.655338943 | 0.11513956 | -5.691692255 | 1.26E-08 | 1.02E-06 | down |
| PRSS27 | 18.97731806 | 0.622019394 | 0.109311803 | 5.690322352 | 1.27E-08 | 1.02E-06 | up |
| SORD2P | 118.226101 | 0.651918424 | 0.114715089 | 5.682935239 | 1.32E-08 | 1.06E-06 | up |
| INPP5J | 533.0001641 | -0.509770608 | 0.089809121 | -5.676156309 | 1.38E-08 | 1.10E-06 | down |
| HIST1H2BI | 2.743955527 | -1.452270557 | 0.256056872 | -5.671671869 | 1.41E-08 | 1.13E-06 | down |
| PRSS23 | 5409.080087 | -0.548383182 | 0.096825161 | -5.66364341 | 1.48E-08 | 1.18E-06 | down |
| AC021534.1 | 1.944462133 | 2.348989586 | 0.414816377 | 5.66272142 | 1.49E-08 | 1.19E-06 | up |
| DRD5 | 10.09352346 | 1.900199441 | 0.335701057 | 5.660391599 | 1.51E-08 | 1.20E-06 | up |
| AC092112.1 | 5.494698212 | 1.350951209 | 0.238920121 | 5.654405351 | 1.56E-08 | 1.24E-06 | up |
| LRRC37A3 | 186.2055293 | -0.551721482 | 0.097596458 | -5.65308919 | 1.58E-08 | 1.24E-06 | down |
| SLC23A1 | 73.5659025 | -0.757240918 | 0.133954485 | -5.652971743 | 1.58E-08 | 1.24E-06 | down |
| GZMH | 62.31004505 | 0.804655226 | 0.142370768 | 5.651828918 | 1.59E-08 | 1.25E-06 | up |
| AHRR | 59.94479718 | 0.607878574 | 0.107620521 | 5.648351903 | 1.62E-08 | 1.27E-06 | up |
| SLC25A48 | 36.42625468 | 0.872752992 | 0.154586566 | 5.645723406 | 1.64E-08 | 1.29E-06 | up |
| USP18 | 209.4176051 | 0.53100069 | 0.094079549 | 5.644167063 | 1.66E-08 | 1.29E-06 | up |
| RNF125 | 303.3143021 | 0.569552893 | 0.10093527 | 5.642753962 | 1.67E-08 | 1.30E-06 | up |
| GTF2F2P1 | 3.497984265 | -0.998831055 | 0.177091343 | -5.6402026 | 1.70E-08 | 1.31E-06 | down |
| PIWIL1 | 289.7766268 | 1.256778125 | 0.222834457 | 5.639963135 | 1.70E-08 | 1.31E-06 | up |
| AC139491.2 | 16.84332035 | 0.973111045 | 0.172568639 | 5.638979652 | 1.71E-08 | 1.32E-06 | up |
| EMX1 | 42.49431293 | 1.344171567 | 0.238399276 | 5.638320682 | 1.72E-08 | 1.32E-06 | up |
| AC021683.2 | 16.31686073 | -1.21533803 | 0.215560638 | -5.638033176 | 1.72E-08 | 1.32E-06 | down |
| AC005256.1 | 13.85831153 | 1.13699315 | 0.201834566 | 5.633292515 | 1.77E-08 | 1.35E-06 | up |
| ZNF663P | 21.42603269 | -1.543034634 | 0.274300579 | -5.625342246 | 1.85E-08 | 1.41E-06 | down |
| SPATA25 | 42.15433287 | -0.651751591 | 0.115901161 | -5.623339638 | 1.87E-08 | 1.42E-06 | down |
| LINC01535 | 3.432818274 | -1.181168235 | 0.210219747 | -5.618731112 | 1.92E-08 | 1.45E-06 | down |
| SPTBN4 | 24.20999777 | 0.847386325 | 0.150839765 | 5.617791358 | 1.93E-08 | 1.45E-06 | up |
| SMTNL2 | 61.65104915 | -0.982664285 | 0.175244444 | -5.607391957 | 2.05E-08 | 1.53E-06 | down |
| ZNF793 | 65.15811329 | -0.872276543 | 0.155611022 | -5.605493296 | 2.08E-08 | 1.54E-06 | down |
| FADS6 | 26.56479414 | -1.484574608 | 0.264856344 | -5.605206902 | 2.08E-08 | 1.54E-06 | down |
| IDO1 | 576.3951492 | 0.972239215 | 0.173483849 | 5.604205933 | 2.09E-08 | 1.54E-06 | up |
| IGSF22 | 40.60018706 | -0.619162712 | 0.110497308 | -5.603418958 | 2.10E-08 | 1.54E-06 | down |
| CARD11 | 596.9366739 | -0.875242277 | 0.156207053 | -5.603090635 | 2.11E-08 | 1.54E-06 | down |
| LARP6 | 182.4146363 | -0.596260671 | 0.10646415 | -5.600577017 | 2.14E-08 | 1.56E-06 | down |
| SLC35D3 | 235.7681967 | -1.11025979 | 0.198408209 | -5.595835944 | 2.20E-08 | 1.60E-06 | down |
| ZG16 | 1909.880563 | -1.497691764 | 0.267914956 | -5.59017603 | 2.27E-08 | 1.64E-06 | down |
| TM4SF4 | 108.639609 | 1.23169055 | 0.220384222 | 5.588832722 | 2.29E-08 | 1.65E-06 | up |
| CTNNA2 | 46.91146876 | -1.346093477 | 0.240998843 | -5.585476932 | 2.33E-08 | 1.67E-06 | down |
| AC010998.3 | 8.342567256 | 1.596452905 | 0.28595503 | 5.582881012 | 2.37E-08 | 1.69E-06 | up |
| FASLG | 27.6609878 | 0.743967756 | 0.133312939 | 5.580611746 | 2.40E-08 | 1.71E-06 | up |
| MAJIN | 1.758378728 | 1.25483832 | 0.224871896 | 5.580236302 | 2.40E-08 | 1.71E-06 | up |
| HOMER2 | 65.14727186 | 0.933228081 | 0.167265632 | 5.579317566 | 2.41E-08 | 1.71E-06 | up |
| AC083805.1 | 5.669766161 | -0.78330529 | 0.1405323 | -5.573845218 | 2.49E-08 | 1.76E-06 | down |
| RARRES3 | 1301.855393 | 0.783523671 | 0.140654309 | 5.570562878 | 2.54E-08 | 1.79E-06 | up |
| CD55 | 5968.141956 | 0.615441576 | 0.110565414 | 5.566311881 | 2.60E-08 | 1.83E-06 | up |
| MUC5AC | 1925.043773 | 1.476410799 | 0.265303873 | 5.564980187 | 2.62E-08 | 1.84E-06 | up |
| KIR2DL4 | 12.73656768 | 1.005512756 | 0.180703004 | 5.564449592 | 2.63E-08 | 1.84E-06 | up |
| ZNF568 | 50.53345098 | -0.618611785 | 0.111261853 | -5.559962983 | 2.70E-08 | 1.89E-06 | down |
| AL353616.2 | 2.872046998 | -1.173051465 | 0.21103403 | -5.558589128 | 2.72E-08 | 1.90E-06 | down |
| OTX1 | 107.0042473 | 0.722586482 | 0.130070618 | 5.555339808 | 2.77E-08 | 1.93E-06 | up |
| HIST1H2BB | 1.595091745 | -2.511488554 | 0.452706907 | -5.547714236 | 2.89E-08 | 2.00E-06 | down |
| RPS3AP54 | 0.774292305 | -1.656775235 | 0.298773812 | -5.545249172 | 2.94E-08 | 2.02E-06 | down |
| GDPD5 | 1302.361473 | -0.635374848 | 0.114661075 | -5.541329959 | 3.00E-08 | 2.07E-06 | down |
| AC025154.2 | 20.98252926 | 1.059960158 | 0.191359898 | 5.539092415 | 3.04E-08 | 2.09E-06 | up |
| SLC6A4 | 136.01437 | -1.102571029 | 0.199080125 | -5.53832798 | 3.05E-08 | 2.09E-06 | down |
| HOXB5 | 739.0607999 | 0.626284346 | 0.113096808 | 5.537595249 | 3.07E-08 | 2.10E-06 | up |
| AL158151.1 | 16.36937887 | -0.922610636 | 0.166683741 | -5.535096769 | 3.11E-08 | 2.12E-06 | down |
| NOS2 | 2272.771964 | 0.928534797 | 0.167953654 | 5.528517988 | 3.23E-08 | 2.20E-06 | up |
| TOX2 | 110.5046791 | 0.543623914 | 0.098360019 | 5.526878879 | 3.26E-08 | 2.21E-06 | up |
| MAPK11 | 155.5361356 | 0.527693666 | 0.095497942 | 5.525707208 | 3.28E-08 | 2.22E-06 | up |
| AP000439.2 | 128.1360659 | -0.611785957 | 0.1107598 | -5.523537928 | 3.32E-08 | 2.24E-06 | down |
| TMEM92-AS1 | 12.35989347 | 0.648250446 | 0.117386494 | 5.52235971 | 3.34E-08 | 2.25E-06 | up |
| ARX | 22.11516763 | 1.360095613 | 0.246347822 | 5.521037698 | 3.37E-08 | 2.26E-06 | up |
| BTNL9 | 303.7714974 | 0.657316698 | 0.119065459 | 5.520632951 | 3.38E-08 | 2.26E-06 | up |
| PCDHGB4 | 17.6939835 | -1.034738703 | 0.187520724 | -5.517996533 | 3.43E-08 | 2.29E-06 | down |
| SNORA49 | 1.353480667 | -2.048283651 | 0.37176605 | -5.509603825 | 3.60E-08 | 2.38E-06 | down |
| GSDMC | 14.34624028 | 0.740532315 | 0.13473673 | 5.496142838 | 3.88E-08 | 2.55E-06 | up |
| GFI1 | 146.2202519 | 0.704847986 | 0.12824851 | 5.495954602 | 3.89E-08 | 2.55E-06 | up |
| SLC19A3 | 327.9192867 | -0.847796353 | 0.154268296 | -5.495596783 | 3.89E-08 | 2.55E-06 | down |
| GJB5 | 167.2909252 | 1.263854919 | 0.230119105 | 5.49217728 | 3.97E-08 | 2.59E-06 | up |
| CAPS | 587.6772922 | -0.661767181 | 0.120631938 | -5.485837277 | 4.12E-08 | 2.67E-06 | down |
| DIO2 | 357.1731498 | -0.605104405 | 0.110340393 | -5.483979036 | 4.16E-08 | 2.70E-06 | down |
| ISG20 | 761.3306193 | 0.523275339 | 0.095436931 | 5.48294388 | 4.18E-08 | 2.70E-06 | up |
| AC108865.1 | 78.56546356 | -1.738317273 | 0.317059305 | -5.482625004 | 4.19E-08 | 2.70E-06 | down |
| CYP2W1 | 1990.582102 | -1.215491712 | 0.221699149 | -5.482617847 | 4.19E-08 | 2.70E-06 | down |
| HOTAIR | 21.56947123 | 1.753065992 | 0.31990473 | 5.479962715 | 4.25E-08 | 2.73E-06 | up |
| CRAT37 | 13.82752377 | -1.423872874 | 0.260116482 | -5.47398174 | 4.40E-08 | 2.81E-06 | down |
| HOXD3 | 12.89026772 | 0.690291036 | 0.126220722 | 5.468920046 | 4.53E-08 | 2.88E-06 | up |
| SLC14A2 | 15.07909673 | 1.338085743 | 0.245010044 | 5.461350564 | 4.73E-08 | 3.00E-06 | up |
| TIAM1 | 133.2326396 | 0.677228546 | 0.124076756 | 5.45814194 | 4.81E-08 | 3.04E-06 | up |
| ETNK2 | 105.0603605 | -0.672451817 | 0.123273902 | -5.454940622 | 4.90E-08 | 3.09E-06 | down |
| HOXC-AS3 | 1.224338039 | 2.082362119 | 0.381756001 | 5.45469387 | 4.91E-08 | 3.09E-06 | up |
| AC022893.1 | 24.99252797 | 0.625966739 | 0.114790411 | 5.45312744 | 4.95E-08 | 3.11E-06 | up |
| AC112484.4 | 10.90026698 | 0.956157896 | 0.175514466 | 5.447744108 | 5.10E-08 | 3.19E-06 | up |
| MYRFL | 31.80077525 | 1.111663406 | 0.204084458 | 5.44707528 | 5.12E-08 | 3.20E-06 | up |
| RHOXF1-AS1 | 11.68650994 | 0.80205191 | 0.147245197 | 5.447049739 | 5.12E-08 | 3.20E-06 | up |
| AC026801.3 | 26.87011276 | -0.632896824 | 0.116211674 | -5.446069239 | 5.15E-08 | 3.21E-06 | down |
| CHRM1 | 50.7349085 | 0.935578262 | 0.171809075 | 5.445453107 | 5.17E-08 | 3.21E-06 | up |
| SOWAHA | 559.7460875 | -0.569013796 | 0.104509983 | -5.444587985 | 5.19E-08 | 3.22E-06 | down |
| CES1P1 | 14.73990406 | -1.652145078 | 0.303625957 | -5.44138286 | 5.29E-08 | 3.27E-06 | down |
| AC091179.2 | 34.4234928 | -2.073339544 | 0.381619789 | -5.432997989 | 5.54E-08 | 3.42E-06 | down |
| HRK | 5.172136842 | 1.22122662 | 0.224953495 | 5.428795952 | 5.67E-08 | 3.49E-06 | up |
| AC108451.2 | 1.12432769 | 1.970036408 | 0.362920948 | 5.42827968 | 5.69E-08 | 3.49E-06 | up |
| LINC00543 | 169.1445234 | -0.537488894 | 0.099071626 | -5.425255606 | 5.79E-08 | 3.54E-06 | down |
| SIRPG | 63.47561305 | 0.638058642 | 0.117641505 | 5.423754485 | 5.84E-08 | 3.56E-06 | up |
| ARID3A | 1626.936857 | -0.583916565 | 0.107677176 | -5.422844348 | 5.87E-08 | 3.57E-06 | down |
| FBXL16 | 288.0217295 | 0.777123375 | 0.143310001 | 5.422673701 | 5.87E-08 | 3.57E-06 | up |
| CD164L2 | 14.09948592 | 0.817894608 | 0.150961504 | 5.41790184 | 6.03E-08 | 3.64E-06 | up |
| AC108474.1 | 13.70964973 | -1.797374626 | 0.331752477 | -5.417818255 | 6.03E-08 | 3.64E-06 | down |
| AGR2 | 31461.34096 | 0.668326542 | 0.12353539 | 5.410000683 | 6.30E-08 | 3.78E-06 | up |
| AC021205.3 | 4.756990241 | 0.677292366 | 0.125211552 | 5.409184358 | 6.33E-08 | 3.79E-06 | up |
| B3GNT4 | 47.8602263 | 0.590950585 | 0.109270731 | 5.408132425 | 6.37E-08 | 3.81E-06 | up |
| DNAAF3 | 39.13204196 | 0.735270226 | 0.136192138 | 5.398771465 | 6.71E-08 | 4.00E-06 | up |
| RSPH1 | 183.8612925 | 0.509089022 | 0.094382323 | 5.393902219 | 6.89E-08 | 4.08E-06 | up |
| GPC3 | 327.3815896 | 0.936150897 | 0.173702133 | 5.38940357 | 7.07E-08 | 4.16E-06 | up |
| GGH | 4670.454338 | -0.563917547 | 0.104643449 | -5.388942688 | 7.09E-08 | 4.17E-06 | down |
| AC008443.4 | 2.531784989 | 1.301619293 | 0.241767856 | 5.38375661 | 7.29E-08 | 4.28E-06 | up |
| AL121885.2 | 2.535440115 | -1.7429294 | 0.324000877 | -5.37939717 | 7.47E-08 | 4.37E-06 | down |
| FRGCA | 6.249147403 | -0.966388742 | 0.17964687 | -5.379379778 | 7.47E-08 | 4.37E-06 | down |
| C6orf58 | 2.999481004 | 0.806147285 | 0.14993225 | 5.376743725 | 7.58E-08 | 4.41E-06 | up |
| FOXC2 | 28.84819851 | -0.842641887 | 0.156867184 | -5.371690025 | 7.80E-08 | 4.53E-06 | down |
| PPP1R3G | 104.8999592 | -0.642675439 | 0.119710783 | -5.36856767 | 7.94E-08 | 4.60E-06 | down |
| SYNE4 | 306.7564102 | -0.846340693 | 0.157690732 | -5.367092184 | 8.00E-08 | 4.62E-06 | down |
| MT1E | 1392.922591 | 0.829514409 | 0.154623971 | 5.364720635 | 8.11E-08 | 4.67E-06 | up |
| INSM1 | 59.64554704 | 1.245432549 | 0.23217006 | 5.364311604 | 8.13E-08 | 4.68E-06 | up |
| PKD1L1 | 56.8519194 | -0.610966798 | 0.11393364 | -5.362479402 | 8.21E-08 | 4.72E-06 | down |
| AC098476.1 | 20.18515496 | 0.9528177 | 0.177773505 | 5.359728383 | 8.33E-08 | 4.78E-06 | up |
| LCK | 526.7187884 | 0.676711836 | 0.12626438 | 5.359483283 | 8.35E-08 | 4.78E-06 | up |
| AC079062.1 | 9.138271505 | -2.175155538 | 0.406133383 | -5.355766431 | 8.52E-08 | 4.87E-06 | down |
| AC021683.1 | 8.198284988 | -1.188538259 | 0.222053166 | -5.352494103 | 8.68E-08 | 4.94E-06 | down |
| ARHGAP27P1-BPTFP1-KPNA2P3 | 132.7017572 | 0.509117487 | 0.095181 | 5.348940295 | 8.85E-08 | 5.03E-06 | up |
| AC106876.1 | 178.9893163 | -0.5352972 | 0.100154017 | -5.344740191 | 9.05E-08 | 5.14E-06 | down |
| SLC4A1 | 2.075895204 | 1.27596772 | 0.238823523 | 5.342722121 | 9.16E-08 | 5.19E-06 | up |
| LINC00330 | 3.097866312 | 1.411471165 | 0.264328594 | 5.339835327 | 9.30E-08 | 5.26E-06 | up |
| TBX21 | 21.75351732 | 0.707689676 | 0.132676674 | 5.333941925 | 9.61E-08 | 5.42E-06 | up |
| CEACAM6 | 44962.52718 | -0.628830864 | 0.11791115 | -5.333090757 | 9.66E-08 | 5.44E-06 | down |
| LRRC74B | 1.631624844 | 1.350558373 | 0.253344095 | 5.330925021 | 9.77E-08 | 5.48E-06 | up |
| SUMO2P17 | 10.04160659 | 0.730566358 | 0.137115532 | 5.328107965 | 9.92E-08 | 5.55E-06 | up |
| PRKAA2 | 69.94402056 | -1.111483584 | 0.208614201 | -5.327938266 | 9.93E-08 | 5.55E-06 | down |
| TAC1 | 84.34101247 | -1.254496481 | 0.235562316 | -5.325539773 | 1.01E-07 | 5.61E-06 | down |
| SERPINA6 | 91.00045076 | -1.141891002 | 0.21443805 | -5.325039099 | 1.01E-07 | 5.62E-06 | down |
| C2orf54 | 429.0625479 | -0.770340569 | 0.144693598 | -5.32394368 | 1.02E-07 | 5.64E-06 | down |
| MUC12 | 5880.251606 | -0.935369272 | 0.175736305 | -5.322572772 | 1.02E-07 | 5.67E-06 | down |
| SCARNA5 | 4.716313165 | -1.140948631 | 0.21451799 | -5.31866177 | 1.05E-07 | 5.79E-06 | down |
| ZNF683 | 45.90645415 | 0.970954214 | 0.182647955 | 5.315987317 | 1.06E-07 | 5.86E-06 | up |
| XKR9 | 101.1411744 | 0.923176668 | 0.174090323 | 5.302860344 | 1.14E-07 | 6.23E-06 | up |
| AC090255.1 | 1.170068885 | 2.03834467 | 0.384397517 | 5.302699891 | 1.14E-07 | 6.23E-06 | up |
| HHIP-AS1 | 19.74825402 | -0.647879982 | 0.122315899 | -5.296776504 | 1.18E-07 | 6.41E-06 | down |
| SAA1 | 445.6193768 | 1.063497238 | 0.200789381 | 5.296581076 | 1.18E-07 | 6.41E-06 | up |
| FRG1JP | 6.990543256 | 0.746526587 | 0.141117299 | 5.2901139 | 1.22E-07 | 6.58E-06 | up |
| AC005392.2 | 25.95704441 | 1.100749682 | 0.208078597 | 5.290066814 | 1.22E-07 | 6.58E-06 | up |
| PRAC2 | 57.66647818 | -0.910829349 | 0.172178511 | -5.290029183 | 1.22E-07 | 6.58E-06 | down |
| DOCK3 | 29.59763405 | 0.654120752 | 0.12368924 | 5.28842083 | 1.23E-07 | 6.62E-06 | up |
| AC026336.3 | 58.97981848 | -1.46432443 | 0.276923965 | -5.287821256 | 1.24E-07 | 6.63E-06 | down |
| TCP11 | 28.13145807 | 1.49789645 | 0.283391646 | 5.28560553 | 1.25E-07 | 6.70E-06 | up |
| AC079228.1 | 2.195491703 | 0.919475106 | 0.174089457 | 5.281624284 | 1.28E-07 | 6.84E-06 | up |
| EPHA3 | 109.1517163 | -0.646890189 | 0.12257481 | -5.277513291 | 1.31E-07 | 6.96E-06 | down |
| AC112484.2 | 11.28512274 | 0.549962066 | 0.104258017 | 5.275009835 | 1.33E-07 | 7.04E-06 | up |
| AL121757.1 | 7.098893932 | -1.19945375 | 0.227711831 | -5.267419545 | 1.38E-07 | 7.29E-06 | down |
| USP30-AS1 | 45.59486311 | 0.615042864 | 0.116767683 | 5.267235315 | 1.38E-07 | 7.29E-06 | up |
| AL645608.7 | 1.910731814 | 1.29028228 | 0.24530121 | 5.2599915 | 1.44E-07 | 7.56E-06 | up |
| CCER2 | 8.520059327 | 0.77894864 | 0.14811131 | 5.259211076 | 1.45E-07 | 7.58E-06 | up |
| AC027449.1 | 1.775074311 | 0.852602395 | 0.162141037 | 5.258399784 | 1.45E-07 | 7.59E-06 | up |
| MAPK12 | 209.1082335 | 0.629139864 | 0.119698517 | 5.256037264 | 1.47E-07 | 7.68E-06 | up |
| IKZF3 | 311.7774085 | 0.593872941 | 0.113102243 | 5.250761815 | 1.51E-07 | 7.89E-06 | up |
| UCKL1-AS1 | 49.74724848 | -0.624187626 | 0.118910043 | -5.249242302 | 1.53E-07 | 7.94E-06 | down |
| CYP1A1 | 10.69724368 | 1.70933867 | 0.326116394 | 5.241498739 | 1.59E-07 | 8.23E-06 | up |
| TRDC | 31.91816705 | 0.884607965 | 0.168953219 | 5.235815986 | 1.64E-07 | 8.45E-06 | up |
| HMSD | 11.93224886 | 0.969281733 | 0.185221365 | 5.233098966 | 1.67E-07 | 8.56E-06 | up |
| SNAI3 | 40.20224618 | 0.547623963 | 0.104658926 | 5.232463039 | 1.67E-07 | 8.58E-06 | up |
| BPI | 8.092322969 | -0.744140229 | 0.14233527 | -5.228080357 | 1.71E-07 | 8.77E-06 | down |
| RPS21 | 32608.06199 | -0.553219579 | 0.105836256 | -5.227127248 | 1.72E-07 | 8.80E-06 | down |
| ADAM11 | 14.90836648 | 0.713385246 | 0.136497625 | 5.226356467 | 1.73E-07 | 8.83E-06 | up |
| UBE2V1P2 | 7.801989148 | -0.721199993 | 0.138083373 | -5.222931458 | 1.76E-07 | 8.96E-06 | down |
| SPRR2D | 26.60417727 | 1.571669244 | 0.300995223 | 5.221575369 | 1.77E-07 | 9.01E-06 | up |
| FOXD2 | 445.6507066 | -0.515345216 | 0.098699591 | -5.221351104 | 1.78E-07 | 9.01E-06 | down |
| TYMP | 2247.534892 | 0.619551487 | 0.118705949 | 5.219211795 | 1.80E-07 | 9.09E-06 | up |
| ERP27 | 229.916425 | -0.881185572 | 0.168882656 | -5.217738724 | 1.81E-07 | 9.15E-06 | down |
| AL513497.1 | 11.63487003 | 0.583679728 | 0.111892551 | 5.2164306 | 1.82E-07 | 9.20E-06 | up |
| ASPN | 604.1546614 | -0.827548767 | 0.158667967 | -5.215600754 | 1.83E-07 | 9.22E-06 | down |
| AL606834.2 | 13.30078776 | 0.520793215 | 0.099918721 | 5.21216853 | 1.87E-07 | 9.37E-06 | up |
| GDA | 1100.753515 | 0.561287496 | 0.107851011 | 5.204285903 | 1.95E-07 | 9.72E-06 | up |
| RAB27B | 507.4745902 | 0.75322642 | 0.144803658 | 5.201708513 | 1.97E-07 | 9.84E-06 | up |
| DUSP4 | 1906.703335 | 0.841996657 | 0.162053952 | 5.195779832 | 2.04E-07 | 1.01E-05 | up |
| SYN3 | 99.7090065 | -1.063819164 | 0.204757689 | -5.195502888 | 2.04E-07 | 1.01E-05 | down |
| AC131212.3 | 73.43621148 | 0.525187762 | 0.101193194 | 5.189951419 | 2.10E-07 | 1.04E-05 | up |
| PDE6A | 33.82571286 | -0.846357325 | 0.163261749 | -5.184051555 | 2.17E-07 | 1.07E-05 | down |
| APCDD1 | 3460.095386 | -0.867546024 | 0.167405345 | -5.182307806 | 2.19E-07 | 1.07E-05 | down |
| HOGA1 | 24.63319548 | -0.835192745 | 0.161315043 | -5.177401496 | 2.25E-07 | 1.10E-05 | down |
| CD8A | 245.8248731 | 0.666199161 | 0.128715727 | 5.17574021 | 2.27E-07 | 1.11E-05 | up |
| GIPR | 85.47854315 | 0.658455441 | 0.127221632 | 5.175656299 | 2.27E-07 | 1.11E-05 | up |
| ZNF542P | 61.56441879 | -0.572340435 | 0.110704236 | -5.169995813 | 2.34E-07 | 1.14E-05 | down |
| AC138811.2 | 3.772957067 | 0.740577509 | 0.143255364 | 5.169631964 | 2.35E-07 | 1.14E-05 | up |
| CALML5 | 2.228152635 | 2.03090172 | 0.392923468 | 5.168695397 | 2.36E-07 | 1.14E-05 | up |
| AC090772.3 | 3.756805505 | 0.620537693 | 0.12006569 | 5.168318222 | 2.36E-07 | 1.14E-05 | up |
| ZNF578 | 4.785506662 | 0.737003285 | 0.143043424 | 5.152304538 | 2.57E-07 | 1.23E-05 | up |
| KRT16 | 71.84147815 | 1.135896215 | 0.220499677 | 5.151464306 | 2.58E-07 | 1.23E-05 | up |
| OASL | 525.6827191 | 0.569896325 | 0.110629129 | 5.151412924 | 2.59E-07 | 1.23E-05 | up |
| SAMD9L | 626.3764182 | 0.567468995 | 0.110261986 | 5.146551551 | 2.65E-07 | 1.25E-05 | up |
| RNU4-1 | 17.06281274 | -1.486030509 | 0.288913304 | -5.143517061 | 2.70E-07 | 1.27E-05 | down |
| VNN2 | 98.79723817 | 0.861885152 | 0.167569361 | 5.143453109 | 2.70E-07 | 1.27E-05 | up |
| AL050331.1 | 11.79308893 | -0.685252191 | 0.133419405 | -5.136075914 | 2.81E-07 | 1.31E-05 | down |
| TENM2 | 15.89201956 | -0.895358377 | 0.174327958 | -5.136057264 | 2.81E-07 | 1.31E-05 | down |
| AC116351.1 | 22.16587651 | -0.651586606 | 0.126896355 | -5.134793728 | 2.82E-07 | 1.32E-05 | down |
| FAM189A2 | 33.78818248 | 0.743829829 | 0.144899404 | 5.133422291 | 2.85E-07 | 1.33E-05 | up |
| RNLS | 223.6757718 | -0.600535315 | 0.117013535 | -5.132186763 | 2.86E-07 | 1.33E-05 | down |
| LINC01630 | 5.983489407 | 1.66308904 | 0.324124064 | 5.131026128 | 2.88E-07 | 1.34E-05 | up |
| AP003774.4 | 294.8281181 | -0.902364356 | 0.176007642 | -5.126847602 | 2.95E-07 | 1.36E-05 | down |
| TMEM72 | 37.46461947 | -0.8798168 | 0.171706336 | -5.123962333 | 2.99E-07 | 1.38E-05 | down |
| AL662899.2 | 324.4734199 | -1.263009006 | 0.246610912 | -5.121464398 | 3.03E-07 | 1.40E-05 | down |
| CITED1 | 60.31605481 | 0.807262712 | 0.157697627 | 5.119054268 | 3.07E-07 | 1.41E-05 | up |
| SLC34A3 | 19.71522177 | 0.81570375 | 0.159384962 | 5.117821288 | 3.09E-07 | 1.42E-05 | up |
| AC005911.1 | 7.707533798 | 0.573653961 | 0.112161229 | 5.114547747 | 3.14E-07 | 1.44E-05 | up |
| MTUS2 | 12.50956936 | 0.818724729 | 0.160175423 | 5.111425414 | 3.20E-07 | 1.46E-05 | up |
| LINC01750 | 8.953088473 | 0.847305604 | 0.165783441 | 5.110918182 | 3.21E-07 | 1.46E-05 | up |
| RUBCNL | 3139.838538 | -0.690314274 | 0.135096421 | -5.109789504 | 3.23E-07 | 1.47E-05 | down |
| RAMP1 | 634.2343571 | 0.901682385 | 0.176540061 | 5.10752279 | 3.26E-07 | 1.49E-05 | up |
| AL022316.1 | 17.50442829 | 0.919050879 | 0.180003856 | 5.105728831 | 3.30E-07 | 1.50E-05 | up |
| ATP2A1 | 61.13431035 | 0.559337599 | 0.109551584 | 5.105700717 | 3.30E-07 | 1.50E-05 | up |
| KIF19 | 87.62892423 | 0.965454688 | 0.189119912 | 5.104986967 | 3.31E-07 | 1.50E-05 | up |
| CALCB | 13.70039662 | 1.464580193 | 0.287110649 | 5.101100206 | 3.38E-07 | 1.53E-05 | up |
| RN7SKP255 | 1.436265003 | -3.23747223 | 0.635049135 | -5.097986996 | 3.43E-07 | 1.55E-05 | down |
| AC069200.1 | 1.933094579 | 0.815270609 | 0.15997433 | 5.096258945 | 3.46E-07 | 1.56E-05 | up |
| AL121829.2 | 6.07522358 | -0.782354785 | 0.153551787 | -5.095054913 | 3.49E-07 | 1.57E-05 | down |
| RSPH14 | 21.12283081 | -0.610436801 | 0.119885983 | -5.09181128 | 3.55E-07 | 1.59E-05 | down |
| APOL3 | 668.4750948 | 0.540953821 | 0.106312042 | 5.088358847 | 3.61E-07 | 1.62E-05 | up |
| DDX3P1 | 4.888056032 | -0.86731965 | 0.170532997 | -5.085934477 | 3.66E-07 | 1.64E-05 | down |
| AC090181.2 | 19.25783742 | 0.534175416 | 0.10508717 | 5.083164941 | 3.71E-07 | 1.66E-05 | up |
| AC108865.2 | 24.63031492 | -1.801851918 | 0.354515139 | -5.082581027 | 3.72E-07 | 1.66E-05 | down |
| TNFRSF11A | 1312.285044 | 0.505981171 | 0.099580191 | 5.081142817 | 3.75E-07 | 1.67E-05 | up |
| EREG | 1853.746862 | -0.913209703 | 0.179862794 | -5.077257414 | 3.83E-07 | 1.70E-05 | down |
| CA5A | 3.23164181 | -0.858667963 | 0.169324613 | -5.071134949 | 3.95E-07 | 1.75E-05 | down |
| SH2D1B | 12.85655006 | 0.692523759 | 0.136590044 | 5.070089592 | 3.98E-07 | 1.76E-05 | up |
| ZFP92 | 31.23038533 | -0.646453349 | 0.127647976 | -5.064344703 | 4.10E-07 | 1.81E-05 | down |
| RASL10B | 104.0270773 | -0.802157956 | 0.158619059 | -5.057134747 | 4.26E-07 | 1.87E-05 | down |
| SNORA74B | 1.926087627 | -1.586064665 | 0.313659165 | -5.056650151 | 4.27E-07 | 1.87E-05 | down |
| CD74 | 39913.95373 | 0.568308101 | 0.112625409 | 5.046002535 | 4.51E-07 | 1.97E-05 | up |
| BX640514.2 | 6.196383227 | 0.846881314 | 0.167955797 | 5.042286891 | 4.60E-07 | 2.00E-05 | up |
| AC005498.2 | 1.524890199 | -0.886281649 | 0.175783114 | -5.041904357 | 4.61E-07 | 2.00E-05 | down |
| SPINK6 | 2.201011809 | -1.289440856 | 0.255759243 | -5.041619767 | 4.62E-07 | 2.00E-05 | down |
| COLGALT2 | 84.88963072 | 1.012123343 | 0.200817138 | 5.040024738 | 4.65E-07 | 2.01E-05 | up |
| AL596442.2 | 4.247838968 | 0.762309969 | 0.151278237 | 5.03912514 | 4.68E-07 | 2.02E-05 | up |
| CREB3L1 | 4217.025853 | 0.64403981 | 0.127909569 | 5.035118294 | 4.78E-07 | 2.06E-05 | up |
| AC011815.2 | 3.999336956 | 0.642508035 | 0.127680688 | 5.032147336 | 4.85E-07 | 2.08E-05 | up |
| CHRNA1 | 33.92393293 | -0.630663884 | 0.125372085 | -5.030337358 | 4.90E-07 | 2.10E-05 | down |
| ZNF528-AS1 | 40.62308036 | -0.645882178 | 0.12843297 | -5.028943715 | 4.93E-07 | 2.11E-05 | down |
| MCMDC2 | 72.58062587 | -0.602034979 | 0.119725572 | -5.028457757 | 4.94E-07 | 2.11E-05 | down |
| FGF8 | 2.835125009 | 0.859641048 | 0.170957328 | 5.028395427 | 4.95E-07 | 2.11E-05 | up |
| CD68 | 35.60238078 | 0.62901315 | 0.125213285 | 5.023533663 | 5.07E-07 | 2.15E-05 | up |
| BCYRN1 | 123.6074445 | -0.564696627 | 0.112432484 | -5.022539799 | 5.10E-07 | 2.16E-05 | down |
| ALOXE3 | 11.43115521 | 0.89855426 | 0.178942596 | 5.021466541 | 5.13E-07 | 2.17E-05 | up |
| AC133065.6 | 71.90990771 | 0.504937109 | 0.100565337 | 5.020985582 | 5.14E-07 | 2.17E-05 | up |
| BCAS4 | 335.0080346 | -0.519549754 | 0.103476806 | -5.020929579 | 5.14E-07 | 2.17E-05 | down |
| CXCL13 | 215.0977844 | 0.90188867 | 0.179645068 | 5.020392036 | 5.16E-07 | 2.17E-05 | up |
| PRSS51 | 29.28021861 | -1.028530377 | 0.204909624 | -5.01943422 | 5.18E-07 | 2.18E-05 | down |
| ETV5 | 640.4282776 | 0.53626259 | 0.106854141 | 5.018641144 | 5.20E-07 | 2.19E-05 | up |
| VAV3 | 2933.055139 | -0.641727776 | 0.127876898 | -5.018324558 | 5.21E-07 | 2.19E-05 | down |
| PCDHGC3 | 271.7073002 | -0.609739051 | 0.121553457 | -5.016221389 | 5.27E-07 | 2.20E-05 | down |
| AIM2 | 97.26306241 | 0.897576669 | 0.178972484 | 5.01516572 | 5.30E-07 | 2.21E-05 | up |
| CTSW | 99.29184114 | 0.704903192 | 0.14060714 | 5.013281616 | 5.35E-07 | 2.23E-05 | up |
| RPL23AP65 | 47.82695189 | -0.788817358 | 0.157375778 | -5.012317441 | 5.38E-07 | 2.24E-05 | down |
| AC239803.3 | 2.930780782 | 0.756022838 | 0.150842904 | 5.011988074 | 5.39E-07 | 2.24E-05 | up |
| AP002004.1 | 73.47661336 | 0.602157379 | 0.120240431 | 5.007944269 | 5.50E-07 | 2.28E-05 | up |
| ZIC2 | 239.0808089 | 1.203784188 | 0.240419864 | 5.007008026 | 5.53E-07 | 2.29E-05 | up |
| PSPC1P1 | 4.594575375 | 0.673280328 | 0.134469836 | 5.006924586 | 5.53E-07 | 2.29E-05 | up |
| MT2A | 2138.927362 | 0.588269798 | 0.117532675 | 5.005159627 | 5.58E-07 | 2.30E-05 | up |
| AC106869.1 | 30.7784186 | -0.517531964 | 0.103409949 | -5.004663187 | 5.60E-07 | 2.30E-05 | down |
| NAT8 | 10.53839954 | 1.340002729 | 0.267781447 | 5.004091008 | 5.61E-07 | 2.31E-05 | up |
| ST6GAL1 | 4677.119924 | -0.511942816 | 0.102325065 | -5.003102752 | 5.64E-07 | 2.32E-05 | down |
| ALDH1A2 | 72.71107806 | -1.046250998 | 0.209135534 | -5.00274142 | 5.65E-07 | 2.32E-05 | down |
| AP005136.2 | 7.662107648 | 0.532980662 | 0.106557077 | 5.001832626 | 5.68E-07 | 2.33E-05 | up |
| COL9A2 | 649.4220418 | 0.661070791 | 0.132186003 | 5.001064967 | 5.70E-07 | 2.33E-05 | up |
| DDIT4L | 36.48136753 | -0.990507534 | 0.198100036 | -5.000037122 | 5.73E-07 | 2.34E-05 | down |
| CA3-AS1 | 9.198836341 | 0.710063304 | 0.14201661 | 4.999860956 | 5.74E-07 | 2.34E-05 | up |
| WASF3 | 161.2279229 | -0.823451578 | 0.164760343 | -4.997874888 | 5.80E-07 | 2.36E-05 | down |
| ZNF285 | 39.90300163 | -0.860819367 | 0.172350775 | -4.994577871 | 5.90E-07 | 2.39E-05 | down |
| XXYLT1-AS2 | 6.005934707 | 0.968841139 | 0.194005974 | 4.993872717 | 5.92E-07 | 2.40E-05 | up |
| ZNF582 | 20.61688108 | -0.564921 | 0.113220512 | -4.989564059 | 6.05E-07 | 2.44E-05 | down |
| HRASLS2 | 43.68034573 | 0.759360289 | 0.15221363 | 4.988779835 | 6.08E-07 | 2.45E-05 | up |
| TKTL1 | 9.549170419 | 1.367800854 | 0.274289277 | 4.98670918 | 6.14E-07 | 2.47E-05 | up |
| AC007277.1 | 41.79409749 | 1.106334385 | 0.221941877 | 4.984793305 | 6.20E-07 | 2.50E-05 | up |
| RBP4 | 570.2417604 | -0.861849647 | 0.172969384 | -4.982671656 | 6.27E-07 | 2.52E-05 | down |
| ARHGAP4 | 2091.510308 | 0.640974962 | 0.128646039 | 4.982469484 | 6.28E-07 | 2.52E-05 | up |
| SHROOM4 | 871.4608903 | -0.501041008 | 0.100622079 | -4.979434077 | 6.38E-07 | 2.55E-05 | down |
| FER1L4 | 668.7508612 | -0.72076633 | 0.144834214 | -4.976492155 | 6.47E-07 | 2.59E-05 | down |
| TPPP3 | 731.2406639 | -0.620582773 | 0.124707982 | -4.976287491 | 6.48E-07 | 2.59E-05 | down |
| AC016582.3 | 6.134877627 | -0.879145776 | 0.176684171 | -4.975803843 | 6.50E-07 | 2.59E-05 | down |
| SERPINA7 | 34.43500204 | -1.370686441 | 0.275550148 | -4.974362929 | 6.55E-07 | 2.61E-05 | down |
| LINC02308 | 0.927736581 | -1.240109135 | 0.249420514 | -4.971961273 | 6.63E-07 | 2.63E-05 | down |
| ANXA10 | 30.89310566 | 1.510818212 | 0.303946995 | 4.970663434 | 6.67E-07 | 2.64E-05 | up |
| CD7 | 253.5439468 | 0.649496144 | 0.130766474 | 4.966839924 | 6.81E-07 | 2.69E-05 | up |
| ADSSL1 | 82.23958935 | 0.620884331 | 0.125022221 | 4.966191807 | 6.83E-07 | 2.69E-05 | up |
| APOL4 | 304.8628426 | 0.558860331 | 0.112560852 | 4.964961797 | 6.87E-07 | 2.71E-05 | up |
| TMEM176A | 7296.645732 | -0.541382555 | 0.109091527 | -4.962645288 | 6.95E-07 | 2.74E-05 | down |
| CRTAM | 20.68424791 | 0.74717401 | 0.150783441 | 4.955278935 | 7.22E-07 | 2.82E-05 | up |
| DMBX1 | 29.62133729 | 0.938642046 | 0.189424565 | 4.955228741 | 7.22E-07 | 2.82E-05 | up |
| CXCL14 | 5632.366614 | -0.769183268 | 0.155276107 | -4.953648589 | 7.28E-07 | 2.84E-05 | down |
| OSR2 | 183.7869848 | 0.606338465 | 0.122526859 | 4.948616744 | 7.47E-07 | 2.91E-05 | up |
| HOXD4 | 9.132653808 | 0.71107577 | 0.143719806 | 4.947653294 | 7.51E-07 | 2.92E-05 | up |
| VENTX | 139.9336114 | -1.047093532 | 0.211690032 | -4.946352553 | 7.56E-07 | 2.93E-05 | down |
| CLIC6 | 171.0626312 | 0.724934913 | 0.146583615 | 4.945538501 | 7.59E-07 | 2.94E-05 | up |
| MLPH | 2203.757544 | 0.665436541 | 0.134561487 | 4.945222842 | 7.61E-07 | 2.94E-05 | up |
| RNVU1-19 | 1.039436455 | -1.353083264 | 0.273804729 | -4.941781931 | 7.74E-07 | 2.99E-05 | down |
| OFCC1 | 4.883732957 | 1.324060172 | 0.268102593 | 4.938632476 | 7.87E-07 | 3.03E-05 | up |
| ZNF571-AS1 | 10.38278151 | -0.647438685 | 0.131170832 | -4.935843408 | 7.98E-07 | 3.07E-05 | down |
| AC058791.1 | 41.90634904 | -0.788506357 | 0.159869226 | -4.932196004 | 8.13E-07 | 3.12E-05 | down |
| NPW | 61.396247 | -0.867054444 | 0.175902464 | -4.929177365 | 8.26E-07 | 3.16E-05 | down |
| EYA1 | 59.852179 | -1.076251849 | 0.218431228 | -4.92718857 | 8.34E-07 | 3.19E-05 | down |
| KIR2DL1 | 1.217221237 | 1.495806475 | 0.303848809 | 4.922864366 | 8.53E-07 | 3.26E-05 | up |
| C6orf15 | 102.4071551 | -1.32637226 | 0.269497675 | -4.92164639 | 8.58E-07 | 3.27E-05 | down |
| BX322639.1 | 11.14869642 | -0.996308511 | 0.202595788 | -4.917715809 | 8.76E-07 | 3.33E-05 | down |
| GOLGA6L2 | 5.007016226 | -1.535501484 | 0.31228374 | -4.917007467 | 8.79E-07 | 3.34E-05 | down |
| AP000864.1 | 1.319698607 | 1.079007861 | 0.219453367 | 4.916797932 | 8.80E-07 | 3.34E-05 | up |
| CLCN1 | 25.02675196 | 0.750104628 | 0.15258559 | 4.915959823 | 8.83E-07 | 3.34E-05 | up |
| AC114296.1 | 7.234841779 | -0.893739063 | 0.181808802 | -4.915818446 | 8.84E-07 | 3.34E-05 | down |
| TRNP1 | 338.3153183 | 0.786050618 | 0.160027792 | 4.911963138 | 9.02E-07 | 3.40E-05 | up |
| SPIB | 83.37756423 | 0.821832906 | 0.167346877 | 4.91095455 | 9.06E-07 | 3.42E-05 | up |
| SMO | 355.5764029 | -0.530227688 | 0.108029151 | -4.908190839 | 9.19E-07 | 3.46E-05 | down |
| ASGR1 | 160.6043269 | -0.663168372 | 0.135144646 | -4.907100581 | 9.24E-07 | 3.47E-05 | down |
| PHACTR3 | 98.50212911 | -0.864129308 | 0.176135646 | -4.906044445 | 9.29E-07 | 3.48E-05 | down |
| QPRT | 2731.154954 | -0.703242846 | 0.143391546 | -4.904353614 | 9.37E-07 | 3.51E-05 | down |
| KCNN2 | 30.70360391 | 0.634459899 | 0.129410591 | 4.902689157 | 9.45E-07 | 3.54E-05 | up |
| ERN2 | 2750.360508 | 0.517270435 | 0.105558774 | 4.900307342 | 9.57E-07 | 3.57E-05 | up |
| IGF2 | 20064.32284 | -1.318314962 | 0.269227459 | -4.896658631 | 9.75E-07 | 3.63E-05 | down |
| TTC24 | 5.14755664 | 0.897662732 | 0.183345805 | 4.896009127 | 9.78E-07 | 3.63E-05 | up |
| RNF152 | 194.1390301 | 0.504591067 | 0.103120216 | 4.893231292 | 9.92E-07 | 3.67E-05 | up |
| IQGAP2 | 1448.223472 | 0.501328369 | 0.102500056 | 4.891005781 | 1.00E-06 | 3.71E-05 | up |
| LINC01443 | 22.92006442 | 1.088547319 | 0.222603696 | 4.890068483 | 1.01E-06 | 3.72E-05 | up |
| FOXL2NB | 2.252931413 | 1.503736559 | 0.30774586 | 4.886293384 | 1.03E-06 | 3.78E-05 | up |
| RADIL | 24.42971151 | 0.738326633 | 0.151303259 | 4.87978009 | 1.06E-06 | 3.90E-05 | up |
| CLDN9 | 71.77395247 | -0.786142875 | 0.161137618 | -4.878704833 | 1.07E-06 | 3.92E-05 | down |
| MTATP8P2 | 11.31945573 | 0.903411338 | 0.185178446 | 4.878598762 | 1.07E-06 | 3.92E-05 | up |
| LY6G6D | 324.3840324 | -1.227254134 | 0.251735101 | -4.875180811 | 1.09E-06 | 3.97E-05 | down |
| RPL17P36 | 17.78507833 | 0.502591501 | 0.103124977 | 4.873615637 | 1.10E-06 | 4.00E-05 | up |
| NKX3-1 | 46.32562527 | 0.705556237 | 0.144860792 | 4.870581101 | 1.11E-06 | 4.05E-05 | up |
| ADGRD2 | 5.10764406 | 1.09700188 | 0.225284324 | 4.869410626 | 1.12E-06 | 4.07E-05 | up |
| FBXW10 | 1.71202379 | 1.168491962 | 0.240139715 | 4.865883853 | 1.14E-06 | 4.13E-05 | up |
| STARD13-AS | 7.134616836 | -0.691941629 | 0.142416471 | -4.858578684 | 1.18E-06 | 4.27E-05 | down |
| SULT1C2 | 437.3268822 | 0.720639283 | 0.148389114 | 4.856416092 | 1.20E-06 | 4.31E-05 | up |
| MUC1 | 6059.416595 | 0.660524109 | 0.136060404 | 4.854638727 | 1.21E-06 | 4.34E-05 | up |
| ABCA12 | 77.50697888 | 1.156017684 | 0.238200002 | 4.853138849 | 1.22E-06 | 4.36E-05 | up |
| PLCXD3 | 9.734778755 | 1.256890166 | 0.259099682 | 4.850990775 | 1.23E-06 | 4.40E-05 | up |
| RIMBP2 | 100.5161811 | 0.868949317 | 0.179132071 | 4.850886333 | 1.23E-06 | 4.40E-05 | up |
| UGT2A3 | 421.433274 | -1.03391522 | 0.213269021 | -4.847939083 | 1.25E-06 | 4.45E-05 | down |
| GCM1 | 4.556528569 | -1.077516014 | 0.222340751 | -4.846237182 | 1.26E-06 | 4.48E-05 | down |
| AF124730.1 | 1.822537349 | 0.976513122 | 0.201610714 | 4.843557674 | 1.28E-06 | 4.54E-05 | up |
| AL355864.2 | 2.594129368 | 0.783317814 | 0.161765083 | 4.84231701 | 1.28E-06 | 4.56E-05 | up |
| MIAT | 193.4151259 | 0.642827639 | 0.132849213 | 4.838776432 | 1.31E-06 | 4.63E-05 | up |
| L3MBTL1 | 250.6137205 | -0.570494262 | 0.11794506 | -4.836949209 | 1.32E-06 | 4.66E-05 | down |
| AREG | 3194.678445 | -0.627688315 | 0.129809107 | -4.835472118 | 1.33E-06 | 4.69E-05 | down |
| OR5BA1P | 6.672359992 | 0.800727731 | 0.165625102 | 4.834579542 | 1.33E-06 | 4.70E-05 | up |
| HIST1H1B | 12.68123657 | -0.901947346 | 0.186642372 | -4.832489737 | 1.35E-06 | 4.75E-05 | down |
| AL162411.1 | 5.773505991 | -1.356861178 | 0.28081757 | -4.831824362 | 1.35E-06 | 4.76E-05 | down |
| KL | 36.66353352 | -0.658927346 | 0.136490574 | -4.827639936 | 1.38E-06 | 4.84E-05 | down |
| SNORA14A | 0.903565459 | -1.640084269 | 0.339942976 | -4.824586436 | 1.40E-06 | 4.90E-05 | down |
| AL135924.2 | 2.477185887 | 1.075591096 | 0.222983457 | 4.823636299 | 1.41E-06 | 4.92E-05 | up |
| MUC5B | 11994.45916 | 0.998323625 | 0.207277217 | 4.816369295 | 1.46E-06 | 5.08E-05 | up |
| SDR16C5 | 572.3328962 | 0.850086034 | 0.176561476 | 4.814674493 | 1.47E-06 | 5.12E-05 | up |
| TCN1 | 964.1843006 | 1.191780548 | 0.247589014 | 4.813543738 | 1.48E-06 | 5.14E-05 | up |
| LINC00513 | 91.15651588 | -0.613737201 | 0.127535729 | -4.812276572 | 1.49E-06 | 5.17E-05 | down |
| AC124248.1 | 24.54788821 | -0.538825874 | 0.112030736 | -4.809625417 | 1.51E-06 | 5.23E-05 | down |
| HGFAC | 4.371539385 | 0.936183459 | 0.194747292 | 4.807170616 | 1.53E-06 | 5.29E-05 | up |
| LY6G6E | 10.24670303 | -0.911465431 | 0.189634058 | -4.80644373 | 1.54E-06 | 5.31E-05 | down |
| OGDHL | 100.9188656 | -0.975647066 | 0.203022567 | -4.805608946 | 1.54E-06 | 5.32E-05 | down |
| KIR2DS4 | 2.050859816 | 1.376232935 | 0.286389259 | 4.805462815 | 1.54E-06 | 5.32E-05 | up |
| TPO | 21.27679175 | 0.772317025 | 0.160727999 | 4.805118148 | 1.55E-06 | 5.32E-05 | up |
| TG | 359.0863835 | -0.759171682 | 0.158011181 | -4.804544057 | 1.55E-06 | 5.33E-05 | down |
| DKFZP434K028 | 7.955918701 | 0.890624981 | 0.185404919 | 4.803675041 | 1.56E-06 | 5.35E-05 | up |
| KRTAP13-2 | 1.145033564 | -2.031369158 | 0.423113887 | -4.800998556 | 1.58E-06 | 5.41E-05 | down |
| FAM83A | 73.46293061 | 0.885574457 | 0.184502588 | 4.79979423 | 1.59E-06 | 5.44E-05 | up |
| SPRR3 | 20.6002759 | 1.641111279 | 0.341934269 | 4.799493429 | 1.59E-06 | 5.44E-05 | up |
| IGSF23 | 26.01657725 | -0.790222886 | 0.164654117 | -4.799290181 | 1.59E-06 | 5.44E-05 | down |
| AC010970.1 | 16.1603343 | -1.329367456 | 0.277026095 | -4.798708433 | 1.60E-06 | 5.45E-05 | down |
| AC009336.1 | 2.175064994 | 0.89202118 | 0.185943738 | 4.797263898 | 1.61E-06 | 5.48E-05 | up |
| TMEM269 | 2.297262797 | 0.675966197 | 0.140936736 | 4.796238483 | 1.62E-06 | 5.51E-05 | up |
| SLC22A31 | 29.42566083 | -1.025701577 | 0.21388843 | -4.795498186 | 1.62E-06 | 5.52E-05 | down |
| THBS4 | 333.2712669 | -1.013122356 | 0.211323157 | -4.794185215 | 1.63E-06 | 5.54E-05 | down |
| SLITRK6 | 377.5919621 | 1.13274284 | 0.236291447 | 4.793837667 | 1.64E-06 | 5.54E-05 | up |
| DPP4 | 1430.957363 | 0.657882521 | 0.137316001 | 4.791011348 | 1.66E-06 | 5.62E-05 | up |
| OAS2 | 1478.338187 | 0.616597178 | 0.128937371 | 4.782144804 | 1.73E-06 | 5.83E-05 | up |
| TOGARAM2 | 16.00667583 | 0.525164613 | 0.109924776 | 4.777490876 | 1.77E-06 | 5.94E-05 | up |
| AC007790.1 | 2.592513361 | -1.169635335 | 0.244837655 | -4.777187297 | 1.78E-06 | 5.94E-05 | down |
| HBA2 | 127.4730971 | 0.751394183 | 0.157318104 | 4.776272802 | 1.79E-06 | 5.96E-05 | up |
| CBR3 | 150.6671383 | 0.502526258 | 0.105269248 | 4.773723268 | 1.81E-06 | 6.02E-05 | up |
| PTCHD3P2 | 9.059149694 | -1.192395711 | 0.24981594 | -4.773096996 | 1.81E-06 | 6.03E-05 | down |
| JAKMIP3 | 12.60047324 | 0.5979453 | 0.125304098 | 4.771953278 | 1.82E-06 | 6.05E-05 | up |
| AC010980.1 | 5.910145956 | 0.753126826 | 0.158041678 | 4.765368453 | 1.89E-06 | 6.22E-05 | up |
| SCNN1B | 232.8333541 | -0.905453952 | 0.190082834 | -4.763470395 | 1.90E-06 | 6.26E-05 | down |
| AL590764.1 | 17.07597688 | -0.815781 | 0.171303631 | -4.762193273 | 1.92E-06 | 6.30E-05 | down |
| REG1A | 15213.11627 | 1.338673092 | 0.281240451 | 4.759888164 | 1.94E-06 | 6.36E-05 | up |
| DRD1 | 14.81388445 | -0.839838706 | 0.176452242 | -4.759580827 | 1.94E-06 | 6.36E-05 | down |
| WFDC3 | 81.02879704 | -0.65633496 | 0.137905771 | -4.75930016 | 1.94E-06 | 6.36E-05 | down |
| RPS15AP17 | 17.37601537 | -0.890521433 | 0.187229041 | -4.756321077 | 1.97E-06 | 6.44E-05 | down |
| UGT2B4 | 5.632088954 | 1.592996986 | 0.334971967 | 4.755612837 | 1.98E-06 | 6.46E-05 | up |
| HOXC-AS1 | 1.902744115 | 1.347796926 | 0.283433926 | 4.755242059 | 1.98E-06 | 6.47E-05 | up |
| AC007663.2 | 2.261215401 | 1.087614194 | 0.228929724 | 4.750864908 | 2.03E-06 | 6.59E-05 | up |
| PSD2 | 11.79898443 | 0.723945115 | 0.152443646 | 4.748935983 | 2.04E-06 | 6.64E-05 | up |
| RN7SL834P | 8.918831695 | 0.51846331 | 0.109198654 | 4.747891028 | 2.06E-06 | 6.66E-05 | up |
| CTSE | 2409.894462 | 1.16496274 | 0.245549084 | 4.744317195 | 2.09E-06 | 6.74E-05 | up |
| PSAPL1 | 4.364779083 | 1.667338759 | 0.351448942 | 4.744184889 | 2.09E-06 | 6.74E-05 | up |
| MUM1L1 | 13.98306535 | -1.163318152 | 0.245443534 | -4.739656951 | 2.14E-06 | 6.87E-05 | down |
| ZIC5 | 102.758086 | 1.349115501 | 0.284651294 | 4.739537572 | 2.14E-06 | 6.87E-05 | up |
| HOXB3 | 1302.890044 | 0.501181509 | 0.10575134 | 4.739244977 | 2.15E-06 | 6.87E-05 | up |
| PRRT4 | 8.969635161 | 0.963122451 | 0.203297752 | 4.737496797 | 2.16E-06 | 6.92E-05 | up |
| TRDV1 | 4.575601161 | 1.131448943 | 0.239050458 | 4.733096741 | 2.21E-06 | 7.02E-05 | up |
| AC020656.2 | 89.44502026 | 0.818338761 | 0.17301233 | 4.729944756 | 2.25E-06 | 7.13E-05 | up |
| AC019117.1 | 6.432957839 | 0.944525723 | 0.199708021 | 4.729533245 | 2.25E-06 | 7.13E-05 | up |
| CKMT2 | 557.8171301 | -1.031649359 | 0.218172684 | -4.72859086 | 2.26E-06 | 7.16E-05 | down |
| KCNF1 | 21.94574555 | -0.87319562 | 0.184727929 | -4.72692801 | 2.28E-06 | 7.20E-05 | down |
| AC087477.2 | 9.349826839 | 0.522164394 | 0.110484704 | 4.726123852 | 2.29E-06 | 7.23E-05 | up |
| SPIN3 | 122.1440758 | -0.548268123 | 0.116062121 | -4.723919549 | 2.31E-06 | 7.28E-05 | down |
| ZFP82 | 46.54265404 | -0.567515001 | 0.12029862 | -4.717552059 | 2.39E-06 | 7.48E-05 | down |
| AC009336.2 | 4.288336235 | 0.765050768 | 0.16231745 | 4.713299578 | 2.44E-06 | 7.62E-05 | up |
| AL645608.3 | 7.074544713 | 1.016583249 | 0.21568405 | 4.713298223 | 2.44E-06 | 7.62E-05 | up |
| AL365475.1 | 4.142383636 | 1.031977081 | 0.219226744 | 4.707350308 | 2.51E-06 | 7.82E-05 | up |
| C4BPB | 333.8043389 | 0.580394856 | 0.123296794 | 4.707298836 | 2.51E-06 | 7.82E-05 | up |
| ROBO3 | 111.0141851 | 0.530498988 | 0.112741872 | 4.705429994 | 2.53E-06 | 7.87E-05 | up |
| OR51E1 | 135.2223336 | 0.666195364 | 0.141588261 | 4.705159583 | 2.54E-06 | 7.88E-05 | up |
| ZNF134 | 214.1802872 | -0.524089225 | 0.111501204 | -4.700301035 | 2.60E-06 | 8.04E-05 | down |
| TMEM132C | 21.47951484 | -1.091274028 | 0.232174572 | -4.700230602 | 2.60E-06 | 8.04E-05 | down |
| LINC01954 | 2.331882982 | -0.993038047 | 0.211538012 | -4.694371657 | 2.67E-06 | 8.27E-05 | down |
| FAM83A-AS1 | 2.900804144 | 1.040523556 | 0.221708248 | 4.693210855 | 2.69E-06 | 8.28E-05 | up |
| LINC00654 | 216.6192561 | -0.701952524 | 0.149573107 | -4.693039661 | 2.69E-06 | 8.28E-05 | down |
| AC066613.1 | 11.61460791 | 0.502447662 | 0.107067479 | 4.692813026 | 2.69E-06 | 8.28E-05 | up |
| GPR142 | 2.033068639 | 0.912389542 | 0.1947297 | 4.685415434 | 2.79E-06 | 8.55E-05 | up |
| RPS3AP49 | 16.1334276 | -0.669620307 | 0.143160021 | -4.677425304 | 2.90E-06 | 8.86E-05 | down |
| MTCL1 | 482.3790038 | 0.578407505 | 0.12376449 | 4.673452838 | 2.96E-06 | 9.01E-05 | up |
| DIRAS3 | 21.21199268 | 0.573582818 | 0.122811 | 4.670451483 | 3.01E-06 | 9.14E-05 | up |
| PMEPA1 | 6948.339902 | -0.518062933 | 0.110962765 | -4.66879977 | 3.03E-06 | 9.19E-05 | down |
| HOXC5 | 1.214771326 | 1.300732034 | 0.278621863 | 4.668449279 | 3.03E-06 | 9.20E-05 | up |
| ARSJ | 337.145243 | 0.568419644 | 0.121884924 | 4.663576312 | 3.11E-06 | 9.40E-05 | up |
| HOXC-AS2 | 6.003092455 | 1.255730954 | 0.269273671 | 4.66340043 | 3.11E-06 | 9.40E-05 | up |
| LRMDA | 138.6005409 | -0.554007792 | 0.118805002 | -4.663168928 | 3.11E-06 | 9.41E-05 | down |
| RAB3B | 185.1179522 | 0.700303878 | 0.150203652 | 4.662362516 | 3.13E-06 | 9.43E-05 | up |
| AC009093.3 | 24.00486499 | -0.845548806 | 0.181465618 | -4.659553767 | 3.17E-06 | 9.54E-05 | down |
| THRB | 213.267925 | -0.653012598 | 0.140207763 | -4.657463933 | 3.20E-06 | 9.61E-05 | down |
| EDA | 211.7719158 | -0.769894963 | 0.165304401 | -4.657437785 | 3.20E-06 | 9.61E-05 | down |
| DAPP1 | 242.5131828 | 0.543438249 | 0.116701331 | 4.65665853 | 3.21E-06 | 9.64E-05 | up |
| AC067930.5 | 18.42471538 | 0.663109606 | 0.14241421 | 4.656203925 | 3.22E-06 | 9.64E-05 | up |
| CPNE6 | 25.9172543 | -0.962774062 | 0.206887234 | -4.653617541 | 3.26E-06 | 9.75E-05 | down |
| IL12RB1 | 165.6006182 | 0.529496977 | 0.113809539 | 4.65248326 | 3.28E-06 | 9.79E-05 | up |
| AC004923.1 | 10.45180896 | -0.502617678 | 0.108070152 | -4.650846401 | 3.31E-06 | 9.85E-05 | down |
| CPN1 | 26.89877492 | -1.324786924 | 0.285071647 | -4.647206895 | 3.36E-06 | 0.000100014 | down |
| KRT87P | 4.549357183 | 0.795468866 | 0.171563288 | 4.63659141 | 3.54E-06 | 0.000105008 | up |
| AL731533.2 | 3.637138636 | 0.550004498 | 0.118653169 | 4.635396623 | 3.56E-06 | 0.000105429 | up |
| AC003991.2 | 11.84769395 | -0.828884091 | 0.178859711 | -4.634269441 | 3.58E-06 | 0.000105818 | down |
| EP300-AS1 | 9.657744808 | 0.505747329 | 0.109177771 | 4.632328761 | 3.62E-06 | 0.000106644 | up |
| AL031055.1 | 3.990874766 | -0.526084052 | 0.113577707 | -4.631930573 | 3.62E-06 | 0.000106644 | down |
| ADARB2 | 16.30205437 | -0.71513204 | 0.154469401 | -4.629603248 | 3.66E-06 | 0.000107754 | down |
| SEZ6 | 5.907673889 | 0.992816615 | 0.214463119 | 4.62931165 | 3.67E-06 | 0.000107811 | up |
| APLP1 | 49.52704488 | 0.546145629 | 0.117993573 | 4.628604908 | 3.68E-06 | 0.000108085 | up |
| NRXN2 | 165.8784893 | -0.656165112 | 0.141832166 | -4.626349083 | 3.72E-06 | 0.000109172 | down |
| NDUFA4L2 | 781.4505042 | 0.702509188 | 0.15186775 | 4.625795705 | 3.73E-06 | 0.000109368 | up |
| F7 | 68.6845572 | -1.028015907 | 0.222265151 | -4.625178099 | 3.74E-06 | 0.000109599 | down |
| ZNRF2P1 | 18.13852273 | -0.501730866 | 0.108564468 | -4.621501614 | 3.81E-06 | 0.000111267 | down |
| AC074135.1 | 6.169573539 | -1.2105915 | 0.262026498 | -4.620110973 | 3.84E-06 | 0.00011182 | down |
| FSTL4 | 37.25841043 | -0.930273945 | 0.201382088 | -4.619447316 | 3.85E-06 | 0.000112081 | down |
| AC007608.4 | 20.427413 | -1.076632807 | 0.233546048 | -4.609938028 | 4.03E-06 | 0.000116823 | down |
| SNORD8 | 0.840672996 | -1.978062299 | 0.429171801 | -4.609022059 | 4.05E-06 | 0.000117237 | down |
| FBLL1 | 12.67530688 | -0.887072822 | 0.192786602 | -4.601319865 | 4.20E-06 | 0.000121342 | down |
| DLX1 | 9.617987045 | 1.019035539 | 0.221861968 | 4.593106019 | 4.37E-06 | 0.000125676 | up |
| MRPL40P1 | 1.694667006 | 0.821551888 | 0.178969373 | 4.590460775 | 4.42E-06 | 0.00012717 | up |
| MIR503HG | 46.41730423 | -0.640677054 | 0.139735442 | -4.584928808 | 4.54E-06 | 0.00013036 | down |
| SAA2 | 119.1627894 | 0.887409668 | 0.193700128 | 4.581358195 | 4.62E-06 | 0.000132303 | up |
| PGLYRP3 | 6.244119681 | 1.432159885 | 0.312610058 | 4.581298162 | 4.62E-06 | 0.000132303 | up |
| AC093525.6 | 4.6311502 | 0.982388298 | 0.214502812 | 4.579838776 | 4.65E-06 | 0.000132889 | up |
| TIGIT | 121.8941741 | 0.555214208 | 0.121259598 | 4.5787238 | 4.68E-06 | 0.000133485 | up |
| SNORA84 | 1.27647345 | -1.153799873 | 0.25220735 | -4.574806691 | 4.77E-06 | 0.000135775 | down |
| RN7SL208P | 2.959228371 | 0.557106988 | 0.121812787 | 4.573468847 | 4.80E-06 | 0.000136345 | up |
| AC034206.1 | 4.271561772 | -0.912990992 | 0.199630887 | -4.573395471 | 4.80E-06 | 0.000136345 | down |
| PHYHIPL | 81.8743272 | -1.118802332 | 0.244776307 | -4.570713342 | 4.86E-06 | 0.000137867 | down |
| PCDH19 | 101.5725079 | -0.81739658 | 0.17893304 | -4.568170196 | 4.92E-06 | 0.000139314 | down |
| AL162274.3 | 6.239476271 | 0.592447192 | 0.129708525 | 4.567527011 | 4.94E-06 | 0.000139595 | up |
| CXCL17 | 53.10046813 | 1.177442673 | 0.257793174 | 4.567392747 | 4.94E-06 | 0.000139595 | up |
| KLRC1 | 10.4371391 | 0.74050784 | 0.162192207 | 4.565619105 | 4.98E-06 | 0.000140662 | up |
| IGDCC3 | 6.880219742 | 1.190614978 | 0.2607966 | 4.565301002 | 4.99E-06 | 0.000140756 | up |
| FCRL1 | 14.82560584 | 1.026390487 | 0.224948436 | 4.562781176 | 5.05E-06 | 0.000141978 | up |
| ITIH4 | 4.731383557 | 0.642548639 | 0.140848607 | 4.5619808 | 5.07E-06 | 0.000142281 | up |
| MAEL | 8.612003002 | -0.881441014 | 0.193417435 | -4.557195241 | 5.18E-06 | 0.000144962 | down |
| KRT8P17 | 5.305794019 | 0.746387279 | 0.163783706 | 4.55715222 | 5.19E-06 | 0.000144962 | up |
| FAM72B | 29.26343424 | 0.686212486 | 0.150641204 | 4.55527748 | 5.23E-06 | 0.000146036 | up |
| TRGC1 | 9.269312862 | 0.622569927 | 0.136771126 | 4.55191051 | 5.32E-06 | 0.000148196 | up |
| SP8 | 33.11488299 | 1.058108409 | 0.232518477 | 4.550642264 | 5.35E-06 | 0.000148794 | up |
| GLIS3-AS1 | 5.965860456 | 1.114787293 | 0.244999773 | 4.550156432 | 5.36E-06 | 0.000149014 | up |
| AP003555.2 | 8.511543763 | 0.719552337 | 0.158211221 | 4.548048695 | 5.41E-06 | 0.000150389 | up |
| SCARNA22 | 3.305329407 | -0.82840314 | 0.18217027 | -4.547411267 | 5.43E-06 | 0.00015047 | down |
| DNAH5 | 56.84205378 | 0.551346625 | 0.121311781 | 4.544872908 | 5.50E-06 | 0.000151917 | up |
| GPR143 | 281.0520154 | -0.743863128 | 0.163827607 | -4.540523661 | 5.61E-06 | 0.000154702 | down |
| PNLIPRP1 | 6.681323765 | -0.74359211 | 0.163791145 | -4.53987979 | 5.63E-06 | 0.000155047 | down |
| XAF1 | 283.6084986 | 0.502180977 | 0.110673241 | 4.537510337 | 5.69E-06 | 0.000156412 | up |
| SLC17A8 | 1.986234497 | 1.301698606 | 0.286926766 | 4.536692842 | 5.71E-06 | 0.000156762 | up |
| MAP7D2 | 357.2549594 | -0.976252892 | 0.215246545 | -4.535510152 | 5.75E-06 | 0.000157514 | down |
| PCP4L1 | 11.03691431 | -0.894810954 | 0.197457499 | -4.531663568 | 5.85E-06 | 0.000160147 | down |
| HTR3A | 10.31757735 | 0.989998403 | 0.21857005 | 4.529433024 | 5.91E-06 | 0.000161583 | up |
| KIR3DL2 | 1.915705636 | 1.175644039 | 0.259591742 | 4.5288191 | 5.93E-06 | 0.000161789 | up |
| EPYC | 47.86528716 | -1.137922255 | 0.251384343 | -4.52662343 | 5.99E-06 | 0.000163212 | down |
| ADAM28 | 247.1073538 | 0.531880725 | 0.117512335 | 4.526169322 | 6.01E-06 | 0.00016343 | up |
| MUC17 | 3021.911645 | 1.078304718 | 0.238301596 | 4.52495803 | 6.04E-06 | 0.000164235 | up |
| VWA5B1 | 25.33273161 | -1.366287887 | 0.302137578 | -4.522072017 | 6.12E-06 | 0.000165951 | down |
| BEX2 | 154.4117623 | -0.896518426 | 0.198363776 | -4.519567249 | 6.20E-06 | 0.000166989 | down |
| HEATR9 | 2.12215453 | 0.783768866 | 0.173425731 | 4.519334358 | 6.20E-06 | 0.000166989 | up |
| SAMD9 | 690.6128887 | 0.50987784 | 0.112822734 | 4.51928281 | 6.20E-06 | 0.000166989 | up |
| AC098864.1 | 10.29936028 | 0.94848953 | 0.210201189 | 4.512293837 | 6.41E-06 | 0.000171979 | up |
| 7SK | 33.41095843 | -0.881366075 | 0.19536457 | -4.511391565 | 6.44E-06 | 0.000172574 | down |
| AC099684.2 | 2.894990163 | -1.398503214 | 0.310006763 | -4.511202271 | 6.45E-06 | 0.00017259 | down |
| C1GALT1C1L | 15.73584273 | -0.687884878 | 0.152711228 | -4.504481337 | 6.65E-06 | 0.00017746 | down |
| C4orf48 | 625.6116193 | -0.664179663 | 0.147516794 | -4.502400337 | 6.72E-06 | 0.000178894 | down |
| YJEFN3 | 84.89360869 | 0.536118285 | 0.11911468 | 4.500858222 | 6.77E-06 | 0.00017991 | up |
| SLC6A16 | 13.10087427 | 0.569093182 | 0.126532905 | 4.497590417 | 6.87E-06 | 0.000182065 | up |
| SERPINB3 | 6.648474626 | 2.052746639 | 0.45687182 | 4.493047171 | 7.02E-06 | 0.000185314 | up |
| AC092811.1 | 6.166054609 | 0.937776766 | 0.208737104 | 4.492621339 | 7.04E-06 | 0.000185539 | up |
| CTF1 | 52.28612017 | -0.603486613 | 0.134374663 | -4.491074428 | 7.09E-06 | 0.000186581 | down |
| KRT18P11 | 21.06787689 | 0.50535108 | 0.112735175 | 4.482638899 | 7.37E-06 | 0.000193218 | up |
| HCRT | 4.23671241 | -0.858643054 | 0.191640649 | -4.480485001 | 7.45E-06 | 0.000194819 | down |
| EMX2 | 5.009166116 | -1.294809262 | 0.289149403 | -4.477993896 | 7.53E-06 | 0.000196698 | down |
| ASCL2 | 4090.046314 | -0.616762551 | 0.137766898 | -4.476855911 | 7.58E-06 | 0.000197595 | down |
| XPNPEP2 | 513.8294876 | -0.870987051 | 0.194879562 | -4.469360685 | 7.85E-06 | 0.000204169 | down |
| IL1R2 | 395.2498955 | 0.672092729 | 0.150401444 | 4.468658758 | 7.87E-06 | 0.000204681 | up |
| AP000696.1 | 1.70188598 | 1.245499894 | 0.278745638 | 4.468230977 | 7.89E-06 | 0.000204931 | up |
| KRT41P | 1.621809722 | -1.577453552 | 0.353321153 | -4.464645091 | 8.02E-06 | 0.000207909 | down |
| NBPF7 | 9.984178021 | 1.215602702 | 0.272290074 | 4.464366556 | 8.03E-06 | 0.000207956 | up |
| AC110772.1 | 4.21534781 | -1.607168387 | 0.36001189 | -4.464209194 | 8.04E-06 | 0.000207956 | down |
| AACSP1 | 1.762534898 | 1.285069548 | 0.28792672 | 4.46318268 | 8.08E-06 | 0.000208525 | up |
| HSPB6 | 278.1470446 | -0.792399809 | 0.177573145 | -4.462385388 | 8.11E-06 | 0.000209141 | down |
| CA9 | 1883.0967 | 0.879909551 | 0.19725553 | 4.460759854 | 8.17E-06 | 0.000210571 | up |
| CECR2 | 71.65880112 | -0.874411375 | 0.196132378 | -4.458271431 | 8.26E-06 | 0.000212702 | down |
| CHRNA7 | 29.86874628 | 0.929161043 | 0.208424158 | 4.458029491 | 8.27E-06 | 0.000212779 | up |
| LINC01923 | 2.141866748 | -1.384976049 | 0.310755485 | -4.456803223 | 8.32E-06 | 0.000213835 | down |
| CYP4F2 | 315.5009825 | -0.815420327 | 0.183168299 | -4.451754652 | 8.52E-06 | 0.000218589 | down |
| AC099518.6 | 3.221624166 | 0.64687817 | 0.145324772 | 4.451258797 | 8.54E-06 | 0.000218927 | up |
| TRG-AS1 | 28.27182019 | 0.503276611 | 0.11308898 | 4.450271028 | 8.58E-06 | 0.000219768 | up |
| ACE2 | 1646.490473 | -0.698300984 | 0.156971296 | -4.448590292 | 8.64E-06 | 0.000221026 | down |
| IFNG | 12.45055781 | 0.784518401 | 0.176402719 | 4.447314672 | 8.70E-06 | 0.000221963 | up |
| RNF2P1 | 1.448178605 | -1.15556717 | 0.260111185 | -4.44258931 | 8.89E-06 | 0.000225861 | down |
| ZNF257 | 16.17205332 | -0.859940483 | 0.193679677 | -4.440014021 | 9.00E-06 | 0.000228356 | down |
| FRMPD1 | 19.45821679 | 0.787292048 | 0.177322084 | 4.439898459 | 9.00E-06 | 0.000228356 | up |
| NKILA | 104.0009532 | -0.541098637 | 0.121919276 | -4.43817134 | 9.07E-06 | 0.000229847 | down |
| DBH | 9.498618934 | 0.693023358 | 0.15618173 | 4.43728827 | 9.11E-06 | 0.000230443 | up |
| AC106785.4 | 4.996295124 | 0.6957035 | 0.156818641 | 4.436357157 | 9.15E-06 | 0.000231138 | up |
| AC007608.2 | 3.093920339 | -1.341020321 | 0.302342738 | -4.43543089 | 9.19E-06 | 0.000231912 | down |
| RN7SL118P | 1.259884641 | -1.089374858 | 0.245718965 | -4.433417899 | 9.28E-06 | 0.000233736 | down |
| FSCN1 | 2462.598009 | 0.584283641 | 0.131955299 | 4.427890711 | 9.52E-06 | 0.000239085 | up |
| AL365223.1 | 2.59249331 | -0.869473836 | 0.196430498 | -4.426368846 | 9.58E-06 | 0.000240596 | down |
| AC002401.4 | 1.699720011 | 1.03873929 | 0.234743649 | 4.424994221 | 9.64E-06 | 0.000241748 | up |
| PROK1 | 1.376935619 | -1.599481306 | 0.361476793 | -4.424851982 | 9.65E-06 | 0.000241748 | down |
| SNRPGP10 | 22.9992061 | -0.564516607 | 0.127617238 | -4.423513755 | 9.71E-06 | 0.000242706 | down |
| HOXD1 | 18.65390579 | 0.698286444 | 0.157940601 | 4.421196578 | 9.82E-06 | 0.00024514 | up |
| ACKR4 | 24.02068507 | 0.683377133 | 0.15466507 | 4.41843226 | 9.94E-06 | 0.000248111 | up |
| C1orf105 | 12.94005089 | -0.730900276 | 0.165486461 | -4.416677184 | 1.00E-05 | 0.00024976 | down |
| SPTB | 44.23625395 | 0.53665074 | 0.121540285 | 4.415414509 | 1.01E-05 | 0.000251036 | up |
| DEFB1 | 105.4720713 | -0.737891143 | 0.167419365 | -4.407442002 | 1.05E-05 | 0.000258911 | down |
| LINC01166 | 1.124263979 | 1.743764248 | 0.396005136 | 4.403387957 | 1.07E-05 | 0.000263214 | up |
| PCDHGA7 | 28.4313767 | -0.794312647 | 0.180415074 | -4.402695571 | 1.07E-05 | 0.000263835 | down |
| CSMD1 | 4.515055927 | 0.949897846 | 0.215760484 | 4.402557068 | 1.07E-05 | 0.000263835 | up |
| AC093585.1 | 7.331563785 | -0.786230754 | 0.178681405 | -4.400182301 | 1.08E-05 | 0.000265954 | down |
| PLA2G3 | 89.3761955 | 1.134834262 | 0.258000532 | 4.398573339 | 1.09E-05 | 0.000267267 | up |
| NMUR2 | 17.91764384 | 1.327684269 | 0.301850953 | 4.398476313 | 1.09E-05 | 0.000267267 | up |
| PALM3 | 135.1009893 | -0.74978236 | 0.170496782 | -4.397633487 | 1.09E-05 | 0.00026811 | down |
| JAKMIP1 | 24.34899923 | 0.560267871 | 0.127431776 | 4.3966104 | 1.10E-05 | 0.000268982 | up |
| LINC01913 | 2.702678432 | 1.355261113 | 0.308319321 | 4.395641214 | 1.10E-05 | 0.000269988 | up |
| CERS4 | 189.6853045 | -0.616890372 | 0.140364137 | -4.394928677 | 1.11E-05 | 0.000270677 | down |
| REEP1 | 259.6438496 | -0.74320398 | 0.169189769 | -4.392724115 | 1.12E-05 | 0.000273238 | down |
| ATP6V1B1 | 17.59639785 | 0.707651847 | 0.161119856 | 4.392083414 | 1.12E-05 | 0.000273645 | up |
| RNU5D-1 | 0.699734131 | -1.63152881 | 0.371624971 | -4.390256133 | 1.13E-05 | 0.000275353 | down |
| LRFN2 | 16.74940951 | -1.095203367 | 0.249504909 | -4.389506291 | 1.14E-05 | 0.000275902 | down |
| LINC01215 | 11.19166925 | 0.693106232 | 0.157911752 | 4.38919981 | 1.14E-05 | 0.000276013 | up |
| TIMP3 | 253.5326598 | -0.563887957 | 0.128474525 | -4.389103263 | 1.14E-05 | 0.000276013 | down |
| FZD10-AS1 | 108.9404911 | -0.781918336 | 0.178196778 | -4.387948789 | 1.14E-05 | 0.00027708 | down |
| OR13A1 | 1.630047564 | 0.875227039 | 0.199563482 | 4.385707392 | 1.16E-05 | 0.000279544 | up |
| CPNE9 | 19.73809255 | -0.612264946 | 0.139619197 | -4.385249029 | 1.16E-05 | 0.000279931 | down |
| SLC25A21 | 19.1551455 | 0.880972875 | 0.20108576 | 4.381080358 | 1.18E-05 | 0.000284288 | up |
| AP001554.1 | 5.148156147 | -0.869494292 | 0.198697246 | -4.375975563 | 1.21E-05 | 0.000289384 | down |
| SCUBE3 | 157.01988 | -0.703919406 | 0.161107746 | -4.369246206 | 1.25E-05 | 0.000297369 | down |
| AC022182.2 | 15.52524221 | 0.766855156 | 0.175517869 | 4.369100191 | 1.25E-05 | 0.000297369 | up |
| ACSL6 | 835.2968719 | -0.836329874 | 0.191588669 | -4.365236624 | 1.27E-05 | 0.000301599 | down |
| TMEM238 | 809.3733481 | -0.582117617 | 0.133359831 | -4.365014661 | 1.27E-05 | 0.000301691 | down |
| S100A4 | 2997.573576 | -0.540179256 | 0.123792427 | -4.36358888 | 1.28E-05 | 0.000303235 | down |
| KRT42P | 4.009648898 | 1.025568833 | 0.23503854 | 4.363407095 | 1.28E-05 | 0.000303272 | up |
| VWCE | 55.21431657 | -0.682577978 | 0.156451369 | -4.362876338 | 1.28E-05 | 0.000303704 | down |
| CECR7 | 15.23362317 | 0.863552025 | 0.197935897 | 4.362786319 | 1.28E-05 | 0.000303704 | up |
| SNORA28 | 2.25009496 | -1.021837616 | 0.2342542 | -4.362088769 | 1.29E-05 | 0.000304029 | down |
| KRT2 | 2.790109689 | 1.029724041 | 0.236110047 | 4.361203826 | 1.29E-05 | 0.000304837 | up |
| OMD | 25.76346887 | -0.972474585 | 0.223018547 | -4.360509921 | 1.30E-05 | 0.000305584 | down |
| KCND3 | 176.2698387 | 0.618445951 | 0.142193678 | 4.349321018 | 1.37E-05 | 0.000319773 | up |
| SLC28A2 | 332.1380996 | 1.041063995 | 0.239369997 | 4.34918331 | 1.37E-05 | 0.000319773 | up |
| LINC00525 | 23.04015864 | -0.585569043 | 0.134652599 | -4.348739265 | 1.37E-05 | 0.000319974 | down |
| HMGN2P17 | 24.12174774 | 0.735069767 | 0.169246198 | 4.343198096 | 1.40E-05 | 0.000327926 | up |
| AC005498.3 | 12.78627555 | -0.55941335 | 0.128813195 | -4.342826435 | 1.41E-05 | 0.000328252 | down |
| EOMES | 33.37054707 | 0.654467343 | 0.150718424 | 4.342318109 | 1.41E-05 | 0.000328579 | up |
| AC004009.1 | 5.632906586 | 1.509500693 | 0.347639122 | 4.342148506 | 1.41E-05 | 0.000328579 | up |
| Z98257.1 | 3.714749335 | 0.998329927 | 0.229941245 | 4.341674002 | 1.41E-05 | 0.00032906 | up |
| POU2AF1 | 314.2058081 | 0.684384059 | 0.157675727 | 4.340452841 | 1.42E-05 | 0.000330664 | up |
| LINC02446 | 12.4266564 | 0.923766934 | 0.212847791 | 4.34003534 | 1.42E-05 | 0.000331063 | up |
| KCNQ2 | 13.83348651 | 1.088492727 | 0.250843324 | 4.339333053 | 1.43E-05 | 0.000331892 | up |
| FOXI1 | 16.8982965 | 1.327719338 | 0.306017284 | 4.338707016 | 1.43E-05 | 0.000332608 | up |
| SERTAD4 | 137.6765566 | 0.56707742 | 0.130734315 | 4.337632563 | 1.44E-05 | 0.000334006 | up |
| KRT6A | 152.9822109 | 1.080315405 | 0.249065936 | 4.337467503 | 1.44E-05 | 0.000334025 | up |
| SLC5A5 | 2.915231309 | -0.772221239 | 0.178090869 | -4.33610799 | 1.45E-05 | 0.000335399 | down |
| RHOF | 202.6688696 | 0.513861711 | 0.118523487 | 4.335526417 | 1.45E-05 | 0.000336055 | up |
| AL032819.2 | 6.027816279 | 0.733939222 | 0.169299631 | 4.335149557 | 1.46E-05 | 0.000336399 | up |
| ALDH1L1 | 699.2241603 | 0.887461676 | 0.204734265 | 4.334700291 | 1.46E-05 | 0.000336758 | up |
| CLDN18 | 292.0504815 | 1.335678944 | 0.30816852 | 4.33424849 | 1.46E-05 | 0.000337075 | up |
| CYMP-AS1 | 0.686269706 | 1.644899086 | 0.379524912 | 4.33410043 | 1.46E-05 | 0.000337075 | up |
| LINC02195 | 5.055090533 | 0.687998128 | 0.158780857 | 4.333004235 | 1.47E-05 | 0.000338228 | up |
| AC113146.2 | 3.279233145 | -0.729636584 | 0.168442451 | -4.331666874 | 1.48E-05 | 0.000339887 | down |
| CASC8 | 88.15928195 | 0.661800089 | 0.152799663 | 4.331161982 | 1.48E-05 | 0.0003402 | up |
| RNA5-8SP6 | 1.076310402 | -1.811887454 | 0.418461819 | -4.3298752 | 1.49E-05 | 0.00034196 | down |
| SCARNA21 | 0.901856116 | -1.16300028 | 0.268680764 | -4.328558029 | 1.50E-05 | 0.000343775 | down |
| ZNF662 | 78.62363841 | -0.678638881 | 0.156818849 | -4.327533863 | 1.51E-05 | 0.000345141 | down |
| NCR1 | 5.1032353 | 0.743278756 | 0.171807454 | 4.326231124 | 1.52E-05 | 0.000346713 | up |
| CTD-3080P12.3 | 4.014657426 | -1.114649668 | 0.257688287 | -4.325573663 | 1.52E-05 | 0.000347511 | down |
| ZNF831 | 27.82301402 | 0.607143631 | 0.140476152 | 4.322040587 | 1.55E-05 | 0.000351922 | up |
| BARX2 | 231.49589 | 0.693106257 | 0.160433851 | 4.320199593 | 1.56E-05 | 0.000353907 | up |
| S100A7 | 5.882924071 | 1.316950783 | 0.304951981 | 4.318551331 | 1.57E-05 | 0.000355133 | up |
| AC010319.1 | 7.105887766 | 0.570506694 | 0.132106392 | 4.318539669 | 1.57E-05 | 0.000355133 | up |
| SYT3 | 6.548806415 | -0.712049532 | 0.164916496 | -4.317636802 | 1.58E-05 | 0.000355839 | down |
| AC027348.1 | 7.833995986 | -0.800621786 | 0.185476523 | -4.316566719 | 1.58E-05 | 0.000357113 | down |
| MTATP6P2 | 5.658978244 | 0.744274385 | 0.172620589 | 4.311620015 | 1.62E-05 | 0.000364461 | up |
| BHMT | 7.024756921 | 1.184301228 | 0.274703614 | 4.31119638 | 1.62E-05 | 0.000364915 | up |
| RPL21P67 | 4.666538578 | -1.029275723 | 0.238774131 | -4.310666823 | 1.63E-05 | 0.000365544 | down |
| AC004231.1 | 9.504572059 | -0.937326652 | 0.217479111 | -4.309961757 | 1.63E-05 | 0.000366334 | down |
| SNORA79B | 3.369577474 | -0.838871204 | 0.194646774 | -4.309710287 | 1.63E-05 | 0.000366391 | down |
| HNF4A-AS1 | 4.534440356 | -0.738011124 | 0.171296803 | -4.308376516 | 1.64E-05 | 0.00036836 | down |
| CASP16P | 17.55431605 | 0.609286576 | 0.141456389 | 4.307239724 | 1.65E-05 | 0.00037001 | up |
| AP000911.1 | 6.398644354 | -0.659587232 | 0.153173901 | -4.306133287 | 1.66E-05 | 0.000371616 | down |
| LMX1A | 3.62079025 | -1.199456849 | 0.278625068 | -4.304913615 | 1.67E-05 | 0.000373419 | down |
| MRPS18AP1 | 4.356956357 | 0.552165869 | 0.128312898 | 4.303276408 | 1.68E-05 | 0.000374438 | up |
| TNFRSF13C | 71.20455343 | 0.55601326 | 0.129225731 | 4.302651317 | 1.69E-05 | 0.000374998 | up |
| B3GNT7 | 1068.142651 | -0.697296184 | 0.162086342 | -4.302004563 | 1.69E-05 | 0.000375844 | down |
| LILRP2 | 3.107719168 | 0.731848699 | 0.170149646 | 4.301206107 | 1.70E-05 | 0.000376951 | up |
| FSIP1 | 27.93850048 | -0.660951749 | 0.153748162 | -4.298924547 | 1.72E-05 | 0.000380347 | down |
| CES1 | 708.3066024 | -0.881647507 | 0.205139591 | -4.297793034 | 1.73E-05 | 0.00038204 | down |
| GABRQ | 4.294533988 | -0.697690563 | 0.162410847 | -4.295837234 | 1.74E-05 | 0.000384153 | down |
| ZNF43 | 201.4255628 | -0.652477676 | 0.151929661 | -4.294603646 | 1.75E-05 | 0.000385893 | down |
| SPRR1A | 23.06133641 | 1.454491973 | 0.338683859 | 4.294541752 | 1.75E-05 | 0.000385893 | up |
| FFAR4 | 626.9975485 | 0.686777077 | 0.159961389 | 4.293392802 | 1.76E-05 | 0.000387386 | up |
| ARNT2 | 225.3205703 | 0.591489544 | 0.137780884 | 4.292972495 | 1.76E-05 | 0.000387865 | up |
| ATP8B3 | 41.42330111 | 0.569837875 | 0.132767565 | 4.291996119 | 1.77E-05 | 0.000389318 | up |
| HIST1H3F | 5.427906067 | -0.868096187 | 0.20236537 | -4.289746743 | 1.79E-05 | 0.000392251 | down |
| AKAP4 | 3.739293431 | -1.03675216 | 0.241761636 | -4.288323727 | 1.80E-05 | 0.000394513 | down |
| NPFFR1 | 132.6417008 | -0.581437889 | 0.135673307 | -4.285573219 | 1.82E-05 | 0.000398382 | down |
| AL662890.2 | 5.358327882 | 0.609323932 | 0.142236357 | 4.283883138 | 1.84E-05 | 0.00040116 | up |
| AC135388.1 | 5.975730357 | -1.042851655 | 0.243459514 | -4.283470541 | 1.84E-05 | 0.000401604 | down |
| LINCR-0001 | 21.01816355 | -0.805171163 | 0.188064048 | -4.281366747 | 1.86E-05 | 0.000404666 | down |
| COL6A5 | 6.317068269 | 0.93581341 | 0.218660065 | 4.279763698 | 1.87E-05 | 0.000407062 | up |
| SNORA80B | 3.579839368 | -0.804622188 | 0.188024445 | -4.279348818 | 1.87E-05 | 0.000407291 | down |
| CEACAM8 | 17.29517746 | -0.765434306 | 0.178914982 | -4.278201295 | 1.88E-05 | 0.000408865 | down |
| MS4A1 | 96.59215598 | 0.921456386 | 0.215573407 | 4.274443684 | 1.92E-05 | 0.000414476 | up |
| AC022148.1 | 1.369602203 | -0.938829659 | 0.219736075 | -4.272533118 | 1.93E-05 | 0.000416697 | down |
| RIPPLY3 | 45.1209646 | -0.767067826 | 0.179600485 | -4.270967447 | 1.95E-05 | 0.000419093 | down |
| AL133325.3 | 6.921034884 | 1.007275133 | 0.235937971 | 4.269237069 | 1.96E-05 | 0.000422008 | up |
| KLK10 | 1956.34595 | 0.80511068 | 0.188588733 | 4.269134573 | 1.96E-05 | 0.000422008 | up |
| AC011840.1 | 1.411112847 | 3.031870386 | 0.710601069 | 4.266627955 | 1.98E-05 | 0.000426227 | up |
| VIT | 12.32740037 | 0.699055234 | 0.163890708 | 4.265374418 | 2.00E-05 | 0.000427793 | up |
| CR2 | 106.9824195 | 0.864373788 | 0.202718335 | 4.263915202 | 2.01E-05 | 0.000430057 | up |
| RDH16 | 26.05601809 | 0.574861978 | 0.134861745 | 4.262602239 | 2.02E-05 | 0.000431763 | up |
| HUNK | 1240.353475 | -0.573423273 | 0.134530508 | -4.262403224 | 2.02E-05 | 0.000431872 | down |
| AC079150.2 | 0.687892874 | -1.283194664 | 0.301099315 | -4.261699043 | 2.03E-05 | 0.000432528 | down |
| AL157871.2 | 6.223532612 | 0.579837959 | 0.136124201 | 4.259624349 | 2.05E-05 | 0.000436162 | up |
| GPM6A | 26.17753563 | -1.024316276 | 0.240484825 | -4.259380091 | 2.05E-05 | 0.000436361 | down |
| KIAA1549L | 166.5951254 | 0.77575805 | 0.182148658 | 4.258928172 | 2.05E-05 | 0.000436965 | up |
| NKX3-2 | 42.48937793 | -0.53460089 | 0.125561224 | -4.257690977 | 2.07E-05 | 0.000439111 | down |
| ABCC2 | 248.8589146 | -0.762363633 | 0.179341212 | -4.250911575 | 2.13E-05 | 0.000449758 | down |
| AC020907.1 | 8.884231201 | 1.130789114 | 0.266161217 | 4.248511958 | 2.15E-05 | 0.000454028 | up |
| COL9A1 | 156.2199156 | -0.734004486 | 0.172789203 | -4.247976562 | 2.16E-05 | 0.000454827 | down |
| KNG1 | 22.4202178 | -0.844100233 | 0.198786173 | -4.246272373 | 2.17E-05 | 0.000457146 | down |
| NKD2 | 875.9510185 | -0.588440762 | 0.13860378 | -4.245488556 | 2.18E-05 | 0.00045817 | down |
| MATN3 | 103.6125184 | -0.63790348 | 0.150264568 | -4.24520223 | 2.18E-05 | 0.000458263 | down |
| PDE3A | 669.4651361 | -0.561240064 | 0.132297056 | -4.242271743 | 2.21E-05 | 0.000463915 | down |
| SYNGR3 | 127.6862363 | 0.639796242 | 0.150854811 | 4.241139123 | 2.22E-05 | 0.00046597 | up |
| FNDC11 | 41.6818855 | -0.572397601 | 0.135105898 | -4.236658875 | 2.27E-05 | 0.000474308 | down |
| LINC01871 | 20.01559623 | 0.563289871 | 0.132961268 | 4.236495939 | 2.27E-05 | 0.000474308 | up |
| AL358334.2 | 1.844219266 | -0.66343988 | 0.15669759 | -4.233886944 | 2.30E-05 | 0.000478862 | down |
| CCDC160 | 2.167953828 | 1.208189673 | 0.285378691 | 4.233636612 | 2.30E-05 | 0.000479097 | up |
| ZNF334 | 69.53587306 | -0.922217554 | 0.217874606 | -4.232790465 | 2.31E-05 | 0.000480604 | down |
| ABCG2 | 103.551166 | 0.619734204 | 0.146451296 | 4.231674434 | 2.32E-05 | 0.000482395 | up |
| AGBL4 | 12.0492562 | 0.814265157 | 0.192517398 | 4.229566595 | 2.34E-05 | 0.000486634 | up |
| AL161909.1 | 45.56247464 | -0.765776898 | 0.181083096 | -4.228870146 | 2.35E-05 | 0.000487839 | down |
| RNF212 | 44.81732411 | -0.651082207 | 0.154023715 | -4.227155587 | 2.37E-05 | 0.000490352 | down |
| AHCYP7 | 1.338835209 | -1.334890147 | 0.315851773 | -4.226318358 | 2.38E-05 | 0.000491874 | down |
| SRIP3 | 4.143297063 | -1.40218351 | 0.331797148 | -4.226026415 | 2.38E-05 | 0.000492208 | down |
| AC083809.1 | 20.88331925 | 0.744696578 | 0.176237745 | 4.225522624 | 2.38E-05 | 0.000492728 | up |
| ASIP | 4.463896069 | -0.5017312 | 0.118769764 | -4.224401752 | 2.40E-05 | 0.000494548 | down |
| SV2C | 31.22433512 | -0.849332569 | 0.201183285 | -4.22168555 | 2.42E-05 | 0.000499621 | down |
| AL049873.2 | 16.79868004 | -0.548904907 | 0.130080713 | -4.219725531 | 2.45E-05 | 0.000503054 | down |
| CAMKV | 40.6714257 | -0.938911016 | 0.222597146 | -4.217983167 | 2.46E-05 | 0.000506588 | down |
| AL445933.1 | 17.29704739 | 0.64299888 | 0.152446351 | 4.21786993 | 2.47E-05 | 0.000506588 | up |
| MIR3142HG | 119.517051 | 0.55733663 | 0.132206148 | 4.215663491 | 2.49E-05 | 0.000510842 | up |
| AC018553.1 | 9.421858813 | 1.128417116 | 0.267756907 | 4.214334301 | 2.51E-05 | 0.000513045 | up |
| ASB5 | 4.02418548 | -1.591367741 | 0.377625149 | -4.214146609 | 2.51E-05 | 0.000513126 | down |
| AC245100.7 | 7.283519091 | 0.537666966 | 0.127656834 | 4.211814982 | 2.53E-05 | 0.000517502 | up |
| AP002784.1 | 1.812906499 | 0.937009891 | 0.22263566 | 4.208714331 | 2.57E-05 | 0.000522963 | up |
| AL592071.1 | 1.176963971 | 0.668334534 | 0.158943421 | 4.204858116 | 2.61E-05 | 0.000530761 | up |
| TGFBR3L | 8.586865283 | 0.724004602 | 0.172216992 | 4.204025367 | 2.62E-05 | 0.000532287 | up |
| COL4A3 | 27.64696185 | -0.733349798 | 0.174443689 | -4.203934244 | 2.62E-05 | 0.000532287 | down |
| PLAG1 | 105.6498106 | -0.590534725 | 0.140600641 | -4.200085584 | 2.67E-05 | 0.000541087 | down |
| HIST1H3C | 6.132228892 | -0.915564481 | 0.218058146 | -4.198717161 | 2.68E-05 | 0.000543708 | down |
| ACTBP13 | 5.581382845 | 0.743141332 | 0.17702648 | 4.197910564 | 2.69E-05 | 0.000545317 | up |
| AC138466.4 | 1.079568086 | 1.296509602 | 0.308858805 | 4.197742082 | 2.70E-05 | 0.000545393 | up |
| RNU6ATAC | 2.531391754 | -1.215876302 | 0.289751475 | -4.196273036 | 2.71E-05 | 0.000548609 | down |
| PRF1 | 292.297101 | 0.532959251 | 0.127096757 | 4.193334764 | 2.75E-05 | 0.000553757 | up |
| PRKAR2B | 225.9002359 | 0.523438368 | 0.124898585 | 4.190907102 | 2.78E-05 | 0.000558707 | up |
| PCCA | 2390.486654 | -0.508046448 | 0.12133846 | -4.187019094 | 2.83E-05 | 0.000566997 | down |
| KIAA0087 | 3.129847497 | 0.990849425 | 0.236690703 | 4.186262553 | 2.84E-05 | 0.000568548 | up |
| MTNR1A | 12.87204439 | 0.726084554 | 0.173480141 | 4.185404442 | 2.85E-05 | 0.000570358 | up |
| MX1 | 2488.65501 | 0.531811626 | 0.127137037 | 4.182979546 | 2.88E-05 | 0.000575534 | up |
| CCDC83 | 0.865400532 | 1.126444632 | 0.26929471 | 4.18294378 | 2.88E-05 | 0.000575534 | up |
| LYZL4 | 1.374745941 | 1.057034586 | 0.252808586 | 4.181165685 | 2.90E-05 | 0.000578446 | up |
| MMEL1 | 155.4656297 | -0.534631584 | 0.127868093 | -4.18111798 | 2.90E-05 | 0.000578446 | down |
| Z97192.2 | 4.371465845 | 0.639011884 | 0.152869115 | 4.180124187 | 2.91E-05 | 0.000580634 | up |
| AC129926.1 | 2.006505628 | -1.097468141 | 0.262674346 | -4.178056047 | 2.94E-05 | 0.000585001 | down |
| SPATC1L | 302.8219442 | -0.586816756 | 0.140453535 | -4.178013445 | 2.94E-05 | 0.000585001 | down |
| ADRB1 | 35.61904355 | 0.673300493 | 0.161224273 | 4.176173228 | 2.96E-05 | 0.000589051 | up |
| GBA3 | 35.90714849 | 0.82992149 | 0.198757544 | 4.175547118 | 2.97E-05 | 0.000590322 | up |
| GRM8 | 529.1145218 | -0.734073086 | 0.175836215 | -4.174754817 | 2.98E-05 | 0.000592029 | down |
| ZBTB7C | 696.9460231 | 0.598058084 | 0.143277936 | 4.174111523 | 2.99E-05 | 0.000593352 | up |
| TNFSF9 | 361.2748012 | 0.667550618 | 0.15998722 | 4.172524641 | 3.01E-05 | 0.000596163 | up |
| MAP3K15 | 4.837812603 | 0.581576972 | 0.139383491 | 4.17249537 | 3.01E-05 | 0.000596163 | up |
| SIT1 | 92.19955063 | -0.505427369 | 0.121230126 | -4.169156492 | 3.06E-05 | 0.000603892 | down |
| RAET1E-AS1 | 12.18863383 | -0.724307653 | 0.173770261 | -4.168191087 | 3.07E-05 | 0.000606096 | down |
| C9orf66 | 10.61304657 | 0.605681374 | 0.145388592 | 4.16594841 | 3.10E-05 | 0.000611004 | up |
| RIMBP3 | 4.43319203 | 0.642304717 | 0.154190118 | 4.165667201 | 3.10E-05 | 0.000611398 | up |
| PACRG | 11.08218452 | -0.615951465 | 0.147924608 | -4.163955357 | 3.13E-05 | 0.000615278 | down |
| RNVU1-6 | 1.232502729 | -1.130470553 | 0.271593753 | -4.162358454 | 3.15E-05 | 0.000618869 | down |
| CFAP73 | 17.65376561 | 0.505345754 | 0.121441389 | 4.161231687 | 3.17E-05 | 0.000620837 | up |
| SLC22A12 | 1.495764375 | -1.33164833 | 0.320032067 | -4.16098406 | 3.17E-05 | 0.000620973 | down |
| DLX3 | 62.76622187 | -1.0657446 | 0.256132332 | -4.160913972 | 3.17E-05 | 0.000620973 | down |
| FEZF1-AS1 | 163.7664135 | 0.871594872 | 0.209513385 | 4.160091597 | 3.18E-05 | 0.000622848 | up |
| CDIPT-AS1 | 7.188366221 | -0.763795492 | 0.183614105 | -4.159786591 | 3.19E-05 | 0.000623316 | down |
| AQP2 | 2.884468191 | 1.014988136 | 0.244033624 | 4.159214293 | 3.19E-05 | 0.000624514 | up |
| FBXO17 | 106.6878002 | -0.588517624 | 0.141614829 | -4.155762705 | 3.24E-05 | 0.000633278 | down |
| MUC4 | 2986.796292 | 0.698352241 | 0.168063742 | 4.155281994 | 3.25E-05 | 0.00063424 | up |
| BX679664.3 | 21.94589906 | 0.574611754 | 0.138306019 | 4.154640257 | 3.26E-05 | 0.000635281 | up |
| WFDC21P | 72.83930902 | 0.650431993 | 0.156627009 | 4.152744767 | 3.29E-05 | 0.000639449 | up |
| CHST13 | 84.03042505 | -0.702913626 | 0.169324796 | -4.151274009 | 3.31E-05 | 0.000642832 | down |
| RNVU1-15 | 1.514205507 | -1.015971937 | 0.24473754 | -4.151271339 | 3.31E-05 | 0.000642832 | down |
| U1 | 1.417489654 | -0.76349111 | 0.183973719 | -4.15000096 | 3.32E-05 | 0.00064566 | down |
| SNORA14B | 5.014018526 | -0.59612375 | 0.143669068 | -4.149283907 | 3.34E-05 | 0.000647309 | down |
| AC112487.1 | 1.336800296 | 0.891996708 | 0.215091044 | 4.147065781 | 3.37E-05 | 0.000651405 | up |
| RPL23AP74 | 20.61680925 | -0.658685419 | 0.158832524 | -4.147043693 | 3.37E-05 | 0.000651405 | down |
| ZAR1 | 2.098970971 | -1.096241856 | 0.264416248 | -4.145894452 | 3.38E-05 | 0.000654303 | down |
| GREB1L | 37.41897963 | -0.957159048 | 0.230886342 | -4.145585404 | 3.39E-05 | 0.000654807 | down |
| IGF2BP1 | 147.7960045 | -1.14676197 | 0.276654605 | -4.145103494 | 3.40E-05 | 0.000655807 | down |
| AC012085.2 | 3.365050435 | -1.022346101 | 0.24669758 | -4.144126994 | 3.41E-05 | 0.000658227 | down |
| WNT11 | 834.0142885 | -0.768160682 | 0.185610964 | -4.138552302 | 3.50E-05 | 0.000671711 | down |
| KLHDC7B | 56.91951626 | 0.554142119 | 0.133930005 | 4.137550214 | 3.51E-05 | 0.000673876 | up |
| AL157714.2 | 3.782143108 | -1.234680833 | 0.298481542 | -4.13653998 | 3.53E-05 | 0.00067638 | down |
| AL121785.1 | 2.330365893 | -1.10608348 | 0.267428416 | -4.135998318 | 3.53E-05 | 0.00067728 | down |
| FOXA1 | 927.9997274 | 0.517081304 | 0.125036029 | 4.135458465 | 3.54E-05 | 0.000678486 | up |
| LINC01709 | 1.03544662 | 1.734630827 | 0.41952389 | 4.134760539 | 3.55E-05 | 0.000679772 | up |
| AL121957.1 | 0.692566257 | 1.06662586 | 0.258006798 | 4.134099828 | 3.56E-05 | 0.000680952 | up |
| B3GALT5-AS1 | 18.04127134 | -0.92716073 | 0.224302975 | -4.133519549 | 3.57E-05 | 0.000682284 | down |
| LINC01910 | 2.377589752 | 0.90745338 | 0.219652116 | 4.131320915 | 3.61E-05 | 0.000688058 | up |
| HLA-DRB1 | 8747.396326 | 0.510719147 | 0.123654835 | 4.130199577 | 3.62E-05 | 0.000690635 | up |
| KLRC3 | 1.285485497 | 0.861341387 | 0.208556102 | 4.130022461 | 3.63E-05 | 0.000690773 | up |
| MIR3609 | 0.893484763 | -1.586773541 | 0.384631173 | -4.125441861 | 3.70E-05 | 0.000703466 | down |
| NPM1P47 | 3.011520798 | 0.870119301 | 0.211019299 | 4.123411011 | 3.73E-05 | 0.000708089 | up |
| TAS1R3 | 147.4539814 | -0.63535243 | 0.154139055 | -4.121943198 | 3.76E-05 | 0.00071181 | down |
| MRAP2 | 317.7130357 | 0.520608197 | 0.12631381 | 4.121546156 | 3.76E-05 | 0.000712634 | up |
| PACERR | 2.112829582 | 0.884912525 | 0.214767334 | 4.120331091 | 3.78E-05 | 0.000715593 | up |
| RNU6-1280P | 1.102376031 | -1.078414971 | 0.261791259 | -4.119369673 | 3.80E-05 | 0.000716963 | down |
| MYBPC1 | 35.19980577 | 0.715958578 | 0.173865336 | 4.117891427 | 3.82E-05 | 0.000719951 | up |
| LINC00930 | 4.0074569 | 0.975722629 | 0.237091669 | 4.115381335 | 3.87E-05 | 0.000727012 | up |
| LINC01996 | 12.44818775 | -0.852994006 | 0.207360664 | -4.113576755 | 3.90E-05 | 0.000730667 | down |
| SPINK1 | 4690.920631 | -0.602097602 | 0.146404932 | -4.11255 | 3.91E-05 | 0.000733103 | down |
| AGTR1 | 16.89388021 | -0.891349518 | 0.217048367 | -4.106686122 | 4.01E-05 | 0.000748601 | down |
| OCSTAMP | 1.630679844 | -0.839790323 | 0.204517201 | -4.106208766 | 4.02E-05 | 0.000749731 | down |
| AC010468.1 | 31.15886986 | -0.501702224 | 0.122190068 | -4.105916568 | 4.03E-05 | 0.000750261 | down |
| AGR3 | 1294.026026 | 0.636449402 | 0.155021243 | 4.1055625 | 4.03E-05 | 0.000750665 | up |
| NPTX2 | 941.8861703 | -0.919759513 | 0.224095299 | -4.10432311 | 4.06E-05 | 0.000753561 | down |
| HOXB8 | 1306.912219 | 0.590395156 | 0.143849388 | 4.104259066 | 4.06E-05 | 0.000753561 | up |
| NELL2 | 100.9038122 | 0.661804316 | 0.161408313 | 4.100187311 | 4.13E-05 | 0.000766092 | up |
| AC019109.1 | 4.737933558 | 0.926047004 | 0.22586703 | 4.099965386 | 4.13E-05 | 0.000766402 | up |
| SYNM | 587.0520962 | -0.675024531 | 0.164666892 | -4.099333643 | 4.14E-05 | 0.00076807 | down |
| AL031733.2 | 2.209382663 | 0.806898682 | 0.196955328 | 4.096861413 | 4.19E-05 | 0.000774598 | up |
| C10orf91 | 27.57311813 | 0.533901119 | 0.130331559 | 4.0964838 | 4.19E-05 | 0.000775433 | up |
| AC007207.2 | 4.201321286 | 1.111038072 | 0.271288377 | 4.095413463 | 4.21E-05 | 0.000777735 | up |
| CHRFAM7A | 5.747520243 | 0.768793397 | 0.188164617 | 4.085749002 | 4.39E-05 | 0.0008086 | up |
| XDH | 840.094585 | 0.522098927 | 0.127811251 | 4.084921505 | 4.41E-05 | 0.000810486 | up |
| CARNS1 | 35.05175109 | 0.527463835 | 0.129134489 | 4.084608522 | 4.42E-05 | 0.000810796 | up |
| ST6GALNAC2 | 199.1882413 | 0.527789552 | 0.129224329 | 4.08428935 | 4.42E-05 | 0.000811466 | up |
| PTPRO | 2033.828399 | -0.645897622 | 0.158151649 | -4.084039752 | 4.43E-05 | 0.000811893 | down |
| AL844908.2 | 2.417197838 | 0.672385934 | 0.164662421 | 4.083420667 | 4.44E-05 | 0.000813568 | up |
| RNF148 | 3.522954089 | 0.620549213 | 0.152058142 | 4.080999571 | 4.48E-05 | 0.000820664 | up |
| ABCG5 | 28.18455767 | 0.661909808 | 0.162196739 | 4.080906999 | 4.49E-05 | 0.000820664 | up |
| RNU5F-1 | 1.212751602 | -1.084332894 | 0.265762199 | -4.080087008 | 4.50E-05 | 0.000821766 | down |
| SECTM1 | 875.2527742 | 0.535758165 | 0.131331344 | 4.079438678 | 4.51E-05 | 0.000823192 | up |
| KRT16P2 | 0.809391922 | 1.341194671 | 0.328770092 | 4.079430289 | 4.51E-05 | 0.000823192 | up |
| EFCAB12 | 8.694449209 | 0.520878499 | 0.127707395 | 4.078687048 | 4.53E-05 | 0.000824821 | up |
| EGFR-AS1 | 12.09289502 | -0.939488778 | 0.230346427 | -4.078590618 | 4.53E-05 | 0.000824821 | down |
| GAS2 | 82.00888443 | -0.521889675 | 0.12797061 | -4.07819948 | 4.54E-05 | 0.00082576 | down |
| AL391883.1 | 4.204299151 | -0.790942156 | 0.194001207 | -4.076996056 | 4.56E-05 | 0.000828241 | down |
| AC020663.2 | 30.48414385 | -0.516328106 | 0.126670617 | -4.076147388 | 4.58E-05 | 0.000829917 | down |
| AC090515.4 | 1.392221246 | -1.019993695 | 0.25024319 | -4.076009798 | 4.58E-05 | 0.000829958 | down |
| B3GAT1 | 19.97061028 | 0.588625709 | 0.144443796 | 4.075119344 | 4.60E-05 | 0.000832689 | up |
| BNIP3P1 | 3.194304877 | -0.92133349 | 0.226228906 | -4.072571921 | 4.65E-05 | 0.000840487 | down |
| PEX5L | 3.157181814 | 0.67820697 | 0.166595196 | 4.070987563 | 4.68E-05 | 0.000845311 | up |
| AC098848.1 | 0.446969277 | 0.971903269 | 0.238848373 | 4.069122418 | 4.72E-05 | 0.000849811 | up |
| GPX1P1 | 103.9750542 | -0.693094126 | 0.170364991 | -4.068289624 | 4.74E-05 | 0.000852394 | down |
| AL355512.1 | 1.181663049 | 1.038497955 | 0.255320826 | 4.067423611 | 4.75E-05 | 0.000854647 | up |
| Z94160.1 | 1.43762114 | 1.436536333 | 0.35342151 | 4.064654502 | 4.81E-05 | 0.000863465 | up |
| AC091544.6 | 0.912361287 | 0.940615504 | 0.231428833 | 4.064383388 | 4.82E-05 | 0.000863813 | up |
| LINC02544 | 5.187692438 | -0.901656363 | 0.222029678 | -4.060972264 | 4.89E-05 | 0.000874851 | down |
| TRMT112P6 | 12.33622046 | -0.647509882 | 0.15945856 | -4.060678099 | 4.89E-05 | 0.000875485 | down |
| CEL | 1412.886395 | -0.941903 | 0.231998246 | -4.059957425 | 4.91E-05 | 0.000877722 | down |
| ST8SIA5 | 4.471450481 | 0.724400268 | 0.178455984 | 4.059265782 | 4.92E-05 | 0.000879855 | up |
| WFDC12 | 2.646592322 | 0.742704742 | 0.183057364 | 4.057224069 | 4.97E-05 | 0.000885214 | up |
| CCDC169 | 4.957452808 | -0.984850852 | 0.242771406 | -4.056700366 | 4.98E-05 | 0.000886255 | down |
| KLRC2 | 11.25613671 | 0.726115438 | 0.17900519 | 4.056393208 | 4.98E-05 | 0.000886949 | up |
| AC004687.1 | 57.13946336 | 0.616084001 | 0.151888739 | 4.056153239 | 4.99E-05 | 0.000887388 | up |
| GUCY1A2 | 94.23959213 | 0.562804143 | 0.138804276 | 4.054659984 | 5.02E-05 | 0.000890234 | up |
| UST | 107.4311991 | 0.617170267 | 0.152262256 | 4.053337198 | 5.05E-05 | 0.000893863 | up |
| DKK4 | 96.44572425 | 0.923271413 | 0.227929969 | 4.050680208 | 5.11E-05 | 0.000902641 | up |
| DUSP5P1 | 5.05530766 | 0.774077839 | 0.191127241 | 4.050065471 | 5.12E-05 | 0.000904538 | up |
| LINC00702 | 24.71313412 | -0.641258464 | 0.158340445 | -4.049871553 | 5.12E-05 | 0.00090481 | down |
| PTPRD-AS1 | 66.36870733 | -0.666143395 | 0.164563308 | -4.047946072 | 5.17E-05 | 0.000911802 | down |
| GLP1R | 1.449835194 | -1.090788358 | 0.269505816 | -4.047364819 | 5.18E-05 | 0.000913151 | down |
| AL512329.2 | 5.110513524 | -0.94198749 | 0.232779634 | -4.046692023 | 5.19E-05 | 0.000915249 | down |
| SPINK2 | 7.187805714 | 0.760632702 | 0.18799819 | 4.045957578 | 5.21E-05 | 0.000916958 | up |
| WT1-AS | 6.01597165 | -1.253418358 | 0.3100378 | -4.042792068 | 5.28E-05 | 0.000927193 | down |
| AL022718.1 | 53.5535731 | -1.11703096 | 0.27639112 | -4.041486429 | 5.31E-05 | 0.000931393 | down |
| GS1-594A7.3 | 1.097072979 | -1.454607448 | 0.359973654 | -4.040871965 | 5.33E-05 | 0.000932673 | down |
| ANO1 | 1325.482604 | 0.532343556 | 0.131744054 | 4.040740658 | 5.33E-05 | 0.000932673 | up |
| IGHEP2 | 1.465231718 | 0.912036118 | 0.22571391 | 4.040673081 | 5.33E-05 | 0.000932673 | up |
| SPRR2A | 25.30273542 | 1.275531144 | 0.316015161 | 4.036297306 | 5.43E-05 | 0.000947752 | up |
| TMEM61 | 51.60506089 | 0.635505386 | 0.157483824 | 4.035369291 | 5.45E-05 | 0.000950514 | up |
| LINC01594 | 13.84554665 | 0.701256598 | 0.173873624 | 4.033139594 | 5.50E-05 | 0.000958582 | up |
| AC087623.2 | 2.423186079 | 0.671835982 | 0.166594732 | 4.032756464 | 5.51E-05 | 0.000959647 | up |
| KARSP2 | 2.14207109 | -0.849914658 | 0.210767119 | -4.032482223 | 5.52E-05 | 0.000959769 | down |
| AL161669.1 | 5.812237508 | 0.578611347 | 0.14349524 | 4.032268586 | 5.52E-05 | 0.000960143 | up |
| GJC3 | 22.56452495 | -0.534839049 | 0.132690271 | -4.03073297 | 5.56E-05 | 0.000965936 | down |
| RNU4-47P | 2.240375853 | 0.536163015 | 0.133097237 | 4.028355709 | 5.62E-05 | 0.00097373 | up |
| KLK6 | 966.8450547 | 0.858350158 | 0.213123573 | 4.02747638 | 5.64E-05 | 0.000976871 | up |
| VSIG1 | 38.48486084 | -0.789631693 | 0.196123369 | -4.02619891 | 5.67E-05 | 0.000981606 | down |
| MTND4P12 | 1449.365183 | 0.810178393 | 0.201294287 | 4.024845442 | 5.70E-05 | 0.000985813 | up |
| SLC16A7 | 103.8191842 | 0.619933481 | 0.154101126 | 4.022900391 | 5.75E-05 | 0.000991942 | up |
| LRRC43 | 25.05485109 | -0.52877013 | 0.131469062 | -4.022011895 | 5.77E-05 | 0.000994153 | down |
| AC127071.1 | 3.309096856 | 1.136697455 | 0.282935557 | 4.017513629 | 5.88E-05 | 0.001011232 | up |
| GLP2R | 27.3240258 | -0.551611479 | 0.137321874 | -4.016923612 | 5.90E-05 | 0.001013246 | down |
| FOXC2-AS1 | 1.068101407 | -1.639867336 | 0.40848908 | -4.014470436 | 5.96E-05 | 0.001020196 | down |
| SDK2 | 100.0057583 | 0.607269102 | 0.151270253 | 4.01446478 | 5.96E-05 | 0.001020196 | up |
| RAET1K | 9.123432753 | 0.543919448 | 0.135525998 | 4.013395619 | 5.99E-05 | 0.001024306 | up |
| RPL35P2 | 70.1893057 | -0.769349473 | 0.191730165 | -4.012667875 | 6.00E-05 | 0.001025895 | down |
| AK5 | 16.74889275 | -0.549910434 | 0.137093631 | -4.011203366 | 6.04E-05 | 0.00102997 | down |
| AC141930.1 | 6.564465271 | 0.886913882 | 0.221164656 | 4.010197188 | 6.07E-05 | 0.00103352 | up |
| AC007384.1 | 10.29900731 | 0.567276249 | 0.141468743 | 4.009905216 | 6.07E-05 | 0.001034272 | up |
| KRT9 | 1.747972367 | 1.048445109 | 0.261601337 | 4.007797217 | 6.13E-05 | 0.001040766 | up |
| DKK1 | 68.59990739 | 0.952692643 | 0.237711559 | 4.007767436 | 6.13E-05 | 0.001040766 | up |
| AC025538.1 | 0.897170713 | -0.876152796 | 0.218679611 | -4.006559152 | 6.16E-05 | 0.001045302 | down |
| GPR171 | 34.71569972 | 0.503324701 | 0.125665171 | 4.005284018 | 6.19E-05 | 0.001050049 | up |
| AP000851.2 | 1.457885866 | -1.323028562 | 0.330323713 | -4.005248522 | 6.20E-05 | 0.001050049 | down |
| HAR1B | 14.12382926 | 0.792576796 | 0.197936059 | 4.004206213 | 6.22E-05 | 0.001054155 | up |
| SCARNA10 | 2.898320115 | -0.771088783 | 0.192624615 | -4.003064637 | 6.25E-05 | 0.00105872 | down |
| FBXO27 | 25.81040673 | -0.566976262 | 0.141664325 | -4.002251522 | 6.27E-05 | 0.001061829 | down |
| MEGF11 | 27.9098637 | -0.649215092 | 0.162290198 | -4.000334587 | 6.33E-05 | 0.001068844 | down |
| KLK14 | 16.54385037 | 0.737378108 | 0.184345673 | 3.999975139 | 6.33E-05 | 0.001069929 | up |
| MS4A8 | 280.9817685 | 0.645300622 | 0.161355015 | 3.999259787 | 6.35E-05 | 0.001072084 | up |
| NKX2-1 | 24.74974172 | -1.692175733 | 0.423138456 | -3.999106465 | 6.36E-05 | 0.001072238 | down |
| AL080312.2 | 1.717537069 | -0.876768103 | 0.219360113 | -3.996934952 | 6.42E-05 | 0.001081027 | down |
| LINC01558 | 103.4756817 | -0.543590507 | 0.136091225 | -3.994309738 | 6.49E-05 | 0.001091974 | down |
| FLJ16779 | 25.88356286 | -0.772799992 | 0.193526441 | -3.993252748 | 6.52E-05 | 0.001096304 | down |
| AP005233.2 | 30.42163914 | 0.630604036 | 0.158027945 | 3.990459008 | 6.59E-05 | 0.001108741 | up |
| AL357060.1 | 5.161427605 | 0.780627775 | 0.195641939 | 3.990084025 | 6.60E-05 | 0.001109128 | up |
| KRT40 | 85.22756998 | -1.093035567 | 0.274082649 | -3.987977966 | 6.66E-05 | 0.001116468 | down |
| NTN5 | 9.761117145 | 0.562474028 | 0.141068476 | 3.98724113 | 6.68E-05 | 0.00111844 | up |
| AC106037.1 | 3.866962118 | 0.5127461 | 0.128608741 | 3.986868205 | 6.70E-05 | 0.001119335 | up |
| AC005324.3 | 3.240842874 | 0.622175257 | 0.15605977 | 3.986775427 | 6.70E-05 | 0.001119335 | up |
| AC103810.2 | 3.222545912 | -0.65356236 | 0.164235429 | -3.97942371 | 6.91E-05 | 0.001150498 | down |
| AC254629.1 | 319.8897938 | -0.500925305 | 0.125878896 | -3.979422447 | 6.91E-05 | 0.001150498 | down |
| ACSS3 | 71.65549454 | -0.53057844 | 0.133352947 | -3.978753004 | 6.93E-05 | 0.001153168 | down |
| CYP2T3P | 12.51912857 | -0.528471011 | 0.132829748 | -3.978559154 | 6.93E-05 | 0.001153175 | down |
| AL645608.9 | 1.933743423 | 0.84934992 | 0.213611122 | 3.976150266 | 7.00E-05 | 0.001162961 | up |
| AC010422.3 | 109.0577878 | -0.739092231 | 0.18595729 | -3.974526798 | 7.05E-05 | 0.00116978 | down |
| AC093382.1 | 1.115240478 | 0.645998195 | 0.162534779 | 3.974522866 | 7.05E-05 | 0.00116978 | up |
| TEX101 | 5.756885596 | 0.82861601 | 0.208532461 | 3.973558866 | 7.08E-05 | 0.001173944 | up |
| MPV17L | 513.0378891 | -0.701417274 | 0.176571807 | -3.972419425 | 7.11E-05 | 0.001178991 | down |
| AP000902.1 | 7.521677588 | -1.124593495 | 0.283163472 | -3.971534482 | 7.14E-05 | 0.001181553 | down |
| C11orf91 | 2.421993704 | 0.627292957 | 0.157951375 | 3.971430811 | 7.14E-05 | 0.001181553 | up |
| MYADML2 | 87.1496465 | -0.65388943 | 0.164711506 | -3.969907416 | 7.19E-05 | 0.001187371 | down |
| AL035458.2 | 11.31070867 | -0.506564921 | 0.127624784 | -3.969173587 | 7.21E-05 | 0.001190355 | down |
| AL158839.1 | 2.610614815 | 0.692112526 | 0.174376316 | 3.96907413 | 7.22E-05 | 0.001190355 | up |
| SLC28A3 | 297.4029412 | 0.647342483 | 0.163105242 | 3.968863761 | 7.22E-05 | 0.001190819 | up |
| SNORA60 | 8.440600826 | -0.503076248 | 0.126769634 | -3.968428648 | 7.23E-05 | 0.001192407 | down |
| SLC39A5 | 2730.909848 | -0.522464537 | 0.131684728 | -3.967540856 | 7.26E-05 | 0.001195848 | down |
| SRL | 22.30306758 | 0.519787715 | 0.131133993 | 3.963790799 | 7.38E-05 | 0.001211055 | up |
| AC008543.4 | 2.106735988 | 0.575378558 | 0.145204208 | 3.962547401 | 7.42E-05 | 0.001216181 | up |
| AC023090.1 | 4.633862835 | 0.830329595 | 0.209629651 | 3.960935816 | 7.47E-05 | 0.00122322 | up |
| VIPR2 | 21.18652391 | -0.570939157 | 0.144157901 | -3.96051241 | 7.48E-05 | 0.001224191 | down |
| ABCG8 | 19.76811295 | 0.812554467 | 0.205316081 | 3.957578309 | 7.57E-05 | 0.001235693 | up |
| HABP2 | 65.21536976 | 1.091045034 | 0.275739243 | 3.956799986 | 7.60E-05 | 0.001237912 | up |
| AC012363.1 | 6.704741588 | 0.692449935 | 0.175125193 | 3.954028101 | 7.68E-05 | 0.001249912 | up |
| AC135782.1 | 5.860200089 | 0.621619245 | 0.157233521 | 3.95347785 | 7.70E-05 | 0.001252183 | up |
| AL589993.1 | 1.143589421 | 0.747628759 | 0.189183004 | 3.951881217 | 7.75E-05 | 0.001259344 | up |
| CNPY1 | 1.165200237 | 1.313858413 | 0.332600708 | 3.950257416 | 7.81E-05 | 0.001267303 | up |
| RERG | 101.4665254 | -0.52842763 | 0.133820069 | -3.948792081 | 7.85E-05 | 0.001274466 | down |
| CAB39L | 814.668678 | -0.502242046 | 0.127225785 | -3.947643533 | 7.89E-05 | 0.001279973 | down |
| HIST1H2BE | 4.412054195 | -0.7134388 | 0.180732269 | -3.947489856 | 7.90E-05 | 0.001280174 | down |
| AC018755.3 | 13.24320032 | -0.631508539 | 0.159990239 | -3.94716917 | 7.91E-05 | 0.001280925 | down |
| ID1 | 5527.680268 | -0.533337469 | 0.135163802 | -3.945860224 | 7.95E-05 | 0.001286598 | down |
| BTBD17 | 1.521178239 | 0.985463248 | 0.249778546 | 3.945347846 | 7.97E-05 | 0.001287926 | up |
| GPR55 | 32.89503409 | 0.627558222 | 0.159114614 | 3.944064019 | 8.01E-05 | 0.001293594 | up |
| HOXB13-AS1_2 | 1.197030075 | -0.868608251 | 0.220328666 | -3.942329739 | 8.07E-05 | 0.00130173 | down |
| ATP5G1P6 | 1.978457193 | 0.753376631 | 0.191210527 | 3.940037411 | 8.15E-05 | 0.001311069 | up |
| ADCY2 | 40.37288982 | -0.505603706 | 0.12834347 | -3.939457977 | 8.17E-05 | 0.001313607 | down |
| AL831784.1 | 0.627447099 | -1.528165075 | 0.387960556 | -3.938970219 | 8.18E-05 | 0.001315647 | down |
| NEUROD2 | 2.082694113 | 0.772531655 | 0.196155635 | 3.93836075 | 8.20E-05 | 0.001317094 | up |
| LINGO1 | 206.9482679 | 0.533993974 | 0.135608038 | 3.937775234 | 8.22E-05 | 0.001319677 | up |
| LY6G6F | 7.873510166 | -0.771548642 | 0.195956038 | -3.937355803 | 8.24E-05 | 0.001321351 | down |
| AC090498.1 | 342.2585554 | 0.525977592 | 0.133597477 | 3.937032389 | 8.25E-05 | 0.001322498 | up |
| EYA2 | 114.4906153 | -0.66640304 | 0.16933522 | -3.935407165 | 8.31E-05 | 0.001329568 | down |
| AC091839.1 | 4.210189795 | -1.382989885 | 0.351512415 | -3.9343984 | 8.34E-05 | 0.001334525 | down |
| SNORA63C | 0.683838628 | -1.624440427 | 0.412999522 | -3.933274349 | 8.38E-05 | 0.001339034 | down |
| CCR9 | 6.188121161 | 0.637629221 | 0.162115104 | 3.933188247 | 8.38E-05 | 0.001339034 | up |
| KRT23 | 2317.85221 | -0.81672074 | 0.207681393 | -3.932565785 | 8.40E-05 | 0.001340531 | down |
| AL603839.4 | 2.372669518 | 0.521655594 | 0.132651302 | 3.932532797 | 8.41E-05 | 0.001340531 | up |
| SNORA38B | 1.228876591 | -0.92397714 | 0.234971934 | -3.932287251 | 8.41E-05 | 0.001340531 | down |
| ABCF2P1 | 2.727698436 | -0.9529065 | 0.242414685 | -3.930894278 | 8.46E-05 | 0.00134768 | down |
| ONECUT2 | 182.6443976 | 0.590787579 | 0.150328671 | 3.929972735 | 8.50E-05 | 0.001350926 | up |
| LINC01229 | 1.397302009 | 0.845360081 | 0.215203695 | 3.92818571 | 8.56E-05 | 0.001359062 | up |
| OVOL3 | 4.000745202 | 0.589111244 | 0.150003133 | 3.927326273 | 8.59E-05 | 0.001362632 | up |
| NRIR | 3.66279508 | 0.69480747 | 0.17693502 | 3.926907584 | 8.60E-05 | 0.001364358 | up |
| GMPR | 410.8001364 | 0.505061999 | 0.128645816 | 3.92598853 | 8.64E-05 | 0.001368704 | up |
| MTND4P24 | 23.11137991 | 0.986243648 | 0.251228779 | 3.925679422 | 8.65E-05 | 0.001369391 | up |
| SMIM1 | 29.19220032 | -0.516961711 | 0.131700701 | -3.925276831 | 8.66E-05 | 0.001370449 | down |
| CXCL11 | 595.740372 | 0.668121608 | 0.170220713 | 3.925031197 | 8.67E-05 | 0.001371136 | up |
| LYZ | 19773.51572 | 0.688587259 | 0.175451753 | 3.924653054 | 8.69E-05 | 0.001371602 | up |
| AC084864.1 | 2.204428756 | 0.635134971 | 0.161833982 | 3.924608201 | 8.69E-05 | 0.001371602 | up |
| GRIP2 | 80.58909233 | 0.572360903 | 0.145881114 | 3.923474994 | 8.73E-05 | 0.001375002 | up |
| AFP | 12.16763799 | -0.934315914 | 0.238220212 | -3.922068183 | 8.78E-05 | 0.00138158 | down |
| GPR25 | 13.64876331 | 0.696649514 | 0.177707048 | 3.920213184 | 8.85E-05 | 0.001388338 | up |
| LRRC15 | 503.6112371 | -0.554438928 | 0.141483183 | -3.918762045 | 8.90E-05 | 0.001395412 | down |
| SCG5 | 233.5072149 | 0.546643449 | 0.139546275 | 3.917291587 | 8.95E-05 | 0.001402633 | up |
| SOX2 | 114.5443864 | 1.182801711 | 0.301996371 | 3.916609021 | 8.98E-05 | 0.001404231 | up |
| GUCY2GP | 7.799139585 | -0.53442411 | 0.136564271 | -3.913352347 | 9.10E-05 | 0.001420401 | down |
| LINC00520 | 17.23154992 | 0.788954323 | 0.201719564 | 3.911144311 | 9.19E-05 | 0.001430114 | up |
| PPP1R3C | 71.36863166 | -0.5295392 | 0.135476501 | -3.908716244 | 9.28E-05 | 0.001442547 | down |
| TRGC2 | 22.13480758 | 0.547908981 | 0.140210826 | 3.907750885 | 9.32E-05 | 0.001446976 | up |
| AC016831.6 | 1.569846221 | 1.382506167 | 0.353821542 | 3.907354419 | 9.33E-05 | 0.001448679 | up |
| AC009163.6 | 3.986803964 | 0.941468921 | 0.241256425 | 3.902357915 | 9.53E-05 | 0.00147413 | up |
| AC023510.2 | 1.61076118 | 0.781073768 | 0.200209043 | 3.901291153 | 9.57E-05 | 0.001479957 | up |
| AC078820.1 | 3.745617574 | 0.760271103 | 0.195008645 | 3.89865332 | 9.67E-05 | 0.001494097 | up |
| AL139020.1 | 0.889586795 | 1.376733029 | 0.3532959 | 3.896827076 | 9.75E-05 | 0.001504301 | up |
| HIST1H2BM | 2.457455834 | -1.065115396 | 0.273332131 | -3.896780779 | 9.75E-05 | 0.001504301 | down |
| LINC02084 | 4.661476306 | 0.612025002 | 0.157158725 | 3.894311312 | 9.85E-05 | 0.001517607 | up |
| CAPN13 | 263.8471783 | -0.568732162 | 0.146101702 | -3.892714133 | 9.91E-05 | 0.001525577 | down |
| ROR1 | 91.94124654 | 0.530967691 | 0.136471306 | 3.890691073 | 1.00E-04 | 0.001534772 | up |
| SNORA71C | 12.82137508 | -0.51281832 | 0.131888654 | -3.88826715 | 0.000100962 | 0.001548755 | down |
| AC008687.4 | 1.622821712 | 0.78950407 | 0.203098415 | 3.887298032 | 0.000101366 | 0.001553524 | up |
| GP5 | 5.178667353 | -0.572079424 | 0.147198075 | -3.886459969 | 0.000101717 | 0.00155818 | down |
| PNLIPRP2 | 93.05440288 | -0.826807943 | 0.212825527 | -3.884909643 | 0.000102368 | 0.001566005 | down |
| MESTP1 | 3.461581677 | -0.577194319 | 0.148608608 | -3.883989804 | 0.000102756 | 0.001570508 | down |
| UTF1 | 0.795896735 | -1.317240412 | 0.339183241 | -3.883565731 | 0.000102936 | 0.001572305 | down |
| FAM66B | 7.468434584 | -0.537358293 | 0.13837949 | -3.883222089 | 0.000103081 | 0.001572602 | down |
| PKIA | 123.5134005 | -0.639630195 | 0.164751743 | -3.882388034 | 0.000103436 | 0.00157585 | down |
| IL17C | 15.39153928 | 0.781004126 | 0.201287995 | 3.880033315 | 0.000104442 | 0.001589013 | up |
| RNU6ATAC35P | 2.463647901 | 0.510590324 | 0.131601844 | 3.879811307 | 0.000104538 | 0.001589018 | up |
| AP001357.2 | 2.008327145 | -0.926162612 | 0.238785905 | -3.878631834 | 0.000105046 | 0.001595135 | down |
| MTRNR2L12 | 56.35364427 | -0.537194107 | 0.138510774 | -3.878356125 | 0.000105165 | 0.00159565 | down |
| BX248409.1 | 9.803115763 | -0.707363312 | 0.182415878 | -3.877750776 | 0.000105427 | 0.001598899 | down |
| RNA5SP18 | 2.146361733 | 0.851743388 | 0.219673824 | 3.877309424 | 0.000105618 | 0.00160035 | up |
| U62317.1 | 1.936439897 | 0.812581301 | 0.209602436 | 3.876774121 | 0.000105851 | 0.001603147 | up |
| ARSE | 2573.399174 | -0.500178207 | 0.129039315 | -3.876169113 | 0.000106114 | 0.001606409 | down |
| RHBG | 5.584110678 | 0.763487504 | 0.197006321 | 3.875446746 | 0.000106429 | 0.001609018 | up |
| AC006238.1 | 1.077518547 | 0.79508284 | 0.205273032 | 3.873294173 | 0.000107374 | 0.00161889 | up |
| AC091544.5 | 11.59508765 | 0.808811783 | 0.208829641 | 3.87306983 | 0.000107473 | 0.001619651 | up |
| TDGF1P3 | 5.021898986 | -0.748716063 | 0.19335653 | -3.872204703 | 0.000107855 | 0.00162468 | down |
| AC138123.1 | 121.1606696 | 0.535904453 | 0.138411727 | 3.871813931 | 0.000108028 | 0.001626555 | up |
| LRRC10B | 99.20421308 | -0.533586339 | 0.137885864 | -3.869768253 | 0.000108939 | 0.001638051 | down |
| HRASLS5 | 23.36365063 | 0.822813524 | 0.212647107 | 3.869384985 | 0.00010911 | 0.001639153 | up |
| MIR7-3HG | 1.059046151 | 1.248447102 | 0.322671721 | 3.869093631 | 0.000109241 | 0.001640376 | up |
| BNIP3 | 520.14065 | -0.523059971 | 0.135255918 | -3.867187335 | 0.000110098 | 0.001650283 | down |
| AC002525.1 | 3.409651591 | 0.694181342 | 0.179661392 | 3.863831484 | 0.000111622 | 0.001670886 | up |
| GALR2 | 9.18546963 | -0.546447791 | 0.141431906 | -3.863681161 | 0.000111691 | 0.001671167 | down |
| NLRP9 | 2.77172977 | 0.814395438 | 0.210824873 | 3.86290017 | 0.000112049 | 0.001675771 | up |
| AC022415.1 | 9.971892546 | -0.503137358 | 0.130285153 | -3.861816538 | 0.000112547 | 0.001682471 | down |
| TDGF1 | 963.1713246 | -0.557865711 | 0.144480719 | -3.861177562 | 0.000112842 | 0.001685371 | down |
| MAFA | 2.748286751 | 0.659059516 | 0.170701105 | 3.860897771 | 0.000112971 | 0.001686549 | up |
| CPE | 1120.816305 | -0.550115583 | 0.142554252 | -3.858991052 | 0.000113856 | 0.00169673 | down |
| AP001626.1 | 2.072882702 | 0.868775815 | 0.225149685 | 3.858658809 | 0.000114011 | 0.001698281 | up |
| ABCA3 | 451.2408882 | 0.613214673 | 0.159021224 | 3.856181309 | 0.000115172 | 0.00171405 | up |
| AC127070.3 | 7.015551909 | -0.567789475 | 0.147262233 | -3.855635371 | 0.000115429 | 0.001717116 | down |
| C2orf66 | 6.441705904 | 0.684804405 | 0.177694737 | 3.853824922 | 0.000116287 | 0.001728331 | up |
| HHIP | 106.4885255 | -0.546746216 | 0.141883981 | -3.853473894 | 0.000116454 | 0.001728897 | down |
| AC023983.2 | 1.617686485 | 0.679646239 | 0.176393693 | 3.8530076 | 0.000116676 | 0.001730268 | up |
| AC007221.1 | 3.133760783 | -0.648709346 | 0.168396073 | -3.852283094 | 0.000117022 | 0.001733859 | down |
| LINC02128 | 0.834048807 | -1.365442456 | 0.354474041 | -3.852023838 | 0.000117146 | 0.00173416 | down |
| RN7SKP127 | 1.196610751 | -1.065330138 | 0.276713532 | -3.849938702 | 0.000118147 | 0.001747711 | down |
| RN7SL664P | 0.981271777 | -1.47125274 | 0.382272005 | -3.848706471 | 0.000118743 | 0.001754702 | down |
| CPNE4 | 8.605292454 | 0.935410717 | 0.243073498 | 3.848262867 | 0.000118958 | 0.001756952 | up |
| MPO | 12.42462707 | -0.558827211 | 0.145243214 | -3.847527165 | 0.000119316 | 0.001758505 | down |
| AC006272.2 | 2.394692549 | 0.633472691 | 0.164676333 | 3.846774329 | 0.000119683 | 0.001762361 | up |
| RNU2-63P | 0.933685313 | -0.981513951 | 0.255187851 | -3.846240907 | 0.000119944 | 0.001764647 | down |
| NOS1 | 18.60304351 | -0.858732498 | 0.223299229 | -3.845658139 | 0.000120229 | 0.001766588 | down |
| PI3 | 3195.733132 | 0.715716999 | 0.186141021 | 3.84502564 | 0.00012054 | 0.00176797 | up |
| CTTNBP2 | 390.1303616 | -0.523078852 | 0.136053586 | -3.844653175 | 0.000120723 | 0.001769882 | down |
| AC008875.1 | 3.160903128 | 0.513442116 | 0.133689248 | 3.840564033 | 0.000122752 | 0.001796476 | up |
| IGFBP7-AS1 | 12.48752579 | -0.849413922 | 0.221220691 | -3.839667605 | 0.000123201 | 0.001800741 | down |
| ZNF730 | 7.672332327 | -0.870874083 | 0.226814992 | -3.839579015 | 0.000123245 | 0.001800741 | down |
| RARRES1 | 967.0227276 | 0.586115485 | 0.152652028 | 3.839552551 | 0.000123259 | 0.001800741 | up |
| OR7E22P | 4.778462343 | 0.916677446 | 0.238912255 | 3.836879134 | 0.000124608 | 0.001817273 | up |
| OR2B6 | 2.77077647 | 0.684780279 | 0.178498264 | 3.836341395 | 0.000124881 | 0.001820361 | up |
| AC007040.2 | 1.700543193 | 0.824867872 | 0.215019439 | 3.836247898 | 0.000124928 | 0.001820361 | up |
| AC011511.5 | 4.094420338 | 0.501117468 | 0.130633812 | 3.836047187 | 0.00012503 | 0.001821055 | up |
| ADTRP | 509.5926542 | 0.654677478 | 0.170680378 | 3.835692698 | 0.000125211 | 0.001822169 | up |
| AC108725.1 | 1.939390694 | -1.079839197 | 0.281524629 | -3.835682867 | 0.000125216 | 0.001822169 | down |
| SND1-IT1 | 1.683104338 | -1.009460933 | 0.263461677 | -3.831528534 | 0.00012735 | 0.001847287 | down |
| AC148477.4 | 7.915784907 | 0.960717758 | 0.250768531 | 3.831093773 | 0.000127575 | 0.001849255 | up |
| FGL1 | 4.610096712 | 1.216619564 | 0.317747803 | 3.828884264 | 0.000128726 | 0.001861094 | up |
| AP003385.3 | 46.49878992 | -0.576508515 | 0.150603682 | -3.827984188 | 0.000129197 | 0.001866297 | down |
| RNA5SP202 | 2.898532266 | -2.214102943 | 0.578537134 | -3.82707144 | 0.000129677 | 0.001870804 | down |
| POPDC3 | 12.7578044 | 0.722299294 | 0.188754036 | 3.826669413 | 0.000129889 | 0.001872516 | up |
| HIF3A | 76.84543233 | -0.595166328 | 0.155533869 | -3.826602729 | 0.000129924 | 0.001872516 | down |
| CACNG4 | 158.9823658 | -0.943175728 | 0.246483441 | -3.826527758 | 0.000129964 | 0.001872516 | down |
| SNORA20 | 0.608343143 | -1.749072724 | 0.457254382 | -3.825163395 | 0.000130685 | 0.001878062 | down |
| NCOA4P3 | 2.979930627 | -0.71452551 | 0.186836021 | -3.824345582 | 0.00013112 | 0.001881453 | down |
| CASC21 | 65.60423586 | -0.632130961 | 0.165302456 | -3.824086929 | 0.000131258 | 0.001881453 | down |
| TNNT1 | 166.3644651 | 0.861045958 | 0.225236073 | 3.822859928 | 0.000131913 | 0.001887588 | up |
| RAMP2-AS1 | 30.50556129 | -0.555474871 | 0.145454132 | -3.818900583 | 0.000134048 | 0.001914044 | down |
| AL845472.1 | 6.278011933 | -0.666246533 | 0.174570649 | -3.816486533 | 0.000135365 | 0.001932034 | down |
| HNRNPA1P25 | 1.629573205 | 0.693099674 | 0.181680493 | 3.814937221 | 0.000136218 | 0.001940883 | up |
| AC105235.1 | 6.040441751 | -0.815865867 | 0.213981876 | -3.812780227 | 0.000137412 | 0.001955807 | down |
| IGKV2D-40 | 38.08438958 | 0.945415364 | 0.248081965 | 3.810899205 | 0.000138462 | 0.001965329 | up |
| CBSL | 3.317205744 | -1.015129997 | 0.266411158 | -3.810388447 | 0.000138749 | 0.001967724 | down |
| AC007182.1 | 6.367993772 | 0.775128879 | 0.203465769 | 3.809627931 | 0.000139176 | 0.001972949 | up |
| SLC15A1 | 144.0431026 | -0.808955927 | 0.212412508 | -3.808419445 | 0.000139858 | 0.001979258 | down |
| ANKRD18B | 8.795437029 | -0.958941701 | 0.251948018 | -3.806109324 | 0.00014117 | 0.001995294 | down |
| LINC01096 | 6.948257 | -0.592611341 | 0.155717433 | -3.805683977 | 0.000141413 | 0.001997882 | down |
| RTP5 | 1.768100781 | 0.863568865 | 0.22694144 | 3.805249779 | 0.000141661 | 0.001999701 | up |
| OPRD1 | 47.49558352 | 0.805647622 | 0.211791204 | 3.803971105 | 0.000142395 | 0.002008361 | up |
| CELP | 193.403585 | -0.763527102 | 0.20076079 | -3.803168437 | 0.000142857 | 0.002012652 | down |
| IGLV1-41 | 31.26546027 | -0.966862864 | 0.254314863 | -3.801833886 | 0.000143629 | 0.002021506 | down |
| POU5F1B | 276.1243099 | -0.567762898 | 0.149349918 | -3.801561507 | 0.000143787 | 0.002022878 | down |
| AC005391.1 | 1.938386957 | -0.73712673 | 0.193918068 | -3.801227683 | 0.000143981 | 0.002024754 | down |
| AC105254.2 | 0.996381612 | -1.093210626 | 0.287810918 | -3.79836398 | 0.000145654 | 0.002045706 | down |
| SLC30A2 | 124.319894 | -0.807874054 | 0.213168409 | -3.789839495 | 0.000150745 | 0.002107463 | down |
| AC005858.1 | 11.05081238 | 1.168285845 | 0.308326538 | 3.789118682 | 0.000151183 | 0.002110939 | up |
| CRISP3 | 0.679314163 | 1.766386011 | 0.466203122 | 3.788876408 | 0.00015133 | 0.002112117 | up |
| RBFOX3 | 21.85925814 | -0.663405531 | 0.175197381 | -3.786617851 | 0.000152712 | 0.002128733 | down |
| AC127071.2 | 0.814777373 | 1.4508358 | 0.383166749 | 3.786434508 | 0.000152824 | 0.002129416 | up |
| AC139100.1 | 3.224904554 | 0.606432211 | 0.160215251 | 3.785109127 | 0.000153641 | 0.002138125 | up |
| AC068870.1 | 1.846890978 | 0.615135741 | 0.162530122 | 3.784749158 | 0.000153864 | 0.002139225 | up |
| KCNJ3 | 113.7750991 | 0.789690819 | 0.208655086 | 3.784670847 | 0.000153912 | 0.002139225 | up |
| AC015961.2 | 1.576470691 | 0.652013883 | 0.17230641 | 3.784037312 | 0.000154305 | 0.002142987 | up |
| AC024884.2 | 2.617268636 | 0.563139626 | 0.148836318 | 3.783617008 | 0.000154566 | 0.002144742 | up |
| KRT6C | 4.538892422 | 1.041108022 | 0.275185579 | 3.783294267 | 0.000154766 | 0.002146636 | up |
| LINC00887 | 4.326823811 | 0.650396521 | 0.172031224 | 3.780688789 | 0.000156395 | 0.00216743 | up |
| LINC00173 | 16.94824846 | 0.587342658 | 0.155407288 | 3.779376531 | 0.000157222 | 0.002176971 | up |
| AL359976.1 | 2.282671806 | -0.995466964 | 0.263570545 | -3.776852096 | 0.000158823 | 0.002196526 | down |
| SLC38A8 | 1.959122608 | 1.081079906 | 0.286377171 | 3.775021252 | 0.000159994 | 0.002211807 | up |
| AMELX | 11.5466826 | -0.739726235 | 0.196140265 | -3.77141447 | 0.000162325 | 0.002237553 | down |
| BX539320.1 | 6.186436904 | 0.535885579 | 0.142156119 | 3.7696976 | 0.000163445 | 0.002252072 | up |
| HIST1H3J | 5.907622413 | -0.695818364 | 0.184653447 | -3.76823924 | 0.000164403 | 0.002262471 | down |
| AC091132.5 | 3.27895184 | -0.509043663 | 0.135106088 | -3.767732983 | 0.000164737 | 0.00226613 | down |
| AC002463.1 | 0.60807507 | 1.176424913 | 0.312333795 | 3.766562993 | 0.00016551 | 0.0022749 | up |
| AL590683.1 | 2.130012134 | -0.679235824 | 0.180389121 | -3.765392396 | 0.000166288 | 0.002284646 | down |
| NPTXR | 185.0951441 | -0.586796944 | 0.155860879 | -3.764876384 | 0.000166631 | 0.002287489 | down |
| SPON1-AS1 | 0.978582093 | 0.722859059 | 0.192014551 | 3.764605622 | 0.000166812 | 0.00228903 | up |
| IGLVIVOR22-1 | 2.340323196 | -0.818063381 | 0.217481902 | -3.761523941 | 0.000168881 | 0.002313133 | down |
| LINC02251 | 13.89609129 | -0.752914419 | 0.2001697 | -3.761380553 | 0.000168978 | 0.002313133 | down |
| AC096734.1 | 0.820969054 | 0.910164264 | 0.241976652 | 3.761372249 | 0.000168984 | 0.002313133 | up |
| CASP1P2 | 7.161125174 | 0.74632891 | 0.198439111 | 3.760997046 | 0.000169237 | 0.002315658 | up |
| AC138466.1 | 1.98547706 | 0.824635136 | 0.219285762 | 3.760550294 | 0.00016954 | 0.002318124 | up |
| AC010280.2 | 3.784399507 | -0.732910434 | 0.194932446 | -3.759817567 | 0.000170037 | 0.002321215 | down |
| SLC10A2 | 1.053584572 | 1.981573367 | 0.527498556 | 3.756547471 | 0.000172274 | 0.002348553 | up |
| LINC02489 | 8.727154646 | 0.700948898 | 0.186606695 | 3.756290191 | 0.000172451 | 0.00235001 | up |
| ADORA2A-AS1 | 7.724735273 | -0.593066778 | 0.157922023 | -3.755440602 | 0.000173037 | 0.002355164 | down |
| DAB1 | 39.6363183 | -0.646012627 | 0.172022199 | -3.755402682 | 0.000173063 | 0.002355164 | down |
| ADAM32 | 90.97802882 | -0.517410494 | 0.137781307 | -3.755302558 | 0.000173132 | 0.002355164 | down |
| AL357033.2 | 1.629434121 | 1.150627838 | 0.30642802 | 3.754969395 | 0.000173363 | 0.002356675 | up |
| AL356310.1 | 2.451167137 | -0.600010205 | 0.15987448 | -3.753008023 | 0.000174725 | 0.002373269 | down |
| PRG2 | 1.432416515 | 0.751368012 | 0.200274299 | 3.751694621 | 0.000175643 | 0.002383802 | up |
| DYDC2 | 4.322587278 | -1.165777696 | 0.310765746 | -3.751306923 | 0.000175915 | 0.002386523 | down |
| AC027243.1 | 1.487708402 | 0.949416053 | 0.253218601 | 3.749393013 | 0.000177263 | 0.002399013 | up |
| AHSG | 16.24058028 | -1.034064571 | 0.27579552 | -3.749388573 | 0.000177266 | 0.002399013 | down |
| AC068594.1 | 4.857907554 | 0.537409805 | 0.143428457 | 3.746884106 | 0.000179045 | 0.002418189 | up |
| AL136307.1 | 4.158674593 | -0.709974911 | 0.189494909 | -3.746670113 | 0.000179197 | 0.002418361 | down |
| TMEM236 | 275.2065329 | -0.558471839 | 0.149099836 | -3.745623439 | 0.000179946 | 0.002425477 | down |
| SAA2-SAA4 | 19.66328001 | 1.010548342 | 0.269794498 | 3.745622503 | 0.000179947 | 0.002425477 | up |
| FGF17 | 7.395679518 | 0.629623147 | 0.1681903 | 3.743516415 | 0.000181463 | 0.002438046 | up |
| RIIAD1 | 28.46624137 | -0.695706982 | 0.185916653 | -3.742036926 | 0.000182535 | 0.002449495 | down |
| FGGY | 1231.82077 | -0.548010553 | 0.146505451 | -3.74054718 | 0.00018362 | 0.002462085 | down |
| SSTR5-AS1 | 71.63189715 | 0.935337681 | 0.250194522 | 3.73844188 | 0.000185164 | 0.002480801 | up |
| CNTD2 | 209.627951 | 0.552291233 | 0.147785901 | 3.737103666 | 0.000186152 | 0.002489051 | up |
| AL096803.1 | 1.102015932 | 0.819273658 | 0.219318236 | 3.735547365 | 0.000187307 | 0.002502495 | up |
| CLDN15 | 1206.262055 | -0.524314223 | 0.140374426 | -3.735112132 | 0.000187632 | 0.002505826 | down |
| RNU4ATAC | 3.398840599 | -0.770562755 | 0.206342137 | -3.7343936 | 0.000188168 | 0.002511987 | down |
| PLA2G2F | 38.69093814 | 0.957091752 | 0.256434131 | 3.732310314 | 0.000189732 | 0.002530839 | up |
| LGALS14 | 1.775477009 | -1.505613294 | 0.403447199 | -3.731871972 | 0.000190062 | 0.002533227 | down |
| AC005885.1 | 3.016872406 | 1.569351129 | 0.420569816 | 3.731487782 | 0.000190352 | 0.002536083 | up |
| CDKN2B-AS1 | 46.89852951 | -0.7237647 | 0.193982784 | -3.731076972 | 0.000190663 | 0.002539211 | down |
| SULT1E1 | 20.93530379 | 0.883539531 | 0.236973564 | 3.72843078 | 0.000192676 | 0.002563295 | up |
| HCAR1 | 80.09716251 | -0.821469965 | 0.220327897 | -3.728397432 | 0.000192701 | 0.002563295 | down |
| AC016877.3 | 2.88466128 | 0.729576664 | 0.195699598 | 3.728043764 | 0.000192972 | 0.002565348 | up |
| SNORD116-24 | 0.689118944 | -1.171637299 | 0.314306345 | -3.727692157 | 0.000193241 | 0.002567234 | down |
| UNC5B-AS1 | 15.9625067 | 0.699691797 | 0.187705222 | 3.727609653 | 0.000193304 | 0.002567234 | up |
| PGPEP1L | 1.16713378 | 1.283025773 | 0.34437541 | 3.725660241 | 0.000194805 | 0.002584079 | up |
| OR52K3P | 10.03178491 | -0.745499762 | 0.200264802 | -3.722570084 | 0.000197205 | 0.00261301 | down |
| AC078993.1 | 134.7585347 | -0.971029019 | 0.26100215 | -3.720387045 | 0.000198918 | 0.002631332 | down |
| DIO3 | 157.7643498 | -0.608198023 | 0.163624054 | -3.717045323 | 0.000201566 | 0.002662156 | down |
| VRTN | 12.68619583 | 0.845805778 | 0.227568846 | 3.716702844 | 0.00020184 | 0.002664713 | up |
| FRRS1L | 5.799935455 | -0.745501759 | 0.200606765 | -3.716234383 | 0.000202214 | 0.002667049 | down |
| AL022318.1 | 1.724492427 | -0.844708823 | 0.227395154 | -3.714717779 | 0.000203431 | 0.002679371 | down |
| PNPLA1 | 25.82696334 | -0.550931783 | 0.148340958 | -3.713955947 | 0.000204044 | 0.002685931 | down |
| AFF2 | 7.03668492 | 0.630783587 | 0.16984398 | 3.713900176 | 0.000204089 | 0.002685931 | up |
| VAX2 | 7.538969208 | 0.667936512 | 0.179884304 | 3.713145054 | 0.000204699 | 0.002690435 | up |
| KC6 | 8.640968157 | 1.485560342 | 0.400248006 | 3.711599608 | 0.000205954 | 0.002704081 | up |
| IFI44L | 419.6862833 | 0.604547044 | 0.16297726 | 3.709395059 | 0.000207755 | 0.002724524 | up |
| CTXN1 | 143.4987483 | 0.567627774 | 0.153047793 | 3.708826902 | 0.000208222 | 0.002729574 | up |
| AC006058.1 | 5.710523153 | 0.736837672 | 0.198717817 | 3.707959782 | 0.000208936 | 0.002737862 | up |
| PENK | 13.74734879 | -0.957445303 | 0.258270339 | -3.70714387 | 0.00020961 | 0.002739631 | down |
| RN7SKP271 | 1.739306786 | -0.671482209 | 0.18114366 | -3.706904288 | 0.000209808 | 0.002739631 | down |
| SLC5A12 | 14.06057115 | 0.725573072 | 0.195757862 | 3.706482412 | 0.000210158 | 0.002742055 | up |
| LINC01644 | 2.379568013 | 0.908495733 | 0.24537803 | 3.702433073 | 0.000213542 | 0.002776081 | up |
| CFAP57 | 6.003478341 | 0.522309884 | 0.141120284 | 3.701168023 | 0.000214609 | 0.002786005 | up |
| CHRM4 | 9.080448625 | 0.644184452 | 0.174095115 | 3.700186824 | 0.000215441 | 0.002793468 | up |
| RPS26P4 | 1.000348777 | 2.11932948 | 0.572776964 | 3.700095521 | 0.000215518 | 0.002793468 | up |
| CNN1 | 1466.908978 | -0.620587342 | 0.167749086 | -3.699497604 | 0.000216027 | 0.002797889 | down |
| TMEM63C | 261.8112782 | -0.570127839 | 0.154114898 | -3.699368765 | 0.000216136 | 0.002798225 | down |
| AL023803.1 | 3.009566825 | -0.640661664 | 0.173221287 | -3.698515779 | 0.000216864 | 0.002804895 | down |
| WFDC10B | 10.51756414 | -0.570379631 | 0.154227142 | -3.698309031 | 0.000217041 | 0.002805503 | down |
| HOXB-AS2 | 20.82362799 | 0.55881981 | 0.151155137 | 3.696995163 | 0.000218166 | 0.002816875 | up |
| LINC01436 | 27.47273767 | -0.62307716 | 0.168545194 | -3.696795759 | 0.000218338 | 0.002817631 | down |
| AC067747.1 | 0.763780762 | 0.691140081 | 0.186959799 | 3.696730976 | 0.000218394 | 0.002817631 | up |
| GPRC6A | 4.959435106 | 1.100773631 | 0.297932951 | 3.694702544 | 0.000220144 | 0.002836027 | up |
| AC245041.2 | 5.934235266 | 0.901166609 | 0.24403827 | 3.692726591 | 0.000221863 | 0.0028549 | up |
| NCCRP1 | 13.06623235 | -0.653701776 | 0.177033155 | -3.692538691 | 0.000222027 | 0.002855688 | down |
| ATAD3C | 114.5782147 | -0.546815687 | 0.148158044 | -3.690759343 | 0.000223586 | 0.002872426 | down |
| AP001024.1 | 84.4055857 | -0.65960203 | 0.178732467 | -3.690443264 | 0.000223864 | 0.002874894 | down |
| AC027279.2 | 0.986233942 | -0.891603366 | 0.241660934 | -3.689480756 | 0.000224712 | 0.002883577 | down |
| ALB | 19.74402361 | -1.222726458 | 0.331470039 | -3.688799334 | 0.000225315 | 0.002889093 | down |
| AC087742.1 | 7.966266243 | -0.666287363 | 0.180648694 | -3.688304346 | 0.000225754 | 0.002892499 | down |
| KIAA1210 | 1.0360181 | -0.74695788 | 0.202667329 | -3.685635388 | 0.000228133 | 0.002914052 | down |
| PTGER2 | 214.4436466 | 0.505547386 | 0.137256902 | 3.683220148 | 0.000230306 | 0.002936205 | up |
| AQP12B | 8.046808848 | -0.680034245 | 0.184693506 | -3.681960777 | 0.000231447 | 0.002948503 | down |
| AS3MT | 3.051070928 | -0.628178313 | 0.170732563 | -3.679311679 | 0.000233864 | 0.002972503 | down |
| AP003031.1 | 2.256227142 | -0.598085905 | 0.162723528 | -3.675472817 | 0.000237409 | 0.003006136 | down |
| LINC01346 | 1.315067125 | 1.095035076 | 0.2980499 | 3.673999144 | 0.000238784 | 0.003022392 | up |
| BARX1 | 12.64431633 | -0.788073407 | 0.21451695 | -3.673711603 | 0.000239053 | 0.003024652 | down |
| SAA4 | 4.823468636 | 0.945920943 | 0.257518379 | 3.673217209 | 0.000239516 | 0.003029367 | up |
| ZNF625 | 1.934580212 | -0.676084965 | 0.184067944 | -3.673018514 | 0.000239702 | 0.003030578 | down |
| TCTE1 | 2.305681828 | 0.75286184 | 0.205100277 | 3.670701228 | 0.000241886 | 0.003052418 | up |
| IZUMO2 | 14.85088698 | -0.948227326 | 0.25835016 | -3.670318329 | 0.000242249 | 0.003053235 | down |
| AC092718.6 | 1.780290935 | 0.796039463 | 0.216888509 | 3.670270351 | 0.000242294 | 0.003053235 | up |
| SLC4A4 | 402.6097869 | 0.677360601 | 0.184554513 | 3.670246751 | 0.000242316 | 0.003053235 | up |
| AC026992.2 | 2.574671387 | -0.767536642 | 0.20912788 | -3.670178471 | 0.000242381 | 0.003053235 | down |
| AFAP1-AS1 | 466.2397244 | 1.075176878 | 0.292952988 | 3.670134529 | 0.000242423 | 0.003053235 | up |
| AC099805.1 | 1.424056618 | -0.984672353 | 0.268312613 | -3.669869788 | 0.000242674 | 0.003054295 | down |
| AC012317.1 | 7.924731787 | 0.568309342 | 0.154965826 | 3.667320446 | 0.000245106 | 0.003083738 | up |
| CEBPE | 8.737775726 | -0.705784024 | 0.192482385 | -3.666746049 | 0.000245657 | 0.003088346 | down |
| AC009831.4 | 0.674958718 | 0.8012172 | 0.218571376 | 3.66570049 | 0.000246662 | 0.003098663 | up |
| NKX6-3 | 16.59284821 | 1.425584645 | 0.38890909 | 3.665598672 | 0.000246761 | 0.003098732 | up |
| LINC01593 | 3.62224313 | 0.852238004 | 0.232508713 | 3.665402443 | 0.00024695 | 0.003099003 | up |
| PIGUP1 | 1.557272228 | -0.725064074 | 0.197830927 | -3.665069392 | 0.000247272 | 0.003101653 | down |
| AL354710.1 | 6.854779563 | -0.72803947 | 0.198665961 | -3.664641212 | 0.000247686 | 0.003105682 | down |
| IFITM4P | 3.414489955 | 0.682878561 | 0.186384693 | 3.663812457 | 0.000248489 | 0.003114585 | up |
| DIO3OS | 523.5412632 | -0.566269899 | 0.154609369 | -3.662584635 | 0.000249683 | 0.003127212 | down |
| AC004233.2 | 48.70021128 | 0.994126327 | 0.271456365 | 3.662195678 | 0.000250063 | 0.003129815 | up |
| PCDHGB6 | 62.73205253 | -0.570287253 | 0.155723444 | -3.662179809 | 0.000250078 | 0.003129815 | down |
| AC093809.1 | 7.986421887 | 0.740584741 | 0.202294688 | 3.660920356 | 0.000251311 | 0.003140456 | up |
| ACMSD | 2.530636743 | 0.668968939 | 0.182776935 | 3.660029318 | 0.000252186 | 0.003147953 | up |
| AL139100.1 | 63.97795365 | -0.607740603 | 0.16607136 | -3.659514817 | 0.000252693 | 0.003152471 | down |
| AC243967.2 | 8.298087494 | -0.656591863 | 0.179422641 | -3.659470512 | 0.000252737 | 0.003152471 | down |
| TPRXL | 44.92063515 | 0.814106153 | 0.222483074 | 3.659182413 | 0.000253021 | 0.00315484 | up |
| AC005481.1 | 9.521890945 | -0.630071095 | 0.172289097 | -3.657057274 | 0.000255127 | 0.003176365 | down |
| DOC2B | 315.6136475 | 0.554264638 | 0.151636211 | 3.655226107 | 0.000256955 | 0.003193148 | up |
| AIPL1 | 0.761307565 | 1.369819467 | 0.374758715 | 3.655203766 | 0.000256978 | 0.003193148 | up |
| AC010329.1 | 0.880496129 | 1.097433724 | 0.300582404 | 3.651024508 | 0.000261196 | 0.003232667 | up |
| C2CD4C | 36.07352076 | 0.569013312 | 0.155936035 | 3.649017434 | 0.000263245 | 0.003254822 | up |
| CCR3 | 13.42346841 | -0.556492057 | 0.152536103 | -3.64826456 | 0.000264018 | 0.00326155 | down |
| ITGAD | 7.979882141 | 0.555368137 | 0.152257153 | 3.647566802 | 0.000264735 | 0.00326921 | up |
| LINC00158 | 0.91803519 | 0.880716177 | 0.241676137 | 3.644199991 | 0.000268225 | 0.003304976 | up |
| KRT16P6 | 1.037500112 | 1.600321865 | 0.439175369 | 3.643924452 | 0.000268512 | 0.003307173 | up |
| AC026765.2 | 1.395623194 | 0.756576349 | 0.207685276 | 3.642898358 | 0.000269585 | 0.003314413 | up |
| ATP5G2P3 | 0.927837761 | -1.01932966 | 0.279875097 | -3.642087742 | 0.000270436 | 0.003322427 | down |
| C5orf49 | 2.917870736 | -0.68787461 | 0.188926746 | -3.640959385 | 0.000271624 | 0.00333335 | down |
| LINC00954 | 29.28533104 | -0.543512647 | 0.149295185 | -3.640523629 | 0.000272084 | 0.003336548 | down |
| AC007218.1 | 1.349198333 | -0.880596299 | 0.241922641 | -3.639991257 | 0.000272647 | 0.003342228 | down |
| HULC | 106.2783164 | -1.523398524 | 0.418577728 | -3.639463886 | 0.000273206 | 0.003347853 | down |
| AC079145.1 | 5.123193255 | -0.529146489 | 0.145444509 | -3.638133151 | 0.000274621 | 0.003361181 | down |
| COLCA2 | 205.8136837 | 0.510513897 | 0.140340149 | 3.637689569 | 0.000275095 | 0.003364829 | up |
| PTPRD | 1241.831835 | -0.54247207 | 0.14927205 | -3.634116827 | 0.000278934 | 0.003405567 | down |
| HOXC13 | 2.993415019 | 1.433909683 | 0.394680969 | 3.633085441 | 0.000280052 | 0.003417966 | up |
| AP002414.4 | 23.77273092 | -0.608950087 | 0.167832491 | -3.628320613 | 0.000285271 | 0.003468997 | down |
| AC020907.5 | 1.978546965 | -0.702214165 | 0.193561695 | -3.627857075 | 0.000285783 | 0.003473967 | down |
| C17orf78 | 34.30504786 | 1.001894493 | 0.276368398 | 3.625213667 | 0.000288723 | 0.003505873 | up |
| AC100823.1 | 4.056557532 | 1.002387112 | 0.276581844 | 3.624197077 | 0.000289861 | 0.003513311 | up |
| CLVS1 | 6.529719159 | 0.558398577 | 0.154263776 | 3.619764739 | 0.000294871 | 0.003562421 | up |
| AC016205.1 | 42.76828245 | 0.503296304 | 0.139060235 | 3.619268328 | 0.000295437 | 0.003566684 | up |
| LINC00689 | 3.459221944 | -0.729299456 | 0.201547746 | -3.618494724 | 0.000296322 | 0.00357349 | down |
| SULT1C2P1 | 4.448014736 | 0.696332148 | 0.192461731 | 3.618029113 | 0.000296855 | 0.003578633 | up |
| AC104371.1 | 2.259563691 | -0.65526315 | 0.181117521 | -3.617889357 | 0.000297015 | 0.003579275 | down |
| SOSTDC1 | 194.7995484 | -0.640173011 | 0.177008491 | -3.616623182 | 0.000298471 | 0.003592937 | down |
| KCTD16 | 23.67279578 | -0.758024505 | 0.209652459 | -3.615624204 | 0.000299625 | 0.003604227 | down |
| MUC2 | 38168.5824 | 0.789956789 | 0.218522904 | 3.61498394 | 0.000300366 | 0.003611485 | up |
| CA4 | 466.9240743 | -0.896247852 | 0.247984163 | -3.614133427 | 0.000301354 | 0.003618519 | down |
| AP003170.4 | 1.407802453 | -0.613776485 | 0.169858467 | -3.61345828 | 0.00030214 | 0.003625355 | down |
| AL139412.1 | 5.516201941 | -0.529768158 | 0.146616476 | -3.613292121 | 0.000302334 | 0.003626378 | down |
| MASP1 | 93.47977631 | -0.66515039 | 0.184182119 | -3.611373325 | 0.00030458 | 0.00364417 | down |
| BOLA3P3 | 9.570686407 | -0.596569382 | 0.165203757 | -3.611112686 | 0.000304886 | 0.00364653 | down |
| AL078621.2 | 22.65252419 | -0.51865869 | 0.143663895 | -3.610222953 | 0.000305934 | 0.003657753 | down |
| TMEM151A | 28.39419253 | 0.676981778 | 0.187562458 | 3.609367165 | 0.000306945 | 0.003667218 | up |
| RNU2-6P | 3.643975312 | -0.758795694 | 0.210256788 | -3.60889987 | 0.000307498 | 0.003672518 | down |
| GCKR | 6.807827307 | 0.561610069 | 0.15573898 | 3.606098289 | 0.000310835 | 0.003707078 | up |
| LINC02441 | 52.41205439 | -0.567143153 | 0.157304861 | -3.605375878 | 0.000311701 | 0.003714757 | down |
| AC144450.1 | 7.870735113 | 0.77275601 | 0.214438337 | 3.603628076 | 0.000313806 | 0.003734515 | up |
| RAB39A | 13.31275508 | -0.594057532 | 0.164896467 | -3.602609218 | 0.000315039 | 0.003747854 | down |
| LINC01468 | 1.263577518 | 1.416435714 | 0.393463107 | 3.599920013 | 0.000318315 | 0.003777422 | up |
| AL356124.1 | 1.90192353 | -0.665476102 | 0.184931022 | -3.598509838 | 0.000320046 | 0.003793921 | down |
| LHB | 3.359358085 | 0.617933568 | 0.171912192 | 3.594472036 | 0.00032505 | 0.003845065 | up |
| AC116917.1 | 0.975489154 | -1.445383039 | 0.402215193 | -3.593556542 | 0.000326195 | 0.003853155 | down |
| DYNLRB2 | 2.366150775 | -0.64743193 | 0.180173725 | -3.593375954 | 0.000326421 | 0.003854466 | down |
| BRSK2 | 217.8403272 | -0.619827022 | 0.172752013 | -3.58795833 | 0.000333278 | 0.003924343 | down |
| AC022150.3 | 0.567165515 | 1.150238215 | 0.32060132 | 3.587752583 | 0.000333541 | 0.003926058 | up |
| PLCH2 | 208.7492557 | 0.516841887 | 0.144277964 | 3.58226491 | 0.000340628 | 0.003998226 | up |
| NOXO1 | 2.200219246 | -0.978440781 | 0.273285707 | -3.580285231 | 0.000343219 | 0.004022993 | down |
| LINC00487 | 1.803937321 | 0.704242099 | 0.196735151 | 3.579645508 | 0.000344061 | 0.004028123 | up |
| WNT1 | 1.571944358 | 0.767310561 | 0.214480775 | 3.577526061 | 0.000346862 | 0.004051485 | up |
| HS3ST4 | 7.796976829 | -1.084613113 | 0.303325494 | -3.57574004 | 0.000349239 | 0.004074978 | down |
| SLC6A20 | 1046.904433 | 0.586355754 | 0.164054222 | 3.574158279 | 0.000351356 | 0.004098259 | up |
| ABCB11 | 5.654362987 | 0.704766265 | 0.197268809 | 3.572618852 | 0.000353429 | 0.004119111 | up |
| PHGR1 | 7077.174705 | -0.540881427 | 0.151419807 | -3.572065237 | 0.000354177 | 0.004125405 | down |
| AL354861.3 | 4.823713031 | -0.550268266 | 0.154059958 | -3.571779926 | 0.000354563 | 0.004128465 | down |
| PDE10A | 142.2020001 | 0.520667315 | 0.145865506 | 3.569502685 | 0.00035766 | 0.004155835 | up |
| DCDC2 | 149.9170099 | 0.709397783 | 0.1988241 | 3.567966776 | 0.000359762 | 0.004175913 | up |
| TDRD12 | 6.524537209 | 0.525799388 | 0.147396553 | 3.567243443 | 0.000360756 | 0.004180929 | up |
| EFNA5 | 147.4862477 | 0.542543402 | 0.152092333 | 3.567197587 | 0.000360819 | 0.004180929 | up |
| SNORA24B | 0.492532159 | -1.7743573 | 0.497574647 | -3.566012275 | 0.000362454 | 0.004194061 | down |
| FDCSP | 53.78677962 | 0.877190359 | 0.246032155 | 3.565348434 | 0.000363373 | 0.004202668 | up |
| CYP2C18 | 92.79526148 | 0.524544435 | 0.147194379 | 3.563617295 | 0.000365779 | 0.004223764 | up |
| LRRC3-AS1 | 1.022890394 | 0.664983108 | 0.186799546 | 3.559875403 | 0.000371031 | 0.004277022 | up |
| RNU1-88P | 0.765687518 | -2.180988988 | 0.612982717 | -3.557994256 | 0.000373697 | 0.004305182 | down |
| SLC5A9 | 133.9386228 | -0.575475826 | 0.161767615 | -3.557422956 | 0.000374511 | 0.004312679 | down |
| PTP4A1P1 | 0.845844272 | -1.248703831 | 0.351031306 | -3.5572435 | 0.000374767 | 0.004313833 | down |
| AC091305.2 | 2.402984824 | -0.709602261 | 0.199484963 | -3.557171687 | 0.000374869 | 0.004313833 | down |
| UBE2NL | 2.434922504 | -0.720404377 | 0.202721747 | -3.553661056 | 0.000379908 | 0.004364462 | down |
| PTMAP4 | 122.2676043 | 0.515024813 | 0.144951839 | 3.553075392 | 0.000380755 | 0.004372541 | up |
| LINC00941 | 43.01886554 | 0.719570754 | 0.202603344 | 3.551623289 | 0.000382863 | 0.004390709 | up |
| RFX6 | 13.93444131 | 0.98767449 | 0.278113011 | 3.551342269 | 0.000383272 | 0.004392388 | up |
| CACNA1B | 15.04117963 | -0.791040824 | 0.222762449 | -3.551051024 | 0.000383696 | 0.004395745 | down |
| STEAP4 | 161.9638563 | 0.512066199 | 0.144260707 | 3.549588873 | 0.000385833 | 0.004415691 | up |
| KCTD4 | 3.505159597 | -0.776220444 | 0.218768872 | -3.548130207 | 0.000387976 | 0.004432633 | down |
| AC104779.1 | 1.212166943 | 0.692761204 | 0.195297051 | 3.547217944 | 0.000389322 | 0.004441941 | up |
| AC010332.1 | 1.812454397 | -0.648903835 | 0.182946985 | -3.54695014 | 0.000389718 | 0.004444352 | down |
| AC113935.1 | 356.0841801 | 0.658824436 | 0.185802444 | 3.545832988 | 0.000391374 | 0.004457746 | up |
| GRIN1 | 95.49704255 | 0.56420533 | 0.159131886 | 3.545520275 | 0.000391839 | 0.004460001 | up |
| RN7SL559P | 0.933271557 | -1.041947259 | 0.293965984 | -3.544448395 | 0.000393436 | 0.004475128 | down |
| IRX3 | 49.64402935 | 0.715712671 | 0.201990002 | 3.543307416 | 0.000395142 | 0.00448843 | up |
| SPRR2E | 5.362712541 | 1.619327624 | 0.457122241 | 3.542438934 | 0.000396445 | 0.004498651 | up |
| LINC00629 | 1.677719602 | -0.796177673 | 0.224863173 | -3.540720611 | 0.000399036 | 0.004514262 | down |
| PTGER1 | 21.74489414 | -0.560270178 | 0.158268177 | -3.540005262 | 0.000400119 | 0.004524985 | down |
| AC138305.3 | 7.407042936 | -0.572840817 | 0.161828756 | -3.539796221 | 0.000400436 | 0.00452704 | down |
| DGKB | 10.59507311 | 0.692264935 | 0.195584693 | 3.539463772 | 0.000400941 | 0.004529683 | up |
| XG | 18.72972056 | -0.608326766 | 0.171959683 | -3.537612751 | 0.000403762 | 0.004556934 | down |
| AC007998.2 | 0.644102961 | 0.749653601 | 0.212124002 | 3.53403478 | 0.000409267 | 0.004611289 | up |
| AC009948.5 | 0.487945211 | -1.19297727 | 0.337680032 | -3.532862943 | 0.000411086 | 0.004627099 | down |
| MIR4754 | 1.449207915 | -0.772434465 | 0.218937151 | -3.528110516 | 0.000418537 | 0.004690452 | down |
| AL356652.1 | 3.697103052 | -0.553880214 | 0.157200159 | -3.523407464 | 0.000426036 | 0.004764904 | down |
| CLPS | 1.193020818 | 1.499162423 | 0.425748524 | 3.521239272 | 0.000429535 | 0.004799223 | up |
| AC078923.1 | 10.47492462 | 0.60079668 | 0.170662431 | 3.520380411 | 0.000430928 | 0.004813184 | up |
| AP000438.1 | 2.415118106 | 0.637005292 | 0.180968362 | 3.519981534 | 0.000431577 | 0.004817224 | up |
| ATP5F1P5 | 3.327482105 | -0.51154007 | 0.145363224 | -3.519047358 | 0.000433099 | 0.004827759 | down |
| AQP3 | 1223.401339 | 0.591349945 | 0.168050081 | 3.518891161 | 0.000433354 | 0.00482821 | up |
| AL390198.1 | 25.51778227 | -0.571985944 | 0.162631357 | -3.517070465 | 0.000436338 | 0.004851926 | down |
| AC092683.1 | 7.823413372 | 0.519426961 | 0.147688894 | 3.517034677 | 0.000436397 | 0.004851926 | up |
| LINC00589 | 10.29379881 | 0.620400609 | 0.176434376 | 3.516325008 | 0.000437565 | 0.004859718 | up |
| SNORA74D | 1.144383514 | -0.874540751 | 0.248747086 | -3.515782906 | 0.000438459 | 0.004868034 | down |
| TIMM8AP1 | 2.982935581 | 0.609388623 | 0.173356233 | 3.515239194 | 0.000439358 | 0.004873159 | up |
| AP004243.1 | 0.925040627 | 1.341786422 | 0.381928195 | 3.513190275 | 0.00044276 | 0.004901141 | up |
| PCDHA1 | 2.513114602 | 0.892801451 | 0.254155504 | 3.512815725 | 0.000443385 | 0.004904808 | up |
| KIF5A | 43.10945391 | -0.522693533 | 0.148816618 | -3.51233309 | 0.000444191 | 0.004910475 | down |
| MIR10B | 0.963745442 | 0.770980788 | 0.219640802 | 3.510189271 | 0.000447788 | 0.004943702 | up |
| LAIR2 | 24.43975408 | 0.573505735 | 0.163401178 | 3.509801724 | 0.000448441 | 0.004949279 | up |
| RPL7AP4 | 10.44460097 | -0.834502106 | 0.237842646 | -3.508631104 | 0.000450419 | 0.004967844 | down |
| HSPB7 | 174.5842801 | -0.589760881 | 0.168096526 | -3.508465615 | 0.000450699 | 0.004967844 | down |
| LINC01694 | 51.31178658 | -0.682685054 | 0.194592534 | -3.508279781 | 0.000451014 | 0.004967844 | down |
| PON1 | 9.284519537 | 0.863339188 | 0.246156376 | 3.50727941 | 0.000452714 | 0.004979998 | up |
| AC073592.2 | 3.200804884 | 0.63251463 | 0.180371317 | 3.506736211 | 0.000453639 | 0.004986893 | up |
| DTHD1 | 8.098974465 | 0.640698513 | 0.182784329 | 3.50521577 | 0.000456237 | 0.005012166 | up |
| PTGDR | 101.183918 | 0.714547938 | 0.203963896 | 3.503305977 | 0.000459521 | 0.005041618 | up |
| AC037198.2 | 14.4146422 | -0.51161744 | 0.14604685 | -3.503104939 | 0.000459868 | 0.00504377 | down |
| RPRM | 12.42091014 | -0.627795815 | 0.179258255 | -3.50218635 | 0.000461457 | 0.005054561 | down |
| AC080038.2 | 0.888119831 | 0.715866002 | 0.204527193 | 3.500101832 | 0.00046508 | 0.005086274 | up |
| RPL31P61 | 18.17791295 | -0.878991567 | 0.251134456 | -3.500083513 | 0.000465112 | 0.005086274 | down |
| AL645939.4 | 0.810094822 | 0.80299194 | 0.229450471 | 3.49963082 | 0.000465903 | 0.00509226 | up |
| ISM2 | 81.36302225 | -0.811424632 | 0.231862395 | -3.499595665 | 0.000465964 | 0.00509226 | down |
| PDCD5P1 | 4.855358166 | -0.635879595 | 0.181740313 | -3.498836258 | 0.000467293 | 0.005101783 | down |
| ANXA8 | 3.496565708 | 0.974234213 | 0.278518278 | 3.497918409 | 0.000468905 | 0.00511586 | up |
| IL17REL | 11.9172625 | 0.690709556 | 0.19752386 | 3.496841117 | 0.000470802 | 0.005130041 | up |
| AL355877.1 | 16.34511503 | -0.615731528 | 0.176094887 | -3.496589472 | 0.000471246 | 0.005131466 | down |
| KIR3DX1 | 1.379807748 | 0.727115073 | 0.208238362 | 3.4917441 | 0.000479878 | 0.005215337 | up |
| AL096803.2 | 1.064555443 | 0.825608177 | 0.236506121 | 3.490853314 | 0.000481481 | 0.005229826 | up |
| AC105046.1 | 3.459362899 | 0.588343299 | 0.168563812 | 3.490329807 | 0.000482425 | 0.005235044 | up |
| RPL18P10 | 3.562253978 | -0.578921634 | 0.165987808 | -3.487735875 | 0.000487129 | 0.005280416 | down |
| APOA4 | 2.229959919 | 1.59095122 | 0.456243439 | 3.487066513 | 0.00048835 | 0.005288507 | up |
| APCDD1L-AS1 | 4.762201234 | -0.538371271 | 0.154458136 | -3.485548164 | 0.00049113 | 0.005313449 | down |
| HMX3 | 7.284199676 | 1.046432709 | 0.300269626 | 3.484976892 | 0.00049218 | 0.005321363 | up |
| CNDP1 | 14.21405947 | 0.82737777 | 0.237564543 | 3.482749398 | 0.000496293 | 0.005357174 | up |
| RAD17P1 | 5.381797398 | 0.627112775 | 0.180089152 | 3.482235145 | 0.000497247 | 0.005365741 | up |
| AL031726.1 | 1.60646544 | 0.68300213 | 0.196319004 | 3.47904235 | 0.000503209 | 0.005421332 | up |
| RNU1-22P | 1.204803175 | -0.86082872 | 0.247451499 | -3.478777551 | 0.000503707 | 0.005424944 | down |
| AQP12A | 5.517019983 | -0.651415951 | 0.187305752 | -3.477821402 | 0.000505507 | 0.005440826 | down |
| PCDHA4 | 41.05193419 | -0.852320647 | 0.245084263 | -3.47766371 | 0.000505804 | 0.005441207 | down |
| AL807752.4 | 2.309346702 | 0.582029999 | 0.167363974 | 3.47763012 | 0.000505867 | 0.005441207 | up |
| CRABP1 | 15.19363727 | -0.744312495 | 0.214049614 | -3.477289593 | 0.00050651 | 0.005446371 | down |
| TMEM249 | 22.69581074 | -0.579087466 | 0.166660433 | -3.474654751 | 0.000511511 | 0.005483341 | down |
| PLA2G2D | 75.43139627 | 0.690615946 | 0.198763655 | 3.474558488 | 0.000511695 | 0.005483341 | up |
| ATP6V0A4 | 7.110693401 | 0.803311808 | 0.231207344 | 3.474421681 | 0.000511956 | 0.005483341 | up |
| AC021188.1 | 2.799786354 | 0.528573293 | 0.152174965 | 3.473457636 | 0.000513798 | 0.005498219 | up |
| KLK11 | 435.8594045 | 0.693856212 | 0.199769741 | 3.473279825 | 0.000514139 | 0.005500103 | up |
| LCN8 | 4.202252197 | 1.254885152 | 0.361351299 | 3.472756719 | 0.000515142 | 0.005509071 | up |
| KCNH3 | 19.80095743 | -0.517509579 | 0.149034494 | -3.472414771 | 0.000515799 | 0.005512519 | down |
| Z97653.2 | 3.281518406 | 0.62570514 | 0.180199823 | 3.472284989 | 0.000516048 | 0.005512519 | up |
| DPCR1 | 6.757843248 | 0.866674221 | 0.249606654 | 3.472159926 | 0.000516289 | 0.005512519 | up |
| PF4 | 92.24324845 | 0.655318189 | 0.188912404 | 3.468899735 | 0.000522594 | 0.005569178 | up |
| RGN | 39.69893694 | -0.653677187 | 0.188581778 | -3.466279693 | 0.000527714 | 0.005615322 | down |
| RN7SL105P | 0.550851235 | -1.44733187 | 0.417641043 | -3.465492418 | 0.000529261 | 0.005627673 | down |
| RGL3 | 228.1490401 | 0.551879109 | 0.159294377 | 3.464523482 | 0.000531172 | 0.005642603 | up |
| AP000654.2 | 0.88794578 | -0.794223843 | 0.229382951 | -3.462436242 | 0.000535309 | 0.005681136 | down |
| AC091304.1 | 0.707505595 | -1.453388949 | 0.419856291 | -3.461634323 | 0.000536906 | 0.005690865 | down |
| COMP | 750.0350483 | -0.712574981 | 0.206012211 | -3.45889682 | 0.000542393 | 0.005745376 | down |
| AC084262.1 | 2.401120505 | 0.809961352 | 0.234193162 | 3.458518371 | 0.000543155 | 0.005749298 | up |
| RNU6-118P | 1.537678207 | 0.504123535 | 0.145769035 | 3.458371906 | 0.000543451 | 0.005749298 | up |
| KRT17P3 | 1.129594839 | 1.201223218 | 0.347489148 | 3.456865411 | 0.000546498 | 0.005777879 | up |
| AC017013.1 | 1.221405938 | -0.808023965 | 0.233917729 | -3.454308358 | 0.000551706 | 0.005827418 | down |
| Z99943.2 | 3.619177618 | -0.554457148 | 0.160549964 | -3.453486599 | 0.00055339 | 0.005839668 | down |
| AC016705.2 | 14.77870032 | 0.71672637 | 0.207625272 | 3.452018929 | 0.000556409 | 0.005866825 | up |
| PRSS50 | 5.432325632 | -0.852872407 | 0.247176888 | -3.450453695 | 0.000559645 | 0.005896375 | down |
| AC012254.5 | 2.020210873 | 0.663179502 | 0.192265492 | 3.449290331 | 0.000562062 | 0.005916247 | up |
| AL135999.3 | 15.5901109 | -0.510507126 | 0.148079029 | -3.447531571 | 0.000565734 | 0.005947918 | down |
| COL4A2-AS1 | 2.480066774 | 0.532910779 | 0.15462489 | 3.446474749 | 0.000567952 | 0.005963225 | up |
| AL121985.1 | 5.463804137 | 0.606152793 | 0.17602148 | 3.443629685 | 0.000573961 | 0.006013107 | up |
| MYO16-AS1 | 3.185898548 | 0.949786461 | 0.275849012 | 3.443138889 | 0.000575004 | 0.006020259 | up |
| AC010336.2 | 1.038845251 | 0.694778272 | 0.201813069 | 3.442682264 | 0.000575976 | 0.006026658 | up |
| AC136475.3 | 227.8689529 | 0.603197191 | 0.175219556 | 3.442522092 | 0.000576317 | 0.006028342 | up |
| U62631.1 | 1.155266472 | 0.978266575 | 0.284257478 | 3.441480521 | 0.00057854 | 0.006047815 | up |
| LINC00989 | 1.685448295 | 0.686177114 | 0.199433022 | 3.440639402 | 0.000580341 | 0.006059839 | up |
| LINC01122 | 0.810855626 | 0.837598886 | 0.243496337 | 3.439882897 | 0.000581966 | 0.006072238 | up |
| RNU2-33P | 0.822735059 | 0.681240063 | 0.198146936 | 3.438055002 | 0.000585909 | 0.00611147 | up |
| AC245041.1 | 2.921797046 | 1.05114143 | 0.30580377 | 3.437306967 | 0.000587529 | 0.006124554 | up |
| NPIPB6 | 2.250008221 | 0.578897116 | 0.168450498 | 3.436600798 | 0.000589063 | 0.006138629 | up |
| AR | 59.79769289 | 0.515154062 | 0.149908543 | 3.436455661 | 0.000589379 | 0.006140005 | up |
| AC016831.5 | 10.38255816 | -0.597647921 | 0.173936451 | -3.43601308 | 0.000590342 | 0.006144281 | down |
| CATSPERZ | 0.720692239 | 0.643793251 | 0.187370903 | 3.4359297 | 0.000590524 | 0.006144281 | up |
| TCL6 | 13.4997014 | 0.947423685 | 0.275760379 | 3.435677339 | 0.000591074 | 0.006148094 | up |
| WT1 | 71.28960619 | -0.70466692 | 0.205238354 | -3.433407576 | 0.000596045 | 0.006194019 | down |
| SERP2 | 103.8986239 | -0.510322495 | 0.148734393 | -3.431099447 | 0.00060114 | 0.006235338 | down |
| AL121917.1 | 15.19174622 | -0.537695942 | 0.156744858 | -3.430389668 | 0.000602715 | 0.006247797 | down |
| OSR1 | 43.18635283 | 0.578015589 | 0.168553771 | 3.429265246 | 0.000605218 | 0.006271796 | up |
| Z98752.2 | 0.934067591 | -0.792839529 | 0.231275524 | -3.428116889 | 0.000607784 | 0.006291286 | down |
| FCRL6 | 15.22099615 | 0.509071243 | 0.148646687 | 3.424706279 | 0.000615464 | 0.006354352 | up |
| AC092802.3 | 0.896073058 | -0.791020164 | 0.23101119 | -3.424163838 | 0.000616694 | 0.006361629 | down |
| LYPLA1P3 | 5.870947408 | -0.503813876 | 0.147135728 | -3.424143695 | 0.00061674 | 0.006361629 | down |
| RN7SKP203 | 0.810923406 | -2.70235371 | 0.789271811 | -3.423856867 | 0.000617391 | 0.006366384 | down |
| SERPINA10 | 75.55861323 | -0.821076031 | 0.239827223 | -3.42361481 | 0.000617941 | 0.006370092 | down |
| PAX2 | 4.33396836 | 0.786215277 | 0.229672242 | 3.42320548 | 0.000618873 | 0.006377727 | up |
| AC009303.2 | 1.621310785 | -0.842065268 | 0.24601784 | -3.422781326 | 0.000619839 | 0.006383752 | down |
| SLC22A11 | 100.0950117 | -0.720889644 | 0.210858385 | -3.418833179 | 0.000628903 | 0.00644929 | down |
| SOHLH2 | 1.692150206 | -1.080080371 | 0.315966082 | -3.418342764 | 0.000630037 | 0.006458942 | down |
| HOXD10 | 134.5205347 | -0.631333564 | 0.184871709 | -3.414982029 | 0.000637862 | 0.006523165 | down |
| AL031846.1 | 3.036872288 | 0.562440455 | 0.164736338 | 3.414185743 | 0.000639729 | 0.006540261 | up |
| PTCHD1 | 18.32100795 | 0.709877577 | 0.208021823 | 3.412514927 | 0.000643664 | 0.006572367 | up |
| AC110285.3 | 6.021495344 | 0.807153301 | 0.236598529 | 3.411489091 | 0.000646091 | 0.006587172 | up |
| AC012368.2 | 2.784909868 | 0.529448252 | 0.155383184 | 3.407371632 | 0.000655918 | 0.006677181 | up |
| GPR158 | 35.95975517 | 0.576305047 | 0.169187144 | 3.406317013 | 0.000658457 | 0.00670037 | up |
| AL136528.1 | 1.206897434 | 0.767832387 | 0.225561759 | 3.40408938 | 0.00066385 | 0.006747665 | up |
| STAP1 | 14.7370754 | 0.534535709 | 0.157179586 | 3.400796006 | 0.000671899 | 0.006798484 | up |
| NAT8B | 40.5391126 | 0.74844489 | 0.220124317 | 3.400100906 | 0.00067361 | 0.006811669 | up |
| AC099811.4 | 0.945959781 | -0.867436859 | 0.255204194 | -3.398991397 | 0.000676348 | 0.006837294 | down |
| GCG | 26.7544458 | -0.983877358 | 0.289495705 | -3.398590524 | 0.00067734 | 0.006844767 | down |
| ADAMTS16 | 57.88262851 | -0.570610226 | 0.167899262 | -3.398527293 | 0.000677497 | 0.006844767 | down |
| SNX18P7 | 1.366023032 | -1.335015235 | 0.392835056 | -3.398411656 | 0.000677783 | 0.006845593 | down |
| PNMA6A | 12.01256316 | -0.608745379 | 0.17913173 | -3.398311273 | 0.000678032 | 0.006845666 | down |
| AC103858.1 | 1.551296047 | 0.612560682 | 0.180258029 | 3.398243539 | 0.0006782 | 0.006845666 | up |
| AGXT | 39.10596577 | -0.537561965 | 0.158199006 | -3.39801101 | 0.000678777 | 0.006845736 | down |
| LINC02041 | 8.309510356 | 0.628869595 | 0.18533559 | 3.393139946 | 0.000690963 | 0.006944966 | up |
| FXYD1 | 9.400341855 | -0.510739707 | 0.15052643 | -3.393023439 | 0.000691257 | 0.006944966 | down |
| AP006565.1 | 1.895291423 | 0.880953348 | 0.259654923 | 3.392785074 | 0.000691859 | 0.006947885 | up |
| GBP1P1 | 43.87788103 | 0.50071636 | 0.147596616 | 3.392465034 | 0.000692668 | 0.006951831 | up |
| RN7SKP185 | 0.594435152 | -1.254016818 | 0.369702251 | -3.391964248 | 0.000693935 | 0.006955022 | down |
| AC105243.1 | 4.532436279 | 1.499749569 | 0.442194205 | 3.391608373 | 0.000694837 | 0.006961066 | up |
| KCNK15-AS1 | 8.104285238 | -0.735019705 | 0.216750004 | -3.39109431 | 0.000696141 | 0.006967873 | down |
| MAPT | 60.07730018 | 0.554866206 | 0.163728605 | 3.388938711 | 0.000701637 | 0.007018678 | up |
| DPY19L2P1 | 1.718693578 | 0.713774514 | 0.210676631 | 3.388009907 | 0.000704017 | 0.007031971 | up |
| AL033519.2 | 29.4949313 | -0.526249607 | 0.15533548 | -3.387826192 | 0.000704489 | 0.007034582 | down |
| HS6ST3 | 5.303290871 | 0.820878042 | 0.24235606 | 3.387074549 | 0.000706422 | 0.007043367 | up |
| FLJ12825 | 3.842258571 | 0.642153339 | 0.189734442 | 3.384484819 | 0.00071312 | 0.007093228 | up |
| UMODL1 | 48.82119672 | -0.607071138 | 0.179381041 | -3.384254749 | 0.000713717 | 0.007097064 | down |
| FTLP3 | 302.9497441 | -0.548558625 | 0.162109537 | -3.383876338 | 0.000714702 | 0.00710297 | down |
| AC013439.1 | 4.581630545 | -0.712206653 | 0.210471472 | -3.383863118 | 0.000714736 | 0.00710297 | down |
| AC016825.1 | 1.419824819 | -0.863475682 | 0.255250837 | -3.38285152 | 0.000717374 | 0.00712495 | down |
| AC123912.1 | 1.880496062 | -0.777057007 | 0.229789212 | -3.381607867 | 0.000720629 | 0.007155156 | down |
| RPL5P5 | 12.68861382 | -0.621341247 | 0.183779129 | -3.380913009 | 0.000722454 | 0.00716066 | down |
| SIRPAP1 | 2.3685856 | -0.642746967 | 0.190110778 | -3.380907553 | 0.000722468 | 0.00716066 | down |
| TRAV41 | 4.149987882 | 0.590745508 | 0.174752989 | 3.380460099 | 0.000723646 | 0.00716808 | up |
| DUXAP8 | 29.39946878 | -0.614053454 | 0.181652595 | -3.38037259 | 0.000723876 | 0.00716824 | down |
| SBSPON | 555.4164335 | 0.623205055 | 0.184394176 | 3.379743691 | 0.000725535 | 0.007180409 | up |
| C20orf204 | 42.66036436 | -0.511950505 | 0.151490621 | -3.37942047 | 0.000726388 | 0.007185205 | down |
| SERPINB5 | 2541.489263 | 0.559319684 | 0.165530026 | 3.378962104 | 0.0007276 | 0.007192339 | up |
| LINC01170 | 1.611497522 | 0.948647268 | 0.280769573 | 3.378739577 | 0.00072819 | 0.007196036 | up |
| TGM3 | 64.13744692 | -0.521729414 | 0.154515136 | -3.376558613 | 0.000733987 | 0.007242629 | down |
| GRPR | 29.70588411 | -0.738570421 | 0.218855103 | -3.374700476 | 0.000738961 | 0.007280962 | down |
| SATB2-AS1 | 402.579013 | -0.516435438 | 0.153050777 | -3.374275178 | 0.000740103 | 0.007287927 | down |
| RPL30P4 | 18.25599085 | -0.705904004 | 0.209328307 | -3.372233862 | 0.000745611 | 0.007330124 | down |
| AP003484.1 | 0.785194403 | 0.781962996 | 0.231968667 | 3.37098543 | 0.000748998 | 0.007358181 | up |
| AC010627.1 | 4.804990229 | -0.591040658 | 0.175380109 | -3.370055251 | 0.000751531 | 0.00737873 | down |
| C3orf14 | 115.3239662 | -0.511191009 | 0.151727055 | -3.3691487 | 0.000754007 | 0.007396378 | down |
| LINC01555 | 30.12724668 | -0.514684333 | 0.152767681 | -3.369065573 | 0.000754235 | 0.007396378 | down |
| AC005695.1 | 1.0593387 | 0.83234655 | 0.24705724 | 3.369043341 | 0.000754296 | 0.007396378 | up |
| AC073109.1 | 0.937367381 | -1.062136999 | 0.315275987 | -3.368911817 | 0.000754656 | 0.007396378 | down |
| CILP2 | 69.01243343 | -0.578204863 | 0.171872629 | -3.364147436 | 0.000767805 | 0.007501073 | down |
| HADHAP1 | 4.53519543 | -0.595652329 | 0.17708363 | -3.363678098 | 0.000769112 | 0.00750889 | down |
| PAX5 | 103.3250714 | 0.689246545 | 0.204918716 | 3.36351193 | 0.000769575 | 0.007509589 | up |
| TRGV2 | 1.82228963 | 0.628418452 | 0.18695061 | 3.361414293 | 0.000775444 | 0.007553635 | up |
| SHOX | 3.733850136 | -0.610916265 | 0.18176834 | -3.360960795 | 0.000776718 | 0.007563845 | down |
| FAM183BP | 1.067244171 | 0.760455407 | 0.22629103 | 3.360519453 | 0.000777961 | 0.007573734 | up |
| LINC01214 | 4.203192886 | -0.572115137 | 0.170256939 | -3.360304379 | 0.000778566 | 0.007577427 | down |
| LINC02520 | 1.235315636 | -0.958223341 | 0.285313203 | -3.35849631 | 0.000783678 | 0.007620519 | down |
| NTSR1 | 95.95239812 | 0.718312587 | 0.213918872 | 3.357873857 | 0.000785444 | 0.007631583 | up |
| AC016597.1 | 3.402665367 | 0.585991237 | 0.1745136 | 3.357854271 | 0.0007855 | 0.007631583 | up |
| ART1 | 0.744206114 | 0.990248265 | 0.29496535 | 3.357168103 | 0.000787452 | 0.007646106 | up |
| OTC | 96.66776088 | 0.738866262 | 0.220106573 | 3.356856874 | 0.000788339 | 0.007648179 | up |
| WDR17 | 9.462353059 | -0.6630707 | 0.197531517 | -3.356784323 | 0.000788546 | 0.007648179 | down |
| AC008694.2 | 1.437803539 | -0.817309321 | 0.243490697 | -3.356634692 | 0.000788973 | 0.007649765 | down |
| TAAR3P | 2.248943251 | 0.916381098 | 0.273062119 | 3.355943695 | 0.000790947 | 0.007662239 | up |
| RPS21P8 | 0.731874 | -1.03977222 | 0.310033635 | -3.353740054 | 0.000797272 | 0.007715811 | down |
| AC073592.10 | 1.941577524 | 0.661246411 | 0.197291477 | 3.351621771 | 0.000803397 | 0.007755889 | up |
| LINC02126 | 1.862274199 | -0.623242348 | 0.185976657 | -3.351185894 | 0.000804663 | 0.007765866 | down |
| RN7SKP237 | 0.598116431 | -1.110022255 | 0.331392605 | -3.349568572 | 0.000809375 | 0.007793353 | down |
| PRH2 | 2.230584337 | 0.647712088 | 0.193529759 | 3.346834573 | 0.0008174 | 0.007859306 | up |
| AC104564.1 | 2.775775497 | -0.666153229 | 0.199046064 | -3.346728969 | 0.000817711 | 0.00786004 | down |
| MEG8 | 1.951903782 | -0.669308921 | 0.200010055 | -3.34637637 | 0.000818752 | 0.007863262 | down |
| CYP2B6 | 1051.584634 | -0.530214119 | 0.158474367 | -3.345740581 | 0.000820631 | 0.007878843 | down |
| ADARB2-AS1 | 0.658231676 | -1.099264536 | 0.328601228 | -3.345284322 | 0.000821983 | 0.007887495 | down |
| AP000640.2 | 1.062024394 | 0.55219282 | 0.165124644 | 3.344096969 | 0.000825509 | 0.007914518 | up |
| CABP7 | 13.50800684 | 0.50468603 | 0.150960097 | 3.343175042 | 0.000828256 | 0.007934036 | up |
| AL096870.1 | 1.227139955 | 0.573258852 | 0.171509927 | 3.342423738 | 0.000830502 | 0.007949602 | up |
| AF001548.3 | 1.526636711 | -0.852249653 | 0.25512788 | -3.340480282 | 0.000836336 | 0.007997689 | down |
| HIST1H3A | 2.133760789 | 0.505963239 | 0.151509872 | 3.339473736 | 0.000839373 | 0.00801371 | up |
| C10orf99 | 2500.284118 | -0.554465563 | 0.166035073 | -3.33944842 | 0.000839449 | 0.00801371 | down |
| AC025244.1 | 1.25639667 | -0.803972084 | 0.240756782 | -3.339353844 | 0.000839735 | 0.008014152 | down |
| TRPM6 | 218.8281567 | -0.530041793 | 0.158771744 | -3.338388675 | 0.000842658 | 0.008035165 | down |
| DCAF13P3 | 2.497808585 | -0.605853899 | 0.181562766 | -3.336884057 | 0.000847233 | 0.008061706 | down |
| LINC02525 | 4.281704981 | -1.036163235 | 0.31054741 | -3.336570207 | 0.00084819 | 0.00806492 | down |
| FGF21 | 0.859837121 | -0.837279141 | 0.251094144 | -3.334522773 | 0.000854459 | 0.008114566 | down |
| KRT84 | 1.451788784 | 1.085696907 | 0.325598153 | 3.334468878 | 0.000854625 | 0.008114566 | up |
| ORAOV1P1 | 2.879270858 | 0.627043284 | 0.188073216 | 3.334038186 | 0.000855949 | 0.008122529 | up |
| SLC26A2 | 3385.732252 | -0.529938396 | 0.159046799 | -3.331965176 | 0.00086235 | 0.008160119 | down |
| HCN2 | 33.48794792 | 0.528041799 | 0.158528061 | 3.330904294 | 0.000865644 | 0.008186648 | up |
| RPL21P12 | 1.157628063 | -0.812499221 | 0.243987306 | -3.330088081 | 0.000868185 | 0.008202056 | down |
| RPS6KA6 | 220.0931054 | -0.538992632 | 0.161920861 | -3.328741142 | 0.000872394 | 0.008233575 | down |
| UGT2B15 | 141.7269394 | 0.613601299 | 0.184337537 | 3.328683404 | 0.000872575 | 0.008233575 | up |
| CALB1 | 122.9406533 | 0.921576844 | 0.27709779 | 3.325818095 | 0.000881594 | 0.008302688 | up |
| DNAJC5G | 1.398052081 | 0.738214422 | 0.221980796 | 3.325577868 | 0.000882354 | 0.008307099 | up |
| AL020993.1 | 0.74886284 | 0.69270698 | 0.208370361 | 3.324402644 | 0.000886082 | 0.008337839 | up |
| AL445490.1 | 6.030891676 | 0.552369211 | 0.166174289 | 3.324035345 | 0.00088725 | 0.008341442 | up |
| AC103591.3 | 7.703227771 | -0.505792893 | 0.152166361 | -3.323946838 | 0.000887531 | 0.008341745 | down |
| AC074397.1 | 1.013461079 | -1.008153654 | 0.303369255 | -3.323189937 | 0.000889943 | 0.008359714 | down |
| TRAV19 | 6.468546251 | 0.50408365 | 0.151835297 | 3.319937193 | 0.000900377 | 0.008447966 | up |
| HOXD11 | 72.14294396 | -0.629746469 | 0.189691632 | -3.319843173 | 0.00090068 | 0.008447966 | down |
| RUNDC3A | 31.46181223 | -0.525825586 | 0.158422171 | -3.319141394 | 0.000902947 | 0.008460479 | down |
| AP000943.1 | 1.028980956 | 1.040965201 | 0.313682537 | 3.318530931 | 0.000904923 | 0.008471873 | up |
| RN7SKP180 | 0.594230919 | -1.077765282 | 0.324919835 | -3.317019048 | 0.000909834 | 0.008508323 | down |
| AC007278.1 | 2.58896613 | 0.620127239 | 0.186976262 | 3.316609458 | 0.000911169 | 0.008516042 | up |
| LINC02068 | 3.005787315 | 0.539243826 | 0.162641265 | 3.315541277 | 0.000914658 | 0.008541494 | up |
| ANKRD55 | 3.965066323 | 0.508598995 | 0.153406522 | 3.315367493 | 0.000915227 | 0.00854442 | up |
| AC093850.1 | 0.906260052 | -0.941597996 | 0.284158335 | -3.313638484 | 0.000920905 | 0.008590232 | down |
| ORM1 | 20.31276819 | -0.957943459 | 0.289100307 | -3.31353318 | 0.000921252 | 0.008591071 | down |
| KRT18P22 | 1.644354492 | 0.652568714 | 0.196992344 | 3.312660278 | 0.000924132 | 0.008610723 | up |
| LINC00565 | 4.005337362 | 0.553823478 | 0.167249781 | 3.311355472 | 0.000928452 | 0.008646159 | up |
| NKAIN1 | 9.312693001 | 0.562047916 | 0.169747868 | 3.311074953 | 0.000929383 | 0.008652422 | up |
| AC006529.1 | 2.241104549 | -0.978465184 | 0.295568029 | -3.310456772 | 0.000931438 | 0.008669143 | down |
| TECTB | 1.770920602 | -0.599351605 | 0.181056963 | -3.310293036 | 0.000931983 | 0.008670938 | down |
| ANO7 | 326.708049 | 0.505409213 | 0.152700692 | 3.309803024 | 0.000933616 | 0.008682166 | up |
| MLIP | 4.001905682 | 0.6123969 | 0.185033792 | 3.30964898 | 0.00093413 | 0.008682291 | up |
| CLEC18B | 5.196761214 | 0.537718984 | 0.162504775 | 3.30894267 | 0.00093649 | 0.008694383 | up |
| AL138889.1 | 1.116031351 | -0.693981615 | 0.209765628 | -3.308366675 | 0.000938419 | 0.008709868 | down |
| AOAH-IT1 | 2.61952609 | -0.623707288 | 0.188536019 | -3.308159854 | 0.000939112 | 0.008713885 | down |
| DES | 5158.496701 | -0.744777421 | 0.225149327 | -3.307926474 | 0.000939895 | 0.00871405 | down |
| AP000812.2 | 2.762359198 | -0.719682538 | 0.217565761 | -3.307885089 | 0.000940034 | 0.00871405 | down |
| AC005523.1 | 1.150317459 | 0.58167315 | 0.175846615 | 3.307843881 | 0.000940172 | 0.00871405 | up |
| THRB-IT1 | 0.513491311 | -1.051474246 | 0.31796518 | -3.306884885 | 0.000943397 | 0.008736672 | down |
| AC103740.2 | 5.821847553 | -0.69375244 | 0.209893261 | -3.305263042 | 0.000948873 | 0.008766484 | down |
| C12orf71 | 1.229506855 | 0.567196678 | 0.171658766 | 3.304210395 | 0.000952443 | 0.008793665 | up |
| FAM69C | 11.7590291 | 0.762369022 | 0.230734821 | 3.304091767 | 0.000952846 | 0.008794959 | up |
| AL109935.2 | 0.991460599 | -1.072528566 | 0.324658857 | -3.303555547 | 0.000954671 | 0.00880935 | down |
| MIR4449 | 1.099130063 | -1.081800703 | 0.327670263 | -3.301491851 | 0.000961721 | 0.008852442 | down |
| AL139174.1 | 2.365092556 | -0.544119883 | 0.164854133 | -3.300614148 | 0.000964735 | 0.008872852 | down |
| TM4SF20 | 437.7488715 | 0.672024197 | 0.203708449 | 3.298951027 | 0.000970468 | 0.008909425 | up |
| TATDN1P1 | 4.633236966 | -0.531979125 | 0.161258578 | -3.298919868 | 0.000970576 | 0.008909425 | down |
| SLC13A2 | 305.2019849 | -0.717914471 | 0.217637898 | -3.298664785 | 0.000971458 | 0.008915077 | down |
| AC009812.2 | 0.879622005 | -1.270091828 | 0.385201796 | -3.297211595 | 0.000976499 | 0.008952016 | down |
| AC090116.1 | 10.35908863 | 0.625493565 | 0.189704715 | 3.297195674 | 0.000976554 | 0.008952016 | up |
| RPL5P10 | 1.097609303 | -1.004627804 | 0.30481103 | -3.295903714 | 0.000981056 | 0.008988357 | down |
| AP3B2 | 14.40429747 | -0.572396724 | 0.173676804 | -3.295758044 | 0.000981565 | 0.008989799 | down |
| AC027279.1 | 7.167775692 | -0.54325519 | 0.164863424 | -3.295183234 | 0.000983575 | 0.009004035 | down |
| MIR6859-1 | 2.491995396 | -0.6268701 | 0.190277123 | -3.294511136 | 0.00098593 | 0.009023125 | down |
| TMEM229A | 3.681160753 | 0.998622653 | 0.303335964 | 3.292134035 | 0.000994302 | 0.009094764 | up |
| HEPACAM2 | 606.0647981 | 0.704935929 | 0.214175588 | 3.291392529 | 0.000996927 | 0.009111297 | up |
| AC133961.1 | 1.175900043 | 0.577043094 | 0.175340208 | 3.290991277 | 0.00099835 | 0.009121634 | up |
| CLEC18C | 0.988092643 | 0.976534893 | 0.296763664 | 3.290614762 | 0.000999687 | 0.009129038 | up |
| RPS2P45 | 1.118759929 | -0.760932158 | 0.231290329 | -3.289943686 | 0.001002074 | 0.009145843 | down |
| Z97353.1 | 55.7593172 | -0.668902168 | 0.203358568 | -3.289274579 | 0.00100446 | 0.009165113 | down |
| HES5 | 14.77450652 | 0.600733207 | 0.182648974 | 3.289004005 | 0.001005426 | 0.009168925 | up |
| AL035420.3 | 2.293166657 | -0.53276435 | 0.162009808 | -3.288469737 | 0.001007336 | 0.00917809 | down |
| RNA5SP477 | 0.779945621 | 0.779942601 | 0.237180178 | 3.288397068 | 0.001007596 | 0.00917809 | up |
| LINC02347 | 1.160339411 | 1.716283676 | 0.52196008 | 3.288151226 | 0.001008477 | 0.009181722 | up |
| RFX4 | 2.321860856 | 0.538803264 | 0.163934628 | 3.286695863 | 0.001013703 | 0.009219262 | up |
| MLN | 2.040879011 | 1.371452104 | 0.417353869 | 3.28606539 | 0.001015974 | 0.009235118 | up |
| AC009093.5 | 1.761203298 | -0.697027323 | 0.212367429 | -3.282176211 | 0.001030092 | 0.009332785 | down |
| SNORA7B | 1.148231706 | -0.808504448 | 0.246372099 | -3.281639645 | 0.001032054 | 0.009345497 | down |
| AC005832.1 | 0.524209922 | 0.745660668 | 0.227261804 | 3.281064639 | 0.00103416 | 0.009362035 | up |
| HMGN2P31 | 0.872870022 | 0.592635623 | 0.180730551 | 3.279111476 | 0.001041345 | 0.009409245 | up |
| AC009014.1 | 145.3236634 | -0.592404303 | 0.180690675 | -3.278554929 | 0.001043401 | 0.009425272 | down |
| FCRL3 | 35.7302189 | 0.544083986 | 0.165971944 | 3.278168429 | 0.00104483 | 0.009431981 | up |
| AC015849.1 | 1.244129911 | 0.613221501 | 0.187170842 | 3.276266185 | 0.001051894 | 0.00948406 | up |
| VTN | 12.88969106 | 0.533055738 | 0.162727543 | 3.275756075 | 0.001053795 | 0.009498644 | up |
| DNAH11 | 14.35257851 | 0.512173724 | 0.156364257 | 3.275516625 | 0.001054689 | 0.009501578 | up |
| ASMT | 4.045732316 | -0.540985006 | 0.165354988 | -3.271658222 | 0.001069187 | 0.009608895 | down |
| MS4A10 | 1.364413329 | 0.908170302 | 0.277596626 | 3.271546615 | 0.00106961 | 0.009608937 | up |
| AC015743.1 | 1.269629765 | 0.939666037 | 0.287256503 | 3.271174111 | 0.001071019 | 0.009617606 | up |
| RTP3 | 0.735374891 | -0.955140032 | 0.292009264 | -3.270923736 | 0.001071968 | 0.009620957 | down |
| AC012640.3 | 2.079301699 | 0.606073966 | 0.185343179 | 3.270009546 | 0.001075439 | 0.009646752 | up |
| RPL7P16 | 3.36775964 | 0.516195073 | 0.157861465 | 3.26992451 | 0.001075762 | 0.009646752 | up |
| LINC00607 | 8.191835192 | -0.650409872 | 0.198910439 | -3.269862934 | 0.001075996 | 0.009646752 | down |
| RN7SL444P | 1.15091909 | -0.790777965 | 0.241872102 | -3.269405434 | 0.001077738 | 0.009659775 | down |
| AC126768.2 | 1.102914865 | -1.044374777 | 0.319496294 | -3.268816557 | 0.001079983 | 0.00967065 | down |
| RPEL1 | 3.031613195 | -0.522371982 | 0.15980781 | -3.268751262 | 0.001080232 | 0.00967065 | down |
| BLOC1S5-TXNDC5 | 3.106773036 | 0.530071323 | 0.162195281 | 3.268105699 | 0.001082699 | 0.009688666 | up |
| CLCNKB | 3.54933735 | -0.594995302 | 0.182100166 | -3.267406706 | 0.001085376 | 0.009707232 | down |
| AC005476.1 | 2.157840723 | -0.799489814 | 0.244704008 | -3.267170904 | 0.001086281 | 0.009710323 | down |
| IGSF1 | 24.83142507 | -0.659727179 | 0.201940042 | -3.266945827 | 0.001087145 | 0.009712853 | down |
| KCCAT198 | 1.180735498 | -0.706591709 | 0.216361157 | -3.265797429 | 0.001091563 | 0.009749722 | down |
| AL031595.2 | 2.52281666 | 0.614010731 | 0.18803709 | 3.265370312 | 0.001093211 | 0.00976183 | up |
| AL031665.1 | 3.081037453 | 0.781631204 | 0.239379975 | 3.265232205 | 0.001093744 | 0.009762795 | up |
| AC093155.1 | 2.179542746 | -0.876131635 | 0.268403623 | -3.264231779 | 0.001097613 | 0.009785467 | down |
| AC104561.3 | 2.175719299 | 0.506186632 | 0.155129899 | 3.262985635 | 0.001102451 | 0.009823359 | up |
| SERPINB7 | 18.16741025 | 0.807875949 | 0.2476843 | 3.261716415 | 0.001107399 | 0.009862189 | up |
| KLK4 | 24.54779856 | 0.656805157 | 0.201462366 | 3.260187844 | 0.001113384 | 0.009907581 | up |
| UGT2B10 | 8.1798897 | -0.75757039 | 0.232532643 | -3.257909865 | 0.00112236 | 0.009982143 | down |
| LINC01641 | 3.017572778 | -0.886285666 | 0.272051589 | -3.257785291 | 0.001122853 | 0.009982724 | down |
| KIR2DL3 | 1.820418139 | 0.826686972 | 0.253760691 | 3.257742437 | 0.001123023 | 0.009982724 | up |
| AL353151.1 | 6.437738248 | -0.850054689 | 0.260940088 | -3.257662312 | 0.00112334 | 0.009982889 | down |
| MIR8075 | 0.806454499 | -1.692820274 | 0.519671337 | -3.257482475 | 0.001124052 | 0.009986563 | down |
| UBD | 585.8684131 | 0.500240568 | 0.153575708 | 3.257289665 | 0.001124816 | 0.009989841 | up |
| WNT7A | 6.387288233 | -0.953308996 | 0.292673988 | -3.257238546 | 0.001125018 | 0.009989841 | down |
| MIR583HG | 0.857445995 | 0.583823991 | 0.179280372 | 3.256485832 | 0.001128006 | 0.010003085 | up |
| AL512306.1 | 1.768574863 | -0.82520704 | 0.253442705 | -3.255990506 | 0.001129975 | 0.010017896 | down |
| AC009486.1 | 0.784684925 | 0.780563143 | 0.239834786 | 3.254586861 | 0.001135574 | 0.010059534 | up |
| AL121761.2 | 46.36893115 | 0.651522324 | 0.200210512 | 3.254186386 | 0.001137176 | 0.010068404 | up |
| CCDC187 | 29.80696496 | 0.563114143 | 0.173052287 | 3.254011567 | 0.001137876 | 0.010069258 | up |
| C10orf82 | 43.99569471 | -0.741310952 | 0.227847613 | -3.253538371 | 0.001139773 | 0.010080783 | down |
| AL356056.2 | 1.214288461 | 0.680077695 | 0.209027237 | 3.253536267 | 0.001139782 | 0.010080783 | up |
| AC092127.1 | 1.948778609 | -0.671584335 | 0.206486439 | -3.252437978 | 0.001144196 | 0.010111799 | down |
| RPS26P13 | 5.847220818 | -0.558688699 | 0.171862107 | -3.250796293 | 0.001150823 | 0.010154266 | down |
| AL450311.2 | 0.836911212 | 0.858419181 | 0.264093684 | 3.250434345 | 0.001152289 | 0.010161837 | up |
| AF186192.1 | 1.846859425 | -0.787894884 | 0.242441635 | -3.249833237 | 0.001154727 | 0.010177972 | down |
| HMGN2P3 | 14.06538997 | 0.547044445 | 0.168386528 | 3.248742349 | 0.001159164 | 0.010203635 | up |
| FBN3 | 4.946118113 | 0.83181088 | 0.256162667 | 3.247197917 | 0.001165473 | 0.010245685 | up |
| AC007991.4 | 2.013362753 | 0.814148756 | 0.250752849 | 3.246817569 | 0.001167032 | 0.01025669 | up |
| AL645608.4 | 3.124564005 | -0.688026487 | 0.211950418 | -3.246167156 | 0.001169701 | 0.010274753 | down |
| ATP11A-AS1 | 1.183675025 | -0.995252568 | 0.306801958 | -3.243957684 | 0.001178813 | 0.010346633 | down |
| SLC35F3 | 3.146595754 | 0.659049086 | 0.203181558 | 3.243646193 | 0.001180102 | 0.010352519 | up |
| SLAMF6P1 | 6.189586011 | 0.581282394 | 0.179234902 | 3.243131716 | 0.001182235 | 0.010365793 | up |
| FTCD | 6.574028244 | 0.708532038 | 0.218596023 | 3.241285133 | 0.001189921 | 0.010422249 | up |
| ZDHHC19 | 3.888081627 | -0.527127394 | 0.162675641 | -3.240358477 | 0.001193795 | 0.010450708 | down |
| XCL2 | 7.110578195 | 0.512051214 | 0.158055355 | 3.239695453 | 0.001196574 | 0.010469555 | up |
| AL354707.3 | 1.942563079 | -0.718424308 | 0.221790099 | -3.239208203 | 0.00119862 | 0.010481973 | down |
| LIPF | 0.815124394 | 1.550235856 | 0.478617611 | 3.238986239 | 0.001199554 | 0.010482425 | up |
| UTS2 | 29.49104339 | 0.616694372 | 0.190398165 | 3.238972237 | 0.001199613 | 0.010482425 | up |
| LINC00704 | 15.85041562 | -0.559968659 | 0.172907402 | -3.238546486 | 0.001201404 | 0.010495341 | down |
| OR2I1P | 489.245064 | 0.524980409 | 0.162142196 | 3.237777832 | 0.001204646 | 0.010515663 | up |
| AC108449.1 | 0.897170574 | -0.800485407 | 0.247233473 | -3.237771148 | 0.001204674 | 0.010515663 | down |
| AC009652.1 | 0.544813034 | 0.807557377 | 0.249450914 | 3.237339819 | 0.001206496 | 0.010528558 | up |
| RPS15AP36 | 1.995189496 | -0.520740899 | 0.160860258 | -3.237225312 | 0.001206981 | 0.010528558 | down |
| CST6 | 25.14288071 | -0.647916411 | 0.200217673 | -3.236060044 | 0.001211919 | 0.010562361 | down |
| SNORA22 | 1.309852531 | -0.849878294 | 0.262646841 | -3.235821492 | 0.001212932 | 0.010568437 | down |
| RPL5P22 | 8.794812641 | -0.584114554 | 0.180684554 | -3.23278631 | 0.001225892 | 0.01067024 | down |
| AC010343.3 | 3.778910523 | -0.789754319 | 0.244358023 | -3.231955754 | 0.001229461 | 0.010689619 | down |
| CSTP2 | 1.056422329 | -0.754768051 | 0.233537215 | -3.231896256 | 0.001229717 | 0.010689619 | down |
| CCDC194 | 2.061414045 | 0.746642382 | 0.231084726 | 3.231033031 | 0.001233437 | 0.010716384 | up |
| AC012653.2 | 1.000964421 | -1.438802224 | 0.445355948 | -3.230679258 | 0.001234964 | 0.010726869 | down |
| F11 | 3.626251161 | -0.818091299 | 0.253240109 | -3.23049656 | 0.001235754 | 0.010728447 | down |
| POM121L2 | 1.784808744 | -0.636880847 | 0.197146902 | -3.230488748 | 0.001235788 | 0.010728447 | down |
| AC022075.1 | 141.9274025 | 0.520708733 | 0.16132353 | 3.227729609 | 0.001247769 | 0.010812446 | up |
| AC106772.1 | 4.217493946 | 0.532700386 | 0.165042027 | 3.227665074 | 0.00124805 | 0.010812446 | up |
| AC103705.1 | 1.007644268 | -0.906770041 | 0.281158188 | -3.225124075 | 0.001259181 | 0.010891947 | down |
| AC007405.2 | 1.430759422 | 0.813624114 | 0.252411643 | 3.223401677 | 0.001266778 | 0.010946335 | up |
| MCOLN3 | 87.27468485 | 0.532401714 | 0.165176582 | 3.223227585 | 0.001267548 | 0.010947333 | up |
| AC148477.2 | 4.479199041 | 0.645676851 | 0.200405532 | 3.221851445 | 0.001273652 | 0.010983028 | up |
| PGLYRP4 | 7.19737261 | 0.671727298 | 0.208551042 | 3.220925159 | 0.001277775 | 0.011007233 | up |
| KARSP3 | 0.70207668 | -0.951043008 | 0.295294612 | -3.220658187 | 0.001278966 | 0.011011817 | down |
| AL034370.1 | 8.044722529 | -0.591931826 | 0.183824354 | -3.220094695 | 0.001281483 | 0.011029986 | down |
| ERVH-1 | 6.701158285 | -0.814514864 | 0.252951937 | -3.220038058 | 0.001281736 | 0.011029986 | down |
| SCNN1G | 15.71847586 | -0.773571188 | 0.240308339 | -3.219077592 | 0.001286037 | 0.011055619 | down |
| PHACTR2-AS1 | 1.691199457 | -0.815714922 | 0.253406665 | -3.218995526 | 0.001286405 | 0.011055941 | down |
| AL590128.1 | 0.590170427 | -0.995822166 | 0.309417098 | -3.218381188 | 0.001289164 | 0.011071115 | down |
| CTRC | 2.230715259 | 0.507409657 | 0.157802758 | 3.215467605 | 0.001302322 | 0.011158326 | up |
| IL37 | 14.93365082 | 0.620935026 | 0.193126012 | 3.215180698 | 0.001303624 | 0.011166624 | up |
| WFDC13 | 3.642024602 | -0.581790592 | 0.181088086 | -3.212749135 | 0.001314711 | 0.011244307 | down |
| IMPA1P1 | 2.311388798 | 0.729178355 | 0.226985686 | 3.21244202 | 0.001316117 | 0.011253457 | up |
| AL450311.1 | 1.184595064 | -0.636551398 | 0.198224979 | -3.211257229 | 0.001321556 | 0.011294186 | down |
| KLRC4-KLRK1 | 2.54847411 | 0.562331079 | 0.175137412 | 3.210799296 | 0.001323663 | 0.011309308 | up |
| AC096637.2 | 1.726385754 | 0.563930118 | 0.175639657 | 3.210722046 | 0.001324019 | 0.011309459 | up |
| KCNK17 | 21.03946541 | -0.503398871 | 0.156791384 | -3.210628401 | 0.001324451 | 0.011310255 | down |
| AL136295.4 | 1.527365521 | 0.525856728 | 0.163856223 | 3.209256986 | 0.001330785 | 0.011352749 | up |
| HYPM | 0.996478108 | -0.965070179 | 0.300890625 | -3.207378689 | 0.001339505 | 0.011406774 | down |
| AC010327.3 | 1.056383892 | 0.848339892 | 0.26455452 | 3.206673214 | 0.001342794 | 0.011426053 | up |
| RPL12P47 | 13.90061598 | -0.876820975 | 0.273506616 | -3.205849228 | 0.001346645 | 0.01145008 | down |
| MARK2P8 | 1.422214334 | -0.818186748 | 0.255279406 | -3.20506366 | 0.001350326 | 0.011468615 | down |
| FAM155B | 111.6962109 | -0.731231176 | 0.228168249 | -3.204789363 | 0.001351614 | 0.011474687 | down |
| AL451069.2 | 1.685225477 | 0.579227703 | 0.18088984 | 3.202101921 | 0.001364287 | 0.01156773 | up |
| MT1M | 105.8468276 | 0.576581624 | 0.180081268 | 3.201785666 | 0.001365786 | 0.011574567 | up |
| STRA6 | 444.1936022 | -0.546868139 | 0.170806368 | -3.201684733 | 0.001366264 | 0.01157569 | down |
| AL121985.3 | 1.406458575 | -0.627028692 | 0.195865382 | -3.201324736 | 0.001367973 | 0.011578081 | down |
| UBE2E1-AS1 | 0.825040045 | 0.642327623 | 0.20069494 | 3.200517279 | 0.001371811 | 0.011599177 | up |
| TPTE2P6 | 0.821139756 | -0.831279695 | 0.259762984 | -3.200146856 | 0.001373576 | 0.01161116 | down |
| AC012498.2 | 4.639987773 | -0.956761241 | 0.29899727 | -3.199899588 | 0.001374755 | 0.011615253 | down |
| AC023421.1 | 9.551446567 | 0.91120732 | 0.284890242 | 3.198450436 | 0.001381683 | 0.011653179 | up |
| AC104964.1 | 1.080941087 | -0.81435983 | 0.254626918 | -3.198247211 | 0.001382657 | 0.011658455 | down |
| AL591846.1 | 190.4240726 | -0.801042444 | 0.250526636 | -3.197434238 | 0.00138656 | 0.011684907 | down |
| CASQ2 | 46.16065213 | -0.738343917 | 0.230921862 | -3.197375554 | 0.001386842 | 0.011684907 | down |
| LRAT | 4.486813903 | 0.642922721 | 0.20109864 | 3.197051557 | 0.001388401 | 0.011692148 | up |
| AL049830.1 | 16.2301209 | -0.804703716 | 0.251836274 | -3.195344747 | 0.001396639 | 0.011743778 | down |
| CLVS2 | 1.837576193 | -0.833049225 | 0.260734965 | -3.195003881 | 0.00139829 | 0.011754701 | down |
| AC005618.2 | 14.91988394 | -0.638728204 | 0.200021123 | -3.193303758 | 0.001406549 | 0.011809389 | down |
| AL122020.1 | 170.104219 | -0.60571699 | 0.189683629 | -3.193301361 | 0.001406561 | 0.011809389 | down |
| AC138123.2 | 10.44873688 | -0.5277929 | 0.165295232 | -3.19303161 | 0.001407875 | 0.01181746 | down |
| PAX7 | 4.715181903 | 1.456515526 | 0.456271326 | 3.192213587 | 0.001411869 | 0.011842064 | up |
| AC009065.1 | 1.796058797 | 0.561987738 | 0.176104298 | 3.191221019 | 0.001416729 | 0.01187389 | up |
| PLA2G4A | 370.651487 | 0.528762728 | 0.165705902 | 3.190970995 | 0.001417955 | 0.01187842 | up |
| IGLV3-12 | 13.35151193 | 0.767261681 | 0.240492815 | 3.190372569 | 0.001420895 | 0.011896882 | up |
| MALRD1 | 109.8031341 | 0.778019188 | 0.244174327 | 3.186326745 | 0.001440918 | 0.012025387 | up |
| SLN | 10.78253105 | 0.662817864 | 0.208076026 | 3.185460032 | 0.001445241 | 0.012055448 | up |
| AL138785.1 | 120.8553177 | -0.751232687 | 0.236023666 | -3.182870178 | 0.00145823 | 0.012154702 | down |
| FBXO39 | 5.795710657 | 0.52037514 | 0.163524896 | 3.182238016 | 0.001461417 | 0.012166103 | up |
| RPS6P25 | 79.44644447 | -0.540931949 | 0.170007323 | -3.181815578 | 0.00146355 | 0.012174768 | down |
| SAMD7 | 0.746437806 | 0.927831085 | 0.291703372 | 3.180734857 | 0.00146902 | 0.012210762 | up |
| GDAP1L1 | 5.429185368 | -0.51351581 | 0.161599748 | -3.177701798 | 0.001484473 | 0.012315127 | down |
| RPS3P6 | 27.85056914 | -0.587875929 | 0.185093796 | -3.176097418 | 0.001492708 | 0.0123681 | down |
| SLC26A3 | 5192.498253 | -0.699527433 | 0.220287804 | -3.175515934 | 0.001495703 | 0.012386777 | down |
| HYAL1 | 426.7409577 | 0.551665583 | 0.173814961 | 3.173867076 | 0.001504226 | 0.012432726 | up |
| APOC4-APOC2 | 3.369420211 | -0.549231267 | 0.173112425 | -3.172685417 | 0.001510361 | 0.012468649 | down |
| LINC01506 | 3.07761287 | -0.544976173 | 0.171772035 | -3.172671118 | 0.001510435 | 0.012468649 | down |
| CNMD | 0.978600136 | -0.693129059 | 0.218498684 | -3.172234482 | 0.001512708 | 0.012483846 | down |
| ALAS2 | 5.867987932 | 0.585803395 | 0.18469135 | 3.171796601 | 0.001514991 | 0.01249391 | up |
| AC010547.2 | 9.100813978 | 0.998421591 | 0.314852353 | 3.171078702 | 0.00151874 | 0.012515569 | up |
| AD000671.2 | 0.816014765 | 0.59391124 | 0.187302893 | 3.17085994 | 0.001519884 | 0.012521912 | up |
| FAM201B | 1.399823041 | -0.586231342 | 0.184897753 | -3.170570403 | 0.0015214 | 0.012531311 | down |
| WFDC10A | 3.872444662 | -0.626290511 | 0.19756009 | -3.170126667 | 0.001523725 | 0.012544285 | down |
| AC092135.3 | 4.420917143 | -0.532661503 | 0.168034048 | -3.169961731 | 0.00152459 | 0.012545231 | down |
| TCL1A | 19.17714547 | 0.775174538 | 0.24476635 | 3.166997989 | 0.001540214 | 0.012648893 | up |
| AC009509.1 | 26.65539365 | 0.59859105 | 0.189031907 | 3.166613831 | 0.00154225 | 0.012662503 | up |
| OLFM3 | 2.490566725 | 0.810410529 | 0.256023497 | 3.165375596 | 0.001548829 | 0.01270716 | up |
| MT-TV | 8.818299315 | -0.7902497 | 0.249673596 | -3.16513124 | 0.00155013 | 0.012714717 | down |
| CEACAMP1 | 0.946396742 | -0.751064464 | 0.237371708 | -3.164085849 | 0.001555709 | 0.01275422 | down |
| FOXB1 | 2.203538776 | 0.691079774 | 0.218631093 | 3.160940038 | 0.001572609 | 0.012858095 | up |
| ERICH5 | 54.42049634 | -0.551972473 | 0.174673137 | -3.160030683 | 0.001577525 | 0.012887811 | down |
| ELOVL3 | 9.457412764 | 0.504578427 | 0.159682667 | 3.159882255 | 0.001578329 | 0.012887811 | up |
| AC018992.1 | 3.498116515 | -0.650598783 | 0.205901656 | -3.159754985 | 0.001579019 | 0.012888446 | down |
| LINC01738 | 8.418087678 | -0.530629183 | 0.167950403 | -3.159439778 | 0.001580728 | 0.0128961 | down |
| GABRG3 | 1.065892459 | -1.025324563 | 0.324570552 | -3.159019069 | 0.001583011 | 0.012903829 | down |
| CHRM2 | 30.94766601 | -0.942211563 | 0.298563726 | -3.155813921 | 0.001600509 | 0.013016204 | down |
| SEPHS1P4 | 4.714477757 | -0.657981874 | 0.208659618 | -3.153374282 | 0.001613947 | 0.013106365 | down |
| ASB4 | 20.39889585 | 0.699816319 | 0.22199238 | 3.152433968 | 0.001619154 | 0.01312952 | up |
| FHP1 | 3.81371448 | -0.563332575 | 0.178808071 | -3.15048741 | 0.001629983 | 0.013201321 | down |
| TUBB8 | 2.377069453 | 0.606267415 | 0.19261876 | 3.147499314 | 0.001646735 | 0.013302681 | up |
| AC009145.4 | 1.632205416 | -0.710610767 | 0.225771716 | -3.147474707 | 0.001646874 | 0.013302681 | down |
| UPK1A | 8.907686645 | -0.706846448 | 0.224728433 | -3.145336073 | 0.001658961 | 0.01339385 | down |
| RNA5-8SP2 | 1.574572452 | -1.739607396 | 0.553227454 | -3.144470479 | 0.001663877 | 0.013420576 | down |
| KCNE1 | 5.229600744 | 0.543282858 | 0.172890621 | 3.14235009 | 0.001675975 | 0.01351164 | up |
| IGHV3-43 | 93.36154065 | 0.683381117 | 0.217601856 | 3.140511428 | 0.001686531 | 0.013582636 | up |
| ZNF365 | 12.20257664 | -0.532675088 | 0.169654565 | -3.139762773 | 0.001690847 | 0.01360918 | down |
| AL133383.2 | 1.678124197 | -0.924452183 | 0.294434998 | -3.139749657 | 0.001690923 | 0.01360918 | down |
| AC092167.1 | 0.711136009 | 0.858962389 | 0.273673287 | 3.138641693 | 0.001697329 | 0.013654165 | up |
| AC008667.3 | 0.738012927 | 0.780523215 | 0.248781029 | 3.137390411 | 0.00170459 | 0.013690632 | up |
| AC012508.1 | 0.951125015 | -0.828840415 | 0.264183464 | -3.137366749 | 0.001704727 | 0.013690632 | down |
| LINC01168 | 1.778577476 | 0.962144909 | 0.306922751 | 3.134811298 | 0.001719647 | 0.013790578 | up |
| AC084026.2 | 1.821207897 | 0.687445614 | 0.219536994 | 3.131342931 | 0.001740088 | 0.013931122 | up |
| FEZF2 | 2.662650725 | -1.147022294 | 0.366323067 | -3.131176819 | 0.001741073 | 0.013935668 | down |
| RN7SL329P | 0.884270085 | -1.36324792 | 0.435523709 | -3.130134803 | 0.001747261 | 0.013978628 | down |
| AL163636.2 | 7.670686389 | 0.679894337 | 0.21723131 | 3.129817419 | 0.00174915 | 0.013986925 | up |
| RN7SL8P | 5.699203896 | -0.681525789 | 0.217862758 | -3.128234465 | 0.001758598 | 0.014055757 | down |
| LINC01819 | 316.5707932 | -0.858535755 | 0.274548456 | -3.127082804 | 0.001765502 | 0.014100824 | down |
| AC112206.1 | 1.83571662 | -1.004926163 | 0.321464297 | -3.126089495 | 0.001771476 | 0.01413841 | down |
| AC016168.3 | 0.752023106 | 1.067000915 | 0.34137583 | 3.1255901 | 0.001774487 | 0.01415906 | up |
| CYCSP55 | 3.714160809 | -0.567519848 | 0.181579456 | -3.125462878 | 0.001775255 | 0.014161807 | down |
| AC087481.1 | 1.532314093 | 0.557220091 | 0.178305102 | 3.125093371 | 0.001777486 | 0.014172847 | up |
| AC006141.1 | 1.068085174 | -0.937102417 | 0.300191927 | -3.121677611 | 0.001798237 | 0.014314415 | down |
| GFY | 1.500219342 | 0.947505911 | 0.303577818 | 3.121130249 | 0.001801583 | 0.014337636 | up |
| MT-TT | 8.247147186 | 0.527226903 | 0.168982254 | 3.120013435 | 0.001808428 | 0.014378421 | up |
| AC016717.2 | 2.525570305 | 0.877484328 | 0.281284645 | 3.119560003 | 0.001811214 | 0.014393725 | up |
| AC017074.2 | 0.901776082 | -0.67943903 | 0.217842241 | -3.118949873 | 0.001814968 | 0.014416712 | down |
| AC015813.7 | 1.596821274 | -0.590837066 | 0.189482635 | -3.118159443 | 0.001819843 | 0.014441713 | down |
| AL158064.1 | 1.119071584 | 0.863537227 | 0.276981161 | 3.11767495 | 0.001822837 | 0.014455326 | up |
| AC124856.1 | 2.423889317 | -0.756488841 | 0.242729773 | -3.116588593 | 0.001829567 | 0.014491367 | down |
| AC010200.1 | 2.343488799 | -0.516655706 | 0.165792484 | -3.11627942 | 0.001831486 | 0.014503135 | down |
| B4GALNT4 | 371.4405447 | -0.61482689 | 0.19730263 | -3.116161655 | 0.001832218 | 0.014505493 | down |
| SRP72P2 | 4.492145097 | -0.514331033 | 0.165076237 | -3.115718177 | 0.001834975 | 0.014520449 | down |
| AC087072.1 | 3.56281346 | -0.803699711 | 0.257977675 | -3.11538474 | 0.001837051 | 0.014529998 | down |
| CDK8P2 | 1.047668974 | 0.670004057 | 0.215079403 | 3.115147465 | 0.00183853 | 0.014534816 | up |
| KRT89P | 2.319500976 | 0.87364507 | 0.28049139 | 3.114694789 | 0.001841353 | 0.014549418 | up |
| MYH11 | 6315.054082 | -0.559741311 | 0.17971288 | -3.114642143 | 0.001841682 | 0.014549418 | down |
| ASB9P1 | 2.011243902 | -0.533160163 | 0.171218446 | -3.113917776 | 0.001846209 | 0.014570136 | down |
| ADH5P2 | 0.912564476 | -0.92879198 | 0.298358873 | -3.113002707 | 0.001851943 | 0.014587426 | down |
| AC005035.1 | 6.481173634 | 0.590288607 | 0.189622473 | 3.112967552 | 0.001852164 | 0.014587426 | up |
| KIR3DL1 | 2.076428674 | 0.907798389 | 0.291627974 | 3.112864576 | 0.00185281 | 0.01458908 | up |
| SPDEF | 872.6395726 | 0.538757022 | 0.173084889 | 3.112675092 | 0.001854 | 0.014591433 | up |
| LINC00261 | 1373.730828 | 0.589995317 | 0.189548685 | 3.112632083 | 0.00185427 | 0.014591433 | up |
| KRT6B | 437.0940912 | 0.656871409 | 0.211052244 | 3.112364017 | 0.001855955 | 0.014596658 | up |
| KLC3 | 40.48230434 | 0.547515026 | 0.175928275 | 3.112149119 | 0.001857307 | 0.014603853 | up |
| VWC2 | 5.39649475 | -0.559680747 | 0.179852779 | -3.111882677 | 0.001858984 | 0.014613604 | down |
| AC112236.1 | 0.929498577 | 0.950513521 | 0.30552427 | 3.111090068 | 0.001863981 | 0.014642561 | up |
| RPS29P8 | 3.203167212 | -0.88095262 | 0.28318777 | -3.110842747 | 0.001865543 | 0.014651388 | down |
| MTCO1P53 | 8.910836482 | 0.600982644 | 0.19326518 | 3.109627121 | 0.001873237 | 0.014702388 | up |
| AC087283.1 | 1.149921304 | -0.950446926 | 0.305739412 | -3.108683045 | 0.001879232 | 0.014731696 | down |
| H19 | 1483.079683 | 0.64302836 | 0.206868128 | 3.108397438 | 0.001881049 | 0.014738558 | up |
| BDH2P1 | 2.200252421 | -0.628445922 | 0.202194368 | -3.108127728 | 0.001882767 | 0.014744402 | down |
| AC025265.3 | 3.93936105 | 0.623368626 | 0.200570357 | 3.107979832 | 0.001883709 | 0.014744402 | up |
| ADRA1A | 2.316519303 | 0.615883621 | 0.198264375 | 3.106375626 | 0.00189396 | 0.014811951 | up |
| AC067942.1 | 20.64328898 | -0.579438531 | 0.186570272 | -3.105738795 | 0.001898043 | 0.014840415 | down |
| MYL3 | 7.933369806 | -0.639378488 | 0.205891507 | -3.105414585 | 0.001900125 | 0.014849749 | down |
| AC026774.1 | 2.403540904 | -1.038656799 | 0.33448562 | -3.105236034 | 0.001901272 | 0.014855245 | down |
| HPN | 132.7304434 | -0.709404132 | 0.228466111 | -3.105073788 | 0.001902316 | 0.014856454 | down |
| RPL30P2 | 1.264654954 | -0.745081449 | 0.239995935 | -3.104558619 | 0.001905632 | 0.014868704 | down |
| FCER2 | 14.01881249 | 0.631874604 | 0.203536061 | 3.104484781 | 0.001906107 | 0.014868704 | up |
| CYP4X1 | 388.0326523 | 0.539441983 | 0.173904649 | 3.101941132 | 0.001922562 | 0.014962158 | up |
| SALL3 | 1.501017359 | -1.709646935 | 0.551392134 | -3.100600879 | 0.001931284 | 0.015012569 | down |
| AC104984.1 | 1.297307212 | -0.711071687 | 0.229384767 | -3.099908054 | 0.001935807 | 0.015040737 | down |
| CXCL9 | 1247.534538 | 0.504263948 | 0.162777008 | 3.097881913 | 0.001949091 | 0.015103126 | up |
| LMO1 | 1.013708143 | 0.973754901 | 0.314331811 | 3.097856683 | 0.001949257 | 0.015103126 | up |
| AL139317.2 | 2.698800561 | -0.996956178 | 0.321898487 | -3.097113585 | 0.00195415 | 0.015130522 | down |
| AP000943.2 | 1.361206302 | 1.16496357 | 0.376306244 | 3.095785919 | 0.00196292 | 0.015177345 | up |
| TLX3 | 3.226664805 | -1.108089448 | 0.358038726 | -3.094887135 | 0.001968878 | 0.015212698 | down |
| AF127577.2 | 0.737547368 | -0.937804208 | 0.303045867 | -3.094594947 | 0.001970818 | 0.015213889 | down |
| AC006946.2 | 2.563860506 | 0.7056255 | 0.22815015 | 3.0928119 | 0.001982698 | 0.015291373 | up |
| AC011492.1 | 2.780116259 | -0.829437007 | 0.268303756 | -3.091410346 | 0.001992081 | 0.015353152 | down |
| AC012513.2 | 1.013987075 | -0.717971454 | 0.23253677 | -3.087560961 | 0.002018064 | 0.015511322 | down |
| PRSS56 | 39.032843 | 1.085473077 | 0.351581813 | 3.087398261 | 0.002019169 | 0.015511322 | up |
| DPP6 | 11.92638504 | -0.614111782 | 0.199019667 | -3.0856839 | 0.002030846 | 0.015590799 | down |
| AC012508.2 | 0.906034547 | -0.780518882 | 0.253332554 | -3.081005061 | 0.002063031 | 0.015787147 | down |
| DDC-AS1 | 3.246267437 | -0.735379461 | 0.238838116 | -3.078987024 | 0.002077057 | 0.01585819 | down |
| AL359740.1 | 1.729429818 | -0.628598665 | 0.204249679 | -3.077599285 | 0.002086753 | 0.015906795 | down |
| SPATA13-AS1 | 0.49861559 | -1.073502279 | 0.348821124 | -3.077515111 | 0.002087343 | 0.015907662 | down |
| AL662899.1 | 2.708222374 | -0.612626392 | 0.199082087 | -3.077255217 | 0.002089163 | 0.015917912 | down |
| AL353662.2 | 26.33697333 | -0.610638956 | 0.19857582 | -3.075092208 | 0.002104375 | 0.015997363 | down |
| AP000560.1 | 3.484184168 | -0.504692103 | 0.164156935 | -3.074448875 | 0.002108919 | 0.016020979 | down |
| GAPDHP35 | 62.88804079 | 0.502759916 | 0.16361709 | 3.072783627 | 0.002120722 | 0.016077774 | up |
| RPS20P22 | 2.073796234 | 0.56519187 | 0.183964523 | 3.072287319 | 0.002124251 | 0.016097234 | up |
| RPL10P12 | 4.488955031 | -0.504634301 | 0.164263236 | -3.072107381 | 0.002125532 | 0.016103292 | down |
| WDR72 | 597.2756781 | 0.548626393 | 0.17867761 | 3.070482038 | 0.002137135 | 0.016172031 | up |
| ALDOB | 1447.285391 | 0.564805821 | 0.183949372 | 3.070441696 | 0.002137424 | 0.016172031 | up |
| IGHV1-69 | 193.0946746 | -0.699739516 | 0.227968467 | -3.069457476 | 0.002144479 | 0.016209852 | down |
| FOXN4 | 1.889705208 | 0.921509392 | 0.300465829 | 3.066935749 | 0.002162653 | 0.016311954 | up |
| AHCYP4 | 1.465536099 | -0.875658163 | 0.285619683 | -3.065818696 | 0.002170749 | 0.016364163 | down |
| SLITRK4 | 9.722565678 | -0.614837361 | 0.200550147 | -3.065753727 | 0.002171221 | 0.016364163 | down |
| RNU5A-1 | 4.288388098 | -1.230406741 | 0.401638783 | -3.063465962 | 0.002187891 | 0.016460126 | down |
| RPS28P7 | 1817.903439 | 0.517086328 | 0.16884037 | 3.062575195 | 0.002194413 | 0.016501771 | up |
| ANXA8L1 | 4.905032175 | 0.503093669 | 0.164328013 | 3.06152103 | 0.002202155 | 0.016541391 | up |
| UGT1A6 | 46.12740037 | 0.507542517 | 0.165801029 | 3.061154202 | 0.002204855 | 0.016554234 | up |
| SNX6P1 | 1.034811571 | -0.834196096 | 0.272635611 | -3.059747375 | 0.002215238 | 0.016602367 | down |
| MORN5 | 3.950373106 | -0.920352139 | 0.30080843 | -3.059595566 | 0.002216361 | 0.016607062 | down |
| POLR3GP1 | 1.120126869 | 0.607542048 | 0.198619138 | 3.058829354 | 0.002222037 | 0.016634684 | up |
| ALX3 | 0.551157181 | -2.066788967 | 0.67575687 | -3.05848014 | 0.002224628 | 0.016648144 | down |
| AC004009.3 | 0.420908 | 1.383664 | 0.4528433 | 3.055503 | 0.002246831 | NA | up |
| AC145207.1 | 3.431677476 | -0.534107513 | 0.174837317 | -3.054882801 | 0.002251485 | 0.016836294 | down |
| CHST4 | 135.6912612 | 0.793523318 | 0.259839945 | 3.053892727 | 0.002258928 | 0.016869703 | up |
| AL121760.1 | 1.199499972 | -0.616513528 | 0.201993491 | -3.052145511 | 0.002272119 | 0.016945535 | down |
| CXCL8 | 3875.973033 | -0.542086086 | 0.177632743 | -3.051723898 | 0.002275313 | 0.016957597 | down |
| AL109741.3 | 0.861845295 | 0.564869048 | 0.185190972 | 3.050197534 | 0.002286909 | 0.01700612 | up |
| RNU1-11P | 0.62250443 | -1.822073003 | 0.59745696 | -3.049714247 | 0.002290592 | 0.017025936 | down |
| AC009533.2 | 1.423475502 | 0.592151537 | 0.194182217 | 3.049463267 | 0.002292507 | 0.017032596 | up |
| LINC01649 | 0.947484216 | 0.792328655 | 0.25988789 | 3.048732494 | 0.00229809 | 0.017066496 | up |
| AC037198.3 | 13.10371774 | -0.64948718 | 0.213044518 | -3.048598419 | 0.002299116 | 0.01706712 | down |
| CLIC4P3 | 1.898208198 | 0.591149053 | 0.193943101 | 3.048054045 | 0.002303285 | 0.017087628 | up |
| COLCA1 | 326.6344596 | 0.555824004 | 0.182357455 | 3.04799168 | 0.002303763 | 0.017087628 | up |
| PCDHGB5 | 51.06339073 | -0.543320422 | 0.178299494 | -3.047234793 | 0.002309572 | 0.017125146 | down |
| AC015802.1 | 1.219640224 | 0.644842902 | 0.211751279 | 3.04528457 | 0.002324602 | 0.017221318 | up |
| HIST1H4B | 3.87775274 | -0.632139701 | 0.207723985 | -3.043171456 | 0.002340989 | 0.017315863 | down |
| AC003005.2 | 1.11611724 | 0.632927877 | 0.20803292 | 3.042440963 | 0.002346678 | 0.017354107 | up |
| KLHL34 | 18.47502013 | -0.720154002 | 0.23678831 | -3.04134103 | 0.002355269 | 0.017399663 | down |
| AC083805.3 | 1.164129146 | -0.685369515 | 0.225545963 | -3.038713286 | 0.002375909 | 0.017512179 | down |
| REG1CP | 0.932187391 | 1.327854535 | 0.437368903 | 3.03600582 | 0.002397347 | 0.017629549 | up |
| AL773572.1 | 5.573893547 | -0.680780052 | 0.224617401 | -3.030842888 | 0.002438721 | 0.017853168 | down |
| AC005865.1 | 4.960756193 | 0.526426312 | 0.173720718 | 3.030302415 | 0.00244309 | 0.017877323 | up |
| LINC00707 | 1.406198092 | 0.714232371 | 0.23578849 | 3.029123143 | 0.002452647 | 0.017919809 | up |
| AMER3 | 5.565792447 | -0.856467135 | 0.283007903 | -3.026301123 | 0.002475656 | 0.018056362 | down |
| CLDN2 | 6003.401226 | 0.538289744 | 0.178065387 | 3.022989209 | 0.002502912 | 0.01821146 | up |
| RET | 125.3833132 | 0.536969026 | 0.177695627 | 3.021847164 | 0.002512374 | 0.018268382 | up |
| AC011511.3 | 5.458083856 | -0.625710035 | 0.207113975 | -3.021090374 | 0.002518662 | 0.018308392 | down |
| AC090204.1 | 24.24769715 | -0.53134262 | 0.175978203 | -3.019366105 | 0.002533042 | 0.018386687 | down |
| OXT | 2.860158631 | -0.553142519 | 0.18329507 | -3.017770852 | 0.002546414 | 0.018467712 | down |
| LINC02404 | 1.951089017 | 1.307887736 | 0.433435549 | 3.017490693 | 0.002548769 | 0.018480783 | up |
| AC104964.2 | 2.642883012 | -0.52976662 | 0.175576336 | -3.01730081 | 0.002550366 | 0.018488356 | down |
| AC093063.1 | 1.819877823 | 0.853428123 | 0.283025431 | 3.015376112 | 0.002566608 | 0.018581928 | up |
| ESPNL | 38.36887548 | -0.504630889 | 0.167439326 | -3.013813435 | 0.002579864 | 0.018649639 | down |
| RBP1 | 765.0457578 | -0.503277724 | 0.167000209 | -3.013635303 | 0.002581379 | 0.018656559 | down |
| AC004870.2 | 2.757820079 | 0.897689932 | 0.298120779 | 3.011161901 | 0.0026025 | 0.018776746 | up |
| SNORA80D | 1.547844168 | -0.576876966 | 0.19174388 | -3.008580844 | 0.00262471 | 0.01888968 | down |
| SNORD3B-2 | 1.805946948 | -0.852987521 | 0.283579207 | -3.007933944 | 0.002630303 | 0.018912284 | down |
| ZNF603P | 0.55858172 | 0.982107678 | 0.326506135 | 3.007930245 | 0.002630335 | 0.018912284 | up |
| KRT14 | 13.98317793 | 0.958770189 | 0.318851226 | 3.006951553 | 0.002638818 | 0.018956975 | up |
| AC004832.6 | 1.967324517 | 0.53246898 | 0.177100383 | 3.006594175 | 0.002641922 | 0.018971122 | up |
| TFF1 | 5402.62209 | 0.608234018 | 0.202367015 | 3.005598606 | 0.002650586 | 0.019012926 | up |
| IQCF1 | 0.942415565 | 0.774776538 | 0.258000489 | 3.003004149 | 0.002673288 | 0.019141737 | up |
| AL645608.2 | 1.662303954 | 0.500593829 | 0.166703875 | 3.002892587 | 0.002674268 | 0.019141737 | up |
| CLCA1 | 7731.317982 | 0.800522224 | 0.266603647 | 3.002667941 | 0.002676243 | 0.019147675 | up |
| BBOX1 | 3.739898476 | 0.593855324 | 0.197864523 | 3.001322906 | 0.002688093 | 0.019216018 | up |
| ZNF840P | 0.770575906 | -1.366893686 | 0.455915592 | -2.998128841 | 0.002716428 | 0.019385419 | down |
| AC132812.2 | 5.984633458 | -0.688281761 | 0.229602311 | -2.997712694 | 0.00272014 | 0.019399487 | down |
| LINC02422 | 1.292950847 | 0.75652957 | 0.252612425 | 2.994823275 | 0.002746039 | 0.019561512 | up |
| AQP7P1 | 3.71663841 | -0.569835492 | 0.190342598 | -2.993736015 | 0.002755843 | 0.019608114 | down |
| LINC02033 | 2.064169952 | 0.501729019 | 0.167666002 | 2.992431459 | 0.002767648 | 0.019667004 | up |
| AC241520.1 | 0.501030235 | -1.386032138 | 0.463560182 | -2.989972373 | 0.002790027 | 0.019780104 | down |
| TUBA3C | 0.668032456 | 1.366125565 | 0.457040513 | 2.989068861 | 0.002798291 | 0.019829971 | up |
| CCDC92B | 0.91834829 | 0.891594307 | 0.298353021 | 2.988387063 | 0.002804541 | 0.019857435 | up |
| SNORA38 | 0.610449358 | -1.860538993 | 0.622741664 | -2.987657806 | 0.002811241 | 0.01990066 | down |
| TUBB8P11 | 1.072221101 | -0.804714512 | 0.269427022 | -2.986762448 | 0.002819487 | 0.019916872 | down |
| RNY4P19 | 0.784149244 | 0.636631971 | 0.213401967 | 2.983252585 | 0.002852025 | 0.020093216 | up |
| SFTPC | 1.486178672 | 1.314590776 | 0.440660634 | 2.983227171 | 0.002852262 | 0.020093216 | up |
| TFPI2 | 99.35947814 | -0.517463499 | 0.173467437 | -2.983058433 | 0.002853835 | 0.020097294 | down |
| CELF3 | 61.51928423 | -0.569996105 | 0.191079182 | -2.983036136 | 0.002854043 | 0.020097294 | down |
| ARPC3P2 | 3.461466873 | -0.570546305 | 0.191301355 | -2.982447794 | 0.002859534 | 0.020115715 | down |
| LRRTM4 | 3.536714168 | 0.662911631 | 0.222275524 | 2.982386988 | 0.002860102 | 0.020115715 | up |
| NMNAT2 | 92.70390536 | -0.526303771 | 0.17664719 | -2.979406419 | 0.002888074 | 0.020281414 | down |
| CALHM1 | 2.504998098 | 0.619281046 | 0.208039811 | 2.976742982 | 0.002913281 | 0.0204284 | up |
| LINC00545 | 1.975931386 | -0.78211148 | 0.262788718 | -2.976198845 | 0.002918456 | 0.020450755 | down |
| IPO7P1 | 1.576301857 | -0.72125211 | 0.242359204 | -2.975963354 | 0.002920698 | 0.020450755 | down |
| GP9 | 3.616314145 | 0.66427481 | 0.223333777 | 2.974358914 | 0.002936014 | 0.020544728 | up |
| AC006058.4 | 1.356175052 | 0.796808751 | 0.267932032 | 2.973921201 | 0.002940206 | 0.020569753 | up |
| AL512625.2 | 2.777310815 | -0.553908232 | 0.186296599 | -2.973260023 | 0.002946547 | 0.020591258 | down |
| PSCA | 47.39390706 | 0.667679012 | 0.224579145 | 2.973023222 | 0.002948821 | 0.020591258 | up |
| AL772337.3 | 1.489964716 | -0.858085086 | 0.288811354 | -2.971091937 | 0.002967429 | 0.020694287 | down |
| AL357078.1 | 0.735176813 | -0.639777439 | 0.215335217 | -2.971076672 | 0.002967577 | 0.020694287 | down |
| AL355309.1 | 18.48394346 | -0.706029111 | 0.237781932 | -2.969229435 | 0.002985476 | 0.020786446 | down |
| AL353752.1 | 0.448746448 | 1.223471843 | 0.412166216 | 2.968394289 | 0.002993601 | 0.020816307 | up |
| ATP8A2P2 | 1.213555007 | -0.573657783 | 0.193262896 | -2.968276856 | 0.002994745 | 0.020816307 | down |
| CCT4P1 | 0.701673612 | -1.147514591 | 0.386631786 | -2.967977885 | 0.002997659 | 0.020832235 | down |
| CDH22 | 4.240084334 | -0.822604744 | 0.277275717 | -2.966739217 | 0.003009761 | 0.020906099 | down |
| GLRA4 | 9.668179584 | -0.50175982 | 0.169216051 | -2.965202273 | 0.00302484 | 0.020968836 | down |
| FAM41C | 1.345427058 | -0.878382251 | 0.296351438 | -2.963988486 | 0.003036796 | 0.021034282 | down |
| RPRML | 3.015543181 | -0.866777646 | 0.29260703 | -2.962258447 | 0.003053913 | 0.021130957 | down |
| NUTM1 | 2.990610811 | 0.57768303 | 0.195100436 | 2.960952021 | 0.003066897 | 0.021185727 | up |
| RPL35P3 | 3.272519745 | -0.547129022 | 0.184815933 | -2.960399647 | 0.003072402 | 0.02121061 | down |
| AL589647.1 | 3.914377016 | -0.645571016 | 0.218124196 | -2.959648809 | 0.003079899 | 0.021249207 | down |
| GUCA2A | 533.5893671 | -0.582138482 | 0.196722981 | -2.959178838 | 0.0030846 | 0.021269295 | down |
| AC012507.2 | 0.910877101 | 0.641092699 | 0.216672039 | 2.958816015 | 0.003088234 | 0.021280371 | up |
| EVX2 | 2.342787987 | -0.6845775 | 0.231508076 | -2.957035069 | 0.003106128 | 0.021377243 | down |
| AC004543.1 | 0.567166177 | 0.689897431 | 0.23337857 | 2.956130169 | 0.003115256 | 0.021435652 | up |
| LINC00327 | 2.394082125 | 0.550241018 | 0.186143107 | 2.956010711 | 0.003116463 | 0.021439545 | up |
| AC012499.1 | 1.526432135 | 0.837485667 | 0.28344031 | 2.954716167 | 0.003129568 | 0.021512002 | up |
| SLC18A1 | 89.39593973 | 0.642781089 | 0.217548873 | 2.954651426 | 0.003130225 | 0.021512095 | up |
| RNU1-134P | 1.121392958 | -0.695868094 | 0.235593483 | -2.953681422 | 0.00314008 | 0.021561615 | down |
| SNORD11B | 0.546501431 | -0.909949582 | 0.308078919 | -2.953624947 | 0.003140654 | 0.021561615 | down |
| GNAS-AS1 | 4.733083097 | -0.628799249 | 0.212919403 | -2.953226618 | 0.00314471 | 0.021580597 | down |
| GACAT3 | 1.349497885 | 0.837461043 | 0.283680603 | 2.95212656 | 0.003155936 | 0.021653188 | up |
| LINC01940 | 2.099354816 | 1.205206986 | 0.408361587 | 2.951323107 | 0.003164157 | 0.021700694 | up |
| DDX25 | 2.015864889 | 0.585754302 | 0.198646417 | 2.948728252 | 0.003190844 | 0.02183949 | up |
| AC099535.1 | 3.379484162 | -0.608727665 | 0.206471218 | -2.948244653 | 0.00319584 | 0.021864177 | down |
| RTL3 | 1.903247748 | -0.600268641 | 0.203709883 | -2.946683935 | 0.003212013 | 0.021947882 | down |
| RNU5A-8P | 0.615238587 | -1.499278548 | 0.508960917 | -2.945763611 | 0.003221585 | 0.021990819 | down |
| RPS3P7 | 5.78818547 | -0.59373014 | 0.201640633 | -2.94449651 | 0.003234806 | 0.022049561 | down |
| AC016894.1 | 2.132310239 | -0.626168012 | 0.212691154 | -2.944024711 | 0.003239741 | 0.022074203 | down |
| AC090150.1 | 0.871724693 | -0.979755996 | 0.333081465 | -2.941490589 | 0.003266368 | 0.0222375 | down |
| SAPCD2P1 | 0.673966532 | -1.130731301 | 0.384546379 | -2.940428938 | 0.003277582 | 0.022291155 | down |
| RPS8P4 | 9.015032436 | -0.644119413 | 0.219112726 | -2.939671396 | 0.003285605 | 0.022323022 | down |
| GBP7 | 4.550655853 | 0.552542941 | 0.188078671 | 2.937828826 | 0.003305195 | 0.022429282 | up |
| SPATA4 | 0.482413405 | -0.775759517 | 0.264235177 | -2.935867684 | 0.003326162 | 0.022529911 | down |
| TRIM72 | 180.6285245 | 0.781510432 | 0.266239715 | 2.935363835 | 0.003331568 | 0.022552827 | up |
| Z75746.1 | 0.3686086 | 0.7002426 | 0.2386978 | 2.933595 | 0.003350614 | NA | up |
| RN7SL827P | 0.704368563 | -0.960868301 | 0.327720719 | -2.931973003 | 0.00336816 | 0.022732116 | down |
| AC005740.2 | 1.373862216 | -0.861404672 | 0.293930498 | -2.930640668 | 0.003382638 | 0.022810745 | down |
| AC027338.1 | 0.949596366 | 0.578567206 | 0.197424204 | 2.930578889 | 0.003383311 | 0.022810745 | up |
| BX842568.2 | 8.193418043 | -0.534623733 | 0.182485759 | -2.929673717 | 0.003393181 | 0.022866664 | down |
| ADGB | 8.973159423 | -0.599592159 | 0.204724428 | -2.928776822 | 0.003402987 | 0.022920297 | down |
| RN7SL125P | 0.576300092 | -0.993287641 | 0.339175537 | -2.928535621 | 0.003405628 | 0.022927538 | down |
| AC012676.2 | 2.006319728 | 0.743798689 | 0.253987028 | 2.928490855 | 0.003406119 | 0.022927538 | up |
| RNU1-13P | 0.617979636 | -1.495192946 | 0.510841651 | -2.926920587 | 0.003423363 | 0.023001944 | down |
| PEG10 | 149.036137 | -0.612423762 | 0.209323323 | -2.925731123 | 0.003436478 | 0.023071524 | down |
| AL117382.2 | 1.800760808 | -0.555136356 | 0.189752577 | -2.925580056 | 0.003438147 | 0.023072955 | down |
| AC104794.3 | 4.751616153 | 0.64938241 | 0.222136342 | 2.923350603 | 0.003462864 | 0.023192608 | up |
| PCAT14 | 30.09073246 | 0.851426499 | 0.291354712 | 2.922302142 | 0.003474543 | 0.023257044 | up |
| AC004593.1 | 13.10527298 | -0.556011831 | 0.190299974 | -2.921765137 | 0.003480539 | 0.023273886 | down |
| SLC9A4 | 5.420466741 | 0.617910815 | 0.211600906 | 2.920170941 | 0.003498394 | 0.02335592 | up |
| LINC01952 | 1.401922787 | -0.506382431 | 0.173465907 | -2.919204353 | 0.003509261 | 0.02341595 | down |
| AC024884.1 | 0.706320409 | 0.603122204 | 0.206606415 | 2.919184309 | 0.003509487 | 0.02341595 | up |
| AC087257.1 | 0.752286597 | -1.129137081 | 0.386841734 | -2.91886056 | 0.003513133 | 0.02342626 | down |
| FP671120.3 | 0.895949063 | -1.29048488 | 0.442220116 | -2.918195786 | 0.003520632 | 0.023462228 | down |
| DRC1 | 4.078798828 | -0.507300365 | 0.173903984 | -2.917129064 | 0.003532696 | 0.02352387 | down |
| CYP21A2 | 2.424357535 | 0.577774462 | 0.198094707 | 2.916657741 | 0.003538038 | 0.023531328 | up |
| KCNJ12 | 75.48490934 | -0.583313243 | 0.200027356 | -2.91616734 | 0.003543604 | 0.023558977 | down |
| RNU4-62P | 0.877488503 | 0.635161691 | 0.217907703 | 2.91481982 | 0.003558939 | 0.023651529 | up |
| LINC02303 | 0.814249929 | 1.355327687 | 0.465093462 | 2.914097482 | 0.003567185 | 0.023687497 | up |
| HLA-DPB2 | 21.67677443 | 0.530893382 | 0.182255869 | 2.912901432 | 0.003580876 | 0.023759539 | up |
| AP000812.3 | 1.966090384 | 0.997631581 | 0.342522746 | 2.912599506 | 0.00358434 | 0.023777803 | up |
| SFRP4 | 1473.367025 | -0.530213916 | 0.182058629 | -2.912325101 | 0.00358749 | 0.023784549 | down |
| AC011890.1 | 1.267128614 | -0.804049084 | 0.276107507 | -2.912087011 | 0.003590226 | 0.023797969 | down |
| PCDH8 | 6.217207235 | -0.767625098 | 0.263616918 | -2.911896192 | 0.00359242 | 0.023807792 | down |
| LINC01016 | 1.861271605 | -0.558971114 | 0.192014245 | -2.911091908 | 0.003601681 | 0.023852447 | down |
| AC018695.1 | 32.95176913 | -0.847031637 | 0.291029535 | -2.910466242 | 0.0036089 | 0.02389333 | down |
| AC068658.1 | 2.891116033 | 0.589217744 | 0.202506647 | 2.909621741 | 0.003618664 | 0.023943758 | up |
| AL583844.1 | 0.670303613 | -0.860720553 | 0.295916495 | -2.908660276 | 0.003629811 | 0.024003262 | down |
| GPR22 | 1.738513416 | -0.553384512 | 0.190322887 | -2.907608855 | 0.003642036 | 0.024050812 | down |
| LINC01251 | 1.068841703 | 0.580705672 | 0.199730468 | 2.907446608 | 0.003643925 | 0.02405519 | up |
| AL513366.1 | 3.168807995 | -0.527710906 | 0.181536125 | -2.906919524 | 0.003650071 | 0.024074466 | down |
| ADCY8 | 1.521810011 | -1.091490975 | 0.37561587 | -2.905870229 | 0.003662333 | 0.024137185 | down |
| AC108734.3 | 2.005991996 | -0.646951171 | 0.22276905 | -2.904133999 | 0.003682705 | 0.024229284 | down |
| HSD3B2 | 13.87781226 | 0.866492569 | 0.29836629 | 2.904123543 | 0.003682828 | 0.024229284 | up |
| AC005224.3 | 1.82651954 | 0.56786869 | 0.195655274 | 2.902393985 | 0.003703225 | 0.024320412 | up |
| KRT18P40 | 1.136629783 | 0.681480323 | 0.234921698 | 2.900882835 | 0.00372113 | 0.024404452 | up |
| AC006120.1 | 1.566341179 | -0.761572478 | 0.262550737 | -2.900667841 | 0.003723684 | 0.024412332 | down |
| ORM2 | 11.91810342 | -0.57351965 | 0.197740502 | -2.900365095 | 0.003727282 | 0.02443043 | down |
| ALPP | 21.01620482 | 0.758758405 | 0.261664426 | 2.899738479 | 0.003734741 | 0.024464933 | up |
| AC124312.3 | 4.043967149 | -0.541062213 | 0.186613162 | -2.89937863 | 0.003739031 | 0.02448344 | down |
| AC253536.5 | 31.02793856 | -0.571019261 | 0.196979805 | -2.898872099 | 0.003745076 | 0.024513427 | down |
| SLC7A10 | 7.375762214 | -0.696929788 | 0.240475172 | -2.898136139 | 0.003753876 | 0.024556606 | down |
| DMRT1 | 3.124449764 | -0.950629839 | 0.328061412 | -2.897719159 | 0.00375887 | 0.024578531 | down |
| AC010533.1 | 1.715469169 | -0.573858904 | 0.19817763 | -2.895679511 | 0.003783386 | 0.024677239 | down |
| CA15P1 | 1.473324937 | 0.556407494 | 0.192223051 | 2.894592974 | 0.003796505 | 0.024733861 | up |
| AC011474.4 | 0.4019752 | -1.588392 | 0.5488495 | -2.894039 | 0.003803204 | NA | down |
| NCAPD2P1 | 15.62432282 | -0.564183659 | 0.19494853 | -2.8940134 | 0.003803519 | 0.024766856 | down |
| AL162578.1 | 1.64652353 | -0.761252591 | 0.263097834 | -2.893420217 | 0.003810711 | 0.024802254 | down |
| TCAM1P | 11.21243268 | 0.913576271 | 0.315905033 | 2.891933261 | 0.003828793 | 0.024895714 | up |
| SNORA2C | 2.19700049 | -0.506439047 | 0.175164462 | -2.891220299 | 0.00383749 | 0.024932875 | down |
| PRDM13 | 16.24429479 | -0.779307448 | 0.269558525 | -2.891051013 | 0.003839558 | 0.02493662 | down |
| POU6F2-AS1 | 4.971343477 | -1.20767 | 0.417916395 | -2.889740659 | 0.003855598 | 0.0249971 | down |
| IGLVI-20 | 0.532505453 | -1.116965397 | 0.38663212 | -2.888961727 | 0.003865161 | 0.025054246 | down |
| AP001636.1 | 1.492713786 | 0.607520817 | 0.210376138 | 2.887783867 | 0.003879664 | 0.025119038 | up |
| AC136475.9 | 5.417138594 | -0.568138666 | 0.196785817 | -2.887091535 | 0.003888211 | 0.025150031 | down |
| WFDC2 | 1616.858261 | -0.542052316 | 0.187762306 | -2.886907008 | 0.003890492 | 0.025159919 | down |
| AC013270.1 | 0.987969571 | -0.553970536 | 0.191999766 | -2.88526672 | 0.003910822 | 0.025271844 | down |
| AC009362.1 | 27.20253836 | -0.690935852 | 0.239500712 | -2.884901035 | 0.003915367 | 0.025296328 | down |
| DPP10-AS1 | 23.32114834 | -0.959157605 | 0.332599232 | -2.883823867 | 0.003928784 | 0.025364604 | down |
| LINC01749 | 3.655554346 | 0.75826248 | 0.262937823 | 2.883809071 | 0.003928969 | 0.025364604 | up |
| CP | 98.925542 | 0.523436009 | 0.181590834 | 2.882502367 | 0.003945302 | 0.025440085 | up |
| CLC | 59.05183703 | -0.713049401 | 0.247590552 | -2.879954006 | 0.003977332 | 0.025607616 | down |
| AC140479.3 | 1.039943733 | -0.755811581 | 0.262660134 | -2.877526827 | 0.004008058 | 0.025755857 | down |
| FER1L6 | 170.9059695 | 0.608379442 | 0.211586549 | 2.875321912 | 0.004036158 | 0.025906554 | up |
| TEX15 | 0.792233713 | 1.144235533 | 0.398299836 | 2.872799409 | 0.004068524 | 0.026069265 | up |
| AC093720.1 | 20.73921102 | -0.643767812 | 0.224144516 | -2.872110481 | 0.004077404 | 0.026111157 | down |
| AC009041.1 | 1.988169987 | 0.601285152 | 0.209420759 | 2.871182177 | 0.004089398 | 0.026172928 | up |
| LINC01687 | 2.069185436 | 0.945482358 | 0.329344255 | 2.870802645 | 0.004094311 | 0.026194345 | up |
| RPS7P15 | 3.266415146 | -0.652515794 | 0.227307095 | -2.870635397 | 0.004096477 | 0.026203193 | down |
| PKHD1L1 | 9.680340551 | 0.501265503 | 0.174778839 | 2.867998809 | 0.004130771 | 0.026363752 | up |
| AL589743.4 | 1.012697452 | -1.009202553 | 0.352033086 | -2.866783242 | 0.004146669 | 0.026443319 | down |
| MRPS36P2 | 0.948592097 | -1.179072388 | 0.411312489 | -2.866609743 | 0.004148943 | 0.026452774 | down |
| SERPINA3 | 3.982070437 | 0.666934541 | 0.232743899 | 2.865529632 | 0.004163123 | 0.02652801 | up |
| LINC01500 | 0.550945022 | -0.706579042 | 0.246590641 | -2.865392777 | 0.004164923 | 0.026533919 | down |
| AC104212.1 | 4.204177006 | -0.504720744 | 0.176278415 | -2.863202198 | 0.004193828 | 0.026677918 | down |
| AC090971.4 | 1.002727796 | -0.777377102 | 0.271614742 | -2.862057839 | 0.004209001 | 0.026734263 | down |
| IGBP1-AS2 | 1.89161429 | -0.574742292 | 0.20086416 | -2.861348147 | 0.004218435 | 0.026773334 | down |
| AC007638.2 | 3.280796785 | -0.70524226 | 0.246577188 | -2.860127758 | 0.004234704 | 0.026851105 | down |
| AC243919.1 | 2.570896848 | 0.509598648 | 0.178221571 | 2.859354476 | 0.004245041 | 0.026904522 | up |
| TDGF1P5 | 1.209975281 | -0.588061886 | 0.205695826 | -2.858890713 | 0.004251252 | 0.026930503 | down |
| AC114947.1 | 0.736801902 | 0.735209367 | 0.257211528 | 2.858384198 | 0.004258045 | 0.026958213 | up |
| AC096664.2 | 7.739061505 | -0.660974432 | 0.231476565 | -2.855470197 | 0.004297316 | 0.027155429 | down |
| AL049830.2 | 0.846468767 | -1.184764473 | 0.414947836 | -2.855213041 | 0.004300797 | 0.027164332 | down |
| CCDC42 | 1.089195934 | 0.716979415 | 0.251147433 | 2.854814827 | 0.004306193 | 0.027164332 | up |
| CER1 | 1.59837673 | 0.842952168 | 0.295283634 | 2.854720248 | 0.004307475 | 0.027164332 | up |
| AL451142.1 | 0.693854266 | 0.635283021 | 0.22263192 | 2.853512737 | 0.004323879 | 0.027240929 | up |
| AC020951.1 | 4.222431591 | -0.557045497 | 0.195270551 | -2.852685639 | 0.004335148 | 0.027278471 | down |
| PPP1R14BP2 | 12.27103378 | -0.567812359 | 0.199046887 | -2.852656308 | 0.004335548 | 0.027278471 | down |
| SLC6A14 | 611.801761 | 0.539084456 | 0.188998837 | 2.852316265 | 0.004340189 | 0.0272974 | up |
| RASGEF1A | 140.5560805 | 0.547499698 | 0.192049064 | 2.850832419 | 0.004360494 | 0.027399341 | up |
| SLC39A2 | 171.8068103 | -0.765729005 | 0.26861937 | -2.850609782 | 0.004363548 | 0.027402392 | down |
| AL354863.1 | 1.800738376 | -1.256219293 | 0.440692402 | -2.850558093 | 0.004364258 | 0.027402392 | down |
| AC012531.2 | 0.3195818 | 1.056749 | 0.3709833 | 2.848509 | 0.004392459 | NA | up |
| AL161670.2 | 1.348412751 | -0.63429696 | 0.222849194 | -2.846305825 | 0.004422969 | 0.027708603 | down |
| SEMA3D | 36.56881157 | 0.575914699 | 0.202387484 | 2.845604314 | 0.004432724 | 0.027758895 | up |
| RNA5SP226 | 0.760040617 | -1.780012803 | 0.625620965 | -2.84519366 | 0.004438443 | 0.027769857 | down |
| AP000781.2 | 1.887498911 | 0.565728684 | 0.198845919 | 2.845060577 | 0.004440298 | 0.027769857 | up |
| CPA1 | 1.150042818 | 0.9773079 | 0.343829358 | 2.84242133 | 0.004477228 | 0.027956724 | up |
| HSPE1P11 | 0.887285456 | -0.670930054 | 0.2362236 | -2.84023295 | 0.00450806 | 0.028120507 | down |
| AL031668.1 | 0.786096849 | -0.815935844 | 0.287480165 | -2.83823353 | 0.004536398 | 0.028244588 | down |
| IGHD3-9 | 1.349659938 | 0.80053568 | 0.282080786 | 2.837966003 | 0.004540202 | 0.02826301 | up |
| AL139811.2 | 0.802158388 | -0.951837805 | 0.335630272 | -2.835971258 | 0.004568656 | 0.028392569 | down |
| C1QBPP2 | 4.146283454 | -0.525248154 | 0.185275527 | -2.834957013 | 0.004583185 | 0.028466994 | down |
| AC025810.1 | 0.44233841 | -1.745610924 | 0.615806723 | -2.834673377 | 0.004587256 | 0.028486987 | down |
| C20orf203 | 2.104303962 | -0.500445048 | 0.17655189 | -2.834549365 | 0.004589037 | 0.028492755 | down |
| FUNDC2P2 | 1.867073916 | -0.705026444 | 0.24874455 | -2.834339263 | 0.004592055 | 0.028506204 | down |
| GLYATL1P4 | 3.555976686 | 0.593083698 | 0.209363452 | 2.832794799 | 0.0046143 | 0.028628348 | up |
| SLC6A10P | 0.61067418 | 0.915523159 | 0.323237006 | 2.832358744 | 0.004620598 | 0.028656789 | up |
| AC010335.1 | 0.90428246 | 0.510304814 | 0.180206832 | 2.831772854 | 0.004629072 | 0.028701744 | up |
| CDK5R2 | 12.16989982 | 0.546742058 | 0.193082193 | 2.831654493 | 0.004630786 | 0.028704004 | up |
| AIRN | 1.122773173 | -0.950065446 | 0.335566656 | -2.831227206 | 0.004636977 | 0.028721086 | down |
| RGS7 | 5.497055097 | 0.709906911 | 0.250753208 | 2.831098018 | 0.00463885 | 0.028727369 | up |
| SEC13P1 | 2.098747353 | -0.556775625 | 0.196693063 | -2.830682572 | 0.004644879 | 0.028754056 | down |
| AL158214.1 | 1.578898645 | -0.853480296 | 0.301586381 | -2.829969619 | 0.004655242 | 0.028807543 | down |
| RN7SKP243 | 0.618280643 | -0.97607338 | 0.345192807 | -2.82761796 | 0.004689573 | 0.02897147 | down |
| KCNT1 | 32.63737503 | -0.581998578 | 0.205900735 | -2.826597869 | 0.004704537 | 0.029015936 | down |
| SNRPD2P1 | 3.822631952 | -0.640409314 | 0.226648831 | -2.825557542 | 0.004719841 | 0.029088869 | down |
| SOX2-OT | 7.218630287 | 0.51841279 | 0.183548875 | 2.82438554 | 0.004737136 | 0.029168584 | up |
| IGHD | 87.51802755 | 0.653235113 | 0.23132268 | 2.823912954 | 0.004744127 | 0.029206249 | up |
| FSCN1P1 | 3.354065309 | 0.758317927 | 0.268626047 | 2.822950098 | 0.004758398 | 0.029277935 | up |
| AC124276.2 | 0.904220061 | -0.671766849 | 0.238036766 | -2.82211383 | 0.004770824 | 0.029332805 | down |
| OVCH2 | 0.789108554 | 0.918011 | 0.325312765 | 2.82193353 | 0.004773507 | 0.029343906 | up |
| AC244157.2 | 2.542134356 | 0.953326498 | 0.337847605 | 2.821764852 | 0.004776018 | 0.029353947 | up |
| FZD10 | 261.9384321 | -0.681056455 | 0.241387364 | -2.821425463 | 0.004781075 | 0.029375669 | down |
| COPS8P2 | 2.909382388 | -0.657291125 | 0.232965823 | -2.821405805 | 0.004781368 | 0.029375669 | down |
| IGKV3OR22-2 | 1.79420995 | -0.966166374 | 0.342723578 | -2.819083467 | 0.004816099 | 0.029540568 | down |
| GALNT13 | 8.482092192 | 0.635833664 | 0.225561441 | 2.818893422 | 0.004818952 | 0.029549037 | up |
| INSRR | 4.127254462 | 0.531294923 | 0.188516771 | 2.818289963 | 0.004828019 | 0.029583169 | up |
| AC091544.2 | 3.015144313 | 0.72856836 | 0.258521768 | 2.818208944 | 0.004829237 | 0.029583169 | up |
| YAP1P1 | 2.14141134 | 0.50128601 | 0.177893569 | 2.817898435 | 0.00483391 | 0.029595529 | up |
| UPK3A | 73.7809816 | -0.562738934 | 0.199707279 | -2.817818849 | 0.004835108 | 0.029597446 | down |
| ISPD-AS1 | 1.760655709 | -0.509461759 | 0.180856111 | -2.816945232 | 0.004848279 | 0.029650934 | down |
| LINC01397 | 0.445211442 | 0.782846959 | 0.277996522 | 2.816031484 | 0.00486209 | 0.029679434 | up |
| LRP2 | 5.872223682 | 0.669426727 | 0.237723314 | 2.815991059 | 0.004862702 | 0.029679434 | up |
| AL450322.1 | 1.318665953 | 0.602135075 | 0.213846126 | 2.815739926 | 0.004866504 | 0.029691803 | up |
| AC010320.4 | 1.430350795 | -0.619815903 | 0.220165376 | -2.815228776 | 0.004874252 | 0.029722806 | down |
| RN7SL273P | 0.4036421 | -1.108578 | 0.393811 | -2.814999 | 0.004877731 | NA | down |
| NRK | 27.67029858 | -0.566270173 | 0.201210446 | -2.81431797 | 0.004888085 | 0.029800872 | down |
| PHF21B | 1.612970934 | 0.708942354 | 0.251932733 | 2.814014461 | 0.004892703 | 0.029819003 | up |
| LINC00890 | 7.004875663 | 0.626544153 | 0.222673123 | 2.813739466 | 0.00489689 | 0.029833648 | up |
| DUXAP10 | 9.468693963 | -0.624244927 | 0.222236321 | -2.808923957 | 0.004970739 | 0.030179098 | down |
| NOL4 | 10.32462135 | 0.685348092 | 0.244023185 | 2.808536782 | 0.00497672 | 0.030204443 | up |
| LINC01825 | 1.048516375 | 0.651557087 | 0.231999964 | 2.80843615 | 0.004978275 | 0.030208402 | up |
| SLC6A13 | 2.593413997 | 0.554690666 | 0.19756154 | 2.80768548 | 0.004989894 | 0.030256941 | up |
| AC079466.1 | 15.39836094 | 0.866576123 | 0.308698613 | 2.807191497 | 0.004997552 | 0.030297887 | up |
| DRAIC | 57.08109581 | -0.51401869 | 0.183140221 | -2.806694718 | 0.005005265 | 0.030328782 | down |
| LINC00567 | 0.707503517 | 0.818196662 | 0.291521936 | 2.80663841 | 0.00500614 | 0.030328782 | up |
| C17orf64 | 0.974179452 | 0.634204298 | 0.226269855 | 2.802866954 | 0.005065056 | 0.030612784 | up |
| AC063926.1 | 0.719168832 | 0.793400192 | 0.283116336 | 2.80238224 | 0.005072673 | 0.030647744 | up |
| AP005018.2 | 25.34910036 | 0.754308416 | 0.26922133 | 2.801815207 | 0.005081597 | 0.030690571 | up |
| C9orf129 | 0.799011412 | -0.70191441 | 0.250634402 | -2.800550937 | 0.005101546 | 0.030777699 | down |
| AL645939.5 | 1.014851685 | 0.773676516 | 0.27636341 | 2.799489689 | 0.005118345 | 0.030854152 | up |
| LGALS17A | 18.57392289 | 0.568890002 | 0.203232116 | 2.799213099 | 0.005122732 | 0.030866536 | up |
| AC011998.1 | 0.732994867 | -0.941222974 | 0.336377596 | -2.798114337 | 0.005140191 | 0.03093829 | down |
| HIST2H2BA | 0.730067827 | -0.775756108 | 0.277304561 | -2.797487737 | 0.005150172 | 0.030976063 | down |
| AC090954.1 | 2.316209001 | 0.504240514 | 0.180258966 | 2.797311696 | 0.005152979 | 0.030987374 | up |
| L1TD1 | 1013.838781 | 0.678375891 | 0.24286903 | 2.793175773 | 0.005219332 | 0.031290782 | up |
| LINC01429 | 1.969153217 | -0.60432286 | 0.216356945 | -2.793175226 | 0.005219341 | 0.031290782 | down |
| UCN3 | 36.60466888 | 0.603435278 | 0.216044648 | 2.793104499 | 0.005220482 | 0.031292015 | up |
| SLC38A4 | 129.2624351 | -0.509301699 | 0.182462377 | -2.791269672 | 0.005250171 | 0.031424909 | down |
| NTSR2 | 0.616961576 | 0.95476284 | 0.342137734 | 2.790580359 | 0.005261364 | 0.03147709 | up |
| AC011753.1 | 1.166529689 | -1.136637718 | 0.407482382 | -2.789415615 | 0.005280325 | 0.03156585 | down |
| AC093904.3 | 2.092376588 | 0.623423858 | 0.223525831 | 2.789046144 | 0.005286353 | 0.031590589 | up |
| AC140076.1 | 9.72014121 | -0.784583041 | 0.281359997 | -2.788537995 | 0.005294654 | 0.031628888 | down |
| AC104581.2 | 0.852700013 | -0.638460356 | 0.228995261 | -2.788094194 | 0.005301913 | 0.031660939 | down |
| NXPE4 | 893.9418787 | -0.581113736 | 0.208478261 | -2.787406873 | 0.005313172 | 0.031705531 | down |
| AC099520.1 | 1.153442562 | 0.745271934 | 0.267471376 | 2.786361464 | 0.00533034 | 0.031762632 | up |
| GRK1 | 15.64600489 | -0.579773461 | 0.20820408 | -2.784640248 | 0.005358714 | 0.031897607 | down |
| AL133371.1 | 0.889956102 | -0.875909324 | 0.314560902 | -2.784546074 | 0.005360271 | 0.031900936 | down |
| AC009166.2 | 0.560805054 | -0.732344411 | 0.263080603 | -2.783726369 | 0.005373835 | 0.03195917 | down |
| AL050327.1 | 0.464454389 | -0.924538873 | 0.332156502 | -2.783443551 | 0.005378522 | 0.031981358 | down |
| MRPS21P9 | 0.3656891 | -0.9391777 | 0.3374554 | -2.783117 | 0.005383941 | NA | down |
| CLDN10-AS1 | 9.02724124 | -0.66342359 | 0.238388596 | -2.782950194 | 0.005386707 | 0.032022321 | down |
| AC112512.1 | 1.21241113 | -0.56570459 | 0.20333626 | -2.782113684 | 0.005400612 | 0.032078483 | down |
| TMEM14DP | 7.726376938 | -0.528830172 | 0.190174574 | -2.780761706 | 0.005423153 | 0.03218755 | down |
| GRIA1 | 4.178927202 | 0.562813397 | 0.202470947 | 2.779724225 | 0.005440508 | 0.032252442 | up |
| AC004386.1 | 18.72030263 | -0.62704444 | 0.225670942 | -2.778578558 | 0.005459731 | 0.032314843 | down |
| TRIM58 | 41.42943619 | 0.54894612 | 0.197664695 | 2.777158154 | 0.005483649 | 0.032433446 | up |
| AC027243.2 | 0.672092158 | 0.714970092 | 0.257508333 | 2.776493027 | 0.005494881 | 0.032488389 | up |
| BANF2 | 0.948075157 | -0.866228114 | 0.312059342 | -2.775844196 | 0.005505859 | 0.032530286 | down |
| HS3ST5 | 7.515877436 | -0.633106326 | 0.228084662 | -2.775751425 | 0.00550743 | 0.032533821 | down |
| AP000523.1 | 5.082046609 | -0.518556061 | 0.186826074 | -2.775608611 | 0.005509849 | 0.032538865 | down |
| ACTL6B | 1.135032667 | 0.708495438 | 0.255291641 | 2.775239464 | 0.005516108 | 0.032562075 | up |
| RPL29P2 | 8.475705424 | -0.585613293 | 0.21105302 | -2.774721224 | 0.005524904 | 0.032590991 | down |
| AC106772.2 | 3.846923945 | -0.721294603 | 0.259964404 | -2.774589876 | 0.005527136 | 0.032595311 | down |
| DAW1 | 1.84901549 | -0.586339004 | 0.211326584 | -2.774563387 | 0.005527586 | 0.032595311 | down |
| PRICKLE2-AS3 | 0.504393669 | -1.071460636 | 0.386210205 | -2.774293954 | 0.005532167 | 0.03261657 | down |
| OR7E85P | 2.283499998 | -0.673011884 | 0.242704254 | -2.772971105 | 0.005554705 | 0.032689255 | down |
| AC010326.1 | 1.106497453 | -0.707253752 | 0.255125611 | -2.772178578 | 0.005568248 | 0.032736952 | down |
| AC009078.1 | 1.632284504 | -1.305338481 | 0.471150426 | -2.770534441 | 0.005596438 | 0.03287379 | down |
| RNA5S9 | 4.676635059 | -1.49138358 | 0.53864755 | -2.768755896 | 0.005627078 | 0.033001597 | down |
| KHDC1L | 0.4148798 | 1.171848 | 0.4237803 | 2.765226 | 0.005688337 | NA | up |
| HYDIN2 | 3.064709946 | -0.533849414 | 0.193069959 | -2.765056855 | 0.005691288 | 0.033276704 | down |
| RN7SL836P | 0.611674537 | -1.069937145 | 0.387343256 | -2.762245448 | 0.005740531 | 0.033520958 | down |
| MRPL53P1 | 1.030744515 | -0.747159273 | 0.270630048 | -2.76081418 | 0.005765747 | 0.03362521 | down |
| ST6GAL2-IT1 | 0.3991202 | -0.9278716 | 0.3361172 | -2.760559 | 0.005770247 | NA | down |
| LINC02413 | 7.30125475 | -0.569808181 | 0.206452116 | -2.760001648 | 0.005780107 | 0.033667851 | down |
| AC108688.1 | 1.074800416 | -0.792534274 | 0.287197879 | -2.759540831 | 0.005788265 | 0.033692843 | down |
| RPL13P2 | 50.72989803 | -0.625330073 | 0.226622196 | -2.759350514 | 0.005791638 | 0.033706606 | down |
| SHANK2-AS2 | 0.4246948 | -0.7723391 | 0.2799798 | -2.758553 | 0.005805788 | NA | down |
| PPP1R1A | 22.70245336 | -0.545938963 | 0.198041495 | -2.756689761 | 0.005838972 | 0.033940738 | down |
| AC103988.1 | 0.685915759 | -0.733154357 | 0.265971666 | -2.756513004 | 0.005842129 | 0.033953186 | down |
| B4GALNT2 | 317.6865018 | 0.706565147 | 0.256338064 | 2.756380138 | 0.005844503 | 0.033961081 | up |
| PNPT1P1 | 2.012276724 | -0.641038241 | 0.232578425 | -2.756224022 | 0.005847293 | 0.033971393 | down |
| AC022405.1 | 0.623866915 | 0.519295023 | 0.188447396 | 2.755649774 | 0.005857568 | 0.034004808 | up |
| AC011374.1 | 0.788105222 | 0.679761113 | 0.24668414 | 2.7555931 | 0.005858583 | 0.034004808 | up |
| BANCR | 1.873798016 | 0.598610401 | 0.21733397 | 2.754334268 | 0.005881167 | 0.034108931 | up |
| SNORD3A | 0.618921139 | 0.645908914 | 0.23456397 | 2.753657832 | 0.005893335 | 0.034161727 | up |
| AL356481.2 | 0.762149864 | -0.627882009 | 0.228178579 | -2.751713202 | 0.005928442 | 0.034305765 | down |
| RN7SL200P | 0.694007247 | -1.044565984 | 0.379814717 | -2.750198815 | 0.005955912 | 0.034446842 | down |
| LINC02132 | 0.847185286 | 0.66878497 | 0.243349651 | 2.748247096 | 0.005991484 | 0.034616656 | up |
| AC117947.1 | 0.658166171 | -0.892006081 | 0.324616445 | -2.747877057 | 0.00599825 | 0.034643776 | down |
| AC004973.1 | 0.586599036 | -0.537244404 | 0.195533172 | -2.747587018 | 0.006003558 | 0.034652271 | down |
| LINC01198 | 1.097014679 | -0.89327269 | 0.325146198 | -2.747295507 | 0.006008897 | 0.034669344 | down |
| AC011287.1 | 1.466202234 | 0.963346226 | 0.35072125 | 2.746757509 | 0.006018762 | 0.034707596 | up |
| AICDA | 1.922203851 | 0.704694905 | 0.256565141 | 2.746651015 | 0.006020716 | 0.034707596 | up |
| KRT18P41 | 1.189710925 | 0.511504204 | 0.186242298 | 2.746444865 | 0.006024501 | 0.034717446 | up |
| SNORA74A | 1.492970099 | -0.624706331 | 0.227468958 | -2.746336628 | 0.006026489 | 0.03472292 | down |
| TBX4 | 15.5054049 | 0.613407354 | 0.22336675 | 2.746189186 | 0.006029198 | 0.034728273 | up |
| SNORD116-4 | 0.92934483 | -0.649592248 | 0.236618217 | -2.745317985 | 0.006045229 | 0.03478295 | down |
| MIR215 | 0.605478345 | -0.807857245 | 0.294312582 | -2.744895372 | 0.006053019 | 0.034815792 | down |
| C10orf62 | 0.469879555 | -0.765211054 | 0.278827229 | -2.744391415 | 0.006062321 | 0.034851309 | down |
| AC034238.1 | 1.562345103 | 0.582301988 | 0.21232527 | 2.74249969 | 0.006097351 | 0.034977019 | up |
| AC005410.1 | 0.769611428 | -0.546995297 | 0.199626142 | -2.740098527 | 0.006142077 | 0.035137182 | down |
| AP004290.1 | 0.860437123 | -0.65170831 | 0.237841639 | -2.740093426 | 0.006142172 | 0.035137182 | down |
| AL161658.1 | 1.16059826 | 1.015517414 | 0.370648608 | 2.739838735 | 0.006146934 | 0.035144973 | up |
| AC239584.1 | 8.429987329 | -0.613850009 | 0.224057733 | -2.739695704 | 0.006149609 | 0.03514768 | down |
| PLIN4 | 169.0411599 | -0.563425082 | 0.205693082 | -2.739154266 | 0.006159746 | 0.035175559 | down |
| SNHG25 | 225.4792057 | -0.546067664 | 0.199381984 | -2.738801432 | 0.00616636 | 0.035201307 | down |
| AP002754.1 | 6.252598798 | -0.579649895 | 0.211731023 | -2.737671061 | 0.006187593 | 0.035287621 | down |
| AC090164.2 | 4.391643872 | -0.64318677 | 0.234969453 | -2.737320797 | 0.006194186 | 0.035305908 | down |
| LINC02560 | 3.649404713 | -0.580309297 | 0.212191925 | -2.734832147 | 0.006241209 | 0.035513405 | down |
| VTRNA1-3 | 0.600322747 | -0.96835563 | 0.354446909 | -2.732018828 | 0.006294754 | 0.035762397 | down |
| AL049629.1 | 1.209374911 | 0.556722362 | 0.203827897 | 2.731335449 | 0.006307823 | 0.035782852 | up |
| AC022028.2 | 2.20742521 | -0.640453129 | 0.2345179 | -2.730934949 | 0.006315494 | 0.035820289 | down |
| AC034223.2 | 0.642914227 | -0.870957932 | 0.319052501 | -2.729826373 | 0.006336769 | 0.03592268 | down |
| VTRNA2-1 | 0.944120717 | -0.899742663 | 0.329681419 | -2.729127608 | 0.006350213 | 0.03599279 | down |
| PLD5 | 1.038501504 | -0.734918245 | 0.269317228 | -2.728820023 | 0.006356138 | 0.036020272 | down |
| AC007347.1 | 0.542933976 | -0.762212065 | 0.279493902 | -2.727115182 | 0.006389073 | 0.036170137 | down |
| PPY | 1.175735535 | -0.883703187 | 0.324068774 | -2.726900149 | 0.006393238 | 0.036181465 | down |
| AL391261.4 | 1.228264114 | 0.502931816 | 0.18447063 | 2.72635171 | 0.006403872 | 0.036211005 | up |
| FOXD3-AS1 | 3.332501819 | 0.636764642 | 0.233661778 | 2.725155335 | 0.006427124 | 0.036311785 | up |
| RNA5SP311 | 0.738910062 | 0.587028013 | 0.215472392 | 2.724376929 | 0.006442294 | 0.036375415 | up |
| BFSP2-AS1 | 0.627232249 | 0.680869618 | 0.249964125 | 2.723869344 | 0.006452203 | 0.03640427 | up |
| RNU1-2 | 0.519711391 | -1.353864053 | 0.49711292 | -2.723453765 | 0.006460326 | 0.03643639 | down |
| AL162426.1 | 1.05682561 | 0.536430625 | 0.19697014 | 2.723410896 | 0.006461164 | 0.03643639 | up |
| AC018797.3 | 1.283794698 | -0.661346625 | 0.242848086 | -2.723293538 | 0.00646346 | 0.036443191 | down |
| FAM25A | 1.186468325 | 1.029265539 | 0.378314721 | 2.720659501 | 0.006515183 | 0.036629832 | up |
| AL355112.1 | 2.25939919 | -0.561368219 | 0.206395447 | -2.71986726 | 0.006530813 | 0.036699506 | down |
| AC068580.2 | 4.826758571 | -0.718856734 | 0.264404783 | -2.718773566 | 0.006552445 | 0.036808383 | down |
| AL353803.2 | 0.763735295 | -0.748675652 | 0.275422873 | -2.71827697 | 0.006562288 | 0.036851297 | down |
| AC018735.1 | 0.860724382 | -0.819032426 | 0.301432839 | -2.717130713 | 0.00658506 | 0.036929561 | down |
| AC079298.3 | 1.839731295 | -0.675401408 | 0.248667444 | -2.716082958 | 0.006605936 | 0.037021806 | down |
| RPL37AP8 | 42.90529366 | -0.723420263 | 0.266472402 | -2.714803697 | 0.006631507 | 0.037140213 | down |
| AC110994.1 | 1.09921397 | -0.903827253 | 0.333162595 | -2.71287133 | 0.0066703 | 0.037319978 | down |
| FOXCUT | 0.665982043 | 0.65715267 | 0.242302711 | 2.712114399 | 0.006685552 | 0.037380292 | up |
| MAGEB2 | 4.788757243 | -1.64261425 | 0.606044832 | -2.710384056 | 0.006720534 | 0.037525694 | down |
| AL023881.1 | 0.594972268 | 0.531208903 | 0.195998164 | 2.710274889 | 0.006722747 | 0.037531781 | up |
| GPR27 | 18.78711154 | 0.501315471 | 0.185067991 | 2.708817811 | 0.006752341 | 0.037652998 | up |
| HTR6 | 1.997785026 | 0.510639546 | 0.188646423 | 2.706860472 | 0.00679228 | 0.03783155 | up |
| AL161804.1 | 0.540636926 | 0.61483144 | 0.227366286 | 2.704145149 | 0.006848037 | 0.038091351 | up |
| SCN7A | 28.72689715 | -0.616897238 | 0.228199919 | -2.703319264 | 0.006865077 | 0.038149406 | down |
| SNORA40B | 0.3965206 | -1.494736 | 0.5530561 | -2.702684 | 0.006878219 | NA | down |
| KARSP1 | 1.039180824 | -0.919241432 | 0.340214308 | -2.701948188 | 0.00689345 | 0.038267575 | down |
| ATP5G2P1 | 4.413041443 | -0.622613243 | 0.230500325 | -2.701138243 | 0.006910261 | 0.038329082 | down |
| PRSS45 | 3.907060505 | -0.769174487 | 0.284801653 | -2.700737437 | 0.006918593 | 0.038362573 | down |
| AC016542.3 | 0.554504661 | -1.292047771 | 0.478442841 | -2.700526921 | 0.006922973 | 0.038374134 | down |
| AL353572.1 | 1.265334565 | 0.567619123 | 0.210266544 | 2.69952182 | 0.00694392 | 0.038452001 | up |
| ENPP7P5 | 0.515674813 | -0.69700785 | 0.258216219 | -2.699318626 | 0.006948162 | 0.038452216 | down |
| IRX2 | 202.1358242 | 0.682157133 | 0.252882708 | 2.697523835 | 0.006985728 | 0.038613181 | up |
| SLC5A7 | 2.727380915 | -0.739199592 | 0.2740685 | -2.697134446 | 0.006993903 | 0.038639204 | down |
| AL136964.1 | 1.43950694 | 0.805233256 | 0.298608158 | 2.696621757 | 0.007004678 | 0.038681167 | up |
| ARHGAP40 | 9.120558925 | 0.647818733 | 0.240237564 | 2.696575519 | 0.007005651 | 0.038681167 | up |
| ERICH3 | 1.340588886 | 0.648936676 | 0.240742684 | 2.69556136 | 0.007027013 | 0.038768018 | up |
| MYL6P2 | 6.822755393 | -0.748405651 | 0.277644276 | -2.69555584 | 0.00702713 | 0.038768018 | down |
| SLC8A1-AS1 | 2.448135892 | -0.505045049 | 0.187375632 | -2.695361424 | 0.007031232 | 0.038768577 | down |
| AL451164.1 | 0.3904669 | -1.009393 | 0.3745871 | -2.69468 | 0.007045622 | NA | down |
| AL360091.3 | 0.550036857 | -0.903747073 | 0.335618367 | -2.69278193 | 0.00708586 | 0.039012218 | down |
| AC078852.2 | 1.637383991 | 0.509930828 | 0.189475195 | 2.691280125 | 0.00711784 | 0.039149291 | up |
| AC079313.2 | 1.370871007 | -0.687900634 | 0.255650561 | -2.690784763 | 0.007128417 | 0.039191527 | down |
| SAMM50P1 | 0.600333048 | -0.91963239 | 0.341856939 | -2.690108886 | 0.007142871 | 0.039254683 | down |
| AP002840.1 | 1.238671363 | -0.815734119 | 0.303301462 | -2.689515948 | 0.007155573 | 0.03930511 | down |
| RNU6-181P | 0.583696255 | 0.569142102 | 0.211696823 | 2.688477297 | 0.007177872 | 0.039382314 | up |
| AL355076.3 | 0.84643992 | 0.53390017 | 0.198660998 | 2.687493646 | 0.007199047 | 0.039472592 | up |
| HRH3 | 1.044016032 | 0.878861165 | 0.327051323 | 2.68722706 | 0.007204796 | 0.039497635 | up |
| AC073592.8 | 3.097632596 | -0.528331491 | 0.196658008 | -2.686549588 | 0.007219423 | 0.03954541 | down |
| AC108206.1 | 1.214958688 | 0.685861606 | 0.255386 | 2.685588118 | 0.007240228 | 0.039639893 | up |
| SNORD67 | 1.289181418 | -0.533876394 | 0.198881315 | -2.684396934 | 0.007266078 | 0.039748883 | down |
| SOD1P3 | 1.513048666 | -0.515853871 | 0.192202791 | -2.683904159 | 0.007276796 | 0.039801005 | down |
| HMGA1P5 | 12.48441588 | -0.605494701 | 0.225730879 | -2.682374272 | 0.007310162 | 0.03993164 | down |
| MKRN7P | 1.015662076 | -1.175141062 | 0.438106534 | -2.682318048 | 0.007311391 | 0.03993164 | down |
| AC026464.5 | 1.853971278 | -0.520785839 | 0.194200055 | -2.681697692 | 0.007324962 | 0.039992499 | down |
| RN7SL709P | 0.452233609 | -1.070009304 | 0.399057013 | -2.681344442 | 0.0073327 | 0.040021677 | down |
| UBL5P2 | 13.36443518 | -0.561981825 | 0.209596307 | -2.681258235 | 0.007334589 | 0.040025456 | down |
| ARHGAP36 | 1.34773924 | -0.75549274 | 0.281972877 | -2.679309969 | 0.007377407 | 0.040193524 | down |
| AC092104.1 | 0.490874928 | -0.89610571 | 0.334468311 | -2.67919465 | 0.007379948 | 0.040194273 | down |
| HNRNPABP1 | 5.793469007 | -0.515195387 | 0.192342625 | -2.678529459 | 0.007394623 | 0.040241429 | down |
| AL121974.1 | 0.4061429 | 1.849333 | 0.6906403 | 2.677708 | 0.007412775 | NA | up |
| PHBP8 | 5.214297079 | -0.514869124 | 0.192385422 | -2.676237729 | 0.007445381 | 0.040464976 | down |
| PICSAR | 3.724331551 | -0.668453845 | 0.249802669 | -2.675927553 | 0.007452275 | 0.040489283 | down |
| ESRRAP1 | 2.396631371 | -0.523317927 | 0.195653509 | -2.674717811 | 0.007479217 | 0.040582916 | down |
| AC061961.1 | 4.625169852 | 0.662877462 | 0.247922835 | 2.673724919 | 0.007501395 | 0.040683452 | up |
| HCG4P8 | 1.059293961 | -0.734342652 | 0.274679086 | -2.673456731 | 0.007507396 | 0.040702941 | down |
| FAM106A | 1.548125141 | 0.643142634 | 0.240566699 | 2.673448304 | 0.007507585 | 0.040702941 | up |
| AL645949.1 | 1.277344209 | 0.537972614 | 0.201261039 | 2.673009227 | 0.007517418 | 0.040727584 | up |
| ZNF812P | 1.961090082 | 0.548252422 | 0.20510901 | 2.672980681 | 0.007518058 | 0.040727584 | up |
| ART3 | 88.54677739 | 0.540247995 | 0.202143973 | 2.67259017 | 0.007526814 | 0.040761813 | up |
| HIST1H3I | 2.730874612 | -0.605301615 | 0.226491585 | -2.672512595 | 0.007528555 | 0.040764638 | down |
| UGT2B17 | 1168.652279 | 0.686562583 | 0.256962324 | 2.671841431 | 0.007543629 | 0.040826426 | up |
| BSND | 0.746149583 | 0.682083148 | 0.255392802 | 2.670721897 | 0.007568833 | 0.040942953 | up |
| TECRP2 | 6.775789085 | -0.767497733 | 0.287413694 | -2.670358962 | 0.00757702 | 0.040967358 | down |
| AC008555.4 | 0.565541332 | 0.605640172 | 0.226850758 | 2.66977363 | 0.00759024 | 0.041005689 | up |
| AL096855.2 | 0.824135799 | -0.763424652 | 0.285979121 | -2.669511851 | 0.007596159 | 0.041017788 | down |
| ZSCAN4 | 2.607607745 | 0.702210146 | 0.263254134 | 2.667423058 | 0.007643539 | 0.041207044 | up |
| AC008021.1 | 97.62234463 | -0.522311314 | 0.1958212 | -2.667286864 | 0.007646637 | 0.041210507 | down |
| ZPLD1 | 1.511668552 | 0.677676766 | 0.254097974 | 2.666990045 | 0.007653394 | 0.041233631 | up |
| AC099542.1 | 0.886728046 | 0.894170738 | 0.335291387 | 2.666846723 | 0.007656658 | 0.041244573 | up |
| NKAIN3 | 2.260980829 | -0.611226891 | 0.229376677 | -2.664729905 | 0.007705018 | 0.041411685 | down |
| SLC25A6P2 | 32.64485113 | -0.618781327 | 0.232253993 | -2.664244084 | 0.007716155 | 0.041448449 | down |
| FCRL4 | 4.056281961 | 0.537631498 | 0.201864633 | 2.663326849 | 0.007737222 | 0.041517119 | up |
| HPN-AS1 | 1.71340475 | 0.762137189 | 0.286161166 | 2.663314522 | 0.007737505 | 0.041517119 | up |
| CHGB | 66.86620465 | 0.55095286 | 0.206911318 | 2.662748778 | 0.007750525 | 0.041562752 | up |
| IGHV3-54 | 1.117476054 | -0.790959508 | 0.297204443 | -2.661331374 | 0.007783232 | 0.041704702 | down |
| AC138749.1 | 0.3483733 | -1.208387 | 0.4541974 | -2.660489 | 0.007802723 | NA | down |
| AC004801.1 | 2.541201797 | -0.572396627 | 0.215233813 | -2.659417773 | 0.007827583 | 0.041888657 | down |
| PNKDP1 | 2.530004556 | -0.741117826 | 0.278962193 | -2.656696301 | 0.007891049 | 0.042160822 | down |
| AC112247.1 | 4.209371169 | -0.698575721 | 0.263055534 | -2.655620703 | 0.00791626 | 0.042232288 | down |
| RPL5P17 | 18.49899847 | -0.511449894 | 0.192744046 | -2.653518508 | 0.00796574 | 0.04242826 | down |
| HBQ1 | 7.437342785 | 0.68750745 | 0.259127781 | 2.653159948 | 0.007974207 | 0.042450235 | up |
| CACNA1C-AS4 | 0.453319114 | -1.345976678 | 0.507422575 | -2.652575474 | 0.007988027 | 0.042495641 | down |
| REG4 | 21429.36511 | 0.664015435 | 0.250382955 | 2.651999355 | 0.008001669 | 0.042547914 | up |
| SNORD3B-1 | 1.236461516 | -0.686449432 | 0.259021722 | -2.650161644 | 0.008045327 | 0.042725707 | down |
| AC012671.1 | 2.487223425 | -0.823809988 | 0.310906649 | -2.649702062 | 0.008056278 | 0.042757489 | down |
| UBA52P3 | 10.10277876 | -0.617636083 | 0.233279081 | -2.647627384 | 0.008105881 | 0.042974482 | down |
| AC141930.2 | 1.109304835 | 0.734493707 | 0.277555764 | 2.646292396 | 0.008137944 | 0.043094376 | up |
| CYMP | 0.50343887 | 1.090433868 | 0.412093714 | 2.646082263 | 0.008143001 | 0.043107504 | up |
| UTS2R | 6.324835418 | -0.692610181 | 0.261886675 | -2.644694237 | 0.008176476 | 0.043216303 | down |
| AC109309.1 | 0.586336104 | 0.817954231 | 0.309313387 | 2.644419105 | 0.008183126 | 0.043224124 | up |
| AC008764.10 | 0.760250273 | -0.980491882 | 0.370811023 | -2.644182134 | 0.008188858 | 0.043240739 | down |
| AL132777.1 | 2.46632013 | -0.517988657 | 0.195906531 | -2.644060178 | 0.008191809 | 0.043249493 | down |
| AC007991.2 | 1.627000847 | 0.685359176 | 0.259237469 | 2.643750455 | 0.008199308 | 0.043282251 | up |
| MIR6763 | 0.712221842 | 0.526768319 | 0.199329019 | 2.642707624 | 0.008224602 | 0.043367853 | up |
| SELENOWP1 | 3.84197366 | -0.759174974 | 0.287330776 | -2.642163792 | 0.008237821 | 0.043410659 | down |
| NOTUM | 2168.813989 | -0.627046963 | 0.237465646 | -2.640579693 | 0.008276433 | 0.043557462 | down |
| AC092135.2 | 0.551662066 | -0.966223356 | 0.366002849 | -2.639933971 | 0.008292218 | 0.043628078 | down |
| LONRF2 | 18.773311 | -0.560760211 | 0.212544661 | -2.638317082 | 0.008331864 | 0.043759112 | down |
| AL121949.2 | 0.855036322 | -0.960758129 | 0.364225604 | -2.637810514 | 0.008344319 | 0.043778221 | down |
| GAPDHP66 | 0.966802889 | 0.756922805 | 0.287375603 | 2.633914625 | 0.008440671 | 0.04419099 | up |
| AL591806.1 | 1.363854087 | -0.542060446 | 0.205802901 | -2.633881461 | 0.008441495 | 0.04419099 | down |
| RN7SL388P | 0.559239348 | -0.669328342 | 0.254144644 | -2.633651181 | 0.008447222 | 0.044197089 | down |
| AC008063.1 | 0.947164314 | 0.601534189 | 0.228590938 | 2.631487471 | 0.0085012 | 0.044406222 | up |
| AC020914.1 | 24.37080213 | 0.727682077 | 0.276562065 | 2.631170972 | 0.008509122 | 0.044432011 | up |
| RPL21P65 | 3.092486314 | -0.512384305 | 0.194852888 | -2.629595643 | 0.008548648 | 0.044581349 | down |
| AC097372.1 | 0.825578627 | 0.570403507 | 0.216949667 | 2.629197429 | 0.008558666 | 0.044615797 | up |
| CCT7P2 | 0.431945363 | 0.994199429 | 0.37817158 | 2.628963893 | 0.008564545 | 0.044625578 | up |
| HOXC11 | 57.85903139 | 0.812773589 | 0.309300698 | 2.627778061 | 0.008594457 | 0.044746572 | up |
| NDUFB10P1 | 0.937609257 | -0.749310641 | 0.2853044 | -2.626355008 | 0.008630475 | 0.044878203 | down |
| DNAJA1P5 | 1.53916249 | 1.069613615 | 0.407284838 | 2.626205334 | 0.008634271 | 0.044883984 | up |
| HIF1A-AS1 | 0.840102335 | 0.507420249 | 0.193302815 | 2.625001862 | 0.008664849 | 0.045007957 | up |
| AC107983.2 | 8.908537875 | 0.605283405 | 0.230783972 | 2.622727216 | 0.008722908 | 0.045246281 | up |
| MGAT4C | 1.947101253 | 0.704377292 | 0.268635803 | 2.622052925 | 0.008740186 | 0.045310443 | up |
| RC3H1-IT1 | 0.626382255 | -1.057834596 | 0.403699587 | -2.620350948 | 0.008783932 | 0.045450018 | down |
| LSM6P2 | 0.80128544 | -1.04198759 | 0.397892931 | -2.618763763 | 0.008824904 | 0.045619664 | down |
| AP000295.1 | 0.899847792 | 0.50721232 | 0.193734945 | 2.618073476 | 0.008842776 | 0.045669696 | up |
| AL357992.1 | 0.660530811 | 0.560189229 | 0.214097711 | 2.616511997 | 0.008883324 | 0.04581543 | up |
| AC005005.1 | 0.947053459 | -0.821375324 | 0.314155329 | -2.614551624 | 0.008934466 | 0.045986989 | down |
| AC138932.2 | 1.0757149 | 0.50612955 | 0.193600796 | 2.614294771 | 0.008941186 | 0.046002302 | up |
| AL035409.1 | 1.385911239 | -0.528389771 | 0.202116733 | -2.614280187 | 0.008941568 | 0.046002302 | down |
| AC022296.1 | 0.627449273 | 0.596726716 | 0.228291315 | 2.613882691 | 0.008951977 | 0.04602753 | up |
| ZNF444P1 | 0.535947937 | -1.009407561 | 0.386196462 | -2.613715197 | 0.008956366 | 0.046043019 | down |
| AC020633.1 | 1.147754942 | 0.508287226 | 0.194526614 | 2.612944396 | 0.008976591 | 0.046125717 | up |
| SERPINA9 | 2.145489451 | 0.794487925 | 0.304192295 | 2.611795034 | 0.009006824 | 0.046238438 | up |
| WDR45P1 | 5.014793907 | -0.556603734 | 0.213209251 | -2.610598417 | 0.009038396 | 0.04638628 | down |
| AC134878.2 | 2.736084497 | 0.624070408 | 0.239079744 | 2.610302309 | 0.009046224 | 0.046419331 | up |
| HCN4 | 2.603191275 | 0.511518665 | 0.196022765 | 2.609486024 | 0.009067835 | 0.046508814 | up |
| RN7SL220P | 0.518933738 | -1.034668418 | 0.397030192 | -2.606019487 | 0.009160125 | 0.046884294 | down |
| TRBV21-1 | 0.784386273 | 0.650643534 | 0.249811473 | 2.60453824 | 0.009199815 | 0.047055832 | up |
| AL359265.2 | 1.582061892 | -0.701427237 | 0.269331558 | -2.604326213 | 0.009205509 | 0.04706336 | down |
| AC021549.1 | 2.15476132 | 0.537611862 | 0.206446887 | 2.604117067 | 0.009211129 | 0.047084892 | up |
| VAX1 | 5.126311479 | -1.21077946 | 0.465241832 | -2.602473329 | 0.009255401 | 0.047249545 | down |
| AC016168.2 | 0.659137105 | 0.9787077 | 0.376201005 | 2.601555249 | 0.009280212 | 0.047351168 | up |
| CKBP1 | 0.51796651 | -0.741591638 | 0.285198434 | -2.600265464 | 0.009315167 | 0.047478814 | down |
| BNIP3P27 | 1.202142115 | -0.606424047 | 0.233234056 | -2.600066457 | 0.009320571 | 0.047495576 | down |
| NR2E1 | 0.714005508 | 0.939349776 | 0.361304794 | 2.599881849 | 0.009325586 | 0.04750296 | up |
| SMIM28 | 1.192343458 | -0.865092013 | 0.332794607 | -2.59947726 | 0.009336586 | 0.04753593 | down |
| CROCC2 | 7.869549659 | 0.595532253 | 0.229145691 | 2.598924074 | 0.009351645 | 0.047577724 | up |
| SP7 | 1.525120742 | -0.714850688 | 0.275105483 | -2.598460339 | 0.009364286 | 0.0476203 | down |
| RPS24P16 | 6.961285307 | -0.738675646 | 0.284387018 | -2.597430964 | 0.0093924 | 0.047728657 | down |
| TAS2R30 | 0.905256961 | -0.802410175 | 0.309245082 | -2.594738677 | 0.009466286 | 0.048029446 | down |
| AC126773.3 | 1.413891753 | -0.634060152 | 0.244414166 | -2.594203771 | 0.009481028 | 0.048075064 | down |
| KLRC4 | 1.54869204 | 0.534784428 | 0.206224661 | 2.593212783 | 0.009508392 | 0.048177295 | up |
| AL079342.1 | 0.976159379 | -0.609083477 | 0.23491063 | -2.592830629 | 0.009518964 | 0.048209926 | down |
| OR1H1P | 1.196881439 | -0.75046213 | 0.289438171 | -2.59282363 | 0.009519157 | 0.048209926 | down |
| AC078991.1 | 29.33414527 | -0.687644999 | 0.265498725 | -2.590012438 | 0.009597246 | 0.048568632 | down |
| MAGEB17 | 85.12257006 | -0.734630732 | 0.28365954 | -2.589832629 | 0.009602261 | 0.048579303 | down |
| RNU11 | 0.759086515 | -0.696971331 | 0.269164057 | -2.589392277 | 0.00961455 | 0.048625436 | down |
| LGALS7 | 0.890762641 | 0.938077182 | 0.362480812 | 2.587936107 | 0.009655289 | 0.048759063 | up |
| FAM32BP | 1.404143471 | -0.690849457 | 0.266973962 | -2.587703503 | 0.009661811 | 0.048777265 | down |
| HLA-T | 2.338661214 | 0.502521056 | 0.194205108 | 2.587578982 | 0.009665303 | 0.048787534 | up |
| TRPM1 | 1.609586106 | 0.798301665 | 0.308631276 | 2.586587062 | 0.009693168 | 0.048884713 | up |
| LINC00470 | 3.657192481 | 0.958888915 | 0.37085292 | 2.585631293 | 0.009720085 | 0.04899749 | up |
| RANBP20P | 1.360993532 | -0.571543692 | 0.221075828 | -2.585283509 | 0.009729895 | 0.049027583 | down |
| AC069528.1 | 1.464841562 | 0.517552073 | 0.200192591 | 2.585270862 | 0.009730252 | 0.049027583 | up |
| RPL5P28 | 2.554284185 | -0.550869247 | 0.213137788 | -2.584568661 | 0.009750089 | 0.049102115 | down |
| RBMX2P3 | 0.568318947 | -0.65648355 | 0.254002732 | -2.58455311 | 0.009750529 | 0.049102115 | down |
| G6PC2 | 0.96110441 | 0.626999077 | 0.242717656 | 2.583244607 | 0.00978759 | 0.049205875 | up |
| AC007458.1 | 0.497299148 | -1.045354687 | 0.404686784 | -2.583120402 | 0.009791115 | 0.049207194 | down |
| SRGAP3-AS2 | 1.963358525 | -0.724825019 | 0.280677727 | -2.582410174 | 0.00981129 | 0.049279447 | down |
| CNTN2 | 7.440926104 | -0.523549106 | 0.202795961 | -2.581654501 | 0.009832797 | 0.049372156 | down |
| TTTY14 | 14.03935097 | -0.80019308 | 0.310089597 | -2.580522168 | 0.009865102 | 0.049476568 | down |
| AC119424.1 | 1.089648549 | -0.501897513 | 0.19460645 | -2.579038435 | 0.009907576 | 0.04965072 | down |
| GJB6 | 8.163757139 | 0.59115346 | 0.229299414 | 2.57808535 | 0.009934946 | 0.049743152 | up |
| RN7SL359P | 0.608590264 | -1.460704369 | 0.566858288 | -2.576842218 | 0.009970745 | 0.04987759 | down |
| RPL6P7 | 1.324885802 | -0.690936433 | 0.268187063 | -2.576322756 | 0.009985739 | 0.049937653 | down |
| LINC01300 | 0.813439795 | 0.747920786 | 0.290331408 | 2.576093272 | 0.009992369 | 0.049963338 | up |
| AC003986.2 | 1.101720436 | -0.608576059 | 0.236355176 | -2.574837031 | 0.010028733 | 0.050100217 | down |
| FMN2 | 15.70320948 | -0.580567755 | 0.225506967 | -2.574500307 | 0.0100385 | 0.050134031 | down |
| AL357552.1 | 0.658855893 | -0.871337 | 0.338828658 | -2.571615414 | 0.010122527 | 0.050454371 | down |
| ODF3 | 0.4104159 | -1.063357 | 0.4135165 | -2.571499 | 0.01012593 | NA | down |
| AL032821.1 | 0.4194624 | 0.8421147 | 0.3275215 | 2.571174 | 0.01013545 | NA | up |
| AC008011.2 | 0.573938868 | 0.726442596 | 0.282607175 | 2.570503017 | 0.010155094 | 0.050565293 | up |
| CU633906.1 | 0.870394213 | 0.544008511 | 0.211656694 | 2.570240051 | 0.010162807 | 0.05058863 | up |
| AL606537.1 | 2.019269135 | -0.508614535 | 0.198259784 | -2.565394376 | 0.010305859 | 0.051125685 | down |
| AP005060.1 | 0.557030604 | -0.981590242 | 0.383017684 | -2.562780471 | 0.010383768 | 0.051443495 | down |
| AC105399.1 | 18.22616158 | -0.643957847 | 0.251328076 | -2.562220098 | 0.010400538 | 0.05150503 | down |
| SIDT1-AS1 | 1.095519837 | 0.565885337 | 0.220878909 | 2.561970903 | 0.010408004 | 0.051533025 | up |
| CACNG6 | 1.462792556 | 0.737463651 | 0.287930558 | 2.561255245 | 0.01042947 | 0.05162018 | up |
| AL590560.3 | 0.60083281 | 0.556255547 | 0.217234118 | 2.560626998 | 0.010448347 | 0.051671571 | up |
| OR7E15P | 0.3239703 | -1.364417 | 0.5330352 | -2.559712 | 0.0104759 | NA | down |
| AC048380.2 | 5.332229786 | -0.522309918 | 0.204089913 | -2.559214766 | 0.010490891 | 0.051828317 | down |
| AC068756.1 | 1.430790629 | -1.243611898 | 0.48601489 | -2.558793823 | 0.010503602 | 0.051868125 | down |
| AC079776.2 | 0.784157754 | -0.568247769 | 0.222146537 | -2.557986162 | 0.010528028 | 0.051960228 | down |
| LINC02365 | 2.131410177 | 0.699844719 | 0.273620285 | 2.557722355 | 0.010536018 | 0.051982143 | up |
| IFNK | 0.3971993 | -1.391916 | 0.5442891 | -2.557309 | 0.01054854 | NA | down |
| AC104852.1 | 1.992650634 | -0.59743537 | 0.233678529 | -2.556654964 | 0.010568399 | 0.052118835 | down |
| ESRG | 2.708034575 | -1.018067338 | 0.398638747 | -2.553859466 | 0.010653625 | 0.052492686 | down |
| AC073910.1 | 0.446170514 | 0.824073521 | 0.322734958 | 2.553406441 | 0.010667493 | 0.052553277 | up |
| AHCYP2 | 3.34050853 | -0.54552993 | 0.213713748 | -2.552619729 | 0.010691616 | 0.052617854 | down |
| AL928654.4 | 0.688129262 | 0.520554149 | 0.204021697 | 2.551464651 | 0.010727121 | 0.052715009 | up |
| AC246817.1 | 2.68906286 | 0.651767527 | 0.25562498 | 2.549702016 | 0.010781503 | 0.052912274 | up |
| XIAPP2 | 0.654417683 | -0.832887545 | 0.326925161 | -2.547639775 | 0.01084544 | 0.053132485 | down |
| AC055716.2 | 3.747859528 | -0.560461541 | 0.220016425 | -2.547362278 | 0.010854069 | 0.053154973 | down |
| TRBV27 | 1.332841838 | 0.523936014 | 0.205688451 | 2.547231068 | 0.010858151 | 0.053163606 | up |
| CSAG3 | 2.717506745 | 1.433461872 | 0.562968006 | 2.546258147 | 0.010888463 | 0.053280817 | up |
| BAATP1 | 0.809283822 | 1.012292332 | 0.397583464 | 2.546112765 | 0.010892999 | 0.053287418 | up |
| RNF182 | 54.53345163 | -0.595590967 | 0.234024095 | -2.544998482 | 0.010927821 | 0.053387476 | down |
| RBMXP1 | 1.353476431 | -0.551070726 | 0.216636002 | -2.543763365 | 0.010966535 | 0.05354532 | down |
| AC243773.2 | 1.092794317 | 1.077676011 | 0.423736505 | 2.54326922 | 0.010982058 | 0.053597634 | up |
| AC092535.2 | 1.55723496 | -0.576606905 | 0.226899791 | -2.541240352 | 0.011045996 | 0.053834644 | down |
| HTR3C | 2.822362164 | 0.843973059 | 0.332149317 | 2.540944737 | 0.011055339 | 0.053868803 | up |
| PYDC1 | 3.208601484 | 0.846206266 | 0.333121788 | 2.540230919 | 0.01107793 | 0.053950113 | up |
| ZNF849P | 0.379304 | -1.099534 | 0.4329643 | -2.539548 | 0.01109958 | NA | down |
| ATP4B | 1.37873643 | 0.57554549 | 0.227124 | 2.534058439 | 0.011274993 | 0.054731751 | up |
| SFTA3 | 1.164633276 | -2.419018168 | 0.954630641 | -2.533983369 | 0.011277408 | 0.054735532 | down |
| AC095059.2 | 10.10262641 | -0.680888618 | 0.268870604 | -2.532402603 | 0.011328384 | 0.054895302 | down |
| RPL7P39 | 0.684001329 | -0.531064463 | 0.209722781 | -2.53222115 | 0.011334248 | 0.054902954 | down |
| AC111149.2 | 2.052890303 | 0.788665012 | 0.311463667 | 2.532125238 | 0.011337349 | 0.054902954 | up |
| SNORA22C | 1.007953758 | -0.951684344 | 0.375852299 | -2.532070038 | 0.011339134 | 0.054902954 | down |
| OTOP3 | 1.110645592 | 1.034428767 | 0.408655881 | 2.531295438 | 0.011364208 | 0.054981263 | up |
| AC009060.1 | 0.596687851 | 0.636086797 | 0.25130138 | 2.531171127 | 0.011368237 | 0.054984844 | up |
| RPS6P4 | 0.639993563 | -1.255407581 | 0.496019271 | -2.530965334 | 0.011374909 | 0.055009159 | down |
| EPPIN | 1.027683724 | -0.802791894 | 0.317302057 | -2.53005575 | 0.01140444 | 0.055112123 | down |
| APOOP5 | 0.81068141 | -0.752714216 | 0.297723208 | -2.528234939 | 0.011463761 | 0.055336897 | down |
| HPR | 2.157698374 | 0.632529949 | 0.250300833 | 2.527078882 | 0.011501567 | 0.055477267 | up |
| AC016924.1 | 0.746468547 | 0.66119779 | 0.261773817 | 2.525836231 | 0.011542327 | 0.055577677 | up |
| AL354943.1 | 0.625659944 | -0.941001819 | 0.372887097 | -2.523556932 | 0.011617425 | 0.055866883 | down |
| DBX1 | 2.608550716 | -0.733545636 | 0.290768426 | -2.522782981 | 0.011643023 | 0.055973883 | down |
| EEF1DP5 | 8.796457342 | -0.773122907 | 0.306576359 | -2.521795582 | 0.011675754 | 0.056090918 | down |
| AC020661.5 | 0.538323751 | -0.808975629 | 0.320940353 | -2.520641672 | 0.011714107 | 0.05621057 | down |
| LCN1P1 | 2.390295789 | 0.527941542 | 0.209471723 | 2.520347537 | 0.011723902 | 0.056241428 | up |
| SNORA70J | 1.001159007 | -0.856439003 | 0.34022457 | -2.517275585 | 0.011826628 | 0.056636729 | down |
| AC002480.1 | 0.951036036 | 0.505246497 | 0.200731075 | 2.517031787 | 0.011834815 | 0.056651596 | up |
| CDH7 | 11.41690493 | 0.681578852 | 0.270798398 | 2.516923499 | 0.011838453 | 0.0566609 | up |
| MTND1P9 | 0.889191579 | -0.725674768 | 0.288585001 | -2.514596272 | 0.011916876 | 0.056928101 | down |
| UBL4B | 0.884959078 | 0.599238646 | 0.238307604 | 2.514559479 | 0.01191812 | 0.056928101 | up |
| AC053503.4 | 8.420659435 | -0.698983898 | 0.27799227 | -2.514400486 | 0.011923495 | 0.056937077 | down |
| PHBP18 | 1.085907114 | -0.71680865 | 0.285422116 | -2.511398415 | 0.012025389 | 0.057316141 | down |
| CA6 | 4.791830572 | 0.845249629 | 0.336625723 | 2.510947828 | 0.012040749 | 0.057374564 | up |
| AC015712.4 | 0.648133298 | 0.869015749 | 0.34611093 | 2.510801231 | 0.01204575 | 0.057390217 | up |
| UNC93B5 | 1.709796534 | 0.501151985 | 0.199631884 | 2.510380479 | 0.012060114 | 0.057434103 | up |
| AL355922.5 | 2.966027049 | -0.81694968 | 0.325505316 | -2.509789053 | 0.01208033 | 0.057513998 | down |
| AC009292.2 | 0.543455014 | 0.582814028 | 0.232260932 | 2.509307194 | 0.012096823 | 0.057584323 | up |
| EVX1-AS | 20.53478588 | 0.58611798 | 0.233597559 | 2.509092911 | 0.012104164 | 0.057603149 | up |
| EZH2P1 | 0.74323166 | -0.874802845 | 0.348661158 | -2.509034413 | 0.012106169 | 0.057603149 | down |
| RNA5SP355 | 0.670788203 | -1.765592863 | 0.704044973 | -2.507784205 | 0.012149084 | 0.057767313 | down |
| COX7A2P2 | 4.839624118 | -0.615791412 | 0.245681291 | -2.506464414 | 0.012194533 | 0.057925765 | down |
| VN1R80P | 0.730423824 | -0.544793538 | 0.217448385 | -2.505392433 | 0.01223156 | 0.058068653 | down |
| DRC7 | 2.601261332 | 0.517065744 | 0.206400729 | 2.505154638 | 0.012239787 | 0.058075461 | up |
| LRRC53 | 1.235367938 | 0.952550063 | 0.380392167 | 2.504126385 | 0.012275418 | 0.058172045 | up |
| AC097493.2 | 1.736775774 | -0.542998125 | 0.216883203 | -2.503643056 | 0.012292198 | 0.058216029 | down |
| AC091144.2 | 0.587594827 | -0.874049135 | 0.349355197 | -2.501892464 | 0.012353144 | 0.058363262 | down |
| LRTM2 | 1.648240604 | 0.630876847 | 0.252164353 | 2.501847862 | 0.0123547 | 0.058363262 | up |
| OR10Q1 | 1.200547809 | 0.664804973 | 0.265752472 | 2.501594693 | 0.012363537 | 0.058388512 | up |
| FTLP6 | 0.4196002 | -0.8684332 | 0.3475515 | -2.498718 | 0.01246434 | NA | down |
| NUS1P2 | 1.388352102 | -0.6922911 | 0.277098534 | -2.498357137 | 0.012477042 | 0.058775147 | down |
| POU3F3 | 4.537362037 | 0.564262468 | 0.225895221 | 2.497894667 | 0.012493331 | 0.058827018 | up |
| AL121917.2 | 3.017039722 | -0.506561575 | 0.203166275 | -2.493334957 | 0.01265494 | 0.05937063 | down |
| AC007683.2 | 0.3773695 | -0.9392886 | 0.3767828 | -2.492918 | 0.01266982 | NA | down |
| DPP10 | 61.69304346 | -0.661957609 | 0.26558228 | -2.492476567 | 0.01268557 | 0.059472613 | down |
| AL162511.1 | 0.999300306 | -0.938483623 | 0.376588503 | -2.49206658 | 0.012700223 | 0.059516277 | down |
| AL035078.1 | 0.941566279 | -0.581758395 | 0.233779237 | -2.488494709 | 0.012828515 | 0.059972848 | down |
| AL031598.1 | 0.625347804 | 0.920664253 | 0.370003734 | 2.488256654 | 0.012837106 | 0.059972848 | up |
| NPM1P38 | 0.657147487 | 0.522775614 | 0.210102081 | 2.488198177 | 0.012839217 | 0.059974332 | up |
| PTGES3P4 | 5.831088307 | -0.521122033 | 0.209753459 | -2.484450249 | 0.012975164 | 0.060440514 | down |
| AF131215.2 | 0.845036702 | -0.788192308 | 0.317380185 | -2.483432635 | 0.013012294 | 0.060588156 | down |
| AC110491.1 | 0.4205473 | -1.106369 | 0.4455094 | -2.483378 | 0.01301427 | NA | down |
| AL137845.2 | 1.924531295 | -0.543721517 | 0.218993091 | -2.482824982 | 0.013034511 | 0.060674706 | down |
| AC138649.1 | 3.445080277 | -0.620257649 | 0.249876091 | -2.482260892 | 0.013055165 | 0.060728583 | down |
| SNORD116-1 | 0.3767114 | -1.22984 | 0.4954696 | -2.482169 | 0.01305852 | NA | down |
| LINC01120 | 0.662681586 | -0.717568725 | 0.289151405 | -2.481636651 | 0.013078055 | 0.060802953 | down |
| AL135978.1 | 0.816038071 | -0.523267323 | 0.21096356 | -2.480368284 | 0.013124674 | 0.060916341 | down |
| DCAF12L2 | 1.326710024 | -0.669474864 | 0.269966502 | -2.479844198 | 0.01314398 | 0.060963639 | down |
| UPK2 | 10.68616887 | -0.583035488 | 0.235203048 | -2.478860258 | 0.013180293 | 0.061112823 | down |
| CCKAR | 1.043012653 | 0.995952742 | 0.401797416 | 2.47874352 | 0.013184608 | 0.061118167 | up |
| AL133284.1 | 1.242443103 | -0.631539685 | 0.255220233 | -2.474489099 | 0.013342689 | 0.061679966 | down |
| AC004884.1 | 13.60126135 | -0.597900786 | 0.241639464 | -2.474350741 | 0.013347858 | 0.061689219 | down |
| CRISP2 | 0.478763257 | 1.658954253 | 0.67047759 | 2.4742874 | 0.013350225 | 0.061689219 | up |
| SLC25A39P1 | 2.02532372 | -0.613028424 | 0.247926557 | -2.472621053 | 0.013412628 | 0.061926231 | down |
| KERA | 3.909005605 | -0.526447892 | 0.213131408 | -2.47006247 | 0.013508946 | 0.062207747 | down |
| RNU6-42P | 0.542384109 | -0.726412032 | 0.294236091 | -2.468806694 | 0.013556443 | 0.062409279 | down |
| SP9 | 3.988198913 | 1.045872405 | 0.423791446 | 2.46789409 | 0.013591053 | 0.062527662 | up |
| RNA5SP141 | 1.604625821 | -1.46549612 | 0.593912283 | -2.46752957 | 0.013604898 | 0.062580661 | down |
| QRSL1P3 | 1.040280713 | -0.615299068 | 0.249375889 | -2.467355882 | 0.0136115 | 0.062602417 | down |
| IGHV3-60 | 8.988962185 | -0.539998142 | 0.218887647 | -2.467010587 | 0.013624633 | 0.062628364 | down |
| OLIG1 | 2.778366431 | -0.502531122 | 0.203716078 | -2.466821107 | 0.013631844 | 0.062644291 | down |
| TNXA | 0.970335793 | 0.627667308 | 0.254476558 | 2.466503451 | 0.013643941 | 0.062682655 | up |
| AL365226.2 | 454.2063286 | -0.611042044 | 0.247892601 | -2.464946686 | 0.013703364 | 0.062886537 | down |
| AC092666.1 | 0.972401486 | -0.655485848 | 0.266052104 | -2.46374991 | 0.013749201 | 0.063027694 | down |
| LAPTM4BP1 | 1.437937484 | -0.792192796 | 0.321618346 | -2.463145545 | 0.0137724 | 0.063090797 | down |
| MPC1L | 0.812906216 | -0.577308965 | 0.234502872 | -2.461841771 | 0.013822563 | 0.063268593 | down |
| CYP4Z1 | 3.475185983 | 0.562202966 | 0.228463026 | 2.460805042 | 0.013862567 | 0.063373542 | up |
| PRELID3BP10 | 1.579653912 | -0.55816785 | 0.226915979 | -2.459799665 | 0.013901459 | 0.063497888 | down |
| OSTCP6 | 0.965537516 | -0.813591597 | 0.33098345 | -2.458103558 | 0.013967289 | 0.063704326 | down |
| SNORA70B | 0.591904756 | -0.828147374 | 0.336920833 | -2.457988025 | 0.013971783 | 0.063716133 | down |
| AC018685.2 | 1.74659875 | -0.708710131 | 0.288415286 | -2.45725579 | 0.014000296 | 0.063820052 | down |
| AC009495.2 | 0.475593409 | 0.610049235 | 0.248486312 | 2.455061729 | 0.01408604 | 0.064089791 | up |
| AC078909.2 | 0.732370838 | 0.631515959 | 0.257334016 | 2.454071051 | 0.014124907 | 0.064213025 | up |
| AC017007.3 | 1.169639019 | -0.627150525 | 0.255620578 | -2.45344303 | 0.014149595 | 0.064299036 | down |
| LEFTY2 | 38.42037608 | -0.504521204 | 0.205758192 | -2.45201029 | 0.01420606 | 0.064485523 | down |
| AC092535.1 | 1.787695331 | -0.622739076 | 0.254107904 | -2.450687546 | 0.014258366 | 0.064635221 | down |
| DUXAP9 | 8.419184517 | -0.5128188 | 0.209268656 | -2.450528475 | 0.014264668 | 0.064646261 | down |
| IGHV1-14 | 5.987237796 | -0.562960796 | 0.229887215 | -2.44885648 | 0.014331054 | 0.06487678 | down |
| AL049647.1 | 0.634333922 | -0.75577662 | 0.308632922 | -2.448788082 | 0.014333775 | 0.064880317 | down |
| PCDHB1 | 0.677474604 | 0.790603014 | 0.322913823 | 2.448340574 | 0.014351593 | 0.064935533 | up |
| NPBWR1 | 1.921091169 | -0.601018426 | 0.245556406 | -2.447577871 | 0.014382004 | 0.065045795 | down |
| NFE4 | 1.244592644 | 0.553358266 | 0.226253127 | 2.445748585 | 0.014455176 | 0.065275711 | up |
| IGLV4-3 | 5.549233703 | -0.618094027 | 0.252729808 | -2.445671257 | 0.014458276 | 0.065275985 | down |
| TEX49 | 1.128655375 | 0.572910903 | 0.23430408 | 2.445159743 | 0.0144788 | 0.065337757 | up |
| SI | 395.4579094 | 0.541495107 | 0.221458117 | 2.445135513 | 0.014479772 | 0.065337757 | up |
| PRELID1P3 | 1.024677328 | -0.558541726 | 0.228587212 | -2.443451327 | 0.014547532 | 0.065599988 | down |
| ACTBP14 | 1.090272211 | 0.592373801 | 0.242438464 | 2.443398589 | 0.014549658 | 0.065599988 | up |
| TIMM17BP1 | 1.76196937 | -0.720025664 | 0.294882823 | -2.441734846 | 0.014616878 | 0.06579661 | down |
| AC079915.1 | 1.636001941 | -0.623666649 | 0.255445486 | -2.441486278 | 0.014626944 | 0.065826243 | down |
| AC004012.1 | 1.130024405 | 0.503290364 | 0.206308073 | 2.43950882 | 0.014707244 | 0.06606998 | up |
| ZSCAN23 | 4.862334618 | -0.524945197 | 0.215223987 | -2.439064549 | 0.014725338 | 0.066113135 | down |
| SNORA70G | 0.627194505 | -0.907349676 | 0.372012472 | -2.439030261 | 0.014726735 | 0.066113135 | down |
| LINC02050 | 0.693823899 | -1.128654897 | 0.463002476 | -2.437686526 | 0.014781586 | 0.066322348 | down |
| PNMA5 | 5.346567421 | 0.690553871 | 0.283392841 | 2.436737174 | 0.014820447 | 0.066453548 | up |
| RPL12P50 | 0.612323696 | -0.873221759 | 0.358372419 | -2.436632153 | 0.014824751 | 0.066458829 | down |
| PGM5P3-AS1 | 0.556684991 | 0.818716888 | 0.336262737 | 2.43475354 | 0.014901936 | 0.06672053 | up |
| DBIP1 | 2.637649822 | -0.670325927 | 0.275363491 | -2.434331164 | 0.014919338 | 0.066783699 | down |
| AC093001.1 | 0.763437006 | 0.892202699 | 0.367100695 | 2.430403186 | 0.015082035 | 0.067319584 | up |
| AC026333.3 | 0.801178224 | -0.85346608 | 0.351309326 | -2.429386346 | 0.015124406 | 0.067457453 | down |
| IGFALS | 58.25385901 | 0.547976059 | 0.225572848 | 2.429264265 | 0.0151295 | 0.067468411 | up |
| MSLNL | 10.89522319 | 0.622224705 | 0.256469196 | 2.42611867 | 0.015261279 | 0.067920207 | up |
| HORMAD2-AS1 | 0.923678413 | 0.502603759 | 0.207405966 | 2.423284968 | 0.015380856 | 0.06835232 | up |
| TPM4P1 | 0.805267294 | -1.014367978 | 0.418873981 | -2.421654302 | 0.01545004 | 0.068569878 | down |
| RNFT1P3 | 2.904092477 | -0.503734585 | 0.208013148 | -2.421647808 | 0.015450316 | 0.068569878 | down |
| AC009686.1 | 0.485025683 | -0.921932979 | 0.380826223 | -2.420875778 | 0.015483167 | 0.068657718 | down |
| RPSAP71 | 14.96343933 | -0.768645118 | 0.317533979 | -2.420670445 | 0.015491915 | 0.068681575 | down |
| S100A11P1 | 4.200062879 | -0.611455822 | 0.252624653 | -2.420412318 | 0.015502918 | 0.068712136 | down |
| AC063977.6 | 0.97450298 | 0.533757043 | 0.220593548 | 2.419640319 | 0.015535865 | 0.068803453 | up |
| AKAIN1 | 1.51900738 | -0.643867555 | 0.266176068 | -2.418953591 | 0.015565226 | 0.068887867 | down |
| PSG4 | 0.516980588 | -0.836179226 | 0.345892462 | -2.417454321 | 0.015629496 | 0.069097226 | down |
| GOLGA6L7P | 0.734021561 | -0.978544787 | 0.404789515 | -2.417416338 | 0.015631127 | 0.069097226 | down |
| PRSS48 | 1.782374923 | -0.584637489 | 0.241942435 | -2.416432197 | 0.015673445 | 0.069212664 | down |
| AC019330.1 | 5.165073834 | -0.512122333 | 0.212055117 | -2.415043502 | 0.01573333 | 0.069393076 | down |
| AC092139.4 | 2.60826744 | -0.591093107 | 0.24483042 | -2.41429601 | 0.015765648 | 0.069471478 | down |
| STX8P1 | 1.180536397 | -0.652187578 | 0.270827757 | -2.408126793 | 0.016034611 | 0.070369217 | down |
| AC109454.3 | 1.222778564 | 0.859676708 | 0.357319604 | 2.40590412 | 0.016132498 | 0.070631955 | up |
| HHATL | 0.881527705 | 0.7438877 | 0.309416023 | 2.404166708 | 0.016209379 | 0.0708665 | up |
| CCDC129 | 9.903729381 | 0.50490306 | 0.210033038 | 2.403922089 | 0.01622023 | 0.070901735 | up |
| FAM19A4 | 0.963764286 | 0.76195654 | 0.317030353 | 2.403418261 | 0.016242598 | 0.070946801 | up |
| ADH5P3 | 0.947278012 | -0.593777731 | 0.247086895 | -2.403113006 | 0.016256163 | 0.07096629 | down |
| RPL34P33 | 8.284962154 | -0.538465495 | 0.224085141 | -2.402950474 | 0.01626339 | 0.070972729 | down |
| XRCC6P5 | 2.086345323 | -0.562957659 | 0.234302877 | -2.40269205 | 0.016274886 | 0.070976587 | down |
| CCNB2P1 | 1.035263347 | -0.684838602 | 0.285139526 | -2.401766638 | 0.016316113 | 0.071119284 | down |
| AC074281.1 | 0.694342219 | -0.823597496 | 0.343070112 | -2.400668169 | 0.016365169 | 0.071286651 | down |
| LINC00402 | 2.667667316 | 0.520958743 | 0.217057718 | 2.40009315 | 0.0163909 | 0.071370846 | up |
| AC055748.1 | 1.025345454 | 0.539664116 | 0.224855824 | 2.400045089 | 0.016393052 | 0.071370924 | up |
| AC010894.1 | 0.592388146 | -1.008102024 | 0.420173548 | -2.399251521 | 0.016428626 | 0.071469971 | down |
| CNTNAP5 | 1.675965123 | -0.864610908 | 0.360457411 | -2.398649277 | 0.016455668 | 0.071559685 | down |
| BOK-AS1 | 25.63078501 | -0.924522967 | 0.385445015 | -2.398585871 | 0.016458517 | 0.07156277 | down |
| AL353997.2 | 1.196579895 | 0.651334026 | 0.271575513 | 2.398353293 | 0.016468972 | 0.071589614 | up |
| AC108112.1 | 1.175006348 | 0.701986391 | 0.292739508 | 2.397989928 | 0.016485319 | 0.071651357 | up |
| AC105109.1 | 0.405012 | -0.9480054 | 0.3956436 | -2.396109 | 0.01657014 | NA | down |
| LMO7DN | 0.706197997 | 0.556164281 | 0.232153455 | 2.395675222 | 0.016589783 | 0.071984266 | up |
| FBXL21 | 0.506735116 | -1.198358067 | 0.500298788 | -2.39528477 | 0.016607461 | 0.072023099 | down |
| Z82190.1 | 0.78977224 | -0.569426954 | 0.237773555 | -2.394828784 | 0.016628128 | 0.07206345 | down |
| NDUFB1P1 | 5.380709706 | -0.566266411 | 0.236479766 | -2.394566013 | 0.016640048 | 0.072089628 | down |
| Z98885.1 | 1.095327402 | -0.561631681 | 0.23462378 | -2.393754296 | 0.016676916 | 0.072239229 | down |
| AC011921.3 | 2.384673786 | -0.578623548 | 0.241791051 | -2.393072639 | 0.016707933 | 0.072346236 | down |
| RPL23AP14 | 1.144209705 | -0.550071766 | 0.230016619 | -2.39144358 | 0.016782263 | 0.072602285 | down |
| AL049830.4 | 0.605460287 | -0.900541867 | 0.376617835 | -2.391129105 | 0.016796645 | 0.072636314 | down |
| GNRHR2P1 | 0.565942627 | 0.692229766 | 0.289582284 | 2.390442385 | 0.016828089 | 0.072734669 | up |
| FDPSP5 | 2.708682795 | -0.556708765 | 0.232921248 | -2.39011585 | 0.016843059 | 0.072784137 | down |
| AC144836.1 | 0.827326213 | 0.508705272 | 0.212886693 | 2.389558804 | 0.016868623 | 0.072853368 | up |
| AC244196.3 | 1.861924916 | 0.554412556 | 0.232029246 | 2.389408088 | 0.016875546 | 0.072873854 | up |
| SLC2A2 | 2.726465248 | 0.674652472 | 0.282661805 | 2.38678329 | 0.016996507 | 0.073234305 | up |
| AC017101.1 | 0.640083346 | -0.769037813 | 0.322291471 | -2.386156265 | 0.017025515 | 0.073303753 | down |
| RN7SL45P | 0.627985521 | -0.685432182 | 0.287278134 | -2.385953197 | 0.017034919 | 0.073334799 | down |
| SEC14L4 | 21.29940655 | -0.607608098 | 0.254886594 | -2.383837013 | 0.017133189 | 0.073624916 | down |
| BTF3P13 | 7.422499281 | -0.541645052 | 0.227232765 | -2.383657356 | 0.017141554 | 0.073632719 | down |
| LINC01581 | 1.083019628 | -0.570686479 | 0.239524993 | -2.382575912 | 0.017191987 | 0.073754617 | down |
| AC015910.1 | 1.512727888 | 0.562538777 | 0.236201842 | 2.381601993 | 0.017237517 | 0.073921492 | up |
| FEZF1 | 85.43034832 | 0.563902647 | 0.236990705 | 2.379429376 | 0.017339465 | 0.074292 | up |
| OR2W3 | 7.900048264 | 0.547811346 | 0.230256546 | 2.379134733 | 0.017353332 | 0.07432668 | up |
| LHX1 | 2.471344482 | 0.778750382 | 0.327548779 | 2.377509648 | 0.017429987 | 0.074603378 | up |
| AC023512.1 | 4.116465408 | -0.533766587 | 0.224523479 | -2.377330819 | 0.01743844 | 0.074630009 | down |
| SIRPG-AS1 | 1.498102738 | 0.54130077 | 0.227717082 | 2.377075817 | 0.017450501 | 0.074672067 | up |
| TAS2R13 | 0.747821986 | -0.763750447 | 0.321617345 | -2.37471784 | 0.01756237 | 0.075045132 | down |
| LINC02512 | 3.434878908 | 0.705469447 | 0.29710323 | 2.374492685 | 0.017573084 | 0.075071732 | up |
| AC018630.2 | 1.678548583 | -0.520478123 | 0.219216391 | -2.374266459 | 0.017583856 | 0.075108153 | down |
| ANKRD44-IT1 | 0.768304884 | -0.715518303 | 0.30215415 | -2.368057179 | 0.017881772 | 0.075967428 | down |
| AC036164.1 | 3.219815269 | -0.72010201 | 0.304327359 | -2.366208591 | 0.017971316 | 0.076227648 | down |
| AC012456.2 | 0.4085019 | 0.6751701 | 0.2854572 | 2.365224 | 0.01801919 | NA | up |
| AL354751.1 | 0.693605785 | 0.653928836 | 0.276529166 | 2.364773474 | 0.018041102 | 0.076455772 | up |
| BCRP2 | 0.925417085 | -0.518693798 | 0.219452195 | -2.363584466 | 0.0180991 | 0.076652991 | down |
| HIST1H4L | 1.163889772 | -0.792556421 | 0.335331489 | -2.36350133 | 0.018103162 | 0.076660482 | down |
| RPL30P3 | 1.333936792 | -0.797352675 | 0.337384539 | -2.363334959 | 0.018111292 | 0.076685199 | down |
| AC010287.1 | 0.811376717 | -0.674945352 | 0.285889765 | -2.360858747 | 0.018232674 | 0.077082029 | down |
| ANKRD30B | 8.567075601 | 0.693562531 | 0.294014683 | 2.358938416 | 0.018327297 | 0.077413558 | up |
| AC110813.1 | 1.925284738 | -0.643353887 | 0.27279618 | -2.358368382 | 0.018355468 | 0.077483614 | down |
| AC122718.2 | 1.102569919 | -0.639520181 | 0.271486746 | -2.355622104 | 0.018491719 | 0.077881809 | down |
| RPS29P11 | 0.998339127 | -0.660582627 | 0.280532906 | -2.354742034 | 0.018535568 | 0.077976631 | down |
| HCAR3 | 29.36005423 | -0.524185199 | 0.222681178 | -2.353971731 | 0.018574024 | 0.078105692 | down |
| REG3A | 3474.638411 | 0.683844439 | 0.290513991 | 2.353912241 | 0.018576996 | 0.078105692 | up |
| TAS2R50 | 0.627906067 | -0.656819294 | 0.279114224 | -2.353227593 | 0.018611239 | 0.078207896 | down |
| MRLN | 13.59662573 | 0.766572037 | 0.32580329 | 2.352867699 | 0.018629261 | 0.078273793 | up |
| GMCL1P2 | 2.449039004 | 0.575385265 | 0.244673297 | 2.351647162 | 0.018690495 | 0.07845222 | up |
| BMS1P8 | 1.046504185 | 0.635099311 | 0.270105277 | 2.351302857 | 0.0187078 | 0.078485453 | up |
| RPS18P1 | 6.449562333 | -0.643367025 | 0.273633295 | -2.351201542 | 0.018712895 | 0.078486523 | down |
| AP003465.1 | 0.3925786 | -0.5753981 | 0.2447971 | -2.35051 | 0.01874768 | NA | down |
| AC053503.6 | 3.346582682 | -0.706127981 | 0.300416449 | -2.350497065 | 0.018748356 | 0.078555673 | down |
| SDHCP2 | 2.031482716 | -0.60330224 | 0.256763721 | -2.349639729 | 0.01879159 | 0.078675969 | down |
| MT-TM | 35.24844022 | -0.551173449 | 0.234582056 | -2.349597661 | 0.018793713 | 0.078675969 | down |
| CLPSL1 | 0.840496085 | -1.234181877 | 0.525669897 | -2.347826807 | 0.018883299 | 0.078954291 | down |
| FO393422.1 | 0.3263296 | -0.8866909 | 0.3778778 | -2.346502 | 0.01895058 | NA | down |
| RNU6ATAC39P | 0.511169005 | -0.732773011 | 0.312413773 | -2.34552083 | 0.019000517 | 0.079275893 | down |
| AC005183.1 | 1.049654867 | -0.692220816 | 0.295264775 | -2.344407038 | 0.019057361 | 0.07945864 | down |
| AC007319.1 | 1.961153161 | -0.520699996 | 0.222175218 | -2.34364571 | 0.019096301 | 0.079546514 | down |
| CUL1P1 | 0.689698671 | -0.70201252 | 0.299575857 | -2.343354789 | 0.0191112 | 0.079578837 | down |
| SLC13A1 | 1.247001515 | 0.99598865 | 0.425622692 | 2.340074129 | 0.019279913 | 0.080081923 | up |
| LKAAEAR1 | 1.073614983 | -0.643998108 | 0.275211965 | -2.340007665 | 0.019283344 | 0.080085517 | down |
| MTCO3P13 | 1.082184364 | 0.542197496 | 0.231746704 | 2.339612543 | 0.019303755 | 0.080125771 | up |
| FOXG1 | 11.98131867 | -0.974103127 | 0.416497474 | -2.338797201 | 0.019345932 | 0.080242781 | down |
| AC009163.2 | 4.300007122 | 0.534194488 | 0.22842127 | 2.338637242 | 0.019354216 | 0.080243022 | up |
| SERPINB4 | 3.046486345 | 0.955084672 | 0.408638862 | 2.337234075 | 0.019427016 | 0.080443147 | up |
| LINC02261 | 0.3876024 | -0.7821459 | 0.3346628 | -2.337116 | 0.01943315 | NA | down |
| PAH | 130.5673599 | -0.547781312 | 0.234470747 | -2.336245863 | 0.019478431 | 0.080592067 | down |
| OR5G3 | 1.322075923 | -0.583220418 | 0.249642912 | -2.336218618 | 0.01947985 | 0.080592067 | down |
| AL034399.2 | 0.4192502 | -0.9367371 | 0.4009756 | -2.336145 | 0.01948371 | NA | down |
| RNVU1-7 | 1.274004292 | -0.804048526 | 0.344317753 | -2.335193348 | 0.019533322 | 0.08077332 | down |
| OGN | 78.16791611 | -0.514307579 | 0.220294948 | -2.334631744 | 0.019562667 | 0.080844679 | down |
| AL117378.1 | 0.490469596 | 0.947813295 | 0.406049828 | 2.334229026 | 0.019583733 | 0.080911738 | up |
| AC087588.1 | 3.225096408 | -0.794493716 | 0.340423334 | -2.33384036 | 0.019604083 | 0.080975806 | down |
| AC130472.1 | 6.326929298 | -0.605549593 | 0.259653694 | -2.332143188 | 0.01969316 | 0.0811869 | down |
| AC079316.2 | 0.63644258 | -1.100244144 | 0.472190716 | -2.330084238 | 0.019801699 | 0.081470057 | down |
| FAM9C | 0.902886888 | -0.536237548 | 0.230186063 | -2.32958304 | 0.019828199 | 0.08154519 | down |
| AL121972.1 | 1.438886666 | -0.547287561 | 0.235321053 | -2.325705903 | 0.020034245 | 0.082113641 | down |
| PCNPP3 | 0.450695312 | -0.641657209 | 0.275912371 | -2.325583323 | 0.02004079 | 0.0821304 | down |
| BRD9P2 | 1.740446164 | 0.519640273 | 0.223522237 | 2.32478111 | 0.020083668 | 0.082265792 | up |
| UBE2V2P1 | 1.007768039 | 0.885418586 | 0.380880229 | 2.32466408 | 0.020089929 | 0.082281363 | up |
| AC092834.1 | 1.810427859 | -0.611985701 | 0.263312407 | -2.324181029 | 0.020115794 | 0.082377205 | down |
| LINC01983 | 0.941658041 | 0.663077268 | 0.28530277 | 2.324117881 | 0.020119177 | 0.082380972 | up |
| RNA5SP183 | 1.405853404 | -1.125088189 | 0.484255971 | -2.323333643 | 0.020161236 | 0.08245223 | down |
| IGKV1OR1-1 | 1.014051667 | -0.682947378 | 0.294082432 | -2.322299135 | 0.020216834 | 0.082598796 | down |
| AC012501.2 | 29.49692074 | -0.655541173 | 0.282419067 | -2.321164717 | 0.020277956 | 0.082818163 | down |
| RNU6-204P | 0.450626351 | -0.609820398 | 0.263033385 | -2.318414437 | 0.020426808 | 0.083232959 | down |
| TBC1D3L | 7.420494667 | 0.570579938 | 0.246180794 | 2.317727267 | 0.020464148 | 0.083344435 | up |
| LINC02235 | 0.3782767 | 0.5937612 | 0.256238 | 2.317225 | 0.02049147 | NA | up |
| AC009803.1 | 0.4171664 | -0.7301081 | 0.3153712 | -2.315075 | 0.02060882 | NA | down |
| RNA5SP473 | 0.3589052 | 0.7724378 | 0.3337352 | 2.314523 | 0.02063903 | NA | up |
| AP000785.1 | 8.985608489 | -0.577596085 | 0.249635115 | -2.313761371 | 0.020680815 | 0.084022251 | down |
| AC089983.1 | 1.500737001 | 0.514685314 | 0.222507529 | 2.313114154 | 0.020716364 | 0.084125795 | up |
| AC096746.1 | 0.823824562 | -0.756035047 | 0.327019455 | -2.311896238 | 0.020783402 | 0.084346814 | down |
| TEKT1 | 1.210101228 | 0.593087956 | 0.256559886 | 2.311694027 | 0.020794551 | 0.084371581 | up |
| CD177P1 | 2.188912258 | -0.696896389 | 0.3014895 | -2.311511311 | 0.020804629 | 0.084402232 | down |
| LINC01187 | 0.926590461 | 0.822501656 | 0.355853608 | 2.311348369 | 0.02081362 | 0.084412319 | up |
| AC009511.2 | 0.3935609 | -0.607912 | 0.2630903 | -2.310659 | 0.02085168 | NA | down |
| PPAN-P2RY11 | 0.536835921 | 0.539889585 | 0.233707059 | 2.310112442 | 0.02088193 | 0.084643953 | up |
| RN7SL300P | 0.3275722 | -1.394401 | 0.6036374 | -2.309998 | 0.02088825 | NA | down |
| AC138409.1 | 9.730319601 | -0.509602109 | 0.220692247 | -2.309107434 | 0.02093762 | 0.084808014 | down |
| C4BPAP1 | 0.824253727 | 0.917266621 | 0.397500102 | 2.307588392 | 0.021022041 | 0.085026375 | up |
| RNF225 | 1.50589068 | -0.615169912 | 0.266616846 | -2.307318238 | 0.021037086 | 0.085076935 | down |
| LIX1 | 2.817860433 | 0.555312853 | 0.240751785 | 2.306578342 | 0.021078338 | 0.085171665 | up |
| AL034369.1 | 1.09608055 | -0.766467534 | 0.332308405 | -2.306494575 | 0.021083013 | 0.085180263 | down |
| AC023034.1 | 0.52343059 | 0.923984652 | 0.400636919 | 2.306289334 | 0.021094471 | 0.085204872 | up |
| SAGE1 | 1.331302175 | 1.042411317 | 0.452208134 | 2.305158264 | 0.021157711 | 0.085409829 | up |
| AC006237.1 | 1.207670615 | 0.511109831 | 0.221932977 | 2.30299182 | 0.021279303 | 0.085797104 | up |
| RARRES2P1 | 0.858443864 | 0.587209586 | 0.255069483 | 2.302155399 | 0.02132641 | 0.085935231 | up |
| AC011483.2 | 1.03679773 | 0.675472851 | 0.293422522 | 2.302048412 | 0.021332442 | 0.085949181 | up |
| ZPBP | 0.90620042 | -0.767011246 | 0.333210558 | -2.301881581 | 0.021341851 | 0.085975761 | down |
| AP001885.1 | 1.305125813 | -0.580411333 | 0.252230296 | -2.301116649 | 0.021385039 | 0.08610922 | down |
| AC026254.2 | 33.72861948 | -0.581199057 | 0.252634617 | -2.300551934 | 0.021416971 | 0.08617554 | down |
| CELA3A | 2.141304561 | 0.556296062 | 0.241876459 | 2.299918164 | 0.021452857 | 0.086243717 | up |
| AL590240.3 | 0.4221325 | -0.7382156 | 0.3211547 | -2.298629 | 0.02152599 | NA | down |
| SIK1 | 45.6484007 | 0.504073215 | 0.219329584 | 2.298245436 | 0.021547824 | 0.086566649 | up |
| AL034405.1 | 0.827708554 | 0.540736158 | 0.235496815 | 2.296150625 | 0.021667271 | 0.086900359 | up |
| APOA1 | 22.35122407 | 0.509427637 | 0.222071147 | 2.293983907 | 0.021791423 | 0.087335448 | up |
| AP000526.1 | 1.009971948 | -0.698352726 | 0.304484616 | -2.293556684 | 0.021815976 | 0.087384526 | down |
| GABRA3 | 17.66942453 | -0.729537082 | 0.318244141 | -2.292381817 | 0.02188362 | 0.087609475 | down |
| MMP20 | 6.226516493 | -0.500493772 | 0.218361435 | -2.292042881 | 0.021903169 | 0.087657237 | down |
| TPRG1LP1 | 2.300039709 | 0.547485561 | 0.238880965 | 2.291876045 | 0.021912797 | 0.087674784 | up |
| NPY6R | 25.3967679 | 0.632446885 | 0.276068362 | 2.290906792 | 0.021968805 | 0.087839991 | up |
| RNF113B | 0.7836304 | -0.707402624 | 0.308814896 | -2.290701109 | 0.021980706 | 0.087872899 | down |
| A4GNT | 0.644185723 | 0.570970843 | 0.249493447 | 2.288520395 | 0.022107234 | 0.088231054 | up |
| PDCL3P3 | 0.680435167 | 0.524332515 | 0.229136196 | 2.28830069 | 0.022120016 | 0.088271535 | up |
| MIR199A2 | 0.572384313 | -0.70632538 | 0.308764253 | -2.287587936 | 0.022161529 | 0.088395002 | down |
| AC015920.1 | 0.442956241 | -0.868313992 | 0.37959537 | -2.287472558 | 0.022168255 | 0.088411285 | down |
| AC016027.3 | 0.615507708 | -0.89056286 | 0.389376027 | -2.287153801 | 0.022186848 | 0.088451038 | down |
| SAP18P2 | 2.173429774 | -0.611390499 | 0.267345955 | -2.28688891 | 0.022202308 | 0.088473235 | down |
| AC004253.2 | 0.3843293 | -0.6976277 | 0.3053433 | -2.284732 | 0.02232852 | NA | down |
| EI24P2 | 6.922755622 | -0.550197518 | 0.240865042 | -2.284256416 | 0.022356466 | 0.088896857 | down |
| AC078962.3 | 0.3881194 | -1.078085 | 0.4719922 | -2.284115 | 0.02236476 | NA | down |
| AC084871.1 | 0.968710176 | -0.599575541 | 0.262637539 | -2.282901152 | 0.022436192 | 0.089103771 | down |
| GMNC | 0.650288553 | -0.935473749 | 0.40986379 | -2.28240155 | 0.022465644 | 0.089133967 | down |
| RN7SL502P | 0.584015175 | -0.775807562 | 0.339959654 | -2.282057745 | 0.022485931 | 0.089188949 | down |
| CCDC177 | 1.007152464 | 0.623557594 | 0.273266647 | 2.281864988 | 0.022497312 | 0.089202351 | up |
| IFNE | 3.83819541 | 0.65061325 | 0.285220839 | 2.281085955 | 0.022543361 | 0.089363743 | up |
| UPK1A-AS1 | 1.057382878 | 0.58950643 | 0.258447269 | 2.28095438 | 0.022551147 | 0.08938401 | up |
| KIRREL1-IT1 | 0.4208159 | -0.8420482 | 0.3692271 | -2.28057 | 0.02257392 | NA | down |
| ROPN1 | 1.265919684 | -0.646590612 | 0.283599826 | -2.279940091 | 0.022611242 | 0.089558515 | down |
| AL355482.2 | 2.711471644 | -0.764519528 | 0.335414711 | -2.279326165 | 0.022647684 | 0.089649369 | down |
| RNU2-59P | 0.3462077 | -1.205191 | 0.528923 | -2.278576 | 0.02269227 | NA | down |
| AL589743.5 | 2.843966161 | -0.518077225 | 0.227372137 | -2.278543153 | 0.022694236 | 0.089780902 | down |
| AL162393.1 | 1.166521502 | -0.73885176 | 0.324510641 | -2.276818282 | 0.022797079 | 0.090059909 | down |
| AC000367.1 | 0.667845428 | -0.649562129 | 0.285313742 | -2.276659105 | 0.02280659 | 0.09008684 | down |
| LINC00443 | 0.3076407 | 0.8217678 | 0.3609942 | 2.276402 | 0.02282198 | NA | up |
| AC005537.1 | 5.567894295 | 0.572677816 | 0.251609137 | 2.276061284 | 0.022842342 | 0.090170018 | up |
| AC233982.1 | 0.770709942 | -0.75845929 | 0.333235766 | -2.276044071 | 0.022843372 | 0.090170018 | down |
| RNU4-38P | 0.572487965 | -0.699359839 | 0.30742439 | -2.274900309 | 0.022911911 | 0.090356607 | down |
| AP005121.2 | 0.3232326 | 0.8425936 | 0.3705581 | 2.27385 | 0.02297499 | NA | up |
| AL035045.1 | 1.999955798 | -0.503920495 | 0.221684175 | -2.273145996 | 0.023017383 | 0.090661739 | down |
| AC245407.1 | 0.916466324 | -0.525782059 | 0.231385571 | -2.272319992 | 0.02306719 | 0.090815859 | down |
| MYMK | 0.891232978 | 0.718310345 | 0.3161767 | 2.271863626 | 0.023094748 | 0.090881558 | up |
| LINC01213 | 3.758949621 | -0.502295612 | 0.221101414 | -2.271788334 | 0.023099298 | 0.09088525 | down |
| AL161645.1 | 0.955168932 | -0.58083931 | 0.255714611 | -2.271435758 | 0.023120612 | 0.090951227 | down |
| AC100843.2 | 0.328481 | 0.6741098 | 0.2967773 | 2.271433 | 0.02312076 | NA | up |
| SPINK13 | 1.417603905 | -0.66236244 | 0.29180487 | -2.269881377 | 0.023214781 | 0.091128713 | down |
| MT-TS2 | 2.568832435 | -0.580362765 | 0.255702748 | -2.269677469 | 0.023227159 | 0.091136595 | down |
| WFDC5 | 0.956570347 | 0.547274741 | 0.241163441 | 2.269310547 | 0.023249448 | 0.091189864 | up |
| AC008551.1 | 4.096072285 | -0.60887017 | 0.26833525 | -2.269065173 | 0.023264363 | 0.091226965 | down |
| LINC00626 | 0.897840833 | -0.774683099 | 0.34145885 | -2.268745114 | 0.02328383 | 0.091292598 | down |
| AP000904.1 | 1.404438497 | -0.555628743 | 0.244930032 | -2.268520277 | 0.023297515 | 0.091329931 | down |
| TMEM210 | 2.792412894 | 0.595059203 | 0.262416844 | 2.267610545 | 0.023352954 | 0.091499256 | up |
| LINC01098 | 0.700487335 | 0.867505364 | 0.382816885 | 2.266110504 | 0.023444618 | 0.091761643 | up |
| SNORA11B | 0.4291847 | -0.9376436 | 0.413829 | -2.265776 | 0.02346511 | NA | down |
| TET2-AS1 | 0.826450556 | -0.598152135 | 0.263995994 | -2.26576217 | 0.023465949 | 0.091802152 | down |
| RAD1P1 | 0.446104639 | -0.879660707 | 0.388412259 | -2.264760413 | 0.023527386 | 0.091956441 | down |
| C2orf91 | 1.649108614 | 0.532079135 | 0.235068301 | 2.26350866 | 0.023604351 | 0.092177578 | up |
| AC019117.2 | 0.782077838 | 0.528507764 | 0.233493301 | 2.263481481 | 0.023606025 | 0.092177578 | up |
| MIR3681HG | 1.645031685 | 0.531856093 | 0.235048474 | 2.262750678 | 0.023651063 | 0.092306251 | up |
| AC022240.1 | 0.3654239 | 0.9952969 | 0.4402628 | 2.260688 | 0.02377856 | NA | up |
| TPT1P12 | 17.02793029 | -0.609412987 | 0.269695357 | -2.259634699 | 0.023843932 | 0.092835596 | down |
| RN7SL165P | 0.363469 | 0.6345455 | 0.2811454 | 2.257001 | 0.02400802 | NA | up |
| EMX2OS | 8.276635964 | -0.546985416 | 0.242504082 | -2.255571997 | 0.024097449 | 0.09357203 | down |
| HSPE1P9 | 0.638820311 | -0.602465705 | 0.267205858 | -2.25468749 | 0.024152951 | 0.093700562 | down |
| CDH16 | 55.09501875 | 0.55582301 | 0.246668532 | 2.253319491 | 0.024239012 | 0.093936353 | up |
| PDCD6IPP1 | 2.574386054 | 0.549978116 | 0.244097764 | 2.253105921 | 0.024252471 | 0.093966735 | up |
| AL357055.1 | 14.69447779 | -0.556047483 | 0.246809418 | -2.252942721 | 0.024262761 | 0.093990628 | down |
| MIR554 | 0.439171349 | -0.701381455 | 0.311381522 | -2.252482584 | 0.024291792 | 0.094086382 | down |
| AC026111.1 | 2.828756141 | -0.532786789 | 0.236579189 | -2.252044195 | 0.024319479 | 0.094115157 | down |
| SIX3 | 10.44250062 | 0.679571598 | 0.301762451 | 2.252008475 | 0.024321736 | 0.094115157 | up |
| AC011369.2 | 0.447418346 | -1.36728172 | 0.607469808 | -2.250781357 | 0.024399389 | 0.09428472 | down |
| AL034374.1 | 1.530365476 | -0.542769869 | 0.241156952 | -2.250691359 | 0.024405092 | 0.09428497 | down |
| LINC01779 | 0.542018606 | -0.519188639 | 0.23073151 | -2.25018524 | 0.024437189 | 0.094354466 | down |
| RN7SL753P | 0.569507522 | -0.596913989 | 0.265313524 | -2.249843807 | 0.024458862 | 0.094405449 | down |
| AC098818.1 | 1.497870502 | -0.691925111 | 0.307891346 | -2.247302889 | 0.024620676 | 0.094887638 | down |
| AC104390.1 | 2.354606184 | -0.501496969 | 0.223229684 | -2.246551446 | 0.024668708 | 0.095039893 | down |
| AC131182.1 | 0.779700926 | -0.757467309 | 0.337287462 | -2.245761835 | 0.024719267 | 0.095174106 | down |
| VPREB1 | 1.134267936 | -0.960957609 | 0.427919218 | -2.245651906 | 0.024726313 | 0.095174106 | down |
| RPL23AP23 | 0.4222887 | -0.871635 | 0.388164 | -2.245533 | 0.02473394 | NA | down |
| AC138058.1 | 0.652545011 | -0.9280643 | 0.413704109 | -2.243304527 | 0.024877184 | 0.095644734 | down |
| AC103724.4 | 0.722337539 | 0.510947164 | 0.227945719 | 2.24152999 | 0.024991765 | 0.095953975 | up |
| RLIMP1 | 0.990832839 | -0.591218119 | 0.263782995 | -2.241304902 | 0.025006332 | 0.09598677 | down |
| NKX2-5 | 1.661742077 | 0.665887345 | 0.297392288 | 2.239087467 | 0.025150226 | 0.096373175 | up |
| AL133467.4 | 1.51130568 | 1.380783635 | 0.616861047 | 2.238403029 | 0.025194785 | 0.096513796 | up |
| RPL37P3 | 0.719098769 | -0.912289263 | 0.407802682 | -2.237085 | 0.025280785 | 0.096784744 | down |
| AL109930.1 | 0.3252443 | -0.8736752 | 0.3905887 | -2.236817 | 0.02529833 | NA | down |
| SCARNA3 | 0.528673009 | -1.204478493 | 0.538689604 | -2.235941596 | 0.025355597 | 0.096953478 | down |
| AC018470.1 | 0.618549224 | 1.107221934 | 0.495198593 | 2.235914942 | 0.025357343 | 0.096953478 | up |
| AC007849.1 | 1.369627038 | -0.705573581 | 0.315850757 | -2.233882821 | 0.025490784 | 0.097343532 | down |
| EIF2S2P5 | 0.3891753 | -0.904553 | 0.405263 | -2.232015 | 0.02561398 | NA | down |
| AC055811.3 | 1.022198731 | -0.525350956 | 0.235448741 | -2.231275284 | 0.0256629 | 0.097768857 | down |
| LINC02395 | 1.298248724 | -0.604469578 | 0.271145179 | -2.22932076 | 0.02579257 | 0.098148537 | down |
| AC006994.1 | 0.804853859 | -0.575590372 | 0.258330937 | -2.228112426 | 0.025873019 | 0.098353941 | down |
| AC116049.2 | 3.39223718 | -0.535027171 | 0.240140487 | -2.227975703 | 0.025882135 | 0.098377414 | down |
| AL031005.1 | 1.284789224 | -0.532726419 | 0.239133274 | -2.227738572 | 0.025897953 | 0.09842635 | down |
| AL160274.1 | 0.607362515 | -0.707871435 | 0.317792813 | -2.227462061 | 0.025916409 | 0.098474108 | down |
| AC068858.1 | 0.642649752 | -0.628142913 | 0.282102047 | -2.226651379 | 0.025970582 | 0.098616708 | down |
| AL162584.1 | 0.50965896 | -1.05868964 | 0.475550706 | -2.226239236 | 0.025998161 | 0.098683826 | down |
| CHRNA2 | 0.689058781 | -0.678299027 | 0.304710167 | -2.226046587 | 0.026011061 | 0.098710383 | down |
| AL445437.1 | 1.160494065 | 0.556936676 | 0.250306514 | 2.225018705 | 0.026079982 | 0.09892703 | up |
| MIR181A1HG | 1.070406391 | -0.611781871 | 0.275069579 | -2.22409862 | 0.02614181 | 0.099037981 | down |
| ALDH1L1-AS2 | 5.538670648 | 0.579318421 | 0.260508447 | 2.223798989 | 0.026161971 | 0.099079803 | up |
| KCNJ13 | 2.457506283 | 0.640399378 | 0.287981486 | 2.223751909 | 0.026165141 | 0.099079803 | up |
| ZNF98 | 0.725735165 | 0.589262375 | 0.265039103 | 2.223303539 | 0.026195339 | 0.099150915 | up |
| CR381670.1 | 5.184038231 | -0.543140048 | 0.244445295 | -2.221928829 | 0.026288116 | 0.099400823 | down |
| AC090559.2 | 0.672230207 | -0.755153699 | 0.339991899 | -2.221093212 | 0.026344649 | 0.099551377 | down |
| RPL30P13 | 0.468638934 | -0.906548566 | 0.408179118 | -2.220957727 | 0.026353825 | 0.099560165 | down |
| VN1R51P | 0.3831496 | 0.7618116 | 0.343035 | 2.220799 | 0.02636461 | NA | up |
| RNU4-53P | 0.566594105 | 0.506096728 | 0.227955433 | 2.220156464 | 0.026408148 | 0.099696872 | up |
| RNA5SP195 | 0.758235377 | -0.760343474 | 0.342625199 | -2.219169743 | 0.026475179 | 0.09984831 | down |
| USP32P2 | 1.528229135 | 0.605671769 | 0.272960681 | 2.218897483 | 0.0264937 | 0.099876875 | up |
| FAM95B1 | 1.10098966 | 0.509224425 | 0.229666628 | 2.217232996 | 0.026607174 | 0.100188098 | up |
| AL731577.1 | 0.678131018 | -0.745766658 | 0.336361995 | -2.217154936 | 0.026612506 | 0.100196893 | down |
| AC112196.1 | 0.486868797 | -0.840267159 | 0.378993809 | -2.217099964 | 0.026616261 | 0.100199751 | down |
| AC092324.1 | 2.851125613 | -0.664122815 | 0.299653009 | -2.216306176 | 0.02667054 | 0.100370194 | down |
| AC104984.2 | 1.046861613 | -0.516241768 | 0.232990281 | -2.215722327 | 0.026710525 | 0.100486743 | down |
| TRIM26BP | 0.2635842 | 0.6557875 | 0.295983 | 2.215625 | 0.02671717 | NA | up |
| H2AFZP4 | 3.820120468 | -0.533148435 | 0.240996871 | -2.212262893 | 0.026948505 | 0.101159419 | down |
| AC073525.1 | 0.562596705 | 0.929031144 | 0.420062295 | 2.211650878 | 0.026990797 | 0.101245008 | up |
| Z99127.2 | 0.524711968 | -0.760712222 | 0.34417809 | -2.210228499 | 0.027089308 | 0.101534807 | down |
| GABRR1 | 12.18726121 | -0.696733622 | 0.315277129 | -2.209908547 | 0.02711151 | 0.101554664 | down |
| AL132655.2 | 2.686230501 | -0.502996076 | 0.227611407 | -2.209889577 | 0.027112827 | 0.101554664 | down |
| LINC02003 | 2.043733049 | 0.522534593 | 0.236494454 | 2.209500405 | 0.027139854 | 0.101620908 | up |
| HHATL-AS1 | 0.2864213 | 0.694934 | 0.3147866 | 2.207636 | 0.02726968 | NA | up |
| PRDX2P3 | 1.181357807 | -0.50839323 | 0.230330042 | -2.207238037 | 0.027297434 | 0.102006199 | down |
| AC130365.1 | 4.266029196 | -0.556652672 | 0.25243644 | -2.205120114 | 0.027445667 | 0.102445647 | down |
| AC123912.2 | 0.473637907 | -0.689715868 | 0.312928158 | -2.204070967 | 0.027519354 | 0.102640498 | down |
| ANAPC10P1 | 0.710607848 | -0.53813899 | 0.244185195 | -2.203814973 | 0.027537359 | 0.102684749 | down |
| POU5F1P4 | 1.07928357 | -0.511567298 | 0.232291329 | -2.202266006 | 0.027646524 | 0.102919671 | down |
| AC087312.1 | 0.4284887 | -0.8492104 | 0.3857752 | -2.201309 | 0.02771416 | NA | down |
| AC100788.1 | 0.658391024 | -0.683741992 | 0.310628915 | -2.201153724 | 0.027725143 | 0.103097577 | down |
| OPRM1 | 0.2794004 | -0.9139526 | 0.4153651 | -2.20036 | 0.02778138 | NA | down |
| PRPF38AP1 | 0.835441764 | -0.674990735 | 0.30706353 | -2.198211993 | 0.027934002 | 0.103655235 | down |
| CCL1 | 0.912831627 | 0.572079512 | 0.260326675 | 2.197544729 | 0.027981566 | 0.103774153 | up |
| AC007255.2 | 0.432160238 | -0.654642419 | 0.298167149 | -2.195555147 | 0.0281238 | 0.104214006 | down |
| SNORA31 | 0.606051694 | -0.505849266 | 0.230428062 | -2.195258949 | 0.028145028 | 0.104245825 | down |
| RNU6-36P | 0.2929545 | -1.277509 | 0.5821477 | -2.194476 | 0.02820118 | NA | down |
| RIMBP3C | 0.534068922 | 0.570356487 | 0.260008377 | 2.19360812 | 0.028263594 | 0.10456575 | up |
| AC018946.1 | 0.543837165 | -0.74753039 | 0.340809378 | -2.193397362 | 0.028278762 | 0.104597952 | down |
| LINC00683 | 1.478807562 | 0.594989722 | 0.271280166 | 2.193266577 | 0.028288178 | 0.104609639 | up |
| AC026401.1 | 0.576694728 | -0.610665926 | 0.278557336 | -2.192244999 | 0.028361821 | 0.104800845 | down |
| AC002550.1 | 0.3914185 | -0.6237967 | 0.2845791 | -2.191998 | 0.02837967 | NA | down |
| TUBB4BP2 | 0.492058483 | -0.680817302 | 0.310621559 | -2.191790238 | 0.028394656 | 0.104898995 | down |
| AC025647.1 | 0.74141476 | -0.533836786 | 0.243611373 | -2.191345914 | 0.02842677 | 0.104959657 | down |
| LINC00404 | 1.057743442 | 0.839014176 | 0.38290472 | 2.191182639 | 0.028438578 | 0.104980075 | up |
| AC098936.1 | 1.273998876 | 0.555489455 | 0.253688737 | 2.189649659 | 0.028549655 | 0.10528551 | up |
| AC015853.2 | 0.4077547 | 0.8211734 | 0.3750305 | 2.189618 | 0.02855196 | NA | up |
| AC093677.1 | 1.335542648 | -0.638679743 | 0.291700459 | -2.189505446 | 0.028560123 | 0.105300891 | down |
| LINC02275 | 1.099093165 | -0.530038905 | 0.242111085 | -2.18923849 | 0.02857951 | 0.105349141 | down |
| TRIM71 | 21.46748949 | 0.658378368 | 0.300805651 | 2.188716752 | 0.028617434 | 0.10545406 | up |
| CLLU1 | 4.355789171 | -0.607420272 | 0.277632681 | -2.187855802 | 0.028680108 | 0.105638449 | down |
| KLF3P1 | 0.616209777 | -0.631707248 | 0.28876272 | -2.187634355 | 0.028696247 | 0.105686256 | down |
| AP001056.1 | 0.734910235 | 0.535727944 | 0.244914087 | 2.187411718 | 0.028712482 | 0.105734401 | up |
| XAGE2 | 1.909708194 | -1.19563351 | 0.547107899 | -2.185370584 | 0.028861686 | 0.106112956 | down |
| AP000251.1 | 10.68328123 | 0.528325665 | 0.241813009 | 2.184852116 | 0.028899691 | 0.106189933 | up |
| PITPNM2-AS1 | 2.066490614 | -0.559569084 | 0.256331968 | -2.182985949 | 0.029036845 | 0.106576789 | down |
| AC131009.2 | 0.65804432 | -0.55485949 | 0.254357874 | -2.181412672 | 0.029152907 | 0.106944095 | down |
| ARHGAP26-IT1 | 0.520312241 | -0.787308345 | 0.360990567 | -2.180966535 | 0.029185892 | 0.107029872 | down |
| LINC01882 | 2.288347148 | 0.628608791 | 0.288296265 | 2.180426412 | 0.029225868 | 0.107129481 | up |
| ATP6V1B1-AS1 | 0.615998459 | 0.754873987 | 0.346371157 | 2.179378888 | 0.029303533 | 0.107331813 | up |
| AC110998.1 | 1.952717894 | -0.505783406 | 0.232152641 | -2.178667467 | 0.02935638 | 0.107478291 | down |
| ADRB3 | 2.943127078 | -0.543991393 | 0.249751781 | -2.178128188 | 0.029396495 | 0.107613374 | down |
| AC099494.3 | 0.808779895 | -0.561280447 | 0.257705796 | -2.177989224 | 0.029406839 | 0.107627679 | down |
| AC011270.1 | 0.56389461 | -0.551692093 | 0.253309898 | -2.17793342 | 0.029410994 | 0.107631106 | down |
| AC074085.2 | 69.51522218 | -0.50363959 | 0.231405456 | -2.176437837 | 0.029522537 | 0.107838685 | down |
| ZNF556 | 25.33482013 | 0.590767691 | 0.271535853 | 2.175652623 | 0.029581245 | 0.107980883 | up |
| ASTN2-AS1 | 0.371108 | -0.9998313 | 0.4596647 | -2.175132 | 0.02962026 | NA | down |
| AL008733.1 | 1.416820197 | 0.615253964 | 0.282954747 | 2.17438997 | 0.029675861 | 0.108221424 | up |
| AC139085.1 | 1.0229963 | -0.570153489 | 0.262237225 | -2.174189756 | 0.029690887 | 0.108264417 | down |
| AL359636.2 | 1.44953913 | -0.593197538 | 0.273604686 | -2.168082521 | 0.030152408 | 0.109565 | down |
| ATP5LP3 | 3.808291303 | -0.548478667 | 0.252991093 | -2.167976195 | 0.030160498 | 0.109582487 | down |
| RDH8 | 1.952598711 | -0.55281697 | 0.255361669 | -2.164839276 | 0.030399996 | 0.110189276 | down |
| AQP4-AS1 | 0.542586218 | 0.594448345 | 0.274647855 | 2.164401921 | 0.030433517 | 0.110265108 | up |
| AC025271.1 | 2.44930505 | -0.674661792 | 0.311727868 | -2.16426525 | 0.030443998 | 0.110265108 | down |
| AL354861.2 | 0.769316533 | -0.59236618 | 0.273871408 | -2.162935461 | 0.030546144 | 0.110484025 | down |
| AC074019.1 | 1.142423808 | -0.724352728 | 0.335056287 | -2.161883708 | 0.030627141 | 0.110712596 | down |
| LRRC3B | 0.71545712 | -0.57267532 | 0.265065422 | -2.160505565 | 0.030733553 | 0.110993623 | down |
| AC093523.1 | 0.917821643 | -0.861939402 | 0.398998804 | -2.160255601 | 0.030752888 | 0.111034892 | down |
| GRID2 | 1.050267841 | 0.770378287 | 0.356786823 | 2.15921171 | 0.030833746 | 0.111239956 | up |
| ENPP7 | 3.409018384 | 0.512236371 | 0.237249786 | 2.159059357 | 0.030845562 | 0.111261617 | up |
| RNU6-6P | 0.355327 | -0.7725349 | 0.357865 | -2.158733 | 0.03087089 | NA | down |
| AC008627.1 | 0.3239366 | -1.097066 | 0.5083568 | -2.158063 | 0.03092296 | NA | down |
| AC079602.2 | 0.508346777 | -0.929956363 | 0.43099411 | -2.157700862 | 0.030951097 | 0.111529996 | down |
| AC005186.1 | 0.453608047 | 0.624650852 | 0.289553694 | 2.157288491 | 0.030983193 | 0.111565518 | up |
| AL024507.1 | 0.798134993 | -0.733683793 | 0.34046253 | -2.154961937 | 0.031164813 | 0.112062635 | down |
| RNA5SP301 | 0.3345738 | -0.7837975 | 0.3637464 | -2.154791 | 0.03117819 | NA | down |
| AC068491.3 | 0.950686533 | -0.564186684 | 0.261925916 | -2.153993357 | 0.031240693 | 0.112271931 | down |
| AC087257.2 | 0.932944925 | -0.560459358 | 0.260199294 | -2.15396187 | 0.031243162 | 0.112271931 | down |
| RN7SL395P | 0.786198158 | -0.529119326 | 0.245801835 | -2.152625614 | 0.031348114 | 0.112547501 | down |
| PMP2 | 1.914027683 | 0.581197635 | 0.270087646 | 2.151885299 | 0.031406389 | 0.112688956 | up |
| OR5M11 | 0.996323455 | 0.541998107 | 0.25190274 | 2.151616557 | 0.031427567 | 0.112752845 | up |
| AF127577.6 | 1.889616824 | -0.524421087 | 0.243844611 | -2.150636367 | 0.031504913 | 0.112945517 | down |
| GAPDHP29 | 2.88747106 | -0.672407589 | 0.312730807 | -2.150116244 | 0.031546021 | 0.113028062 | down |
| MUC21 | 1.602249065 | 0.708055218 | 0.329560434 | 2.148483693 | 0.03167535 | 0.113337834 | up |
| TBC1D26 | 0.3951483 | 0.6341928 | 0.2952125 | 2.148258 | 0.03169325 | NA | up |
| AP001207.1 | 0.527944676 | -0.694293771 | 0.323356627 | -2.147145638 | 0.031781689 | 0.113612414 | down |
| AF127577.3 | 13.08250285 | -0.609527615 | 0.283981429 | -2.146364346 | 0.031843922 | 0.11371265 | down |
| DDX53 | 1.170492046 | 1.560120019 | 0.726984985 | 2.146014087 | 0.031871855 | 0.113797427 | up |
| AC073651.1 | 0.684361689 | -0.662146961 | 0.308571656 | -2.145845054 | 0.031885343 | 0.113809131 | down |
| SULT1C2P2 | 0.641592813 | 0.565999404 | 0.263780402 | 2.145721972 | 0.031895167 | 0.113832047 | up |
| AC100771.1 | 3.347371103 | -0.523443883 | 0.243994142 | -2.14531332 | 0.031927804 | 0.113894375 | down |
| AC234781.2 | 0.608428648 | -0.503212737 | 0.234589639 | -2.145076566 | 0.031946726 | 0.113930943 | down |
| AC084026.3 | 0.442672768 | 0.763728101 | 0.356173168 | 2.14426063 | 0.032012009 | 0.114090761 | up |
| IL1RAPL2 | 1.190878711 | 0.867457499 | 0.404846909 | 2.142680309 | 0.032138778 | 0.1144328 | up |
| AL499627.1 | 1.220783936 | 0.848652135 | 0.396184988 | 2.142060301 | 0.03218863 | 0.114573967 | up |
| VTRNA1-1 | 1.074962873 | -0.95377701 | 0.445383929 | -2.141471543 | 0.032236031 | 0.114681395 | down |
| CR392039.2 | 0.932592724 | 0.713723688 | 0.333632616 | 2.139250341 | 0.032415399 | 0.115086884 | up |
| AC106795.3 | 0.458531746 | 0.613351003 | 0.286731844 | 2.139110171 | 0.032426747 | 0.115102732 | up |
| CHRM3-AS1 | 0.538613951 | -0.587135319 | 0.274538222 | -2.138628689 | 0.032465752 | 0.115186962 | down |
| UBE2CP3 | 0.569305541 | -0.741221748 | 0.346590324 | -2.138610624 | 0.032467216 | 0.115186962 | down |
| LINC00523 | 1.038066635 | 0.980738735 | 0.458870929 | 2.137286704 | 0.03257468 | 0.115431796 | up |
| AC022730.4 | 2.86464278 | -0.767115935 | 0.359182232 | -2.13572907 | 0.032701503 | 0.115672835 | down |
| AC012625.1 | 0.63434964 | 0.727095301 | 0.340497018 | 2.135394034 | 0.032728838 | 0.115741868 | up |
| EVPLL | 1.378872966 | 0.55500682 | 0.260012081 | 2.134542434 | 0.032798404 | 0.115917504 | up |
| ZNF969P | 0.618139759 | -0.55347248 | 0.259414785 | -2.133542544 | 0.032880246 | 0.11613311 | down |
| AL627223.1 | 0.600250004 | -0.609037739 | 0.285507358 | -2.133177035 | 0.032910206 | 0.116202113 | down |
| RPL18P13 | 27.97949662 | -0.530649158 | 0.248986671 | -2.131235202 | 0.03306977 | 0.116654661 | down |
| RPS16P5 | 0.5139145 | -0.808441104 | 0.379461988 | -2.130492988 | 0.033130934 | 0.11680881 | down |
| RNA5SP155 | 0.701474397 | -0.58012215 | 0.272368885 | -2.129913443 | 0.03317876 | 0.116958376 | down |
| SHMT1P1 | 0.613402405 | -0.582699374 | 0.273612019 | -2.129655616 | 0.033200056 | 0.11701464 | down |
| HIST1H2BPS2 | 0.461205248 | 0.594823947 | 0.27958826 | 2.127499729 | 0.033378584 | 0.117464627 | up |
| AC234772.3 | 1.27929398 | 0.569571746 | 0.267721243 | 2.127480586 | 0.033380173 | 0.117464627 | up |
| SNRPCP2 | 7.384961355 | -0.511675002 | 0.240533039 | -2.127254554 | 0.033398939 | 0.117487248 | down |
| MTCYBP3 | 0.459894138 | -0.632518396 | 0.29748408 | -2.126226032 | 0.033484444 | 0.117720067 | down |
| AL137141.1 | 0.648423041 | 0.611412468 | 0.287692411 | 2.125229742 | 0.033567447 | 0.117900327 | up |
| RN7SL660P | 0.3039976 | -1.254421 | 0.5903125 | -2.125012 | 0.03358559 | NA | down |
| LRRC14B | 2.027390672 | -0.55831631 | 0.262781858 | -2.124637956 | 0.033616834 | 0.118037455 | down |
| AC069431.2 | 0.543615408 | -0.860607529 | 0.405447486 | -2.12261158 | 0.033786414 | 0.118465992 | down |
| AC009088.2 | 2.529844332 | -0.542412304 | 0.25554692 | -2.122554652 | 0.033791188 | 0.118465992 | down |
| OR1J4 | 0.899008356 | -0.622290694 | 0.293187662 | -2.122499597 | 0.033795806 | 0.118465992 | down |
| AC009263.1 | 0.484572427 | -0.868609428 | 0.409260111 | -2.122389659 | 0.03380503 | 0.118485903 | down |
| AC005587.1 | 0.923997066 | 0.565279704 | 0.266363128 | 2.12221454 | 0.033819726 | 0.118512571 | up |
| LINC00379 | 0.434261603 | -0.686666099 | 0.323572747 | -2.12213824 | 0.033826131 | 0.118522595 | down |
| AL512844.2 | 0.449220468 | -0.528756593 | 0.249191835 | -2.12188571 | 0.033847337 | 0.118547213 | down |
| C7orf33 | 0.769110298 | 1.116598766 | 0.526313152 | 2.121548285 | 0.033875689 | 0.118621667 | up |
| AC080188.2 | 0.690426002 | -0.59274705 | 0.279490348 | -2.120814028 | 0.033937456 | 0.118800634 | down |
| OR2T8 | 0.4292422 | 0.6514519 | 0.3071708 | 2.120813 | 0.03393752 | NA | up |
| PLA2G2E | 0.79265922 | 0.632650688 | 0.298315764 | 2.120741724 | 0.033943543 | 0.118801827 | up |
| AC092535.3 | 0.58847355 | -0.739791921 | 0.348839059 | -2.120725592 | 0.033944902 | 0.118801827 | down |
| MRGPRE | 1.184596946 | 0.589353653 | 0.278077662 | 2.119385097 | 0.034057935 | 0.118998162 | up |
| AC021146.12 | 1.319046514 | -1.28530928 | 0.606518008 | -2.119160954 | 0.034076867 | 0.119039434 | down |
| FTX_1 | 0.739654569 | -0.621269575 | 0.293223833 | -2.118755382 | 0.034111145 | 0.119096973 | down |
| ZNF37CP | 0.676724785 | 0.500043969 | 0.236056452 | 2.118323667 | 0.034147666 | 0.119174712 | up |
| IGKV7-3 | 6.993235358 | -0.518947409 | 0.24512892 | -2.117038698 | 0.034256564 | 0.119455031 | down |
| NRBF2P2 | 0.4065293 | 0.6405946 | 0.3025921 | 2.117024 | 0.03425784 | NA | up |
| AC025822.2 | 0.740598446 | 0.544133258 | 0.257104668 | 2.116388095 | 0.034311814 | 0.119585343 | up |
| AC138123.3 | 0.924975729 | -0.556022631 | 0.262730986 | -2.1163192 | 0.034317669 | 0.119593285 | down |
| AC096664.1 | 0.481864319 | -0.810414823 | 0.383133994 | -2.115225576 | 0.034410726 | 0.119755343 | down |
| AC245100.6 | 1.594757286 | -0.508951214 | 0.24070078 | -2.114456029 | 0.034476337 | 0.119908806 | down |
| APOB | 30.58341106 | -0.607676533 | 0.287491963 | -2.113716594 | 0.03453948 | 0.120073385 | down |
| MT-TH | 1.766019663 | -0.650130716 | 0.307624942 | -2.113387529 | 0.034567612 | 0.120151287 | down |
| TMPRSS15 | 1.630458495 | 0.850467761 | 0.403063978 | 2.110006867 | 0.034857764 | 0.121007619 | up |
| LINC02243 | 0.3894212 | -0.6706457 | 0.3179712 | -2.10914 | 0.03493249 | NA | down |
| LEP | 5.292540921 | -0.547238711 | 0.259570081 | -2.108250335 | 0.035009341 | 0.121358701 | down |
| AC055854.1 | 0.614813692 | 0.61654037 | 0.292715033 | 2.106281881 | 0.035179874 | 0.121816269 | up |
| FAM58DP | 1.013817924 | -0.554437142 | 0.263232478 | -2.106264192 | 0.03518141 | 0.121816269 | down |
| IGHV1OR15-6 | 0.589465463 | -0.86600719 | 0.411277562 | -2.105651437 | 0.035234641 | 0.121912223 | down |
| CALHM3 | 6.728005187 | 0.506757834 | 0.240759206 | 2.104832638 | 0.035305879 | 0.122057676 | up |
| AC122134.1 | 0.575111513 | -0.779491586 | 0.370483079 | -2.103987013 | 0.03537958 | 0.122236649 | down |
| RPL41P1 | 352.5243707 | 0.583427183 | 0.277486379 | 2.1025435 | 0.035505693 | 0.122545762 | up |
| AC099509.1 | 1.465231273 | 0.507322418 | 0.241375019 | 2.101801668 | 0.035570653 | 0.122710929 | up |
| AC087623.1 | 0.453794257 | 0.509596878 | 0.242704907 | 2.099656262 | 0.03575909 | 0.123153421 | up |
| CDH9 | 1.550358318 | -1.30402759 | 0.621120907 | -2.099474636 | 0.035775081 | 0.123195809 | down |
| AL356441.2 | 0.497151109 | 0.511535488 | 0.243672489 | 2.099274682 | 0.035792694 | 0.123204815 | up |
| AC016716.2 | 0.453670494 | -1.673625304 | 0.797608636 | -2.09830389 | 0.035878309 | 0.123425223 | down |
| MIR219A2 | 0.3752922 | -0.6951974 | 0.331823 | -2.095085 | 0.03616345 | NA | down |
| AP003097.1 | 2.330891663 | -0.508914878 | 0.243010194 | -2.094212059 | 0.036241094 | 0.124390683 | down |
| AC073324.2 | 0.682153368 | -0.561658152 | 0.268475419 | -2.092028216 | 0.036435992 | 0.124931406 | down |
| AL353795.1 | 0.588654502 | -0.765106114 | 0.365870724 | -2.091192499 | 0.036510811 | 0.125072531 | down |
| MYL6P3 | 9.543657632 | -0.547436407 | 0.26180205 | -2.091031778 | 0.036525215 | 0.125109058 | down |
| AC079035.1 | 0.824579792 | -0.611569892 | 0.292593525 | -2.090168922 | 0.036602628 | 0.125231488 | down |
| AL157931.1 | 0.579694519 | 0.774230916 | 0.370447034 | 2.089990865 | 0.03661862 | 0.125241753 | up |
| C5orf66-AS2 | 0.71991899 | -0.852072431 | 0.40811774 | -2.087810323 | 0.036814948 | 0.125702336 | down |
| MMP23A | 5.189234125 | 0.542580989 | 0.260047992 | 2.086464827 | 0.036936538 | 0.126014565 | up |
| BANF1P1 | 0.4305151 | -0.7221162 | 0.3461578 | -2.08609 | 0.03697048 | NA | down |
| LINC01603 | 0.531350919 | -0.510951108 | 0.244986674 | -2.08562817 | 0.037012318 | 0.126221042 | down |
| LINC02390 | 0.4035701 | 0.5578533 | 0.2675174 | 2.085297 | 0.03704237 | NA | up |
| AC237221.1 | 5.751612837 | 0.669797533 | 0.321351322 | 2.084315474 | 0.037131481 | 0.126498966 | up |
| DSCR8 | 0.504058704 | -1.289277296 | 0.618805265 | -2.083494386 | 0.037206183 | 0.126676029 | down |
| MIR642A | 0.43698612 | 0.553160327 | 0.265507149 | 2.083410294 | 0.037213841 | 0.126689204 | up |
| LINC01549 | 5.380373879 | -0.505801676 | 0.242982575 | -2.081637644 | 0.03737558 | 0.127007091 | down |
| FRMD6-AS2 | 0.650598926 | -0.695246138 | 0.334014762 | -2.081483267 | 0.037389694 | 0.127016331 | down |
| NUPR2 | 2.147848963 | -0.750673066 | 0.360676274 | -2.081293172 | 0.03740708 | 0.127020307 | down |
| AC109481.1 | 1.192903683 | -0.717608579 | 0.345105439 | -2.079389363 | 0.037581577 | 0.127383492 | down |
| AC006070.1 | 0.536421023 | -0.542564323 | 0.260951319 | -2.079178313 | 0.037600964 | 0.127423373 | down |
| AC098869.1 | 0.44139431 | -1.094903574 | 0.526897923 | -2.07801839 | 0.037707665 | 0.127686023 | down |
| AL138918.1 | 0.625546388 | 0.553929614 | 0.26657556 | 2.077945982 | 0.037714335 | 0.127686023 | up |
| AL450023.1 | 0.759118883 | -0.73778591 | 0.355059682 | -2.077920834 | 0.037716651 | 0.127686023 | down |
| CYP3A43 | 1.334189374 | -0.515466216 | 0.248276302 | -2.076179695 | 0.037877335 | 0.128113274 | down |
| GRM5 | 0.3799693 | -0.6094371 | 0.2936798 | -2.075176 | 0.03797026 | NA | down |
| HAND1 | 25.25573247 | -0.667724533 | 0.321821332 | -2.074829931 | 0.0380023 | 0.128401917 | down |
| AL160175.1 | 0.545171715 | -0.510039434 | 0.24582562 | -2.074801783 | 0.038004909 | 0.128401917 | down |
| MOG | 0.561434354 | 0.754248915 | 0.363577023 | 2.074523049 | 0.03803076 | 0.128411416 | up |
| NUS1P3 | 1.863647651 | -0.790003621 | 0.380900178 | -2.074043719 | 0.038075251 | 0.128535681 | down |
| LINC00403 | 1.758544446 | 0.821439855 | 0.396181803 | 2.073391178 | 0.038135889 | 0.1287144 | up |
| LINC01508 | 1.006058278 | 0.617243286 | 0.298614548 | 2.067023493 | 0.03873194 | 0.130182759 | up |
| FETUB | 1.365548288 | -0.749649588 | 0.362671459 | -2.067021181 | 0.038732158 | 0.130182759 | down |
| AC092427.1 | 0.3101364 | -1.106393 | 0.5354678 | -2.066218 | 0.03880791 | NA | down |
| AC023481.1 | 0.3112102 | -0.7504369 | 0.3633519 | -2.065317 | 0.038893 | NA | down |
| GLDCP1 | 1.085952499 | -0.7724318 | 0.374173456 | -2.064368244 | 0.038982815 | 0.130728472 | down |
| RPL7P46 | 0.892636134 | 0.62075861 | 0.300986987 | 2.062410126 | 0.039168706 | 0.131259715 | up |
| PRSS1 | 36.52868954 | -0.51695723 | 0.250858632 | -2.060751215 | 0.039326781 | 0.131697064 | down |
| IGHV1OR16-3 | 1.68260787 | -0.621405004 | 0.301574356 | -2.06053662 | 0.039347269 | 0.131712914 | down |
| AL358178.1 | 0.645361474 | -0.715550093 | 0.347365404 | -2.059934824 | 0.039404772 | 0.131839418 | down |
| LINC01413 | 1.14188789 | 0.763798079 | 0.370843059 | 2.059626196 | 0.03943429 | 0.131911783 | up |
| AC012354.2 | 9.182388807 | -0.529465523 | 0.257125191 | -2.059174057 | 0.039477567 | 0.132030136 | down |
| AL035405.1 | 1.116148097 | -0.618597361 | 0.300529377 | -2.058359041 | 0.039555681 | 0.13223848 | down |
| SNORD30 | 0.963394152 | -0.789047941 | 0.383428003 | -2.057877709 | 0.039601874 | 0.132313545 | down |
| IGKV3-25 | 0.4248466 | -0.935373 | 0.4549324 | -2.05607 | 0.03977574 | NA | down |
| AC026951.1 | 0.642460837 | -0.643715859 | 0.313172351 | -2.055468361 | 0.039833789 | 0.132862729 | down |
| AL513175.2 | 0.702948727 | -0.504067971 | 0.245439207 | -2.053738589 | 0.040001 | 0.133234405 | down |
| AC105118.1 | 0.983615561 | 0.551371036 | 0.268535699 | 2.053250416 | 0.040048297 | 0.133352096 | up |
| AC092801.1 | 0.930141293 | -0.767883688 | 0.373993306 | -2.053201691 | 0.040053021 | 0.133354546 | down |
| CSAG2 | 1.519602593 | 1.353394502 | 0.659469021 | 2.052248793 | 0.04014549 | 0.13359773 | up |
| BMP2KL | 0.3655375 | -0.7909478 | 0.3855202 | -2.051637 | 0.04020491 | NA | down |
| TMPOP1 | 0.4119992 | 0.5720875 | 0.2788966 | 2.051253 | 0.04024234 | NA | up |
| AC007552.1 | 0.614240422 | -0.647840297 | 0.315887687 | -2.05085644 | 0.040280929 | 0.133926681 | down |
| RN7SL272P | 3.786260655 | -0.528537929 | 0.257854062 | -2.049756068 | 0.040388241 | 0.134149986 | down |
| AHCYP3 | 0.533123783 | -0.815892294 | 0.398154518 | -2.049185071 | 0.040444021 | 0.134171783 | down |
| ANKRD63 | 0.765739588 | 0.680360902 | 0.332146025 | 2.048378879 | 0.040522889 | 0.13430386 | up |
| AL512649.1 | 0.928712614 | -0.589803158 | 0.288356492 | -2.0453958 | 0.040815853 | 0.135020569 | down |
| AC087893.1 | 0.929513777 | -0.52765911 | 0.258036143 | -2.04490388 | 0.040864335 | 0.135114123 | down |
| LINC02263 | 2.080888026 | -0.647403058 | 0.316627713 | -2.044682232 | 0.040886196 | 0.13517304 | down |
| TMED11P | 0.3749033 | -0.763625 | 0.373857 | -2.042559 | 0.04109609 | NA | down |
| AL390774.1 | 0.551501734 | -0.81451603 | 0.398940783 | -2.041696575 | 0.041181639 | 0.135967099 | down |
| SLC25A1P3 | 0.435631325 | -0.501932269 | 0.245963441 | -2.040678349 | 0.041282809 | 0.136120924 | down |
| VN1R48P | 0.999159326 | -0.57386932 | 0.281277165 | -2.040227192 | 0.041327703 | 0.136242084 | down |
| AL358176.3 | 5.264611874 | -0.516218677 | 0.253047855 | -2.040004162 | 0.041349911 | 0.136297594 | down |
| AK3P2 | 0.510060663 | -0.597751959 | 0.293369865 | -2.037537014 | 0.041596257 | 0.136951532 | down |
| AC133963.1 | 0.95954677 | 0.685101493 | 0.336254951 | 2.03744656 | 0.041605312 | 0.136951532 | up |
| RN7SL344P | 0.3671164 | -1.060947 | 0.5208589 | -2.036919 | 0.04165821 | NA | down |
| AC125603.1 | 3.328106653 | 0.609529617 | 0.299307372 | 2.036467104 | 0.041703473 | 0.137147757 | up |
| ASIC5 | 0.447620854 | 0.914405527 | 0.449415556 | 2.034654819 | 0.041885617 | 0.137552482 | up |
| AL450226.1 | 0.3398713 | -0.8207401 | 0.4035811 | -2.033643 | 0.04198758 | NA | down |
| LINC02433 | 0.555649175 | -0.814803528 | 0.400838377 | -2.032748295 | 0.042077959 | 0.138021485 | down |
| AC008953.1 | 1.458122423 | -0.515434689 | 0.253865942 | -2.030342016 | 0.042321786 | 0.138563036 | down |
| MZT1P1 | 0.4279083 | -0.8610735 | 0.4242948 | -2.029423 | 0.04241524 | NA | down |
| NDUFB4P11 | 0.727001006 | 0.550728529 | 0.271620471 | 2.027566357 | 0.042604526 | 0.139270571 | up |
| AL360091.2 | 0.350863 | -0.9489267 | 0.468347 | -2.026119 | 0.0427526 | NA | down |
| AL391839.1 | 0.3815411 | -0.7653234 | 0.3779844 | -2.024749 | 0.04289317 | NA | down |
| FGF20 | 26.99022068 | 0.617477346 | 0.30501292 | 2.024430133 | 0.042925914 | 0.139989548 | up |
| MIR4479 | 0.3861495 | -0.5821497 | 0.2875855 | -2.024266 | 0.04294274 | NA | down |
| AL136164.3 | 0.624296901 | -0.66742767 | 0.329910728 | -2.023055373 | 0.043067439 | 0.140303772 | down |
| OR2T10 | 0.527608248 | -1.19194766 | 0.589450913 | -2.02213218 | 0.043162698 | 0.140552026 | down |
| MEIS1-AS2 | 0.554216485 | -0.552924894 | 0.273444641 | -2.022072521 | 0.04316886 | 0.140552026 | down |
| MIR23C | 0.485980487 | -0.66835142 | 0.330570409 | -2.021812605 | 0.043195715 | 0.140584711 | down |
| AC008013.3 | 0.4044298 | 0.5832372 | 0.2884819 | 2.021746 | 0.04320256 | NA | up |
| AP000654.1 | 0.79645094 | -0.571518739 | 0.282731972 | -2.021415313 | 0.043236791 | 0.140677323 | down |
| AL162376.1 | 0.724156119 | 0.527607242 | 0.261054454 | 2.021062019 | 0.043273345 | 0.140741485 | up |
| AC018639.1 | 0.85693083 | -0.686329209 | 0.339604513 | -2.020966102 | 0.043283274 | 0.140760087 | down |
| SNORA63D | 0.4166082 | -1.266803 | 0.6271792 | -2.019842 | 0.04339977 | NA | down |
| COPS5P1 | 0.89056698 | -0.733372397 | 0.363138887 | -2.01953694 | 0.043431442 | 0.141118427 | down |
| NPSR1 | 202.2114717 | 0.632865314 | 0.313611126 | 2.01799382 | 0.043591905 | 0.141529795 | up |
| ITPKB-IT1 | 0.617993587 | -0.581546451 | 0.288224875 | -2.017683068 | 0.043624279 | 0.141552448 | down |
| IGKV1D-35 | 1.183810356 | -0.668314178 | 0.331559899 | -2.015666487 | 0.043834862 | 0.142070327 | down |
| COL18A1-AS1 | 0.849881267 | -0.567839518 | 0.281726927 | -2.015567076 | 0.043845265 | 0.142090273 | down |
| ADGRG4 | 0.752903033 | 0.966460152 | 0.479795176 | 2.014318191 | 0.043976137 | 0.142390202 | up |
| AC097721.1 | 0.3887848 | -0.8769004 | 0.4353813 | -2.014098 | 0.04399927 | NA | down |
| AC073310.1 | 0.615114513 | -0.580719163 | 0.288433256 | -2.013357168 | 0.044077068 | 0.142522432 | down |
| SUMO1P1 | 0.590135666 | -0.758095287 | 0.376595405 | -2.013023203 | 0.044112188 | 0.142568444 | down |
| LINC02412 | 0.653291066 | -0.552669721 | 0.274616362 | -2.01251563 | 0.044165611 | 0.142713517 | down |
| AP003306.2 | 0.518874602 | 0.597994231 | 0.297233318 | 2.0118681 | 0.044233843 | 0.142851177 | up |
| AP002008.3 | 0.4230468 | 0.7541966 | 0.3751222 | 2.010536 | 0.04437453 | NA | up |
| AC013262.1 | 0.873813808 | 0.643200381 | 0.319992828 | 2.010046239 | 0.044426295 | 0.143402707 | up |
| GKN2 | 0.490315347 | 0.750399757 | 0.373331746 | 2.010007894 | 0.044430353 | 0.143402707 | up |
| AC087286.1 | 0.486215338 | -1.258782049 | 0.62637856 | -2.009618671 | 0.044471564 | 0.143508015 | down |
| AC016542.2 | 0.597029728 | -0.799729583 | 0.398209779 | -2.008312265 | 0.044610119 | 0.143802487 | down |
| LINC00319 | 0.4172353 | 0.5305323 | 0.2641722 | 2.008282 | 0.0446133 | NA | up |
| MYCBP2-AS2 | 0.3869565 | -0.6088224 | 0.3036059 | -2.005305 | 0.0449305 | NA | down |
| AL645568.2 | 0.3681617 | -0.6937594 | 0.3460796 | -2.004624 | 0.04500326 | NA | down |
| AC245517.5 | 0.91655324 | -0.613489088 | 0.306120308 | -2.004078373 | 0.045061666 | 0.144880989 | down |
| SCGB1A1 | 0.696868908 | 0.867272341 | 0.432787293 | 2.00392284 | 0.045078326 | 0.144889615 | up |
| AC100791.1 | 0.2993291 | 0.6661181 | 0.332707 | 2.002116 | 0.04527221 | NA | up |
| FAM133A | 1.341448743 | -0.533941934 | 0.267317904 | -1.997404314 | 0.04578128 | 0.146596053 | down |
| PRELID2P1 | 0.436194237 | -0.510227247 | 0.255929132 | -1.993627073 | 0.046192824 | 0.147604608 | down |
| KRT17P4 | 0.703438105 | 0.545774316 | 0.273818978 | 1.993193895 | 0.046240219 | 0.147690454 | up |
| PCDH11X | 0.60827616 | 0.656616795 | 0.32958567 | 1.992249226 | 0.04634372 | 0.147922146 | up |
| OR1J1 | 0.729499004 | -0.645500874 | 0.324091924 | -1.991721563 | 0.046401616 | 0.148072178 | down |
| AC127455.1 | 0.3306992 | -0.5904261 | 0.2964486 | -1.991664 | 0.04640792 | NA | down |
| AC092135.1 | 1.053756433 | -0.645868701 | 0.324739197 | -1.988884331 | 0.046713972 | 0.148791497 | down |
| LINC01896 | 0.403841 | -1.816618 | 0.9137547 | -1.988081 | 0.04680273 | NA | down |
| CYP4F26P | 3.060133238 | -0.643385965 | 0.323732653 | -1.987399045 | 0.046878193 | 0.149159555 | down |
| LINC02466 | 0.699867047 | -0.959385763 | 0.483111335 | -1.985848176 | 0.047050185 | 0.149591674 | down |
| DEFB126 | 0.855946763 | -1.328371516 | 0.669217106 | -1.984963482 | 0.047148535 | 0.149784284 | down |
| AC034223.1 | 0.449190177 | -0.695721712 | 0.35050001 | -1.984940634 | 0.047151077 | 0.149784284 | down |
| MT-TI | 1.123055871 | 0.587688035 | 0.296102269 | 1.984746814 | 0.047172648 | 0.149810356 | up |
| TPM3P4 | 0.713107226 | -0.566740192 | 0.285553741 | -1.984705887 | 0.047177203 | 0.149810356 | down |
| VWDE | 24.8058604 | -0.51307442 | 0.258535428 | -1.984542019 | 0.047195449 | 0.149844413 | down |
| OR6M1 | 0.4230333 | -1.12471 | 0.5674041 | -1.982203 | 0.04745647 | NA | down |
| MIR33B | 0.447832004 | 0.539691378 | 0.272308488 | 1.981911698 | 0.047489124 | 0.150557813 | up |
| AP001790.1 | 0.457340387 | 0.517982031 | 0.261460008 | 1.981113805 | 0.047578512 | 0.150741169 | up |
| NDUFB4P2 | 0.455370562 | -0.843407088 | 0.425866006 | -1.980451776 | 0.047652787 | 0.150890716 | down |
| SUMO2P8 | 0.606789408 | -0.757047125 | 0.38262568 | -1.978558065 | 0.047865785 | 0.151378827 | down |
| AC026798.1 | 0.98984242 | -0.616079134 | 0.311834121 | -1.975662999 | 0.048192958 | 0.152226383 | down |
| LY6D | 32.96363366 | 0.506730111 | 0.256645408 | 1.974436694 | 0.048332109 | 0.152622669 | up |
| AC007298.1 | 0.855051673 | -0.545911086 | 0.276615545 | -1.973537264 | 0.048434383 | 0.152844599 | down |
| RPL22P18 | 0.885300231 | -0.649323238 | 0.329082996 | -1.973129108 | 0.048480855 | 0.15296238 | down |
| ACP7 | 2.545839945 | -0.545170124 | 0.276387616 | -1.972483901 | 0.048554392 | 0.153131358 | down |
| C6orf118 | 0.843893921 | 0.908760415 | 0.460769182 | 1.972268221 | 0.048578995 | 0.153170864 | up |
| AC026124.1 | 0.574471067 | -0.850479656 | 0.431401766 | -1.971432948 | 0.048674375 | 0.153370375 | down |
| RPL35AP31 | 0.739212807 | -0.815194031 | 0.414023571 | -1.968955605 | 0.048958189 | 0.154104933 | down |
| AL357568.1 | 0.3935867 | -0.8919432 | 0.4531575 | -1.968285 | 0.04903526 | NA | down |
| AL132655.1 | 1.455340094 | -0.560050484 | 0.28458641 | -1.96794529 | 0.049074333 | 0.154412379 | down |
| Z98751.2 | 0.461163971 | -0.616941505 | 0.313693872 | -1.966699261 | 0.049217892 | 0.154631298 | down |
| AC010136.1 | 0.590145076 | -0.502040075 | 0.255321239 | -1.96630753 | 0.049263098 | 0.154688605 | down |
| CHCHD2P11 | 0.65370841 | -0.516856347 | 0.262888478 | -1.966066947 | 0.049290878 | 0.154729771 | down |
| AL161646.1 | 0.455394393 | 1.318068593 | 0.670472415 | 1.965880421 | 0.049312425 | 0.154782881 | up |
| TCEAL3-AS1 | 0.554259387 | -0.522268648 | 0.265850366 | -1.964521077 | 0.049469694 | 0.15515184 | down |
| AK6P1 | 1.351443101 | -0.824141405 | 0.419887245 | -1.962768374 | 0.049673094 | 0.155535388 | down |
| AC002543.1 | 0.921582045 | -0.548529647 | 0.279529947 | -1.962328735 | 0.049724224 | 0.155680907 | down |
| AP003393.1 | 1.019587811 | 0.5075211 | 0.258673235 | 1.962016284 | 0.049760588 | 0.155780176 | up |
| AC108059.1 | 0.3772898 | -0.8029357 | 0.4093344 | -1.961564 | 0.04981328 | NA | down |
| AC092652.1 | 0.3450711 | -0.8305611 | 0.4237069 | -1.960225 | 0.04996945 | NA | down |
