# Supplementary material for: Prognostic and Predictive Models for Left- and Right- Colorectal Cancer Patients: A Bioinformatics Analysis Based on Ferroptosis-Related Genes
Source: Front Oncol. 2022 Feb 21;12:833834. doi: 10.3389/fonc.2022.833834 (PMC8899601; doi:10.3389/fonc.2022.833834)
Supplement: Supplementary Table 2 — The choice criterion for the DEGs (high-risk group in LCRC/RCRC vs. low-risk group in LCRC/RCRC). P < 0.05 and |log2 fold change (FC)| > 1. [file Table_2.docx]

| Row.names | log2FoldChange | lfcSE | stat | pvalue |
| --- | --- | --- | --- | --- |
| CTXND1 | 2.847081 | 0.326994 | 8.706831 | 3.12E-18 |
| MUC6 | -2.29038 | 0.299402 | -7.64986 | 2.01E-14 |
| MTRNR2L8 | 1.719698 | 0.228356 | 7.530765 | 5.04E-14 |
| AL355075.4 | -3.52681 | 0.505802 | -6.9727 | 3.11E-12 |
| AC010970.1 | 2.627606 | 0.393695 | 6.674213 | 2.49E-11 |
| RNU4-2 | 2.016819 | 0.319038 | 6.321557 | 2.59E-10 |
| CTNNA2 | 1.932327 | 0.307676 | 6.280401 | 3.38E-10 |
| GDF10 | 2.023007 | 0.340443 | 5.942272 | 2.81E-09 |
| SNORA23 | -1.82441 | 0.327592 | -5.56916 | 2.56E-08 |
| ZNF556 | -1.89514 | 0.343973 | -5.50956 | 3.60E-08 |
| IGFBPL1 | 1.284687 | 0.242502 | 5.297638 | 1.17E-07 |
| FP671120.4 | 1.04443 | 0.198121 | 5.271682 | 1.35E-07 |
| PEG10 | -1.41656 | 0.278765 | -5.08154 | 3.74E-07 |
| REG3A | 1.943958 | 0.383953 | 5.063015 | 4.13E-07 |
| HLA-V | 1.473753 | 0.292063 | 5.046014 | 4.51E-07 |
| CLUL1 | 1.0882 | 0.216372 | 5.029292 | 4.92E-07 |
| SNORA73B | -1.24123 | 0.253797 | -4.89065 | 1.01E-06 |
| HIF3A | 1.023741 | 0.209639 | 4.883344 | 1.04E-06 |
| KIF19 | -1.08695 | 0.22404 | -4.85158 | 1.22E-06 |
| LINC00871 | -2.28505 | 0.478986 | -4.7706 | 1.84E-06 |
| GPC3 | 1.005653 | 0.215582 | 4.664828 | 3.09E-06 |
| AC245369.1 | -1.61363 | 0.346652 | -4.65489 | 3.24E-06 |
| CASKIN1 | 1.054134 | 0.227903 | 4.625372 | 3.74E-06 |
| EPHA8 | 1.906273 | 0.419707 | 4.541919 | 5.57E-06 |
| CCL25 | 1.700035 | 0.378094 | 4.496333 | 6.91E-06 |
| MIA | 1.22886 | 0.280401 | 4.382501 | 1.17E-05 |
| SNORA22 | -1.57075 | 0.374859 | -4.19025 | 2.79E-05 |
| TMEM114 | 1.363924 | 0.330491 | 4.126965 | 3.68E-05 |
| SLC14A1 | 1.20137 | 0.292143 | 4.112274 | 3.92E-05 |
| PRSS48 | -1.38912 | 0.338429 | -4.1046 | 4.05E-05 |
| FABP3 | 1.020131 | 0.251571 | 4.055043 | 5.01E-05 |
| UTS2R | 1.482086 | 0.365793 | 4.051702 | 5.08E-05 |
| SNORA54 | -1.82805 | 0.45192 | -4.04508 | 5.23E-05 |
| SERPINB7 | 1.224536 | 0.303741 | 4.031507 | 5.54E-05 |
| SNORA49 | -2.24571 | 0.55904 | -4.01708 | 5.89E-05 |
| ALOX12P2 | -1.32417 | 0.331364 | -3.99612 | 6.44E-05 |
| PAX3 | 1.924313 | 0.481903 | 3.993155 | 6.52E-05 |
| CDC20B | 1.420927 | 0.357277 | 3.977102 | 6.98E-05 |
| NHLH2 | -2.07816 | 0.522627 | -3.97638 | 7.00E-05 |
| RNU4-1 | 1.344806 | 0.34086 | 3.945337 | 7.97E-05 |
| HTR3A | 1.088194 | 0.277635 | 3.919512 | 8.87E-05 |
| NTRK2 | 1.110895 | 0.28371 | 3.915601 | 9.02E-05 |
| DWORF | 2.273179 | 0.581235 | 3.910944 | 9.19E-05 |
| SCNN1G | 1.145659 | 0.293192 | 3.907542 | 9.32E-05 |
| AL049839.2 | 1.392241 | 0.357387 | 3.895613 | 9.80E-05 |
| THRSP | -1.11113 | 0.287578 | -3.86374 | 0.000112 |
| LINC01198 | 1.781271 | 0.464384 | 3.835775 | 0.000125 |
| FGB | 1.2881 | 0.336207 | 3.831274 | 0.000127 |
| PHYHIPL | 1.186729 | 0.310187 | 3.825854 | 0.00013 |
| IGFL3 | -1.44615 | 0.378173 | -3.82404 | 0.000131 |
| L1CAM | 1.02472 | 0.267975 | 3.823937 | 0.000131 |
| NXPH4 | -1.0365 | 0.272385 | -3.80528 | 0.000142 |
| AC133041.1 | -1.23167 | 0.327137 | -3.76498 | 0.000167 |
| PRSS1 | 1.285105 | 0.341493 | 3.763199 | 0.000168 |
| NXPH1 | -1.21015 | 0.322697 | -3.75012 | 0.000177 |
| NUS1P3 | -1.97532 | 0.527751 | -3.7429 | 0.000182 |
| HIST1H2BB | -2.81097 | 0.753006 | -3.733 | 0.000189 |
| NTF4 | 1.24945 | 0.33538 | 3.725478 | 0.000195 |
| ADIPOQ | -2.48724 | 0.67303 | -3.69559 | 0.000219 |
| C5orf17 | -1.67435 | 0.457593 | -3.65903 | 0.000253 |
| LINC00524 | 1.232815 | 0.336932 | 3.658938 | 0.000253 |
| TMEM252 | 1.02019 | 0.279915 | 3.644644 | 0.000268 |
| CALB1 | -1.34326 | 0.373908 | -3.59248 | 0.000328 |
| AL353747.1 | 1.450629 | 0.405274 | 3.579379 | 0.000344 |
| AC108676.1 | -1.0283 | 0.287856 | -3.57226 | 0.000354 |
| HSBP1P2 | 1.661273 | 0.465103 | 3.571835 | 0.000354 |
| NKX6-3 | 1.775518 | 0.499333 | 3.555782 | 0.000377 |
| RPS2P45 | -1.15967 | 0.326443 | -3.55243 | 0.000382 |
| HBQ1 | 1.104257 | 0.313335 | 3.524208 | 0.000425 |
| LINC01516 | 1.426363 | 0.40494 | 3.522407 | 0.000428 |
| A2ML1 | -1.07686 | 0.306438 | -3.51413 | 0.000441 |
| MT3 | 1.052239 | 0.300077 | 3.506566 | 0.000454 |
| SNORA84 | -1.35208 | 0.387232 | -3.49166 | 0.00048 |
| GAPDHP55 | -1.29572 | 0.372735 | -3.47624 | 0.000508 |
| AC009779.4 | 1.295072 | 0.376103 | 3.443392 | 0.000574 |
| RNA5SP123 | -1.01965 | 0.297715 | -3.42491 | 0.000615 |
| RNU5E-1 | 1.321749 | 0.386241 | 3.422088 | 0.000621 |
| IGF2 | 1.256868 | 0.36729 | 3.422003 | 0.000622 |
| XAGE2 | 2.230729 | 0.652598 | 3.418228 | 0.00063 |
| VTCN1 | 1.276817 | 0.376739 | 3.389127 | 0.000701 |
| MTND4P24 | -1.08051 | 0.32229 | -3.35259 | 0.000801 |
| SETP4 | -1.05658 | 0.316266 | -3.34079 | 0.000835 |
| MAGEA1 | -2.32097 | 0.696728 | -3.33124 | 0.000865 |
| KRT42P | -1.07384 | 0.324051 | -3.31381 | 0.00092 |
| SPTSSB | 1.038551 | 0.314258 | 3.304769 | 0.000951 |
| CPN1 | 1.155878 | 0.350795 | 3.295021 | 0.000984 |
| XKR7 | 1.018913 | 0.31032 | 3.283431 | 0.001026 |
| C8orf34-AS1 | 1.728595 | 0.527495 | 3.276987 | 0.001049 |
| LINC02393 | -1.95305 | 0.597186 | -3.27043 | 0.001074 |
| TUSC5 | -1.57542 | 0.482868 | -3.26263 | 0.001104 |
| SERPINA7 | 1.221283 | 0.375817 | 3.249676 | 0.001155 |
| AC079062.1 | 1.792768 | 0.553472 | 3.239127 | 0.001199 |
| RNA5-8SP6 | 2.020168 | 0.624262 | 3.236091 | 0.001212 |
| C20orf85 | 1.341599 | 0.4157 | 3.227329 | 0.00125 |
| IGLV3-29 | -1.3496 | 0.420618 | -3.20861 | 0.001334 |
| KCNC2 | 1.531244 | 0.478117 | 3.202655 | 0.001362 |
| ITLN2 | 1.222865 | 0.382419 | 3.197711 | 0.001385 |
| CT83 | 2.98938 | 0.935562 | 3.195279 | 0.001397 |
| MORF4L1P3 | -1.01044 | 0.317022 | -3.18728 | 0.001436 |
| PRSS21 | -1.18838 | 0.375338 | -3.16617 | 0.001545 |
| SLC38A8 | 1.134159 | 0.359024 | 3.159006 | 0.001583 |
| HIST1H2BM | -1.33277 | 0.422535 | -3.15424 | 0.001609 |
| HMX2 | 1.129253 | 0.358219 | 3.152406 | 0.001619 |
| CCDC160 | 1.27051 | 0.403451 | 3.149106 | 0.001638 |
| GLP1R | 1.161634 | 0.369347 | 3.145107 | 0.00166 |
| SAA2-SAA4 | 1.147455 | 0.36679 | 3.128367 | 0.001758 |
| ERVV-2 | -1.5793 | 0.505769 | -3.12258 | 0.001793 |
| RNA5SP226 | 2.356551 | 0.755624 | 3.118681 | 0.001817 |
| TCP11 | 1.133881 | 0.36732 | 3.0869 | 0.002023 |
| PPBP | 1.048023 | 0.341404 | 3.069741 | 0.002142 |
| ITPK1-AS1 | -1.01303 | 0.330071 | -3.06914 | 0.002147 |
| AC092620.3 | -1.01514 | 0.33138 | -3.06338 | 0.002189 |
| SNORA7B | -1.14418 | 0.374641 | -3.05406 | 0.002258 |
| RPL7P46 | -1.28976 | 0.424415 | -3.03892 | 0.002374 |
| BHMT | -1.03868 | 0.341979 | -3.03726 | 0.002387 |
| RN7SL665P | -1.0766 | 0.354821 | -3.03419 | 0.002412 |
| CELA2A | -1.38931 | 0.458702 | -3.02879 | 0.002455 |
| IL36B | 1.447219 | 0.478673 | 3.023398 | 0.0025 |
| AC010735.1 | -1.01735 | 0.338578 | -3.00477 | 0.002658 |
| RNU1-88P | -2.99738 | 0.999644 | -2.99845 | 0.002714 |
| SNORA22C | -1.30761 | 0.436767 | -2.99383 | 0.002755 |
| MIR3609 | -1.65624 | 0.553676 | -2.99136 | 0.002777 |
| TGM6 | 2.132337 | 0.712857 | 2.991254 | 0.002778 |
| TTC29 | -1.64339 | 0.55292 | -2.9722 | 0.002957 |
| VGLL1 | 1.118956 | 0.376663 | 2.970711 | 0.002971 |
| AC008277.1 | -1.26131 | 0.427022 | -2.95373 | 0.00314 |
| SMILR | 1.103998 | 0.376567 | 2.931748 | 0.003371 |
| AC108136.1 | 1.026359 | 0.354173 | 2.897903 | 0.003757 |
| AL392089.1 | -1.05676 | 0.366293 | -2.88501 | 0.003914 |
| ZG16 | 1.050575 | 0.364905 | 2.879036 | 0.003989 |
| CST11 | -1.30509 | 0.455407 | -2.86576 | 0.00416 |
| FAM238B | 2.161163 | 0.755955 | 2.85885 | 0.004252 |
| RN7SKP203 | -2.54232 | 0.890425 | -2.85517 | 0.004301 |
| AC009271.1 | 1.112947 | 0.391335 | 2.843978 | 0.004455 |
| SOX21-AS1 | 1.271487 | 0.448801 | 2.833072 | 0.00461 |
| BPIFB1 | 1.330963 | 0.471518 | 2.822721 | 0.004762 |
| PCDHA11 | -1.02563 | 0.364281 | -2.8155 | 0.00487 |
| FOXI3 | 1.265066 | 0.450782 | 2.806381 | 0.00501 |
| SULT4A1 | 1.01813 | 0.363056 | 2.804334 | 0.005042 |
| ROPN1 | 1.103812 | 0.394461 | 2.798277 | 0.005138 |
| REG1B | 1.073412 | 0.38411 | 2.794546 | 0.005197 |
| CR392039.2 | 1.241563 | 0.445469 | 2.787093 | 0.005318 |
| RNA5-8SP2 | 2.001715 | 0.721398 | 2.774772 | 0.005524 |
| LINC02055 | -1.3666 | 0.493282 | -2.77043 | 0.005598 |
| LINC00566 | -1.01464 | 0.366967 | -2.76493 | 0.005694 |
| AL138759.1 | -1.01161 | 0.366017 | -2.76382 | 0.005713 |
| AC012506.3 | -1.95864 | 0.709961 | -2.7588 | 0.005801 |
| LCN8 | -1.45497 | 0.529913 | -2.74568 | 0.006039 |
| AC087783.2 | 1.228841 | 0.447955 | 2.743224 | 0.006084 |
| AC018797.1 | -1.03211 | 0.376297 | -2.7428 | 0.006092 |
| AC017104.3 | -1.05914 | 0.386811 | -2.73813 | 0.006179 |
| VSTM2B | 1.436304 | 0.525559 | 2.732907 | 0.006278 |
| CYP1A1 | 1.179591 | 0.433716 | 2.719732 | 0.006533 |
| LCE1E | 1.524493 | 0.56091 | 2.717892 | 0.00657 |
| AC011530.1 | -1.13626 | 0.419464 | -2.70883 | 0.006752 |
| CIDEA | -1.9248 | 0.710881 | -2.70762 | 0.006777 |
| RNVU1-19 | -1.00991 | 0.375632 | -2.68856 | 0.007176 |
| TEX15 | -1.31548 | 0.490241 | -2.68334 | 0.007289 |
| SNORA74B | -1.2932 | 0.48252 | -2.6801 | 0.00736 |
| AC009517.1 | 1.00451 | 0.375423 | 2.675673 | 0.007458 |
| AC069431.2 | -1.64603 | 0.619006 | -2.65915 | 0.007834 |
| AC015871.1 | 1.031757 | 0.388482 | 2.655869 | 0.00791 |
| AC106798.1 | -1.00198 | 0.377864 | -2.6517 | 0.008009 |
| AC020661.5 | -1.31232 | 0.496488 | -2.64321 | 0.008212 |
| IGLV3-30 | -1.33086 | 0.503997 | -2.64061 | 0.008276 |
| FOXN4 | 1.091989 | 0.416367 | 2.622661 | 0.008725 |
| MTCO1P42 | -1.0337 | 0.394297 | -2.62164 | 0.008751 |
| MAGEB2 | 2.083087 | 0.79458 | 2.62162 | 0.008751 |
| AC244157.2 | 1.216725 | 0.466015 | 2.610915 | 0.00903 |
| AC105137.1 | -1.09368 | 0.419782 | -2.60535 | 0.009178 |
| LINC01980 | 1.873783 | 0.719252 | 2.605184 | 0.009182 |
| DEFB126 | 2.281413 | 0.876023 | 2.604283 | 0.009207 |
| CPB1 | 1.021769 | 0.392644 | 2.602276 | 0.009261 |
| CD5L | -1.27581 | 0.494559 | -2.57969 | 0.009889 |
| ANXA10 | 1.114943 | 0.433916 | 2.569491 | 0.010185 |
| UGT1A3 | -1.00759 | 0.397434 | -2.53524 | 0.011237 |
| AC025884.2 | 1.833549 | 0.724561 | 2.530567 | 0.011388 |
| RPL31P50 | 1.008659 | 0.399682 | 2.523653 | 0.011614 |
| DNAJA1P5 | 1.546864 | 0.616606 | 2.508675 | 0.012118 |
| RNU6-1128P | -1.26812 | 0.506059 | -2.50588 | 0.012215 |
| AL390119.1 | 1.183151 | 0.47345 | 2.498996 | 0.012455 |
| RNU6-242P | -1.01741 | 0.407562 | -2.49633 | 0.012548 |
| SNORA37 | -1.22758 | 0.49275 | -2.49129 | 0.012728 |
| AC090559.2 | -1.2898 | 0.518191 | -2.48904 | 0.012809 |
| LINC01446 | 1.795005 | 0.72155 | 2.487708 | 0.012857 |
| AC092675.1 | -1.0483 | 0.423173 | -2.47724 | 0.01324 |
| ANKRD66 | 1.207661 | 0.488485 | 2.472259 | 0.013426 |
| AL627309.1 | 1.068492 | 0.432974 | 2.4678 | 0.013595 |
| PLA2G2E | -1.16237 | 0.471363 | -2.46598 | 0.013664 |
| RNU2-59P | -1.60784 | 0.656472 | -2.44922 | 0.014317 |
| SOHLH2 | 1.039085 | 0.424582 | 2.447313 | 0.014393 |
| AL365181.4 | 1.214846 | 0.497027 | 2.444224 | 0.014516 |
| AC090638.1 | -1.18055 | 0.484159 | -2.43834 | 0.014755 |
| AC108474.1 | 1.078941 | 0.442987 | 2.435607 | 0.014867 |
| CNTNAP4 | 1.190792 | 0.490487 | 2.427774 | 0.015192 |
| GFY | 1.004623 | 0.417884 | 2.404071 | 0.016214 |
| SFTPA1 | 1.106488 | 0.460717 | 2.401662 | 0.016321 |
| IGLV3-31 | -1.18504 | 0.494172 | -2.39803 | 0.016483 |
| AC004832.1 | 1.12233 | 0.468767 | 2.394215 | 0.016656 |
| HEMGN | 1.078828 | 0.453534 | 2.378716 | 0.017373 |
| XIAPP2 | -1.14826 | 0.483095 | -2.37689 | 0.017459 |
| ARMCX7P | -1.33551 | 0.562103 | -2.37592 | 0.017505 |
| FGG | 1.064342 | 0.449877 | 2.36585 | 0.017989 |
| RNA5SP145 | 1.641105 | 0.693668 | 2.365836 | 0.017989 |
| AC005229.1 | 1.019625 | 0.432127 | 2.359551 | 0.018297 |
| ALX3 | 1.805435 | 0.765237 | 2.359315 | 0.018309 |
| AP005117.1 | -1.03231 | 0.437748 | -2.35822 | 0.018363 |
| AC016691.1 | 1.048779 | 0.446489 | 2.348948 | 0.018827 |
| AF274855.1 | 1.945583 | 0.834512 | 2.331403 | 0.019732 |
| AP000812.3 | 1.041499 | 0.447353 | 2.328138 | 0.019905 |
| AC007552.1 | -1.01279 | 0.438652 | -2.30887 | 0.020951 |
| SFTPC | 1.148296 | 0.500091 | 2.296174 | 0.021666 |
| GTF3AP6 | 1.367776 | 0.59666 | 2.292389 | 0.021883 |
| RN7SL246P | -1.23802 | 0.542 | -2.28418 | 0.022361 |
| THSD4-AS1 | -1.13231 | 0.497282 | -2.27699 | 0.022787 |
| C14orf180 | -1.25899 | 0.553873 | -2.27307 | 0.023022 |
| AC091144.2 | -1.16355 | 0.512423 | -2.27068 | 0.023166 |
| RNA5SP298 | 1.023367 | 0.451089 | 2.268658 | 0.023289 |
| RN7SL359P | -1.6184 | 0.715748 | -2.26113 | 0.023751 |
| RN7SKP185 | 1.121021 | 0.496013 | 2.260062 | 0.023817 |
| SCARNA3 | -1.58056 | 0.702889 | -2.24866 | 0.024534 |
| MUC15 | -1.19338 | 0.530831 | -2.24814 | 0.024567 |
| AC103792.1 | -1.19903 | 0.533374 | -2.24801 | 0.024575 |
| LIN28B | -1.86797 | 0.831081 | -2.24764 | 0.024599 |
| SRMP2 | -1.08896 | 0.485798 | -2.24159 | 0.024988 |
| RN7SL338P | -1.06195 | 0.474022 | -2.24029 | 0.025072 |
| AC022639.1 | -1.2923 | 0.578414 | -2.23422 | 0.025469 |
| RN7SL166P | -1.32469 | 0.594352 | -2.2288 | 0.025827 |
| AC021744.1 | -1.17194 | 0.526923 | -2.22411 | 0.026141 |
| SALL3 | -1.5511 | 0.698645 | -2.22016 | 0.026408 |
| SLC5A8 | 1.162525 | 0.524748 | 2.215397 | 0.026733 |
| SNORA63D | -1.93059 | 0.875842 | -2.20427 | 0.027506 |
| AC104332.1 | 1.267778 | 0.577391 | 2.1957 | 0.028113 |
| AL122003.1 | -1.11143 | 0.509178 | -2.18279 | 0.029051 |
| IGHD3-22 | -1.03259 | 0.475288 | -2.17255 | 0.029814 |
| RNA5SP149 | 1.319508 | 0.613802 | 2.149729 | 0.031577 |
| AC008739.5 | -1.07513 | 0.504558 | -2.13083 | 0.033103 |
| SNORA11B | -1.39186 | 0.653823 | -2.12881 | 0.03327 |
| LINC01989 | -1.15302 | 0.547794 | -2.10485 | 0.035305 |
| PROK1 | -1.08841 | 0.520511 | -2.09105 | 0.036524 |
| AC091812.1 | 1.149878 | 0.550582 | 2.08848 | 0.036755 |
| LINC00523 | -1.33157 | 0.6381 | -2.08677 | 0.036909 |
| AL133480.1 | -1.18305 | 0.567209 | -2.08574 | 0.037002 |
| LINC02141 | -1.41348 | 0.67855 | -2.08308 | 0.037244 |
| IGLJ6 | -1.10205 | 0.531984 | -2.07158 | 0.038305 |
| CGB8 | 1.311598 | 0.635626 | 2.063475 | 0.039068 |
| AL499616.1 | -1.23679 | 0.605312 | -2.04322 | 0.04103 |
| AC133963.1 | -1.04578 | 0.513224 | -2.03767 | 0.041583 |
| ABCB10P1 | 1.094141 | 0.537569 | 2.035348 | 0.041816 |
| RPTN | -1.39424 | 0.689212 | -2.02295 | 0.043079 |
| AC011595.1 | -1.19197 | 0.589614 | -2.02162 | 0.043216 |
| RNU1-11P | -1.62077 | 0.803044 | -2.01828 | 0.043562 |
| AC026894.1 | -1.11724 | 0.555059 | -2.01284 | 0.044132 |
| IGLVI-20 | 1.037904 | 0.516855 | 2.008114 | 0.044631 |
| RNA5SP141 | 1.398172 | 0.697358 | 2.004955 | 0.044968 |
| AC016027.3 | -1.17255 | 0.58504 | -2.00422 | 0.045047 |
| AL162511.1 | 1.103865 | 0.55496 | 1.989089 | 0.046691 |
| AC073626.2 | 1.121473 | 0.564684 | 1.98602 | 0.047031 |
| RHEBP3 | -1.36374 | 0.688215 | -1.98156 | 0.047528 |
| CALML5 | -1.06229 | 0.536912 | -1.97851 | 0.047871 |
| SNORA63C | -1.14392 | 0.578196 | -1.97842 | 0.047881 |
| AC053481.3 | 1.27877 | 0.647452 | 1.97508 | 0.048259 |
| AC016251.1 | -1.18837 | 0.602982 | -1.97083 | 0.048744 |
