# Supplementary material for: Prognostic and Predictive Models for Left- and Right- Colorectal Cancer Patients: A Bioinformatics Analysis Based on Ferroptosis-Related Genes
Source: Front Oncol. 2022 Feb 21;12:833834. doi: 10.3389/fonc.2022.833834 (PMC8899601; doi:10.3389/fonc.2022.833834)
Supplement: Supplementary Table 3 — The choice criterion for the DEGs (high-risk group in LCRC/RCRC vs. low-risk group in LCRC/RCRC). P < 0.05 and |log2 fold change (FC)| > 1. [file Table_3.docx]

| Row.names | baseMean | log2FoldChange | lfcSE | stat | pvalue |
| --- | --- | --- | --- | --- | --- |
| MTND1P23 | 900.4588 | -3.12888 | 0.43322 | -7.22238 | 5.11E-13 |
| GPAT2 | 82.9061 | 1.422173 | 0.238384 | 5.965899 | 2.43E-09 |
| PRG4 | 18.59562 | 1.199493 | 0.204119 | 5.876441 | 4.19E-09 |
| PLIN4 | 144.7377 | 1.711101 | 0.291859 | 5.862757 | 4.55E-09 |
| PRSS56 | 45.58876 | -3.08656 | 0.540812 | -5.70727 | 1.15E-08 |
| SFTPA1 | 5.594449 | 3.29158 | 0.589292 | 5.585652 | 2.33E-08 |
| PRRT4 | 12.09071 | 1.749978 | 0.315273 | 5.550677 | 2.85E-08 |
| TMEM132C | 17.9839 | 1.807757 | 0.336313 | 5.375219 | 7.65E-08 |
| AKR1C2 | 145.5249 | 1.233993 | 0.229928 | 5.366866 | 8.01E-08 |
| MS4A1 | 133.3277 | 1.656919 | 0.313436 | 5.2863 | 1.25E-07 |
| OTOF | 16.78233 | 1.27215 | 0.243157 | 5.231796 | 1.68E-07 |
| AC110995.1 | 2.548459 | 1.21901 | 0.23445 | 5.199439 | 2.00E-07 |
| DPYSL5 | 7.252467 | 1.960682 | 0.380477 | 5.153219 | 2.56E-07 |
| IGF2 | 27482.99 | -2.15825 | 0.419682 | -5.14259 | 2.71E-07 |
| AQP5 | 351.0166 | 2.191011 | 0.431483 | 5.077862 | 3.82E-07 |
| BEST3 | 14.36176 | -1.59017 | 0.317921 | -5.00178 | 5.68E-07 |
| LTF | 215.0529 | 1.185706 | 0.237877 | 4.98454 | 6.21E-07 |
| AFP | 9.091027 | -1.72143 | 0.345634 | -4.9805 | 6.34E-07 |
| LY6D | 53.40441 | 1.993116 | 0.401564 | 4.96338 | 6.93E-07 |
| PCDH19 | 96.41185 | -1.40851 | 0.283805 | -4.96295 | 6.94E-07 |
| PCOLCE2 | 42.52812 | 1.495279 | 0.302029 | 4.950787 | 7.39E-07 |
| MT-TM | 23.71081 | -1.67014 | 0.339469 | -4.91987 | 8.66E-07 |
| ALB | 12.90982 | -2.28959 | 0.465831 | -4.91507 | 8.88E-07 |
| APOA2 | 13.22925 | -2.18314 | 0.445963 | -4.89534 | 9.81E-07 |
| SMC1B | 14.49688 | 1.240605 | 0.253706 | 4.889932 | 1.01E-06 |
| PTPRVP | 14.54587 | 1.174072 | 0.243469 | 4.822261 | 1.42E-06 |
| ATP1A3 | 126.3603 | 1.086155 | 0.225428 | 4.818186 | 1.45E-06 |
| C20orf197 | 2.132616 | 1.132136 | 0.235246 | 4.812552 | 1.49E-06 |
| LIN7A | 176.3136 | -1.25945 | 0.262095 | -4.80532 | 1.55E-06 |
| PANX2 | 23.96926 | 1.160326 | 0.241526 | 4.804141 | 1.55E-06 |
| MIA | 41.26493 | 1.563192 | 0.326999 | 4.780416 | 1.75E-06 |
| MAJIN | 2.813852 | 1.634776 | 0.342603 | 4.771634 | 1.83E-06 |
| LEP | 8.632401 | 1.956332 | 0.411245 | 4.75709 | 1.96E-06 |
| CA6 | 9.298526 | -2.2221 | 0.470718 | -4.72066 | 2.35E-06 |
| ABCA17P | 31.54744 | 1.558904 | 0.331208 | 4.706726 | 2.52E-06 |
| FCRL1 | 20.31716 | 1.585674 | 0.337965 | 4.691827 | 2.71E-06 |
| CYP1B1 | 234.8041 | 1.285855 | 0.274092 | 4.691322 | 2.71E-06 |
| COL2A1 | 26.71128 | -1.4636 | 0.312954 | -4.67672 | 2.92E-06 |
| GBX2 | 11.32438 | -1.66095 | 0.355324 | -4.67446 | 2.95E-06 |
| RRAD | 128.1419 | 1.02123 | 0.219715 | 4.647977 | 3.35E-06 |
| ORM2 | 8.901819 | -1.24007 | 0.266833 | -4.64736 | 3.36E-06 |
| CCDC160 | 3.645085 | 1.863661 | 0.401675 | 4.639725 | 3.49E-06 |
| SYNPR | 23.3716 | -1.86934 | 0.405028 | -4.61534 | 3.92E-06 |
| GRM1 | 3.07722 | 1.275753 | 0.276719 | 4.610284 | 4.02E-06 |
| OPRD1 | 54.01502 | -1.39757 | 0.304969 | -4.58267 | 4.59E-06 |
| FAM129C | 23.04753 | 1.203947 | 0.267723 | 4.49698 | 6.89E-06 |
| CD300LG | 6.294264 | 1.84113 | 0.410597 | 4.484032 | 7.32E-06 |
| TMEM151A | 32.81203 | 1.332487 | 0.298426 | 4.465056 | 8.00E-06 |
| PRSS1 | 35.11626 | -1.61505 | 0.362812 | -4.45149 | 8.53E-06 |
| MIR9-3HG | 10.46052 | 1.006372 | 0.226557 | 4.442031 | 8.91E-06 |
| CTNNA2 | 31.25056 | -1.55912 | 0.351054 | -4.44124 | 8.94E-06 |
| EBF2 | 28.72615 | 1.024843 | 0.23093 | 4.437902 | 9.08E-06 |
| GFAP | 11.43147 | 1.058758 | 0.239349 | 4.423483 | 9.71E-06 |
| RAB3C | 28.61882 | 1.313666 | 0.298749 | 4.397231 | 1.10E-05 |
| MYL12AP1 | 2.104108 | -1.65153 | 0.377135 | -4.37915 | 1.19E-05 |
| ADD2 | 23.73331 | 1.116595 | 0.255122 | 4.376717 | 1.20E-05 |
| BHMT2 | 27.99643 | 1.011086 | 0.231031 | 4.376407 | 1.21E-05 |
| CCL19 | 149.5485 | 1.295604 | 0.297606 | 4.353417 | 1.34E-05 |
| MTCYBP18 | 19.44332 | 1.055243 | 0.242762 | 4.346823 | 1.38E-05 |
| RIC3 | 11.99493 | 1.22566 | 0.282417 | 4.33989 | 1.43E-05 |
| MUC16 | 53.72069 | 1.658119 | 0.382476 | 4.335227 | 1.46E-05 |
| CYP7A1 | 1.157793 | 2.059176 | 0.47501 | 4.335013 | 1.46E-05 |
| LGSN | 8.514492 | -2.22229 | 0.516314 | -4.30415 | 1.68E-05 |
| CDH16 | 72.48564 | 1.659711 | 0.386944 | 4.289279 | 1.79E-05 |
| MEOX2 | 31.99361 | 1.146742 | 0.270211 | 4.243881 | 2.20E-05 |
| SPESP1 | 24.75781 | 1.267754 | 0.299035 | 4.239489 | 2.24E-05 |
| SLC26A9 | 44.07029 | 1.481051 | 0.349406 | 4.238762 | 2.25E-05 |
| AC016597.1 | 4.565341 | -1.09048 | 0.259452 | -4.203 | 2.63E-05 |
| MUC6 | 350.4206 | 1.615025 | 0.384761 | 4.197475 | 2.70E-05 |
| FCRLA | 38.87511 | 1.173135 | 0.279543 | 4.196626 | 2.71E-05 |
| TP63 | 16.23019 | 1.0418 | 0.248804 | 4.187232 | 2.82E-05 |
| NMNAT2 | 82.09557 | 1.064222 | 0.255496 | 4.165312 | 3.11E-05 |
| VWA5B2 | 41.45697 | -1.08005 | 0.260235 | -4.15028 | 3.32E-05 |
| AC022730.4 | 2.082619 | 1.726932 | 0.417472 | 4.136639 | 3.52E-05 |
| CDO1 | 18.859 | 1.128395 | 0.275303 | 4.098738 | 4.15E-05 |
| SUSD4 | 20.36365 | 1.030384 | 0.252004 | 4.088767 | 4.34E-05 |
| THBS4 | 207.3758 | 1.190751 | 0.291717 | 4.081874 | 4.47E-05 |
| ADCYAP1R1 | 12.11684 | 1.413399 | 0.347864 | 4.063077 | 4.84E-05 |
| MIR8071-1 | 1.644316 | 1.615555 | 0.398466 | 4.054439 | 5.03E-05 |
| BCORP1 | 2.778247 | 1.630409 | 0.402215 | 4.053577 | 5.04E-05 |
| SPTBN4 | 33.86781 | 1.00746 | 0.24871 | 4.050748 | 5.11E-05 |
| AL031595.2 | 2.856239 | 1.061683 | 0.263035 | 4.036276 | 5.43E-05 |
| NGFR | 108.207 | 1.027047 | 0.255937 | 4.012884 | 6.00E-05 |
| AL445437.1 | 1.389683 | 1.540376 | 0.384648 | 4.004637 | 6.21E-05 |
| TYR | 2.725325 | -2.53184 | 0.63399 | -3.9935 | 6.51E-05 |
| CCDC169 | 3.499609 | 1.427148 | 0.35949 | 3.969923 | 7.19E-05 |
| CNTN5 | 3.467788 | -1.84906 | 0.466099 | -3.9671 | 7.28E-05 |
| GAPDHP64 | 2.258385 | -1.14591 | 0.289273 | -3.96136 | 7.45E-05 |
| LINC02397 | 4.734709 | 1.210724 | 0.30717 | 3.941541 | 8.10E-05 |
| SLITRK2 | 4.387411 | 1.326686 | 0.337379 | 3.932333 | 8.41E-05 |
| RNY1 | 2.547004 | -2.3866 | 0.606949 | -3.93213 | 8.42E-05 |
| FAM69C | 16.03877 | -1.39817 | 0.355804 | -3.92961 | 8.51E-05 |
| VPREB3 | 27.17756 | 1.001181 | 0.255547 | 3.917799 | 8.94E-05 |
| AC093214.1 | 1.020392 | -1.60149 | 0.409736 | -3.90858 | 9.28E-05 |
| GIF | 80.71927 | -1.3319 | 0.340775 | -3.90844 | 9.29E-05 |
| AL136968.2 | 3.137376 | -1.02887 | 0.263319 | -3.90733 | 9.33E-05 |
| LINC02551 | 1.416318 | 1.173901 | 0.300905 | 3.901231 | 9.57E-05 |
| AF279873.1 | 4.685472 | -1.06073 | 0.273153 | -3.88329 | 0.000103 |
| TCF23 | 3.3511 | 1.227941 | 0.316695 | 3.877357 | 0.000106 |
| AHSG | 11.65168 | -1.52346 | 0.394273 | -3.86396 | 0.000112 |
| ACADL | 2.525916 | 1.670008 | 0.433078 | 3.856139 | 0.000115 |
| DTHD1 | 10.83906 | 1.03158 | 0.2679 | 3.850612 | 0.000118 |
| RGS7 | 7.382963 | 1.503588 | 0.390836 | 3.847113 | 0.00012 |
| MRAP | 1.712212 | 1.464902 | 0.381046 | 3.844426 | 0.000121 |
| MT-TL1 | 35.89707 | -1.08593 | 0.283272 | -3.83353 | 0.000126 |
| HNRNPA1P25 | 2.347798 | -1.0786 | 0.281588 | -3.83043 | 0.000128 |
| AC108725.1 | 1.304026 | -1.64353 | 0.429259 | -3.82878 | 0.000129 |
| AC017013.1 | 0.921237 | -1.44059 | 0.37662 | -3.82505 | 0.000131 |
| LMX1B | 10.33588 | 1.608238 | 0.422022 | 3.810791 | 0.000139 |
| ESPNP | 7.205835 | -1.03035 | 0.271127 | -3.80023 | 0.000145 |
| AC019080.3 | 0.948216 | 1.09234 | 0.287805 | 3.795424 | 0.000147 |
| CSAG3 | 5.263137 | -3.03546 | 0.801238 | -3.78846 | 0.000152 |
| IRX6 | 2.167486 | 2.030128 | 0.538261 | 3.771645 | 0.000162 |
| RPL3P12 | 6.304072 | -1.18403 | 0.31508 | -3.75788 | 0.000171 |
| DEFA5 | 1166.832 | -1.74244 | 0.464198 | -3.75367 | 0.000174 |
| SULT4A1 | 26.1527 | 1.54144 | 0.411271 | 3.747989 | 0.000178 |
| IGHE | 9.638007 | 1.169043 | 0.31197 | 3.747291 | 0.000179 |
| SLC10A2 | 1.993381 | -3.07405 | 0.821577 | -3.74164 | 0.000183 |
| KCNJ13 | 2.404826 | -1.46754 | 0.392929 | -3.73486 | 0.000188 |
| C7 | 373.0742 | 1.145243 | 0.307292 | 3.726891 | 0.000194 |
| BPIFB2 | 3.446025 | -2.05607 | 0.554027 | -3.71113 | 0.000206 |
| AC138409.1 | 9.433559 | 1.215617 | 0.328834 | 3.69675 | 0.000218 |
| KRT14 | 20.38968 | 1.78692 | 0.483835 | 3.693242 | 0.000221 |
| FAM83C | 5.83212 | 1.455665 | 0.394193 | 3.692777 | 0.000222 |
| TNPO1P1 | 1.225914 | -1.02386 | 0.278363 | -3.67816 | 0.000235 |
| DPP6 | 10.31221 | 1.121098 | 0.304811 | 3.678006 | 0.000235 |
| ABCA8 | 57.36717 | 1.074523 | 0.293428 | 3.661965 | 0.00025 |
| WT1-AS | 3.433786 | 1.725746 | 0.472204 | 3.654665 | 0.000258 |
| GAPDHP25 | 2.17459 | -1.07703 | 0.295047 | -3.65037 | 0.000262 |
| PAX5 | 139.381 | 1.055209 | 0.289238 | 3.648236 | 0.000264 |
| APOA1 | 23.6852 | -1.21204 | 0.332443 | -3.64586 | 0.000266 |
| NTNG1 | 5.895674 | 1.243388 | 0.3432 | 3.622924 | 0.000291 |
| CASP1P2 | 10.00027 | 1.11553 | 0.307964 | 3.622269 | 0.000292 |
| ANXA10 | 60.03485 | -1.59589 | 0.440666 | -3.62154 | 0.000293 |
| FMO2 | 29.29993 | 1.00565 | 0.278104 | 3.616099 | 0.000299 |
| NKX6-3 | 20.48025 | -2.14079 | 0.592724 | -3.61179 | 0.000304 |
| DDX25 | 2.817928 | 1.120138 | 0.310458 | 3.608021 | 0.000309 |
| LGALS12 | 8.089511 | 1.109196 | 0.307535 | 3.606729 | 0.00031 |
| NAT8L | 39.26322 | 1.012375 | 0.28075 | 3.605973 | 0.000311 |
| LINC01882 | 3.169401 | 1.489726 | 0.414763 | 3.59175 | 0.000328 |
| PRDX2P1 | 3.73937 | -1.44558 | 0.40288 | -3.58811 | 0.000333 |
| AC016831.6 | 1.317897 | -1.80423 | 0.503973 | -3.58002 | 0.000344 |
| RNU4-2 | 14.70165 | -1.21288 | 0.339136 | -3.57639 | 0.000348 |
| FAM180B | 1.343221 | 1.284556 | 0.359228 | 3.575874 | 0.000349 |
| AL353678.1 | 2.739201 | -1.12902 | 0.316352 | -3.56889 | 0.000359 |
| CXCR5 | 5.193595 | 1.216149 | 0.341108 | 3.565285 | 0.000363 |
| NANOGP1 | 0.768003 | -1.49738 | 0.420027 | -3.56496 | 0.000364 |
| LINC02544 | 3.658032 | 1.066754 | 0.299238 | 3.5649 | 0.000364 |
| MTND2P13 | 0.695843 | -2.00875 | 0.564496 | -3.55847 | 0.000373 |
| GABRR1 | 8.836651 | -1.68879 | 0.475229 | -3.55364 | 0.00038 |
| CLEC4GP1 | 1.654478 | 1.539355 | 0.433247 | 3.553064 | 0.000381 |
| FAM83A-AS1 | 3.795842 | 1.196101 | 0.337628 | 3.542654 | 0.000396 |
| CPA1 | 1.385044 | 1.797156 | 0.509866 | 3.524761 | 0.000424 |
| AL359265.2 | 1.338759 | -1.5836 | 0.45003 | -3.51888 | 0.000433 |
| SFTA2 | 105.3034 | 1.040502 | 0.295709 | 3.518671 | 0.000434 |
| IL21 | 0.716632 | 1.483378 | 0.42187 | 3.516198 | 0.000438 |
| LINC01485 | 17.11127 | 1.236569 | 0.352359 | 3.509404 | 0.000449 |
| CSMD1 | 5.396689 | 1.150348 | 0.327795 | 3.509351 | 0.000449 |
| C10orf90 | 2.156423 | 1.340662 | 0.383447 | 3.496343 | 0.000472 |
| CADM3 | 38.16407 | 1.095568 | 0.313598 | 3.493538 | 0.000477 |
| HMX3 | 9.7864 | -1.51106 | 0.432722 | -3.49199 | 0.000479 |
| CST4 | 20.12663 | 1.118243 | 0.321336 | 3.479982 | 0.000501 |
| PRELID3BP10 | 1.358191 | -1.2982 | 0.373183 | -3.47872 | 0.000504 |
| STON1-GTF2A1L | 1.616329 | 1.224404 | 0.353492 | 3.463742 | 0.000533 |
| ALDOAP1 | 7.184883 | -1.17939 | 0.340908 | -3.45956 | 0.000541 |
| SLC7A10 | 5.16881 | 1.207724 | 0.349259 | 3.457964 | 0.000544 |
| HSD3BP4 | 1.226731 | 1.468376 | 0.424931 | 3.455564 | 0.000549 |
| TNR | 7.082646 | 1.012935 | 0.293362 | 3.452845 | 0.000555 |
| AC025822.2 | 0.748795 | 1.209225 | 0.350223 | 3.452728 | 0.000555 |
| SFTPC | 2.617378 | 2.656247 | 0.771644 | 3.442322 | 0.000577 |
| EEF1A1P15 | 2.226087 | -1.03051 | 0.299755 | -3.43785 | 0.000586 |
| APOA4 | 3.74455 | -2.52503 | 0.735148 | -3.43472 | 0.000593 |
| ANXA8 | 5.043107 | 1.443785 | 0.421478 | 3.425533 | 0.000614 |
| HBG2 | 1.845368 | -1.19765 | 0.349715 | -3.42464 | 0.000616 |
| AC098936.1 | 1.605555 | 1.257709 | 0.368425 | 3.413742 | 0.000641 |
| CBLN4 | 4.7063 | 1.393358 | 0.408163 | 3.413731 | 0.000641 |
| G6PC | 1.720691 | -2.00573 | 0.591815 | -3.38912 | 0.000701 |
| FZD10 | 145.9003 | -1.1372 | 0.33588 | -3.38573 | 0.00071 |
| AC108751.5 | 11.21219 | -1.02653 | 0.303806 | -3.3789 | 0.000728 |
| CILP | 165.2799 | 1.078513 | 0.319314 | 3.377597 | 0.000731 |
| PRSS2 | 573.4387 | -1.37167 | 0.406879 | -3.37119 | 0.000748 |
| IGHV1OR16-1 | 3.017713 | 1.33455 | 0.39595 | 3.370502 | 0.00075 |
| INSM1 | 90.59912 | -1.21622 | 0.360952 | -3.36947 | 0.000753 |
| SNRPD2P1 | 3.138426 | -1.07271 | 0.318635 | -3.36658 | 0.000761 |
| ANKRD20A10P | 1.267952 | 1.498517 | 0.445341 | 3.364873 | 0.000766 |
| NRXN1 | 9.1824 | 1.164861 | 0.346348 | 3.363262 | 0.00077 |
| RPS24P6 | 1.649762 | -1.10288 | 0.327952 | -3.36294 | 0.000771 |
| AL583785.1 | 7.488112 | 1.00005 | 0.298052 | 3.355294 | 0.000793 |
| GALNTL6 | 36.27686 | 1.051419 | 0.314942 | 3.338451 | 0.000842 |
| AL390778.2 | 3.296632 | 1.616152 | 0.48464 | 3.334744 | 0.000854 |
| AL021155.1 | 1.232491 | -1.47995 | 0.44736 | -3.30818 | 0.000939 |
| GAPDHP21 | 8.302643 | -1.027 | 0.310607 | -3.30643 | 0.000945 |
| AC005329.1 | 1.665608 | 1.243662 | 0.376186 | 3.305976 | 0.000946 |
| GPAT2P1 | 1.812815 | 1.236458 | 0.37496 | 3.297574 | 0.000975 |
| AC010328.2 | 3.312294 | -1.04316 | 0.316421 | -3.29673 | 0.000978 |
| AC098935.2 | 0.880278 | 1.237877 | 0.376298 | 3.289621 | 0.001003 |
| AL121886.1 | 4.708893 | -1.05953 | 0.322426 | -3.28612 | 0.001016 |
| HRH3 | 1.6876 | 1.784257 | 0.543884 | 3.280587 | 0.001036 |
| GRIN2A | 6.10011 | 1.080788 | 0.329489 | 3.280194 | 0.001037 |
| MIR8071-2 | 3.26575 | 1.105809 | 0.337161 | 3.279768 | 0.001039 |
| P2RX6P | 3.235516 | -1.27541 | 0.389548 | -3.27408 | 0.00106 |
| FGB | 33.35361 | -1.28036 | 0.391255 | -3.27244 | 0.001066 |
| DEFA6 | 755.9185 | -1.43288 | 0.438083 | -3.2708 | 0.001072 |
| WNT1 | 2.369903 | 1.103956 | 0.33776 | 3.268463 | 0.001081 |
| PHBP7 | 1.77052 | -1.27283 | 0.389698 | -3.2662 | 0.00109 |
| AL162457.2 | 2.320377 | 1.365153 | 0.418217 | 3.264223 | 0.001098 |
| DYDC2 | 2.262315 | -1.37846 | 0.422544 | -3.26228 | 0.001105 |
| AC009852.2 | 0.871905 | -1.35343 | 0.416686 | -3.24809 | 0.001162 |
| AL161670.2 | 1.135704 | -1.01502 | 0.312832 | -3.24462 | 0.001176 |
| TRIM71 | 19.9298 | -1.45043 | 0.449445 | -3.22716 | 0.00125 |
| CGB5 | 2.846374 | 1.917555 | 0.595883 | 3.218007 | 0.001291 |
| AC079316.1 | 1.086176 | -1.81917 | 0.565496 | -3.21694 | 0.001296 |
| AF001548.1 | 3.115132 | 1.037175 | 0.322594 | 3.215113 | 0.001304 |
| LINC02422 | 1.591699 | 1.171155 | 0.364404 | 3.213896 | 0.001309 |
| ADH1B | 194.1283 | 1.15828 | 0.360653 | 3.211619 | 0.00132 |
| AC244196.4 | 1.02152 | 1.205866 | 0.376256 | 3.204904 | 0.001351 |
| RPL23AP83 | 0.94429 | -1.04936 | 0.327767 | -3.20155 | 0.001367 |
| VDAC1P7 | 0.895424 | -1.01739 | 0.317912 | -3.20021 | 0.001373 |
| RPLP0P7 | 0.816364 | -1.87979 | 0.588015 | -3.19684 | 0.001389 |
| RERGL | 4.945305 | 1.214816 | 0.380012 | 3.196778 | 0.00139 |
| IGDCC3 | 7.171218 | -1.29183 | 0.40434 | -3.1949 | 0.001399 |
| ERVV-2 | 2.447103 | -1.69271 | 0.530153 | -3.19286 | 0.001409 |
| FOXCUT | 0.838711 | 1.024393 | 0.320857 | 3.192673 | 0.00141 |
| AC018450.1 | 0.767923 | 1.045258 | 0.329003 | 3.177043 | 0.001488 |
| TESC-AS1 | 1.453809 | -1.11274 | 0.350495 | -3.17477 | 0.0015 |
| FDCSP | 79.76154 | 1.219662 | 0.384361 | 3.173224 | 0.001508 |
| RGR | 17.14186 | -1.53354 | 0.483355 | -3.17271 | 0.00151 |
| PADI3 | 81.79483 | 1.137113 | 0.359148 | 3.166144 | 0.001545 |
| TRBV10-2 | 1.654961 | 1.078528 | 0.342205 | 3.151704 | 0.001623 |
| CFL1P4 | 3.578096 | -1.06873 | 0.339516 | -3.1478 | 0.001645 |
| EEF1A1P36 | 1.578073 | -1.10546 | 0.351629 | -3.14383 | 0.001668 |
| LINC00567 | 1.112509 | 1.51464 | 0.482103 | 3.141733 | 0.00168 |
| AHCYP4 | 1.153452 | -1.47311 | 0.469028 | -3.14078 | 0.001685 |
| DDX3P3 | 1.017337 | -1.47251 | 0.469394 | -3.13705 | 0.001707 |
| DKK4 | 125.5273 | -1.05806 | 0.33742 | -3.13574 | 0.001714 |
| RPS11P7 | 3.872095 | -1.0473 | 0.334393 | -3.13193 | 0.001737 |
| AC140479.3 | 0.828195 | -1.20209 | 0.384466 | -3.12666 | 0.001768 |
| ATP5F1P6 | 1.96535 | -1.44747 | 0.463407 | -3.12355 | 0.001787 |
| AC015818.3 | 0.774526 | 1.758659 | 0.563314 | 3.121984 | 0.001796 |
| AC112487.1 | 1.735375 | 1.036135 | 0.332031 | 3.120594 | 0.001805 |
| AC034207.1 | 9.294721 | -1.14179 | 0.366609 | -3.11446 | 0.001843 |
| LRP2 | 7.694275 | 1.078866 | 0.346514 | 3.113485 | 0.001849 |
| AC015977.1 | 1.422833 | -1.25361 | 0.403236 | -3.10888 | 0.001878 |
| U62631.1 | 1.543517 | 1.334534 | 0.430066 | 3.103088 | 0.001915 |
| AC005858.1 | 13.66403 | 1.281887 | 0.41407 | 3.095825 | 0.001963 |
| AC022239.2 | 2.025571 | 1.264523 | 0.410081 | 3.083595 | 0.002045 |
| MT-TF | 16.8682 | -1.00014 | 0.3247 | -3.08021 | 0.002069 |
| AL357632.1 | 2.900513 | -1.09009 | 0.354037 | -3.07903 | 0.002077 |
| AC080100.1 | 1.252476 | 1.203268 | 0.391417 | 3.074131 | 0.002111 |
| PHBP11 | 3.164463 | -1.01042 | 0.328798 | -3.07306 | 0.002119 |
| NEUROG3 | 55.02864 | -1.27377 | 0.415141 | -3.06828 | 0.002153 |
| RPL7P33 | 2.341773 | -1.04683 | 0.341278 | -3.06739 | 0.002159 |
| AC008957.3 | 2.408387 | 1.084509 | 0.354254 | 3.061389 | 0.002203 |
| AC092810.1 | 0.964682 | -1.38236 | 0.451981 | -3.05844 | 0.002225 |
| ADIPOQ | 12.03324 | 2.101444 | 0.687517 | 3.056569 | 0.002239 |
| CALM1P1 | 0.966363 | -1.2114 | 0.396466 | -3.0555 | 0.002247 |
| AL512649.1 | 0.897621 | -1.52797 | 0.500575 | -3.05243 | 0.00227 |
| SOST | 1.400418 | -1.46154 | 0.479258 | -3.04958 | 0.002292 |
| AC104667.1 | 0.968606 | 1.344902 | 0.441876 | 3.043621 | 0.002337 |
| EIF4A1P6 | 1.454352 | -1.08304 | 0.356341 | -3.03934 | 0.002371 |
| AC126768.2 | 0.678501 | 1.478968 | 0.486617 | 3.039286 | 0.002371 |
| CLDN18 | 960.1026 | -1.48603 | 0.489532 | -3.03561 | 0.0024 |
| MAGEA4 | 5.593144 | -3.01013 | 0.992817 | -3.03191 | 0.00243 |
| CCDC194 | 2.986588 | 1.078889 | 0.35619 | 3.028972 | 0.002454 |
| AQP4 | 0.983623 | 1.323306 | 0.437074 | 3.027646 | 0.002465 |
| PHYHIPL | 47.77854 | 1.119482 | 0.370364 | 3.022652 | 0.002506 |
| PSMA2P3 | 2.055673 | -1.14351 | 0.378402 | -3.02195 | 0.002511 |
| TRDV1 | 6.343496 | 1.109997 | 0.368254 | 3.014213 | 0.002576 |
| AC109327.1 | 1.967756 | -1.01587 | 0.337127 | -3.0133 | 0.002584 |
| AC073592.7 | 0.483673 | 1.333435 | 0.442589 | 3.012807 | 0.002588 |
| SLC2A2 | 3.282401 | -1.1492 | 0.383156 | -2.9993 | 0.002706 |
| ITLN2 | 19.93564 | -1.16675 | 0.389722 | -2.99379 | 0.002755 |
| AC012512.1 | 11.0295 | -1.10875 | 0.373073 | -2.97193 | 0.002959 |
| TREML4 | 2.49472 | 1.225536 | 0.412785 | 2.968947 | 0.002988 |
| IGHV3-76 | 4.523334 | 1.024588 | 0.345555 | 2.965048 | 0.003026 |
| BRINP2 | 3.074477 | -1.07606 | 0.363088 | -2.96362 | 0.00304 |
| GATA5 | 10.31702 | 1.346307 | 0.45436 | 2.963085 | 0.003046 |
| AC073465.1 | 0.794931 | -1.55449 | 0.525072 | -2.96052 | 0.003071 |
| AL391807.1 | 1.076263 | 1.0212 | 0.345379 | 2.956748 | 0.003109 |
| IGHV2-70 | 107.135 | 1.035578 | 0.351702 | 2.944478 | 0.003235 |
| RPL18AP15 | 1.419832 | -1.07079 | 0.364865 | -2.93475 | 0.003338 |
| AL591846.1 | 129.7215 | -1.06662 | 0.364119 | -2.9293 | 0.003397 |
| OR7E102P | 1.074899 | 1.205488 | 0.412731 | 2.920757 | 0.003492 |
| MICD | 2.835962 | 1.028556 | 0.352183 | 2.920516 | 0.003495 |
| AC006120.1 | 1.297386 | -1.22493 | 0.420812 | -2.91087 | 0.003604 |
| SERPINA3 | 5.197166 | -1.01587 | 0.3498 | -2.90414 | 0.003683 |
| AC119428.2 | 0.975134 | 1.125265 | 0.387644 | 2.902827 | 0.003698 |
| GAPDHP37 | 1.353286 | -1.19944 | 0.413709 | -2.89923 | 0.003741 |
| TTR | 22.02676 | -1.0094 | 0.348264 | -2.89837 | 0.003751 |
| TBX5-AS1 | 0.487873 | 1.63796 | 0.566654 | 2.890582 | 0.003845 |
| AC053503.4 | 5.760025 | 1.323057 | 0.457909 | 2.889344 | 0.00386 |
| GDF10 | 10.1902 | 1.034601 | 0.358401 | 2.886714 | 0.003893 |
| AL365209.1 | 1.955744 | 1.098701 | 0.381161 | 2.882513 | 0.003945 |
| GPRC6A | 7.698206 | -1.2657 | 0.440995 | -2.87009 | 0.004104 |
| TRIM54 | 97.74797 | 1.141851 | 0.398034 | 2.86873 | 0.004121 |
| VTRNA1-1 | 0.848237 | -1.86716 | 0.652371 | -2.86211 | 0.004208 |
| MYOC | 5.079348 | 1.359883 | 0.475677 | 2.858836 | 0.004252 |
| LCE1E | 1.786815 | 1.50745 | 0.528537 | 2.85212 | 0.004343 |
| HULC | 31.39398 | -1.67058 | 0.586088 | -2.85038 | 0.004367 |
| AL158801.4 | 3.047849 | -1.21476 | 0.426198 | -2.85022 | 0.004369 |
| AC022239.3 | 1.174068 | 1.383253 | 0.485361 | 2.849944 | 0.004373 |
| AJ011931.1 | 1.540835 | 1.147955 | 0.40355 | 2.844639 | 0.004446 |
| AL354861.2 | 0.625283 | 1.024099 | 0.360047 | 2.84435 | 0.00445 |
| RNF7P1 | 0.836322 | -1.08426 | 0.382622 | -2.83377 | 0.0046 |
| AL109914.1 | 0.811333 | 1.020232 | 0.360388 | 2.83093 | 0.004641 |
| COPS8P2 | 2.426816 | -1.07367 | 0.379382 | -2.83005 | 0.004654 |
| MRPL48P1 | 0.672311 | -1.30254 | 0.460333 | -2.82956 | 0.004661 |
| AL512599.1 | 2.773685 | -1.25109 | 0.442275 | -2.82877 | 0.004673 |
| AC125238.2 | 2.082507 | -1.14942 | 0.40756 | -2.82025 | 0.004799 |
| AC116348.1 | 3.804702 | 1.024841 | 0.363814 | 2.816933 | 0.004848 |
| AC011374.1 | 1.13917 | 1.067422 | 0.379293 | 2.814239 | 0.004889 |
| FBN3 | 4.06653 | -1.06812 | 0.379791 | -2.81239 | 0.004917 |
| AC010457.1 | 0.518333 | 1.094489 | 0.389634 | 2.809019 | 0.004969 |
| KRT16P6 | 1.130586 | 1.845164 | 0.65764 | 2.805736 | 0.00502 |
| OBP2B | 9.348759 | -1.24064 | 0.4424 | -2.80433 | 0.005042 |
| AC009812.2 | 0.618471 | -1.64777 | 0.589149 | -2.79687 | 0.00516 |
| FAM183DP | 0.586803 | 1.317482 | 0.471757 | 2.792713 | 0.005227 |
| AL137786.1 | 1.10555 | 1.000561 | 0.358523 | 2.790786 | 0.005258 |
| GPR87 | 1.234101 | 1.154863 | 0.413964 | 2.789769 | 0.005275 |
| AC078991.1 | 18.39903 | -1.09008 | 0.39092 | -2.78849 | 0.005295 |
| AC009779.4 | 1.591803 | -1.04506 | 0.375041 | -2.78652 | 0.005328 |
| APOC3 | 1.377162 | -1.68957 | 0.60691 | -2.78388 | 0.005371 |
| AP002358.1 | 1.588287 | 1.166117 | 0.418897 | 2.783776 | 0.005373 |
| HBQ1 | 9.559136 | 1.214774 | 0.436793 | 2.78112 | 0.005417 |
| LINC01913 | 4.149433 | -1.20879 | 0.434865 | -2.77968 | 0.005441 |
| AC108215.1 | 2.171369 | 1.005178 | 0.362228 | 2.774988 | 0.00552 |
| AC245041.2 | 7.580968 | 1.008522 | 0.363872 | 2.771636 | 0.005578 |
| IGHJ1 | 15.78185 | 1.099265 | 0.397181 | 2.767671 | 0.005646 |
| GMCL1P1 | 0.706563 | -1.13403 | 0.410099 | -2.76525 | 0.005688 |
| EXOC1L | 1.423192 | 1.073278 | 0.388748 | 2.760855 | 0.005765 |
| LINC01629 | 0.658331 | 1.295835 | 0.469433 | 2.760426 | 0.005773 |
| CDK5P1 | 0.627713 | -1.59841 | 0.579068 | -2.76031 | 0.005775 |
| APOB | 26.94408 | -1.24622 | 0.451646 | -2.75929 | 0.005793 |
| AL357084.1 | 0.949227 | -1.07356 | 0.389779 | -2.75428 | 0.005882 |
| SAGE1 | 1.962942 | -1.97926 | 0.718704 | -2.75393 | 0.005888 |
| RNU5B-1 | 1.577735 | -2.07404 | 0.754435 | -2.74913 | 0.005975 |
| FP325317.1 | 0.791615 | 1.348657 | 0.49254 | 2.738166 | 0.006178 |
| TTTY14 | 10.67218 | 1.199028 | 0.437912 | 2.738058 | 0.00618 |
| BNC1 | 2.373509 | 1.172898 | 0.429789 | 2.729008 | 0.006353 |
| LINC01105 | 1.094398 | 1.272315 | 0.466691 | 2.726248 | 0.006406 |
| RPS15AP5 | 1.166071 | -1.07671 | 0.394994 | -2.72589 | 0.006413 |
| AC083967.1 | 2.938315 | 1.022241 | 0.375896 | 2.719479 | 0.006538 |
| PCSK1 | 861.1333 | -1.04658 | 0.386269 | -2.70946 | 0.006739 |
| AC099684.2 | 1.975509 | 1.203418 | 0.444243 | 2.70892 | 0.00675 |
| KIAA0408 | 0.923074 | 1.055032 | 0.389495 | 2.708714 | 0.006754 |
| AC133065.1 | 0.496636 | 1.230488 | 0.454342 | 2.708284 | 0.006763 |
| EIF4E2P2 | 1.241459 | -1.39841 | 0.517741 | -2.70097 | 0.006914 |
| RPL14P5 | 1.514268 | -1.06923 | 0.396184 | -2.69883 | 0.006958 |
| ELOBP1 | 2.551567 | -1.03351 | 0.383342 | -2.69605 | 0.007017 |
| MAGEA8 | 1.156968 | 1.054892 | 0.391631 | 2.693587 | 0.007069 |
| AC010327.3 | 1.386506 | 1.028402 | 0.381857 | 2.693162 | 0.007078 |
| AC131254.1 | 0.612923 | 1.060127 | 0.394227 | 2.689126 | 0.007164 |
| AC023421.1 | 14.45602 | -1.10733 | 0.412251 | -2.68605 | 0.00723 |
| FAM204BP | 0.983938 | -1.11952 | 0.416807 | -2.68595 | 0.007232 |
| DGKK | 2.818578 | 1.069014 | 0.398855 | 2.680208 | 0.007358 |
| UGT2B27P | 0.635392 | -1.51924 | 0.567763 | -2.67583 | 0.007454 |
| AC015656.1 | 0.894144 | 1.622441 | 0.606537 | 2.674925 | 0.007475 |
| EIF3KP2 | 2.289378 | -1.22389 | 0.45773 | -2.67382 | 0.007499 |
| RPL30P3 | 1.032216 | -1.43171 | 0.536137 | -2.67042 | 0.007576 |
| AC110588.1 | 0.651292 | -1.44035 | 0.540096 | -2.66684 | 0.007657 |
| TCEAL2 | 12.37671 | 1.079112 | 0.40474 | 2.666182 | 0.007672 |
| CCNYL2 | 0.838594 | -1.27923 | 0.479891 | -2.66567 | 0.007683 |
| ATP5BP1 | 2.566119 | -1.21579 | 0.456311 | -2.6644 | 0.007713 |
| RPS3P1 | 0.768829 | -1.23364 | 0.463396 | -2.66218 | 0.007764 |
| LINC01170 | 2.30582 | 1.038157 | 0.390251 | 2.660229 | 0.007809 |
| AC108075.1 | 0.660469 | -1.2237 | 0.460253 | -2.65876 | 0.007843 |
| AP000902.1 | 3.984614 | 1.08872 | 0.409739 | 2.657106 | 0.007881 |
| AP005403.1 | 0.698936 | -1.36657 | 0.514664 | -2.65527 | 0.007924 |
| AC021146.11 | 0.625926 | 1.381711 | 0.521179 | 2.651126 | 0.008022 |
| PAX3 | 3.349099 | -1.55481 | 0.586496 | -2.65102 | 0.008025 |
| SLC28A2 | 504.1582 | 1.030862 | 0.389283 | 2.648104 | 0.008094 |
| AL591926.3 | 0.525701 | 1.203299 | 0.45513 | 2.643858 | 0.008197 |
| AL353705.2 | 0.901629 | -1.05044 | 0.397615 | -2.64186 | 0.008245 |
| NAA11 | 0.907145 | -1.221 | 0.462657 | -2.6391 | 0.008313 |
| FGG | 5.051365 | -1.3808 | 0.523267 | -2.63881 | 0.00832 |
| AC079062.1 | 3.332206 | 1.445695 | 0.54802 | 2.638034 | 0.008339 |
| FMO6P | 0.988296 | -1.29195 | 0.491151 | -2.63046 | 0.008527 |
| AC004835.1 | 0.834148 | -1.2565 | 0.478208 | -2.62752 | 0.008601 |
| AC120498.2 | 1.358713 | 1.049822 | 0.399769 | 2.626074 | 0.008638 |
| UPK1A-AS1 | 1.227444 | 1.082328 | 0.412581 | 2.623312 | 0.008708 |
| SLC5A8 | 8.111897 | 1.421478 | 0.54442 | 2.610995 | 0.009028 |
| MTND2P29 | 0.769135 | -1.40973 | 0.540201 | -2.60963 | 0.009064 |
| AC006058.4 | 2.036114 | 1.108088 | 0.425433 | 2.604612 | 0.009198 |
| AC078867.1 | 0.744221 | -1.38577 | 0.532416 | -2.6028 | 0.009247 |
| C1orf87 | 1.032392 | 1.150984 | 0.442784 | 2.599425 | 0.009338 |
| TCAM1P | 13.92484 | -1.27426 | 0.490281 | -2.59904 | 0.009349 |
| SEPT2P1 | 1.782049 | -1.04085 | 0.401155 | -2.59464 | 0.009469 |
| AL645927.1 | 1.34658 | 1.211264 | 0.468041 | 2.587945 | 0.009655 |
| GBX1 | 0.502266 | 1.04612 | 0.404523 | 2.586054 | 0.009708 |
| AC104651.1 | 1.841321 | -1.05227 | 0.407012 | -2.58536 | 0.009728 |
| PHBP18 | 0.986016 | -1.18279 | 0.457747 | -2.58393 | 0.009768 |
| SERPINA10 | 59.90654 | -1.04319 | 0.404242 | -2.5806 | 0.009863 |
| AC073862.5 | 0.989062 | 1.011433 | 0.39239 | 2.577624 | 0.009948 |
| TOMM22P5 | 1.230157 | -1.12754 | 0.437672 | -2.57622 | 0.009989 |
| MAGEA1 | 4.477806 | -2.28169 | 0.886693 | -2.57326 | 0.010074 |
| TTC29 | 7.241121 | 1.452202 | 0.564545 | 2.572343 | 0.010101 |
| AL138895.2 | 0.713548 | -1.18633 | 0.461407 | -2.57112 | 0.010137 |
| RPL23AP19 | 0.984623 | -1.06567 | 0.414534 | -2.57076 | 0.010148 |
| LAPTM4BP2 | 0.689908 | -1.28575 | 0.500904 | -2.56686 | 0.010262 |
| PITX3 | 0.88963 | 1.056414 | 0.413467 | 2.555014 | 0.010618 |
| AC109454.3 | 2.422615 | 1.495968 | 0.586821 | 2.549276 | 0.010795 |
| TEX26 | 0.461444 | 1.083603 | 0.425292 | 2.547905 | 0.010837 |
| AC107890.1 | 1.942395 | -1.12083 | 0.440404 | -2.545 | 0.010928 |
| ARL6IP1P3 | 0.884495 | -1.06875 | 0.421092 | -2.53804 | 0.011147 |
| KRTAP3-3 | 1.469148 | -1.06152 | 0.418354 | -2.53737 | 0.011169 |
| HSBP1P2 | 0.94171 | -1.34867 | 0.532832 | -2.53114 | 0.011369 |
| AC138466.4 | 1.073658 | 1.124709 | 0.444568 | 2.529893 | 0.01141 |
| AL121835.1 | 0.88656 | -1.48812 | 0.589058 | -2.52627 | 0.011528 |
| AC133561.1 | 2.214755 | -1.00991 | 0.400858 | -2.51936 | 0.011757 |
| AC063943.2 | 1.235228 | -1.17012 | 0.466232 | -2.50973 | 0.012082 |
| TUSC5 | 4.690909 | 1.189836 | 0.47573 | 2.501073 | 0.012382 |
| ANKRD34B | 2.418666 | 1.289501 | 0.518989 | 2.484638 | 0.012968 |
| AC108474.1 | 4.83632 | 1.212149 | 0.489033 | 2.478665 | 0.013188 |
| HOXC12 | 3.988141 | 1.406883 | 0.568017 | 2.476833 | 0.013255 |
| SH3GL3 | 1.682379 | 1.451391 | 0.587233 | 2.471576 | 0.013452 |
| AC011753.1 | 0.705022 | -1.4983 | 0.607335 | -2.46701 | 0.013625 |
| AC053503.6 | 1.820276 | 1.092595 | 0.444169 | 2.459861 | 0.013899 |
| C9orf170 | 0.692844 | 1.051181 | 0.427814 | 2.457095 | 0.014007 |
| LINC01098 | 1.17013 | -1.67141 | 0.684261 | -2.44265 | 0.01458 |
| MKRN7P | 0.666148 | 1.494848 | 0.61319 | 2.437823 | 0.014776 |
| PMP2 | 2.07416 | 1.04628 | 0.43017 | 2.432248 | 0.015005 |
| AC239600.2 | 0.950787 | -1.34355 | 0.552684 | -2.43096 | 0.015059 |
| LDHBP1 | 0.981043 | -1.0059 | 0.414123 | -2.42899 | 0.015141 |
| AC108451.2 | 1.515879 | -1.09412 | 0.450719 | -2.4275 | 0.015203 |
| GDI2P1 | 1.435735 | -1.00122 | 0.412632 | -2.42644 | 0.015248 |
| AC011601.1 | 0.66664 | 1.236293 | 0.510227 | 2.423026 | 0.015392 |
| C8orf17 | 0.90244 | 1.208669 | 0.499834 | 2.418143 | 0.0156 |
| IGLV3-13 | 3.023037 | -1.04081 | 0.430503 | -2.41767 | 0.01562 |
| AC007159.1 | 1.945577 | -1.20152 | 0.497731 | -2.41399 | 0.015779 |
| EN1 | 5.457101 | 1.165474 | 0.483098 | 2.412501 | 0.015844 |
| COX11P1 | 0.758403 | -1.14932 | 0.476615 | -2.41142 | 0.015891 |
| AC106875.1 | 1.482448 | -1.36995 | 0.568549 | -2.40956 | 0.015972 |
| RNU5E-1 | 2.513022 | -1.06887 | 0.443991 | -2.40741 | 0.016066 |
| AL135924.1 | 1.336722 | -1.15135 | 0.478338 | -2.40698 | 0.016085 |
| LL22NC03-63E9.3 | 0.793521 | -1.15547 | 0.480265 | -2.4059 | 0.016132 |
| KRT16P2 | 1.229503 | 1.122348 | 0.466774 | 2.404478 | 0.016196 |
| AC025524.2 | 0.415695 | -1.29598 | 0.540184 | -2.39915 | 0.016433 |
| PHB2P1 | 0.998123 | -1.72976 | 0.721597 | -2.39712 | 0.016524 |
| LIN28A | 2.095364 | -1.35419 | 0.565986 | -2.39262 | 0.016728 |
| FGF13-AS1 | 0.641042 | 1.118769 | 0.46764 | 2.392373 | 0.01674 |
| LINC02119 | 4.029153 | -1.25383 | 0.524373 | -2.39111 | 0.016798 |
| DEFB4A | 1.587095 | -1.49209 | 0.625071 | -2.38708 | 0.016983 |
| CRCT1 | 0.536465 | -1.25631 | 0.526568 | -2.38584 | 0.01704 |
| CLEC4M | 0.968509 | 1.270066 | 0.532491 | 2.385139 | 0.017073 |
| CGB8 | 0.984049 | 1.569344 | 0.658541 | 2.383063 | 0.017169 |
| RHAG | 0.988602 | -1.86214 | 0.781432 | -2.38299 | 0.017173 |
| TIMM17BP1 | 1.387197 | -1.14705 | 0.481502 | -2.38224 | 0.017208 |
| CPN1 | 16.46657 | -1.14496 | 0.48109 | -2.37993 | 0.017316 |
| RPL38P4 | 0.943579 | -1.13468 | 0.478392 | -2.37186 | 0.017699 |
| AC009387.1 | 0.51178 | 1.599971 | 0.676405 | 2.365405 | 0.01801 |
| RPL36P2 | 0.732577 | -1.05408 | 0.445996 | -2.36342 | 0.018107 |
| LCN15 | 966.9673 | -1.0671 | 0.451768 | -2.36204 | 0.018175 |
| AC127070.4 | 0.816696 | -1.15471 | 0.489051 | -2.36112 | 0.01822 |
| MTCYBP16 | 1.730286 | -1.0289 | 0.436021 | -2.35974 | 0.018288 |
| PRDX2P2 | 0.843788 | -1.28514 | 0.545098 | -2.35764 | 0.018392 |
| SP9 | 5.583081 | -1.61628 | 0.686056 | -2.3559 | 0.018478 |
| DSCAM-AS1 | 1.167983 | -2.95944 | 1.256674 | -2.35498 | 0.018524 |
| AC026316.1 | 5.853991 | -1.11062 | 0.472222 | -2.3519 | 0.018678 |
| MLN | 2.813647 | -1.52003 | 0.646516 | -2.35111 | 0.018718 |
| AC026798.1 | 0.910506 | -1.15685 | 0.493606 | -2.34367 | 0.019095 |
| TMPRSS15 | 1.53259 | -1.30423 | 0.557316 | -2.3402 | 0.019273 |
| AC133681.1 | 0.84038 | -1.53165 | 0.654849 | -2.33894 | 0.019338 |
| CYCSP51 | 1.935219 | -1.16081 | 0.498076 | -2.33059 | 0.019775 |
| SERPINB13 | 1.003872 | 2.244849 | 0.963428 | 2.330064 | 0.019803 |
| AC011754.1 | 3.435129 | -1.22026 | 0.524177 | -2.32795 | 0.019915 |
| KRT3 | 1.399569 | 1.245859 | 0.537814 | 2.316524 | 0.02053 |
| RNU5A-1 | 1.946063 | -1.29823 | 0.560496 | -2.31622 | 0.020546 |
| LINC00221 | 0.80095 | -1.67989 | 0.726035 | -2.31379 | 0.020679 |
| AC087783.2 | 1.179545 | -1.26103 | 0.548056 | -2.30092 | 0.021396 |
| TKTL1 | 15.63761 | -1.05906 | 0.462796 | -2.28839 | 0.022115 |
| LINC01468 | 1.830919 | 1.353409 | 0.592863 | 2.282836 | 0.02244 |
| AL110505.1 | 0.889396 | -1.83609 | 0.804961 | -2.28097 | 0.02255 |
| RACGAP1P | 0.934229 | -1.3059 | 0.572946 | -2.27928 | 0.022651 |
| PGPEP1L | 0.935334 | 1.211615 | 0.532023 | 2.277375 | 0.022764 |
| AC026904.1 | 0.746987 | 1.050271 | 0.461577 | 2.275398 | 0.022882 |
| EPHA8 | 3.975397 | 1.017811 | 0.447772 | 2.273056 | 0.023023 |
| LIN28B | 1.812071 | -1.9799 | 0.87178 | -2.2711 | 0.023141 |
| AC013444.1 | 0.535247 | 1.034207 | 0.456419 | 2.265916 | 0.023457 |
| IGHD3-3 | 2.467353 | 1.04364 | 0.460626 | 2.265699 | 0.02347 |
| SERTM1 | 0.948299 | 1.525333 | 0.673883 | 2.263497 | 0.023605 |
| AC034105.5 | 0.462192 | 1.068793 | 0.472336 | 2.262783 | 0.023649 |
| RNU6-33P | 0.403033 | -1.05394 | 0.466045 | -2.26145 | 0.023732 |
| AC008739.5 | 0.61484 | 1.172999 | 0.518825 | 2.260879 | 0.023767 |
| IGHEP1 | 0.952391 | 1.054221 | 0.466525 | 2.259732 | 0.023838 |
| LINC01991 | 0.591111 | 1.711198 | 0.757949 | 2.25767 | 0.023966 |
| AL160408.4 | 0.488263 | 1.479393 | 0.656588 | 2.253152 | 0.02425 |
| AC233982.1 | 0.613866 | -1.08686 | 0.483544 | -2.2477 | 0.024595 |
| AL591926.5 | 0.287916 | 1.121287 | 0.499867 | 2.243173 | 0.024886 |
| ASB5 | 1.741015 | 1.261712 | 0.56299 | 2.241089 | 0.02502 |
| MIR4420 | 0.565523 | 1.07522 | 0.48105 | 2.23515 | 0.025408 |
| LINC00626 | 0.648379 | 1.076223 | 0.482211 | 2.231851 | 0.025625 |
| AC004672.1 | 0.931327 | 1.079492 | 0.483742 | 2.231547 | 0.025645 |
| UBBP3 | 0.652462 | -1.03625 | 0.465195 | -2.22756 | 0.02591 |
| AC012174.1 | 0.662867 | -1.30799 | 0.589213 | -2.21989 | 0.026426 |
| PCDHB1 | 0.678069 | -1.0184 | 0.459375 | -2.21693 | 0.026628 |
| AP004550.1 | 0.67471 | 1.07117 | 0.483413 | 2.215849 | 0.026702 |
| RNU1-85P | 0.478816 | 1.30093 | 0.588155 | 2.211885 | 0.026975 |
| C6orf10 | 0.470175 | 1.170031 | 0.529549 | 2.209485 | 0.027141 |
| MAGEA9B | 0.798474 | -2.48935 | 1.128694 | -2.20552 | 0.027418 |
| PSAPL1 | 7.221214 | -1.19162 | 0.540475 | -2.20477 | 0.02747 |
| RNU1-70P | 0.596799 | 1.026472 | 0.467073 | 2.197668 | 0.027973 |
| AC022150.3 | 0.91423 | -1.06503 | 0.485097 | -2.19551 | 0.028127 |
| AC107302.1 | 3.856301 | -1.86682 | 0.85247 | -2.18989 | 0.028532 |
| TRDV3 | 1.041382 | 1.250919 | 0.57265 | 2.184439 | 0.02893 |
| AC020891.4 | 1.03889 | -1.12165 | 0.514504 | -2.18006 | 0.029253 |
| NUPR2 | 1.463421 | -1.22891 | 0.564415 | -2.17732 | 0.029457 |
| IGKV2D-26 | 1.081655 | 1.150257 | 0.528501 | 2.176451 | 0.029522 |
| AC106799.3 | 0.865031 | 1.905775 | 0.875836 | 2.175951 | 0.029559 |
| AL627422.1 | 0.514318 | -1.02997 | 0.476991 | -2.15931 | 0.030826 |
| AC018639.1 | 0.656242 | -1.15573 | 0.536064 | -2.15596 | 0.031087 |
| ESRG | 1.516287 | -1.11451 | 0.517006 | -2.1557 | 0.031107 |
| CRHR1 | 0.976595 | 1.209844 | 0.561391 | 2.155082 | 0.031155 |
| HBZ | 1.880806 | -2.01326 | 0.934399 | -2.15461 | 0.031193 |
| HBE1 | 9.859424 | -1.38335 | 0.642504 | -2.15306 | 0.031314 |
| KRT24 | 1.409872 | 1.784187 | 0.831402 | 2.146 | 0.031873 |
| SYT10 | 1.510028 | 1.041097 | 0.485273 | 2.145384 | 0.031922 |
| MAGEA12 | 15.14568 | -1.80231 | 0.841036 | -2.14296 | 0.032116 |
| AC040174.2 | 1.688721 | 1.155353 | 0.539824 | 2.140243 | 0.032335 |
| PTPRQ | 0.748285 | 1.222533 | 0.572177 | 2.136633 | 0.032628 |
| MTHFD2P1 | 0.682395 | -1.29688 | 0.608454 | -2.13143 | 0.033053 |
| KRT17P1 | 0.583982 | 1.068316 | 0.501317 | 2.131019 | 0.033088 |
| RPA2P3 | 0.811662 | -1.29988 | 0.610188 | -2.1303 | 0.033147 |
| LINC02343 | 0.911325 | 1.005437 | 0.474226 | 2.120162 | 0.033992 |
| MYL6P4 | 0.422917 | -1.2424 | 0.589078 | -2.10906 | 0.034939 |
| NEFLP1 | 0.506656 | 1.287666 | 0.612494 | 2.102332 | 0.035524 |
| RPL35AP31 | 0.505861 | -1.1009 | 0.525663 | -2.09431 | 0.036233 |
| PINCR | 1.439916 | 1.270809 | 0.60698 | 2.093658 | 0.03629 |
| AC010086.1 | 0.869692 | 1.097991 | 0.527045 | 2.083297 | 0.037224 |
| AP002469.2 | 0.869187 | -1.0369 | 0.502402 | -2.06389 | 0.039028 |
| GLYAT | 0.904456 | 1.16738 | 0.565964 | 2.062638 | 0.039147 |
| FAM19A4 | 1.04452 | 1.029406 | 0.49962 | 2.060378 | 0.039362 |
| LAMP5-AS1 | 0.722488 | 1.004683 | 0.488091 | 2.058394 | 0.039552 |
| PGM5P3-AS1 | 0.897075 | 1.104317 | 0.537003 | 2.056446 | 0.03974 |
| AC015871.1 | 1.460881 | -1.04595 | 0.510298 | -2.04968 | 0.040396 |
| RN7SL494P | 0.466687 | 1.01626 | 0.496173 | 2.048199 | 0.04054 |
| AC104574.2 | 1.117313 | -1.25929 | 0.615133 | -2.04719 | 0.04064 |
| RPS4XP18 | 0.657977 | -1.01854 | 0.49818 | -2.04453 | 0.040901 |
| AP003174.1 | 0.511345 | -1.15308 | 0.564924 | -2.04113 | 0.041238 |
| CLEC3A | 1.95316 | -1.21142 | 0.596982 | -2.02924 | 0.042434 |
| IGHD3-10 | 0.547324 | 1.116745 | 0.550517 | 2.02854 | 0.042505 |
| RPL22P18 | 0.778734 | -1.09905 | 0.542777 | -2.02487 | 0.042881 |
| KCNC2 | 1.688956 | 1.219774 | 0.602499 | 2.024526 | 0.042916 |
| AL450023.1 | 0.708503 | -1.17821 | 0.58201 | -2.02438 | 0.042931 |
| AL662797.1 | 1.164221 | 1.203396 | 0.594973 | 2.022606 | 0.043114 |
| RAP2CP1 | 0.637196 | 1.054414 | 0.524121 | 2.011775 | 0.044244 |
| PIN1P1 | 0.577099 | -1.04099 | 0.517528 | -2.01147 | 0.044276 |
| SPRR2G | 0.675932 | 2.072551 | 1.031833 | 2.008611 | 0.044578 |
| FGF23 | 3.325605 | -1.02671 | 0.511268 | -2.00817 | 0.044626 |
| MIR205HG | 1.165469 | -1.31718 | 0.657734 | -2.00261 | 0.04522 |
| ACTG1P18 | 0.679525 | -1.03211 | 0.515646 | -2.00158 | 0.04533 |
| BX890604.2 | 0.552251 | -1.03234 | 0.517201 | -1.99601 | 0.045933 |
| TEX15 | 0.711275 | 1.156923 | 0.581339 | 1.990101 | 0.04658 |
| FOXC2-AS1 | 0.504902 | 1.53459 | 0.772071 | 1.987629 | 0.046853 |
| AL512638.2 | 0.536358 | 1.141806 | 0.574989 | 1.985789 | 0.047057 |
| AC012558.1 | 0.326401 | -1.23038 | 0.62088 | -1.98167 | 0.047516 |
| ATG12P2 | 0.465731 | 1.007195 | 0.509549 | 1.976641 | 0.048082 |
| ME2P1 | 1.205076 | -1.06761 | 0.540379 | -1.97566 | 0.048193 |
| SCGB1A1 | 1.191107 | 1.301659 | 0.661473 | 1.967818 | 0.049089 |
| RFPL4B | 0.644987 | 1.106448 | 0.563876 | 1.962218 | 0.049737 |
| CSAG4 | 0.688536 | -2.06083 | 1.050276 | -1.96218 | 0.049742 |
