# Supplementary material for: Prognostic and Predictive Models for Left- and Right- Colorectal Cancer Patients: A Bioinformatics Analysis Based on Ferroptosis-Related Genes
Source: Front Oncol. 2022 Feb 21;12:833834. doi: 10.3389/fonc.2022.833834 (PMC8899601; doi:10.3389/fonc.2022.833834)
Supplement: Supplementary Table 4 — Results of GO enrichment analysis related to immunity. GO – Gene Ontology. P-value <0.05. [file Table_4.docx]

|  | ONTOLOGY | ID | Description | GeneRatio | BgRatio | pvalue | p.adjust | qvalue | geneID | Count |
| --- | --- | --- | --- | --- | --- | --- | --- | --- | --- | --- |
| GO:0000353 | BP | GO:0000353 | formation of quadruple SL/U4/U5/U6 snRNP | 3/109 | 12/18670 | 4.10E-05 | 0.021571918 | 0.02069397 | RNU4-2/RNU4-1/RNU5E-1 | 3 |
| GO:0000365 | BP | GO:0000365 | mRNA trans splicing, via spliceosome | 3/109 | 12/18670 | 4.10E-05 | 0.021571918 | 0.02069397 | RNU4-2/RNU4-1/RNU5E-1 | 3 |
| GO:0045291 | BP | GO:0045291 | mRNA trans splicing, SL addition | 3/109 | 12/18670 | 4.10E-05 | 0.021571918 | 0.02069397 | RNU4-2/RNU4-1/RNU5E-1 | 3 |
| GO:0019730 | BP | GO:0019730 | antimicrobial humoral response | 6/109 | 122/18670 | 8.05E-05 | 0.031774969 | 0.030481771 | REG3A/FGB/PPBP/BPIFB1/REG1B/DEFB126 | 6 |
| GO:0000244 | BP | GO:0000244 | spliceosomal tri-snRNP complex assembly | 3/109 | 26/18670 | 0.000456381 | 0.120104285 | 0.115216204 | RNU4-2/RNU4-1/RNU5E-1 | 3 |
| GO:0034114 | BP | GO:0034114 | regulation of heterotypic cell-cell adhesion | 3/109 | 26/18670 | 0.000456381 | 0.120104285 | 0.115216204 | FGB/ADIPOQ/FGG | 3 |
| GO:0061844 | BP | GO:0061844 | antimicrobial humoral immune response mediated by antimicrobial peptide | 4/109 | 73/18670 | 0.00087787 | 0.198022432 | 0.189963188 | REG3A/PPBP/REG1B/DEFB126 | 4 |
| GO:0006959 | BP | GO:0006959 | humoral immune response | 8/109 | 356/18670 | 0.00115355 | 0.21075733 | 0.202179793 | REG3A/FGB/CPN1/PPBP/BPIFB1/REG1B/DEFB126/CD5L | 8 |
| GO:0035137 | BP | GO:0035137 | hindlimb morphogenesis | 3/109 | 36/18670 | 0.001201277 | 0.21075733 | 0.202179793 | GPC3/ALX3/SALL3 | 3 |
| GO:0090193 | BP | GO:0090193 | positive regulation of glomerulus development | 2/109 | 11/18670 | 0.001794774 | 0.283394843 | 0.271861057 | SERPINB7/ADIPOQ | 2 |
| GO:0015908 | BP | GO:0015908 | fatty acid transport | 4/109 | 97/18670 | 0.0025112 | 0.356852397 | 0.342328989 | FABP3/THRSP/SLC5A8/PLA2G2E | 4 |
| GO:0009812 | BP | GO:0009812 | flavonoid metabolic process | 2/109 | 15/18670 | 0.003374555 | 0.356852397 | 0.342328989 | CYP1A1/UGT1A3 | 2 |
| GO:0090192 | BP | GO:0090192 | regulation of glomerulus development | 2/109 | 15/18670 | 0.003374555 | 0.356852397 | 0.342328989 | SERPINB7/ADIPOQ | 2 |
| GO:1990089 | BP | GO:1990089 | response to nerve growth factor | 3/109 | 52/18670 | 0.003475202 | 0.356852397 | 0.342328989 | NTRK2/NTF4/KCNC2 | 3 |
| GO:0000387 | BP | GO:0000387 | spliceosomal snRNP assembly | 3/109 | 53/18670 | 0.003668218 | 0.356852397 | 0.342328989 | RNU4-2/RNU4-1/RNU5E-1 | 3 |
| GO:0034116 | BP | GO:0034116 | positive regulation of heterotypic cell-cell adhesion | 2/109 | 16/18670 | 0.003841983 | 0.356852397 | 0.342328989 | FGB/FGG | 2 |
| GO:0071732 | BP | GO:0071732 | cellular response to nitric oxide | 2/109 | 16/18670 | 0.003841983 | 0.356852397 | 0.342328989 | MT3/KCNC2 | 2 |
| GO:0010466 | BP | GO:0010466 | negative regulation of peptidase activity | 6/109 | 262/18670 | 0.004382243 | 0.374079838 | 0.358855296 | GPC3/SERPINB7/A2ML1/MT3/SERPINA7/CST11 | 6 |
| GO:0042743 | BP | GO:0042743 | hydrogen peroxide metabolic process | 3/109 | 57/18670 | 0.004505189 | 0.374079838 | 0.358855296 | HBQ1/MT3/CYP1A1 | 3 |
| GO:0044320 | BP | GO:0044320 | cellular response to leptin stimulus | 2/109 | 18/18670 | 0.004861403 | 0.374079838 | 0.358855296 | FGB/MT3 | 2 |
| GO:0071731 | BP | GO:0071731 | response to nitric oxide | 2/109 | 19/18670 | 0.005412718 | 0.374079838 | 0.358855296 | MT3/KCNC2 | 2 |
| GO:1902170 | BP | GO:1902170 | cellular response to reactive nitrogen species | 2/109 | 19/18670 | 0.005412718 | 0.374079838 | 0.358855296 | MT3/KCNC2 | 2 |
| GO:0034113 | BP | GO:0034113 | heterotypic cell-cell adhesion | 3/109 | 61/18670 | 0.005448915 | 0.374079838 | 0.358855296 | FGB/ADIPOQ/FGG | 3 |
| GO:0072111 | BP | GO:0072111 | cell proliferation involved in kidney development | 2/109 | 21/18670 | 0.006596887 | 0.410271718 | 0.393574215 | GPC3/SERPINB7 | 2 |
| GO:0002576 | BP | GO:0002576 | platelet degranulation | 4/109 | 128/18670 | 0.006738818 | 0.410271718 | 0.393574215 | FGB/IGF2/PPBP/FGG | 4 |
| GO:0010842 | BP | GO:0010842 | retina layer formation | 2/109 | 22/18670 | 0.007229081 | 0.410271718 | 0.393574215 | CALB1/FOXN4 | 2 |
| GO:0044321 | BP | GO:0044321 | response to leptin | 2/109 | 22/18670 | 0.007229081 | 0.410271718 | 0.393574215 | FGB/MT3 | 2 |
| GO:0015909 | BP | GO:0015909 | long-chain fatty acid transport | 3/109 | 69/18670 | 0.007670293 | 0.410271718 | 0.393574215 | FABP3/THRSP/PLA2G2E | 3 |
| GO:0072243 | BP | GO:0072243 | metanephric nephron epithelium development | 2/109 | 23/18670 | 0.007887579 | 0.410271718 | 0.393574215 | ADIPOQ/CALB1 | 2 |
| GO:0002758 | BP | GO:0002758 | innate immune response-activating signal transduction | 6/109 | 298/18670 | 0.008081425 | 0.410271718 | 0.393574215 | MUC6/FGB/BPIFB1/SFTPA1/FGG/MUC15 | 6 |
| GO:1900118 | BP | GO:1900118 | negative regulation of execution phase of apoptosis | 2/109 | 24/18670 | 0.008572058 | 0.410271718 | 0.393574215 | MTRNR2L8/CIDEA | 2 |
| GO:0010876 | BP | GO:0010876 | lipid localization | 7/109 | 400/18670 | 0.00904491 | 0.410271718 | 0.393574215 | FABP3/THRSP/ADIPOQ/CIDEA/SFTPA1/SLC5A8/PLA2G2E | 7 |
| GO:0031639 | BP | GO:0031639 | plasminogen activation | 2/109 | 25/18670 | 0.009282199 | 0.410271718 | 0.393574215 | FGB/FGG | 2 |
| GO:0072006 | BP | GO:0072006 | nephron development | 4/109 | 142/18670 | 0.009640185 | 0.410271718 | 0.393574215 | GPC3/SERPINB7/ADIPOQ/CALB1 | 4 |
| GO:0002224 | BP | GO:0002224 | toll-like receptor signaling pathway | 4/109 | 146/18670 | 0.010597273 | 0.410271718 | 0.393574215 | FGB/BPIFB1/SFTPA1/FGG | 4 |
| GO:0072207 | BP | GO:0072207 | metanephric epithelium development | 2/109 | 27/18670 | 0.010778198 | 0.410271718 | 0.393574215 | ADIPOQ/CALB1 | 2 |
| GO:0072378 | BP | GO:0072378 | blood coagulation, fibrin clot formation | 2/109 | 27/18670 | 0.010778198 | 0.410271718 | 0.393574215 | FGB/FGG | 2 |
| GO:0002218 | BP | GO:0002218 | activation of innate immune response | 6/109 | 319/18670 | 0.011067937 | 0.410271718 | 0.393574215 | MUC6/FGB/BPIFB1/SFTPA1/FGG/MUC15 | 6 |
| GO:0009914 | BP | GO:0009914 | hormone transport | 6/109 | 322/18670 | 0.011550765 | 0.410271718 | 0.393574215 | FGB/ADIPOQ/SERPINA7/KCNC2/GLP1R/FGG | 6 |
| GO:0035116 | BP | GO:0035116 | embryonic hindlimb morphogenesis | 2/109 | 28/18670 | 0.011563431 | 0.410271718 | 0.393574215 | GPC3/ALX3 | 2 |
| GO:0042730 | BP | GO:0042730 | fibrinolysis | 2/109 | 28/18670 | 0.011563431 | 0.410271718 | 0.393574215 | FGB/FGG | 2 |
| GO:1905954 | BP | GO:1905954 | positive regulation of lipid localization | 3/109 | 82/18670 | 0.012277606 | 0.410271718 | 0.393574215 | FABP3/ADIPOQ/CIDEA | 3 |
| GO:0048240 | BP | GO:0048240 | sperm capacitation | 2/109 | 29/18670 | 0.012373071 | 0.410271718 | 0.393574215 | TCP11/ROPN1 | 2 |
| GO:0071280 | BP | GO:0071280 | cellular response to copper ion | 2/109 | 29/18670 | 0.012373071 | 0.410271718 | 0.393574215 | MT3/CYP1A1 | 2 |
| GO:0010770 | BP | GO:0010770 | positive regulation of cell morphogenesis involved in differentiation | 4/109 | 154/18670 | 0.012691223 | 0.410271718 | 0.393574215 | NTRK2/FGB/L1CAM/FGG | 4 |
| GO:0015849 | BP | GO:0015849 | organic acid transport | 6/109 | 333/18670 | 0.013448889 | 0.410271718 | 0.393574215 | FABP3/NTRK2/THRSP/SLC38A8/SLC5A8/PLA2G2E | 6 |
| GO:0046942 | BP | GO:0046942 | carboxylic acid transport | 6/109 | 333/18670 | 0.013448889 | 0.410271718 | 0.393574215 | FABP3/NTRK2/THRSP/SLC38A8/SLC5A8/PLA2G2E | 6 |
| GO:0007202 | BP | GO:0007202 | activation of phospholipase C activity | 2/109 | 31/18670 | 0.014064357 | 0.410271718 | 0.393574215 | NTRK2/NTF4 | 2 |
| GO:0045879 | BP | GO:0045879 | negative regulation of smoothened signaling pathway | 2/109 | 31/18670 | 0.014064357 | 0.410271718 | 0.393574215 | GPC3/SALL3 | 2 |
| GO:0051781 | BP | GO:0051781 | positive regulation of cell division | 3/109 | 87/18670 | 0.014390834 | 0.410271718 | 0.393574215 | IGF2/PPBP/PROK1 | 3 |
| GO:0097194 | BP | GO:0097194 | execution phase of apoptosis | 3/109 | 88/18670 | 0.014836659 | 0.410271718 | 0.393574215 | MTRNR2L8/XKR7/CIDEA | 3 |
| GO:0007616 | BP | GO:0007616 | long-term memory | 2/109 | 32/18670 | 0.014945396 | 0.410271718 | 0.393574215 | NTF4/CALB1 | 2 |
| GO:0042573 | BP | GO:0042573 | retinoic acid metabolic process | 2/109 | 32/18670 | 0.014945396 | 0.410271718 | 0.393574215 | CYP1A1/UGT1A3 | 2 |
| GO:0042744 | BP | GO:0042744 | hydrogen peroxide catabolic process | 2/109 | 32/18670 | 0.014945396 | 0.410271718 | 0.393574215 | HBQ1/MT3 | 2 |
| GO:0045907 | BP | GO:0045907 | positive regulation of vasoconstriction | 2/109 | 32/18670 | 0.014945396 | 0.410271718 | 0.393574215 | FGB/FGG | 2 |
| GO:0015718 | BP | GO:0015718 | monocarboxylic acid transport | 4/109 | 162/18670 | 0.015033056 | 0.410271718 | 0.393574215 | FABP3/THRSP/SLC5A8/PLA2G2E | 4 |
| GO:0010951 | BP | GO:0010951 | negative regulation of endopeptidase activity | 5/109 | 250/18670 | 0.015673384 | 0.410271718 | 0.393574215 | SERPINB7/A2ML1/MT3/SERPINA7/CST11 | 5 |
| GO:0001656 | BP | GO:0001656 | metanephros development | 3/109 | 90/18670 | 0.015751621 | 0.410271718 | 0.393574215 | GPC3/ADIPOQ/CALB1 | 3 |
| GO:0010737 | BP | GO:0010737 | protein kinase A signaling | 2/109 | 33/18670 | 0.015849636 | 0.410271718 | 0.393574215 | ADIPOQ/TCP11 | 2 |
| GO:0048011 | BP | GO:0048011 | neurotrophin TRK receptor signaling pathway | 2/109 | 33/18670 | 0.015849636 | 0.410271718 | 0.393574215 | NTRK2/NTF4 | 2 |
| GO:2000352 | BP | GO:2000352 | negative regulation of endothelial cell apoptotic process | 2/109 | 33/18670 | 0.015849636 | 0.410271718 | 0.393574215 | FGB/FGG | 2 |
| GO:1900026 | BP | GO:1900026 | positive regulation of substrate adhesion-dependent cell spreading | 2/109 | 37/18670 | 0.019692712 | 0.440196889 | 0.422281473 | FGB/FGG | 2 |
| GO:0045861 | BP | GO:0045861 | negative regulation of proteolysis | 6/109 | 363/18670 | 0.019717852 | 0.440196889 | 0.422281473 | GPC3/SERPINB7/A2ML1/MT3/SERPINA7/CST11 | 6 |
| GO:0046883 | BP | GO:0046883 | regulation of hormone secretion | 5/109 | 266/18670 | 0.019928458 | 0.440196889 | 0.422281473 | FGB/ADIPOQ/KCNC2/GLP1R/FGG | 5 |
| GO:0048469 | BP | GO:0048469 | cell maturation | 4/109 | 177/18670 | 0.020121995 | 0.440196889 | 0.422281473 | EPHA8/TCP11/ROPN1/FGG | 4 |
| GO:0006869 | BP | GO:0006869 | lipid transport | 6/109 | 365/18670 | 0.020196081 | 0.440196889 | 0.422281473 | FABP3/THRSP/ADIPOQ/SFTPA1/SLC5A8/PLA2G2E | 6 |
| GO:0032094 | BP | GO:0032094 | response to food | 2/109 | 38/18670 | 0.020708565 | 0.440196889 | 0.422281473 | MT3/CYP1A1 | 2 |
| GO:0046326 | BP | GO:0046326 | positive regulation of glucose import | 2/109 | 38/18670 | 0.020708565 | 0.440196889 | 0.422281473 | GPC3/ADIPOQ | 2 |
| GO:0022408 | BP | GO:0022408 | negative regulation of cell-cell adhesion | 4/109 | 181/18670 | 0.021637706 | 0.440196889 | 0.422281473 | CCL25/ADIPOQ/VTCN1/FGG | 4 |
| GO:0038179 | BP | GO:0038179 | neurotrophin signaling pathway | 2/109 | 39/18670 | 0.021745882 | 0.440196889 | 0.422281473 | NTRK2/NTF4 | 2 |
| GO:1902042 | BP | GO:1902042 | negative regulation of extrinsic apoptotic signaling pathway via death domain receptors | 2/109 | 39/18670 | 0.021745882 | 0.440196889 | 0.422281473 | FGB/FGG | 2 |
| GO:0090184 | BP | GO:0090184 | positive regulation of kidney development | 2/109 | 40/18670 | 0.022804383 | 0.440196889 | 0.422281473 | SERPINB7/ADIPOQ | 2 |
| GO:0001523 | BP | GO:0001523 | retinoid metabolic process | 3/109 | 104/18670 | 0.02303338 | 0.440196889 | 0.422281473 | GPC3/CYP1A1/UGT1A3 | 3 |
| GO:0030593 | BP | GO:0030593 | neutrophil chemotaxis | 3/109 | 104/18670 | 0.02303338 | 0.440196889 | 0.422281473 | CCL25/PPBP/IL36B | 3 |
| GO:0035136 | BP | GO:0035136 | forelimb morphogenesis | 2/109 | 41/18670 | 0.023883789 | 0.440196889 | 0.422281473 | ALX3/SALL3 | 2 |
| GO:1900117 | BP | GO:1900117 | regulation of execution phase of apoptosis | 2/109 | 41/18670 | 0.023883789 | 0.440196889 | 0.422281473 | MTRNR2L8/CIDEA | 2 |
| GO:0045089 | BP | GO:0045089 | positive regulation of innate immune response | 6/109 | 381/18670 | 0.024309277 | 0.440196889 | 0.422281473 | MUC6/FGB/BPIFB1/SFTPA1/FGG/MUC15 | 6 |
| GO:1904659 | BP | GO:1904659 | glucose transmembrane transport | 3/109 | 108/18670 | 0.025397064 | 0.440196889 | 0.422281473 | GPC3/ADIPOQ/PPBP | 3 |
| GO:0072009 | BP | GO:0072009 | nephron epithelium development | 3/109 | 109/18670 | 0.026007657 | 0.440196889 | 0.422281473 | GPC3/ADIPOQ/CALB1 | 3 |
| GO:0010863 | BP | GO:0010863 | positive regulation of phospholipase C activity | 2/109 | 43/18670 | 0.026104219 | 0.440196889 | 0.422281473 | NTRK2/NTF4 | 2 |
| GO:0032365 | BP | GO:0032365 | intracellular lipid transport | 2/109 | 43/18670 | 0.026104219 | 0.440196889 | 0.422281473 | FABP3/THRSP | 2 |
| GO:0016101 | BP | GO:0016101 | diterpenoid metabolic process | 3/109 | 110/18670 | 0.02662611 | 0.440196889 | 0.422281473 | GPC3/CYP1A1/UGT1A3 | 3 |
| GO:0010828 | BP | GO:0010828 | positive regulation of glucose transmembrane transport | 2/109 | 44/18670 | 0.0272447 | 0.440196889 | 0.422281473 | GPC3/ADIPOQ | 2 |
| GO:0046688 | BP | GO:0046688 | response to copper ion | 2/109 | 44/18670 | 0.0272447 | 0.440196889 | 0.422281473 | MT3/CYP1A1 | 2 |
| GO:0008645 | BP | GO:0008645 | hexose transmembrane transport | 3/109 | 112/18670 | 0.027886576 | 0.440196889 | 0.422281473 | GPC3/ADIPOQ/PPBP | 3 |
| GO:0002221 | BP | GO:0002221 | pattern recognition receptor signaling pathway | 4/109 | 197/18670 | 0.028389745 | 0.440196889 | 0.422281473 | FGB/BPIFB1/SFTPA1/FGG | 4 |
| GO:1900274 | BP | GO:1900274 | regulation of phospholipase C activity | 2/109 | 45/18670 | 0.028405 | 0.440196889 | 0.422281473 | NTRK2/NTF4 | 2 |
| GO:0072376 | BP | GO:0072376 | protein activation cascade | 4/109 | 198/18670 | 0.028848887 | 0.440196889 | 0.422281473 | FGB/CPN1/CD5L/FGG | 4 |
| GO:0015749 | BP | GO:0015749 | monosaccharide transmembrane transport | 3/109 | 114/18670 | 0.029178411 | 0.440196889 | 0.422281473 | GPC3/ADIPOQ/PPBP | 3 |
| GO:1904036 | BP | GO:1904036 | negative regulation of epithelial cell apoptotic process | 2/109 | 46/18670 | 0.029584853 | 0.440196889 | 0.422281473 | FGB/FGG | 2 |
| GO:0034219 | BP | GO:0034219 | carbohydrate transmembrane transport | 3/109 | 116/18670 | 0.030501547 | 0.440196889 | 0.422281473 | GPC3/ADIPOQ/PPBP | 3 |
| GO:0120163 | BP | GO:0120163 | negative regulation of cold-induced thermogenesis | 2/109 | 47/18670 | 0.030783995 | 0.440196889 | 0.422281473 | ADIPOQ/CIDEA | 2 |
| GO:0022407 | BP | GO:0022407 | regulation of cell-cell adhesion | 6/109 | 403/18670 | 0.030833353 | 0.440196889 | 0.422281473 | CCL25/FGB/ADIPOQ/IGF2/VTCN1/FGG | 6 |
| GO:0070372 | BP | GO:0070372 | regulation of ERK1 and ERK2 cascade | 5/109 | 300/18670 | 0.031327882 | 0.440196889 | 0.422281473 | CCL25/FGB/ADIPOQ/MT3/FGG | 5 |
| GO:0034764 | BP | GO:0034764 | positive regulation of transmembrane transport | 4/109 | 204/18670 | 0.031696636 | 0.440196889 | 0.422281473 | GPC3/HTR3A/ADIPOQ/KCNC2 | 4 |
| GO:0010769 | BP | GO:0010769 | regulation of cell morphogenesis involved in differentiation | 5/109 | 301/18670 | 0.031714036 | 0.440196889 | 0.422281473 | NTRK2/FGB/L1CAM/MT3/FGG | 5 |
| GO:1990266 | BP | GO:1990266 | neutrophil migration | 3/109 | 118/18670 | 0.031855908 | 0.440196889 | 0.422281473 | CCL25/PPBP/IL36B | 3 |
| GO:1903727 | BP | GO:1903727 | positive regulation of phospholipid metabolic process | 2/109 | 48/18670 | 0.032002166 | 0.440196889 | 0.422281473 | EPHA8/FABP3 | 2 |
| GO:0019216 | BP | GO:0019216 | regulation of lipid metabolic process | 6/109 | 410/18670 | 0.033128529 | 0.440196889 | 0.422281473 | EPHA8/FABP3/THRSP/ADIPOQ/CYP1A1/CIDEA | 6 |
| GO:0060042 | BP | GO:0060042 | retina morphogenesis in camera-type eye | 2/109 | 49/18670 | 0.033239107 | 0.440196889 | 0.422281473 | CALB1/FOXN4 | 2 |
| GO:1990090 | BP | GO:1990090 | cellular response to nerve growth factor stimulus | 2/109 | 49/18670 | 0.033239107 | 0.440196889 | 0.422281473 | NTRK2/NTF4 | 2 |
| GO:0006721 | BP | GO:0006721 | terpenoid metabolic process | 3/109 | 120/18670 | 0.033241406 | 0.440196889 | 0.422281473 | GPC3/CYP1A1/UGT1A3 | 3 |
| GO:0090276 | BP | GO:0090276 | regulation of peptide hormone secretion | 4/109 | 208/18670 | 0.033683989 | 0.440196889 | 0.422281473 | FGB/KCNC2/GLP1R/FGG | 4 |
| GO:0010811 | BP | GO:0010811 | positive regulation of cell-substrate adhesion | 3/109 | 121/18670 | 0.033945799 | 0.440196889 | 0.422281473 | CCL25/FGB/FGG | 3 |
| GO:0071621 | BP | GO:0071621 | granulocyte chemotaxis | 3/109 | 123/18670 | 0.03537781 | 0.440196889 | 0.422281473 | CCL25/PPBP/IL36B | 3 |
| GO:0097366 | BP | GO:0097366 | response to bronchodilator | 2/109 | 51/18670 | 0.035768277 | 0.440196889 | 0.422281473 | MT3/KCNC2 | 2 |
| GO:0046879 | BP | GO:0046879 | hormone secretion | 5/109 | 312/18670 | 0.036159887 | 0.440196889 | 0.422281473 | FGB/ADIPOQ/KCNC2/GLP1R/FGG | 5 |
| GO:0045471 | BP | GO:0045471 | response to ethanol | 3/109 | 125/18670 | 0.036840685 | 0.440196889 | 0.422281473 | HTR3A/ADIPOQ/KCNC2 | 3 |
| GO:1900024 | BP | GO:1900024 | regulation of substrate adhesion-dependent cell spreading | 2/109 | 52/18670 | 0.03706 | 0.440196889 | 0.422281473 | FGB/FGG | 2 |
| GO:0070374 | BP | GO:0070374 | positive regulation of ERK1 and ERK2 cascade | 4/109 | 215/18670 | 0.037333794 | 0.440196889 | 0.422281473 | CCL25/FGB/MT3/FGG | 4 |
| GO:0070371 | BP | GO:0070371 | ERK1 and ERK2 cascade | 5/109 | 317/18670 | 0.038301912 | 0.440196889 | 0.422281473 | CCL25/FGB/ADIPOQ/MT3/FGG | 5 |
| GO:0030195 | BP | GO:0030195 | negative regulation of blood coagulation | 2/109 | 53/18670 | 0.038369482 | 0.440196889 | 0.422281473 | FGB/FGG | 2 |
| GO:0031638 | BP | GO:0031638 | zymogen activation | 2/109 | 53/18670 | 0.038369482 | 0.440196889 | 0.422281473 | FGB/FGG | 2 |
| GO:0061512 | BP | GO:0061512 | protein localization to cilium | 2/109 | 54/18670 | 0.039696475 | 0.440196889 | 0.422281473 | ROPN1/GFY | 2 |
| GO:1900047 | BP | GO:1900047 | negative regulation of hemostasis | 2/109 | 54/18670 | 0.039696475 | 0.440196889 | 0.422281473 | FGB/FGG | 2 |
| GO:0090183 | BP | GO:0090183 | regulation of kidney development | 2/109 | 56/18670 | 0.042402018 | 0.440196889 | 0.422281473 | SERPINB7/ADIPOQ | 2 |
| GO:0043434 | BP | GO:0043434 | response to peptide hormone | 6/109 | 436/18670 | 0.042616692 | 0.440196889 | 0.422281473 | REG3A/FABP3/ADIPOQ/IGF2/GLP1R/REG1B | 6 |
| GO:0007160 | BP | GO:0007160 | cell-matrix adhesion | 4/109 | 225/18670 | 0.042928827 | 0.440196889 | 0.422281473 | CCL25/FGB/L1CAM/FGG | 4 |
| GO:0050819 | BP | GO:0050819 | negative regulation of coagulation | 2/109 | 57/18670 | 0.043780085 | 0.440196889 | 0.422281473 | FGB/FGG | 2 |
| GO:1903825 | BP | GO:1903825 | organic acid transmembrane transport | 3/109 | 135/18670 | 0.044613263 | 0.440196889 | 0.422281473 | THRSP/SLC38A8/SLC5A8 | 3 |
| GO:1905039 | BP | GO:1905039 | carboxylic acid transmembrane transport | 3/109 | 135/18670 | 0.044613263 | 0.440196889 | 0.422281473 | THRSP/SLC38A8/SLC5A8 | 3 |
| GO:0017001 | BP | GO:0017001 | antibiotic catabolic process | 2/109 | 58/18670 | 0.045174696 | 0.440196889 | 0.422281473 | HBQ1/MT3 | 2 |
| GO:0019229 | BP | GO:0019229 | regulation of vasoconstriction | 2/109 | 58/18670 | 0.045174696 | 0.440196889 | 0.422281473 | FGB/FGG | 2 |
| GO:1902041 | BP | GO:1902041 | regulation of extrinsic apoptotic signaling pathway via death domain receptors | 2/109 | 58/18670 | 0.045174696 | 0.440196889 | 0.422281473 | FGB/FGG | 2 |
| GO:0016042 | BP | GO:0016042 | lipid catabolic process | 5/109 | 333/18670 | 0.045672327 | 0.440196889 | 0.422281473 | FABP3/ADIPOQ/MT3/CIDEA/PLA2G2E | 5 |
| GO:0010518 | BP | GO:0010518 | positive regulation of phospholipase activity | 2/109 | 59/18670 | 0.046585616 | 0.440196889 | 0.422281473 | NTRK2/NTF4 | 2 |
| GO:0070527 | BP | GO:0070527 | platelet aggregation | 2/109 | 59/18670 | 0.046585616 | 0.440196889 | 0.422281473 | FGB/FGG | 2 |
| GO:2000351 | BP | GO:2000351 | regulation of endothelial cell apoptotic process | 2/109 | 59/18670 | 0.046585616 | 0.440196889 | 0.422281473 | FGB/FGG | 2 |
| GO:0006720 | BP | GO:0006720 | isoprenoid metabolic process | 3/109 | 139/18670 | 0.047933178 | 0.440196889 | 0.422281473 | GPC3/CYP1A1/UGT1A3 | 3 |
| GO:0018149 | BP | GO:0018149 | peptide cross-linking | 2/109 | 60/18670 | 0.048012611 | 0.440196889 | 0.422281473 | TGM6/LCE1E | 2 |
| GO:0033013 | BP | GO:0033013 | tetrapyrrole metabolic process | 2/109 | 60/18670 | 0.048012611 | 0.440196889 | 0.422281473 | PRSS1/CYP1A1 | 2 |
| GO:0046324 | BP | GO:0046324 | regulation of glucose import | 2/109 | 60/18670 | 0.048012611 | 0.440196889 | 0.422281473 | GPC3/ADIPOQ | 2 |
| GO:0042737 | BP | GO:0042737 | drug catabolic process | 3/109 | 140/18670 | 0.04878171 | 0.440196889 | 0.422281473 | HBQ1/MT3/CPN1 | 3 |
| GO:0072073 | BP | GO:0072073 | kidney epithelium development | 3/109 | 140/18670 | 0.04878171 | 0.440196889 | 0.422281473 | GPC3/ADIPOQ/CALB1 | 3 |
| GO:0045088 | BP | GO:0045088 | regulation of innate immune response | 6/109 | 452/18670 | 0.049229708 | 0.440196889 | 0.422281473 | MUC6/FGB/BPIFB1/SFTPA1/FGG/MUC15 | 6 |
| GO:0052547 | BP | GO:0052547 | regulation of peptidase activity | 6/109 | 452/18670 | 0.049229708 | 0.440196889 | 0.422281473 | GPC3/SERPINB7/A2ML1/MT3/SERPINA7/CST11 | 6 |
| GO:0016266 | BP | GO:0016266 | O-glycan processing | 2/109 | 61/18670 | 0.049455448 | 0.440196889 | 0.422281473 | MUC6/MUC15 | 2 |
| GO:0032370 | BP | GO:0032370 | positive regulation of lipid transport | 2/109 | 61/18670 | 0.049455448 | 0.440196889 | 0.422281473 | FABP3/ADIPOQ | 2 |
| GO:0042490 | BP | GO:0042490 | mechanoreceptor differentiation | 2/109 | 61/18670 | 0.049455448 | 0.440196889 | 0.422281473 | NTRK2/NTF4 | 2 |
| GO:0097530 | BP | GO:0097530 | granulocyte migration | 3/109 | 141/18670 | 0.049637616 | 0.440196889 | 0.422281473 | CCL25/PPBP/IL36B | 3 |
