# Supplementary material for: Prognostic and Predictive Models for Left- and Right- Colorectal Cancer Patients: A Bioinformatics Analysis Based on Ferroptosis-Related Genes
Source: Front Oncol. 2022 Feb 21;12:833834. doi: 10.3389/fonc.2022.833834 (PMC8899601; doi:10.3389/fonc.2022.833834)
Supplement: Supplementary Table 5 — Results of GO enrichment analysis related to lipid-related biological processes. P-value <0.05. [file Table_5.docx]

|  | ONTOLOGY | ID | Description | GeneRatio | BgRatio | pvalue | p.adjust | qvalue | geneID | Count |
| --- | --- | --- | --- | --- | --- | --- | --- | --- | --- | --- |
| GO:0034378 | BP | GO:0034378 | chylomicron assembly | 5/219 | 10/18670 | 5.10E-08 | 8.08E-05 | 7.29E-05 | APOA2/APOA1/APOA4/APOC3/APOB | 5 |
| GO:0015669 | BP | GO:0015669 | gas transport | 6/219 | 19/18670 | 5.81E-08 | 8.08E-05 | 7.29E-05 | AQP5/HBG2/HBQ1/RHAG/HBZ/HBE1 | 6 |
| GO:0034370 | BP | GO:0034370 | triglyceride-rich lipoprotein particle remodeling | 5/219 | 14/18670 | 3.90E-07 | 0.00035021 | 0.000316131 | APOA2/APOA1/APOA4/APOC3/APOB | 5 |
| GO:0034368 | BP | GO:0034368 | protein-lipid complex remodeling | 6/219 | 28/18670 | 7.39E-07 | 0.00035021 | 0.000316131 | ALB/APOA2/APOA1/APOA4/APOC3/APOB | 6 |
| GO:0034369 | BP | GO:0034369 | plasma lipoprotein particle remodeling | 6/219 | 28/18670 | 7.39E-07 | 0.00035021 | 0.000316131 | ALB/APOA2/APOA1/APOA4/APOC3/APOB | 6 |
| GO:0007586 | BP | GO:0007586 | digestion | 11/219 | 139/18670 | 7.56E-07 | 0.00035021 | 0.000316131 | AKR1C2/AQP5/APOA2/LEP/PRSS1/MUC6/APOA1/APOA4/PRSS2/SLC2A2/SERPINA3 | 11 |
| GO:0034367 | BP | GO:0034367 | protein-containing complex remodeling | 6/219 | 29/18670 | 9.22E-07 | 0.000366263 | 0.000330621 | ALB/APOA2/APOA1/APOA4/APOC3/APOB | 6 |
| GO:0034375 | BP | GO:0034375 | high-density lipoprotein particle remodeling | 5/219 | 18/18670 | 1.61E-06 | 0.000557791 | 0.000503512 | ALB/APOA2/APOA1/APOA4/APOC3 | 5 |
| GO:0001523 | BP | GO:0001523 | retinoid metabolic process | 9/219 | 104/18670 | 3.74E-06 | 0.001153838 | 0.001041556 | APOA2/CYP1B1/APOA1/APOA4/ADH1B/LRP2/TTR/APOC3/APOB | 9 |
| GO:0016101 | BP | GO:0016101 | diterpenoid metabolic process | 9/219 | 110/18670 | 5.94E-06 | 0.001569679 | 0.001416931 | APOA2/CYP1B1/APOA1/APOA4/ADH1B/LRP2/TTR/APOC3/APOB | 9 |
| GO:0033700 | BP | GO:0033700 | phospholipid efflux | 4/219 | 12/18670 | 8.47E-06 | 0.001569679 | 0.001416931 | APOA2/APOA1/APOA4/APOC3 | 4 |
| GO:0034372 | BP | GO:0034372 | very-low-density lipoprotein particle remodeling | 4/219 | 12/18670 | 8.47E-06 | 0.001569679 | 0.001416931 | APOA2/APOA1/APOA4/APOC3 | 4 |
| GO:0006721 | BP | GO:0006721 | terpenoid metabolic process | 9/219 | 120/18670 | 1.21E-05 | 0.002101155 | 0.001896689 | APOA2/CYP1B1/APOA1/APOA4/ADH1B/LRP2/TTR/APOC3/APOB | 9 |
| GO:0071827 | BP | GO:0071827 | plasma lipoprotein particle organization | 6/219 | 45/18670 | 1.35E-05 | 0.002211381 | 0.001996188 | ALB/APOA2/APOA1/APOA4/APOC3/APOB | 6 |
| GO:0034377 | BP | GO:0034377 | plasma lipoprotein particle assembly | 5/219 | 28/18670 | 1.67E-05 | 0.002584361 | 0.002332873 | APOA2/APOA1/APOA4/APOC3/APOB | 5 |
| GO:0071825 | BP | GO:0071825 | protein-lipid complex subunit organization | 6/219 | 49/18670 | 2.23E-05 | 0.002939151 | 0.002653138 | ALB/APOA2/APOA1/APOA4/APOC3/APOB | 6 |
| GO:0015671 | BP | GO:0015671 | oxygen transport | 4/219 | 15/18670 | 2.27E-05 | 0.002939151 | 0.002653138 | HBG2/HBQ1/HBZ/HBE1 | 4 |
| GO:0030299 | BP | GO:0030299 | intestinal cholesterol absorption | 4/219 | 15/18670 | 2.27E-05 | 0.002939151 | 0.002653138 | APOA2/LEP/APOA1/APOA4 | 4 |
| GO:0022600 | BP | GO:0022600 | digestive system process | 8/219 | 100/18670 | 2.33E-05 | 0.002939151 | 0.002653138 | AQP5/APOA2/LEP/MUC6/APOA1/APOA4/SLC2A2/SERPINA3 | 8 |
| GO:0042744 | BP | GO:0042744 | hydrogen peroxide catabolic process | 5/219 | 32/18670 | 3.30E-05 | 0.003822757 | 0.003450759 | APOA4/HBG2/HBQ1/HBZ/HBE1 | 5 |
| GO:0065005 | BP | GO:0065005 | protein-lipid complex assembly | 5/219 | 32/18670 | 3.30E-05 | 0.003822757 | 0.003450759 | APOA2/APOA1/APOA4/APOC3/APOB | 5 |
| GO:0050905 | BP | GO:0050905 | neuromuscular process | 8/219 | 107/18670 | 3.80E-05 | 0.003883546 | 0.003505631 | CTNNA2/CSMD1/HMX3/TNR/NRXN1/GRIN2A/IGDCC3/GBX1 | 8 |
| GO:0008202 | BP | GO:0008202 | steroid metabolic process | 14/219 | 331/18670 | 3.85E-05 | 0.003883546 | 0.003505631 | AKR1C2/AFP/APOA2/LEP/CYP1B1/CYP7A1/ACADL/SULT4A1/APOA1/APOA4/G6PC/LRP2/APOB/FGF23 | 14 |
| GO:0098856 | BP | GO:0098856 | intestinal lipid absorption | 4/219 | 17/18670 | 3.89E-05 | 0.003883546 | 0.003505631 | APOA2/LEP/APOA1/APOA4 | 4 |
| GO:0006720 | BP | GO:0006720 | isoprenoid metabolic process | 9/219 | 139/18670 | 3.91E-05 | 0.003883546 | 0.003505631 | APOA2/CYP1B1/APOA1/APOA4/ADH1B/LRP2/TTR/APOC3/APOB | 9 |
| GO:0060192 | BP | GO:0060192 | negative regulation of lipase activity | 4/219 | 18/18670 | 4.95E-05 | 0.004748826 | 0.00428671 | APOA2/APOA1/ANXA8/APOC3 | 4 |
| GO:0033344 | BP | GO:0033344 | cholesterol efflux | 6/219 | 57/18670 | 5.36E-05 | 0.004966138 | 0.004482875 | APOA2/APOA1/APOA4/ADIPOQ/APOC3/APOB | 6 |
| GO:0044241 | BP | GO:0044241 | lipid digestion | 4/219 | 19/18670 | 6.22E-05 | 0.005575505 | 0.005032943 | APOA2/LEP/APOA1/APOA4 | 4 |
| GO:0015850 | BP | GO:0015850 | organic hydroxy compound transport | 12/219 | 262/18670 | 6.51E-05 | 0.005652324 | 0.005102287 | APOA2/LEP/SLC10A2/APOA1/APOA4/HRH3/ADIPOQ/APOC3/APOB/SLC5A8/CRHR1/SYT10 | 12 |
| GO:0043691 | BP | GO:0043691 | reverse cholesterol transport | 4/219 | 20/18670 | 7.70E-05 | 0.006352071 | 0.005733941 | APOA2/APOA1/APOA4/APOC3 | 4 |
| GO:0050892 | BP | GO:0050892 | intestinal absorption | 5/219 | 38/18670 | 7.77E-05 | 0.006352071 | 0.005733941 | APOA2/LEP/APOA1/APOA4/SLC2A2 | 5 |
| GO:0006959 | BP | GO:0006959 | humoral immune response | 14/219 | 356/18670 | 8.42E-05 | 0.006677508 | 0.006027709 | MS4A1/LTF/SUSD4/DEFA5/IGHE/C7/BPIFB2/PAX5/PRSS2/FGB/DEFA6/IGHV2-70/DEFB4A/CPN1 | 14 |
| GO:0032374 | BP | GO:0032374 | regulation of cholesterol transport | 6/219 | 62/18670 | 8.65E-05 | 0.006677508 | 0.006027709 | APOA2/LEP/APOA1/APOA4/ADIPOQ/APOC3 | 6 |
| GO:0032371 | BP | GO:0032371 | regulation of sterol transport | 6/219 | 63/18670 | 9.46E-05 | 0.007111362 | 0.006419344 | APOA2/LEP/APOA1/APOA4/ADIPOQ/APOC3 | 6 |
| GO:0097305 | BP | GO:0097305 | response to alcohol | 11/219 | 233/18670 | 0.000100377 | 0.007343338 | 0.006628746 | AKR1C2/LEP/CCL19/CYP7A1/CDO1/ADCYAP1R1/GRIN2A/ADIPOQ/CLDN18/CRHR1/KCNC2 | 11 |
| GO:0045471 | BP | GO:0045471 | response to ethanol | 8/219 | 125/18670 | 0.000114481 | 0.008160464 | 0.007366356 | LEP/CDO1/ADCYAP1R1/GRIN2A/ADIPOQ/CLDN18/CRHR1/KCNC2 | 8 |
| GO:0002576 | BP | GO:0002576 | platelet degranulation | 8/219 | 128/18670 | 0.00013505 | 0.009006449 | 0.008130016 | IGF2/ALB/ORM2/AHSG/APOA1/FGB/SERPINA3/FGG | 8 |
| GO:0042632 | BP | GO:0042632 | cholesterol homeostasis | 7/219 | 96/18670 | 0.00013669 | 0.009006449 | 0.008130016 | APOA2/CYP7A1/APOA1/APOA4/G6PC/APOC3/APOB | 7 |
| GO:0046461 | BP | GO:0046461 | neutral lipid catabolic process | 5/219 | 43/18670 | 0.000142059 | 0.009006449 | 0.008130016 | APOA2/APOA1/APOA4/APOC3/APOB | 5 |
| GO:0046464 | BP | GO:0046464 | acylglycerol catabolic process | 5/219 | 43/18670 | 0.000142059 | 0.009006449 | 0.008130016 | APOA2/APOA1/APOA4/APOC3/APOB | 5 |
| GO:0006639 | BP | GO:0006639 | acylglycerol metabolic process | 8/219 | 129/18670 | 0.000142548 | 0.009006449 | 0.008130016 | GPAT2/APOA2/APOA1/APOA4/G6PC/APOC3/APOB/DGKK | 8 |
| GO:0055092 | BP | GO:0055092 | sterol homeostasis | 7/219 | 97/18670 | 0.000145874 | 0.009011742 | 0.008134795 | APOA2/CYP7A1/APOA1/APOA4/G6PC/APOC3/APOB | 7 |
| GO:0006638 | BP | GO:0006638 | neutral lipid metabolic process | 8/219 | 130/18670 | 0.000150386 | 0.009088564 | 0.008204141 | GPAT2/APOA2/APOA1/APOA4/G6PC/APOC3/APOB/DGKK | 8 |
| GO:0015893 | BP | GO:0015893 | drug transport | 10/219 | 205/18670 | 0.000159087 | 0.009403307 | 0.008488256 | AQP5/SLC7A10/HBG2/LRP2/HBQ1/SLC5A8/RHAG/HBZ/HBE1/SYT10 | 10 |
| GO:0002227 | BP | GO:0002227 | innate immune response in mucosa | 4/219 | 24/18670 | 0.000162782 | 0.009403307 | 0.008488256 | LTF/DEFA5/APOA4/DEFA6 | 4 |
| GO:0030301 | BP | GO:0030301 | cholesterol transport | 7/219 | 99/18670 | 0.000165742 | 0.009403307 | 0.008488256 | APOA2/LEP/APOA1/APOA4/ADIPOQ/APOC3/APOB | 7 |
| GO:0035376 | BP | GO:0035376 | sterol import | 3/219 | 10/18670 | 0.000179773 | 0.009799374 | 0.008845781 | APOA2/APOA1/APOC3 | 3 |
| GO:0070508 | BP | GO:0070508 | cholesterol import | 3/219 | 10/18670 | 0.000179773 | 0.009799374 | 0.008845781 | APOA2/APOA1/APOC3 | 3 |
| GO:0097006 | BP | GO:0097006 | regulation of plasma lipoprotein particle levels | 7/219 | 101/18670 | 0.000187745 | 0.010037136 | 0.009060406 | ALB/APOA2/APOA1/APOA4/ADIPOQ/APOC3/APOB | 7 |
| GO:0000244 | BP | GO:0000244 | spliceosomal tri-snRNP complex assembly | 4/219 | 26/18670 | 0.000224851 | 0.01157564 | 0.010449195 | RNU4-2/RNU5B-1/RNU5E-1/RNU5A-1 | 4 |
| GO:0034114 | BP | GO:0034114 | regulation of heterotypic cell-cell adhesion | 4/219 | 26/18670 | 0.000224851 | 0.01157564 | 0.010449195 | APOA1/FGB/ADIPOQ/FGG | 4 |
| GO:0006641 | BP | GO:0006641 | triglyceride metabolic process | 7/219 | 105/18670 | 0.000238833 | 0.01207194 | 0.0108972 | GPAT2/APOA2/APOA1/APOA4/G6PC/APOC3/APOB | 7 |
| GO:0051673 | BP | GO:0051673 | membrane disruption in other organism | 3/219 | 11/18670 | 0.00024505 | 0.012165003 | 0.010981207 | LTF/DEFA5/DEFA6 | 3 |
| GO:0001964 | BP | GO:0001964 | startle response | 4/219 | 27/18670 | 0.000261542 | 0.012755888 | 0.011514592 | CTNNA2/CSMD1/NRXN1/GRIN2A | 4 |
| GO:0030534 | BP | GO:0030534 | adult behavior | 8/219 | 144/18670 | 0.000302929 | 0.014519702 | 0.013106767 | LEP/OPRD1/SPTBN4/PAX5/NRXN1/EN1/CRHR1/GBX1 | 8 |
| GO:0021903 | BP | GO:0021903 | rostrocaudal neural tube patterning | 3/219 | 12/18670 | 0.000323911 | 0.015262235 | 0.013777042 | GBX2/WNT1/EN1 | 3 |
| GO:0015918 | BP | GO:0015918 | sterol transport | 7/219 | 112/18670 | 0.000354797 | 0.01643893 | 0.014839231 | APOA2/LEP/APOA1/APOA4/ADIPOQ/APOC3/APOB | 7 |
| GO:0019216 | BP | GO:0019216 | regulation of lipid metabolic process | 14/219 | 410/18670 | 0.000361559 | 0.016477628 | 0.014874163 | APOA2/LEP/CCL19/CYP7A1/ACADL/APOA1/LGALS12/APOA4/ADIPOQ/SERPINA3/APOC3/APOB/EPHA8/PSAPL1 | 14 |
| GO:0098742 | BP | GO:0098742 | cell-cell adhesion via plasma-membrane adhesion molecules | 11/219 | 273/18670 | 0.000395551 | 0.017532308 | 0.015826211 | PCDH19/CDH16/SLITRK2/APOA1/NTNG1/CADM3/NRXN1/ADIPOQ/CLDN18/GATA5/PCDHB1 | 11 |
| GO:0045940 | BP | GO:0045940 | positive regulation of steroid metabolic process | 4/219 | 30/18670 | 0.000397315 | 0.017532308 | 0.015826211 | APOA2/CYP7A1/APOA1/APOA4 | 4 |
| GO:0010872 | BP | GO:0010872 | regulation of cholesterol esterification | 3/219 | 13/18670 | 0.000417448 | 0.018132879 | 0.016368339 | APOA2/APOA1/APOA4 | 3 |
| GO:0071695 | BP | GO:0071695 | anatomical structure maturation | 10/219 | 232/18670 | 0.000428289 | 0.01831759 | 0.016535075 | LTF/LEP/CCL19/SPTBN4/IL21/WNT1/MYOC/FGG/EPHA8/HBZ | 10 |
| GO:0042304 | BP | GO:0042304 | regulation of fatty acid biosynthetic process | 5/219 | 55/18670 | 0.000458087 | 0.019295179 | 0.017417534 | CYP7A1/ACADL/APOA4/ADIPOQ/APOC3 | 5 |
| GO:1905952 | BP | GO:1905952 | regulation of lipid localization | 8/219 | 156/18670 | 0.000517895 | 0.021488794 | 0.019397684 | APOA2/LEP/APOA1/APOA4/ADIPOQ/APOC3/APOB/CRHR1 | 8 |
| GO:0042743 | BP | GO:0042743 | hydrogen peroxide metabolic process | 5/219 | 57/18670 | 0.000541006 | 0.022117602 | 0.019965303 | APOA4/HBG2/HBQ1/HBZ/HBE1 | 5 |
| GO:0021700 | BP | GO:0021700 | developmental maturation | 11/219 | 284/18670 | 0.000550628 | 0.022184705 | 0.020025875 | LTF/LEP/CCL19/SPTBN4/IL21/NRXN1/WNT1/MYOC/FGG/EPHA8/HBZ | 11 |
| GO:0017001 | BP | GO:0017001 | antibiotic catabolic process | 5/219 | 58/18670 | 0.00058646 | 0.022639427 | 0.020436347 | APOA4/HBG2/HBQ1/HBZ/HBE1 | 5 |
| GO:0016042 | BP | GO:0016042 | lipid catabolic process | 12/219 | 333/18670 | 0.000591671 | 0.022639427 | 0.020436347 | APOA2/LEP/CYP1B1/CYP7A1/ACADL/APOA1/LGALS12/APOA4/ADIPOQ/APOC3/APOB/FGF23 | 12 |
| GO:0016999 | BP | GO:0016999 | antibiotic metabolic process | 7/219 | 122/18670 | 0.000594489 | 0.022639427 | 0.020436347 | AKR1C2/APOA4/HBG2/ADH1B/HBQ1/HBZ/HBE1 | 7 |
| GO:0019730 | BP | GO:0019730 | antimicrobial humoral response | 7/219 | 122/18670 | 0.000594489 | 0.022639427 | 0.020436347 | LTF/DEFA5/BPIFB2/PRSS2/FGB/DEFA6/DEFB4A | 7 |
| GO:0048871 | BP | GO:0048871 | multicellular organismal homeostasis | 15/219 | 486/18670 | 0.00064465 | 0.023961449 | 0.021629721 | SFTPA1/LTF/ALB/LEP/COL2A1/EBF2/MUC6/TP63/ACADL/CST4/ADIPOQ/CLDN18/AQP4/SERPINA3/RHAG | 15 |
| GO:0034380 | BP | GO:0034380 | high-density lipoprotein particle assembly | 3/219 | 15/18670 | 0.00065271 | 0.023961449 | 0.021629721 | APOA2/APOA1/APOA4 | 3 |
| GO:0019218 | BP | GO:0019218 | regulation of steroid metabolic process | 7/219 | 124/18670 | 0.000655061 | 0.023961449 | 0.021629721 | APOA2/LEP/CYP7A1/ACADL/APOA1/APOA4/APOB | 7 |
| GO:0032368 | BP | GO:0032368 | regulation of lipid transport | 7/219 | 125/18670 | 0.000687129 | 0.024808051 | 0.02239394 | APOA2/LEP/APOA1/APOA4/ADIPOQ/APOC3/CRHR1 | 7 |
| GO:0019433 | BP | GO:0019433 | triglyceride catabolic process | 4/219 | 35/18670 | 0.000725109 | 0.025843622 | 0.023328738 | APOA1/APOA4/APOC3/APOB | 4 |
| GO:0015711 | BP | GO:0015711 | organic anion transport | 15/219 | 495/18670 | 0.000777033 | 0.027083523 | 0.024447981 | APOA2/LEP/CA6/SLC26A9/SLC10A2/APOA1/SLC7A10/APOA4/G6PC/HRH3/LRP2/SLC2A2/APOC3/SLC5A8/RHAG | 15 |
| GO:0016125 | BP | GO:0016125 | sterol metabolic process | 8/219 | 166/18670 | 0.000779382 | 0.027083523 | 0.024447981 | APOA2/LEP/CYP1B1/CYP7A1/ACADL/APOA1/APOA4/APOB | 8 |
| GO:0002385 | BP | GO:0002385 | mucosal immune response | 4/219 | 36/18670 | 0.000808314 | 0.027742121 | 0.02504249 | LTF/DEFA5/APOA4/DEFA6 | 4 |
| GO:0071542 | BP | GO:0071542 | dopaminergic neuron differentiation | 4/219 | 37/18670 | 0.000898035 | 0.030078766 | 0.027151753 | LMX1B/WNT1/PITX3/EN1 | 4 |
| GO:1900026 | BP | GO:1900026 | positive regulation of substrate adhesion-dependent cell spreading | 4/219 | 37/18670 | 0.000898035 | 0.030078766 | 0.027151753 | APOA1/FGB/MYOC/FGG | 4 |
| GO:0046503 | BP | GO:0046503 | glycerolipid catabolic process | 5/219 | 64/18670 | 0.00092196 | 0.030287327 | 0.027340018 | APOA2/APOA1/APOA4/APOC3/APOB | 5 |
| GO:0034384 | BP | GO:0034384 | high-density lipoprotein particle clearance | 3/219 | 17/18670 | 0.000958736 | 0.030287327 | 0.027340018 | APOA2/APOA1/APOC3 | 3 |
| GO:0034433 | BP | GO:0034433 | steroid esterification | 3/219 | 17/18670 | 0.000958736 | 0.030287327 | 0.027340018 | APOA2/APOA1/APOA4 | 3 |
| GO:0034434 | BP | GO:0034434 | sterol esterification | 3/219 | 17/18670 | 0.000958736 | 0.030287327 | 0.027340018 | APOA2/APOA1/APOA4 | 3 |
| GO:0034435 | BP | GO:0034435 | cholesterol esterification | 3/219 | 17/18670 | 0.000958736 | 0.030287327 | 0.027340018 | APOA2/APOA1/APOA4 | 3 |
| GO:0051187 | BP | GO:0051187 | cofactor catabolic process | 5/219 | 65/18670 | 0.000989386 | 0.030904418 | 0.027897059 | APOA4/HBG2/HBQ1/HBZ/HBE1 | 5 |
| GO:0060191 | BP | GO:0060191 | regulation of lipase activity | 6/219 | 98/18670 | 0.001044116 | 0.032251569 | 0.029113116 | APOA2/ADCYAP1R1/APOA1/APOA4/ANXA8/APOC3 | 6 |
| GO:0044242 | BP | GO:0044242 | cellular lipid catabolic process | 9/219 | 217/18670 | 0.001082789 | 0.033078603 | 0.02985967 | APOA2/LEP/CYP1B1/ACADL/APOA1/APOA4/ADIPOQ/APOC3/APOB | 9 |
| GO:0002251 | BP | GO:0002251 | organ or tissue specific immune response | 4/219 | 39/18670 | 0.001098121 | 0.033182365 | 0.029953335 | LTF/DEFA5/APOA4/DEFA6 | 4 |
| GO:0044320 | BP | GO:0044320 | cellular response to leptin stimulus | 3/219 | 18/18670 | 0.001140575 | 0.034094608 | 0.030776806 | LEP/FGB/FGF23 | 3 |
| GO:0044058 | BP | GO:0044058 | regulation of digestive system process | 4/219 | 40/18670 | 0.001209033 | 0.035756497 | 0.032276974 | APOA2/LEP/APOA1/APOA4 | 4 |
| GO:0098869 | BP | GO:0098869 | cellular oxidant detoxification | 6/219 | 102/18670 | 0.001285024 | 0.037536387 | 0.03388366 | ALB/APOA4/HBG2/HBQ1/HBZ/HBE1 | 6 |
| GO:0034381 | BP | GO:0034381 | plasma lipoprotein particle clearance | 5/219 | 69/18670 | 0.001296221 | 0.037536387 | 0.03388366 | APOA2/APOA1/ADIPOQ/APOC3/APOB | 5 |
| GO:0006066 | BP | GO:0006066 | alcohol metabolic process | 12/219 | 370/18670 | 0.001466564 | 0.042031429 | 0.037941282 | AKR1C2/APOA2/LEP/CYP1B1/CYP7A1/ADCYAP1R1/ACADL/APOA1/APOA4/ADH1B/TTR/APOB | 12 |
| GO:0043950 | BP | GO:0043950 | positive regulation of cAMP-mediated signaling | 3/219 | 20/18670 | 0.001566147 | 0.044427432 | 0.040104127 | ADCYAP1R1/MRAP/CRHR1 | 3 |
| GO:0045834 | BP | GO:0045834 | positive regulation of lipid metabolic process | 7/219 | 146/18670 | 0.001699975 | 0.047736669 | 0.043091336 | APOA2/CCL19/CYP7A1/APOA1/APOA4/ADIPOQ/EPHA8 | 7 |
| GO:0006869 | BP | GO:0006869 | lipid transport | 12/219 | 380/18670 | 0.001833111 | 0.050960498 | 0.046001449 | SFTPA1/APOA2/LEP/SLC10A2/ABCA8/APOA1/APOA4/ADIPOQ/APOC3/APOB/SLC5A8/CRHR1 | 12 |
| GO:0008203 | BP | GO:0008203 | cholesterol metabolic process | 7/219 | 150/18670 | 0.001982927 | 0.054536815 | 0.049229749 | APOA2/LEP/CYP7A1/ACADL/APOA1/APOA4/APOB | 7 |
| GO:0019731 | BP | GO:0019731 | antibacterial humoral response | 4/219 | 46/18670 | 0.002043772 | 0.054536815 | 0.049229749 | LTF/DEFA5/FGB/DEFA6 | 4 |
| GO:0055088 | BP | GO:0055088 | lipid homeostasis | 7/219 | 151/18670 | 0.002059054 | 0.054536815 | 0.049229749 | APOA2/CYP7A1/APOA1/APOA4/G6PC/APOC3/APOB | 7 |
| GO:1990748 | BP | GO:1990748 | cellular detoxification | 6/219 | 112/18670 | 0.002073075 | 0.054536815 | 0.049229749 | ALB/APOA4/HBG2/HBQ1/HBZ/HBE1 | 6 |
| GO:0044321 | BP | GO:0044321 | response to leptin | 3/219 | 22/18670 | 0.002079461 | 0.054536815 | 0.049229749 | LEP/FGB/FGF23 | 3 |
| GO:0045717 | BP | GO:0045717 | negative regulation of fatty acid biosynthetic process | 3/219 | 22/18670 | 0.002079461 | 0.054536815 | 0.049229749 | CYP7A1/ACADL/APOC3 | 3 |
| GO:0007626 | BP | GO:0007626 | locomotory behavior | 8/219 | 198/18670 | 0.002390512 | 0.061533549 | 0.055545619 | GRM1/OPRD1/SPTBN4/TNR/PITX3/EN1/CRHR1/GBX1 | 8 |
| GO:0046890 | BP | GO:0046890 | regulation of lipid biosynthetic process | 8/219 | 198/18670 | 0.002390512 | 0.061533549 | 0.055545619 | LEP/CYP7A1/ACADL/APOA1/APOA4/ADIPOQ/APOC3/APOB | 8 |
| GO:0097237 | BP | GO:0097237 | cellular response to toxic substance | 9/219 | 247/18670 | 0.002626211 | 0.066980425 | 0.060462451 | ALB/CYP1B1/OPRD1/APOA4/HBG2/HBQ1/HBZ/HBE1/KCNC2 | 9 |
| GO:1902652 | BP | GO:1902652 | secondary alcohol metabolic process | 7/219 | 161/18670 | 0.002951036 | 0.074580735 | 0.067323163 | APOA2/LEP/CYP7A1/ACADL/APOA1/APOA4/APOB | 7 |
| GO:0030325 | BP | GO:0030325 | adrenal gland development | 3/219 | 25/18670 | 0.00302641 | 0.075119816 | 0.067809785 | APOA1/INSM1/CRHR1 | 3 |
| GO:0051953 | BP | GO:0051953 | negative regulation of amine transport | 3/219 | 25/18670 | 0.00302641 | 0.075119816 | 0.067809785 | LEP/HRH3/CRHR1 | 3 |
| GO:0050885 | BP | GO:0050885 | neuromuscular process controlling balance | 4/219 | 52/18670 | 0.00321032 | 0.078286757 | 0.070668545 | HMX3/TNR/NRXN1/IGDCC3 | 4 |
| GO:1900024 | BP | GO:1900024 | regulation of substrate adhesion-dependent cell spreading | 4/219 | 52/18670 | 0.00321032 | 0.078286757 | 0.070668545 | APOA1/FGB/MYOC/FGG | 4 |
| GO:0099560 | BP | GO:0099560 | synaptic membrane adhesion | 3/219 | 26/18670 | 0.003391831 | 0.081759296 | 0.073803166 | SLITRK2/NTNG1/NRXN1 | 3 |
| GO:0000387 | BP | GO:0000387 | spliceosomal snRNP assembly | 4/219 | 53/18670 | 0.003440949 | 0.081759296 | 0.073803166 | RNU4-2/RNU5B-1/RNU5E-1/RNU5A-1 | 4 |
| GO:0050704 | BP | GO:0050704 | regulation of interleukin-1 secretion | 4/219 | 53/18670 | 0.003440949 | 0.081759296 | 0.073803166 | PANX2/ORM2/CCL19/APOA1 | 4 |
| GO:0050994 | BP | GO:0050994 | regulation of lipid catabolic process | 4/219 | 54/18670 | 0.003682592 | 0.086110295 | 0.077730762 | APOA2/LGALS12/APOA4/APOC3 | 4 |
| GO:0043687 | BP | GO:0043687 | post-translational protein modification | 11/219 | 361/18670 | 0.003686016 | 0.086110295 | 0.077730762 | AFP/ALB/APOA2/AHSG/BPIFB2/APOA1/APOB/FGG/SERPINA10/ASB5/FGF23 | 11 |
| GO:0010876 | BP | GO:0010876 | lipid localization | 12/219 | 415/18670 | 0.003753609 | 0.086958598 | 0.078496516 | SFTPA1/APOA2/LEP/SLC10A2/ABCA8/APOA1/APOA4/ADIPOQ/APOC3/APOB/SLC5A8/CRHR1 | 12 |
| GO:0042157 | BP | GO:0042157 | lipoprotein metabolic process | 6/219 | 130/18670 | 0.004345743 | 0.099844348 | 0.090128333 | APOA2/LEP/APOA1/APOA4/APOC3/APOB | 6 |
| GO:0051897 | BP | GO:0051897 | positive regulation of protein kinase B signaling | 7/219 | 176/18670 | 0.004809866 | 0.109601854 | 0.09893632 | AKR1C2/IGF2/LEP/CCL19/MYOC/LIN28A/FGF23 | 7 |
| GO:0048469 | BP | GO:0048469 | cell maturation | 7/219 | 177/18670 | 0.004959329 | 0.112088899 | 0.101181346 | CCL19/SPTBN4/IL21/MYOC/FGG/EPHA8/HBZ | 7 |
| GO:0010586 | BP | GO:0010586 | miRNA metabolic process | 3/219 | 30/18670 | 0.005117432 | 0.114729535 | 0.103565017 | TRIM71/LIN28A/LIN28B | 3 |
| GO:0046677 | BP | GO:0046677 | response to antibiotic | 10/219 | 327/18670 | 0.005394371 | 0.11997082 | 0.108296265 | LEP/CYP1B1/CDO1/ADCYAP1R1/GRIN2A/ADIPOQ/CLDN18/CRHR1/KCNC2/SCGB1A1 | 10 |
| GO:0019217 | BP | GO:0019217 | regulation of fatty acid metabolic process | 5/219 | 96/18670 | 0.005467863 | 0.120184311 | 0.108488981 | CYP7A1/ACADL/APOA4/ADIPOQ/APOC3 | 5 |
| GO:0098754 | BP | GO:0098754 | detoxification | 6/219 | 137/18670 | 0.005600061 | 0.120184311 | 0.108488981 | ALB/APOA4/HBG2/HBQ1/HBZ/HBE1 | 6 |
| GO:0034694 | BP | GO:0034694 | response to prostaglandin | 3/219 | 31/18670 | 0.005617299 | 0.120184311 | 0.108488981 | AKR1C2/CCL19/APOB | 3 |
| GO:0034113 | BP | GO:0034113 | heterotypic cell-cell adhesion | 4/219 | 61/18670 | 0.00570265 | 0.120184311 | 0.108488981 | APOA1/FGB/ADIPOQ/FGG | 4 |
| GO:0050701 | BP | GO:0050701 | interleukin-1 secretion | 4/219 | 61/18670 | 0.00570265 | 0.120184311 | 0.108488981 | PANX2/ORM2/CCL19/APOA1 | 4 |
| GO:0001894 | BP | GO:0001894 | tissue homeostasis | 8/219 | 229/18670 | 0.005745827 | 0.120184311 | 0.108488981 | SFTPA1/LTF/ALB/COL2A1/MUC6/CST4/CLDN18/SERPINA3 | 8 |
| GO:0010587 | BP | GO:0010587 | miRNA catabolic process | 2/219 | 10/18670 | 0.005793057 | 0.120184311 | 0.108488981 | LIN28A/LIN28B | 2 |
| GO:0034447 | BP | GO:0034447 | very-low-density lipoprotein particle clearance | 2/219 | 10/18670 | 0.005793057 | 0.120184311 | 0.108488981 | APOC3/APOB | 2 |
| GO:0051956 | BP | GO:0051956 | negative regulation of amino acid transport | 2/219 | 10/18670 | 0.005793057 | 0.120184311 | 0.108488981 | LEP/HRH3 | 2 |
| GO:0050764 | BP | GO:0050764 | regulation of phagocytosis | 5/219 | 98/18670 | 0.005962161 | 0.122776359 | 0.110828792 | SFTPA1/APOA2/AHSG/APOA1/ADIPOQ | 5 |
| GO:0031640 | BP | GO:0031640 | killing of cells of other organism | 4/219 | 62/18670 | 0.006040941 | 0.123483942 | 0.111467519 | LTF/DEFA5/DEFA6/DEFB4A | 4 |
| GO:0045922 | BP | GO:0045922 | negative regulation of fatty acid metabolic process | 3/219 | 32/18670 | 0.006145447 | 0.124703228 | 0.112568154 | CYP7A1/ACADL/APOC3 | 3 |
| GO:0042737 | BP | GO:0042737 | drug catabolic process | 6/219 | 140/18670 | 0.006211523 | 0.125130673 | 0.112954004 | APOA4/HBG2/HBQ1/CPN1/HBZ/HBE1 | 6 |
| GO:0042445 | BP | GO:0042445 | hormone metabolic process | 8/219 | 233/18670 | 0.006358106 | 0.127162112 | 0.11478776 | AKR1C2/AFP/LEP/CYP1B1/APOA1/ADH1B/TTR/PCSK1 | 8 |
| GO:0034115 | BP | GO:0034115 | negative regulation of heterotypic cell-cell adhesion | 2/219 | 11/18670 | 0.007025947 | 0.13563981 | 0.12244048 | APOA1/ADIPOQ | 2 |
| GO:0034379 | BP | GO:0034379 | very-low-density lipoprotein particle assembly | 2/219 | 11/18670 | 0.007025947 | 0.13563981 | 0.12244048 | APOC3/APOB | 2 |
| GO:0070587 | BP | GO:0070587 | regulation of cell-cell adhesion involved in gastrulation | 2/219 | 11/18670 | 0.007025947 | 0.13563981 | 0.12244048 | APOA1/ADIPOQ | 2 |
| GO:1903011 | BP | GO:1903011 | negative regulation of bone development | 2/219 | 11/18670 | 0.007025947 | 0.13563981 | 0.12244048 | LTF/CLDN18 | 2 |
| GO:2001204 | BP | GO:2001204 | regulation of osteoclast development | 2/219 | 11/18670 | 0.007025947 | 0.13563981 | 0.12244048 | LTF/CLDN18 | 2 |
| GO:0007605 | BP | GO:0007605 | sensory perception of sound | 6/219 | 145/18670 | 0.007336581 | 0.140659966 | 0.126972117 | OTOF/COL2A1/SPTBN4/CNTN5/LRP2/PAX3 | 6 |
| GO:0001101 | BP | GO:0001101 | response to acid chemical | 10/219 | 343/18670 | 0.007458256 | 0.141390983 | 0.127631997 | AKR1C2/LEP/CCL19/CDO1/FZD10/ADIPOQ/BRINP2/GPRC6A/APOB/SCGB1A1 | 10 |
| GO:0019748 | BP | GO:0019748 | secondary metabolic process | 4/219 | 66/18670 | 0.007527146 | 0.141390983 | 0.127631997 | AKR1C2/CYP1B1/TYR/FMO2 | 4 |
| GO:0001503 | BP | GO:0001503 | ossification | 11/219 | 398/18670 | 0.007531957 | 0.141390983 | 0.127631997 | IGF2/LTF/LEP/COL2A1/TP63/AHSG/SOST/GDF10/MYOC/CLEC3A/FGF23 | 11 |
| GO:0002224 | BP | GO:0002224 | toll-like receptor signaling pathway | 6/219 | 146/18670 | 0.00757815 | 0.141390983 | 0.127631997 | SFTPA1/LTF/FGB/TREML4/APOB/FGG | 6 |
| GO:0022407 | BP | GO:0022407 | regulation of cell-cell adhesion | 11/219 | 402/18670 | 0.008087237 | 0.148143088 | 0.133727044 | IGF2/LEP/CCL19/APOA1/IL21/TNR/FGB/WNT1/ADIPOQ/FGG/SCGB1A1 | 11 |
| GO:0010896 | BP | GO:0010896 | regulation of triglyceride catabolic process | 2/219 | 12/18670 | 0.008366354 | 0.148143088 | 0.133727044 | APOA4/APOC3 | 2 |
| GO:0019755 | BP | GO:0019755 | one-carbon compound transport | 2/219 | 12/18670 | 0.008366354 | 0.148143088 | 0.133727044 | AQP5/RHAG | 2 |
| GO:0035630 | BP | GO:0035630 | bone mineralization involved in bone maturation | 2/219 | 12/18670 | 0.008366354 | 0.148143088 | 0.133727044 | LTF/LEP | 2 |
| GO:0045161 | BP | GO:0045161 | neuronal ion channel clustering | 2/219 | 12/18670 | 0.008366354 | 0.148143088 | 0.133727044 | SPTBN4/MYOC | 2 |
| GO:0060742 | BP | GO:0060742 | epithelial cell differentiation involved in prostate gland development | 2/219 | 12/18670 | 0.008366354 | 0.148143088 | 0.133727044 | TP63/PSAPL1 | 2 |
| GO:0070586 | BP | GO:0070586 | cell-cell adhesion involved in gastrulation | 2/219 | 12/18670 | 0.008366354 | 0.148143088 | 0.133727044 | APOA1/ADIPOQ | 2 |
| GO:0071107 | BP | GO:0071107 | response to parathyroid hormone | 2/219 | 12/18670 | 0.008366354 | 0.148143088 | 0.133727044 | SOST/FGF23 | 2 |
| GO:0061351 | BP | GO:0061351 | neural precursor cell proliferation | 6/219 | 150/18670 | 0.008602053 | 0.151352575 | 0.13662421 | GBX2/INSM1/WNT1/TRIM71/LRP2/PITX3 | 6 |
| GO:0051346 | BP | GO:0051346 | negative regulation of hydrolase activity | 12/219 | 463/18670 | 0.008729255 | 0.152624718 | 0.137772559 | LTF/APOA2/NGFR/AHSG/APOA1/CST4/ANXA8/FZD10/SERPINA3/APOC3/SERPINA10/SERPINB13 | 12 |
| GO:0050766 | BP | GO:0050766 | positive regulation of phagocytosis | 4/219 | 69/18670 | 0.008787203 | 0.152677644 | 0.137820334 | SFTPA1/APOA2/AHSG/APOA1 | 4 |
| GO:0030902 | BP | GO:0030902 | hindbrain development | 6/219 | 152/18670 | 0.009149594 | 0.157986774 | 0.142612824 | GBX2/CTNNA2/WNT1/NEUROG3/EN1/GBX1 | 6 |
| GO:0061515 | BP | GO:0061515 | myeloid cell development | 4/219 | 70/18670 | 0.009235954 | 0.158493529 | 0.143070267 | LTF/CLDN18/RHAG/HBZ | 4 |
| GO:0010951 | BP | GO:0010951 | negative regulation of endopeptidase activity | 8/219 | 250/18670 | 0.009527496 | 0.162358785 | 0.146559388 | LTF/NGFR/AHSG/CST4/ANXA8/SERPINA3/SERPINA10/SERPINB13 | 8 |
| GO:0018206 | BP | GO:0018206 | peptidyl-methionine modification | 2/219 | 13/18670 | 0.00981161 | 0.162358785 | 0.146559388 | APOA2/APOA1 | 2 |
| GO:0032488 | BP | GO:0032488 | Cdc42 protein signal transduction | 2/219 | 13/18670 | 0.00981161 | 0.162358785 | 0.146559388 | APOA1/APOC3 | 2 |
| GO:0034374 | BP | GO:0034374 | low-density lipoprotein particle remodeling | 2/219 | 13/18670 | 0.00981161 | 0.162358785 | 0.146559388 | APOA2/APOB | 2 |
| GO:0035641 | BP | GO:0035641 | locomotory exploration behavior | 2/219 | 13/18670 | 0.00981161 | 0.162358785 | 0.146559388 | TNR/CRHR1 | 2 |
| GO:1990403 | BP | GO:1990403 | embryonic brain development | 2/219 | 13/18670 | 0.00981161 | 0.162358785 | 0.146559388 | WNT1/EN1 | 2 |
| GO:0050716 | BP | GO:0050716 | positive regulation of interleukin-1 secretion | 3/219 | 38/18670 | 0.009928832 | 0.163326356 | 0.147432803 | PANX2/ORM2/CCL19 | 3 |
| GO:0051180 | BP | GO:0051180 | vitamin transport | 3/219 | 39/18670 | 0.010664725 | 0.171475517 | 0.154788956 | APOA1/LRP2/SLC2A2 | 3 |
| GO:0055090 | BP | GO:0055090 | acylglycerol homeostasis | 3/219 | 39/18670 | 0.010664725 | 0.171475517 | 0.154788956 | APOA1/APOA4/APOC3 | 3 |
| GO:0070328 | BP | GO:0070328 | triglyceride homeostasis | 3/219 | 39/18670 | 0.010664725 | 0.171475517 | 0.154788956 | APOA1/APOA4/APOC3 | 3 |
| GO:0061844 | BP | GO:0061844 | antimicrobial humoral immune response mediated by antimicrobial peptide | 4/219 | 73/18670 | 0.010670958 | 0.171475517 | 0.154788956 | LTF/DEFA5/DEFA6/DEFB4A | 4 |
| GO:0048266 | BP | GO:0048266 | behavioral response to pain | 2/219 | 14/18670 | 0.011359093 | 0.17952708 | 0.162057008 | THBS4/CRHR1 | 2 |
| GO:1901386 | BP | GO:1901386 | negative regulation of voltage-gated calcium channel activity | 2/219 | 14/18670 | 0.011359093 | 0.17952708 | 0.162057008 | RRAD/CRHR1 | 2 |
| GO:0021532 | BP | GO:0021532 | neural tube patterning | 3/219 | 40/18670 | 0.011431403 | 0.17952708 | 0.162057008 | GBX2/WNT1/EN1 | 3 |
| GO:0050832 | BP | GO:0050832 | defense response to fungus | 3/219 | 40/18670 | 0.011431403 | 0.17952708 | 0.162057008 | LTF/DEFA5/DEFA6 | 3 |
| GO:0046660 | BP | GO:0046660 | female sex differentiation | 5/219 | 115/18670 | 0.011494899 | 0.17952708 | 0.162057008 | AFP/LEP/TP63/ADCYAP1R1/LRP2 | 5 |
| GO:0006695 | BP | GO:0006695 | cholesterol biosynthetic process | 4/219 | 75/18670 | 0.011703309 | 0.180751111 | 0.163161927 | CYP7A1/APOA1/APOA4/APOB | 4 |
| GO:0021536 | BP | GO:0021536 | diencephalon development | 4/219 | 75/18670 | 0.011703309 | 0.180751111 | 0.163161927 | GBX2/WNT1/CRHR1/KCNC2 | 4 |
| GO:0042572 | BP | GO:0042572 | retinol metabolic process | 3/219 | 41/18670 | 0.012229075 | 0.185981725 | 0.167883541 | CYP1B1/ADH1B/TTR | 3 |
| GO:0050873 | BP | GO:0050873 | brown fat cell differentiation | 3/219 | 41/18670 | 0.012229075 | 0.185981725 | 0.167883541 | LEP/EBF2/ADIPOQ | 3 |
| GO:1902653 | BP | GO:1902653 | secondary alcohol biosynthetic process | 4/219 | 76/18670 | 0.012242682 | 0.185981725 | 0.167883541 | CYP7A1/APOA1/APOA4/APOB | 4 |
| GO:0010466 | BP | GO:0010466 | negative regulation of peptidase activity | 8/219 | 262/18670 | 0.012387308 | 0.186144412 | 0.168030396 | LTF/NGFR/AHSG/CST4/ANXA8/SERPINA3/SERPINA10/SERPINB13 | 8 |
| GO:0046883 | BP | GO:0046883 | regulation of hormone secretion | 8/219 | 262/18670 | 0.012387308 | 0.186144412 | 0.168030396 | LEP/FGB/ADIPOQ/SLC2A2/FGG/CRHR1/KCNC2/FGF23 | 8 |
| GO:1903531 | BP | GO:1903531 | negative regulation of secretion by cell | 7/219 | 212/18670 | 0.012733631 | 0.186159215 | 0.16804376 | APOA2/LEP/APOA1/HRH3/ADIPOQ/CRHR1/FGF23 | 7 |
| GO:0009914 | BP | GO:0009914 | hormone transport | 9/219 | 317/18670 | 0.012780008 | 0.186159215 | 0.16804376 | LEP/FGB/ADIPOQ/SLC2A2/TTR/FGG/CRHR1/KCNC2/FGF23 | 9 |
| GO:0008344 | BP | GO:0008344 | adult locomotory behavior | 4/219 | 77/18670 | 0.012797727 | 0.186159215 | 0.16804376 | OPRD1/SPTBN4/EN1/GBX1 | 4 |
| GO:0003128 | BP | GO:0003128 | heart field specification | 2/219 | 15/18670 | 0.013006229 | 0.186159215 | 0.16804376 | LRP2/GATA5 | 2 |
| GO:0018158 | BP | GO:0018158 | protein oxidation | 2/219 | 15/18670 | 0.013006229 | 0.186159215 | 0.16804376 | APOA2/APOA1 | 2 |
| GO:0031054 | BP | GO:0031054 | pre-miRNA processing | 2/219 | 15/18670 | 0.013006229 | 0.186159215 | 0.16804376 | LIN28A/LIN28B | 2 |
| GO:0044406 | BP | GO:0044406 | adhesion of symbiont to host | 2/219 | 15/18670 | 0.013006229 | 0.186159215 | 0.16804376 | LTF/CLEC4M | 2 |
| GO:0060134 | BP | GO:0060134 | prepulse inhibition | 2/219 | 15/18670 | 0.013006229 | 0.186159215 | 0.16804376 | CTNNA2/NRXN1 | 2 |
| GO:0090136 | BP | GO:0090136 | epithelial cell-cell adhesion | 2/219 | 15/18670 | 0.013006229 | 0.186159215 | 0.16804376 | CYP1B1/THBS4 | 2 |
| GO:0015701 | BP | GO:0015701 | bicarbonate transport | 3/219 | 42/18670 | 0.013057931 | 0.186159215 | 0.16804376 | CA6/SLC26A9/RHAG | 3 |
| GO:0050954 | BP | GO:0050954 | sensory perception of mechanical stimulus | 6/219 | 165/18670 | 0.013330546 | 0.189076116 | 0.170676812 | OTOF/COL2A1/SPTBN4/CNTN5/LRP2/PAX3 | 6 |
| GO:1901617 | BP | GO:1901617 | organic hydroxy compound biosynthetic process | 8/219 | 266/18670 | 0.013467843 | 0.190053831 | 0.171559384 | LEP/CYP7A1/ADCYAP1R1/TYR/APOA1/APOA4/INSM1/APOB | 8 |
| GO:0034383 | BP | GO:0034383 | low-density lipoprotein particle clearance | 3/219 | 43/18670 | 0.013918137 | 0.19443428 | 0.175513565 | ADIPOQ/APOC3/APOB | 3 |
| GO:0099054 | BP | GO:0099054 | presynapse assembly | 3/219 | 43/18670 | 0.013918137 | 0.19443428 | 0.175513565 | CNTN5/SLITRK2/NRXN1 | 3 |
| GO:0015748 | BP | GO:0015748 | organophosphate ester transport | 5/219 | 121/18670 | 0.014079837 | 0.195709738 | 0.176664905 | APOA2/APOA1/APOA4/G6PC/APOC3 | 5 |
| GO:0001906 | BP | GO:0001906 | cell killing | 6/219 | 168/18670 | 0.014458308 | 0.199059998 | 0.179689146 | LTF/LEP/DEFA5/IL21/DEFA6/DEFB4A | 6 |
| GO:0002739 | BP | GO:0002739 | regulation of cytokine secretion involved in immune response | 2/219 | 16/18670 | 0.014750489 | 0.199059998 | 0.179689146 | APOA2/APOA1 | 2 |
| GO:0007158 | BP | GO:0007158 | neuron cell-cell adhesion | 2/219 | 16/18670 | 0.014750489 | 0.199059998 | 0.179689146 | TNR/NRXN1 | 2 |
| GO:0010875 | BP | GO:0010875 | positive regulation of cholesterol efflux | 2/219 | 16/18670 | 0.014750489 | 0.199059998 | 0.179689146 | APOA1/ADIPOQ | 2 |
| GO:0034116 | BP | GO:0034116 | positive regulation of heterotypic cell-cell adhesion | 2/219 | 16/18670 | 0.014750489 | 0.199059998 | 0.179689146 | FGB/FGG | 2 |
| GO:0042448 | BP | GO:0042448 | progesterone metabolic process | 2/219 | 16/18670 | 0.014750489 | 0.199059998 | 0.179689146 | AKR1C2/AFP | 2 |
| GO:0016126 | BP | GO:0016126 | sterol biosynthetic process | 4/219 | 81/18670 | 0.015177577 | 0.20383413 | 0.1839987 | CYP7A1/APOA1/APOA4/APOB | 4 |
| GO:0048483 | BP | GO:0048483 | autonomic nervous system development | 3/219 | 45/18670 | 0.015733179 | 0.21027999 | 0.189817303 | GBX2/TP63/INSM1 | 3 |
| GO:0030279 | BP | GO:0030279 | negative regulation of ossification | 4/219 | 82/18670 | 0.01581315 | 0.210337595 | 0.189869302 | AHSG/SOST/GDF10/FGF23 | 4 |
| GO:0045444 | BP | GO:0045444 | fat cell differentiation | 7/219 | 223/18670 | 0.016393882 | 0.211841764 | 0.191227098 | LEP/EBF2/LGALS12/WNT1/ADIPOQ/GDF10/PTPRQ | 7 |
| GO:0007411 | BP | GO:0007411 | axon guidance | 8/219 | 276/18670 | 0.016469143 | 0.211841764 | 0.191227098 | DPYSL5/GBX2/SPTBN4/NTNG1/TNR/NRXN1/EPHA8/GBX1 | 8 |
| GO:0000097 | BP | GO:0000097 | sulfur amino acid biosynthetic process | 2/219 | 17/18670 | 0.01658939 | 0.211841764 | 0.191227098 | BHMT2/CDO1 | 2 |
| GO:0006883 | BP | GO:0006883 | cellular sodium ion homeostasis | 2/219 | 17/18670 | 0.01658939 | 0.211841764 | 0.191227098 | ATP1A3/C7 | 2 |
| GO:0030540 | BP | GO:0030540 | female genitalia development | 2/219 | 17/18670 | 0.01658939 | 0.211841764 | 0.191227098 | TP63/LRP2 | 2 |
| GO:0032460 | BP | GO:0032460 | negative regulation of protein oligomerization | 2/219 | 17/18670 | 0.01658939 | 0.211841764 | 0.191227098 | ALB/OPRD1 | 2 |
| GO:0035196 | BP | GO:0035196 | production of miRNAs involved in gene silencing by miRNA | 3/219 | 46/18670 | 0.016688254 | 0.211841764 | 0.191227098 | TRIM71/LIN28A/LIN28B | 3 |
| GO:0050706 | BP | GO:0050706 | regulation of interleukin-1 beta secretion | 3/219 | 46/18670 | 0.016688254 | 0.211841764 | 0.191227098 | ORM2/CCL19/APOA1 | 3 |
| GO:0099172 | BP | GO:0099172 | presynapse organization | 3/219 | 46/18670 | 0.016688254 | 0.211841764 | 0.191227098 | CNTN5/SLITRK2/NRXN1 | 3 |
| GO:1904036 | BP | GO:1904036 | negative regulation of epithelial cell apoptotic process | 3/219 | 46/18670 | 0.016688254 | 0.211841764 | 0.191227098 | FGB/FGG/SERPINB13 | 3 |
| GO:0097485 | BP | GO:0097485 | neuron projection guidance | 8/219 | 277/18670 | 0.016793798 | 0.212212542 | 0.191561795 | DPYSL5/GBX2/SPTBN4/NTNG1/TNR/NRXN1/EPHA8/GBX1 | 8 |
| GO:0051186 | BP | GO:0051186 | cofactor metabolic process | 11/219 | 449/18670 | 0.017272154 | 0.2172008 | 0.196064638 | AKR1C2/PRSS1/BHMT2/NMNAT2/APOA4/HBG2/HBQ1/SLC5A8/HBZ/HBE1/GLYAT | 11 |
| GO:0046165 | BP | GO:0046165 | alcohol biosynthetic process | 6/219 | 175/18670 | 0.017344812 | 0.2172008 | 0.196064638 | LEP/CYP7A1/ADCYAP1R1/APOA1/APOA4/APOB | 6 |
| GO:0006953 | BP | GO:0006953 | acute-phase response | 3/219 | 47/18670 | 0.017675161 | 0.219361375 | 0.198014963 | ORM2/AHSG/SERPINA3 | 3 |
| GO:0042551 | BP | GO:0042551 | neuron maturation | 3/219 | 47/18670 | 0.017675161 | 0.219361375 | 0.198014963 | SPTBN4/MYOC/EPHA8 | 3 |
| GO:0034308 | BP | GO:0034308 | primary alcohol metabolic process | 4/219 | 85/18670 | 0.017819383 | 0.220168381 | 0.198743439 | AKR1C2/CYP1B1/ADH1B/TTR | 4 |
| GO:0052547 | BP | GO:0052547 | regulation of peptidase activity | 11/219 | 452/18670 | 0.018049488 | 0.221633255 | 0.200065763 | LTF/PCOLCE2/TP63/NGFR/AHSG/CST4/ANXA8/GRIN2A/SERPINA3/SERPINA10/SERPINB13 | 11 |
| GO:0034754 | BP | GO:0034754 | cellular hormone metabolic process | 5/219 | 129/18670 | 0.018097392 | 0.221633255 | 0.200065763 | AKR1C2/AFP/CYP1B1/ADH1B/TTR | 5 |
| GO:0036035 | BP | GO:0036035 | osteoclast development | 2/219 | 18/18670 | 0.018520491 | 0.223870877 | 0.202085638 | LTF/CLDN18 | 2 |
| GO:0045683 | BP | GO:0045683 | negative regulation of epidermis development | 2/219 | 18/18670 | 0.018520491 | 0.223870877 | 0.202085638 | TP63/DKK4 | 2 |
| GO:0070542 | BP | GO:0070542 | response to fatty acid | 4/219 | 86/18670 | 0.018521691 | 0.223870877 | 0.202085638 | AKR1C2/CCL19/ADIPOQ/APOB | 4 |
| GO:0002762 | BP | GO:0002762 | negative regulation of myeloid leukocyte differentiation | 3/219 | 48/18670 | 0.018693975 | 0.224005393 | 0.202207065 | LTF/ADIPOQ/CLDN18 | 3 |
| GO:0043949 | BP | GO:0043949 | regulation of cAMP-mediated signaling | 3/219 | 48/18670 | 0.018693975 | 0.224005393 | 0.202207065 | ADCYAP1R1/MRAP/CRHR1 | 3 |
| GO:0022408 | BP | GO:0022408 | negative regulation of cell-cell adhesion | 6/219 | 180/18670 | 0.019634352 | 0.232372035 | 0.209759535 | APOA1/TNR/WNT1/ADIPOQ/FGG/SCGB1A1 | 6 |
| GO:0031333 | BP | GO:0031333 | negative regulation of protein-containing complex assembly | 5/219 | 132/18670 | 0.019780402 | 0.232372035 | 0.209759535 | ALB/OPRD1/ADD2/SPTBN4/SOST | 5 |
| GO:0045598 | BP | GO:0045598 | regulation of fat cell differentiation | 5/219 | 132/18670 | 0.019780402 | 0.232372035 | 0.209759535 | LEP/LGALS12/WNT1/ADIPOQ/PTPRQ | 5 |
| GO:0034103 | BP | GO:0034103 | regulation of tissue remodeling | 4/219 | 88/18670 | 0.019977308 | 0.232372035 | 0.209759535 | LEP/THBS4/IL21/CLDN18 | 4 |
| GO:0045921 | BP | GO:0045921 | positive regulation of exocytosis | 4/219 | 88/18670 | 0.019977308 | 0.232372035 | 0.209759535 | FGB/FGG/CRHR1/SYT10 | 4 |
| GO:0050829 | BP | GO:0050829 | defense response to Gram-negative bacterium | 4/219 | 88/18670 | 0.019977308 | 0.232372035 | 0.209759535 | LTF/DEFA5/DEFA6/DEFB4A | 4 |
| GO:2000177 | BP | GO:2000177 | regulation of neural precursor cell proliferation | 4/219 | 88/18670 | 0.019977308 | 0.232372035 | 0.209759535 | INSM1/TRIM71/LRP2/PITX3 | 4 |
| GO:0006833 | BP | GO:0006833 | water transport | 2/219 | 19/18670 | 0.020541399 | 0.235000368 | 0.212132101 | AQP5/AQP4 | 2 |
| GO:0032026 | BP | GO:0032026 | response to magnesium ion | 2/219 | 19/18670 | 0.020541399 | 0.235000368 | 0.212132101 | KCNC2/FGF23 | 2 |
| GO:0071731 | BP | GO:0071731 | response to nitric oxide | 2/219 | 19/18670 | 0.020541399 | 0.235000368 | 0.212132101 | CCL19/KCNC2 | 2 |
| GO:1902074 | BP | GO:1902074 | response to salt | 2/219 | 19/18670 | 0.020541399 | 0.235000368 | 0.212132101 | PITX3/FGF23 | 2 |
| GO:0045104 | BP | GO:0045104 | intermediate filament cytoskeleton organization | 3/219 | 50/18670 | 0.02082754 | 0.237297384 | 0.21420559 | GFAP/KRT14/KRT3 | 3 |
| GO:0007162 | BP | GO:0007162 | negative regulation of cell adhesion | 8/219 | 289/18670 | 0.021057668 | 0.238940069 | 0.215688423 | CYP1B1/APOA1/TNR/WNT1/ADIPOQ/MYOC/FGG/SCGB1A1 | 8 |
| GO:0043255 | BP | GO:0043255 | regulation of carbohydrate biosynthetic process | 4/219 | 90/18670 | 0.021501593 | 0.242985479 | 0.219340168 | IGF2/LEP/ADCYAP1R1/ADIPOQ | 4 |
| GO:0031050 | BP | GO:0031050 | dsRNA processing | 3/219 | 51/18670 | 0.021942358 | 0.243999019 | 0.220255078 | TRIM71/LIN28A/LIN28B | 3 |
| GO:0045103 | BP | GO:0045103 | intermediate filament-based process | 3/219 | 51/18670 | 0.021942358 | 0.243999019 | 0.220255078 | GFAP/KRT14/KRT3 | 3 |
| GO:0070918 | BP | GO:0070918 | production of small RNA involved in gene silencing by RNA | 3/219 | 51/18670 | 0.021942358 | 0.243999019 | 0.220255078 | TRIM71/LIN28A/LIN28B | 3 |
| GO:0097366 | BP | GO:0097366 | response to bronchodilator | 3/219 | 51/18670 | 0.021942358 | 0.243999019 | 0.220255078 | CCL19/GRIN2A/KCNC2 | 3 |
| GO:0009410 | BP | GO:0009410 | response to xenobiotic stimulus | 8/219 | 292/18670 | 0.022233702 | 0.245005191 | 0.221163338 | CYP1B1/CDO1/FMO2/GRIN2A/PITX3/CRHR1/GLYAT/SCGB1A1 | 8 |
| GO:0015914 | BP | GO:0015914 | phospholipid transport | 4/219 | 91/18670 | 0.022289718 | 0.245005191 | 0.221163338 | APOA2/APOA1/APOA4/APOC3 | 4 |
| GO:0030258 | BP | GO:0030258 | lipid modification | 7/219 | 238/18670 | 0.022508871 | 0.245005191 | 0.221163338 | APOA2/LEP/ACADL/APOA1/APOA4/ADIPOQ/DGKK | 7 |
| GO:0051048 | BP | GO:0051048 | negative regulation of secretion | 7/219 | 238/18670 | 0.022508871 | 0.245005191 | 0.221163338 | APOA2/LEP/APOA1/HRH3/ADIPOQ/CRHR1/FGF23 | 7 |
| GO:0007409 | BP | GO:0007409 | axonogenesis | 11/219 | 468/18670 | 0.022642887 | 0.245005191 | 0.221163338 | DPYSL5/GBX2/CTNNA2/SPTBN4/NGFR/SLITRK2/NTNG1/TNR/NRXN1/EPHA8/GBX1 | 11 |
| GO:0002374 | BP | GO:0002374 | cytokine secretion involved in immune response | 2/219 | 20/18670 | 0.02264976 | 0.245005191 | 0.221163338 | APOA2/APOA1 | 2 |
| GO:0043931 | BP | GO:0043931 | ossification involved in bone maturation | 2/219 | 20/18670 | 0.02264976 | 0.245005191 | 0.221163338 | LTF/LEP | 2 |
| GO:0009620 | BP | GO:0009620 | response to fungus | 3/219 | 52/18670 | 0.023089217 | 0.245995703 | 0.222057461 | LTF/DEFA5/DEFA6 | 3 |
| GO:0030514 | BP | GO:0030514 | negative regulation of BMP signaling pathway | 3/219 | 52/18670 | 0.023089217 | 0.245995703 | 0.222057461 | WNT1/LRP2/SOST | 3 |
| GO:2000179 | BP | GO:2000179 | positive regulation of neural precursor cell proliferation | 3/219 | 52/18670 | 0.023089217 | 0.245995703 | 0.222057461 | INSM1/LRP2/PITX3 | 3 |
| GO:0045833 | BP | GO:0045833 | negative regulation of lipid metabolic process | 4/219 | 92/18670 | 0.02309528 | 0.245995703 | 0.222057461 | APOA2/CYP7A1/ACADL/APOC3 | 4 |
| GO:0042593 | BP | GO:0042593 | glucose homeostasis | 7/219 | 241/18670 | 0.023898554 | 0.252825238 | 0.228222403 | LEP/CYP7A1/NGFR/CSMD1/G6PC/ADIPOQ/LIN28A | 7 |
| GO:0051952 | BP | GO:0051952 | regulation of amine transport | 4/219 | 93/18670 | 0.023918359 | 0.252825238 | 0.228222403 | LEP/HRH3/CRHR1/SYT10 | 4 |
| GO:0031638 | BP | GO:0031638 | zymogen activation | 3/219 | 53/18670 | 0.024268113 | 0.253912786 | 0.229204121 | GRIN2A/FGB/FGG | 3 |
| GO:0050702 | BP | GO:0050702 | interleukin-1 beta secretion | 3/219 | 53/18670 | 0.024268113 | 0.253912786 | 0.229204121 | ORM2/CCL19/APOA1 | 3 |
| GO:0033500 | BP | GO:0033500 | carbohydrate homeostasis | 7/219 | 242/18670 | 0.024374632 | 0.253912786 | 0.229204121 | LEP/CYP7A1/NGFR/CSMD1/G6PC/ADIPOQ/LIN28A | 7 |
| GO:0051289 | BP | GO:0051289 | protein homotetramerization | 4/219 | 94/18670 | 0.02475903 | 0.253912786 | 0.229204121 | AQP5/TP63/ACADL/AQP4 | 4 |
| GO:0030277 | BP | GO:0030277 | maintenance of gastrointestinal epithelium | 2/219 | 21/18670 | 0.024843265 | 0.253912786 | 0.229204121 | MUC6/SERPINA3 | 2 |
| GO:0030502 | BP | GO:0030502 | negative regulation of bone mineralization | 2/219 | 21/18670 | 0.024843265 | 0.253912786 | 0.229204121 | AHSG/FGF23 | 2 |
| GO:0030728 | BP | GO:0030728 | ovulation | 2/219 | 21/18670 | 0.024843265 | 0.253912786 | 0.229204121 | AFP/LEP | 2 |
| GO:0032891 | BP | GO:0032891 | negative regulation of organic acid transport | 2/219 | 21/18670 | 0.024843265 | 0.253912786 | 0.229204121 | LEP/HRH3 | 2 |
| GO:0048485 | BP | GO:0048485 | sympathetic nervous system development | 2/219 | 21/18670 | 0.024843265 | 0.253912786 | 0.229204121 | TP63/INSM1 | 2 |
| GO:0051896 | BP | GO:0051896 | regulation of protein kinase B signaling | 7/219 | 244/18670 | 0.025346286 | 0.256173653 | 0.231244978 | AKR1C2/IGF2/LEP/CCL19/MYOC/LIN28A/FGF23 | 7 |
| GO:0062013 | BP | GO:0062013 | positive regulation of small molecule metabolic process | 5/219 | 141/18670 | 0.025434547 | 0.256173653 | 0.231244978 | IGF2/CYP7A1/ADCYAP1R1/APOA4/ADIPOQ | 5 |
| GO:0045747 | BP | GO:0045747 | positive regulation of Notch signaling pathway | 3/219 | 54/18670 | 0.025479026 | 0.256173653 | 0.231244978 | TP63/WNT1/GATA5 | 3 |
| GO:0051703 | BP | GO:0051703 | intraspecies interaction between organisms | 3/219 | 54/18670 | 0.025479026 | 0.256173653 | 0.231244978 | LTF/NRXN1/EN1 | 3 |
| GO:0042102 | BP | GO:0042102 | positive regulation of T cell proliferation | 4/219 | 95/18670 | 0.025617365 | 0.256173653 | 0.231244978 | IGF2/LEP/CCL19/IL21 | 4 |
| GO:0044070 | BP | GO:0044070 | regulation of anion transport | 4/219 | 95/18670 | 0.025617365 | 0.256173653 | 0.231244978 | LEP/APOA1/HRH3/FGF23 | 4 |
| GO:0045861 | BP | GO:0045861 | negative regulation of proteolysis | 9/219 | 358/18670 | 0.025816411 | 0.257238789 | 0.232206464 | LTF/NGFR/AHSG/CST4/ANXA8/WNT1/SERPINA3/SERPINA10/SERPINB13 | 9 |
| GO:1902904 | BP | GO:1902904 | negative regulation of supramolecular fiber organization | 5/219 | 142/18670 | 0.026120287 | 0.259337133 | 0.234100616 | CTNNA2/ADD2/SPTBN4/TRIM54/MYOC | 5 |
| GO:0042180 | BP | GO:0042180 | cellular ketone metabolic process | 7/219 | 246/18670 | 0.026344184 | 0.260629293 | 0.235267033 | AKR1C2/AFP/CYP7A1/ACADL/APOA4/ADIPOQ/APOC3 | 7 |
| GO:0042359 | BP | GO:0042359 | vitamin D metabolic process | 2/219 | 22/18670 | 0.027119646 | 0.264535493 | 0.238793114 | LRP2/FGF23 | 2 |
| GO:0046339 | BP | GO:0046339 | diacylglycerol metabolic process | 2/219 | 22/18670 | 0.027119646 | 0.264535493 | 0.238793114 | APOA2/DGKK | 2 |
| GO:0046628 | BP | GO:0046628 | positive regulation of insulin receptor signaling pathway | 2/219 | 22/18670 | 0.027119646 | 0.264535493 | 0.238793114 | IGF2/LEP | 2 |
| GO:0071379 | BP | GO:0071379 | cellular response to prostaglandin stimulus | 2/219 | 22/18670 | 0.027119646 | 0.264535493 | 0.238793114 | AKR1C2/APOB | 2 |
| GO:0120162 | BP | GO:0120162 | positive regulation of cold-induced thermogenesis | 4/219 | 97/18670 | 0.027387288 | 0.266212102 | 0.24030657 | LEP/EBF2/ACADL/ADIPOQ | 4 |
| GO:0030216 | BP | GO:0030216 | keratinocyte differentiation | 8/219 | 305/18670 | 0.0278687 | 0.269556431 | 0.243325457 | TP63/KRT14/LCE1E/KRTAP3-3/SERPINB13/KRT3/KRT24/SPRR2G | 8 |
| GO:0007612 | BP | GO:0007612 | learning | 5/219 | 145/18670 | 0.028248088 | 0.269556431 | 0.243325457 | TNR/NRXN1/GRIN2A/EN1/CRHR1 | 5 |
| GO:0042472 | BP | GO:0042472 | inner ear morphogenesis | 4/219 | 98/18670 | 0.028298996 | 0.269556431 | 0.243325457 | COL2A1/GBX2/HMX3/WNT1 | 4 |
| GO:0052548 | BP | GO:0052548 | regulation of endopeptidase activity | 10/219 | 425/18670 | 0.028837329 | 0.269556431 | 0.243325457 | LTF/TP63/NGFR/AHSG/CST4/ANXA8/GRIN2A/SERPINA3/SERPINA10/SERPINB13 | 10 |
| GO:0002221 | BP | GO:0002221 | pattern recognition receptor signaling pathway | 6/219 | 197/18670 | 0.02893806 | 0.269556431 | 0.243325457 | SFTPA1/LTF/FGB/TREML4/APOB/FGG | 6 |
| GO:0051384 | BP | GO:0051384 | response to glucocorticoid | 5/219 | 146/18670 | 0.028981065 | 0.269556431 | 0.243325457 | APOA2/CDO1/ADIPOQ/CPN1/SCGB1A1 | 5 |
| GO:0062014 | BP | GO:0062014 | negative regulation of small molecule metabolic process | 4/219 | 99/18670 | 0.029228606 | 0.269556431 | 0.243325457 | CYP7A1/ACADL/ADIPOQ/APOC3 | 4 |
| GO:0046879 | BP | GO:0046879 | hormone secretion | 8/219 | 308/18670 | 0.029297858 | 0.269556431 | 0.243325457 | LEP/FGB/ADIPOQ/SLC2A2/FGG/CRHR1/KCNC2/FGF23 | 8 |
| GO:0051055 | BP | GO:0051055 | negative regulation of lipid biosynthetic process | 3/219 | 57/18670 | 0.02930346 | 0.269556431 | 0.243325457 | CYP7A1/ACADL/APOC3 | 3 |
| GO:0032373 | BP | GO:0032373 | positive regulation of sterol transport | 2/219 | 23/18670 | 0.029476675 | 0.269556431 | 0.243325457 | APOA1/ADIPOQ | 2 |
| GO:0032376 | BP | GO:0032376 | positive regulation of cholesterol transport | 2/219 | 23/18670 | 0.029476675 | 0.269556431 | 0.243325457 | APOA1/ADIPOQ | 2 |
| GO:0034123 | BP | GO:0034123 | positive regulation of toll-like receptor signaling pathway | 2/219 | 23/18670 | 0.029476675 | 0.269556431 | 0.243325457 | LTF/TREML4 | 2 |
| GO:0045109 | BP | GO:0045109 | intermediate filament organization | 2/219 | 23/18670 | 0.029476675 | 0.269556431 | 0.243325457 | GFAP/KRT14 | 2 |
| GO:0050995 | BP | GO:0050995 | negative regulation of lipid catabolic process | 2/219 | 23/18670 | 0.029476675 | 0.269556431 | 0.243325457 | APOA2/APOC3 | 2 |
| GO:0051004 | BP | GO:0051004 | regulation of lipoprotein lipase activity | 2/219 | 23/18670 | 0.029476675 | 0.269556431 | 0.243325457 | APOA4/APOC3 | 2 |
| GO:0070977 | BP | GO:0070977 | bone maturation | 2/219 | 23/18670 | 0.029476675 | 0.269556431 | 0.243325457 | LTF/LEP | 2 |
| GO:0098743 | BP | GO:0098743 | cell aggregation | 2/219 | 23/18670 | 0.029476675 | 0.269556431 | 0.243325457 | LTF/COL2A1 | 2 |
| GO:2000637 | BP | GO:2000637 | positive regulation of gene silencing by miRNA | 2/219 | 23/18670 | 0.029476675 | 0.269556431 | 0.243325457 | TRIM71/LIN28A | 2 |
| GO:0015837 | BP | GO:0015837 | amine transport | 4/219 | 100/18670 | 0.030176169 | 0.274149505 | 0.247471572 | LEP/HRH3/CRHR1/SYT10 | 4 |
| GO:0034446 | BP | GO:0034446 | substrate adhesion-dependent cell spreading | 4/219 | 100/18670 | 0.030176169 | 0.274149505 | 0.247471572 | APOA1/FGB/MYOC/FGG | 4 |
| GO:0051494 | BP | GO:0051494 | negative regulation of cytoskeleton organization | 5/219 | 148/18670 | 0.03048289 | 0.276033987 | 0.249172671 | CTNNA2/ADD2/SPTBN4/TRIM54/MYOC | 5 |
| GO:0019229 | BP | GO:0019229 | regulation of vasoconstriction | 3/219 | 58/18670 | 0.030641968 | 0.276573606 | 0.249659779 | LEP/FGB/FGG | 3 |
| GO:0007631 | BP | GO:0007631 | feeding behavior | 4/219 | 101/18670 | 0.031141727 | 0.28017476 | 0.252910499 | LEP/OPRD1/EN1/CRHR1 | 4 |
| GO:0009404 | BP | GO:0009404 | toxin metabolic process | 2/219 | 24/18670 | 0.031912165 | 0.280737821 | 0.253418768 | CYP1B1/FMO2 | 2 |
| GO:0010804 | BP | GO:0010804 | negative regulation of tumor necrosis factor-mediated signaling pathway | 2/219 | 24/18670 | 0.031912165 | 0.280737821 | 0.253418768 | APOA1/ADIPOQ | 2 |
| GO:0050996 | BP | GO:0050996 | positive regulation of lipid catabolic process | 2/219 | 24/18670 | 0.031912165 | 0.280737821 | 0.253418768 | APOA2/APOA4 | 2 |
| GO:0060148 | BP | GO:0060148 | positive regulation of posttranscriptional gene silencing | 2/219 | 24/18670 | 0.031912165 | 0.280737821 | 0.253418768 | TRIM71/LIN28A | 2 |
| GO:0090023 | BP | GO:0090023 | positive regulation of neutrophil chemotaxis | 2/219 | 24/18670 | 0.031912165 | 0.280737821 | 0.253418768 | CCL19/THBS4 | 2 |
| GO:1900078 | BP | GO:1900078 | positive regulation of cellular response to insulin stimulus | 2/219 | 24/18670 | 0.031912165 | 0.280737821 | 0.253418768 | IGF2/LEP | 2 |
| GO:1903010 | BP | GO:1903010 | regulation of bone development | 2/219 | 24/18670 | 0.031912165 | 0.280737821 | 0.253418768 | LTF/CLDN18 | 2 |
| GO:0032732 | BP | GO:0032732 | positive regulation of interleukin-1 production | 3/219 | 59/18670 | 0.03201219 | 0.280737821 | 0.253418768 | PANX2/ORM2/CCL19 | 3 |
| GO:0021549 | BP | GO:0021549 | cerebellum development | 4/219 | 102/18670 | 0.032125321 | 0.280844003 | 0.253514617 | GBX2/WNT1/EN1/GBX1 | 4 |
| GO:0006898 | BP | GO:0006898 | receptor-mediated endocytosis | 8/219 | 314/18670 | 0.032306464 | 0.281542224 | 0.254144893 | ALB/CCL19/APOA1/LRP2/IGHV2-70/APOC3/APOB/SH3GL3 | 8 |
| GO:0030278 | BP | GO:0030278 | regulation of ossification | 6/219 | 203/18670 | 0.032813308 | 0.285065613 | 0.257325415 | LTF/TP63/AHSG/SOST/GDF10/FGF23 | 6 |
| GO:0032652 | BP | GO:0032652 | regulation of interleukin-1 production | 4/219 | 103/18670 | 0.033126987 | 0.286894158 | 0.258976021 | PANX2/ORM2/CCL19/APOA1 | 4 |
| GO:0002719 | BP | GO:0002719 | negative regulation of cytokine production involved in immune response | 2/219 | 25/18670 | 0.034423969 | 0.295366158 | 0.266623597 | APOA2/APOA1 | 2 |
| GO:0031639 | BP | GO:0031639 | plasminogen activation | 2/219 | 25/18670 | 0.034423969 | 0.295366158 | 0.266623597 | FGB/FGG | 2 |
| GO:0050901 | BP | GO:0050901 | leukocyte tethering or rolling | 2/219 | 25/18670 | 0.034423969 | 0.295366158 | 0.266623597 | LEP/ADD2 | 2 |
| GO:1905953 | BP | GO:1905953 | negative regulation of lipid localization | 3/219 | 61/18670 | 0.034847377 | 0.298079105 | 0.269072543 | APOA2/LEP/APOC3 | 3 |
| GO:0007204 | BP | GO:0007204 | positive regulation of cytosolic calcium ion concentration | 8/219 | 319/18670 | 0.034969962 | 0.298210105 | 0.269190795 | MS4A1/GRM1/CCL19/RIC3/ADCYAP1R1/CXCR5/GRIN2A/CRHR1 | 8 |
| GO:0008593 | BP | GO:0008593 | regulation of Notch signaling pathway | 4/219 | 105/18670 | 0.035184656 | 0.299007947 | 0.269910998 | TP63/WNT1/GATA5/MAGEA1 | 4 |
| GO:0010770 | BP | GO:0010770 | positive regulation of cell morphogenesis involved in differentiation | 5/219 | 154/18670 | 0.035278636 | 0.299007947 | 0.269910998 | NGFR/APOA1/FGB/MYOC/FGG | 5 |
| GO:0050433 | BP | GO:0050433 | regulation of catecholamine secretion | 3/219 | 62/18670 | 0.036312111 | 0.306831823 | 0.27697352 | HRH3/CRHR1/SYT10 | 3 |
| GO:0042129 | BP | GO:0042129 | regulation of T cell proliferation | 5/219 | 156/18670 | 0.03697506 | 0.308047148 | 0.278070579 | IGF2/LEP/CCL19/IL21/SCGB1A1 | 5 |
| GO:0035640 | BP | GO:0035640 | exploration behavior | 2/219 | 26/18670 | 0.037009981 | 0.308047148 | 0.278070579 | TNR/CRHR1 | 2 |
| GO:0042044 | BP | GO:0042044 | fluid transport | 2/219 | 26/18670 | 0.037009981 | 0.308047148 | 0.278070579 | AQP5/AQP4 | 2 |
| GO:0043567 | BP | GO:0043567 | regulation of insulin-like growth factor receptor signaling pathway | 2/219 | 26/18670 | 0.037009981 | 0.308047148 | 0.278070579 | CILP/WNT1 | 2 |
| GO:0071624 | BP | GO:0071624 | positive regulation of granulocyte chemotaxis | 2/219 | 26/18670 | 0.037009981 | 0.308047148 | 0.278070579 | CCL19/THBS4 | 2 |
| GO:0006631 | BP | GO:0006631 | fatty acid metabolic process | 9/219 | 383/18670 | 0.037321596 | 0.30971354 | 0.279574812 | GPAT2/AKR1C2/LEP/CYP1B1/CYP7A1/ACADL/APOA4/ADIPOQ/APOC3 | 9 |
| GO:0046888 | BP | GO:0046888 | negative regulation of hormone secretion | 3/219 | 63/18670 | 0.037808101 | 0.311243027 | 0.280955463 | LEP/ADIPOQ/FGF23 | 3 |
| GO:0090181 | BP | GO:0090181 | regulation of cholesterol metabolic process | 3/219 | 63/18670 | 0.037808101 | 0.311243027 | 0.280955463 | CYP7A1/ACADL/APOB | 3 |
| GO:0043271 | BP | GO:0043271 | negative regulation of ion transport | 5/219 | 157/18670 | 0.037841778 | 0.311243027 | 0.280955463 | BEST3/LEP/RRAD/HRH3/CRHR1 | 5 |
| GO:0002526 | BP | GO:0002526 | acute inflammatory response | 4/219 | 108/18670 | 0.038407343 | 0.314962868 | 0.28431332 | APOA2/ORM2/AHSG/SERPINA3 | 4 |
| GO:0048645 | BP | GO:0048645 | animal organ formation | 3/219 | 64/18670 | 0.039335203 | 0.316889086 | 0.286052094 | TP63/LRP2/GATA5 | 3 |
| GO:0050432 | BP | GO:0050432 | catecholamine secretion | 3/219 | 64/18670 | 0.039335203 | 0.316889086 | 0.286052094 | HRH3/CRHR1/SYT10 | 3 |
| GO:0006706 | BP | GO:0006706 | steroid catabolic process | 2/219 | 27/18670 | 0.03966813 | 0.316889086 | 0.286052094 | CYP7A1/FGF23 | 2 |
| GO:0035902 | BP | GO:0035902 | response to immobilization stress | 2/219 | 27/18670 | 0.03966813 | 0.316889086 | 0.286052094 | PITX3/CRHR1 | 2 |
| GO:0045671 | BP | GO:0045671 | negative regulation of osteoclast differentiation | 2/219 | 27/18670 | 0.03966813 | 0.316889086 | 0.286052094 | LTF/CLDN18 | 2 |
| GO:0048261 | BP | GO:0048261 | negative regulation of receptor-mediated endocytosis | 2/219 | 27/18670 | 0.03966813 | 0.316889086 | 0.286052094 | APOC3/SH3GL3 | 2 |
| GO:0048799 | BP | GO:0048799 | animal organ maturation | 2/219 | 27/18670 | 0.03966813 | 0.316889086 | 0.286052094 | LTF/LEP | 2 |
| GO:0072376 | BP | GO:0072376 | protein activation cascade | 2/219 | 27/18670 | 0.03966813 | 0.316889086 | 0.286052094 | FGB/FGG | 2 |
| GO:0072378 | BP | GO:0072378 | blood coagulation, fibrin clot formation | 2/219 | 27/18670 | 0.03966813 | 0.316889086 | 0.286052094 | FGB/FGG | 2 |
| GO:0043491 | BP | GO:0043491 | protein kinase B signaling | 7/219 | 269/18670 | 0.039792166 | 0.316969115 | 0.286124336 | AKR1C2/IGF2/LEP/CCL19/MYOC/LIN28A/FGF23 | 7 |
| GO:0021915 | BP | GO:0021915 | neural tube development | 5/219 | 160/18670 | 0.040516317 | 0.320898465 | 0.289671314 | GBX2/WNT1/TRIM71/LRP2/EN1 | 5 |
| GO:0032680 | BP | GO:0032680 | regulation of tumor necrosis factor production | 5/219 | 160/18670 | 0.040516317 | 0.320898465 | 0.289671314 | LTF/LEP/ORM2/CCL19/ADIPOQ | 5 |
| GO:0022037 | BP | GO:0022037 | metencephalon development | 4/219 | 111/18670 | 0.041793779 | 0.328970251 | 0.296957622 | GBX2/WNT1/EN1/GBX1 | 4 |
| GO:0031960 | BP | GO:0031960 | response to corticosteroid | 5/219 | 162/18670 | 0.042361594 | 0.328970251 | 0.296957622 | APOA2/CDO1/ADIPOQ/CPN1/SCGB1A1 | 5 |
| GO:0042730 | BP | GO:0042730 | fibrinolysis | 2/219 | 28/18670 | 0.042396386 | 0.328970251 | 0.296957622 | FGB/FGG | 2 |
| GO:0045761 | BP | GO:0045761 | regulation of adenylate cyclase activity | 2/219 | 28/18670 | 0.042396386 | 0.328970251 | 0.296957622 | GPR87/CRHR1 | 2 |
| GO:1902932 | BP | GO:1902932 | positive regulation of alcohol biosynthetic process | 2/219 | 28/18670 | 0.042396386 | 0.328970251 | 0.296957622 | CYP7A1/ADCYAP1R1 | 2 |
| GO:1903792 | BP | GO:1903792 | negative regulation of anion transport | 2/219 | 28/18670 | 0.042396386 | 0.328970251 | 0.296957622 | LEP/HRH3 | 2 |
| GO:0046626 | BP | GO:0046626 | regulation of insulin receptor signaling pathway | 3/219 | 66/18670 | 0.04248213 | 0.328970251 | 0.296957622 | IGF2/LEP/AHSG | 3 |
| GO:1901880 | BP | GO:1901880 | negative regulation of protein depolymerization | 3/219 | 66/18670 | 0.04248213 | 0.328970251 | 0.296957622 | ADD2/SPTBN4/TRIM54 | 3 |
| GO:0070268 | BP | GO:0070268 | cornification | 4/219 | 112/18670 | 0.042959001 | 0.331631621 | 0.299360009 | KRT14/KRT3/KRT24/SPRR2G | 4 |
| GO:0060348 | BP | GO:0060348 | bone development | 6/219 | 217/18670 | 0.043123078 | 0.331631621 | 0.299360009 | LTF/LEP/COL2A1/WNT1/CLDN18/MYOC | 6 |
| GO:0032640 | BP | GO:0032640 | tumor necrosis factor production | 5/219 | 163/18670 | 0.043302978 | 0.331631621 | 0.299360009 | LTF/LEP/ORM2/CCL19/ADIPOQ | 5 |
| GO:1903555 | BP | GO:1903555 | regulation of tumor necrosis factor superfamily cytokine production | 5/219 | 163/18670 | 0.043302978 | 0.331631621 | 0.299360009 | LTF/LEP/ORM2/CCL19/ADIPOQ | 5 |
| GO:0035821 | BP | GO:0035821 | modulation of process of other organism | 4/219 | 113/18670 | 0.044142422 | 0.336825347 | 0.304048325 | LTF/DEFA5/DEFA6/CLEC4M | 4 |
| GO:0006633 | BP | GO:0006633 | fatty acid biosynthetic process | 5/219 | 164/18670 | 0.044256885 | 0.336825347 | 0.304048325 | CYP7A1/ACADL/APOA4/ADIPOQ/APOC3 | 5 |
| GO:0007596 | BP | GO:0007596 | blood coagulation | 8/219 | 336/18670 | 0.045128674 | 0.336825347 | 0.304048325 | ANXA8/HBG2/FGB/GATA5/DGKK/FGG/SERPINA10/HBE1 | 8 |
| GO:0007628 | BP | GO:0007628 | adult walking behavior | 2/219 | 29/18670 | 0.045192753 | 0.336825347 | 0.304048325 | SPTBN4/GBX1 | 2 |
| GO:0009435 | BP | GO:0009435 | NAD biosynthetic process | 2/219 | 29/18670 | 0.045192753 | 0.336825347 | 0.304048325 | NMNAT2/SLC5A8 | 2 |
| GO:0010669 | BP | GO:0010669 | epithelial structure maintenance | 2/219 | 29/18670 | 0.045192753 | 0.336825347 | 0.304048325 | MUC6/SERPINA3 | 2 |
| GO:0032372 | BP | GO:0032372 | negative regulation of sterol transport | 2/219 | 29/18670 | 0.045192753 | 0.336825347 | 0.304048325 | APOA2/APOC3 | 2 |
| GO:0032375 | BP | GO:0032375 | negative regulation of cholesterol transport | 2/219 | 29/18670 | 0.045192753 | 0.336825347 | 0.304048325 | APOA2/APOC3 | 2 |
| GO:0060914 | BP | GO:0060914 | heart formation | 2/219 | 29/18670 | 0.045192753 | 0.336825347 | 0.304048325 | LRP2/GATA5 | 2 |
| GO:1902624 | BP | GO:1902624 | positive regulation of neutrophil migration | 2/219 | 29/18670 | 0.045192753 | 0.336825347 | 0.304048325 | CCL19/THBS4 | 2 |
| GO:0030282 | BP | GO:0030282 | bone mineralization | 4/219 | 114/18670 | 0.045344033 | 0.337049232 | 0.304250424 | LTF/LEP/AHSG/FGF23 | 4 |
| GO:0019915 | BP | GO:0019915 | lipid storage | 3/219 | 68/18670 | 0.04575156 | 0.338485118 | 0.305546582 | LEP/APOA1/APOB | 3 |
| GO:0007159 | BP | GO:0007159 | leukocyte cell-cell adhesion | 8/219 | 337/18670 | 0.045780721 | 0.338485118 | 0.305546582 | IGF2/LEP/ADD2/CCL19/IL21/APOA4/CLEC4M/SCGB1A1 | 8 |
| GO:0002697 | BP | GO:0002697 | regulation of immune effector process | 10/219 | 462/18670 | 0.046484749 | 0.341549934 | 0.308313155 | APOA2/LEP/CCL19/SUSD4/C7/APOA1/IL21/IGHV2-70/CPN1/CRHR1 | 10 |
| GO:0030449 | BP | GO:0030449 | regulation of complement activation | 4/219 | 115/18670 | 0.046563822 | 0.341549934 | 0.308313155 | SUSD4/C7/IGHV2-70/CPN1 | 4 |
| GO:0032612 | BP | GO:0032612 | interleukin-1 production | 4/219 | 115/18670 | 0.046563822 | 0.341549934 | 0.308313155 | PANX2/ORM2/CCL19/APOA1 | 4 |
| GO:0032102 | BP | GO:0032102 | negative regulation of response to external stimulus | 9/219 | 400/18670 | 0.046900152 | 0.343111637 | 0.309722887 | LTF/LEP/CTNNA2/APOA1/TNR/NRXN1/FGB/ADIPOQ/FGG | 9 |
| GO:0071300 | BP | GO:0071300 | cellular response to retinoic acid | 3/219 | 69/18670 | 0.047431758 | 0.344313578 | 0.310807864 | LEP/FZD10/BRINP2 | 3 |
| GO:0008544 | BP | GO:0008544 | epidermis development | 10/219 | 464/18670 | 0.047608964 | 0.344313578 | 0.310807864 | TP63/KRT14/DKK4/LCE1E/BNC1/KRTAP3-3/SERPINB13/KRT3/KRT24/SPRR2G | 10 |
| GO:0010743 | BP | GO:0010743 | regulation of macrophage derived foam cell differentiation | 2/219 | 30/18670 | 0.048055276 | 0.344313578 | 0.310807864 | ADIPOQ/APOB | 2 |
| GO:0048265 | BP | GO:0048265 | response to pain | 2/219 | 30/18670 | 0.048055276 | 0.344313578 | 0.310807864 | THBS4/CRHR1 | 2 |
| GO:0070168 | BP | GO:0070168 | negative regulation of biomineral tissue development | 2/219 | 30/18670 | 0.048055276 | 0.344313578 | 0.310807864 | AHSG/FGF23 | 2 |
| GO:0090022 | BP | GO:0090022 | regulation of neutrophil chemotaxis | 2/219 | 30/18670 | 0.048055276 | 0.344313578 | 0.310807864 | CCL19/THBS4 | 2 |
| GO:0110150 | BP | GO:0110150 | negative regulation of biomineralization | 2/219 | 30/18670 | 0.048055276 | 0.344313578 | 0.310807864 | AHSG/FGF23 | 2 |
| GO:1905606 | BP | GO:1905606 | regulation of presynapse assembly | 2/219 | 30/18670 | 0.048055276 | 0.344313578 | 0.310807864 | SLITRK2/NRXN1 | 2 |
| GO:0071706 | BP | GO:0071706 | tumor necrosis factor superfamily cytokine production | 5/219 | 168/18670 | 0.048198074 | 0.34444896 | 0.310930072 | LTF/LEP/ORM2/CCL19/ADIPOQ | 5 |
| GO:0007599 | BP | GO:0007599 | hemostasis | 8/219 | 341/18670 | 0.048450746 | 0.345366855 | 0.311758646 | ANXA8/HBG2/FGB/GATA5/DGKK/FGG/SERPINA10/HBE1 | 8 |
| GO:0045785 | BP | GO:0045785 | positive regulation of cell adhesion | 9/219 | 403/18670 | 0.048745822 | 0.345860275 | 0.31220405 | IGF2/LEP/CCL19/APOA1/IL21/PRSS2/FGB/MYOC/FGG | 9 |
| GO:0031424 | BP | GO:0031424 | keratinization | 6/219 | 224/18670 | 0.048962355 | 0.345860275 | 0.31220405 | KRT14/LCE1E/KRTAP3-3/KRT3/KRT24/SPRR2G | 6 |
| GO:0050817 | BP | GO:0050817 | coagulation | 8/219 | 342/18670 | 0.049133789 | 0.345860275 | 0.31220405 | ANXA8/HBG2/FGB/GATA5/DGKK/FGG/SERPINA10/HBE1 | 8 |
| GO:0051966 | BP | GO:0051966 | regulation of synaptic transmission, glutamatergic | 3/219 | 70/18670 | 0.049142018 | 0.345860275 | 0.31220405 | GRM1/TNR/NRXN1 | 3 |
| GO:0060395 | BP | GO:0060395 | SMAD protein signal transduction | 3/219 | 70/18670 | 0.049142018 | 0.345860275 | 0.31220405 | AFP/CILP/GDF10 | 3 |
| GO:0001649 | BP | GO:0001649 | osteoblast differentiation | 6/219 | 225/18670 | 0.049834424 | 0.3490888 | 0.315118402 | IGF2/LTF/TP63/GDF10/MYOC/FGF23 | 6 |
