# Supplementary material for: Prognostic and Predictive Models for Left- and Right- Colorectal Cancer Patients: A Bioinformatics Analysis Based on Ferroptosis-Related Genes
Source: Front Oncol. 2022 Feb 21;12:833834. doi: 10.3389/fonc.2022.833834 (PMC8899601; doi:10.3389/fonc.2022.833834)
Supplement: Supplementary Table 6 — KEGG pathway analysis in left- and right- colorectal cancer. KEGG – Kyoto Encyclopedia of Genes and Genomes. P-value <0.05. [file Table_6.docx]

| ID | Description | GeneRatio | BgRatio | pvalue | p.adjust | qvalue | geneID | Count |  |
| --- | --- | --- | --- | --- | --- | --- | --- | --- | --- |
| hsa04972 | hsa04972 | Pancreatic secretion | 4/35 | 102/8075 | 0.000930074 | 0.094867575 | 0.089091324 | PRSS1/CELA2A/CPB1/PLA2G2E | 4 |
| hsa04014 | hsa04014 | Ras signaling pathway | 5/35 | 232/8075 | 0.003009966 | 0.153508266 | 0.14416153 | NTRK2/NTF4/IGF2/CALML5/PLA2G2E | 5 |
| hsa04974 | hsa04974 | Protein digestion and absorption | 3/35 | 103/8075 | 0.009812237 | 0.333616068 | 0.313303015 | PRSS1/CELA2A/CPB1 | 3 |
| hsa04722 | hsa04722 | Neurotrophin signaling pathway | 3/35 | 119/8075 | 0.014493757 | 0.3695908 | 0.347087336 | NTRK2/NTF4/CALML5 | 3 |
| hsa00140 | hsa00140 | Steroid hormone biosynthesis | 2/35 | 61/8075 | 0.02846832 | 0.41722914 | 0.391825096 | CYP1A1/UGT1A3 | 2 |
| hsa00830 | hsa00830 | Retinol metabolism | 2/35 | 67/8075 | 0.033845577 | 0.41722914 | 0.391825096 | CYP1A1/UGT1A3 | 2 |
| hsa04060 | hsa04060 | Cytokine-cytokine receptor interaction | 4/35 | 295/8075 | 0.037535168 | 0.41722914 | 0.391825096 | GDF10/CCL25/PPBP/IL36B | 4 |
| hsa03320 | hsa03320 | PPAR signaling pathway | 2/35 | 76/8075 | 0.042587609 | 0.41722914 | 0.391825096 | FABP3/ADIPOQ | 2 |
| hsa05133 | hsa05133 | Pertussis | 2/35 | 76/8075 | 0.042587609 | 0.41722914 | 0.391825096 | SFTPA1/CALML5 | 2 |
| hsa00980 | hsa00980 | Metabolism of xenobiotics by cytochrome P450 | 2/35 | 78/8075 | 0.044634384 | 0.41722914 | 0.391825096 | CYP1A1/UGT1A3 | 2 |
| hsa05204 | hsa05204 | Chemical carcinogenesis | 2/35 | 83/8075 | 0.049908137 | 0.41722914 | 0.391825096 | CYP1A1/UGT1A3 | 2 |
| hsa04610 | hsa04610 | Complement and coagulation cascades | 2/35 | 85/8075 | 0.052078442 | 0.41722914 | 0.391825096 | FGB/FGG | 2 |
| hsa04742 | hsa04742 | Taste transduction | 2/35 | 86/8075 | 0.053176263 | 0.41722914 | 0.391825096 | HTR3A/SCNN1G | 2 |
| hsa04061 | hsa04061 | Viral protein interaction with cytokine and cytokine receptor | 2/35 | 100/8075 | 0.06938082 | 0.50548883 | 0.474710872 | CCL25/PPBP | 2 |
| hsa04611 | hsa04611 | Platelet activation | 2/35 | 124/8075 | 0.100303931 | 0.513070356 | 0.481830778 | FGB/FGG | 2 |
| hsa00592 | hsa00592 | alpha-Linolenic acid metabolism | 1/35 | 25/8075 | 0.103051387 | 0.513070356 | 0.481830778 | PLA2G2E | 1 |
| hsa04270 | hsa04270 | Vascular smooth muscle contraction | 2/35 | 133/8075 | 0.112748853 | 0.513070356 | 0.481830778 | CALML5/PLA2G2E | 2 |
| hsa04744 | hsa04744 | Phototransduction | 1/35 | 28/8075 | 0.114701365 | 0.513070356 | 0.481830778 | CALML5 | 1 |
| hsa00591 | hsa00591 | Linoleic acid metabolism | 1/35 | 29/8075 | 0.118551925 | 0.513070356 | 0.481830778 | PLA2G2E | 1 |
| hsa00053 | hsa00053 | Ascorbate and aldarate metabolism | 1/35 | 30/8075 | 0.122386213 | 0.513070356 | 0.481830778 | UGT1A3 | 1 |
| hsa04010 | hsa04010 | MAPK signaling pathway | 3/35 | 294/8075 | 0.133516589 | 0.513070356 | 0.481830778 | NTRK2/NTF4/IGF2 | 3 |
| hsa04514 | hsa04514 | Cell adhesion molecules | 2/35 | 149/8075 | 0.135795137 | 0.513070356 | 0.481830778 | L1CAM/VTCN1 | 2 |
| hsa00040 | hsa00040 | Pentose and glucuronate interconversions | 1/35 | 34/8075 | 0.137561983 | 0.513070356 | 0.481830778 | UGT1A3 | 1 |
| hsa03040 | hsa03040 | Spliceosome | 2/35 | 151/8075 | 0.138748857 | 0.513070356 | 0.481830778 | RNU4-2/RNU4-1 | 2 |
| hsa04960 | hsa04960 | Aldosterone-regulated sodium reabsorption | 1/35 | 37/8075 | 0.148776206 | 0.513070356 | 0.481830778 | SCNN1G | 1 |
| hsa00260 | hsa00260 | Glycine, serine and threonine metabolism | 1/35 | 40/8075 | 0.159848725 | 0.513070356 | 0.481830778 | BHMT | 1 |
| hsa00380 | hsa00380 | Tryptophan metabolism | 1/35 | 42/8075 | 0.167152539 | 0.513070356 | 0.481830778 | CYP1A1 | 1 |
| hsa00860 | hsa00860 | Porphyrin and chlorophyll metabolism | 1/35 | 42/8075 | 0.167152539 | 0.513070356 | 0.481830778 | UGT1A3 | 1 |
| hsa04975 | hsa04975 | Fat digestion and absorption | 1/35 | 43/8075 | 0.170781278 | 0.513070356 | 0.481830778 | PLA2G2E | 1 |
| hsa04930 | hsa04930 | Type II diabetes mellitus | 1/35 | 46/8075 | 0.181575584 | 0.513070356 | 0.481830778 | ADIPOQ | 1 |
| hsa04080 | hsa04080 | Neuroactive ligand-receptor interaction | 3/35 | 341/8075 | 0.182460507 | 0.513070356 | 0.481830778 | UTS2R/PRSS1/GLP1R | 3 |
| hsa04360 | hsa04360 | Axon guidance | 2/35 | 181/8075 | 0.184568131 | 0.513070356 | 0.481830778 | EPHA8/L1CAM | 2 |
| hsa00565 | hsa00565 | Ether lipid metabolism | 1/35 | 49/8075 | 0.192233338 | 0.513070356 | 0.481830778 | PLA2G2E | 1 |
| hsa04672 | hsa04672 | Intestinal immune network for IgA production | 1/35 | 49/8075 | 0.192233338 | 0.513070356 | 0.481830778 | CCL25 | 1 |
| hsa03013 | hsa03013 | RNA transport | 2/35 | 186/8075 | 0.192425396 | 0.513070356 | 0.481830778 | RNU4-2/RNU4-1 | 2 |
| hsa05034 | hsa05034 | Alcoholism | 2/35 | 187/8075 | 0.194002699 | 0.513070356 | 0.481830778 | NTRK2/CALML5 | 2 |
| hsa00270 | hsa00270 | Cysteine and methionine metabolism | 1/35 | 50/8075 | 0.195755869 | 0.513070356 | 0.481830778 | BHMT | 1 |
| hsa04151 | hsa04151 | PI3K-Akt signaling pathway | 3/35 | 354/8075 | 0.196809682 | 0.513070356 | 0.481830778 | NTRK2/NTF4/IGF2 | 3 |
| hsa04913 | hsa04913 | Ovarian steroidogenesis | 1/35 | 51/8075 | 0.199263476 | 0.513070356 | 0.481830778 | CYP1A1 | 1 |
| hsa04062 | hsa04062 | Chemokine signaling pathway | 2/35 | 192/8075 | 0.201916026 | 0.513070356 | 0.481830778 | CCL25/PPBP | 2 |
| hsa04961 | hsa04961 | Endocrine and other factor-regulated calcium reabsorption | 1/35 | 53/8075 | 0.206234163 | 0.513070356 | 0.481830778 | CALB1 | 1 |
| hsa05205 | hsa05205 | Proteoglycans in cancer | 2/35 | 205/8075 | 0.222668494 | 0.519870395 | 0.48821678 | GPC3/IGF2 | 2 |
| hsa05213 | hsa05213 | Endometrial cancer | 1/35 | 58/8075 | 0.223403973 | 0.519870395 | 0.48821678 | CTNNA2 | 1 |
| hsa00590 | hsa00590 | Arachidonic acid metabolism | 1/35 | 61/8075 | 0.2335321 | 0.519870395 | 0.48821678 | PLA2G2E | 1 |
| hsa04024 | hsa04024 | cAMP signaling pathway | 2/35 | 216/8075 | 0.240377868 | 0.519870395 | 0.48821678 | GLP1R/CALML5 | 2 |
| hsa04720 | hsa04720 | Long-term potentiation | 1/35 | 67/8075 | 0.253404848 | 0.519870395 | 0.48821678 | CALML5 | 1 |
| hsa04920 | hsa04920 | Adipocytokine signaling pathway | 1/35 | 69/8075 | 0.259917173 | 0.519870395 | 0.48821678 | ADIPOQ | 1 |
| hsa04924 | hsa04924 | Renin secretion | 1/35 | 69/8075 | 0.259917173 | 0.519870395 | 0.48821678 | CALML5 | 1 |
| hsa05031 | hsa05031 | Amphetamine addiction | 1/35 | 69/8075 | 0.259917173 | 0.519870395 | 0.48821678 | CALML5 | 1 |
| hsa04520 | hsa04520 | Adherens junction | 1/35 | 71/8075 | 0.266374303 | 0.519870395 | 0.48821678 | CTNNA2 | 1 |
| hsa00982 | hsa00982 | Drug metabolism - cytochrome P450 | 1/35 | 72/8075 | 0.269582311 | 0.519870395 | 0.48821678 | UGT1A3 | 1 |
| hsa04918 | hsa04918 | Thyroid hormone synthesis | 1/35 | 75/8075 | 0.279124788 | 0.519870395 | 0.48821678 | SERPINA7 | 1 |
| hsa05214 | hsa05214 | Glioma | 1/35 | 75/8075 | 0.279124788 | 0.519870395 | 0.48821678 | CALML5 | 1 |
| hsa04971 | hsa04971 | Gastric acid secretion | 1/35 | 76/8075 | 0.282278617 | 0.519870395 | 0.48821678 | CALML5 | 1 |
| hsa05100 | hsa05100 | Bacterial invasion of epithelial cells | 1/35 | 77/8075 | 0.28541904 | 0.519870395 | 0.48821678 | CTNNA2 | 1 |
| hsa05412 | hsa05412 | Arrhythmogenic right ventricular cardiomyopathy | 1/35 | 77/8075 | 0.28541904 | 0.519870395 | 0.48821678 | CTNNA2 | 1 |
| hsa00983 | hsa00983 | Drug metabolism - other enzymes | 1/35 | 80/8075 | 0.294760431 | 0.522550954 | 0.490734126 | UGT1A3 | 1 |
| hsa04911 | hsa04911 | Insulin secretion | 1/35 | 86/8075 | 0.313088661 | 0.522550954 | 0.490734126 | GLP1R | 1 |
| hsa04211 | hsa04211 | Longevity regulating pathway | 1/35 | 89/8075 | 0.322078412 | 0.522550954 | 0.490734126 | ADIPOQ | 1 |
| hsa04976 | hsa04976 | Bile secretion | 1/35 | 90/8075 | 0.325049519 | 0.522550954 | 0.490734126 | UGT1A3 | 1 |
| hsa04912 | hsa04912 | GnRH signaling pathway | 1/35 | 93/8075 | 0.333887141 | 0.522550954 | 0.490734126 | CALML5 | 1 |
| hsa04970 | hsa04970 | Salivary secretion | 1/35 | 93/8075 | 0.333887141 | 0.522550954 | 0.490734126 | CALML5 | 1 |
| hsa05150 | hsa05150 | Staphylococcus aureus infection | 1/35 | 96/8075 | 0.34261231 | 0.522550954 | 0.490734126 | FGG | 1 |
| hsa04070 | hsa04070 | Phosphatidylinositol signaling system | 1/35 | 97/8075 | 0.345495951 | 0.522550954 | 0.490734126 | CALML5 | 1 |
| hsa04713 | hsa04713 | Circadian entrainment | 1/35 | 97/8075 | 0.345495951 | 0.522550954 | 0.490734126 | CALML5 | 1 |
| hsa00564 | hsa00564 | Glycerophospholipid metabolism | 1/35 | 98/8075 | 0.348367302 | 0.522550954 | 0.490734126 | PLA2G2E | 1 |
| hsa04750 | hsa04750 | Inflammatory mediator regulation of TRP channels | 1/35 | 98/8075 | 0.348367302 | 0.522550954 | 0.490734126 | CALML5 | 1 |
| hsa04925 | hsa04925 | Aldosterone synthesis and secretion | 1/35 | 98/8075 | 0.348367302 | 0.522550954 | 0.490734126 | CALML5 | 1 |
| hsa04916 | hsa04916 | Melanogenesis | 1/35 | 101/8075 | 0.356908128 | 0.52760332 | 0.495478866 | CALML5 | 1 |
| hsa04625 | hsa04625 | C-type lectin receptor signaling pathway | 1/35 | 104/8075 | 0.365340169 | 0.532352818 | 0.499939179 | CALML5 | 1 |
| hsa04922 | hsa04922 | Glucagon signaling pathway | 1/35 | 107/8075 | 0.37366477 | 0.536814176 | 0.504128896 | CALML5 | 1 |
| hsa04670 | hsa04670 | Leukocyte transendothelial migration | 1/35 | 114/8075 | 0.392678452 | 0.552404754 | 0.518770203 | CTNNA2 | 1 |
| hsa04726 | hsa04726 | Serotonergic synapse | 1/35 | 115/8075 | 0.395348501 | 0.552404754 | 0.518770203 | HTR3A | 1 |
| hsa04152 | hsa04152 | AMPK signaling pathway | 1/35 | 120/8075 | 0.408528614 | 0.563107008 | 0.528820823 | ADIPOQ | 1 |
| hsa04114 | hsa04114 | Oocyte meiosis | 1/35 | 129/8075 | 0.431552938 | 0.581546158 | 0.546137258 | CALML5 | 1 |
| hsa04728 | hsa04728 | Dopaminergic synapse | 1/35 | 132/8075 | 0.439032408 | 0.581546158 | 0.546137258 | CALML5 | 1 |
| hsa04371 | hsa04371 | Apelin signaling pathway | 1/35 | 137/8075 | 0.451286252 | 0.581546158 | 0.546137258 | CALML5 | 1 |
| hsa04910 | hsa04910 | Insulin signaling pathway | 1/35 | 137/8075 | 0.451286252 | 0.581546158 | 0.546137258 | CALML5 | 1 |
| hsa04915 | hsa04915 | Estrogen signaling pathway | 1/35 | 138/8075 | 0.453705625 | 0.581546158 | 0.546137258 | CALML5 | 1 |
| hsa05418 | hsa05418 | Fluid shear stress and atherosclerosis | 1/35 | 139/8075 | 0.456114633 | 0.581546158 | 0.546137258 | CALML5 | 1 |
| hsa05226 | hsa05226 | Gastric cancer | 1/35 | 149/8075 | 0.4796441 | 0.583572313 | 0.548040046 | CTNNA2 | 1 |
| hsa04261 | hsa04261 | Adrenergic signaling in cardiomyocytes | 1/35 | 150/8075 | 0.481941912 | 0.583572313 | 0.548040046 | CALML5 | 1 |
| hsa04932 | hsa04932 | Non-alcoholic fatty liver disease | 1/35 | 150/8075 | 0.481941912 | 0.583572313 | 0.548040046 | ADIPOQ | 1 |
| hsa04145 | hsa04145 | Phagosome | 1/35 | 152/8075 | 0.486508002 | 0.583572313 | 0.548040046 | SFTPA1 | 1 |
| hsa04921 | hsa04921 | Oxytocin signaling pathway | 1/35 | 154/8075 | 0.491034988 | 0.583572313 | 0.548040046 | CALML5 | 1 |
| hsa04218 | hsa04218 | Cellular senescence | 1/35 | 156/8075 | 0.495523193 | 0.583572313 | 0.548040046 | CALML5 | 1 |
| hsa04390 | hsa04390 | Hippo signaling pathway | 1/35 | 157/8075 | 0.497752855 | 0.583572313 | 0.548040046 | CTNNA2 | 1 |
| hsa04022 | hsa04022 | cGMP-PKG signaling pathway | 1/35 | 167/8075 | 0.519529419 | 0.597852867 | 0.561451093 | CALML5 | 1 |
| hsa05225 | hsa05225 | Hepatocellular carcinoma | 1/35 | 168/8075 | 0.521655933 | 0.597852867 | 0.561451093 | IGF2 | 1 |
| hsa05164 | hsa05164 | Influenza A | 1/35 | 171/8075 | 0.527980763 | 0.598378198 | 0.561944438 | PRSS1 | 1 |
| hsa05152 | hsa05152 | Tuberculosis | 1/35 | 180/8075 | 0.546471673 | 0.612528688 | 0.57523334 | CALML5 | 1 |
| hsa05202 | hsa05202 | Transcriptional misregulation in cancer | 1/35 | 192/8075 | 0.57003493 | 0.62729336 | 0.589099028 | PAX3 | 1 |
| hsa05167 | hsa05167 | Kaposi sarcoma-associated herpesvirus infection | 1/35 | 193/8075 | 0.571943946 | 0.62729336 | 0.589099028 | CALML5 | 1 |
| hsa04020 | hsa04020 | Calcium signaling pathway | 1/35 | 201/8075 | 0.586922505 | 0.636873357 | 0.598095722 | CALML5 | 1 |
| hsa04015 | hsa04015 | Rap1 signaling pathway | 1/35 | 210/8075 | 0.603165019 | 0.644607371 | 0.605358832 | CALML5 | 1 |
| hsa05170 | hsa05170 | Human immunodeficiency virus 1 infection | 1/35 | 212/8075 | 0.60668929 | 0.644607371 | 0.605358832 | CALML5 | 1 |
| hsa05163 | hsa05163 | Human cytomegalovirus infection | 1/35 | 225/8075 | 0.628867039 | 0.661282866 | 0.621018997 | CALML5 | 1 |
| hsa05012 | hsa05012 | Parkinson disease | 1/35 | 249/8075 | 0.666662005 | 0.693872699 | 0.651624516 | CALML5 | 1 |
| hsa05010 | hsa05010 | Alzheimer disease | 1/35 | 369/8075 | 0.806139311 | 0.830567775 | 0.779996569 | CALML5 | 1 |
| hsa04740 | hsa04740 | Olfactory transduction | 1/35 | 447/8075 | 0.864323504 | 0.881609975 | 0.827930936 | CALML5 | 1 |
| hsa05022 | hsa05022 | Pathways of neurodegeneration - multiple diseases | 1/35 | 475/8075 | 0.880743733 | 0.889463968 | 0.835306719 | CALML5 | 1 |
| hsa05168 | hsa05168 | Herpes simplex virus 1 infection | 1/35 | 498/8075 | 0.892772295 | 0.892772295 | 0.83841361 | ZNF556 | 1 |
