# Supplementary material for: Prognostic and Predictive Models for Left- and Right- Colorectal Cancer Patients: A Bioinformatics Analysis Based on Ferroptosis-Related Genes
Source: Front Oncol. 2022 Feb 21;12:833834. doi: 10.3389/fonc.2022.833834 (PMC8899601; doi:10.3389/fonc.2022.833834)
Supplement: Supplementary Table 7 — KEGG pathway analysis in left- and right- colorectal cancer. KEGG – Kyoto Encyclopedia of Genes and Genomes. P-value <0.05. [file Table_7.docx]

|  | ID | Description | GeneRatio | BgRatio | pvalue | p.adjust | qvalue | geneID | Count |
| --- | --- | --- | --- | --- | --- | --- | --- | --- | --- |
| hsa04979 | hsa04979 | Cholesterol metabolism | 7/96 | 50/8081 | 1.76E-06 | 0.00028031 | 0.000269083 | APOA2/CYP7A1/APOA1/APOA4/LRP2/APOC3/APOB | 7 |
| hsa03320 | hsa03320 | PPAR signaling pathway | 7/96 | 76/8081 | 3.00E-05 | 0.002386111 | 0.00229054 | PLIN4/APOA2/CYP7A1/ACADL/APOA1/ADIPOQ/APOC3 | 7 |
| hsa05150 | hsa05150 | Staphylococcus aureus infection | 6/96 | 96/8081 | 0.000942199 | 0.049936527 | 0.047936421 | DEFA5/KRT14/DEFA6/FGG/DEFB4A/KRT24 | 6 |
| hsa04974 | hsa04974 | Protein digestion and absorption | 6/96 | 103/8081 | 0.00135975 | 0.054050065 | 0.0518852 | ATP1A3/COL2A1/PRSS1/KCNJ13/CPA1/PRSS2 | 6 |
| hsa04080 | hsa04080 | Neuroactive ligand-receptor interaction | 11/96 | 341/8081 | 0.002279942 | 0.072502154 | 0.069598228 | LEP/GRM1/OPRD1/PRSS1/ADCYAP1R1/GABRR1/PRSS2/HRH3/GRIN2A/MLN/CRHR1 | 11 |
| hsa04977 | hsa04977 | Vitamin digestion and absorption | 3/96 | 24/8081 | 0.002744089 | 0.072718351 | 0.069805766 | APOA1/APOA4/APOB | 3 |
| hsa04918 | hsa04918 | Thyroid hormone synthesis | 4/96 | 75/8081 | 0.011954755 | 0.271543729 | 0.260667598 | ALB/ATP1A3/LRP2/TTR | 4 |
| hsa04975 | hsa04975 | Fat digestion and absorption | 3/96 | 43/8081 | 0.014225378 | 0.282729383 | 0.271405234 | APOA1/APOA4/APOB | 3 |
| hsa04973 | hsa04973 | Carbohydrate digestion and absorption | 3/96 | 47/8081 | 0.018066993 | 0.319183544 | 0.306399298 | ATP1A3/G6PC/SLC2A2 | 3 |
| hsa04976 | hsa04976 | Bile secretion | 4/96 | 90/8081 | 0.021982584 | 0.34952308 | 0.335523645 | ATP1A3/CYP7A1/SLC10A2/AQP4 | 4 |
| hsa04972 | hsa04972 | Pancreatic secretion | 4/96 | 102/8081 | 0.03286383 | 0.461459299 | 0.442976487 | ATP1A3/PRSS1/CPA1/PRSS2 | 4 |
| hsa00140 | hsa00140 | Steroid hormone biosynthesis | 3/96 | 61/8081 | 0.035629163 | 0.461459299 | 0.442976487 | AKR1C2/CYP1B1/CYP7A1 | 3 |
| hsa04950 | hsa04950 | Maturity onset diabetes of the young | 2/96 | 26/8081 | 0.037729377 | 0.461459299 | 0.442976487 | NEUROG3/SLC2A2 | 2 |
| hsa04920 | hsa04920 | Adipocytokine signaling pathway | 3/96 | 69/8081 | 0.048501082 | 0.55083372 | 0.528771198 | LEP/G6PC/ADIPOQ | 3 |
| hsa00350 | hsa00350 | Tyrosine metabolism | 2/96 | 36/8081 | 0.067795337 | 0.612432458 | 0.587902723 | TYR/ADH1B | 2 |
| hsa05204 | hsa05204 | Chemical carcinogenesis | 3/96 | 83/8081 | 0.075685382 | 0.612432458 | 0.587902723 | AKR1C2/CYP1B1/ADH1B | 3 |
| hsa05202 | hsa05202 | Transcriptional misregulation in cancer | 5/96 | 192/8081 | 0.077957072 | 0.612432458 | 0.587902723 | NGFR/DEFA5/PAX5/DEFA6/PAX3 | 5 |
| hsa04610 | hsa04610 | Complement and coagulation cascades | 3/96 | 85/8081 | 0.080021449 | 0.612432458 | 0.587902723 | C7/FGB/FGG | 3 |
| hsa05033 | hsa05033 | Nicotine addiction | 2/96 | 40/8081 | 0.081445795 | 0.612432458 | 0.587902723 | GABRR1/GRIN2A | 2 |
| hsa04911 | hsa04911 | Insulin secretion | 3/96 | 86/8081 | 0.082229505 | 0.612432458 | 0.587902723 | ATP1A3/ADCYAP1R1/SLC2A2 | 3 |
| hsa04512 | hsa04512 | ECM-receptor interaction | 3/96 | 88/8081 | 0.086724139 | 0.612432458 | 0.587902723 | COL2A1/THBS4/TNR | 3 |
| hsa00071 | hsa00071 | Fatty acid degradation | 2/96 | 43/8081 | 0.092189585 | 0.612432458 | 0.587902723 | ACADL/ADH1B | 2 |
| hsa04970 | hsa04970 | Salivary secretion | 3/96 | 93/8081 | 0.09840409 | 0.612432458 | 0.587902723 | AQP5/ATP1A3/CST4 | 3 |
| hsa04514 | hsa04514 | Cell adhesion molecules | 4/96 | 149/8081 | 0.100964305 | 0.612432458 | 0.587902723 | NTNG1/CADM3/NRXN1/CLDN18 | 4 |
| hsa05226 | hsa05226 | Gastric cancer | 4/96 | 149/8081 | 0.100964305 | 0.612432458 | 0.587902723 | CTNNA2/FZD10/WNT1/FGF23 | 4 |
| hsa04930 | hsa04930 | Type II diabetes mellitus | 2/96 | 46/8081 | 0.103319854 | 0.612432458 | 0.587902723 | ADIPOQ/SLC2A2 | 2 |
| hsa04934 | hsa04934 | Cushing syndrome | 4/96 | 155/8081 | 0.112417735 | 0.612432458 | 0.587902723 | MRAP/FZD10/WNT1/CRHR1 | 4 |
| hsa04390 | hsa04390 | Hippo signaling pathway | 4/96 | 157/8081 | 0.116360035 | 0.612432458 | 0.587902723 | AFP/CTNNA2/FZD10/WNT1 | 4 |
| hsa04916 | hsa04916 | Melanogenesis | 3/96 | 101/8081 | 0.118324724 | 0.612432458 | 0.587902723 | TYR/FZD10/WNT1 | 3 |
| hsa00270 | hsa00270 | Cysteine and methionine metabolism | 2/96 | 50/8081 | 0.118692819 | 0.612432458 | 0.587902723 | BHMT2/CDO1 | 2 |
| hsa04310 | hsa04310 | Wnt signaling pathway | 4/96 | 160/8081 | 0.122386588 | 0.612432458 | 0.587902723 | FZD10/WNT1/DKK4/SOST | 4 |
| hsa00430 | hsa00430 | Taurine and hypotaurine metabolism | 1/96 | 11/8081 | 0.123256847 | 0.612432458 | 0.587902723 | CDO1 | 1 |
| hsa04151 | hsa04151 | PI3K-Akt signaling pathway | 7/96 | 354/8081 | 0.127249521 | 0.613111327 | 0.588554402 | IGF2/COL2A1/THBS4/NGFR/TNR/G6PC/FGF23 | 7 |
| hsa04060 | hsa04060 | Cytokine-cytokine receptor interaction | 6/96 | 295/8081 | 0.138049104 | 0.645582575 | 0.61972508 | LEP/CCL19/NGFR/CXCR5/IL21/GDF10 | 6 |
| hsa04978 | hsa04978 | Mineral absorption | 2/96 | 59/8081 | 0.155089614 | 0.667720999 | 0.640976795 | ATP1A3/SLC26A9 | 2 |
| hsa04730 | hsa04730 | Long-term depression | 2/96 | 60/8081 | 0.159259492 | 0.667720999 | 0.640976795 | GRM1/CRHR1 | 2 |
| hsa00561 | hsa00561 | Glycerolipid metabolism | 2/96 | 61/8081 | 0.163450351 | 0.667720999 | 0.640976795 | GPAT2/DGKK | 2 |
| hsa04621 | hsa04621 | NOD-like receptor signaling pathway | 4/96 | 181/8081 | 0.168060168 | 0.667720999 | 0.640976795 | DEFA5/DEFA6/GPRC6A/DEFB4A | 4 |
| hsa04152 | hsa04152 | AMPK signaling pathway | 3/96 | 120/8081 | 0.17078593 | 0.667720999 | 0.640976795 | LEP/G6PC/ADIPOQ | 3 |
| hsa05217 | hsa05217 | Basal cell carcinoma | 2/96 | 63/8081 | 0.171890747 | 0.667720999 | 0.640976795 | FZD10/WNT1 | 2 |
| hsa00120 | hsa00120 | Primary bile acid biosynthesis | 1/96 | 17/8081 | 0.184021267 | 0.667720999 | 0.640976795 | CYP7A1 | 1 |
| hsa00360 | hsa00360 | Phenylalanine metabolism | 1/96 | 17/8081 | 0.184021267 | 0.667720999 | 0.640976795 | GLYAT | 1 |
| hsa00910 | hsa00910 | Nitrogen metabolism | 1/96 | 17/8081 | 0.184021267 | 0.667720999 | 0.640976795 | CA6 | 1 |
| hsa00010 | hsa00010 | Glycolysis / Gluconeogenesis | 2/96 | 67/8081 | 0.188977641 | 0.667720999 | 0.640976795 | G6PC/ADH1B | 2 |
| hsa04720 | hsa04720 | Long-term potentiation | 2/96 | 67/8081 | 0.188977641 | 0.667720999 | 0.640976795 | GRM1/GRIN2A | 2 |
| hsa00982 | hsa00982 | Drug metabolism - cytochrome P450 | 2/96 | 72/8081 | 0.210643519 | 0.728093901 | 0.698931583 | FMO2/ADH1B | 2 |
| hsa04915 | hsa04915 | Estrogen signaling pathway | 3/96 | 138/8081 | 0.225453139 | 0.762703173 | 0.732154651 | GRM1/KRT14/KRT24 | 3 |
| hsa00980 | hsa00980 | Metabolism of xenobiotics by cytochrome P450 | 2/96 | 78/8081 | 0.236948534 | 0.780771543 | 0.74949933 | CYP1B1/ADH1B | 2 |
| hsa04964 | hsa04964 | Proximal tubule bicarbonate reclamation | 1/96 | 23/8081 | 0.24061513 | 0.780771543 | 0.74949933 | ATP1A3 | 1 |
| hsa05224 | hsa05224 | Breast cancer | 3/96 | 147/8081 | 0.253980117 | 0.807656773 | 0.775307727 | FZD10/WNT1/FGF23 | 3 |
| hsa04145 | hsa04145 | Phagosome | 3/96 | 152/8081 | 0.270056306 | 0.817904951 | 0.785145435 | SFTPA1/THBS4/CLEC4M | 3 |
| hsa04014 | hsa04014 | Ras signaling pathway | 4/96 | 232/8081 | 0.297130674 | 0.817904951 | 0.785145435 | IGF2/NGFR/GRIN2A/FGF23 | 4 |
| hsa00030 | hsa00030 | Pentose phosphate pathway | 1/96 | 30/8081 | 0.301747246 | 0.817904951 | 0.785145435 | TKTL1 | 1 |
| hsa04630 | hsa04630 | JAK-STAT signaling pathway | 3/96 | 162/8081 | 0.30252498 | 0.817904951 | 0.785145435 | LEP/GFAP/IL21 | 3 |
| hsa00052 | hsa00052 | Galactose metabolism | 1/96 | 31/8081 | 0.310073201 | 0.817904951 | 0.785145435 | G6PC | 1 |
| hsa00512 | hsa00512 | Mucin type O-glycan biosynthesis | 1/96 | 32/8081 | 0.318300899 | 0.817904951 | 0.785145435 | GALNTL6 | 1 |
| hsa04215 | hsa04215 | Apoptosis - multiple species | 1/96 | 32/8081 | 0.318300899 | 0.817904951 | 0.785145435 | NGFR | 1 |
| hsa04713 | hsa04713 | Circadian entrainment | 2/96 | 97/8081 | 0.320716593 | 0.817904951 | 0.785145435 | ADCYAP1R1/GRIN2A | 2 |
| hsa05225 | hsa05225 | Hepatocellular carcinoma | 3/96 | 168/8081 | 0.322118663 | 0.817904951 | 0.785145435 | IGF2/FZD10/WNT1 | 3 |
| hsa00564 | hsa00564 | Glycerophospholipid metabolism | 2/96 | 98/8081 | 0.325092251 | 0.817904951 | 0.785145435 | GPAT2/DGKK | 2 |
| hsa04061 | hsa04061 | Viral protein interaction with cytokine and cytokine receptor | 2/96 | 100/8081 | 0.333822283 | 0.817904951 | 0.785145435 | CCL19/CXCR5 | 2 |
| hsa00760 | hsa00760 | Nicotinate and nicotinamide metabolism | 1/96 | 35/8081 | 0.342405864 | 0.817904951 | 0.785145435 | NMNAT2 | 1 |
| hsa00500 | hsa00500 | Starch and sucrose metabolism | 1/96 | 36/8081 | 0.350251879 | 0.817904951 | 0.785145435 | G6PC | 1 |
| hsa05165 | hsa05165 | Human papillomavirus infection | 5/96 | 331/8081 | 0.357476231 | 0.817904951 | 0.785145435 | COL2A1/THBS4/TNR/FZD10/WNT1 | 5 |
| hsa00250 | hsa00250 | Alanine, aspartate and glutamate metabolism | 1/96 | 37/8081 | 0.358005244 | 0.817904951 | 0.785145435 | NAT8L | 1 |
| hsa04960 | hsa04960 | Aldosterone-regulated sodium reabsorption | 1/96 | 37/8081 | 0.358005244 | 0.817904951 | 0.785145435 | ATP1A3 | 1 |
| hsa05143 | hsa05143 | African trypanosomiasis | 1/96 | 37/8081 | 0.358005244 | 0.817904951 | 0.785145435 | APOA1 | 1 |
| hsa04928 | hsa04928 | Parathyroid hormone synthesis, secretion and action | 2/96 | 106/8081 | 0.359811147 | 0.817904951 | 0.785145435 | SOST/FGF23 | 2 |
| hsa04922 | hsa04922 | Glucagon signaling pathway | 2/96 | 107/8081 | 0.36410905 | 0.817904951 | 0.785145435 | G6PC/SLC2A2 | 2 |
| hsa04360 | hsa04360 | Axon guidance | 3/96 | 182/8081 | 0.367822934 | 0.817904951 | 0.785145435 | DPYSL5/NTNG1/EPHA8 | 3 |
| hsa04931 | hsa04931 | Insulin resistance | 2/96 | 108/8081 | 0.368396356 | 0.817904951 | 0.785145435 | G6PC/SLC2A2 | 2 |
| hsa04670 | hsa04670 | Leukocyte transendothelial migration | 2/96 | 114/8081 | 0.393878516 | 0.817904951 | 0.785145435 | CTNNA2/CLDN18 | 2 |
| hsa04724 | hsa04724 | Glutamatergic synapse | 2/96 | 114/8081 | 0.393878516 | 0.817904951 | 0.785145435 | GRM1/GRIN2A | 2 |
| hsa00380 | hsa00380 | Tryptophan metabolism | 1/96 | 42/8081 | 0.395419772 | 0.817904951 | 0.785145435 | CYP1B1 | 1 |
| hsa04962 | hsa04962 | Vasopressin-regulated water reabsorption | 1/96 | 44/8081 | 0.409773976 | 0.817904951 | 0.785145435 | AQP4 | 1 |
| hsa02010 | hsa02010 | ABC transporters | 1/96 | 45/8081 | 0.416824081 | 0.817904951 | 0.785145435 | ABCA8 | 1 |
| hsa04510 | hsa04510 | Focal adhesion | 3/96 | 201/8081 | 0.428955555 | 0.817904951 | 0.785145435 | COL2A1/THBS4/TNR | 3 |
| hsa00514 | hsa00514 | Other types of O-glycan biosynthesis | 1/96 | 47/8081 | 0.430675232 | 0.817904951 | 0.785145435 | GALNTL6 | 1 |
| hsa04611 | hsa04611 | Platelet activation | 2/96 | 124/8081 | 0.435291636 | 0.817904951 | 0.785145435 | FGB/FGG | 2 |
| hsa05205 | hsa05205 | Proteoglycans in cancer | 3/96 | 205/8081 | 0.441590426 | 0.817904951 | 0.785145435 | IGF2/FZD10/WNT1 | 3 |
| hsa00600 | hsa00600 | Sphingolipid metabolism | 1/96 | 49/8081 | 0.444200748 | 0.817904951 | 0.785145435 | PSAPL1 | 1 |
| hsa05030 | hsa05030 | Cocaine addiction | 1/96 | 49/8081 | 0.444200748 | 0.817904951 | 0.785145435 | GRIN2A | 1 |
| hsa04340 | hsa04340 | Hedgehog signaling pathway | 1/96 | 50/8081 | 0.450843767 | 0.817904951 | 0.785145435 | LRP2 | 1 |
| hsa05144 | hsa05144 | Malaria | 1/96 | 50/8081 | 0.450843767 | 0.817904951 | 0.785145435 | THBS4 | 1 |
| hsa04015 | hsa04015 | Rap1 signaling pathway | 3/96 | 210/8081 | 0.457236208 | 0.817904951 | 0.785145435 | NGFR/GRIN2A/FGF23 | 3 |
| hsa04913 | hsa04913 | Ovarian steroidogenesis | 1/96 | 51/8081 | 0.457408204 | 0.817904951 | 0.785145435 | CYP1B1 | 1 |
| hsa04068 | hsa04068 | FoxO signaling pathway | 2/96 | 131/8081 | 0.463379863 | 0.817904951 | 0.785145435 | GRM1/G6PC | 2 |
| hsa04961 | hsa04961 | Endocrine and other factor-regulated calcium reabsorption | 1/96 | 53/8081 | 0.470305004 | 0.817904951 | 0.785145435 | ATP1A3 | 1 |
| hsa04024 | hsa04024 | cAMP signaling pathway | 3/96 | 216/8081 | 0.475772039 | 0.817904951 | 0.785145435 | ATP1A3/ADCYAP1R1/GRIN2A | 3 |
| hsa05322 | hsa05322 | Systemic lupus erythematosus | 2/96 | 136/8081 | 0.482944714 | 0.817904951 | 0.785145435 | C7/GRIN2A | 2 |
| hsa01212 | hsa01212 | Fatty acid metabolism | 1/96 | 57/8081 | 0.495195396 | 0.817904951 | 0.785145435 | ACADL | 1 |
| hsa05213 | hsa05213 | Endometrial cancer | 1/96 | 58/8081 | 0.501234933 | 0.817904951 | 0.785145435 | CTNNA2 | 1 |
| hsa05206 | hsa05206 | MicroRNAs in cancer | 4/96 | 310/8081 | 0.505852465 | 0.817904951 | 0.785145435 | CYP1B1/TP63/TNR/TRIM71 | 4 |
| hsa04550 | hsa04550 | Signaling pathways regulating pluripotency of stem cells | 2/96 | 143/8081 | 0.509597471 | 0.817904951 | 0.785145435 | FZD10/WNT1 | 2 |
| hsa05017 | hsa05017 | Spinocerebellar ataxia | 2/96 | 143/8081 | 0.509597471 | 0.817904951 | 0.785145435 | GRM1/GRIN2A | 2 |
| hsa05171 | hsa05171 | Coronavirus disease - COVID-19 | 3/96 | 232/8081 | 0.523739642 | 0.817904951 | 0.785145435 | C7/FGB/FGG | 3 |
| hsa04072 | hsa04072 | Phospholipase D signaling pathway | 2/96 | 148/8081 | 0.528087807 | 0.817904951 | 0.785145435 | GRM1/DGKK | 2 |
| hsa04723 | hsa04723 | Retrograde endocannabinoid signaling | 2/96 | 148/8081 | 0.528087807 | 0.817904951 | 0.785145435 | GRM1/GABRR1 | 2 |
| hsa04932 | hsa04932 | Non-alcoholic fatty liver disease | 2/96 | 150/8081 | 0.53535314 | 0.817904951 | 0.785145435 | LEP/ADIPOQ | 2 |
| hsa03040 | hsa03040 | Spliceosome | 2/96 | 151/8081 | 0.538957469 | 0.817904951 | 0.785145435 | RNU4-2/RNU5A-1 | 2 |
| hsa04927 | hsa04927 | Cortisol synthesis and secretion | 1/96 | 65/8081 | 0.541555553 | 0.817904951 | 0.785145435 | MRAP | 1 |
| hsa05321 | hsa05321 | Inflammatory bowel disease | 1/96 | 65/8081 | 0.541555553 | 0.817904951 | 0.785145435 | IL21 | 1 |
| hsa04020 | hsa04020 | Calcium signaling pathway | 3/96 | 240/8081 | 0.546831823 | 0.817904951 | 0.785145435 | GRM1/GRIN2A/FGF23 | 3 |
| hsa04150 | hsa04150 | mTOR signaling pathway | 2/96 | 155/8081 | 0.553184382 | 0.817904951 | 0.785145435 | FZD10/WNT1 | 2 |
| hsa00830 | hsa00830 | Retinol metabolism | 1/96 | 69/8081 | 0.563129553 | 0.817904951 | 0.785145435 | ADH1B | 1 |
| hsa04924 | hsa04924 | Renin secretion | 1/96 | 69/8081 | 0.563129553 | 0.817904951 | 0.785145435 | ADCYAP1R1 | 1 |
| hsa05031 | hsa05031 | Amphetamine addiction | 1/96 | 69/8081 | 0.563129553 | 0.817904951 | 0.785145435 | GRIN2A | 1 |
| hsa04917 | hsa04917 | Prolactin signaling pathway | 1/96 | 70/8081 | 0.568364146 | 0.817904951 | 0.785145435 | SLC2A2 | 1 |
| hsa05230 | hsa05230 | Central carbon metabolism in cancer | 1/96 | 70/8081 | 0.568364146 | 0.817904951 | 0.785145435 | SLC2A2 | 1 |
| hsa04520 | hsa04520 | Adherens junction | 1/96 | 71/8081 | 0.573536664 | 0.817904951 | 0.785145435 | CTNNA2 | 1 |
| hsa05218 | hsa05218 | Melanoma | 1/96 | 72/8081 | 0.578647835 | 0.817904951 | 0.785145435 | FGF23 | 1 |
| hsa01230 | hsa01230 | Biosynthesis of amino acids | 1/96 | 75/8081 | 0.593620425 | 0.817904951 | 0.785145435 | TKTL1 | 1 |
| hsa04022 | hsa04022 | cGMP-PKG signaling pathway | 2/96 | 167/8081 | 0.594012523 | 0.817904951 | 0.785145435 | ATP1A3/OPRD1 | 2 |
| hsa04971 | hsa04971 | Gastric acid secretion | 1/96 | 76/8081 | 0.598493325 | 0.817904951 | 0.785145435 | ATP1A3 | 1 |
| hsa05133 | hsa05133 | Pertussis | 1/96 | 76/8081 | 0.598493325 | 0.817904951 | 0.785145435 | SFTPA1 | 1 |
| hsa05100 | hsa05100 | Bacterial invasion of epithelial cells | 1/96 | 77/8081 | 0.603308396 | 0.817904951 | 0.785145435 | CTNNA2 | 1 |
| hsa05412 | hsa05412 | Arrhythmogenic right ventricular cardiomyopathy | 1/96 | 77/8081 | 0.603308396 | 0.817904951 | 0.785145435 | CTNNA2 | 1 |
| hsa05164 | hsa05164 | Influenza A | 2/96 | 171/8081 | 0.606998643 | 0.817904951 | 0.785145435 | PRSS1/PRSS2 | 2 |
| hsa04742 | hsa04742 | Taste transduction | 1/96 | 86/8081 | 0.644151201 | 0.825736301 | 0.792663116 | GRM1 | 1 |
| hsa05010 | hsa05010 | Alzheimer disease | 4/96 | 369/8081 | 0.644793055 | 0.825736301 | 0.792663116 | FZD10/GRIN2A/WNT1/DKK4 | 4 |
| hsa04260 | hsa04260 | Cardiac muscle contraction | 1/96 | 87/8081 | 0.648424057 | 0.825736301 | 0.792663116 | ATP1A3 | 1 |
| hsa04540 | hsa04540 | Gap junction | 1/96 | 88/8081 | 0.652646135 | 0.825736301 | 0.792663116 | GRM1 | 1 |
| hsa03013 | hsa03013 | RNA transport | 2/96 | 186/8081 | 0.65292049 | 0.825736301 | 0.792663116 | RNU4-2/RNU5A-1 | 2 |
| hsa04211 | hsa04211 | Longevity regulating pathway | 1/96 | 89/8081 | 0.656818031 | 0.825736301 | 0.792663116 | ADIPOQ | 1 |
| hsa04727 | hsa04727 | GABAergic synapse | 1/96 | 89/8081 | 0.656818031 | 0.825736301 | 0.792663116 | GABRR1 | 1 |
| hsa05032 | hsa05032 | Morphine addiction | 1/96 | 91/8081 | 0.665013636 | 0.825736301 | 0.792663116 | GABRR1 | 1 |
| hsa04062 | hsa04062 | Chemokine signaling pathway | 2/96 | 192/8081 | 0.670073772 | 0.825736301 | 0.792663116 | CCL19/CXCR5 | 2 |
| hsa05022 | hsa05022 | Pathways of neurodegeneration - multiple diseases | 5/96 | 475/8081 | 0.67322054 | 0.825736301 | 0.792663116 | GRM1/FZD10/GRIN2A/WNT1/DKK4 | 5 |
| hsa04657 | hsa04657 | IL-17 signaling pathway | 1/96 | 94/8081 | 0.676945218 | 0.825736301 | 0.792663116 | DEFB4A | 1 |
| hsa04010 | hsa04010 | MAPK signaling pathway | 3/96 | 294/8081 | 0.684556858 | 0.825736301 | 0.792663116 | IGF2/NGFR/FGF23 | 3 |
| hsa04070 | hsa04070 | Phosphatidylinositol signaling system | 1/96 | 97/8081 | 0.68845609 | 0.825736301 | 0.792663116 | DGKK | 1 |
| hsa04925 | hsa04925 | Aldosterone synthesis and secretion | 1/96 | 98/8081 | 0.692202109 | 0.825736301 | 0.792663116 | ATP1A3 | 1 |
| hsa05231 | hsa05231 | Choline metabolism in cancer | 1/96 | 98/8081 | 0.692202109 | 0.825736301 | 0.792663116 | DGKK | 1 |
| hsa04640 | hsa04640 | Hematopoietic cell lineage | 1/96 | 99/8081 | 0.695903549 | 0.825736301 | 0.792663116 | MS4A1 | 1 |
| hsa05146 | hsa05146 | Amoebiasis | 1/96 | 102/8081 | 0.706745617 | 0.828378663 | 0.795199643 | SERPINB13 | 1 |
| hsa04064 | hsa04064 | NF-kappa B signaling pathway | 1/96 | 104/8081 | 0.713760232 | 0.828378663 | 0.795199643 | CCL19 | 1 |
| hsa04625 | hsa04625 | C-type lectin receptor signaling pathway | 1/96 | 104/8081 | 0.713760232 | 0.828378663 | 0.795199643 | CLEC4M | 1 |
| hsa04659 | hsa04659 | Th17 cell differentiation | 1/96 | 107/8081 | 0.723971969 | 0.834141616 | 0.800731773 | IL21 | 1 |
| hsa01200 | hsa01200 | Carbon metabolism | 1/96 | 118/8081 | 0.758423965 | 0.858528221 | 0.824141622 | TKTL1 | 1 |
| hsa04071 | hsa04071 | Sphingolipid signaling pathway | 1/96 | 119/8081 | 0.761336347 | 0.858528221 | 0.824141622 | OPRD1 | 1 |
| hsa04722 | hsa04722 | Neurotrophin signaling pathway | 1/96 | 119/8081 | 0.761336347 | 0.858528221 | 0.824141622 | NGFR | 1 |
| hsa04919 | hsa04919 | Thyroid hormone signaling pathway | 1/96 | 121/8081 | 0.767057273 | 0.858888073 | 0.824487061 | ATP1A3 | 1 |
| hsa04110 | hsa04110 | Cell cycle | 1/96 | 124/8081 | 0.775385148 | 0.862141529 | 0.827610206 | SMC1B | 1 |
| hsa04142 | hsa04142 | Lysosome | 1/96 | 128/8081 | 0.786032299 | 0.864757231 | 0.830121142 | PSAPL1 | 1 |
| hsa04114 | hsa04114 | Oocyte meiosis | 1/96 | 129/8081 | 0.788615085 | 0.864757231 | 0.830121142 | SMC1B | 1 |
| hsa04728 | hsa04728 | Dopaminergic synapse | 1/96 | 132/8081 | 0.796179764 | 0.867072483 | 0.832343661 | GRIN2A | 1 |
| hsa04910 | hsa04910 | Insulin signaling pathway | 1/96 | 137/8081 | 0.80819668 | 0.873215847 | 0.838240965 | G6PC | 1 |
| hsa05162 | hsa05162 | Measles | 1/96 | 139/8081 | 0.812804688 | 0.873215847 | 0.838240965 | CLEC4M | 1 |
| hsa04261 | hsa04261 | Adrenergic signaling in cardiomyocytes | 1/96 | 150/8081 | 0.836257744 | 0.88877116 | 0.853173242 | ATP1A3 | 1 |
| hsa05020 | hsa05020 | Prion disease | 2/96 | 273/8081 | 0.840860328 | 0.88877116 | 0.853173242 | C7/GRIN2A | 2 |
| hsa01240 | hsa01240 | Biosynthesis of cofactors | 1/96 | 156/8081 | 0.847799175 | 0.88877116 | 0.853173242 | NMNAT2 | 1 |
| hsa05160 | hsa05160 | Hepatitis C | 1/96 | 157/8081 | 0.84964287 | 0.88877116 | 0.853173242 | CLDN18 | 1 |
| hsa04530 | hsa04530 | Tight junction | 1/96 | 169/8081 | 0.870115949 | 0.904238143 | 0.868020727 | CLDN18 | 1 |
| hsa05152 | hsa05152 | Tuberculosis | 1/96 | 180/8081 | 0.886446715 | 0.915227452 | 0.878569881 | CLEC4M | 1 |
| hsa05034 | hsa05034 | Alcoholism | 1/96 | 187/8081 | 0.895763117 | 0.918879584 | 0.882075734 | GRIN2A | 1 |
| hsa05130 | hsa05130 | Pathogenic Escherichia coli infection | 1/96 | 197/8081 | 0.907774291 | 0.925231489 | 0.888173227 | CLDN18 | 1 |
| hsa04810 | hsa04810 | Regulation of actin cytoskeleton | 1/96 | 218/8081 | 0.928718795 | 0.940549608 | 0.90287781 | FGF23 | 1 |
| hsa04144 | hsa04144 | Endocytosis | 1/96 | 252/8081 | 0.953096856 | 0.959129115 | 0.920713152 | SH3GL3 | 1 |
| hsa05014 | hsa05014 | Amyotrophic lateral sclerosis | 1/96 | 364/8081 | 0.988338558 | 0.988338558 | 0.948752671 | GRIN2A | 1 |
